# Identification of Novel Variants in Cleft Palate-Associated Genes in Brazilian Patients With Non-syndromic Cleft Palate Only

**DOI:** 10.3389/fcell.2021.638522

**Published:** 2021-07-08

**Authors:** Renato Assis Machado, Hercílio Martelli-Junior, Silvia Regina de Almeida Reis, Erika Calvano Küchler, Rafaela Scariot, Lucimara Teixeira das Neves, Ricardo D. Coletta

**Affiliations:** ^1^Department of Oral Diagnosis, School of Dentistry, University of Campinas (FOP), Piracicaba, Brazil; ^2^Hospital for Rehabilitation of Craniofacial Anomalies, University of São Paulo, Bauru, Brazil; ^3^Stomatology Clinic, School of Dental, State University of Montes Claros, Montes Claros, Brazil; ^4^Center for Rehabilitation of Craniofacial Anomalies, School of Dental, UNIFENAS - Universidade José do Rosario Vellano, Alfenas, Brazil; ^5^Department of Basic Science, Bahiana School of Medicine and Public Health, Salvador, Brazil; ^6^Department of Orthodontics, University of Regensburg, Regensburg, Germany; ^7^Department of Oral and Maxillofacial Surgery, School of Health Science, Federal University of Paraná, Curitiba, Brazil; ^8^Department of Biological Sciences, Bauru School of Dentistry, University of São Paulo (FOB), Bauru, Brazil

**Keywords:** non-syndromic cleft palate only, exome sequence, risk factor, syndrome, oral cleft

## Abstract

The identification of genetic risk factors for non-syndromic oral clefts is of great importance for better understanding the biological processes related to this heterogeneous and complex group of diseases. Herein we applied whole-exome sequencing to identify potential variants related to non-syndromic cleft palate only (NSCPO) in the multiethnic Brazilian population. Thirty NSCPO samples and 30 sex- and genetic ancestry-matched healthy controls were pooled (3 pools with 10 samples for each group) and subjected to whole-exome sequencing. After filtering, the functional affects, individually and through interactions, of the selected variants and genes were assessed by bioinformatic analyses. As a group, 399 variants in 216 genes related to palatogenesis/cleft palate, corresponding to 6.43%, were exclusively identified in the NSCPO pools. Among those genes are 99 associated with syndromes displaying cleft palate in their clinical spectrum and 92 previously related to cleft lip palate. The most significantly biological processes and pathways overrepresented in the NSCPO-identified genes were associated with the folic acid metabolism, highlighting the interaction between LDL receptor-related protein 6 (*LRP6*) and 5-methyltetrahydrofolate-homocysteine methyltransferase (*MTR*) that interconnect two large networks. This study yields novel data on characterization of specific variants and complex processes and pathways related to NSCPO, including many variants in genes of the folate/homocysteine pathway, and confirms that variants in genes related to syndromic cleft palate and cleft lip-palate may cause NSCPO.

## Introduction

Orofacial clefts represent the most common craniofacial malformation in humans with an overall prevalence of approximately 1 per 700 live births, but considerable geographic and ethnic variations exist (Leslie and Marazita, 2013). It may represent the manifestation of a syndrome or as a non-syndromic isolated condition, which corresponds to approximately 70% of all cases (Dixon et al., 2011). Non-syndromic oral clefts (NSOC) comprise two main forms, the cleft lip with or without cleft palate (NSCL ± P) and cleft palate only (NSCPO). Although a multifactorial etiology with a strong genetic component is reported in both NSOC subtypes, many studies have indicated that the genetic factors behind them may be distinct (Mangold et al., 2016; Ludwig et al., 2017). Different genes and chromosomal regions, many identified by genome-wide association studies (GWAS), were described as causal genetic factors for NSCL ± P (Beaty et al., 2016; Yu et al., 2017), but due to lower prevalence, embryological aspects, and recurrence rates, less is known about NSCPO.

To date, five GWAS have been conducted with NSCPO (Beaty et al., 2011; Leslie et al., 2016; Butali et al., 2019; Huang et al., 2019; He et al., 2020). The first studies have identified variants in grainyhead-like transcription factor 3 (*GRHL3*), a gene underlying van der Woude syndrome, the most common genetic syndrome associated with cleft lip and palate, and other few markers that, in interaction with maternal smoking or multivitamin supplementation (gene–environment interactions), were associated with increased odds to NSCPO development (Beaty et al., 2011; Leslie et al., 2016), whereas the most recent GWAS have identified only single-nucleotide polymorphisms (SNPs) in intergenic regions in association with NSCPO (Butali et al., 2019; Huang et al., 2019; He et al., 2020). The study conducted by Butali et al. (2019) has identified the SNP rs80004662 on chromosome 2, near the catenin alpha 2 (*CTNNA2*), as a novel marker for NSCPO in the African population, and the study of Huang et al. (2019) has revealed 11 SNPs in nine loci in genome-wide significance with NSCPO in the Han Chinese population. Four SNPs in a novel locus (15q24.3) were reported in the GWAS conducted by He et al. (2020) with a Han Chinese population of patients with NSCPO. These genetic findings suggest that the effects of polymorphic variants in NSCPO are much less frequent or may be caused by rare variants, which are undetectable in GWAS (Hecht et al., 2002; Koillinen et al., 2005; Ishorst et al., 2018). Moreover, it is possible that specific variants in genes associated with syndromic forms of CPO (SCPO) display a causal effect in the non-syndromic clinical phenotype, and the NSCPO may be the result of genetic factors associated with clefts involving both lip and palate (CLP). A previous study supports this hypothesis; out of 350 genes associated with NSCPO, 177 were also identified in patients with CLP and 28 in patients with SCPO (Funato and Nakamura, 2017).

Whole-exome sequencing (WES) has been applied for the genetic characterization of NSCPO (Bureau et al., 2014; Pengelly et al., 2016; Liu et al., 2017; Hoebel et al., 2017; Basha et al., 2018). The study performed by Liu et al. (2017) has identified a rare variant (p.Ser552Pro) in Rho GTPase activating protein 29 (*ARHGAP29*) in a family spanning NSCPO under the assumption of an autosomal dominant of inheritance. Applying WES in multiple affected NSCPO pedigrees and validation of the potential deleterious variants in a case–control approach, Hoebel et al. (2017) have suggested the participation of acetyl-CoA carboxylase beta (*ACACB*), CREB-binding protein (*CREBBP*), *GRHL3*, MIB E3 ubiquitin protein ligase 1 (*MIB1*), and protein tyrosine phosphatase receptor type S (*PTPRS*) in the etiology of NSCPO. In the study conducted by Basha et al. (2018), the c.819_820dupCC and c.3373C > T mutations in tumor protein P63 (*TP63*) and LDL receptor-related protein 6 (*LRP6*), respectively, were described in two families with NSCPO. On the other hand, the studies conducted by Bureau et al. (2014); Pengelly et al. (2016) have analyzed multiplex families of patients with NSOC, including NSCPO among those affected, which makes more difficult to understand the exact participation of genes identified in the pathogenesis of NSCPO.

Large-scale genetic studies have confirmed the genetic contribution to the etiology of NSCL ± P but have also demonstrated that these defects can result from variation in multiple genes (Carlson et al., 2019). However, much less is known in terms of genetic etiology of NSCPO. Furthermore, most patients included in the genome-wide studies, including GWAS and genomic sequencing, have been of European or Asian ancestry, and the genetic predisposition to NSOC is ethnicity-dependent, e.g., the association of 8q24 is consistently observed in European patients with NSCL ± P, but much less among patients with Asian ancestry or in Brazilians with high African ancestry (do Rego et al., 2015; Wattanawong et al., 2016). In this context, it is quite important to perform large-scale genetic studies in different populations to define common and ethnic-specific risk genes, but the costs of those assays in a large number of participants are still challenging. To genomic sequencing, pool sequencing (mix DNA samples from several patients prior to sequencing) has proved to be an effective alternative to overcome the problems related to costs (Collins et al., 2016; Popp et al., 2017). In the current study, we applied whole-exome pool sequencing to characterize genetic variants related to NSCPO in the multiethnic Brazilian population. This is the first study to perform WES in Brazilian patients with NSCPO, and our findings revealed a large list of novel variants in previously CPO and CLP-associated genes as well in genes not related to oral clefts.

## Materials and Methods

### Study Participants

A total of 30 samples from patients with NSCPO and 30 healthy controls were recruited after approval by the Institutional Review Boards at the Center for Rehabilitation of Craniofacial Anomalies, University of José Rosário Vellano, Alfenas, Minas Gerais, and at the Santo Antonio Hospital, Salvador, Bahia. The patients with NSCPO were carefully investigated by specialist teams of the two Centers for the occurrence of associated abnormalities or syndromes, and only unrelated patients with complete NSCPO, without any other congenital malformation or mental disability, were included. The control group consisted of unrelated healthy patients, without congenital malformations, mental disorders, or family history of orofacial clefts, living in the same geographic areas. Cases and controls were unrelated newborn infants or young children. Supplementary Table 1 depicts the main characteristics of the participants of this study, including sex and proportion of genomic ancestry.

### Samples and Pooling Strategy

**FIGURE 1:**
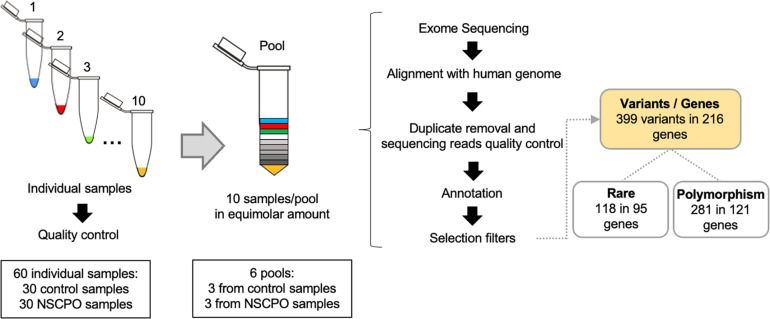
Workflow of whole-exome pool sequencing applied to 30 samples of patients with non-syndromic cleft palate only (NSCPO) and 30 healthy controls. After sequencing and bioinformatic analyses, 399 variants distributed in 216 different genes were found, with 118 rare variants in 95 genes and 281 polymorphisms in 121 genes.

Genomic DNA was isolated from oral mucosa cells, obtained by mouthwash with a 3% sucrose solution or by scraping the buccal mucosa with a swab, using a salting-out protocol (Aidar and Line, 2007). The concentration and quality of DNA samples were assessed by spectrophotometry and agarose gel electrophoresis. Each pool represented 10 samples, and the amount of DNA was adequately balanced to represent each genome equally (Figure 1). After mixing the samples, the pools were quantified using the fluorometric method (Qubit^®^, Thermo Fisher Scientific, Waltham, MA, United States) and normalized to 10 mM Tris–EDTA buffer pH 8.0 at a final concentration of 5 ng/μl. To ensure greater homogeneity, each pool was composed of patients of the same sex (two pools with females and one pool with males for each group) and from the same geographic location. In addition, the genomic ancestry was assessed in each sample with a 40 biallelic short insertion–deletion polymorphism panel, which was previously validated as ancestry informative of the Brazilian population (Messetti et al., 2017), and the averages of contributions from European, African, and Amerindian ancestries among pools were similar (Supplementary Table 1).

### WES and Bioinformatic Analyses

The pools were enriched in libraries with the Nextera^TM^ DNA sample preparation kit (Illumina, San Diego, CA, United States), and each library was quantified by RT-qPCR using the KAPA Library Quantification Kit (KAPA Biosystems Inc., Wilmington, MA, United States). The libraries were diluted to a concentration of 16 pM and pooled by cBOT (Illumina) using the TruSeq PE cluster v3-cBOT-HS Kit (Illumina). Paired sequencing, with a reading length of 100 bp, was performed on the HiSeq 1000 equipment (Illumina), aiming at covering 18 × /pool. Following the sequencing, the sequences of each pool (FASTQ files) were aligned with the human reference genome RCh38/hg19 through the Burrows–Wheeler aligner program [BWA, GNU General Public License version 3.0 (GPLv3), MIT License, Cambridge, MA, United States] (Li and Durbin, 2009). The duplicates were removed, and the quality of the sequencing was verified with the GATK program (Genome Analysis Toolkit). The coverage depth was exported in a BAM file with the Biobambam2 program^1^, and the variants were identified as VCF files with the Freebayes program^2^. The BAM and VCF files for each pool were incorporated into the VarSeq^®^ program (version 1.4.8; Golden Helix, Inc., Bozeman, MT, United States) for genetic variant annotation according to the public databases 1000 Genomes, GnomAD Exome, GnomAD Genome, and ExAC. A total of 102,927 variants were identified.

The selection of the variants of interest started with the exclusion of sequences that did not pass the quality control (coverage ≥ 20 and sequencing depth ≥ 100). After applying this filter, 61,398 variants were maintained. Next, the variants present in the control group were excluded, leaving only the variants contained in the pools of patients with NSCPO. The variants that did not show an allele frequency of at least 5% in the pool were also excluded, where in a universe of 20 possible alleles in each pool, at least one allele should be evidenced. The application of these filters reduced the number of variants to 5,129 variants in 3,360 genes. At this point, the GATACA database^3^, VarElect database^4^, and Online Mendelian Inheritance in Man (OMIM)^5^ were used for identification of genes associated with cleft palate, including non-syndromic, syndromic, and CLP. The resulting list of genes was compared to 3,360 genes found in the exome (Figure 1). The polymorphism phenotyping (PolyPhen2^6^) (Adzhubei et al., 2013), sorting intolerant from tolerant (SIFT^7^) (Kumar et al., 2009), and mutation taster^8^ were used for biological and functional effect prediction of the variants. STRING, protein–protein interaction networks functional enrichment analysis,^9^ was used to investigate the functional significance of genes, including biological processes, pathways and interaction network. The *p*-Values were subjected to false discovery rate to correct multiple tests, and values ≤ 0.05 were considered significant.

## Results

Out of 3,360 cleft palate-related genes, 216 (6.43%) displayed at least a variation in the pools of NSCPO. Since phenotypic human gene classification also offers major insights into gene function (Frech and Chen, 2010), the identified genes were categorized on the basis of their previous association with NSCPO (Table 1), SCPO (Supplementary Table 2), and CLP (Supplementary Table 3). Supplementary Table 4 depicts the variants identified in the pools of patients with NSCPO, but not located in the genes previously associated with palatogenesis or oral clefts.

**TABLE 1:** Novel variants in genes previously described in association with non-syndromic cleft palate only (NSCPO) found exclusively in the samples of patients with NSCPO.

**Gene**	**Protein**	**Variants**	**Chr:Pos**	**MAF 1K**	**MAF gnomAD Exomes**	**MAF gnomAD Genomes**	**MAF ExAC**	**MAF ABraOM**	**Sequence Ontology**	**SIFT**	**Polyphen2**	**Mutation Taster**	**OMIM Number**	**References**
***ACACB***	Acetyl-CoA Carboxylase Beta	rs60293430	12:109665242	0.0517173	0.0479439	0.0660566	0.049	0.066183	missense	Tolerated	Possibly damaging	Damaging	*601557	Hoebel et al., 2017
***CDH1*^†^**	Cadherin 1	rs33969373	16:68856088	0.0227636	0.0103762	0.0177832	0.011	0.038588	synonymous	-	-	-	*192090	Song and Zhang, 2011
		rs33964119	16:68862165	0.0545128	0.0426288	0.0346343	0.04	0.037767	synonymous	-	-	-		
COL2A1*	Collagen Type II Alpha 1 Chain	rs34392760	12:48391657	0.0179712	0.0376268	0.0349919	0.038	0.047619	missense	Tolerated	Benign	Damaging	+120140	Li et al., 1995; Nikopensius et al., 2010
COL11A1*	Collagen Type XI Alpha 1 Chain	rs1012281	1:103427407	0.038139	0.0516874	0.0579359	0.053	0.068144	intron	-	-	-	*120280	Seegmiller et al., 1971
		rs771593293	1:103468856	-	-	-	-	0.087800	-	-	-	-		
		rs111841420	1:103470191	0.0183706	0.0245013	0.022501	0.023	0.025452	synonymous	-	-	-		
		rs3767272	1:103483329	0.0347444	0.0296967	0.0200607	0.03	0.026273	intron	-	-	-		
COL11A2*	Collagen Type XI Alpha 2 Chain	rs2229784	6:33136310	0.057508	0.0344903	0.0395325	0.034	0.051724	missense	Damaging	Possibly damaging	Damaging	*120290	Nikopensius et al., 2010
		rs73741526	6:33139635	0.0810703	0.0464905	0.0476175	0.047	0.064860	intron	-	-	-		
		rs2855430	6:33141280	0.102236	0.121898	0.106553	0.122	0.084565	missense	Tolerated	Probably damaging	Damaging		
		rs9280359	6:33142013	0.202476	0.162732	0.189819	0.1	0.178053	intron	-	-	-		
		rs41317098	6:33144466	0.102436	0.0473386	0.115006	0.052	0.077997	intron	-	-	-		
		rs2744507	6:33148878	0.102636	0.121967	0.106294	0.122	0.085386	intron	-	-	-		
CREBBP*	CREB Binding Protein	rs73491901	16:3781845	0.00139776	0.0003691	0.0021309	0.0004942	0.001281	missense	Tolerated	Possibly damaging	Damaging	*600140	Hoebel et al., 2017
		rs130004	16:3828207	0.0553115	0.0137934	0.0517921	0.017	0.027754	intron	-	-	-		
		rs130018	16:3831187	0.0780751	0.0281749	0.069947	0.033	0.052519	intron	-	-	-		
***FTCD*^†^**	Formimidoyltransferase Cyclodeaminase	rs10432965	21:47557222	0.167532	0.0830084	0.0992947	0.087	0.091954	synonymous	Damaging	Benign	Damaging	*606806	Boyles et al., 2009
GRHL3^*,†^	Grainyhead Like Transcription Factor 3	rs34637004	1:24663184	0.0175719	0.0343092	0.0398771	0.033	0.033662	missense	Tolerated	Benign	Tolerated	*608317	Peyrard-Janvid et al., 2014; Leslie et al., 2016; Basha et al., 2018
***JAG2*^†^**	Jagged Canonical Notch Ligand 2	rs78862296	14:105634205	0.00519169	0.00102047	0.0052073	0.001451	0.002467	synonymous	-	-	-	*602570	Jiang et al., 1998; Vieira et al., 2005; Jagomagi et al., 2010
		rs113906438	14:105613039	0.023762	0.0125495	0.0139761	0.011	0.018883	synonymous	-	-	-		
LRP6^*,†^	LDL Receptor Related Protein 6	rs34815107	12:12279735	0.00299521	0.00103964	0.0041338	0.001285	0.002463	missense	Tolerated	Bening	Damaging	*603507	Basha et al., 2018
***MSX1*^†^**	Msh Homeobox 1	rs33929633	4:4864405	0.189696	0.216977	0.234219	0.215	0.206076	intron	-	-	-	*142983	Satokata and Maas, 1994; Jagomagi et al., 2010
***MTHFD1*^†^**	Methylenetetrahydrofolate Dehydrogenase, Cyclohydrolase And Formyltetrahydrofolate Synthetase 1	rs60806768	14:64898459	0.0593051	0.0315681	0.0640463	0.036	0.047619	intron	-	-	-	*172460	Boyles et al., 2008; Murthy et al., 2014
		rs59770063	14:64898463	0.0593051	0.0315598	0.0639411	0.036	0.047619	intron	-	-	-		
***MTHFR*^†^**	Methylenetetrahydrofolate Reductase	rs45496998	1:11852412	-	-	-	-	0.002463	-	-	-	-	*607093	Shaw et al., 1999
		rs2274976	1:11850927	0.0744808	0.055472	0.0408481	0.056	0.033662	missense	Tolerated	Benign	Tolerated		
***MTR*^†^**	5-Methyltetrahydrofolate-Homocysteine Methyltransferase	rs113277607	1:236971983	0.0421326	0.0113392	0.0400116	0.015	0.032020	intron	-	-	-	*156570	Boyles et al., 2008
		rs7526063	1:236971998	0.0744808	0.0374526	0.051899	0.04	0.049261	splice region	-	-	-		
		rs12022937	1:237060850	0.215056	0.0764726	0.146143	0.085	0.113300	splice region	-	-	-		
OFD1*	OFD1 Centriole And Centriolar Satellite Protein	rs5979959	X:13769452	0.00503311	0.00163061	0.00527241	0.001738	0.002994	synonymous	-	-	-	*300170	Ferrante et al., 2006; Jugessur et al., 2012
		rs140369491	X:13779341	0.0362914	0.0130494	0.0185598	0.015	0.022954	intron	-	-	-		
POMGNT2*	Protein O-Linked Mannose N-Acetylglucosaminyl transferase 2 (Beta 1,4-)	rs115870061	3:43122162	0.00499201	0.00106109	0.00432956	0.001491	0.00243	synonymous	-	-	-	*614828	Huang et al., 2019
***RFC1*^†^**	Replication Factor C Subunit 1	rs2066782	4:39303925	0.106829	0.115582	0.0985123	0.115	0.096880	synonymous	-	-	-	*600424	Shaw et al., 2003; Boyles et al., 2008, 2009
***SHMT1***	Serine Hydroxymethyltransferase 1	rs8080285	17:18234028	0.0924521	0.026609	0.0748126	0.031	0.068966	intron	-	-	-	*182144	Boyles et al., 2009
		rs2273026	17:18256979	0.135184	0.122439	0.109661	0.119	0.103448	splice region	-	-	-		
SOX9*	SRY-Box Transcription Factor 9	rs2229989	17:70118935	0.136581	0.193679	0.172156	0.192	0.157635	synonymous	-	-	-	*608160	Bi et al., 2001
TBX1*	T-Box Transcription Factor 1	rs139776757	22:19750797	0.000998403	0.0028023	0.0040708	0.003031	0.003284	synonymous	-	-	-	*602054	Basha et al., 2018
TBX22*	T-Box Transcription Factor 22	rs195293	X:79283509	0.101457	0.0254122	0.0901992	0.029	0.092814	synonymous	-	-	-	*300307	Pauws et al., 2009
***TCN2*^†^**	Transcobalamin 2	rs2283873	22:31013296	0.167133	0.0871466	0.1164	0.087	0.100985	intron	-	-	-	*613441	Martinelli et al., 2006
		rs9621049	22:31013419	0.108626	0.114338	0.127767	0.112	0.148604	missense	Tolerated	Benign	Tolerated		
TCOF1*	Treacle Ribosome Biogenesis Factor 1	rs73270831	5:149740772	0.00958466	0.00245265	0.00910853	0.003006	0.008210	synonymous	-	-	-	*606847	Dixon et al., 2000; Machado et al., 2016
		rs73270846	5:149756088	0.0091853	0.00238518	0.00898746	0.0029	0.008210	missense	Tolerated	Possibly damaging	Tolerated		
		rs2071240	5:149755744	0.0605032	0.0693377	0.0643264	0.069	0.045977	missense	Damaging	Probably damaging	Tolerated		
		rs11743855	5:149772932	0.0623003	0.0638	0.0878194	0.065	0.086207	splice region	-	-	-		
TP63*	Tumor Protein P63	rs33979049	3:189584563	0.0145767	0.0164723	0.0142811	0.017	0.012315	synonymous	-	-	-	*603273	Basha et al., 2018
***TYMS*^†^**	Thymidylate Synthetase	rs28602966	18:658170	0.0181709	0.027113	0.0262783	0.022	0.024752	stop gained	-	-	-	*188350	Boyles et al., 2009; Shaw et al., 2013

*Genes described in syndromes with cleft palate. *^†^*Genes described in cleft lip and palate. OMIM, Online Mendelian Inheritance in Man (http://omim.org). An asterisk (*) before an OMIM entry number indicates a gene. A plus sign (+) before an OMIM entry number indicates that the entry includes a description of a gene and a phenotype.

**FIGURE 2:**
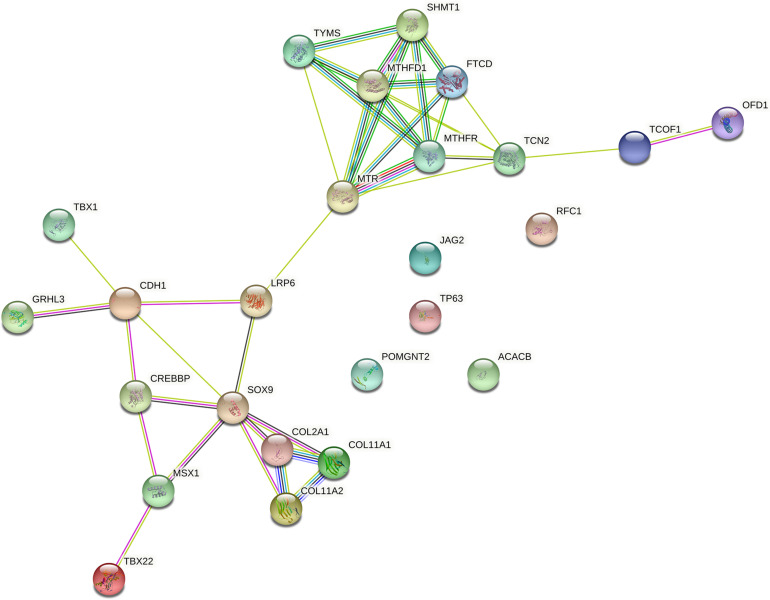
Protein–protein interaction network with the genes associated with non-syndromic cleft palate only (NSCPO). Twenty out of 25 genes formed two nodes interconnected by a validated interaction between *LRP6* and *MTR*. The first encompassed nine genes, including *FTCD*, *MTHFD1*, *MTHFR*, *MTR*, *OFD1*, *SHMT1*, *TCN2*, *TCOF1*, and *TYMS* (*P* < 1.0e-16), and the second node involved *CDH1*, *COL2A1*, *COL11A1*, *COL11A2*, *CREBBP*, *GRHL3*, *LRP6*, *MSX1*, *SOX9*, *TBX1*, and *TBX22* (*P* < 1.0e-16). Both nodes were based on curated databases (light blue), experimentally (purple), gene neighborhood (green), gene fusions (red), gene co-occurrence (blue), text mining (light green), co-expression (black), and protein homology (violet). This analysis had an average confidence score of 0.588 (*P* < 1.0e-16), suggesting a low rate for false-positive interactions.

Among genes previously described in association with NSCPO, 38 novel SNPs (except rs2229989 in *SOX9*, Jia et al., 2017) and 9 novel rare variants were identified in *ACACB*, *CDH1*, *COL11A1*, *COL11A2*, *COL2A1*, *CREBBP*, *FTCD*, *GRHL3*, *JAG2*, *LRP6*, *MSX1*, *MTHFD1*, *MTHFR*, *MTR*, *OFD1*, *POMGNT2*, *RFC1*, *SHMT1*, *SOX9*, *TBX1*, *TBX22*, *TCN2*, *TCOF1*, *TP63*, and *TYMS* (Table 1). The biological processes and pathways for these NSCPO-associated genes were generated by STRING (Table 2 and Supplementary Table 5). The most significant biological processes were skeletal system development (GO:0001501, *P* = 1.96e-10), tetrahydrofolate interconversion (GO:0035999, *P* = 3.57e-09), and pteridine-containing compound metabolic process (GO:0042558, *P* = 3.88e-09) (Supplementary Table 5), and the pathways were of one carbon pool by folate (hsa00670, *P* = 3.63e-11) and antifolate resistance (hsa01523, *P* = 0.00038) (Table 2). Two main networks interconnected by interactions between *LRP6* and *MTR* were identified in NSCPO-associated genes (Figure 2).

**TABLE 2:** List of the overrepresented pathway formatted with the variant-containing-genes identified in patients with non-syndromic cleft palate only (NSCPO).

**Pathway ID**	**Term description**	**Observed gene count**	**Background gene count**	**False discovery rate**	**Matching proteins in your network**
hsa00670*	One carbon pool by folate	6	20	3.63e-11	MTHFD1, FTCD, TYMS, SHMT1, MTR, MTHFR
hsa01523*	Antifolate resistance	3	31	0.00038	TYMS, SHMT1, MTHFR
**hsa01100**	Metabolic pathways	8	1250	0.0027	MTHFD1, FTCD, TYMS, SHMT1, ACACB, POMGNT2, MTR, MTHFR
**hsa04974**	Protein digestion and absorption	3	90	0.0039	COL11A1, COL11A2, COL2A1
**hsa04330**	Notch signaling pathway	2	48	0.0262	CREBBP, JAG2
hsa01230*	Biosynthesis of amino acids	2	72	0.0460	SHMT1, MTR
**hsa04520**	Adherens junction	2	71	0.0460	CDH1, CREBBP
**hsa05200**	Pathways in cancer	4	515	0.0460	LRP6, CDH1, CREBBP, JAG2

*^∗^Pathway found only in NSCPO.*

Variations (*n* = 192) in 99 SCPO-associated genes were found in NSCPO samples, but not in the control (Supplementary Table 2). These genes participate in 437 biological processes and 31 pathways. The most significantly represented GO terms for biological processes and cellular component ontologies for the encoded proteins were developmental process (GO:0032502, *P* = 6.42e-12), anatomical structure development (GO:0048856, *P* = 6.42e-12), and multicellular organism development (GO:0007275, *P* = 8.60e-12) (Supplementary Table 6), and the pathways were thyroid hormone signaling (hsa04919, *P* = 0.00047), protein digestion and absorption (hsa04974, *P* = 0.00077), and proteoglycans in cancer (hsa05205, *P* = 0.0041) (Supplementary Table 7). The network involved in the SCPO-associated genes included 205 interactions (Supplementary Figure 1).

Since CLP results from failure of fusion of lip and palate, we have also looked into variations of genes related to CLP, and 160 variants in 92 genes were identified (Supplementary Table 3). Together, the CLP-associated genes identified exclusively in the NSCPO pools participate in 873 biological processes and 68 pathways, with the most significant being animal organ development (GO:0048513, *P* = 2.24e-20) (Supplementary Table 8) and human papillomavirus infection (hsa05165, *P* = 3.58e-06) (Supplementary Table 9), respectively. A complex network characterized by 244 interactions among these CLP-associated genes identified exclusively in the NSCPO pools was observed (Supplementary Figure 2).

**FIGURE 3:**
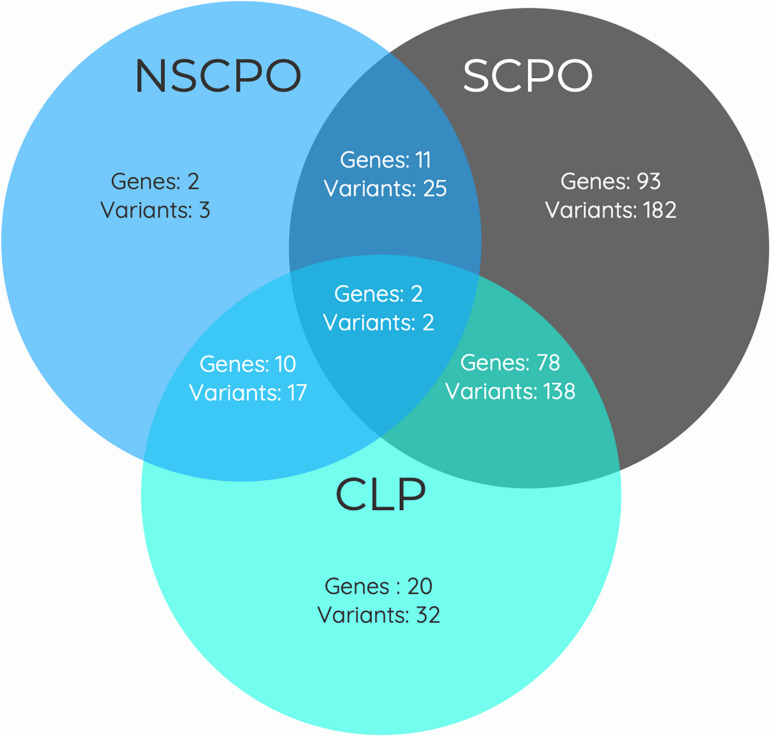
Venn diagram showing the overlap between genes associated with the phenotypes of non-syndromic cleft palate only (NSCPO), syndromic cleft palate only (SCPO), and cleft lip and palate (CLP) found in patients with NSCPO. The numbers in each area represent the gene and variants count for the particular section.

As some genes were exclusive to the NSCPO phenotype and others coincided with the SCPO and CLP phenotypes (Figure 3), we investigated whether biological processes and pathways differ among genes related to NSCPO, SCPO, and CLP, and we compared the biological processes and pathways to identify those ones associated with NSCPO only. Although most genes/pathways are interrelated, 76 biological processes were enriched only for NSCPO-associated genes in the pools of NSCPO, and the most significant were tetrahydrofolate interconversion (GO:0035999, *P* = 3.57e-09) and pteridine-containing compound metabolic process (GO:0042558, *P* = 3.88e-09) (processes indicated with an ^∗^ on Supplementary Table 5). In addition, the pathways of one carbon pool by folate (hsa00670, *P* = 3.63e-11), antifolate resistance (hsa01523, *P* = 0.00038), and biosynthesis of amino acids (hsa01230, *P* = 0.0460), formatted by *FTCD*, *MTHFD1*, *MTHFR*, *MTR*, *SHMT1*, and *TYMS*, were found only in NSCPO-associated genes (pathways indicated with an ^∗^ on Table 2).

## Discussion

Although the palatogenesis is a complex process with involvement of many genes and cellular pathways, genetic variants in association with cleft palate have been identified in only a limited number of these genes. In the present study, after exome sequencing and bioinformatic analyses, we found 399 variants in 216 genes related to palatogenesis/cleft palate exclusively in the DNA pool of patients with NSCPO. Among variations in the 25 NSCPO-associated genes (Table 1), 13 are described in association with syndromes displaying cleft palate in the clinical spectrum (SCPO-associated genes) and 13 were previously associated with CLP. Therefore, variants in *ACACB* and *SHMT1* belonged exclusively to NSCPO. *ACACB*, encoding the acetyl-coenzyme-A-carboxylase β, an enzyme involved in the oxidation of fatty acids (Ma et al., 2011), was recently reported as a potential candidate for NSCPO by exome sequencing (Hoebel et al., 2017). *SHMT1* (serine hydroxymethyltransferase), located at 17p11.2, encodes the cytoplasmic SHMT enzyme consisting of 483 residues (Garrow et al., 1993). Although Shmt1 knockout mice are viable and fertile (MacFarlane et al., 2008), the incidence of neural tube defects among the offspring of Shmt1^–/–^ females is significantly affected by a low-folate diet (Beaudin et al., 2012). Previous studies have also associated SNPs in *SMHT1* with NSCPO in a Norwegian population (Boyles et al., 2008), and with NSCL ± P risk in a Chilean population (Salamanca et al., 2020).

Our analyses also revealed novel variants in *GHRL3* and *LRP6*. *GRHL3* encodes a transcription factor member of the grainyhead family with significant roles during the embryogenesis of craniofacial structure (Dworkin et al., 2014; Peyrard-Janvid et al., 2014). *GRHL3* mutations were reported in patients with VWS (Peyrard-Janvid et al., 2014) and human spina bifida (Lemay et al., 2017), and polymorphisms were associated with both NSCL ± P and NSCPO phenotypes (Leslie et al., 2016; Mangold et al., 2016; Wang Y. et al., 2016; Eshete et al., 2018). In a recent study, we analyzed the previous *GRHL3*-associated variants (rs10903078, rs41268753, and rs4648975) in a large ancestry-structured case–control sample composed of 1,127 Brazilian participants, and none of the variants withstood the multiple logistic regression analysis with *p*-Values adjusted to multiple comparisons (Azevedo et al., 2020). As the common *GRHL3* variants identified as risk factors for NSCPO in European, Asian, and African populations (Leslie et al., 2016; Mangold et al., 2016; Wang Y. et al., 2016) were not confirmed in our previous study, it is suggested that NSCPO risk associated with particular variants in *GRHL3* may differ among ethnic groups and the variant identified in this study (rs34637004) may be the risk variant for the Brazilian population. *LRP6*, which encodes a central co-receptor in the WNT/β-catenin signaling pathway, has pleiotropic roles in both normal development and complex diseases (Wang et al., 2018). LRP6 controls the canonical WNT/β-catenin pathway promoting development of neural crests and participating in the craniofacial morphogenesis, including lip and palate formation (Tamai et al., 2000; Song et al., 2009; Jin et al., 2011). Variants in *LRP6* were associated with congenital neural tube defects (Allache et al., 2014; Lei et al., 2015), tooth agenesis (Massink et al., 2015; Ockeloen et al., 2016), and NSOC (Basha et al., 2018). Although the *LRP6*-variant rs34815107 (c.4202G > A; p.Arg1401His; MAF < 0.002) was predicted as benign and tolerated and to date no disease was associated with this variant, it is located in the LRP6 cytoplasmic domain, which is essential for Wnt phosphorylation of serine/threonine residues of target proteins (Chen et al., 2014). Further validation in large multiethnic cohort studies and functional analysis are warranted to clarify the association between the novel variants in *GRHL3* and *LRP6* and NSCPO.

Using ontology analysis, we found 243 biologic processes and 8 pathways formatted by NSCPO-associated genes that interact with each other constituting a large network with two large nodes interconnected by *LRP6* and *MTR*. *MTR* encodes the methionine synthase protein, which catalyzes the remethylation of homocysteine to form methionine and links of homocysteine cycle to folate metabolism (Leclerc et al., 1996). Strong evidences suggest that the presence of accepted folate/homocysteine levels may not be the only requirement for NSOC prevention, since its absorption, transport, and metabolism, producing active derivatives essentials for DNA methylation and synthesis during embryonic processes, including craniofacial development, are also important to oral cleft predisposing (Desai et al., 2016). Indeed, variants in several genes related to transport and metabolism of folate and homocysteine, including *MTR*, have been shown to alter the disponibility of the active forms of those molecules and influence cleft risk (Wang W. et al., 2016; Lei et al., 2018). Interestingly, a recent study demonstrated that the methionine-depleted medium inhibits canonical Wnt/β-catenin signaling (Albrecht et al., 2019), and as LRP6 is an indispensable co-receptor for WNT signaling (Gray et al., 2010), our findings reinforce the interlink between *MTR* and *LRP6* and the risk of NSCPO through potential epistatic interactions. Furthermore, as cleft palate can be part of a syndrome or concomitant with cleft lip, indicating that variants in multiple genes may be shared by different conditions with a specific cleft palate phenotype, we applied genetic ontology analysis to identify common and specific pathways. We found 167 biological processes and 5 pathways consisting of *ACACB*, *CDH1*, *COL2A1*, *COL11A1*, *COL11A2*, *CREBBP*, *FTCD*, *LRP6*, *JAG2*, *MTHFD1*, *MTHFR*, *MTR*, *POMGNT2*, *SHMT1*, and *TYMS* enriched in SCPO and CLP. In relation to NSCPO, 76 biological processes and 3 pathways, led by *FTCD*, *MTHFD1*, *MTHFR*, *MTR*, *SHMT1*, and *TYMS*, were found. Thus, these findings suggest that although the genes form distinct biological processes and pathways, similar genes participated in both phenotypes, suggesting common etiologies among them.

The DNA sequencing of pooled patients proved to be an excellent and cost-effective strategy to identify genetic variants related to complex diseases (Collins et al., 2016; Popp et al., 2017; Özdoğan and Kaya, 2020), although it has never been applied to study oral clefts. We detected variants in 6.43% of the investigated genes, which is quite similar to the number of variants identified in the study conducted by Basha et al. (2018), applying individual exome sequencing in 46 multiplex families with NSCPO. Furthermore, we found variants in the same genes identified and validated by Basha et al. (2018) as of risk for NSCPO. However, this technique is associated with problems to be considered when analyzing and interpreting the results. One important problem is to correctly identify rare variants, since sequencing errors can be confused with alleles present at low frequencies, generating false-positive variants. Another potential problem is associated with the estimation of the allelic frequency of the polymorphic variants. The power of genetic analysis depends on the calculation of the allele frequency, and in principle, the pool should provide a robust estimate of the allele frequencies, since it represents several samples, decreasing the general variances of the estimated frequencies. This hypothesis is well supported by mathematical models under the assumption that there are no sequencing errors and each individual contributes an equal amount of DNA to the pools (Anand et al., 2016). Therefore, the lack of validation in a large cohort and the non-characterization of the functional impact of the variants are the limitations of this study. Another limitation that should be noted when interpreting the results is that we did not control for environmental risk factors, although the major source of confounding for genetic studies is population stratification, and our results were adjusted for this.

In summary, our results support the findings of earlier epidemiological studies, indicating that specific variants in genes related to SCPO and CLP may cause NSCPO. Furthermore, gene ontology enrichment analysis identified the interconnection of *LRP6* and *MTR*, reinforcing that variants in the folate/homocysteine pathway may influence risk of NSCPO through potential epistatic interactions. While this study has explored novel variants in previously reported cleft palate genes, we have also identified and described a large group of variants in genes without previous association with NSCPO.

## Data Availability Statement

The datasets presented in this study can be found in online repositories. The name of the repository and accession number are as follows: European Nucleotide Archive PRJEB44884 and the samples (SAMEA8730854, SAMEA8730858, SAMEA8730857, SAMEA8730856, SAMEA8730855, SAMEA8730853).

## Ethics Statement

The studies involving human participants were reviewed and approved by the Piracicaba Dental School, University of Campinas – 08452819.0.0000.5418. Written informed consent to participate in this study was provided by the participants’ legal guardian/next of kin.

## Author Contributions

RM contributed to the conception, design, data acquisition, and interpretation, and drafted and critically revised the manuscript. HM-J, SR, EK, RS, and LN contributed to conception, design, data acquisition, and interpretation, and critically revised the manuscript. RC made substantial contributions to the study’s conception and design, and manuscript review and editing. All authors gave their final approval and agreed to be accountable for all aspects of the work.

## Conflict of Interest

The authors declare that the research was conducted in the absence of any commercial or financial relationships that could be construed as a potential conflict of interest.

## Footnotes

1 https://scfbm.biomedcentral.com/articles/10.1186/1751-0473-9-13

2 https://arxiv.org/abs/1207.3907

3 https://gataca.cchmc.org/gataca/

4 http://varelect.genecards.org

5 http://omim.org

6 http://genetics.bwh.harvard.edu/pph2/

7 http://sift.jcvi.org/

8 http://www.mutationtaster.org/

9 http:/string-db.org

## Supplementary Material

Supplementary Figure 1 Protein-protein interaction networks constructed with the previously syndromic cleft palate only (SCPO)-associated genes identified in the pools of patients with non-syndromic cleft palate only (NSCPO), but not in the control, using STRING. The networks include 205 edges (interactions) among 98 nodes based on curated databases (light blue), experimentally (purple), gene neighborhood (green), gene fusions (red), gene co-occurrence (blue), textmining (light green), co-expression (black) and protein homology (violet). The overall confidence score of this analysis was 0.446 (*P* < 1.0e-16), indicating a low change for false-positive interactions.

Supplementary Figure 2 The protein-protein interaction networks with cleft lip-palate (CLP)-associated targets found in the pools of patients with non-syndromic cleft palate only (NSCPO), but not in the control. Networks include 244 interactions among 91 nodes (overall coefficient score = 0.557; *P* < 1.0e-16). Different colors represent different levels of evidence of connection between proteins. Light blue represents curated databases, purple experimental evidence, green gene neighborhood, red gene fusions, blue gene co-occurrence, light green evidence from textmining, black co-expression, and violet protein homology.
