# Supplementary figures and images for: Identification of Novel Variants in Cleft Palate-Associated Genes in Brazilian Patients With Non-syndromic Cleft Palate Only
Source: Front Cell Dev Biol. 2021 Jul 8;9:638522. doi: 10.3389/fcell.2021.638522 (PMC8297955; doi:10.3389/fcell.2021.638522)

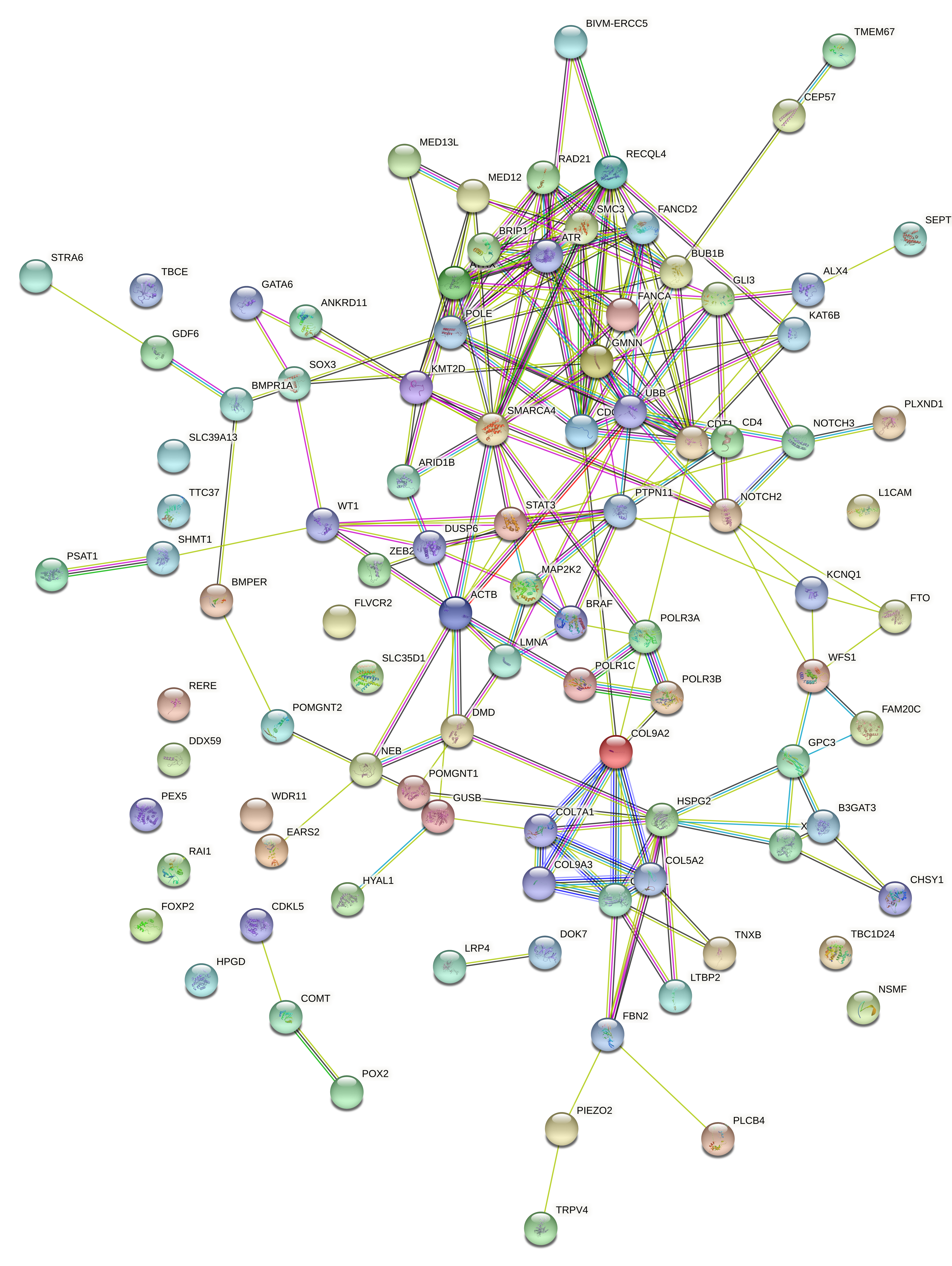

Supplement: Supplementary Figure 1 — Protein-protein interaction networks constructed with the previously syndromic cleft palate only (SCPO)-associated genes identified in the pools of patients with non-syndromic cleft palate only (NSCPO), but not in the control, using STRING. The networks include 205 edges (interactions) among 98 nodes based on curated databases (light blue), experimentally (purple), gene neighborhood (green), gene fusions (red), gene co-occurrence (blue), textmining (light green), co-expression (black) and protein homology (violet). The overall confidence score of this analysis was 0.446 (P < 1.0e-16), indicating a low change for false-positive interactions. [file Image_1.JPEG]

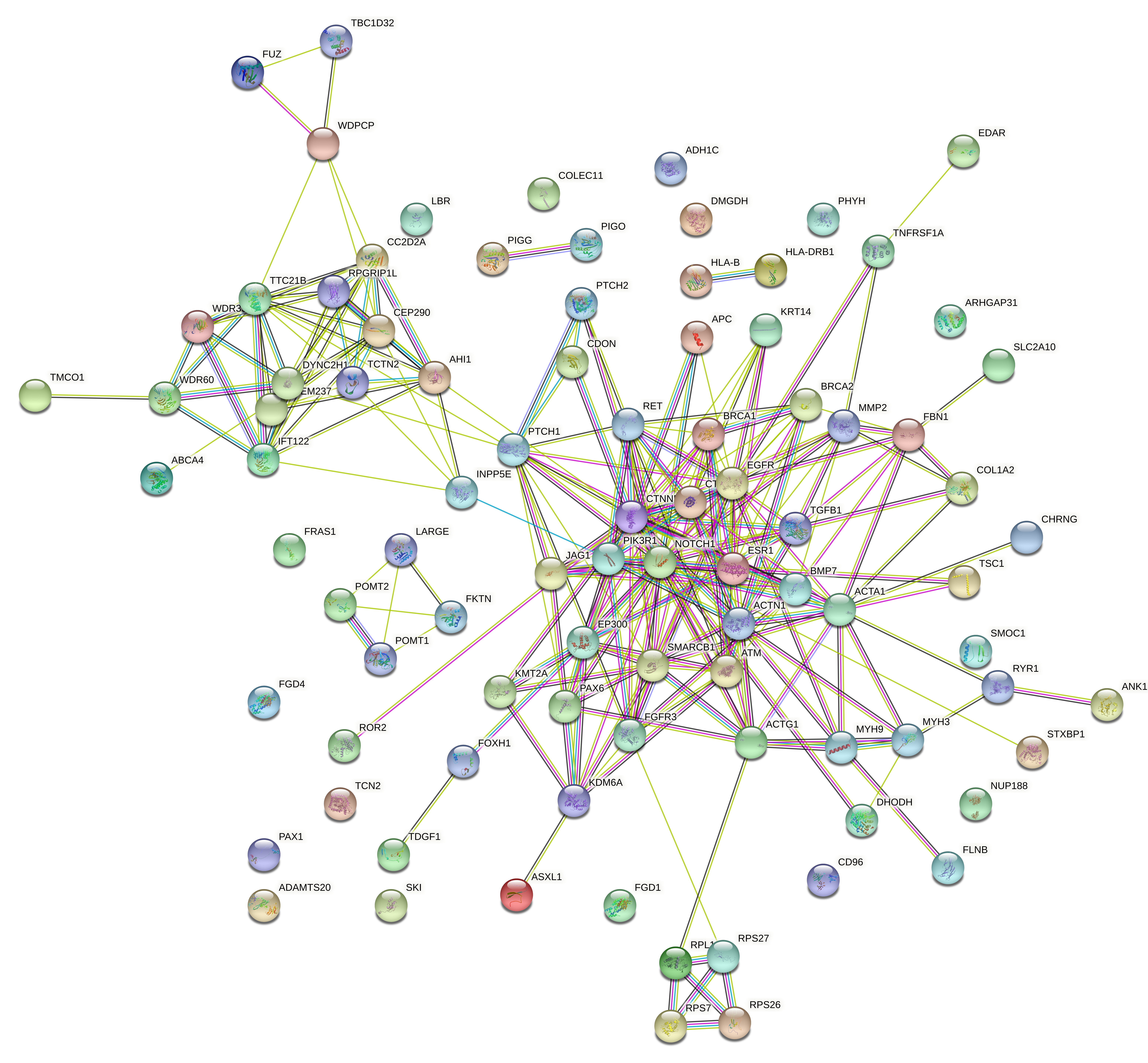

Supplement: Supplementary Figure 2 — The protein-protein interaction networks with cleft lip-palate (CLP)-associated targets found in the pools of patients with non-syndromic cleft palate only (NSCPO), but not in the control. Networks include 244 interactions among 91 nodes (overall coefficient score = 0.557; P < 1.0e-16). Different colors represent different levels of evidence of connection between proteins. Light blue represents curated databases, purple experimental evidence, green gene neighborhood, red gene fusions, blue gene co-occurrence, light green evidence from textmining, black co-expression, and violet protein homology. [file Image_2.JPEG]
