# Supplementary material for: Identification of Novel Variants in Cleft Palate-Associated Genes in Brazilian Patients With Non-syndromic Cleft Palate Only
Source: Front Cell Dev Biol. 2021 Jul 8;9:638522. doi: 10.3389/fcell.2021.638522 (PMC8297955; doi:10.3389/fcell.2021.638522)
Supplement: Supplementary file 3 [file Data_Sheet_1.DOCX]

Supplementary Table 1. Characteristics of the participants of this study, which were divided in 6 pools of 10 samples each.

|  |  |  | **Ancestry** | | |
| --- | --- | --- | --- | --- | --- |
|  | **Sample** | **Sex** | **European** | **African** | **Amerindian** |
| Pools from patients with nonsyndromic cleft palate only (NSCPO) | | | | | |
| Pool 1 | 1 | Female | 74.2% | 17.2% | 8.6% |
|  | 2 | Female | 61,1% | 26,3% | 12.6% |
|  | 3 | Female | 59.7% | 39.5% | 0.8% |
|  | 4 | Female | 81.2% | 13.6% | 5.2% |
|  | 5 | Female | 67,5% | 30,0% | 2,5% |
|  | 6 | Female | 77.0% | 21.9% | 1.1% |
|  | 7 | Female | 78.2% | 21.0% | 0.8% |
|  | 8 | Female | 57.8% | 40.4% | 1.8% |
|  | 9 | Female | 60.5% | 33.1% | 6.4% |
|  | 10 | Female | 67.4% | 31.1% | 1.5% |
| Average | | | 69.5% | 26.4% | 4.1% |
| Pool 2 | 11 | Female | 79.2% | 19.5% | 1.3% |
|  | 12 | Female | 68.8% | 30.7% | 0.5% |
|  | 13 | Female | 60.6% | 38.4% | 1.0% |
|  | 14 | Female | 84.3% | 7.8% | 7.9% |
|  | 15 | Female | 83.1% | 11.8% | 5.1% |
|  | 16 | Female | 75.7% | 23.4% | 0.9% |
|  | 17 | Female | 75.5% | 17.8% | 6.7% |
|  | 18 | Female | 67.8% | 28.2% | 4.0% |
|  | 19 | Female | 82.9% | 13.4% | 3.7% |
|  | 20 | Female | 75.8% | 20.8% | 3.4% |
| Average | | | 75.4% | 21.2% | 3.4% |
| Pool 3 | 21 | Male | 89.6% | 8.7% | 1.7% |
|  | 22 | Male | 68,9% | 16,0% | 15,1% |
|  | 23 | Male | 74.0% | 21.3% | 4.7% |
|  | 24 | Male | 76.9% | 22.4% | 0.7% |
|  | 25 | Male | 68,4% | 30,2% | 1,4% |
|  | 26 | Male | 79.5% | 17.3% | 3.2% |
|  | 27 | Male | 47.9% | 33.4% | 18.7% |
|  | 28 | Male | 62.3% | 36.3% | 1.4% |
|  | 29 | Male | 70.2% | 26.9% | 2.9% |
|  | 30 | Male | 79.7% | 17.1% | 3.2% |
| Average | | | 71.7% | 23.0% | 5.3% |
| Pools from healthy controls | | | | | |
| Pool 1 | 1 | Female | 89.5% | 9.0% | 1.5% |
|  | 2 | Female | 47.5% | 47.9% | 4.6% |
|  | 3 | Female | 74.1% | 22.5% | 3.4% |
|  | 4 | Female | 71.2% | 27.8% | 1.0% |
|  | 5 | Female | 21.3% | 56.5% | 22.2% |
|  | 6 | Female | 13.2% | 82.6% | 4.1% |
|  | 7 | Female | 32.7% | 24.0% | 43.3% |
|  | 8 | Female | 83.6% | 14.0% | 2.4% |
|  | 9 | Female | 85.1% | 13.8% | 1.1% |
|  | 10 | Female | 49.3% | 36.8% | 13.9% |
| Average | | | 56.8% | 33.5% | 9.7% |
| Pool 2 | 11 | Female | 95.3% | 3.8% | 0.9% |
|  | 12 | Female | 81.2% | 16.7% | 2.1% |
|  | 13 | Female | 89.8% | 9.1% | 1.1% |
|  | 14 | Female | 89.5% | 9.4% | 1.1% |
|  | 15 | Female | 91.9% | 7.2% | 0.9% |
|  | 16 | Female | 88.1% | 11.2% | 0.7% |
|  | 17 | Female | 77.4% | 22.3% | 0.3% |
|  | 18 | Female | 97.1% | 2.2% | 0.7% |
|  | 19 | Female | 92.5% | 6.9% | 0.6% |
|  | 20 | Female | 90.7% | 8.9% | 0.4% |
| Average | | | 89.4% | 9.8% | 8.8% |
| Pool 3 | 21 | Male | 97.5% | 1.9% | 0.5% |
|  | 22 | Male | 90.0% | 15.0% | 15.0% |
|  | 23 | Male | 90.0% | 15.0% | 15.0% |
|  | 24 | Male | 87.1% | 12.0% | 0.9% |
|  | 25 | Male | 78.1% | 6.8% | 15.1% |
|  | 26 | Male | 97.5% | 2.0% | 0.5% |
|  | 27 | Male | 91.6% | 7.9% | 0.5% |
|  | 28 | Male | 84.9% | 14.6% | 0.5% |
|  | 29 | Male | 90.5% | 9.2% | 0.3% |
|  | 30 | Male | 92.0% | 5.8% | 2.2% |
| Average | | | 89.9% | 9.0% | 5.1% |
