# Supplementary material for: Identification of Novel Variants in Cleft Palate-Associated Genes in Brazilian Patients With Non-syndromic Cleft Palate Only
Source: Front Cell Dev Biol. 2021 Jul 8;9:638522. doi: 10.3389/fcell.2021.638522 (PMC8297955; doi:10.3389/fcell.2021.638522)
Supplement: Supplementary file 4 [file Data_Sheet_2.DOCX]

| **Supplementary Table 2.** Variants in syndromic cleft palate only-associated genes found exclusively in the exome of patients with nonsyndromic cleft palate only (NSCPO). | | | | | | | | | | | | | | | | |  |
| --- | --- | --- | --- | --- | --- | --- | --- | --- | --- | --- | --- | --- | --- | --- | --- | --- | --- |
| **Gene** | **Protein** | **Cytogenetic Location** | **Variants** | **Chr:Pos** | **MAF 1K** | **MAF gnomAD Exomes** | **MAF gnomAD Genomes** | **MAF ExAC** | **MAF ABraOM** | **Sequence Ontology** | **SIFT** | **Polyphen2** | **Mutation Taster** | **Syndrome** | **OMIM Number** | **Reference** |  |
| *ACTB* | Actin Beta | 7p22.1 | rs11546939 | 7:5567677 | 0.0271566 | 0.0277751 | 0.0310643 | 0.029 | 0.035304 | synonymous_variant | - | - | - | Baraitser-Winter syndrome 1 Dystonia, juvenile-onset | *102630 | Verloes A, Donato N, Masliah-Planchon J, Jongmans M, Abdul-Raman O, Albrecht B, et al. Baraitser-Winter cerebrofrontofacial syndrome: delineation of the spectrum in 42 cases. Eur J Hum Genet. 2015;23(3):292-301. |  |
| *ALX4* | ALX Homeobox 4 | 11p11.2 | rs75147697 | 11:44289183 | 0.0800719 | 0.08027 | 0.0852199 | 0.083 | 0.073892 | intron_variant | - | - | - | Frontonasal dysplasia 2, Parietal foramina 2, Craniosynostosis 5 | *605420 | Beverdam A, Brouwer A, Reijnen M, Korving J, Meijlink F. Severe nasal clefting and abnormal embryonic apoptosis in Alx3/Alx4 double mutant mice. Development 2001;128:3975-3986 |  |
| *ANKRD11* | Ankyrin Repeat Domain 11 | 16q24.3 | rs144364610 | 16:89371668 | 0.00039936 | 0.000219459 | 0.00084196 | 0.0002636 | - | synonymous_variant | - | - | - | Kbg Syndrome | *611192 | Tunovic S, Barkovich J, Sherr EH, Slavotinek AM. De novo ANKRD11 and KDM1A gene mutations in a male with features of KBG syndrome and Kabuki syndrome. Am J Med Genet A. 2014;164A(7):1744-9. |  |
|  |  |  | rs61744778 | 16:89348606 | 0.013778 | 0.0277717 | 0.0228771 | 0.028 | 0.030378 | synonymous_variant | - | - | - |  |  |  |  |
|  |  |  | rs61741725 | 16:89349128 | 0.0297524 | 0.0403408 | 0.0416181 | 0.039 | 0.018062 | synonymous_variant | - | - | - |  |  |  |  |
|  |  |  | rs16965550 | 16:89379900 | 0.0613019 | 0.0255742 | 0.0419815 | 0.029 | 0.052326 | intron_variant | - | - | - |  |  |  |  |
| *ARID1B* | AT-Rich Interaction Domain 1B | 6q25.3 | rs112703040 | 6:157528197 | 0.00638978 | 0.00999553 | 0.00778223 | 0.01 | 0.010673 | synonymous_variant | - | - | - | Coffin-Siris syndrome 1 | *614556 |  |  |
| *ATR* | ATR Serine/Threonine Kinase | 3q23 | rs9870078 | 3:142272632 | 0.00738818 | 0.00152213 | 0.00717054 | 0.00187 | 0.004926 | intron_variant | - | - | - | Seckel syndrome 1 | *601215 |  |  |
|  |  |  | rs2229032 | 3:142178144 | 0.102236 | 0.130956 | 0.133161 | 0.132 | 0.142036 | missense_variant | Tolerated | Benign | Tolerated |  |  |  |  |
|  |  |  | rs58348002 | 3:142234404 | 0.048722 | 0.0104038 | 0.0426817 | 0.012 | 0.041872 | intron_variant | - | - | - |  |  |  |  |
| *ATRX* | ATRX Chromatin Remodeler | Xq21.1 | rs45608236 | X:76855073 | 0.0582781 | 0.0139014 | 0.053526 | 0.016 | 0.042914 | intron_variant | - | - | - | Mental retardation-hypotonic facies syndrome, X-linked, 1 | *300032 |  |  |
| *B3GAT3* | Beta-1,3-Glucuronyltransferase 3 | 11q12.3 | c.619-12G>C | 11:62384280 | - | - | - | - | - | - | - | - | - | Multiple joint dislocations, short stature, craniofacial dysmorphism, and congenital heart defects | *606374 |  |  |
| *BIVM-ERCC5/ERCC5* | BIVM-ERCC5 Readthrough | 13q33.1 | rs143419119 | 13:10351435 | 0.00179712 | 0.000742415 | 0.00258164 | 0.0008896 | - | intron_variant | - | - | - | Xeroderma pigmentosum, complementation group G | *133530 |  |  |
|  |  |  | rs181241783 | 13:10352042 | 0.00099840 | 0.00189046 | 0.00209894 | 0.001866 | 0.002463 | intron_variant | - | - | - |  |  |  |  |
|  |  |  | rs2227869 | 13:10351508 | 0.0423323 | 0.0426357 | 0.049535 | 0.044 | 0.032020 | missense_variant | Tolerated | Benign | Tolerated |  |  |  |  |
| *BMPER* | BMP Binding Endothelial Regulator | 7p14.3 | rs112068976 | 7:33946464 | 0.00079872 | 0.000231585 | 0.00103312 | 0.000313 | - | synonymous_variant | - | - | - | Diaphanospondylodysostosis | *608699 |  |  |
| *BMPR1A* | Bone Morphogenetic Protein Receptor Type 1A | 10q23.2 | rs12267107 | 10:88672153 | 0.0411342 | 0.0101563 | 0.0380512 | 0.013 | 0.040230 | intron_variant | - | - | - | Juvenile Polyposis syndrome | *601299 |  |  |
| *BRAF* | B-Raf Proto-Oncogene, Serine/Threonine Kinase | 7q34 | rs371877084 | 7:140624426 | 0.0091853 | 0.00239124 | 0.0090715 | 0.0006336 | 0.005922 | missense_variant | Tolerated | Benign | Tolerated | Cardiofaciocutaneous syndrome 1 | *164757 |  |  |
|  |  |  | rs3789806 | 7:140449071 | 0.348043 | 0.198481 | 0.293642 | 0.209 | 0.251269 | intron_variant | - | - | - |  |  |  |  |
| *BRIP1* | BRCA1 Interacting Protein C-Terminal Helicase 1 | 17q23.2 | rs28997569 | 17:59885956 | 0.00139776 | 0.00083808 | 0.00074323 | 0.0007083 | 0.000821 | missense_variant | Damaging | Probably damaging | Tolerated | Fanconi anemia, complementation group J | *605882 |  |  |
| *BUB1B* | BUB1 Mitotic Checkpoint Serine/Threonine Kinase B | 15q15.1 | rs16970425 | 15:40462228 | 0.0509185 | 0.0121942 | 0.0473038 | 0.015 | 0.036946 | intron_variant | - | - | - | Mosaic variegated aneuploidy syndrome 1 | *602860 |  |  |
|  |  |  | rs1801528 | 15:40498503 | 0.0928514 | 0.0198377 | 0.0794661 | 0.025 | 0.051724 | missense_variant | Tolerated | Benign | Tolerated |  |  |  |  |
|  |  |  | rs35611758 | 15:40512906 | 0.0958466 | 0.0206284 | 0.0826444 | 0.026 | 0.053366 | synonymous_variant | - | - | - |  |  |  |  |
| *CD4* | CD4 Molecule | 12p13.31 | rs11575099 | 12:6924109 | 0.0129792 | 0.018833 | 0.0198834 | 0.02 | 0.018062 | synonymous_variant | - | - | - | C syndrome | *186940 |  |  |
| *CDC6* | Cell Division Cycle 6 | 17q21.2 | rs1130199 | 17:38447569 | 0.111222 | 0.0244543 | 0.0966482 | 0.031 | 0.076355 | synonymous_variant | - | - | - | Meier-Gorlin syndrome 5 | *602627 |  |  |
|  |  |  | rs13706 | 17:38457151 | 0.279952 | 0.153137 | 0.218229 | 0.159 | 0.195402 | missense_variant | Damaging | Benign | Damaging |  |  |  |  |
| *CDKL5* | Cyclin Dependent Kinase Like 5 | Xp22.13 | rs58669795 | X:18671530 | 0.00741722 | 0.00157342 | 0.00474066 | 0.001673 | 0.000998 | intron_variant | - | - | - | Early Infantile Epileptic Encephalopathy | *300203 |  |  |
| *CDT1* | Chromatin Licensing and DNA Replication Factor 1 | 16q24.3 | rs139633564 | 16:88874566 | 0.0071885 | 0.00198352 | 0.0071447 | 0.00223 | 0.005747 | synonymous_variant | - | - | - | Meier-Gorlin Syndrome 1 | *605525 |  |  |
|  |  |  | rs3218730 | 16:88873790 | 0.0539137 | 0.0554827 | 0.0504169 | 0.054 | 0.079639 | synonymous_variant | - | - | - |  |  |  |  |
| *CEP57* | Centrosomal Protein 57 | 11q21 | rs11742 | 11:95555088 | 0.0151757 | 0.0320285 | 0.0307384 | 0.032 | 0.041051 | synonymous_variant | - | - | - | Mosaic Variegated Aneuploidy Syndrome 1 | *607951 |  |  |
| *CHSY1* | Chondroitin Sulfate Synthase 1 | 15q26.3 | rs62621400 | 15:10171823 | 0.0477236 | 0.0630137 | 0.0431652 | 0.064 | 0.039409 | missense_variant | Tolerated | Benign | Damaging | Temtamy preaxial brachydactyly syndrome | *608183 |  |  |
| *COL3A1* | Collagen Type III Alpha 1 Chain | 2q32.2 | rs2271679 | 2:189859073 | 0.16254 | 0.0555176 | 0.128969 | 0.064 | 0.119048 | intron_variant | - | - | - | Ectodermal dysplasia-like syndrome with mental retardation | *120180 | Rifai L, Port-Lis M, Tabet AC, Bailleul-Forestier I, Benzacken B, Drunat S, et al. Ectodermal dysplasia-like syndrome with mental retardation due to contiguous gene deletion: further clinical and molecular delineation of del(2q32) syndrome. Am J Med Genet A. 2010;152A(1):111-7. |  |
|  |  |  | rs41265549 | 2:189875125 | 0.0121805 | 0.0296068 | 0.0252989 | 0.03 | 0.018062 | intron_variant | - | - | - |  |  |  |  |
| *COL5A2* | Collagen Type V Alpha 2 Chain | 2q32.2 | rs35830636 | 2:189932764 | 0.0115815 | 0.0319492 | 0.0363924 | 0.032 | 0.024631 | missense_variant | Tolerated | Benign | Damaging | Ectodermal dysplasia-like syndrome | *120190 |  |  |
| *COL7A1* | Collagen Type VII Alpha 1 Chain | 3p21.31 | rs116512620 | 3:48623940 | 0.00299521 | 0.000641744 | 0.00381309 | 0.0008895 | 0.000821 | intron_variant | - | - | - | Beckwith-Wiedemann syndrome | *120120 |  |  |
|  |  |  | rs116005007 | 3:48627789 | 0.00219649 | 0.00423994 | 0.00330375 | 0.004192 | 0.012315 | missense_variant | Damaging | Possibly damaging | Damaging |  |  |  |  |
|  |  |  | rs17256786 | 3:48604016 | 0.0257588 | 0.0541009 | 0.0526963 | 0.054 | 0.068966 | intron_variant | - | - | - |  |  |  |  |
|  |  |  | rs41290686 | 3:48604059 | 0.0257588 | 0.0536944 | 0.0522074 | 0.053 | 0.068966 | intron_variant | - | - | - |  |  |  |  |
|  |  |  | rs9814951 | 3:48610925 | 0.0908546 | 0.0578594 | 0.0711279 | 0.06 | 0.060755 | intron_variant | - | - | - |  |  |  |  |
| *COL9A2* | Collagen Type IX Alpha 2 Chain | 1p34.2 | rs12038020 | 1:40768276 | 0.0664936 | 0.029844 | 0.0416532 | 0.026 | 0.030142 | intron_variant | - | - | - | Stickler syndrome, type V | *120260 |  |  |
|  |  |  | rs2228567 | 1:40773123 | 0.0846645 | 0.104312 | 0.0932549 | 0.104 | 0.088670 | missense_variant | Damaging | Possibly damaging | Tolerated |  |  |  |  |
| *COL9A3* | Collagen Type IX Alpha 3 Chain | 20q13.33 | rs117342457 | 20:61461847 | 0.00259585 | 0.00371981 | 0.00247364 | 0.003783 | 0.009852 | intron_variant | - | - | - | Autosomal recessive Stickler syndrome | *120270 |  |  |
|  |  |  | rs61734651 | 20:61451332 | 0.0219649 | 0.0479211 | 0.0459584 | 0.047 | 0.035304 | missense_variant | Damaging | Possibly damaging | Damaging |  |  |  |  |
| *COMT* | Catechol-O-Methyltransferase | 22q11.21 | rs769224 | 22:19951804 | 0.0503195 | 0.0297322 | 0.0468124 | 0.031 | 0.028736 | synonymous_variant | - | - | - | Digeorge syndrome | +116790 | Lachman HM, Morrow B, Shprintzen R, Veit S, Parsia SS, Faedda G, et al. Association of codon 108/158 catechol-O-methyltransferase gene polymorphism with the psychiatric manifestations of velo-cardio-facial syndrome. Am J Med Genet 1996;67:468-472. |  |
| *DDX59* | DEAD-Box Helicase 59 | 1q32.1 | rs143466956 | 1:200618332 | 0.00359425 | 0.000909117 | 0.00365011 | 0.001219 | 0.001642 | missense_variant | Damaging | Probably damaging | Damaging | Orofaciodigital syndrome V | *615464 |  |  |
| *DMD* | Dystrophin | Xp21.2-p21.1 | rs149882431 | X:32536218 | 0.000264901 | 0.000196634 | 0.000461723 | 0.0002142 | 0.000998 | synonymous_variant | - | - | - | Beckwith-Wiedemann Syndrome | *300377 |  |  |
|  |  |  | rs16998350 | X:32563301 | 0.00741722 | 0.00165193 | 0.00623701 | 0.001829 | 0.005988 | missense_variant | Tolerated | Benign | Tolerated |  |  |  |  |
|  |  |  | rs34155804 | X:32662355 | 0.00847682 | 0.00297009 | 0.00846873 | 0.003377 | 0.009980 | missense_variant | Tolerated | Benign | Tolerated |  |  |  |  |
|  |  |  | rs1800278 | X:31496426 | 0.0370861 | 0.0208694 | 0.0298895 | 0.021 | 0.035928 | missense_variant | Damaging | Benign | Tolerated |  |  |  |  |
|  |  |  | rs41305353 | X:31496431 | 0.0357616 | 0.0205521 | 0.0287586 | 0.021 | 0.034930 | missense_variant | Damaging | Possibly damaging | Damaging |  |  |  |  |
|  |  |  | rs796898196 | X:32472764 | - | - | - | - | - | - | - | - | - |  |  |  |  |
| *DOK7* | Docking Protein 7 | 4p16.3 | rs185980724 | 4:3491384 | 0.00139776 | 0.000518109 | 0.000713452 | 0.0003578 | 0.007414 | intron_variant | - | - | - | Fetal akinesia deformation sequence | *610285 |  |  |
| *DUSP6* | Dual Specificity Phosphatase 6 | 12q21.33 | rs61734372 | 12:89745557 | 0.0111821 | 0.023714 | 0.0248238 | 0.023 | 0.024631 | missense_variant | Tolerated | Benign | Damaging | Hypogonadotropic hypogonadism 19 with or without anosmia | *602748 |  |  |
| *EARS2* | Glutamyl-TRNA Synthetase 2, Mitochondrial | 16p12.2 | rs75133940 | 16:23536531 | 0.029353 | 0.0678083 | 0.0782381 | 0.069 | 0.043514 | intron_variant | Damaging | Benign | Tolerated | Combined oxidative phosphorylation deficiency 12 | *612799 |  |  |
| *FAM20C* | FAM20C Golgi Associated Secretory Pathway Kinase | 7p22.3 | rs148276213 | 7:295970 | 0.00339457 | 0.00492175 | 0.0084088 | 0.003385 | 0.002636 | missense_variant | Tolerated | Probably damaging | Damaging | Raine syndrome | *611061 |  |  |
|  |  |  | rs77670366 | 7:228768 | 0.0301518 | 0.0566583 | 0.0609709 | 0.04 | 0.061913 | intron_variant | - | - | - |  |  |  |  |
| *FANCA* | FA Complementation Group A | 16q24.3 | rs9282681 | 16:89805914 | 0.0722843 | 0.0653197 | 0.0673605 | 0.031 | 0.033997 | missense_variant | Tolerated | Benign | Tolerated | Fanconi Anemia, Complementation Group a | *607139 |  |  |
|  |  |  | rs11649210 | 16:89807233 | 0.137979 | 0.0924409 | 0.111564 | 0.095 | 0.072250 | synonymous_variant | - | - | - |  |  |  |  |
| *FANCD2* | FA Complementation Group D2 | 3p25.3 | rs34177396 | 3:10103812 | 0.124201 | 0.075831 | 0.135454 | 0.076 | 0.114122 | intron_variant | - | - | - | Fanconi Anemia, Complementation Group a | *613984 |  |  |
| *FBN2* | Fibrillin 2 | 5q23.3 | rs28763922 | 5:127609548 | 0.00459265 | 0.00104969 | 0.00294004 | 0.00126 | 0.002463 | synonymous_variant | - | - | - | Pierre Robin sequence | *612570 | Ansari M, Rainger JK, Murray JE, Hanson I, Firth HV, Mehendale F, et al. A syndromic form of Pierre Robin sequence is caused by 5q23 deletions encompassing FBN2 and PHAX. Eur J Med Genet. 2014;57(10):587-95. |  |
|  |  |  | rs6862010 | 5:127616045 | 0.00658946 | 0.00131825 | 0.00429531 | 0.001582 | 0.003284 | intron_variant | - | - | - |  |  |  |  |
|  |  |  | rs28763931 | 5:127624841 | 0.00579073 | 0.00113484 | 0.00380842 | 0.0014 | 0.002463 | synonymous_variant | - | - | - |  |  |  |  |
|  |  |  | rs148293104 | 5:127637120 | 0.00579073 | 0.00119503 | 0.00384516 | 0.001458 | 0.002463 | synonymous_variant | - | - | - |  |  |  |  |
|  |  |  | rs186714952 | 5:127673666 | 0.00139776 | 0.00221052 | 0.00238941 | 0.002216 | 0.001642 | intron_variant | - | - | - |  |  |  |  |
|  |  |  | rs182515008 | 5:127800658 | 0.000599042 | 0.000101901 | 0.00029051 | 0.0001977 | 0.000821 | intron_variant | - | - | - |  |  |  |  |
|  |  |  | rs28763927 | 5:127616014 | 0.0804712 | 0.0749994 | 0.0640785 | 0.076 | 0.059934 | splice_region_variant | - | - | - |  |  |  |  |
|  |  |  | rs17676694 | 5:127680042 | 0.0399361 | 0.0774245 | 0.0791462 | 0.081 | 0.060755 | intron_variant | - | - | - |  |  |  |  |
| *FLVCR2* | FLVCR Heme Transporter 2 | 14q24.3 | rs12436885 | 14:76101386 | 0.118211 | 0.115287 | 0.0799251 | 0.11 | 0.099343 | intron_variant | - | - | - | Proliferative vasculopathy and hydranencephaly-hydrocephal y syndrome | *610865 |  |  |
| *FOXP2* | Forkhead Box P2 | 7q31.1 | rs116320717 | 7:114055327 | 0.00938498 | 0.00153534 | 0.00748105 | 0.0006168 | - | 5_prime_UTR_variant | - | - | - | Childhood Apraxia of Speech | *605317 | Shah NSM, Salahshourifar I, Sulong S, Sulaiman WAW, Halim AS. Discovery of candidate genes for nonsyndromic cleft lip palate through genome-wide linkage analysis of large extended families in the Malay population. BMC Genet. 2016; 17:39. |  |
| *FTO* | FTO Alpha-Ketoglutarate Dependent Dioxygenase | 16q12.2 | rs373705985 | 16:53913912 | - | - | - | - | 0.076132 | - | - | - | - | Growth retardation, developmental delay, coarse facies, and early death | *610966 |  |  |
| *GATA6* | GATA Binding Protein 6 | 18q11.2 | rs3764962 | 18:19763011 | 0.224042 | 0.067507 | 0.19142 | 0.079 | 0.165025 | splice_region_variant | - | - | - | Persistent truncus arteriosus | *601656 |  |  |
| *GDF6* | Growth Differentiation Factor 6 | 8q22.1 | rs77859767 | 8:97157791 | 0.0638978 | 0.015123 | 0.0598202 | 0.016 | 0.053542 | intron_variant | - | - | - | Klippel-feil syndrome 1, autosomal dominant | *601147 |  |  |
|  |  |  | rs11783820 | 8:97157792 | 0.0377396 | 0.0433397 | 0.0331695 | 0.043 | 0.057471 | intron_variant | - | - | - |  |  |  |  |
| *GLI3* | GLI Family Zinc Finger 3 | 7p14.1 | rs34089404 | 7:42004600 | 0.0553115 | 0.0682929 | 0.0641125 | 0.068 | 0.069787 | synonymous_variant | - | - | - | Hypothalamic hamartomas Pallister-Hall syndrome | *165240 | Huang X, Goudy SL, Ketova T, Litingtung Y, Chiang C. Gli3-deficient mice exhibit cleft palate associated with abnormal tongue development. Dev Dyn 2008;237:3079-3087. |  |
|  |  |  | rs35280470 | 7:42004664 | 0.0151757 | 0.0353562 | 0.0325574 | 0.035 | 0.036946 | missense_variant | Tolerated | Benign | Damaging |  |  |  |  |
|  |  |  | rs34245321 | 7:42005845 | 0.0195687 | 0.0373087 | 0.0328219 | 0.036 | 0.037891 | synonymous_variant | - | - | - |  |  |  |  |
| *GMNN* | Geminin DNA Replication Inhibitor | 6p22.3 | rs2307307 | 6:24781719 | 0.0864617 | 0.0269529 | 0.0690268 | 0.034 | 0.050082 | missense_variant | Tolerated | Possibly damaging | Tolerated | Meier-Gorlin Syndrome 1; Meier-Gorlin Syndrome 6 | *602842 |  |  |
|  |  |  | rs2307306 | 6:24781735 | 0.0866613 | 0.0270478 | 0.0690726 | 0.034 | 0.050082 | missense_variant | Tolerated | Benign | Tolerated |  |  |  |  |
| *GPC3* | Glypican 3 | Xq26.2 | rs61754631 | X:132670269 | 0.0537748 | 0.0155755 | 0.050832 | 0.018 | 0.042914 | synonymous_variant | - | - | - | Simpson-Golabi-Behmel syndrome, type 1 | *300037 |  |  |
|  |  |  | c.1662T>C | X:132670302 | - | - | - | - | - | - | - | - | - |  |  |  |  |
|  |  |  | rs2314298 | X:132730541 | 0.0654305 | 0.0184154 | 0.0596088 | 0.021 | 0.052894 | synonymous_variant | - | - | - |  |  |  |  |
| *GUSB* | Glucuronidase Beta | 7q11.21 | rs141303888 | 7:65439930 | 0.00279553 | 0.000512274 | 0.00210043 | 0.0006177 | - | synonymous_variant | - | - | - | Mucopolysaccharidosis type VII | *611499 | Allanson JE, Gemmill RM, Hecht BK, Johnsen S, Wenger DA. Deletion mapping of the beta-glucuronidase gene. Am J Med Genet 1988;29:517-522. |  |
| *HPGD* | 15-Hydroxyprostaglandin Dehydrogenase | 4q34.1 | rs17060532 | 4:175413296 | 0.0119808 | 0.0268734 | 0.0250356 | 0.029 | 0.036946 | intron_variant | - | - | - | Hypertrophic osteoarthropathy, primary, autosomal recessive, 1 | *601688 |  |  |
| *HSPG2* | Heparan Sulfate Proteoglycan 2 | 1p36.12 | rs111866498 | 1:22157795 | - | - | - | - | 0.006568 | - | - | - | - | Schwartz-Jampel syndrome, type 1; dyssegmental dysplasia, Silverman-Handmaker type 1 | *142461 |  |  |
|  |  |  | rs112494360 | 1:22157545 | 0.0289537 | 0.0414578 | 0.0309626 | 0.037 | 0.045977 | synonymous_variant | - | - | - |  |  |  |  |
|  |  |  | rs2229487 | 1:22159008 | 0.157748 | 0.171332 | 0.140876 | 0.173 | 0.112479 | synonymous_variant | - | - | - |  |  |  |  |
| *HYAL1* | Hyaluronidase 1 | 3p21.31 | rs116482870 | 3:50339622 | 0.0275559 | 0.0519704 | 0.042111 | 0.051 | 0.037767 | missense_variant | Tolerated | Benign | Tolerated | Mucopolysaccharidosis, type IX | *607071 |  |  |
| *KAT6B* | Lysine Acetyltransferase 6B | 10q22.2 | rs3740322 | 10:76788860 | 0.0615016 | 0.0347489 | 0.0331265 | 0.033 | 0.030378 | synonymous_variant | - | - | - | Genitopatellar syndrome | *605880 |  |  |
|  |  |  | rs3740321 | 10:76789077 | 0.0613019 | 0.0346831 | 0.0330036 | 0.033 | 0.029557 | missense_variant | Tolerated | Benign | Tolerated |  |  |  |  |
| *KCNQ1* | Potassium Voltage-Gated Channel Subfamily Q Member 1 | 11p15.5-p15.4 | rs11024034 | 11:2790163 | 0.0461262 | 0.0866099 | 0.0750485 | 0.086 | 0.073071 | intron_variant | - | - | - | Beckwith-Wiedemann Syndrome | *607542 |  |  |
| *KIFBP/KIAA1279* | Kinesin Family Binding Protein | 10q22.1 | rs114205227 | 10:70775695 | 0.0061901 | 0.00111701 | 0.00384243 | 0.001343 | 0.001642 | synonymous_variant | - | - | - | Goldberg-Shprintzen Syndrome | *609367 |  |  |
| *KMT2D* | Lysine Methyltransferase 2D | 12q13.12 | rs80132640 | 12:49426460 | 0.0171725 | 0.0263664 | 0.0308538 | 0.026 | 0.020525 | missense_variant | Damaging | Benign | Tolerated | Kabuki syndrome 1 | *602113 |  |  |
|  |  |  | rs55865069 | 12:49448463 | 0.0163738 | 0.0260112 | 0.0307847 | 0.026 | 0.021346 | missense_variant | Tolerated | Benign | Tolerated |  |  |  |  |
| *L1CAM* | L1 Cell Adhesion Molecule | Xq28 | rs144605615 | X:153141264 | 0.00821192 | 0.00247477 | 0.00848816 | 0.002818 | 0.008982 | missense_variant | Damaging | Benign | Tolerated | Hydrocephalus due to congenital stenosis of aqueduct of sylvius | *308840 |  |  |
| *LMNA* | Lamin A/C | 1q22 | rs11264444 | 1:156104779 | 0.0565096 | 0.01318 | 0.0534329 | 0.017 | 0.038588 | intron_variant | - | - | - | Restrictive dermopathy, lethal | *150330 |  |  |
| *LRP4* | LDL Receptor Related Protein 4 | 11p11.2 | rs77382495 | 11:46897546 | 0.00419329 | 0.00054523 | 0.00213178 | 0.0006919 | 0.001642 | intron_variant | - | - | - | Cenani-Lenz syndactyly syndrome | *604270 |  |  |
|  |  |  | rs61745166 | 11:46921829 | 0.00738818 | 0.00132788 | 0.00465236 | 0.001804 | 0.003284 | synonymous_variant | - | - | - |  |  |  |  |
|  |  |  | rs2306033 | 11:46897446 | 0.219249 | 0.20826 | 0.151141 | 0.194 | 0.123153 | missense_variant | Tolerated | Benign | Tolerated |  |  |  |  |
| *LTBP2* | Latent Transforming Growth Factor Beta Binding Protein 2 | 7q11.21 | rs151176143 | 14:74983580 | 0.000399361 | 0.0012345 | 0.0010672 | 0.0004263 | 0.000839 | synonymous_variant | - | - | - | Marfan syndrome | *607444 |  |  |
|  |  |  | rs45473602 | 14:74976852 | 0.0241613 | 0.0186032 | 0.0222718 | 0.019 | 0.041051 | synonymous_variant | - | - | - |  |  |  |  |
|  |  |  | rs61738025 | 14:75019002 | 0.0704872 | 0.0594617 | 0.0635634 | 0.059 | 0.064039 | synonymous_variant | - | - | - |  |  |  |  |
| *MAP2K2* | Mitogen-Activated Protein Kinase Kinase 2 | 19p13.3 | rs17851657 | 19:4102449 | 0.111222 | 0.163917 | 0.134131 | 0.155 | 0.151067 | synonymous_variant | - | - | - | Cardiofaciocutaneous Syndrome 1 | *601263 |  |  |
| *MED12* | Mediator Complex Subunit 12 | Xq13.1 | rs201566660 | X:70339715 | 0.00238411 | 0.000557078 | 0.00181717 | 0.0006783 | - | synonymous_variant | - | - | - | Lujan-Fryns syndrome, Opitz-KAVEGGIA syndrome | *300188 |  |  |
|  |  |  | rs5030617 | X:70348317 | 0.183046 | 0.137711 | 0.148852 | 0.141 | 0.151697 | intron_variant | - | - | - |  |  |  |  |
| *MED13L* | Mediator Complex Subunit 13L | 12q24.21 | rs371607017 | 12:116453111 | 0.00159744 | 0.000668253 | 0.00255003 | 0.0008237 | - | intron_variant | - | - | - | Isolated Pierre Robin Sequence, Pierre Robin Syndrome | *608771 |  |  |
| *NEB* | Nebulin | 2q23.3 | rs34471889 | 2:152420160 | - | - | - | - | 0.000821 | - | - | - | - | Nemaline myopathy 2 | *161650 |  |  |
|  |  |  | rs201250579 | 2:152474777 | - | - | - | - | 0.003284 | - | - | - | - |  |  |  |  |
|  |  |  | rs200545007 | 2:152522801 | - | - | - | - | - | - | - | - | - |  |  |  |  |
|  |  |  | rs13031275 | 2:152346494 | 0.0167732 | 0.0419196 | 0.0370502 | 0.044 | 0.036125 | synonymous_variant | - | - | - |  |  |  |  |
|  |  |  | rs11436831 | 2:152447846 | 0.155351 | 0.233112 | 0.202941 | 0.098 | 0.239966 | intron_variant | - | - | - |  |  |  |  |
|  |  |  | rs10909569 | 2:152448640 | - | 0.123267 | 0.121645 | 0.15 | 0.129834 | missense_variant | Tolerated | - | Damaging |  |  |  |  |
|  |  |  | rs61730771 | 2:152497088 | 0.0323482 | 0.0211793 | 0.0226303 | 0.022 | 0.015599 | synonymous_variant | - | - | - |  |  |  |  |
|  |  |  | rs34800215 | 2:152527636 | 0.0271566 | 0.0197783 | 0.0258058 | 0.02 | 0.020525 | missense_variant | Tolerated | Probably damaging | Damaging |  |  |  |  |
| *NOTCH2* | Notch Receptor 2 | 1p12 | rs36084504 | 1:120483244 | - | - | - | - | 0.008210 | - | - | - | - | Hajdu-Cheney syndrome | *600275 |  |  |
|  |  |  | rs41306195 | 1:120463044 | 0.0179712 | 0.0478681 | 0.0571207 | 0.052 | 0.030378 | intron_variant | - | - | - |  |  |  |  |
|  |  |  | rs782658834 | 1:120612041 | - | - | - | 0.044 | 0.263158 | 5_prime_UTR_variant | - | - | - |  |  |  |  |
| *NOTCH3* | Notch Receptor 3 | 19p13.12 | rs16980398 | 19:15276739 | 0.121805 | 0.0343492 | 0.114937 | 0.042 | 0.089491 | synonymous_variant | - | - | - | Lateral Meningocele Syndrome | *600276 |  |  |
|  |  |  | rs10408676 | 19:15290007 | 0.0844649 | 0.0248198 | 0.074552 | 0.03 | 0.058292 | missense_variant | Damaging | Possibly damaging | Damaging |  |  |  |  |
|  |  |  | rs61749020 | 19:15300136 | 0.0151757 | 0.0263579 | 0.0252261 | 0.026 | 0.027094 | synonymous_variant | - | - | - |  |  |  |  |
| *NSMF* | NMDA Receptor Synaptonuclear Signaling And Neuronal Migration Factor | 9q34.3 | rs41297241 | 9:140344010 | 0.118411 | 0.0538491 | 0.0932708 | 0.058 | 0.077997 | intron_variant | - | - | - | Hypogonadotropic hypogonadism 9 with or without anosmia, Kallmann syndrome | *608137 |  |  |
| *PEX5* | Peroxisomal Biogenesis Factor 5 | 12p13.31 | rs73051959 | 12:7361287 | 0.0123802 | 0.0211876 | 0.0186729 | 0.021 | 0.008237 | intron_variant | - | - | - | Peroxisome biogenesis disorder 2a | *600414 |  |  |
| *PIEZO2* | Piezo Type Mechanosensitive Ion Channel Component 2 | 18p11.22-p1 1.21 | rs200276831 | 18:10699142 | 0.000199681 | 0.000626978 | 0.000936269 | 0.0001386 | - | missense_variant | Tolerated | - | Damaging | Arthrogryposis, distal, type 3; Arthrogryposis, distal, type 5; Marden-Walker syndrome | *613629 |  |  |
| *PLCB4* | Phospholipase C Beta 4 | 20p12.3-p12 .2 | rs73248729 | 20:9389683 | 0.048722 | 0.0102626 | 0.0382429 | 0.013 | 0.032020 | intron_variant | - | - | - | Auriculocondylar syndrome 2 | *600810 |  |  |
| *PLXND1* | Plexin D1 | 3q22.1 | rs2285365 | 3:129289752 | 0.146765 | 0.0694273 | 0.120578 | 0.074 | 0.114943 | synonymous_variant | - | - | - | Moebius Syndrome, Conotruncal Heart Malformations | *604282 |  |  |
| *POLE* | DNA Polymerase Epsilon, Catalytic Subunit | 12q24.33 | rs5744758 | 12:133253195 | 0.00359425 | 0.000804152 | 0.00271476 | 0.001038 | 0.002463 | synonymous_variant | - | - | - | Intrauterine Growth Retardation, Metaphyseal Dysplasia, Adrenal Hypoplasia Congenita, and Genital Anomalies | *174762 |  |  |
| *POLR1C* | RNA Polymerase I And III Subunit C | 6p21.1 | rs113209557 | 6:43488836 | 0.0131789 | 0.0180581 | 0.016323 | 0.019 | 0.013957 | intron_variant | - | - | - | Treacher Collins syndrome 3 | *610060 |  |  |
|  |  |  | rs113321982 | 6:43488894 | 0.0173722 | 0.0210157 | 0.0173371 | 0.022 | 0.012336 | intron_variant | - | - | - |  |  |  |  |
| *POLR3A* | RNA Polymerase III Subunit A | 10q22.3 | rs146605905 | 10:79784277 | 0.00439297 | 0.00341928 | 0.00680052 | 0.003524 | 0.011513 | intron_variant | - | - | - | Wiedemann-Rautenstrauch syndrome | *614258 |  |  |
|  |  |  | rs3815891 | 10:79745789 | 0.202875 | 0.0824419 | 0.115091 | 0.086 | 0.101806 | intron_variant | - | - | - |  |  |  |  |
| *POLR3B* | RNA Polymerase III Subunit B | 12q23.3 | c.2005G>A | 12:106838290 | - | - | - | - | - | - | - | - | - | Hypogonadotropic Hypogonadism 7 with or Without Anosmia | *614366 |  |  |
| *POMGNT1* | Protein O-Linked Mannose N-Acetylglucosaminyltransferase 1 (Beta 1,2-) | 1p34.1 | rs115804669 | 1:46659636 | 0.0319489 | 0.0260688 | 0.0296857 | 0.026 | 0.020525 | intron_variant | - | - | - | Walker-Warburg Syndrome | *606822 |  |  |
|  |  |  | rs41309197 | 1:46662609 | 0.0441294 | 0.0292085 | 0.0444811 | 0.03 | 0.032020 | intron_variant | - | - | - |  |  |  |  |
| *POMGNT2* | Protein O-Linked Mannose N-Acetylglucosaminyltransferase 2 (Beta 1,4-) | 3p22.1 | rs115870061 | 3:43122162 | 0.00499201 | 0.00106109 | 0.00432956 | 0.001491 | 0.00243 | synonymous_variant | - | - | - | Walker-Warburg Syndrome | *614828 | Huang L, Jia Z, Shi Y, Du Q, Shi J, Wang Z, et al. Genetic factors define CPO and CLO subtypes of nonsyndromicorofacial cleft. PLoS Genet. 2019; 15(10), e1008357. |  |
| *PRODH* | Proline Dehydrogenase 1 | 22q11.21 | rs16983347 | 22:18900868 | 0.0181709 | 0.0299632 | 0.0257582 | 0.022 | 0.024167 | synonymous_variant | - | - | - | velo-cardio-facial syndrome | *606810 |  |  |
|  |  |  | rs2870983 | 22:18905842 | 0.0397364 | 0.0493524 | 0.0461904 | 0.05 | 0.064860 | missense_variant | Tolerated | Benign | Tolerated |  |  |  |  |
|  |  |  | rs11913840 | 22:18912677 | 0.0563099 | 0.0389765 | 0.0529058 | 0.04 | 0.061576 | stop_gained | - | - | Damaging |  |  |  |  |
| *PSAT1* | Phosphoserine Aminotransferase 1 | 9q21.2 | rs115263053 | 9:80921343 | 0.000199681 | 0.0000730923 | 0.000129124 | 0.00004118 | 0.000821 | missense_variant | Tolerated | Possibly damaging | Damaging | Neu-Laxova syndrome 2 | *610936 |  |  |
| *PTPN11* | Protein Tyrosine Phosphatase Non-Receptor Type 11 | 12q24.13 | rs41304351 | 12:112891203 | 0.00599042 | 0.00994303 | 0.00898281 | 0.009744 | 0.023810 | intron_variant | - | - | - | LEOPARD syndrome 1 | *176876 |  |  |
| *RAD21* | RAD21 Cohesin Complex Component | 8q24.11 | rs75160167 | 8:117864867 | 0.0071885 | 0.0014362 | 0.00504854 | 0.001952 | 0.004153 | missense_variant | Tolerated | Benign | Damaging | Cornelia de Lange syndrome 4 | *606462 |  |  |
| *RAI1* | Retinoic Acid Induced 1 | 17p11.2 | [rs770334543](http://www.ncbi.nlm.nih.gov/projects/SNP/snp_ref.cgi?rs=rs770334543) | 17:17697094 | - | - | - | - | 0.500000 | - | - | - | - | Smith-Magenis syndrome | *607642 |  |  |
| *RECQL4* | RecQ Like Helicase 4 | 8q24.3 | rs34948955 | 8:145740622 | 0.00159744 | 0.00549811 | 0.00698396 | 0.005333 | 0.003284 | synonymous_variant | - | - | - | Baller-Gerold syndrome, RAPADILINO syndrome, Rothmund-Thomson syndrome | *603780 | Mann MB, Hodges CA, Barnes E, Vogel H, Hassold TJ, Lou G. Defective sister-chromatid cohesion, aneuploidy and cancer predisposition in a mouse model of type II Rothmund-Thomson syndrome. Hum Mol Genet 2005;14:813-825. |  |
| *RERE* | Arginine-Glutamic Acid Dipeptide Repeats | 1p36.23 | rs139299521 | 1:8424822 | - | - | - | - | 0.002463 | - | - | - | - | Chromosome 1p36 Deletion Syndrome, Charge Syndrome, Neurodevelopmental Disorder with or Without Anomalies of the Brain, Eye, or Heart | *605226 |  |  |
| *SEPT9* | Septin 9 | 17q25.2-q25 .3 | rs61744333 | 17:75398265 | 0.000599042 | 0.00202144 | 0.00178086 | 0.00196 | 0.006568 | synonymous_variant | - | - | - | Amyotrophy, hereditary neuralgic | *604061 |  |  |
|  |  |  | rs8070026 | 17:75277611 | 0.163938 | 0.0873011 | 0.150649 | 0.048 | 0.121993 | 5_prime_UTR_variant | - | - | - |  |  |  |  |
| *SHMT1* | Serine Hydroxymethyltransferase 1 | 17p11.2 | rs8080285 | 17:18234028 | 0.0924521 | 0.026609 | 0.0748126 | 0.031 | 0.068966 | intron_variant | - | - | - | - | *182144 | Boyles AL, Wilcox AJ, Taylor JA, Shi M, Weinberg CR, Meyer K, et al. Oral facial clefts and gene polymorphisms in metabolism of folate/one-carbon and vitamin A: a pathway-wide association study. Genet Epidemiol. 2009;33(3):247-255. |  |
|  |  |  | rs2273026 | 17:18256979 | 0.135184 | 0.122439 | 0.109661 | 0.119 | 0.103448 | splice_region_variant | - | - | - |  |  |  |  |
| *SLC35D1* | Solute Carrier Family 35 Member D1 | 1p31.3 | rs7539628 | 1:67507914 | 0.0726837 | 0.0166411 | 0.0643775 | 0.021 | 0.057471 | splice_region_variant | - | - | - | Schneckenbecken dysplasia | *610804 |  |  |
| *SLC39A13* | Solute Carrier Family 39 Member 13 | 11p11.2 | rs141246142 | 11:47433581 | 0.00119808 | 0.000237091 | 0.000839793 | 0.0002804 | 0.002467 | missense_variant | Tolerated | Benign | Tolerated | Spondylocheirodysplasia, Ehlers-Danlos syndrome-like | *608735 |  |  |
| *SMARCA4* | SWI/SNF Related, Matrix Associated, Actin Dependent Regulator Of Chromatin, Subfamily A, Member 4 | 19p13.2 | rs538345417 | 19:11098481 | 0.000199681 | 0.0000513899 | 0.000161991 | 0.00007545 | 0.000821 | synonymous_variant | - | - | - | Coffin-Siris syndrome 1 | *603254 |  |  |
|  |  |  | rs28997582 | 19:11145691 | 0.0471246 | 0.0609736 | 0.0496087 | 0.061 | 0.045156 | synonymous_variant | - | - | - |  |  |  |  |
|  |  |  | rs9105 | 19:11169514 | 0.0203674 | 0.0401415 | 0.0404374 | 0.039 | 0.037767 | synonymous_variant | - | - | - |  |  |  |  |
| *SMC3* | Structural Maintenance Of Chromosomes 3 | 10q25.2 | rs75817442 | 10:112350758 | 0.00339457 | 0.000950586 | 0.0039393 | 0.001235 | 0.002463 | synonymous_variant | - | - | - | Cornelia de Lange syndrome 3 with or without Midline Brain Defects; Cornelia de Lange syndrome | *606062 |  |  |
|  |  |  | rs11195194 | 10:112337693 | 0.141973 | 0.152759 | 0.118142 | 0.149 | 0.113300 | intron_variant | - | - | - |  |  |  |  |
|  |  |  | rs78663177 | 10:112338377 | 0.0499201 | 0.0418455 | 0.0605464 | 0.042 | 0.059113 | intron_variant | - | - | - |  |  |  |  |
|  |  |  | rs11815960 | 10:112343116 | 0.0904553 | 0.0513637 | 0.0924082 | 0.055 | 0.079770 | intron_variant | - | - | - |  |  |  |  |
|  |  |  | rs11195199 | 10:112343591 | 0.148163 | 0.150825 | 0.117032 | 0.148 | 0.115764 | splice_region_variant | - | - | - |  |  |  |  |
|  |  |  | rs11195200 | 10:112343923 | 0.148163 | 0.151696 | 0.116768 | 0.148 | 0.115764 | intron_variant | - | - | - |  |  |  |  |
|  |  |  | rs75323904 | 10:112349422 | 0.0497204 | 0.042421 | 0.0603153 | 0.044 | 0.059113 | synonymous_variant | - | - | - |  |  |  |  |
|  |  |  | rs11195213 | 10:112360936 | 0.148962 | 0.154212 | 0.117392 | 0.15 | 0.115764 | intron_variant | - | - | - |  |  |  |  |
| *SOX3* | SRY-Box Transcription Factor 3 | Xq27.1 | rs142709662 | X:139587149 | 0.00211921 | 0.000236567 | 0.00116539 | 0.0004704 | 0.000998 | missense_variant | Tolerated | Benign | Tolerated | Septooptic Dysplasia | *313430 |  |  |
| *STAT3* | Signal Transducer And Activator Of Transcription 3 | 17q21.2 | rs185979539 | 17:40481711 | 0.000199681 | 0.0000974619 | 0.000129191 | 0.0001071 | - | intron_variant | - | - | - | Hyper-Ige Recurrent Infection Syndrome 1, Autosomal Dominant | *102582 |  |  |
|  |  |  | rs3830585 | 17:40475651 | 0.401957 | 0.244593 | 0.339821 | 0.259 | 0.334975 | splice_region_variant | - | - | - |  |  |  |  |
|  |  |  | rs12721576 | 17:40489741 | 0.0842652 | 0.0192341 | 0.0759269 | 0.024 | 0.064860 | intron_variant | - | - | - |  |  |  |  |
| *STRA6* | Signaling Receptor And Transporter Of Retinol STRA6 | 15q24.1 | rs11857410 | 15:74488424 | 0.0617013 | 0.145709 | 0.165208 | 0.145 | 0.105090 | synonymous_variant | - | - | - | Microphthalmia, syndromic 9 | *610745 |  |  |
| *TBC1D24* | TBC1 Domain Family Member 24 | 16p13.3 | rs201060500 | 16:2546934 | 0.000599042 | 0.000658028 | 0.00174419 | 0.0007261 | 0.008210 | missense_variant | Tolerated | Benign | Damaging | Deafness, Onychodystrophy, Osteodystrophy, Mental Retardation, and Seizures Syndrome | *613577 |  |  |
|  |  |  | rs13339237 | 16:2546356 | 0.0650958 | 0.016215 | 0.0564063 | 0.019 | 0.046798 | synonymous_variant | - | - | - |  |  |  |  |
| *TBCE* | Tubulin Folding Cofactor E | 1q42.3 | rs140662460 | 1:235612070 | - | - | - | - | 0.001642 | - | - | - | - | Hypoparathyroidism-retardation-dysmorphism syndrome | *604934 |  |  |
|  |  |  | rs6697653 | 1:235600590 | 0.0355431 | 0.0205763 | 0.0393037 | 0.023 | 0.036946 | intron_variant | - | - | - |  |  |  |  |
|  |  |  | rs6666168 | 1:235600741 | 0.0361422 | 0.0206083 | 0.0395477 | 0.023 | 0.036946 | synonymous_variant | - | - | - |  |  |  |  |
| *TMEM67* | Transmembrane Protein 67 | 8q22.1 | rs35793208 | 8:94792887 | 0.0103834 | 0.0151486 | 0.0139158 | 0.015 | 0.013136 | missense_variant | Tolerated | Benign | Tolerated | Meckel syndrome, type 3 | *609884 |  |  |
| *TNXB* | Tenascin XB | 6p21.33 | rs9501600 | 6:31976899 | 0.299521 | 0.175229 | 0.238279 | 0.067 | 0.216374 | missense_variant | - | - | - | Ehlers-Danlos-like syndrome due to tenascin-X deficiency | *600985 |  |  |
|  |  |  | rs113395614 | 6:31977553 | 0.0145767 | 0.0169488 | 0.0169052 | 0.017 | 0.025084 | synonymous_variant | - | - | - |  |  |  |  |
|  |  |  | rs144415985 | 6:32009634 | - | 0.0496944 | 0.0462885 | 0.055 | 0.115625 | missense_variant | Damaging | Probably damaging | Damaging |  |  |  |  |
|  |  |  | rs199953230 | 6:32009651 | - | 0.0672394 | 0.0616805 | 0.076 | 0.153285 | missense_variant | Tolerated | Benign | Tolerated |  |  |  |  |
|  |  |  | rs200523717 | 6:32009661 | - | 0.0996667 | 0.089726 | 0.126 | 0.168067 | missense_variant | Damaging | Probably damaging | Damaging |  |  |  |  |
|  |  |  | rs200766440 | 6:32010016 | 0.230631 | 0.102352 | 0.13845 | 0.061 | 0.114428 | intron_variant | - | - | - |  |  |  |  |
|  |  |  | rs7742632 | 6:32010572 | - | 0.108266 | 0.16824 | 0.071 | 0.076923 | missense_variant | Tolerated | Possibly damaging | Damaging |  |  |  |  |
|  |  |  | rs17207923 | 6:32036788 | 0.0357428 | 0.0208209 | 0.0338737 | 0.021 | 0.042693 | missense_variant | Tolerated | Possibly damaging | Tolerated |  |  |  |  |
|  |  |  | rs9267799 | 6:32046944 | 0.0449281 | 0.0270473 | 0.0311651 | 0.028 | 0.041051 | missense_variant | Tolerated | Benign | Tolerated |  |  |  |  |
|  |  |  | rs17201602 | 6:32063681 | 0.0924521 | 0.0603274 | 0.0696575 | 0.063 | 0.100985 | missense_variant | Damaging | Probably damaging | Tolerated |  |  |  |  |
|  |  |  | rs41270461 | 6:32065023 | 0.0802716 | 0.0532442 | 0.0646215 | 0.055 | 0.094417 | missense_variant | Damaging | Possibly damaging | Tolerated |  |  |  |  |
| *TRPV4* | Transient Receptor Potential Cation Channel Subfamily V Member 4 | 12q24.11 | rs3742037 | 12:110226379 | 0.180312 | 0.172066 | 0.150036 | 0.167 | 0.146962 | synonymous_variant | - | - | - | Metatropic Dysplasia, Beckwith-Wiedemann Syndrome | *605427 |  |  |
| *TTC37* | Tetratricopeptide Repeat Domain 37 | 5q15 | c.4212+45C>T | 5:94818132 | - | - | - | - | - | - | - | - | - | Trichohepatoenteric syndrome 1 | *614589 |  |  |
| *UBB* | Ubiquitin B | 17p11.2 | rs9908960 | 17:16285404 | 0.0615016 | 0.0287594 | 0.0476143 | 0.03 | 0.062397 | synonymous_variant | - | - | - | Cleft palate, isolated, and mental retardation | *191339 |  |  |
| *WDR11* | WD Repeat Domain 11 | 10q26.12 | rs35692153 | 10:122610983 | 0.0579073 | 0.0123606 | 0.0549273 | 0.015 | 0.041872 | synonymous_variant | - | - | - | Hypogonadotropic hypogonadism 14 with or without anosmia | *606417 |  |  |
|  |  |  | rs74158348 | 10:122663529 | 0.0666933 | 0.0155705 | 0.0609732 | 0.019 | 0.042693 | intron_variant | - | - | - |  |  |  |  |
|  |  |  | rs12268298 | 10:122665489 | 0.066893 | 0.0165646 | 0.058371 | 0.02 | 0.044335 | synonymous_variant | - | - | - |  |  |  |  |
| *WFS1* | Wolframin ER Transmembrane Glycoprotein | 4p16.1 | rs35216268 | 4:6279407 | - | - | - | - | 0.001661 | - | - | - | - | Marden-Walker syndrome, Dandy-Walker syndrome | *606201 |  |  |
|  |  |  | rs56072215 | 4:6302545 | 0.0267572 | 0.0545805 | 0.0428479 | 0.055 | 0.059934 | synonymous_variant | - | - | - |  |  |  |  |
| *WT1* | WT1 Transcription Factor | 11p13 | rs2234590 | 11:32421533 | 0.0449281 | 0.0112815 | 0.043026 | 0.014 | 0.034483 | synonymous_variant | - | - | - | Denys-Drash syndrome | *607102 |  |  |
| *XYLT1* | Xylosyltransferase 1 | 16p12.3 | rs7201590 | 16:17211729 | 0.0634984 | 0.0153136 | 0.0565293 | 0.019 | 0.032841 | synonymous_variant | - | - | - | Desbuquois dysplasia 2 | *608124 |  |  |
| *ZEB2* | Zinc Finger E-Box Binding Homeobox 2 | 2q22.3 | rs910056293 | 2:145270029 | - | - | - | - | - | intron_variant | - | - | - | Mowat-Wilson syndrome | *605802 |  |  |
|  |  |  |  |  |  |  |  |  |  |  |  |  |  |  |  |  |  |
|  |  |  |  |  |  |  |  |  |  |  |  |  |  |  |  |  |  |

OMIM, Online Mendelian Inheritance in Man (http://omim.org).

An asterisk (*) before an OMIM entry number indicates a gene.

A plus sign (+) before an OMIM entry number indicates that the entry includes a description of a gene and a phenotype.
