# Supplementary material for: Identification of Novel Variants in Cleft Palate-Associated Genes in Brazilian Patients With Non-syndromic Cleft Palate Only
Source: Front Cell Dev Biol. 2021 Jul 8;9:638522. doi: 10.3389/fcell.2021.638522 (PMC8297955; doi:10.3389/fcell.2021.638522)
Supplement: Supplementary file 5 [file Data_Sheet_3.DOCX]

| **Supplementary Table 3.** Variants in cleft lip-palate-associated genes found exclusively in the exome of patients with nonsyndromic cleft palate only (NSCPO). | | | | | | | | | | | | | | | |
| --- | --- | --- | --- | --- | --- | --- | --- | --- | --- | --- | --- | --- | --- | --- | --- |
| **Gene** | **Protein** | **Cytogenetic Location** | **Variants** | **Chr:Pos** | **MAF 1K** | **MAF gnomAD Exomes** | **MAF gnomAD Genomes** | **MAF ExAC** | **MAF ABraOM** | **SIFT** | **Polyphen2** | **Mutation Taster** | **Syndrome** | **OMIM Number** | **Reference** |
| *ABCA4* | ATP Binding Cassette Subfamily A Member 4 | 1p22.1 | rs61754045 | 1:94496509 | 0.000998403 | 0.00475243 | 0.00448995 | 0.004308 | 0.008418 | - | - | - | - | *601691 | Beaty TH, Murray JC, Marazita ML, Munger RG, Ruczinski I, Hetmandki JB, et al. A genome-wide association study of cleft lip with and without cleft palate identifies risk variants near MAFB and ABCA4. Nat Genet 2010;42:525-529. |
|  |  |  | [rs1801581](http://www.ncbi.nlm.nih.gov/projects/SNP/snp_ref.cgi?rs=rs1801581) | 1:94512565 | 0.0153754 | 0.030364 | 0.0291134 | 0.031 | 0.033662 | Tolerated | Benign | Tolerated |  |  |  |
| *ACTA1* | Actin Alpha 1, Skeletal Muscle | 1q42.13 | rs146956806 | 1:229568625 | 0.00119808 | 0.0000853665 | 0.0000323478 | 0.00007413 | 0.002463 | - | - | - | Van Der Woude syndrome 1, Fetal Akinesia Deformation sequence 1 | *102610 |  |
| *ACTG1* | Actin Gamma 1 | 17q25.3 | rs55978907 | 17:79477575 | 0.0211661 | 0.0160776 | 0.0121715 | 0.012 | 0.028630 | - | - | - | Baraitser-Winter Syndrome 2 | *102560 | Verloes A, Donato N, Masliah-Planchon J, Jongmans M, Abdul-Raman O, Albrecht B, et al. Baraitser-Winter cerebrofrontofacial syndrome: delineation of the spectrum in 42 cases. Eur J Hum Genet. 2015;23(3):292-301. |
|  |  |  | rs80345231 | 17:79478181 | 0.0507189 | 0.0221765 | 0.0359868 | 0.023 | 0.052545 | - | - | - |  |  |  |
| *ACTN1* | Actinin Alpha 1 | 14q24.1 | rs36039812 | 14:69347632 | 0.0303514 | 0.0451186 | 0.0308478 | 0.045 | 0.040230 | - | - | - | - | *102575 | Liu D, Wang M, Yuan Y, Schwender H, Wang H, Zhou Z, et al., Gene–gene interaction among cell adhesion genes and risk of nonsyndromic cleft lip with or without cleft palate in Chinese case‐parent trios. Mol Genet Genomic Med. 2019; 7(10):e00872. |
| *ADAMTS20* | ADAM Metallopeptidase With Thrombospondin Type 1 Motif 20 | 12q12 | rs7310011 | 12:43822171 | 0.0365415 | 0.0340933 | 0.0233295 | 0.034 | 0.027915 | Tolerated | Possibly damaging | Tolerated | - | *611681 | *Wolf ZT, Brand HA, Shaffer JR, Leslie EJ, Arzi B, Willet CE, et al. Genome-wide association studies in dogs and humans identify ADAMTS20 as a risk variant for cleft lip and palate. PLoS Genet 2015;11:e1005059. |
| *ADH1C* | Alcohol Dehydrogenase 1C (Class I), Gamma Polypeptide | 4q23 | rs2298753 | 4:100257907 | 0.0642971 | 0.10112 | 0.0875662 | 0.098 | 0.065681 | - | - | - |  | *103730 | **Enomoto H, Nelson CM, Somerville RP, Mielke K, Dixon LJ, Powell K, et al. Cooperation of two ADAMTS metalloproteases in closure of the mouse palate identifies a requirement for versican proteolysis in regulating palatal mesenchyme proliferation. Development 2010;137:4029-4038. |
| *AHI1* | Abelson Helper Integration Site 1 | 6q23.3 | rs140280929 | 6:135732531 | 0.00499201 | 0.00117117 | 0.00403564 | 0.001399 | 0.000821 | - | - | - | Joubert syndrome 1, Joubert syndrome 3 | *608894 | Jugessur A, Shi M, Gjessing HK, Lie RT, Wilcox AJ, Weinberg CR, et al. Genetic determinants of facial clefting: analysis of 357 candidate genes using two national cleft studies from Scandinavia. 2009;4(4):e5385 |
|  |  |  | rs7772864 | 6:135679228 | 0.066893 | 0.0145775 | 0.0553615 | 0.018 | 0.044335 | - | - | - |  |  | Poretti A, Vitiello G, Hennekam RCM, Arrigoni F, Bertini E, Borgatti R et al. Delineation and Diagnostic Criteria of Oral-Facial-Digital Syndrome Type VI . Orphanet J Rare Dis. 2012;7:4 |
| *ANK1* | Ankyrin 1 | 8p11.21 | rs75147697 | 11:44289183 | 0.0800719 | 0.08027 | 0.0852199 | 0.083 | 0.073892 | - | - | - | Spherocytosis, type 1 | *612641 |  |
| *ANOS1/KAL1* | Anosmin 1 | Xp22.31 | rs6640177 | X:8505016 | 0.00635762 | 0.00208424 | 0.00623682 | 0.00222 | 0.006986 | - | - | - | Hypogonadotropic hypogonadism 1 with or without anosmia | *300836 |  |
| *APC* | APC Regulator Of WNT Signaling Pathway | 5q22.2 | rs730881230 | 5:112111310 | - | 0.245614 | 0.0264216 | 0.204 | 0.055932 | - | - | - | - | *611731 |  |
| *ARHGAP31* | Rho GTPase Activating Protein 31 | 3q13.33 | rs16829782 | 3:119101925 | 0.0579073 | 0.0800747 | 0.0677605 | 0.075 | 0.066502 | - | - | - | Adams-Oliver syndrome 1 | *610911 | Vijayan V, Ummer R, Weber R, Silva R, Letra A. Association of WNT Pathway Genes With Nonsyndromic Cleft Lip With or Without Cleft Palate. Cleft Palate Craniofac J. 2018; 55(3):335-341. |
| *ASXL1* | ASXL Transcriptional Regulator 1 | 20q11.21 | rs62206933 | 20:31023500 | 0.0401358 | 0.0277178 | 0.0189264 | 0.023 | 0.023810 | - | - | - | Bohring-opitz syndrome | *612990 |  |
| *ATM* | ATM Serine/Threonine Kinase | 11q22.3 | rs4986761 | 11:108124761 | 0.00439297 | 0.00802656 | 0.00775444 | 0.007808 | 0.013957 | Tolerated | Benign | Tolerated | Nijmegen Breakage Syndrome | *607585 | Abdel-Wahab O, Gao J, Adli M, Dey A, Trimarchi T, Chung YR, et al. Deletion of Asxl1 results in myelodysplasia and severe developmental defects in vivo. J Exp Med 2013;210:2641-2659. |
|  |  |  | rs3092910 | 11:108180917 | 0.0061901 | 0.00495319 | 0.00587893 | 0.005181 | 0.004105 | - | - | - |  |  |  |
|  |  |  | rs2234997 | 11:108106443 | 0.0623003 | 0.0156898 | 0.0557995 | 0.019 | 0.051724 | Tolerated | Benign | Tolerated |  |  |  |
|  |  |  | rs1800735 | 11:108115778 | 0.0623003 | 0.016066 | 0.0559576 | 0.018 | 0.051724 | - | - | - |  |  |  |
| *BMP7* | Bone Morphogenetic Protein 7 | 20q13.31 | rs41274738 | 20:55803299 | 0.0173722 | 0.0288055 | 0.0270043 | 0.029 | 0.031199 | - | - | - | - | *112267 |  |
| *BRCA1* | BRCA1 DNA Repair Associated | 17q21.31 | rs1799950 | 17:41246481 | 0.0217652 | 0.0466883 | 0.0514502 | 0.044 | 0.049261 | Damaging | Probably damaging | Tolerated | Fanconi Anemia, Complementation Group a | *113705 | Yu Q, He S, Zeng N, Ma J, Zhang B, Shi B, et al. BMP7 Gene involved in nonsyndromic orofacial clefts in Western Han Chinese. Med Oral Patol Oral Cir Bucal. 2015;20(3):e298-304. |
| *BRCA2* | BRCA2 DNA Repair Associated | 13q13.1 | rs34351119 | 13:32913910 | 0.00678914 | 0.00172745 | 0.0075596 | 0.002323 | 0.006568 | - | - | - | Tracheoesophageal Fistula with or without Esophageal Atresia; Fanconi anemia, Complementation group a | *600185 | Rodriguez N, Maili L, Chiquet BT, Blanton SH, Hecht JT, Letra A. BRCA1 and BRCA2 Gene Variants and Nonsyndromic Cleft Lip/Palate. 2018;110(12):1043-1048. |
|  |  |  | rs1801499 | 13:32910721 | 0.0734824 | 0.0526318 | 0.0307856 | 0.052 | 0.045156 | - | - | - |  |  | Rodriguez N, Maili L, Chiquet BT, Blanton SH, Hecht JT, Letra A. BRCA1 and BRCA2 Gene Variants and Nonsyndromic Cleft Lip/Palate. 2018;110(12):1043-1048. |
| *CC2D2A* | Coiled-Coil And C2 Domain Containing 2A | 4p15.32 | rs114335547 | 4:15512850 | 0.00379393 | 0.00685787 | 0.0076496 | 0.00599 | 0.004926 | - | - | - | COACH syndrome Joubert syndrome 9 | *612013 |  |
| *CD96* | CD96 Molecule | 3q13.1-q13. 2 | rs9831496 | 3:111298145 | 0.139177 | 0.127908 | 0.0801731 | 0.124 | 0.106732 | - | - | - | C syndrome | *606037 |  |
| *CDON* | Cell Adhesion Associated, Oncogene Regulated | 11q24.2 | rs4426144 | 11:125888213 | 0.11222 | 0.108722 | 0.104464 | 0.106 | 0.098522 | - | - | - | Holoprosencephaly 11 | *608707 |  |
|  |  |  | rs3740909 | 11:125889526 | 0.112021 | 0.108635 | 0.10438 | 0.106 | 0.098522 | Tolerated | Benign | Tolerated |  |  | Zhang W, Hong M, Bae GU, Kang JS, Krauss RS. Boc modifies the holoprosencephaly spectrum of Cdo mutant mice. Dis Model Mech 2011;4:368-380. |
|  |  |  | rs3740910 | 11:125889673 | 0.112021 | 0.108696 | 0.104593 | 0.106 | 0.096059 | - | - | - |  |  |  |
| *CEP290* | Centrosomal Protein 290 | 12q21.32 | rs150138016 | 12:88484613 | 0.00938498 | 0.00168646 | 0.00771565 | 0.001511 | 0.004105 | - | - | - | Joubert syndrome 1, Meckel syndrome, type 1, Meckel syndrome, type4 | *610142 |  |
| *CHRNG* | Cholinergic Receptor Nicotinic Gamma Subunit | 2q37.1 | rs2289080 | 2:233406178 | 0.0465256 | 0.0332797 | 0.0273968 | 0.032 | 0.021346 | Tolerated | Benign | Damaging | Multiple pterygium syndrome, lethal type | *100730 |  |
| *COL1A2* | Collagen Type I Alpha 2 Chain | 7q21.3 | rs77347506 | 7:94040509 | 0.00978434 | 0.00197762 | 0.00924848 | 0.001818 | 0.007500 | - | - | - | - | *120160 |  |
|  |  |  | rs1800238 | 7:94047050 | 0.0992412 | 0.0511339 | 0.0361944 | 0.044 | 0.024631 | - | - | - |  |  |  |
|  |  |  | rs1800248 | 7:94056349 | 0.0994409 | 0.122429 | 0.120995 | 0.122 | 0.074713 | - | - | - |  |  |  |
| *COLEC11* | Collectin Subfamily Member 11 | 2p25.3 | rs114716171 | 2:3691669 | 0.00119808 | 0.00343864 | 0.0040359 | 0.003492 | 0.008210 | - | - | - | 3MC syndrome 2 | *612502 |  |
|  |  |  | rs73138818 | 2:3651225 | 0.0349441 | 0.0190187 | 0.0230431 | 0.027 | 0.019704 | - | - | - |  |  |  |
|  |  |  | rs17017791 | 2:3685175 | 0.0419329 | 0.0109271 | 0.0353379 | 0.013 | 0.036946 | - | - | - |  |  |  |
|  |  |  | rs111685093 | 2:3688837 | 0.0509185 | 0.0115915 | 0.0528306 | 0.012 | 0.031250 | - | - | - |  |  |  |
| *CTNNB1* | Catenin Beta 1 | 3p22.1 | rs4135386 | 3:41280807 | 0.00159744 | 0.00385054 | 0.00380965 | 0.003542 | 0.003284 | - | - | - | - | *116806 |  |
| *CTNND1* | Catenin Delta 1 | 11q12.1 | rs111562285 | 11:57505222 | 0.00658946 | 0.00219657 | 0.00933221 | 0.002712 | 0.009852 | - | - | - | Blepharocheilodontic Syndrome 1, Blepharocheilodontic syndrome 2 | *601045 | Vijayan V, Ummer R, Weber R, Silva R, Letra A. Association of WNT Pathway Genes With Nonsyndromic Cleft Lip With or Without Cleft Palate. Cleft Palate Craniofac J. 2018; 55(3):335-341. |
|  |  |  | rs11570223 | 11:57582924 | 0.000998403 | 0.00265053 | 0.0012623 | 0.002525 | 0.003284 | - | - | - |  |  |  |
| *DHODH* | Dihydroorotate Dehydrogenase (Quinone) | 16q22.2 | rs61733129 | 16:72057421 | 0.0233626 | 0.0274182 | 0.0264451 | 0.027 | 0.026273 | Damaging | Probably damaging | Damaging | Miller syndrome | *126064 |  |
| *DMGDH* | Dimethylglycine Dehydrogenase | 5q14.1 | rs2303128 | 5:78320085 | 0.111022 | 0.0478822 | 0.0641266 | 0.052 | 0.064860 | - | - | - | - | *605849 |  |
|  |  |  |  |  |  |  |  |  |  |  |  |  |  |  | Marini NJ, Yang W, Asrani J, Witte JS, Rine J, Lammer E, et al., Sequence variation in folate pathway genes and risks of human cleft lip with or without cleft palate. Am J Med Genet A. 2016;170(11):2777-2787. |
| *DYNC2H1* | Dynein Cytoplasmic 2 Heavy Chain 1 | 11q22.3 | rs12146610 | 11:102988504 | 0.0329473 | 0.0492284 | 0.0370801 | 0.05 | 0.066502 | Tolerated | Probably damaging | Damaging | Short-rib thoracic dysplasia 3 with or without polydactyly | *603297 |  |
|  |  |  | rs11225578 | 11:103044038 | 0.127596 | 0.114813 | 0.0948237 | 0.114 | 0.079639 | - | - | - |  |  |  |
| *EDAR* | Ectodysplasin A Receptor | 2q13 | rs79798733 | 2:109526983 | 0.00758786 | 0.00219864 | 0.00899211 | 0.002759 | 0.005747 | Damaging | Possibly damaging | Damaging | Ectodermal Dysplasia | *604095 |  |
|  |  |  | rs748225 | 2:109539913 | 0.0914537 | 0.0578946 | 0.0662117 | 0.061 | 0.082923 | - | - | - |  |  |  |
| *EGFR* | Epidermal Growth Factor Receptor | 7p11.2 | rs7801956 | 7:55214443 | 0.0553115 | 0.0764511 | 0.0623505 | 0.077 | 0.075534 | - | - | - | - | *131550 |  |
|  |  |  | rs17290225 | 7:55238253 | 0.0249601 | 0.0365651 | 0.0338594 | 0.037 | 0.056572 | - | - | - |  |  |  |
| *EP300* | E1A Binding Protein P300 | 22q13.2 | rs5758235 | 22:41513175 | 0.0902556 | 0.0254462 | 0.0462502 | 0.027 | 0.032020 | - | - | - | Charge syndrome | *602700 |  |
|  |  |  | rs17002316 | 22:41545750 | 0.0902556 | 0.0254373 | 0.0466072 | 0.027 | 0.032020 | - | - | - |  |  |  |
|  |  |  | rs20554 | 22:41553259 | 0.086861 | 0.0244094 | 0.0453723 | 0.026 | 0.028736 | - | - | - |  |  |  |
|  |  |  | rs1046088 | 22:41574383 | 0.0113818 | 0.0238494 | 0.028297 | 0.024 | 0.018062 | Tolerated | Benign | Damaging |  |  |  |
| *ESR1* | Estrogen Receptor 1 | 6q25.1-q25. 2 | rs9397459 | 6:152265659 | 0.0509185 | 0.019924 | 0.0443684 | 0.022 | 0.029557 | - | - | - | - | ✚133430 |  |
| *FBN1* | Fibrillin 1 | 15q21.1 | rs75655780 | 15:48812974 | 0.00838658 | 0.00144659 | 0.00555412 | 0.001952 | 0.002463 | - | - | - | Loeys-Dietz Syndrome, Marden-Walker syndrome, Marfan Syndrome | *134797 | Osoegawa K, Vessere GM, Utami KH, Mansilla MA, Johnson MK, Riley BM, et al. Identification of novel candidate genes associated with cleft lip and palate using array comparative genomic hybridisation. J Med Genet 2008;45:81-86. |
|  |  |  | rs12324002 | 15:48818329 | 0.0081869 | 0.00155168 | 0.00684622 | 0.00229 | 0.004105 | Damaging | Benign | Damaging |  |  |  |
|  |  |  | rs77105137 | 15:48755472 | 0.0467252 | 0.0702336 | 0.0693053 | 0.068 | 0.088670 | - | - | - |  |  |  |
|  |  |  | rs116842662 | 15:48888610 | 0.0151757 | 0.0158777 | 0.0166893 | 0.016 | 0.016420 | - | - | - |  |  |  |
|  |  |  | rs25398 | 15:48936811 | 0.0700879 | 0.0158343 | 0.0653523 | 0.021 | 0.046798 | - | - | - |  |  |  |
| *FGD1* | FYVE, RhoGEF And PH Domain Containing 1 | Xp11.22 | rs61734180 | X:54472671 | 0.00450331 | 0.0012826 | 0.00575878 | 0.001557 | 0.003992 | - | - | - | Aarskog-Scott syndrome | *300546 |  |
|  |  |  | rs2239809 | X:54496989 | 0.209007 | 0.154999 | 0.112297 | 0.141 | 0.153693 | - | - | - |  |  |  |
| *FGD4* | FYVE, RhoGEF And PH Domain Containing 4 | 5q35.1 | rs115383807 | 12:32778050 | 0.0081869 | 0.0023033 | 0.00946872 | 0.002891 | 0.008210 | - | - | - | - | *603726 |  |
| *FGFR3* | Fibroblast Growth Factor Receptor 3 | 4p16.3 | rs147769045 | 4:1803385 | 0.00599042 | 0.0015189 | 0.00685995 | 0.001722 | 0.002463 | - | - | - | Crouzon syndrome with acanthosis nigricans | *134934 | Wan W, Yang S, Liu J, Cui YG, Zhou XP, Guo FF, et al. Correlation of the SNPs of FGFR1, FGF10, FGF18 with nonsyndromic cleft lip with or without palate in Chinese population. Beijing Da Xue Xue Bao Yi Xue Ban. 2009;41(4):409-13. |
| *FKTN* | Fukutin | 9q31.2 | rs34006675 | 9:108366499 | 0.0371406 | 0.013749 | 0.0323581 | 0.015 | 0.024631 | Tolerated | Possibly damaging | Tolerated | Walker-Warburg Syndrome | *607440 |  |
| *FLNB* | Filamin B | 3p14.3 | c.2813T>C | 3:58104666 | - | - | - | - | - | - | - | - | Atelosteogenesis, Larsen syndrome, Spondylocarpotarsal synostosis syndrome | *603381 | van Reeuwijk J, Brunner HG, van Bokhoven H. Glyc-O-genetics of Walker-Warburg syndrome. Clin Genet 2005;67:281-289. |
|  |  |  | rs114342439 | 3:58110260 | 0.0267572 | 0.0598103 | 0.0490538 | 0.058 | 0.070608 | - | - | - |  |  |  |
|  |  |  | rs60183346 | 3:58117724 | 0.0886581 | 0.0666934 | 0.0343536 | 0.034 | 0.038246 | - | - | - |  |  |  |
| *FOXH1* | Forkhead Box H1 | 8q24.3 | rs1871545 | 8:145700674 | 0.0427316 | 0.066122 | 0.0573399 | 0.065 | 0.074959 | - | - | - | Holoprosencephaly | *603621 |  |
| *FRAS1* | Fraser Extracellular Matrix Complex Subunit 1 | 4q21.21 | rs114854941 | 4:79410108 | 0.000599042 | 0.00302786 | 0.00332687 | 0.003382 | 0.001642 | - | - | - | Fraser syndrome | *607830 |  |
|  |  |  | rs35774552 | 4:79300899 | 0.0255591 | 0.0636718 | 0.0610978 | 0.064 | 0.064860 | - | - | - |  |  | Caruana G, Farlie PG, Hart AH, Bagheri-Fam S, Wallace MJ, Dobbie MS, et al. Genome-wide ENU mutagenesis in combination with high density SNP analysis and exome sequencing provides rapid identification of novel mouse models of developmental disease. PLoS One 2013;8:e55429. |
| *FUZ* | Fuzzy Planar Cell Polarity Protein | 19q13.33 | rs12973635 | 19:50314738 | 0.0329473 | 0.0465808 | 0.03498 | 0.048 | 0.033662 | - | - | - | Short-Rib Thoracic Dysplasia 6 with or Without Polydactyly, Sacral Defect with Anterior Meningocele | *610622 |  |
| *HLA-B* | Major Histocompatibility Complex, Class I, B | 6p21.33 | rs41551018 | 6:31324051 | 0.0678914 | 0.0565297 | 0.0566414 | 0.061 | 0.031199 | Damaging | Benign | Damaging | - | ✚142830 |  |
|  |  |  | rs41541519 | 6:31324064 | 0.0682907 | 0.0575323 | 0.0569952 | 0.063 | 0.032841 | Damaging | Benign | Tolerated |  |  | Sakata Y, Tokunaga K, Yonehara Y, Bannai M, Tsuchiya N, Susami T, et al. Significant association of HLA-B and HLA-DRB1 alleles with cleft lip with or without cleft palate. 1999;53(2):147-52. |
| *HLA-DRB1* | Major Histocompatibility Complex, Class II, DR Beta 1 | 6p21.32 | rs16822972 | 6:32549392 | - | 0.0172143 | 0.0129505 | 0.023 | 0.081104 | - | - | - | - | *142857 |  |
|  |  |  | rs77689370 | 6:32549563 | - | 0.0650287 | 0.0793799 | 0.095 | 0.068966 | - | - | - |  |  | Sakata Y, Tokunaga K, Yonehara Y, Bannai M, Tsuchiya N, Susami T, et al. Significant association of HLA-B and HLA-DRB1 alleles with cleft lip with or without cleft palate. 1999;53(2):147-52. |
|  |  |  | rs150747106 | 6:32552039 | - | 0.149196 | 0.140136 | 0.223 | 0.243842 | Damaging | Probably damaging | Tolerated |  |  |  |
|  |  |  | rs148093782 | 6:32557446 | - | 0.019556 | 0.255107 | 0.039 | 0.149425 | Damaging | Probably damaging | Tolerated |  |  |  |
| *IFT122* | Intraflagellar Transport 122 | 3q21.3-q22.1 | rs138793724 | 3:129180119 | 0.00239617 | 0.00307025 | 0.0030348 | 0.00327 | 0.000821 | - | - | - | Short-Rib Thoracic Dysplasia 12 | *606045 |  |
|  |  |  | rs151309730 | 3:129183519 | - | 0.0000284239 | 0.0000968679 | 0.00002471 | - | Tolerated | Probably damaging | Damaging |  |  |  |
|  |  |  | rs139319087 | 3:129195610 | 0.00239617 | 0.00101973 | 0.00329543 | 0.001203 | 0.002463 | - | - | - |  |  |  |
|  |  |  | rs61744639 | 3:129196984 | 0.00139776 | 0.00510507 | 0.00642433 | 0.004752 | 0.004105 | Damaging | Probably damaging | Damaging |  |  |  |
|  |  |  | rs2301570 | 3:129195663 | 0.122804 | 0.0743879 | 0.101579 | 0.08 | 0.084565 | - | - | - |  |  |  |
| *INPP5E* | Inositol Polyphosphate-5-Phosphatase E | 9q34.3 | rs10870199 | 9:139328551 | 0.123802 | 0.144084 | 0.0907885 | 0.137 | 0.115764 | - | - | - | Joubert syndrome 1, Joubert syndrome 3, Coach syndrome | *613037 |  |
| *JAG1* | Jagged Canonical Notch Ligand 1 | 20p12.2 | rs35357944 | 20:10627772 | 0.00299521 | 0.00562808 | 0.00358365 | 0.005831 | 0.009031 | - | - | - | Alagille syndrome | *601920 |  |
|  |  |  | rs557999553 | 20:10622081 | - | - | - | - | - | - | - | - |  |  | Sahoo T, Theisen A, Sanchez-Lara P, Marble M, Schweitzer DN, Torchia BS, et al. Microdeletion 20p12.3 involving BMP2 contributes to syndromic forms of cleft palate. Am J Med Genet A. 2011;155A(7):1646-53. |
|  |  |  | rs1801140 | 20:10625804 | 0.100839 | 0.111844 | 0.0813074 | 0.109 | 0.091954 | - | - | - |  |  |  |
| *KDM6A* | Lysine Demethylase 6A | Xp11.3 | rs34922269 | X:44922880 | 0.00715232 | 0.00174053 | 0.00625492 | 0.001928 | 0.005988 | Damaging | Probably damaging | Damaging | Kabuki syndrome 1, Kabuki syndrome 2 | *300128 |  |
|  |  |  | rs372323256 | X:44918222 | - | - | - | - | 0.123145 | - | - | - |  |  |  |
|  |  |  | rs2230018 | X:44929077 | 0.155497 | 0.133169 | 0.117691 | 0.136 | 0.083832 | Tolerated | Benign | Tolerated |  |  |  |
| *KMT2A* | Lysine Methyltransferase 2A | 11q23.3 | rs9332801 | 11:118355642 | 0.0363419 | 0.0502223 | 0.0416963 | 0.05 | 0.056650 | - | - | - | Kabuki syndrome 1, Cornelia De Lange syndrome 1,Cornelia de Lange syndrome | *159555 |  |
| *KRT14* | Keratin 14 | 17q21.2 | rs75795684 | 17:39740629 | 0.0443291 | 0.0104195 | 0.0393459 | 0.013 | 0.032841 | - | - | - | - | *148066 |  |
|  |  |  | rs35849957 | 17:39741280 | 0.0597045 | 0.0144111 | 0.0542348 | 0.018 | 0.040230 | - | - | - |  |  |  |
| *LARGE1* | LARGE Xylosyl- And Glucuronyltransferase 1 | 22q12.3 | rs147579402 | 22:33828280 | 0.00279553 | 0.00596334 | 0.00768883 | 0.006054 | 0.004926 | - | - | - | Walker-Warburg Syndrome | *603590 |  |
|  |  |  | rs36002910 | 22:34000460 | 0.014377 | 0.0236191 | 0.0245139 | 0.023 | 0.013957 | - | - | - |  |  | van Reeuwijk J, Brunner HG, van Bokhoven H. Glyc-O-genetics of Walker-Warburg syndrome. Clin Genet 2005;67:281-289. |
| *LBR* | Lamin B Receptor | q42.12 | rs112582692 | 1:225600169 | 0.0295527 | 0.0470074 | 0.0434164 | 0.048 | 0.040230 | - | - | - | Short-Rib Thoracic Dysplasia 1 with or Without Polydactyly, Pelger-Huet Anomaly | *600024 |  |
| *MMP2* | Matrix Metallopeptidase 2 | 16q12.2 | rs1053605 | 16:55519607 | 0.0892572 | 0.0723411 | 0.0615813 | 0.074 | 0.081281 | - | - | - | - | *120360 |  |
| *MYH3* | Myosin Heavy Chain 3 | 17p13.1 | rs182229640 | 17:10543123 | 0.00359425 | 0.00134001 | 0.00340839 | 0.001146 | 0.004105 | - | - | - | Arthrogryposis, distal, type 2B | *160720 | Smane L, Pilmane M. Evaluation of the presence of MMP-2, TIMP-2, BMP2/4, and TGFβ3 in the facial tissue of children with cleft lip and palate. Acta Med Litu. 2018; 25(2):86-94. |
|  |  |  | rs73976871 | 17:10535100 | 0.0405351 | 0.033248 | 0.0369195 | 0.036 | 0.029557 | - | - | - |  |  |  |
|  |  |  | rs56163389 | 17:10543385 | 0.0617013 | 0.0387177 | 0.0551677 | 0.043 | 0.040230 | - | - | - |  |  |  |
|  |  |  | rs56259391 | 17:10544461 | 0.0617013 | 0.0387055 | 0.0549202 | 0.043 | 0.040230 | - | - | - |  |  |  |
| *MYH9* | Myosin Heavy Chain 9 | 22q12.3 | rs9622375 | 22:36705447 | 0.00998403 | 0.00241756 | 0.00645911 | 0.002693 | 0.003284 | - | - | - | - | *160775 |  |
|  |  |  | rs56020676 | 22:36690120 | 0.0858626 | 0.0305595 | 0.0867383 | 0.035 | 0.067323 | - | - | - |  |  | Martinelli M, Di Stazio M, Scapoli L, Marchesini J, Bari F, Pezzetti F, et al. Cleft lip with or without cleft palate: implication of the heavy chain of non-muscle myosin IIA. J Med Genet 2007;44:387-392. |
|  |  |  | rs9619601 | 22:36700175 | 0.0603035 | 0.0530946 | 0.0515547 | 0.054 | 0.039409 | - | - | - |  |  |  |
| *NOTCH1* | Notch Receptor 1 | 9q34.3 | rs202023246 | 9:139438562 | 0.00279553 | 0.000709927 | 0.00355711 | 0.0007914 | 0.000832 | - | - | - | - | *190198 |  |
|  |  |  | rs61751489 | 9:139391338 | 0.0211661 | 0.0308615 | 0.0119648 | 0.025 | 0.018883 | Tolerated | Benign | Tolerated |  |  |  |
| *NUP188* | Nucleoporin 188 | 9q34.311 | rs146458704 | 9:131768390 | 0.000199681 | 0.0000365456 | 0.000161676 | 0.00004942 | - | Tolerated | Benign | Damaging | Sandestig-Stefanova Syndrome | *615587 |  |
| *PAX1* | Paired Box 1 | 20p11.22 | rs146191505 | 20:21687042 | 0.000599042 | 0.000743541 | 0.000484559 | 0.0005957 | 0.000821 | Tolerated | Benign | Tolerated | Otofaciocervical Syndrome 1 | *167411 |  |
|  |  |  | rs17861031 | 20:21687344 | 0.0712859 | 0.0289695 | 0.0578253 | 0.033 | 0.035304 | - | - | - |  |  |  |
| *PAX6* | Paired Box 6 | 11p13 | rs667773 | 11:31815362 | 0.0904553 | 0.0843132 | 0.0576563 | 0.078 | 0.070608 | - | - | - | Peters-Plus syndrome | *607108 |  |
| *PHYH* | Phytanoyl-CoA 2-Hydroxylase | 10p13 | rs7901902 | 10:13330395 | 0.00559105 | 0.00147481 | 0.00523222 | 0.001845 | 0.006568 | Tolerated | Possibly damaging | Tolerated | - | *602026 |  |
| *PIGG* | Phosphatidylinositol Glycan Anchor Biosynthesis Class G | 4p16.3 | rs116772777 | 4:499568 | 0.00219649 | 0.000483225 | 0.00187375 | 0.0005848 | 0.000821 | Tolerated | Benign | Tolerated | Hyperphosphatasia with Mental Retardation Syndrome 1 | *616918 | Aylward A, Cai Y, Lee A, Blue E, Rabinowitz D, Haddad Jr J, et al. Using Whole Exome Sequencing to Identify Candidate Genes With Rare Variants In Nonsyndromic Cleft Lip and Palate. Genet Epidemiol. 2016;40(5):432-41. |
| *PIGO* | Phosphatidylinositol Glycan Anchor Biosynthesis Class O | 9p13.3 | rs2298314 | 9:35089403 | 0.077476 | 0.0465167 | 0.0391245 | 0.045 | 0.025452 | - | - | - | Hyperphosphatasia with Mental Retardation Syndrome 1, Hyperphosphatasia with Mental Retardation Syndrome 2 | *614730 |  |
| *PIK3R1* | Phosphoinositide-3-Kinase Regulatory Subunit 1 | 5q13.1 | rs73768883 | 5:67522698 | 0.00539137 | 0.00124699 | 0.00458597 | 0.001573 | 0.012315 | - | - | - | - | *171833 |  |
| *POMT1* | Protein O-Mannosyltransferase 1 | 9q34.13 | rs140258585 | 9:134397428 | 0.00279553 | 0.00692278 | 0.00630089 | 0.006984 | 0.001642 | - | - | - | Muscular dystrophy-dystroglycanopathy (congenital with brain and eye anomalies) | *607423 | Wang H, Qiu T, Shi J, Liang J, Wang Y, Quan L, et al. Gene expression profiling analysis contributes to understanding the association between non-syndromic cleft lip and palate, and cancer. Mol Med Rep. 2016;13(3):2110-6. |
|  |  |  | rs11243406 | 9:134390870 | 0.0125799 | 0.0350141 | 0.0404112 | 0.034 | 0.025452 | Damaging | Probably damaging | Damaging |  |  |  |
| *POMT2* | Protein O-Mannosyltransferase 2 | 14q24.3 | rs116434191 | 14:77743797 | 0.00678914 | 0.00152321 | 0.00555915 | 0.002076 | 0.004926 | - | - | - | Muscular dystrophy-dystroglycanopathy (congenital with brain and eye anomalies), type A, 2 | *607439 |  |
| *PTCH1* | Patched 1 | 9q22.32 | rs2236405 | 9:98211572 | 0.0784744 | 0.0335774 | 0.0480918 | 0.024 | 0.036244 | Tolerated | Benign | Damaging | Holoprosencephaly 7, Basal cell nevus syndrome | *601309 |  |
| *PTCH2* | Patched 2 | 1p34.1 | rs112563011 | 1:45293560 | - | - | - | - | 0.003284 | - | - | - | Basal cell nevus syndrome | *603673 | Metzis V, Courtney AD, Kerr MC, Ferguson C, Galeano MCR, Parton RG, et al. Patched1 is required in neural crest cells for the prevention of orofacial clefts. Hum Mol Genet 2013;22:5026-5035. |
| *RET* | Ret Proto-Oncogene | 10q11.21 | rs17158558 | 10:43620335 | 0.0219649 | 0.0192692 | 0.0129141 | 0.019 | 0.024631 | Damaging | Possibly damaging | Damaging | Multiple Endocrine Neoplasia, type lia; Renal Agenesis, Bilateral | *164761 |  |
| *ROR2* | Receptor Tyrosine Kinase Like Orphan Receptor 2 | 9q22.31 | rs2230577 | 9:94486622 | 0.0776757 | 0.0857321 | 0.067948 | 0.083 | 0.071429 | - | - | - | Robinow syndrome, autosomal recessive | *602337 |  |
|  |  |  | rs10992070 | 9:94493470 | 0.0551118 | 0.0867213 | 0.100983 | 0.086 | 0.117406 | - | - | - |  |  | Schwabe GC, Trepczik B, Suring K, Brieske N, Tucker AS, Sharpe PT, et al. Ror2 knockout mouse as a model for the developmental pathology of autosomal recessive Robinow syndrome. Dev Dyn 2004;229:400-410. |
| *RPGRIP1L* | RPGRIP1 Like | 16q12.2 | rs2111119 | 16:53671754 | 0.166134 | 0.111119 | 0.110443 | 0.114 | 0.112479 | Tolerated | Benign | Tolerated | Meckel syndrome, type 5 | *610937 |  |
|  |  |  | rs61743997 | 16:53692693 | 0.0265575 | 0.0217895 | 0.0215818 | 0.022 | 0.027094 | - | - | - |  |  |  |
|  |  |  | rs74393433 | 16:53721906 | 0.100439 | 0.072743 | 0.0571419 | 0.073 | 0.059934 | - | - | - |  |  |  |
| *RPL11* | Ribosomal Protein L11 | 1p36.11 | rs8880 | 1:24021224 | 0.0129792 | 0.0245502 | 0.0253487 | 0.025 | 0.015599 | - | - | - | Diamond-Blackfan anemia 7 | *604175 |  |
| *RPS26* | Ribosomal Protein S26 | 12q13.2 | rs200345325 | 12:56437325 | 0.000599042 | 0.000159398 | 0.000679216 | 0.0002142 | 0.000821 | - | - | - | Diamond-Blackfan anemia 10 | *603701 |  |
|  |  |  | rs56696262 | 12:56437917 | 0.0916534 | 0.0194396 | 0.0743234 | 0.025 | 0.057471 | - | - | - |  |  |  |
| *RPS27* | Ribosomal Protein S27 | 1q21.3 | rs188748675 | 1:153963239 | - | - | - | - | 0.017241 | - | - | - | Diamond-Blackfan anemia, Diamond-Blackfan anemia 17 | *603702 |  |
| *RPS7* | Ribosomal Protein S7 | 2p25.3 | rs62106034 | 2:3624051 | 0.0593051 | 0.0632881 | 0.0560111 | 0.064 | 0.090232 | - | - | - | Diamond-Blackfan anemia 8 | *603658 |  |
| *RYR1* | Ryanodine Receptor 1 | 19q13.2 | rs186460831 | 19:39019355 | 0.00599042 | 0.000964308 | 0.00455898 | 0.001252 | 0.003284 | - | - | - | Multiple Pterygium syndrome, lethal type; Fetal Akinesia Deformation sequence 1 | *180901 |  |
|  |  |  | rs148772854 | 19:39034444 | 0.00599042 | 0.000952055 | 0.00449344 | 0.001244 | 0.003284 | Damaging | Probably damaging | Damaging |  |  |  |
|  |  |  | rs16972636 | 19:38964109 | 0.0539137 | 0.0114555 | 0.0434359 | 0.015 | 0.033662 | - | - | - |  |  |  |
|  |  |  | rs12973632 | 19:38990593 | 0.0233626 | 0.0351607 | 0.0267926 | 0.033 | 0.027094 | - | - | - |  |  |  |
|  |  |  | rs2228072 | 19:38991516 | 0.150759 | 0.109777 | 0.0902085 | 0.114 | 0.078818 | - | - | - |  |  |  |
|  |  |  | rs2071088 | 19:38991543 | 0.135982 | 0.10765 | 0.0809918 | 0.111 | 0.071429 | - | - | - |  |  |  |
|  |  |  | rs77592501 | 19:39018277 | 0.10643 | 0.0790894 | 0.0460152 | 0.08 | 0.032020 | - | - | - |  |  |  |
| *SKI* | SKI Proto-Oncogene | 1p36.33-p36.32 | rs28384811 | 1:2160390 | 0.0457268 | 0.0875834 | 0.0473644 | 0.031 | 0.072250 | Damaging | Possibly damaging | Tolerated | Shprintzen-Goldberg syndrome | *164780 |  |
| *SLC2A10* | Solute Carrier Family 2 Member 10 | 20q13.12 | rs79849424 | 20:45354829 | 0.0289537 | 0.0113924 | 0.0177983 | 0.013 | 0.009852 | Tolerated | Benign | Tolerated | Arterial Tortuosity syndrome | *606145 | Vieira AR, Avila JR, Daack-Hirsch S, et al. Medical sequencing of candidate genes for nonsyndromic cleft lip and palate. PLoS Genet 2005;1:e64. |
|  |  |  | rs41283344 | 20:45358121 | 0.0694888 | 0.014644 | 0.0597801 | 0.019 | 0.045977 | Tolerated | Benign | Damaging |  |  |  |
| *SMARCB1* | SWI/SNF Related, Matrix Associated, Actin Dependent Regulator Of Chromatin, Subfamily B, Member 1 | 22q11.23 | rs143872602 | 22:24145459 | 0.00978434 | 0.00364489 | 0.00319499 | 0.003682 | 0.011494 | - | - | - | Coffin-Siris Syndrome 1 | *601607 |  |
| *SMOC1* | SPARC Related Modular Calcium Binding 1 | 14q24.2 | rs139737624 | 14:70418858 | 0.000399361 | 0.00113735 | 0.000904393 | 0.001186 | 0.001642 | Tolerated | Probably damaging | Damaging | Microphthalmia with limb anomalies | *608488 |  |
| *STXBP1* | Syntaxin Binding Protein 1 | 9q34.11 | rs140247913 | 9:130427627 | 0.0127796 | 0.0168818 | 0.0165773 | 0.017 | 0.009852 | - | - | - | Epileptic encephalopathy, early infantile, 4 | *602926 | Rainger J, van Beusekom E, Ramsay JK, McKie L, Al-Gazali L, Pallotta R, et al. Loss of the BMP antagonist, SMOC-1, causes Ophthalmo-acromelic (Waardenburg Anophthalmia) syndrome in humans and mice. PLoS Genet 2011;7:e1002114. |
|  |  |  | rs58889246 | 9:130430410 | 0.0519169 | 0.0119293 | 0.0470447 | 0.015 | 0.026273 | - | - | - |  |  |  |
| *TBC1D32* | TBC1 Domain Family Member 32 | 6q22.31 | rs7767455 | 6:121642851 | 0.196286 | 0.0928858 | 0.155896 | 0.099 | 0.147204 | Tolerated | Benign | Tolerated | Oro-Facio-Digital syndrome type IX | *615867 |  |
| *TCN2* | Transcobalamin 2 | 22q12.2 | rs2283873 | 22:31013296 | 0.167133 | 0.0871466 | 0.1164 | 0.087 | 0.100985 | - | - | - | - | *613441 |  |
|  |  |  | rs9621049 | 22:31013419 | 0.108626 | 0.114338 | 0.127767 | 0.112 | 0.148604 | Tolerated | Benign | Tolerated |  |  | Martinelli M, Scapoli L, Palmieri A, Pezzetti F, Baciliero U, Padula E, et al. Study of four genes belonging to the folate pathway: transcobalamin 2 is involved in the onset of non-syndromic cleft lip with or without cleft palate. Hum Mutat 2006;27:294. |
| *TCTN2* | Tectonic Family Member 2 | 12q24.31 | rs145374149 | 12:124171486 | 0.000599042 | 0.000718723 | 0.000742414 | 0.0006919 | 0.002463 | Tolerated | Benign | Tolerated | Meckel syndrome 8, Joubert syndrome 24 | *613846 |  |
| *TDGF1* | Teratocarcinoma-Derived Growth Factor 1 | 3p21.31 | rs2293025 | 3:46620760 | 0.0778754 | 0.0571158 | 0.0314236 | 0.058 | 0.030378 | Tolerated | Benign | Tolerated | Holoprosencephaly | +187395 | Sang L, Miller JJ, Corbit KC, Giles RH, Brauer MJ, Otto EA, et al. Mapping the NPHP-JBTS-MKS protein network reveals ciliopathy disease genes and pathways. Cell 2011;145:513-528. |
| *TGFB1* | Transforming Growth Factor Beta 1 | 19q13.2 | rs1800472 | 19:41847860 | 0.0107827 | 0.0259585 | 0.022309 | 0.025 | 0.031199 | Tolerated | Benign | Damaging | Diamond-Blackfan Anemia | *190180 |  |
| *TMCO1* | Transmembrane And Coiled-Coil Domains 1 | 1q24.1 | rs553722607 | 1:165694095 | - | - | - | - | - | - | - | - | Craniofacial dysmorphism, skeletal anomalies, and mental retardation syndrome | *614123 |  |
|  |  |  | c.*3075C>T | 1:165694185 | - | - | - | - | - | - | - | - |  |  |  |
|  |  |  | rs78363884 | 1:165697341 | 0.0205671 | 0.0340281 | 0.0279589 | 0.032 | 0.023810 | - | - | - |  |  |  |
| *TMEM237* | Transmembrane Protein 237 | 2q33.1 | rs2241133 | 2:202507353 | 0.111422 | 0.0472663 | 0.0812173 | 0.053 | 0.058292 | - | - | - | Joubert syndrome 1, Joubert syndrome 4, Joubert syndrome 14 | *614423 |  |
| *TNFRSF1A* | TNF Receptor Superfamily Member 1A | 12p13.31 | rs147111217 | 12:6443251 | 0.000599042 | 0.000155224 | 0.000355504 | 0.0001812 | - | - | - | - | Charge syndrome | *191190 |  |
| *TSC1* | TSC Complex Subunit 1 | 9q34.13 | rs4962081 | 9:135772717 | 0.0621006 | 0.0794689 | 0.076269 | 0.076 | 0.088670 | - | - | - | Congenital Heart Defects, Hamartomas of Tongue, and Polysyndactyly | *605284 |  |
| *TTC21B* | Tetratricopeptide Repeat Domain 21B | 2q24.3 | rs34486024 | 2:166806142 | - | - | - | - | 0.007389 | - | - | - | Short-rib thoracic dysplasia 4 with or without polydactyly | *612014 |  |
|  |  |  | rs115504901 | 2:166740469 | 0.0167732 | 0.0316246 | 0.0365539 | 0.031 | 0.036789 | - | - | - |  |  |  |
| *WDPCP* | WD Repeat Containing Planar Cell Polarity Effector | 2p15 | rs141340867 | 2:63815338 | - | - | - | - | 0.004132 | - | - | - | Meckel Syndrome, Type 1 | *613580 |  |
| *WDR35* | WD Repeat Domain 35 | 2p24.1 | rs34169020 | 2:20175312 | 0.0636981 | 0.0436054 | 0.0389636 | 0.042 | 0.027094 | - | - | - | Short-rib thoracic dysplasia 7 with or without polydactyly | *613602 |  |
|  |  |  | rs1060742 | 2:20189015 | 0.11861 | 0.107749 | 0.0728857 | 0.107 | 0.093596 | Tolerated | Benign | Tolerated |  |  |  |
| *WDR60* | WD Repeat Domain 60 | 7q36.3 | rs151184533 | 7:158718971 | 0.000798722 | 0.000214555 | 0.000871474 | 0.0002483 | - | Tolerated | Possibly damaging | Damaging | Short-rib thoracic dysplasia 8 with or without polydactyly | *615462 |  |

An asterisk (*) before an OMIM entry number indicates a gene.

A plus sign (+) before an OMIM entry number indicates that the entry includes a description of a gene and a phenotype.
