# Supplementary material for: Identification of Novel Variants in Cleft Palate-Associated Genes in Brazilian Patients With Non-syndromic Cleft Palate Only
Source: Front Cell Dev Biol. 2021 Jul 8;9:638522. doi: 10.3389/fcell.2021.638522 (PMC8297955; doi:10.3389/fcell.2021.638522)
Supplement: Supplementary file 6 [file Data_Sheet_4.docx]

|  | | | | | | |  |  |  |  |  |
| --- | --- | --- | --- | --- | --- | --- | --- | --- | --- | --- | --- |
| **Supplementary Table 4.** List of variants identified in patients with nonsyndromic cleft palate only (NSCPO), but not associated with genes related to palatogenesis or oral cleft. | | | | | | | | | | | |
| **Gene** | **Protein** | **Variants** | **Chr:Pos** | **MAF 1K** | **MAF gnomAD Exomes** | **MAF gnomAD Genomes** | **MAF ExAC** | **Sequence Ontology** | **SIFT** | **Polyphen2** | **MutationTaster** |
| *A2M* | Alpha-2-Macroglobulin | rs190813517 | 12:9230025 | 0.000599042 | 0.00153586 | 0.0013242 | 0.001508 | intron_variant | - | - | - |
| *A2ML1* | Alpha-2-Macroglobulin Like 1 | [rs61749073](http://www.ncbi.nlm.nih.gov/projects/SNP/snp_ref.cgi?rs=rs61749073) | [12:9020563](genomebrowse://api/zoom?locus=12:9020563) | 0.0730831 | 0.0689 | 0.112917 | 0.071 | synonymous_variant | - | - | - |
| *A2ML1* | Alpha-2-Macroglobulin Like 1 | [rs77655847](http://www.ncbi.nlm.nih.gov/projects/SNP/snp_ref.cgi?rs=rs77655847) | [12:9020794](genomebrowse://api/zoom?locus=12:9020794) | 0.0730831 | 0.0689321 | 0.112809 | 0.071 | intron_variant | - | - | - |
| *A2ML1* | Alpha-2-Macroglobulin Like 1 | [rs79655903](http://www.ncbi.nlm.nih.gov/projects/SNP/snp_ref.cgi?rs=rs79655903) | [12:9020796](genomebrowse://api/zoom?locus=12:9020796) | 0.0730831 | 0.0689526 | 0.112817 | 0.071 | intron_variant | - | - | - |
| *AAAS* | Aladin WD Repeat Nucleoporin | rs202149640 | 12:53702334 | 0.000399361 | 0.000300735 | 0.000258498 | 0.000173 | intron_variant | - | - | - |
| *AACS* | Acetoacetyl-CoA Synthetase | [rs3751180](http://www.ncbi.nlm.nih.gov/projects/SNP/snp_ref.cgi?rs=rs3751180) | [12:125626685](genomebrowse://api/zoom?locus=12:125626685) | 0.0948482 | 0.0486011 | 0.0587266 | 0.048 | synonymous_variant | - | - | - |
| *AARS1* | Alanyl-TRNA Synthetase 1 | [rs7190921](http://www.ncbi.nlm.nih.gov/projects/SNP/snp_ref.cgi?rs=rs7190921) | [16:70299401](genomebrowse://api/zoom?locus=16:70299401) | 0.0826677 | 0.0171872 | 0.0705286 | 0.022 | intron_variant | - | - | - |
| *AARS1* | Alanyl-TRNA Synthetase 1 | [rs7192000](http://www.ncbi.nlm.nih.gov/projects/SNP/snp_ref.cgi?rs=rs7192000) | [16:70299420](genomebrowse://api/zoom?locus=16:70299420) | 0.108826 | 0.0231099 | 0.0915352 | 0.029 | intron_variant | - | - | - |
| *AARS1* | Alanyl-TRNA Synthetase 1 | [rs11537667](http://www.ncbi.nlm.nih.gov/projects/SNP/snp_ref.cgi?rs=rs11537667) | [16:70303659](genomebrowse://api/zoom?locus=16:70303659) | 0.0834665 | 0.0172211 | 0.0707361 | 0.022 | missense_variant | Damaging | Probably damaging | Damaging |
| *AARS1* | Alanyl-TRNA Synthetase 1 | [rs74024185](http://www.ncbi.nlm.nih.gov/projects/SNP/snp_ref.cgi?rs=rs74024185) | [16:70305681](genomebrowse://api/zoom?locus=16:70305681) | 0.0836661 | 0.0174391 | 0.0708447 | 0.022 | splice_region_variant | - | - | - |
| *AARS2* | Alanyl-TRNA Synthetase 2, Mitochondrial | rs191792373 | 6:44269749 | 0.000199681 | 5.30851e-05 | 3.23018e-05 | 4.119e-05 | intron_variant | - | - | - |
| *AASDH* | Aminoadipate-Semialdehyde Dehydrogenase | [rs61978622](http://www.ncbi.nlm.nih.gov/projects/SNP/snp_ref.cgi?rs=rs61978622) | [4:57215969](genomebrowse://api/zoom?locus=4:57215969) | 0.0185703 | 0.0250415 | 0.0181801 | 0.024 | missense_variant | Tolerated | Benign | Tolerated |
| *AASS* | Aminoadipate-Semialdehyde Synthase | rs73442839 | 7:121741662 | 0.00259585 | 0.00103673 | 0.00405003 | 0.001286 | intron_variant | - | - | - |
| *ABAT* | 4-Aminobutyrate Aminotransferase | rs41312254 | 16:8860130 | 0.00998403 | 0.00791122 | 0.00729362 | 0.007726 | splice_region_variant | - | - | - |
| *ABCA1* | ATP Binding Cassette Subfamily A Member 1 | rs34590907 | 9:107594088 | 0.00399361 | 0.000898133 | 0.00377712 | 0.001054 | synonymous_variant | - | - | - |
| *ABCA1* | ATP Binding Cassette Subfamily A Member 1 | rs199728899 | 9:107645290 | 0.000399361 | 0.00019088 | 0.000645703 | 0.0001894 | intron_variant | - | - | - |
| *ABCA1* | ATP Binding Cassette Subfamily A Member 1 | [rs9282537](http://www.ncbi.nlm.nih.gov/projects/SNP/snp_ref.cgi?rs=rs9282537) | [9:107550222](genomebrowse://api/zoom?locus=9:107550222) | 0.0764776 | 0.0321898 | 0.0738574 | 0.036 | synonymous_variant | - | - | - |
| *ABCA1* | ATP Binding Cassette Subfamily A Member 1 | [rs2230807](http://www.ncbi.nlm.nih.gov/projects/SNP/snp_ref.cgi?rs=rs2230807) | [9:107578478](genomebrowse://api/zoom?locus=9:107578478) | 0.063099 | 0.0141716 | 0.0532885 | 0.018 | synonymous_variant | - | - | - |
| *ABCA1* | ATP Binding Cassette Subfamily A Member 1 | [rs34788556](http://www.ncbi.nlm.nih.gov/projects/SNP/snp_ref.cgi?rs=rs34788556) | [9:107578529](genomebrowse://api/zoom?locus=9:107578529) | 0.0355431 | 0.0137613 | 0.0356127 | 0.016 | synonymous_variant | - | - | - |
| *ABCA1* | ATP Binding Cassette Subfamily A Member 1 | [rs33918808](http://www.ncbi.nlm.nih.gov/projects/SNP/snp_ref.cgi?rs=rs33918808) | [9:107579632](genomebrowse://api/zoom?locus=9:107579632) | 0.0567093 | 0.0344409 | 0.0664557 | 0.038 | missense_variant | Tolerated | Benign | Damaging |
| *ABCA1* | ATP Binding Cassette Subfamily A Member 1 | [rs12003906](http://www.ncbi.nlm.nih.gov/projects/SNP/snp_ref.cgi?rs=rs12003906) | [9:107645477](genomebrowse://api/zoom?locus=9:107645477) | 0.0664936 | 0.0217708 | 0.0609969 | 0.025 | intron_variant | - | - | - |
| *ABCA1* | ATP Binding Cassette Subfamily A Member 1 | [rs1800978](http://www.ncbi.nlm.nih.gov/projects/SNP/snp_ref.cgi?rs=rs1800978) | [9:107665978](genomebrowse://api/zoom?locus=9:107665978) | 0.141773 | 0.144631 | 0.097824 | 0.105 | 5_prime_UTR_variant | - | - | - |
| *ABCA11P* | ATP Binding Cassette Subfamily A Member 11, Pseudogene | [rs72501950](http://www.ncbi.nlm.nih.gov/projects/SNP/snp_ref.cgi?rs=rs72501950) | [4:437831](genomebrowse://api/zoom?locus=4:437831) | 0.163339 | 0.106185 | 0.139445 | 0.111 | missense_variant | Damaging | Benign | Damaging |
| *ABCA12* | ATP Binding Cassette Subfamily A Member 12 | c.2518C>G | [2:215872525](genomebrowse://api/zoom?locus=2:215872525) | - | - | - | - | missense_variant | Tolerated | Benign | Tolerated |
| *ABCA12* | ATP Binding Cassette Subfamily A Member 12 | [rs16853238](http://www.ncbi.nlm.nih.gov/projects/SNP/snp_ref.cgi?rs=rs16853238) | [2:215914446](genomebrowse://api/zoom?locus=2:215914446) | 0.0702875 | 0.014983 | 0.0585877 | 0.02 | missense_variant | Damaging | Benign | Damaging |
| *ABCA12* | ATP Binding Cassette Subfamily A Member 12 | [rs34273324](http://www.ncbi.nlm.nih.gov/projects/SNP/snp_ref.cgi?rs=rs34273324) | [2:215914491](genomebrowse://api/zoom?locus=2:215914491) | 0.0714856 | 0.0149823 | 0.0586486 | 0.02 | synonymous_variant | - | - | - |
| *ABCA12* | ATP Binding Cassette Subfamily A Member 12 | [rs16853246](http://www.ncbi.nlm.nih.gov/projects/SNP/snp_ref.cgi?rs=rs16853246) | [2:215917181](genomebrowse://api/zoom?locus=2:215917181) | 0.0702875 | 0.0149656 | 0.0587798 | 0.02 | intron_variant | - | - | - |
| *ABCA13* | ATP Binding Cassette Subfamily A Member 13 | rs186526946 | 7:48318613 | 0.00958466 | 0.00811018 | 0.00274441 | 0.006418 | missense_variant | Tolerated | Benign | Tolerated |
| *ABCA13* | ATP Binding Cassette Subfamily A Member 13 | [rs17661364](http://www.ncbi.nlm.nih.gov/projects/SNP/snp_ref.cgi?rs=rs17661364) | [7:48315360](genomebrowse://api/zoom?locus=7:48315360) | 0.0463259 | 0.0388833 | 0.0279465 | 0.036 | missense_variant | Damaging | Benign | Tolerated |
| *ABCA13* | ATP Binding Cassette Subfamily A Member 13 | [rs17132289](http://www.ncbi.nlm.nih.gov/projects/SNP/snp_ref.cgi?rs=rs17132289) | [7:48428715](genomebrowse://api/zoom?locus=7:48428715) | 0.0856629 | 0.084711 | 0.0968117 | 0.081 | missense_variant | Tolerated | Possibly damaging | Tolerated |
| *ABCA13* | ATP Binding Cassette Subfamily A Member 13 | [rs12669976](http://www.ncbi.nlm.nih.gov/projects/SNP/snp_ref.cgi?rs=rs12669976) | [7:48546011](genomebrowse://api/zoom?locus=7:48546011) | 0.157348 | 0.110294 | 0.138242 | 0.109 | intron_variant | - | - | - |
| *ABCA3* | ATP Binding Cassette Subfamily A Member 3 | rs148662935 | 16:2376463 | 0.000998403 | 0.000192456 | 0.000807389 | 0.0002554 | missense_variant | Damaging | Probably damaging | Damaging |
| *ABCA3* | ATP Binding Cassette Subfamily A Member 3 | [rs45538638](http://www.ncbi.nlm.nih.gov/projects/SNP/snp_ref.cgi?rs=rs45538638) | [16:2345758](genomebrowse://api/zoom?locus=16:2345758) | 0.0113818 | 0.0208247 | 0.0170017 | 0.021 | intron_variant | - | - | - |
| *ABCA5* | ATP Binding Cassette Subfamily A Member 5 | rs145300105 | 17:67266828 | 0.000399361 | 0.00106835 | 0.00103426 | 0.001096 | missense_variant | Tolerated | Benign | Tolerated |
| *ABCA5* | ATP Binding Cassette Subfamily A Member 5 | [rs17686569](http://www.ncbi.nlm.nih.gov/projects/SNP/snp_ref.cgi?rs=rs17686569) | [17:67290840](genomebrowse://api/zoom?locus=17:67290840) | 0.14377 | 0.133701 | 0.156005 | 0.135 | missense_variant | Tolerated | Benign | Damaging |
| *ABCA6* | ATP Binding Cassette Subfamily A Member 6 | [rs9282554](http://www.ncbi.nlm.nih.gov/projects/SNP/snp_ref.cgi?rs=rs9282554) | [17:67109833](genomebrowse://api/zoom?locus=17:67109833) | 0.0934505 | 0.0834237 | 0.0711607 | 0.077 | missense_variant | Tolerated | Benign | Tolerated |
| *ABCA8* | ATP Binding Cassette Subfamily A Member 8 | rs148753714 | 17:66877343 | 0.000199681 | 4.87777e-05 | 0.00016152 | 8.237e-05 | missense_variant | Damaging | Probably damaging | Damaging |
| *ABCB4* | ATP Binding Cassette Subfamily B Member 4 | rs45575636 | 7:87060844 | 0.00439297 | 0.00449091 | 0.00510171 | 0.004176 | missense_variant | Damaging | Probably damaging | Damaging |
| *ABCB5* | ATP Binding Cassette Subfamily B Member 5 | [rs17143212](http://www.ncbi.nlm.nih.gov/projects/SNP/snp_ref.cgi?rs=rs17143212) | [7:20682884](genomebrowse://api/zoom?locus=7:20682884) | 0.0704872 | 0.038936 | 0.0405039 | 0.042 | missense_variant | Damaging | Possibly damaging | Tolerated |
| *ABCB6* | ATP Binding Cassette Subfamily B Member 6 (Langereis Blood Group) | rs61733629 | 2:220079136 | 0.00579073 | 0.00612767 | 0.00106589 | 0.004579 | missense_variant | Damaging | Probably damaging | Damaging |
| *ABCB7* | ATP Binding Cassette Subfamily B Member 7 | [rs73502896](http://www.ncbi.nlm.nih.gov/projects/SNP/snp_ref.cgi?rs=rs73502896) | [X:74280041](genomebrowse://api/zoom?locus=X:74280041) | 0.0839735 | 0.0229326 | 0.0792691 | 0.026 | intron_variant | - | - | - |
| *ABCB7* | ATP Binding Cassette Subfamily B Member 7 | [rs1340990](http://www.ncbi.nlm.nih.gov/projects/SNP/snp_ref.cgi?rs=rs1340990) | [X:74284996](genomebrowse://api/zoom?locus=X:74284996) | 0.117881 | 0.0307243 | 0.104712 | 0.035 | synonymous_variant | - | - | - |
| *ABCB7* | ATP Binding Cassette Subfamily B Member 7 | [rs1340989](http://www.ncbi.nlm.nih.gov/projects/SNP/snp_ref.cgi?rs=rs1340989) | [X:74284997](genomebrowse://api/zoom?locus=X:74284997) | 0.0837086 | 0.0226188 | 0.0776568 | 0.026 | missense_variant | Tolerated | Benign | Tolerated |
| *ABCB8* | ATP Binding Cassette Subfamily B Member 8 | [rs76225344](http://www.ncbi.nlm.nih.gov/projects/SNP/snp_ref.cgi?rs=rs76225344) | [7:150733115](genomebrowse://api/zoom?locus=7:150733115) | 0.0371406 | 0.0434528 | 0.0512829 | 0.045 | intron_variant | - | - | - |
| *ABCC1* | ATP Binding Cassette Subfamily C Member 1 | [rs8187858](http://www.ncbi.nlm.nih.gov/projects/SNP/snp_ref.cgi?rs=rs8187858) | [16:16162039](genomebrowse://api/zoom?locus=16:16162039) | 0.0357428 | 0.0665101 | 0.0630773 | 0.066 | synonymous_variant | - | - | - |
| *ABCC11* | ATP Binding Cassette Subfamily C Member 11 | rs201289660 | 16:48247328 | 0.000399361 | 0.000571043 | 0.000258248 | 0.0003871 | intron_variant | - | - | - |
| *ABCC11* | ATP Binding Cassette Subfamily C Member 11 | [rs61739606](http://www.ncbi.nlm.nih.gov/projects/SNP/snp_ref.cgi?rs=rs61739606) | [16:48204078](genomebrowse://api/zoom?locus=16:48204078) | 0.0509185 | 0.0765143 | 0.0812548 | 0.076 | missense_variant | Damaging | Probably damaging | Damaging |
| *ABCC13* | ATP Binding Cassette Subfamily C Member 13 (Pseudogene) | [rs2822558](http://www.ncbi.nlm.nih.gov/projects/SNP/snp_ref.cgi?rs=rs2822558) | [21:15671844](genomebrowse://api/zoom?locus=21:15671844) | 0.0786741 | 0.121954 | 0.123497 | 0.115 | non_coding_exon_variant | - | - | - |
| *ABCC2* | ATP Binding Cassette Subfamily C Member 2 | rs145672804 | 10:101591866 | 0.000599042 | 0.000609142 | 0.000452021 | 0.0006424 | missense_variant | Damaging | Possibly damaging | Damaging |
| *ABCC2* | ATP Binding Cassette Subfamily C Member 2 | [rs17216177](http://www.ncbi.nlm.nih.gov/projects/SNP/snp_ref.cgi?rs=rs17216177) | [10:101603522](genomebrowse://api/zoom?locus=10:101603522) | 0.0722843 | 0.0532575 | 0.0813202 | 0.054 | intron_variant | - | - | - |
| *ABCC3* | ATP Binding Cassette Subfamily C Member 3 | rs35669870 | 17:48735837 | 0.00459265 | 0.0008771 | 0.00297254 | 0.001244 | synonymous_variant | - | - | - |
| *ABCC3* | ATP Binding Cassette Subfamily C Member 3 | [rs4148416](http://www.ncbi.nlm.nih.gov/projects/SNP/snp_ref.cgi?rs=rs4148416) | [17:48753423](genomebrowse://api/zoom?locus=17:48753423) | 0.136981 | 0.0865431 | 0.0995985 | 0.086 | synonymous_variant | - | - | - |
| *ABCC6* | ATP Binding Cassette Subfamily C Member 6 | rs61480102 | 16:16282841 | 0.00419329 | 0.00084126 | 0.00332881 | 0.001063 | intron_variant | - | - | - |
| *ABCC6* | ATP Binding Cassette Subfamily C Member 6 | [rs3902401](http://www.ncbi.nlm.nih.gov/projects/SNP/snp_ref.cgi?rs=rs3902401) | [16:16243973](genomebrowse://api/zoom?locus=16:16243973) | 0.0662939 | 0.0665227 | 0.0514654 | 0.067 | 3_prime_UTR_variant | - | - | - |
| *ABCC8* | ATP Binding Cassette Subfamily C Member 8 | [rs8192690](http://www.ncbi.nlm.nih.gov/projects/SNP/snp_ref.cgi?rs=rs8192690) | [11:17414570](genomebrowse://api/zoom?locus=11:17414570) | 0.0311502 | 0.0549128 | 0.0466326 | 0.056 | missense_variant | Tolerated | Benign | Damaging |
| *ABCD2* | ATP Binding Cassette Subfamily D Member 2 | c.1121-31C>A | [12:40001547](genomebrowse://api/zoom?locus=12:40001547) | - | - | - | - | intron_variant | - | - | - |
| *ABCD3* | ATP Binding Cassette Subfamily D Member 3 | [rs4148058](http://www.ncbi.nlm.nih.gov/projects/SNP/snp_ref.cgi?rs=rs4148058) | [1:94883995](genomebrowse://api/zoom?locus=1:94883995) | 0.0527157 | 0.0477367 | 0.0356749 | 0.038 | 5_prime_UTR_variant | - | - | - |
| *ABCD4* | ATP Binding Cassette Subfamily D Member 4 | [rs1052000](http://www.ncbi.nlm.nih.gov/projects/SNP/snp_ref.cgi?rs=rs1052000) | [14:74759529](genomebrowse://api/zoom?locus=14:74759529) | 0.0726837 | 0.0160347 | 0.0638738 | 0.021 | synonymous_variant | - | - | - |
| *ABCD4* | ATP Binding Cassette Subfamily D Member 4 | [rs34992370](http://www.ncbi.nlm.nih.gov/projects/SNP/snp_ref.cgi?rs=rs34992370) | [14:74763064](genomebrowse://api/zoom?locus=14:74763064) | 0.0726837 | 0.0160542 | 0.0639399 | 0.021 | missense_variant | Tolerated | Benign | Tolerated |
| *ABCD4* | ATP Binding Cassette Subfamily D Member 4 | [rs36031534](http://www.ncbi.nlm.nih.gov/projects/SNP/snp_ref.cgi?rs=rs36031534) | [14:74763086](genomebrowse://api/zoom?locus=14:74763086) | 0.0726837 | 0.0160296 | 0.0639565 | 0.021 | synonymous_variant | - | - | - |
| *ABCG2* | ATP Binding Cassette Subfamily G Member 2 (Junior Blood Group) | [rs2231137](http://www.ncbi.nlm.nih.gov/projects/SNP/snp_ref.cgi?rs=rs2231137) | [4:89061114](genomebrowse://api/zoom?locus=4:89061114) | 0.157548 | 0.113843 | 0.0791207 | 0.106 | missense_variant | Tolerated | Benign | Tolerated |
| *ABCG5* | ATP Binding Cassette Subfamily G Member 5 | rs146801512 | 2:44051125 | 0.000599042 | 0.000743105 | 0.000226098 | 0.0006424 | synonymous_variant | - | - | - |
| *ABCG8* | ATP Binding Cassette Subfamily G Member 8 | rs72647316 | 2:44066243 | 0.00898562 | 0.00164205 | 0.00792419 | 0.002083 | synonymous_variant | - | - | - |
| *ABCG8* | ATP Binding Cassette Subfamily G Member 8 | rs142250628 | 2:44071736 | 0.00738818 | 0.0015072 | 0.00568182 | 0.00196 | missense_variant | Tolerated | Probably damaging | Damaging |
| *ABCG8* | ATP Binding Cassette Subfamily G Member 8 | [rs56132765](http://www.ncbi.nlm.nih.gov/projects/SNP/snp_ref.cgi?rs=rs56132765) | [2:44078853](genomebrowse://api/zoom?locus=2:44078853) | 0.063099 | 0.0660922 | 0.0678306 | 0.064 | synonymous_variant | - | - | - |
| *ABCG8* | ATP Binding Cassette Subfamily G Member 8 | [rs3841852](http://www.ncbi.nlm.nih.gov/projects/SNP/snp_ref.cgi?rs=rs3841852) | [2:44101650](genomebrowse://api/zoom?locus=2:44101650) | 0.296326 | - | 0.165502 | 0.19 | intron_variant | - | - | - |
| *ABCG8* | ATP Binding Cassette Subfamily G Member 8 | [rs3841852](http://www.ncbi.nlm.nih.gov/projects/SNP/snp_ref.cgi?rs=rs3841852) | [2:44101650](genomebrowse://api/zoom?locus=2:44101650) | 0.315695,0.296326 | - | 0.373768,0.165502 | 0.403,0.19 | intron_variant | - | - | - |
| *ABHD12* | Abhydrolase Domain Containing 12 | [rs746748](http://www.ncbi.nlm.nih.gov/projects/SNP/snp_ref.cgi?rs=rs746748) | [20:25282967](genomebrowse://api/zoom?locus=20:25282967) | 0.0183706 | 0.0415874 | 0.0365893 | 0.043 | missense_variant | Tolerated | Possibly damaging | Damaging |
| *ABHD14A-ACY1* | ABHD14A-ACY1 Readthrough | [rs323894](http://www.ncbi.nlm.nih.gov/projects/SNP/snp_ref.cgi?rs=rs323894) | [3:52021092](genomebrowse://api/zoom?locus=3:52021092) | 0.103235 | 0.0391147 | 0.09007 | 0.045 | intron_variant | - | - | - |
| *ABHD14A-ACY1* | ABHD14A-ACY1 Readthrough | [rs323895](http://www.ncbi.nlm.nih.gov/projects/SNP/snp_ref.cgi?rs=rs323895) | [3:52021316](genomebrowse://api/zoom?locus=3:52021316) | 0.128195 | 0.050399 | 0.107002 | 0.057 | intron_variant | - | - | - |
| *ABHD14A-ACY2* | - | [rs404527](http://www.ncbi.nlm.nih.gov/projects/SNP/snp_ref.cgi?rs=rs404527) | [3:52012000](genomebrowse://api/zoom?locus=3:52012000) | 0.0790735 | 0.0201562 | 0.0756919 | 0.025 | missense_variant | Tolerated | Benign | Tolerated |
| *ABHD5* | Abhydrolase Domain Containing 5 | [rs887472](http://www.ncbi.nlm.nih.gov/projects/SNP/snp_ref.cgi?rs=rs887472) | [3:43760062](genomebrowse://api/zoom?locus=3:43760062) | 0.103435 | 0.0542462 | 0.0559793 | 0.056 | 3_prime_UTR_variant | - | - | - |
| *ABI3BP* | ABI Family Member 3 Binding Protein | [rs36077176](http://www.ncbi.nlm.nih.gov/projects/SNP/snp_ref.cgi?rs=rs36077176) | [3:100548484](genomebrowse://api/zoom?locus=3:100548484) | 0.115615 | 0.164906 | 0.145464 | 0.186 | intron_variant | Tolerated | Benign | Tolerated |
| *ABLIM2* | Actin Binding LIM Protein Family Member 2 | rs79014382 | 4:8055907 | 0.000798722 | 0.00018275 | 0.000839414 | 0.0002562 | splice_region_variant | - | - | - |
| *ABO* | ABO, Alpha 1-3-N-Acetylgalactosaminyltransferase And Alpha 1-3-Galactosyltransferase | [rs8176744](http://www.ncbi.nlm.nih.gov/projects/SNP/snp_ref.cgi?rs=rs8176744) | [9:136131350](genomebrowse://api/zoom?locus=9:136131350) | 0.013778 | 0.0302774 | 0.0358467 | 0.028 | missense_variant | - | - | - |
| *ABO* | ABO, Alpha 1-3-N-Acetylgalactosaminyltransferase And Alpha 1-3-Galactosyltransferase | [rs55727303](http://www.ncbi.nlm.nih.gov/projects/SNP/snp_ref.cgi?rs=rs55727303) | [9:136131576](genomebrowse://api/zoom?locus=9:136131576) | 0.0225639 | 0.0336555 | 0.0150159 | 0.028 | missense_variant | - | - | - |
| *ABO* | ABO, Alpha 1-3-N-Acetylgalactosaminyltransferase And Alpha 1-3-Galactosyltransferase | [rs1053878](http://www.ncbi.nlm.nih.gov/projects/SNP/snp_ref.cgi?rs=rs1053878) | [9:136131651](genomebrowse://api/zoom?locus=9:136131651) | 0.132788 | 0.0833534 | 0.119332 | 0.087 | missense_variant | - | Benign | - |
| *ABTB2* | Ankyrin Repeat And BTB Domain Containing 2 | rs116088554 | 11:34175854 | 0.00399361 | 0.000744362 | 0.00323081 | 0.000939 | synonymous_variant | - | - | - |
| SCN9A | Sodium Voltage-Gated Channel Alpha Subunit 9 | [rs3750904](http://www.ncbi.nlm.nih.gov/projects/SNP/snp_ref.cgi?rs=rs3750904) | [2:167055393](genomebrowse://api/zoom?locus=2:167055393) | 0.0654952 | 0.0613848 | 0.0194382 | 0.049 | missense_variant | Damaging | Benign | Damaging |
| SCN9A | Sodium Voltage-Gated Channel Alpha Subunit 9 | [rs149207258](http://www.ncbi.nlm.nih.gov/projects/SNP/snp_ref.cgi?rs=rs149207258) | [2:167056337](genomebrowse://api/zoom?locus=2:167056337) | 0.0319489 | 0.0616963 | 0.0687253 | 0.062 | synonymous_variant | - | - | - |
| GTDC1 | Glycosyltransferase Like Domain Containing 1 | [rs56385884](http://www.ncbi.nlm.nih.gov/projects/SNP/snp_ref.cgi?rs=rs56385884) | [2:144710295](genomebrowse://api/zoom?locus=2:144710295) | 0.119609 | 0.101779 | 0.111348 | 0.104 | intron_variant | - | - | - |
| COL4A3 | Collagen Type IV Alpha 3 Chain | rs147886850 | 2:228162381 | 0.000998403 | 0.00123244 | 0.00119432 | 0.000895 | intron_variant | - | - | - |
| COL4A3 | Collagen Type IV Alpha 3 Chain | c.3419-19_3419-18delTT | [2:228159661](genomebrowse://api/zoom?locus=2:228159661) | 0.228035 | 0.168999 | 0.180676 | 0.204 | intron_variant | - | - | - |
| COL4A3 | Collagen Type IV Alpha 3 Chain | [rs116133488](http://www.ncbi.nlm.nih.gov/projects/SNP/snp_ref.cgi?rs=rs116133488) | [2:228159666](genomebrowse://api/zoom?locus=2:228159666) | 0.0389377 | 0.0445315 | 0.061977 | 0.044 | intron_variant | - | - | - |
| COL4A3 | Collagen Type IV Alpha 3 Chain | [rs57611801](http://www.ncbi.nlm.nih.gov/projects/SNP/snp_ref.cgi?rs=rs57611801) | [2:228163453](genomebrowse://api/zoom?locus=2:228163453) | 0.038738 | 0.0450209 | 0.0631865 | 0.044 | missense_variant | Tolerated | Benign | Damaging |
| COL4A3 | Collagen Type IV Alpha 3 Chain | [rs73993953](http://www.ncbi.nlm.nih.gov/projects/SNP/snp_ref.cgi?rs=rs73993953) | [2:228172375](genomebrowse://api/zoom?locus=2:228172375) | 0.0389377 | 0.0453801 | 0.0634167 | 0.044 | intron_variant | - | - | - |
| *ACAA1* | Acetyl-CoA Acyltransferase 1 | [rs61095322](http://www.ncbi.nlm.nih.gov/projects/SNP/snp_ref.cgi?rs=rs61095322) | [3:38168125](genomebrowse://api/zoom?locus=3:38168125) | 0.0467252 | 0.0465114 | 0.0627708 | 0.047 | missense_variant | Tolerated | Benign | Damaging |
| *ACACA* | Acetyl-CoA Carboxylase Alpha | [rs6607364](http://www.ncbi.nlm.nih.gov/projects/SNP/snp_ref.cgi?rs=rs6607364) | [17:35538304](genomebrowse://api/zoom?locus=17:35538304) | 0.0373403 | 0.0202154 | 0.0366122 | 0.02 | splice_region_variant | - | - | - |
| *ACACA* | Acetyl-CoA Carboxylase Alpha | [rs77260549](http://www.ncbi.nlm.nih.gov/projects/SNP/snp_ref.cgi?rs=rs77260549) | [17:35600518](genomebrowse://api/zoom?locus=17:35600518) | 0.0303514 | 0.0101404 | 0.0226671 | 0.011 | intron_variant | - | - | - |
| *ACAD11* | Acyl-CoA Dehydrogenase Family Member 11 | [rs61748105](http://www.ncbi.nlm.nih.gov/projects/SNP/snp_ref.cgi?rs=rs61748105) | [3:132319361](genomebrowse://api/zoom?locus=3:132319361) | 0.034345 | 0.0535691 | 0.050155 | 0.053 | synonymous_variant | - | - | - |
| *ACAD11* | Acyl-CoA Dehydrogenase Family Member 11 | [rs2270801](http://www.ncbi.nlm.nih.gov/projects/SNP/snp_ref.cgi?rs=rs2270801) | [3:132277866](genomebrowse://api/zoom?locus=3:132277866) | 0.0890575 | 0.118666 | 0.0918519 | 0.115 | synonymous_variant | - | - | - |
| *ACADSB* | Acyl-CoA Dehydrogenase Short/Branched Chain | rs58639322 | 10:124800121 | 0.000599042 | 0.00082458 | 0.000678119 | 0.0007907 | missense_variant | Damaging | Possibly damaging | Tolerated |
| *ACADSB* | Acyl-CoA Dehydrogenase Short/Branched Chain | rs76111609 | 10:124802666 | 0.00539137 | 0.00107633 | 0.00364964 | 0.001359 | synonymous_variant | - | - | - |
| *ACAN* | Aggrecan | [rs16942318](http://www.ncbi.nlm.nih.gov/projects/SNP/snp_ref.cgi?rs=rs16942318) | [15:89382129](genomebrowse://api/zoom?locus=15:89382129) | 0.0573083 | 0.0155533 | 0.0458158 | 0.018 | missense_variant | Tolerated | Benign | Tolerated |
| *ACAP3* | ArfGAP With Coiled-Coil, Ankyrin Repeat And PH Domains 3 | rs143898116 | 1:1242477 | 0.000399361 | 0.000100163 | 0.00051723 | 5.623e-05 | intron_variant | - | - | - |
| *ACAT1* | Acetyl-CoA Acetyltransferase 1 | [rs12365364](http://www.ncbi.nlm.nih.gov/projects/SNP/snp_ref.cgi?rs=rs12365364) | [11:108004687](genomebrowse://api/zoom?locus=11:108004687) | 0.0285543 | 0.0112834 | 0.0229067 | 0.012 | intron_variant | - | - | - |
| *ACAT1* | Acetyl-CoA Acetyltransferase 1 | [rs563412144](http://www.ncbi.nlm.nih.gov/projects/SNP/snp_ref.cgi?rs=rs563412144) | [11:108014685](genomebrowse://api/zoom?locus=11:108014685) | 0.192093 | 0.179408 | 0.0641315 | 0.223 | intron_variant | - | - | - |
| *ACBD5* | Acyl-CoA Binding Domain Containing 5 | [rs76818131](http://www.ncbi.nlm.nih.gov/projects/SNP/snp_ref.cgi?rs=rs76818131) | [10:27499709](genomebrowse://api/zoom?locus=10:27499709) | 0.0636981 | 0.0318452 | 0.059863 | 0.035 | intron_variant | - | - | - |
| *ACBD5* | Acyl-CoA Binding Domain Containing 5 | [rs34613194](http://www.ncbi.nlm.nih.gov/projects/SNP/snp_ref.cgi?rs=rs34613194) | [10:27499771](genomebrowse://api/zoom?locus=10:27499771) | 0.0946486 | 0.0454191 | 0.0709071 | 0.049 | synonymous_variant | - | - | - |
| *ACBD5* | Acyl-CoA Binding Domain Containing 5 | [rs34856168](http://www.ncbi.nlm.nih.gov/projects/SNP/snp_ref.cgi?rs=rs34856168) | [10:27499804](genomebrowse://api/zoom?locus=10:27499804) | 0.0946486 | 0.045432 | 0.0709765 | 0.049 | synonymous_variant | - | - | - |
| *ACE* | Angiotensin I Converting Enzyme | rs12709442 | 17:61574215 | 0.00259585 | 0.000730016 | 0.00252133 | 0.0009884 | missense_variant | Damaging | Probably damaging | Damaging |
| *ACE* | Angiotensin I Converting Enzyme | [rs4318](http://www.ncbi.nlm.nih.gov/projects/SNP/snp_ref.cgi?rs=rs4318) | [17:61562373](genomebrowse://api/zoom?locus=17:61562373) | 0.0569089 | 0.0129419 | 0.0503297 | 0.015 | missense_variant | Tolerated | Benign | Tolerated |
| *ACO1* | Aconitase 1 | [rs34319839](http://www.ncbi.nlm.nih.gov/projects/SNP/snp_ref.cgi?rs=rs34319839) | [9:32425898](genomebrowse://api/zoom?locus=9:32425898) | 0.0405351 | 0.0115578 | 0.0385684 | 0.014 | synonymous_variant | - | - | - |
| *ACO1* | Aconitase 1 | [rs35370505](http://www.ncbi.nlm.nih.gov/projects/SNP/snp_ref.cgi?rs=rs35370505) | [9:32427396](genomebrowse://api/zoom?locus=9:32427396) | 0.0537141 | 0.0140322 | 0.0452638 | 0.017 | synonymous_variant | - | - | - |
| *ACOT2* | Acyl-CoA Thioesterase 2 | [rs60568739](http://www.ncbi.nlm.nih.gov/projects/SNP/snp_ref.cgi?rs=rs60568739) | [14:74036370](genomebrowse://api/zoom?locus=14:74036370) | 0.0632987 | 0.0593007 | 0.0591603 | 0.055 | synonymous_variant | - | - | - |
| *ACOX1* | Acyl-CoA Oxidase 1 | [rs8082018](http://www.ncbi.nlm.nih.gov/projects/SNP/snp_ref.cgi?rs=rs8082018) | [17:73945529](genomebrowse://api/zoom?locus=17:73945529) | 0.0714856 | 0.0195988 | 0.0628795 | 0.022 | intron_variant | - | - | - |
| *ACP2* | Acid Phosphatase 2, Lysosomal | [rs2242261](http://www.ncbi.nlm.nih.gov/projects/SNP/snp_ref.cgi?rs=rs2242261) | [11:47266808](genomebrowse://api/zoom?locus=11:47266808) | 0.36222 | 0.256812 | 0.275355 | 0.249 | intron_variant | Tolerated | - | Tolerated |
| *ACSBG1* | Acyl-CoA Synthetase Bubblegum Family Member 1 | [rs17850483](http://www.ncbi.nlm.nih.gov/projects/SNP/snp_ref.cgi?rs=rs17850483) | [15:78466781](genomebrowse://api/zoom?locus=15:78466781) | 0.0908546 | 0.0649306 | 0.0558864 | 0.067 | synonymous_variant | - | - | - |
| *ACSF3* | Acyl-CoA Synthetase Family Member 3 | rs184912682 | 16:89220619 | 0.000798722 | 0.000147936 | 0.000453574 | 0.0001978 | 3_prime_UTR_variant | - | - | - |
| *ACSF3* | Acyl-CoA Synthetase Family Member 3 | [rs1054747](http://www.ncbi.nlm.nih.gov/projects/SNP/snp_ref.cgi?rs=rs1054747) | [16:89220846](genomebrowse://api/zoom?locus=16:89220846) | 0.0628994 | 0.0544267 | 0.0519018 | 0.025 | 3_prime_UTR_variant | - | - | - |
| *ACSF3* | Acyl-CoA Synthetase Family Member 3 | [rs72819317](http://www.ncbi.nlm.nih.gov/projects/SNP/snp_ref.cgi?rs=rs72819317) | [16:89220986](genomebrowse://api/zoom?locus=16:89220986) | 0.0658946 | 0.0553474 | 0.0526863 | 0.035 | 3_prime_UTR_variant | - | - | - |
| *ACSF3* | Acyl-CoA Synthetase Family Member 3 | [rs78403542](http://www.ncbi.nlm.nih.gov/projects/SNP/snp_ref.cgi?rs=rs78403542) | [16:89221287](genomebrowse://api/zoom?locus=16:89221287) | 0.15016 | 0.101893 | 0.0589455 | 0.121 | 3_prime_UTR_variant | - | - | - |
| *ACSL3* | Acyl-CoA Synthetase Long Chain Family Member 3 | [rs13000358](http://www.ncbi.nlm.nih.gov/projects/SNP/snp_ref.cgi?rs=rs13000358) | [2:223783841](genomebrowse://api/zoom?locus=2:223783841) | 0.0225639 | 0.0400749 | 0.0360442 | 0.041 | synonymous_variant | - | - | - |
| *ACSM2A* | Acyl-CoA Synthetase Medium Chain Family Member 2A | [rs34655000](http://www.ncbi.nlm.nih.gov/projects/SNP/snp_ref.cgi?rs=rs34655000) | [16:20476893](genomebrowse://api/zoom?locus=16:20476893) | - | 0.0230976 | 0.0204161 | 0.023 | missense_variant | Damaging | Possibly damaging | Damaging |
| *ACSM2B* | Acyl-CoA Synthetase Medium Chain Family Member 2B | rs138313532 | 16:20559726 | 0.00279553 | 0.00705102 | 0.00750226 | 0.006878 | missense_variant | Tolerated | Benign | Tolerated |
| *ACSM5* | Acyl-CoA Synthetase Medium Chain Family Member 5 | [rs12931611](http://www.ncbi.nlm.nih.gov/projects/SNP/snp_ref.cgi?rs=rs12931611) | [16:20440953](genomebrowse://api/zoom?locus=16:20440953) | 0.107228 | 0.102395 | 0.0840244 | 0.104 | intron_variant | - | - | - |
| *ACSM5* | Acyl-CoA Synthetase Medium Chain Family Member 5 | [rs12932087](http://www.ncbi.nlm.nih.gov/projects/SNP/snp_ref.cgi?rs=rs12932087) | [16:20441020](genomebrowse://api/zoom?locus=16:20441020) | 0.107228 | 0.104308 | 0.0835441 | 0.104 | missense_variant | Tolerated | Benign | Tolerated |
| *ACTL7A* | Actin Like 7A | [rs7872077](http://www.ncbi.nlm.nih.gov/projects/SNP/snp_ref.cgi?rs=rs7872077) | [9:111625620](genomebrowse://api/zoom?locus=9:111625620) | 0.0890575 | 0.0269866 | 0.0471619 | 0.031 | missense_variant | Tolerated | Benign | Tolerated |
| *ACTN4* | Actinin Alpha 4 | [rs12981131](http://www.ncbi.nlm.nih.gov/projects/SNP/snp_ref.cgi?rs=rs12981131) | [19:39218694](genomebrowse://api/zoom?locus=19:39218694) | 0.0786741 | 0.0657608 | 0.0582069 | 0.057 | intron_variant | - | - | - |
| *ACTRT2* | Actin Related Protein T2 | rs35806103 | 1:2938569 | 0.0371406 | 0.0167798 | 0.0324281 | 0.018 | synonymous_variant | - | - | - |
| *ACVR1C* | Activin A Receptor Type 1C | [rs114245489](http://www.ncbi.nlm.nih.gov/projects/SNP/snp_ref.cgi?rs=rs114245489) | [2:158485075](genomebrowse://api/zoom?locus=2:158485075) | 0.0207668 | 0.0395437 | 0.0366723 | 0.036 | intron_variant | - | - | - |
| *AD000090.2* | - | rs45513793 | 19:36033433 | 0.00199681 | 0.00663882 | 0.00634017 | 0.006218 | synonymous_variant | - | - | - |
| *AD000090.2* | - | [rs2239945](http://www.ncbi.nlm.nih.gov/projects/SNP/snp_ref.cgi?rs=rs2239945) | [19:36033460](genomebrowse://api/zoom?locus=19:36033460) | 0.205471 | 0.203783 | 0.135287 | 0.193 | synonymous_variant | - | - | - |
| *ADAM21* | ADAM Metallopeptidase Domain 21 | c.606C>G | [14:70924822](genomebrowse://api/zoom?locus=14:70924822) | - | - | - | - | missense_variant | - | Benign | Tolerated |
| *ADAM21* | ADAM Metallopeptidase Domain 21 | [rs72735760](http://www.ncbi.nlm.nih.gov/projects/SNP/snp_ref.cgi?rs=rs72735760) | [14:70925501](genomebrowse://api/zoom?locus=14:70925501) | 0.0175719 | 0.0333385 | 0.0324029 | 0.035 | missense_variant | - | Benign | Tolerated |
| *ADAM21P1* | ADAM Metallopeptidase Domain 21 Pseudogene 1 | [rs34309925](http://www.ncbi.nlm.nih.gov/projects/SNP/snp_ref.cgi?rs=rs34309925) | [14:70713721](genomebrowse://api/zoom?locus=14:70713721) | 0.139776 | 0.140861 | 0.172395 | 0.144 | non_coding_exon_variant | - | - | - |
| *ADAM22* | ADAM Metallopeptidase Domain 22 | [rs17255978](http://www.ncbi.nlm.nih.gov/projects/SNP/snp_ref.cgi?rs=rs17255978) | [7:87754915](genomebrowse://api/zoom?locus=7:87754915) | 0.0810703 | 0.0652297 | 0.0645276 | 0.064 | missense_variant | Tolerated | Benign | Tolerated |
| *ADAM28* | ADAM Metallopeptidase Domain 28 | [rs7829965](http://www.ncbi.nlm.nih.gov/projects/SNP/snp_ref.cgi?rs=rs7829965) | [8:24207438](genomebrowse://api/zoom?locus=8:24207438) | 0.0798722 | 0.0466277 | 0.0510699 | 0.049 | missense_variant | Tolerated | Benign | Tolerated |
| *ADAM29* | ADAM Metallopeptidase Domain 29 | [rs10009483](http://www.ncbi.nlm.nih.gov/projects/SNP/snp_ref.cgi?rs=rs10009483) | [4:175899021](genomebrowse://api/zoom?locus=4:175899021) | 0.0914537 | 0.0217306 | 0.0917608 | 0.028 | missense_variant | Tolerated | Benign | Tolerated |
| *ADAM33* | ADAM Metallopeptidase Domain 33 | [rs3918392](http://www.ncbi.nlm.nih.gov/projects/SNP/snp_ref.cgi?rs=rs3918392) | [20:3655219](genomebrowse://api/zoom?locus=20:3655219) | 0.066893 | 0.0445052 | 0.0474434 | 0.045 | missense_variant | Tolerated | Benign | Tolerated |
| *ADAM3A* | ADAM Metallopeptidase Domain 3A (Pseudogene) | [rs143674425](http://www.ncbi.nlm.nih.gov/projects/SNP/snp_ref.cgi?rs=rs143674425) | [8:39331452](genomebrowse://api/zoom?locus=8:39331452) | - | 0.144961 | 0.244654 | 0.155 | splice_region_variant | - | - | - |
| *ADAMTS10* | ADAM Metallopeptidase With Thrombospondin Type 1 Motif 10 | [rs73501572](http://www.ncbi.nlm.nih.gov/projects/SNP/snp_ref.cgi?rs=rs73501572) | [19:8654106](genomebrowse://api/zoom?locus=19:8654106) | 0.0704872 | 0.099142 | 0.0947276 | 0.098 | intron_variant | - | - | - |
| *ADAMTS13* | ADAM Metallopeptidase With Thrombospondin Type 1 Motif 13 | rs587731517 | 9:136319671 | 0.000199681 | 0.000120481 | 3.2329e-05 | 0.0001072 | missense_variant | Tolerated | Benign | Damaging |
| *ADAMTS16* | ADAM Metallopeptidase With Thrombospondin Type 1 Motif 16 | [rs77812048](http://www.ncbi.nlm.nih.gov/projects/SNP/snp_ref.cgi?rs=rs77812048) | [5:5242228](genomebrowse://api/zoom?locus=5:5242228) | 0.0732827 | 0.028802 | 0.0310724 | 0.031 | synonymous_variant | - | - | - |
| *ADAMTS17* | ADAM Metallopeptidase With Thrombospondin Type 1 Motif 17 | [rs61752832](http://www.ncbi.nlm.nih.gov/projects/SNP/snp_ref.cgi?rs=rs61752832) | [15:100649248](genomebrowse://api/zoom?locus=15:100649248) | 0.0852636 | 0.0844244 | 0.0856552 | 0.085 | synonymous_variant | - | - | - |
| *ADAMTS17* | ADAM Metallopeptidase With Thrombospondin Type 1 Motif 17 | [rs72755233](http://www.ncbi.nlm.nih.gov/projects/SNP/snp_ref.cgi?rs=rs72755233) | [15:100692953](genomebrowse://api/zoom?locus=15:100692953) | 0.0305511 | 0.071038 | 0.0780199 | 0.073 | missense_variant | Damaging | Benign | Damaging |
| *ADAMTS18* | ADAM Metallopeptidase With Thrombospondin Type 1 Motif 18 | [rs113097720](http://www.ncbi.nlm.nih.gov/projects/SNP/snp_ref.cgi?rs=rs113097720) | [16:77375571](genomebrowse://api/zoom?locus=16:77375571) | 0.0253594 | 0.0281676 | 0.0261848 | 0.029 | intron_variant | - | - | - |
| *ADAMTS18* | ADAM Metallopeptidase With Thrombospondin Type 1 Motif 18 | [rs17620357](http://www.ncbi.nlm.nih.gov/projects/SNP/snp_ref.cgi?rs=rs17620357) | [16:77375581](genomebrowse://api/zoom?locus=16:77375581) | 0.0253594 | 0.0281807 | 0.0261201 | 0.029 | intron_variant | - | - | - |
| *ADAMTS2* | ADAM Metallopeptidase With Thrombospondin Type 1 Motif 2 | [rs140401199](http://www.ncbi.nlm.nih.gov/projects/SNP/snp_ref.cgi?rs=rs140401199) | [5:178557107](genomebrowse://api/zoom?locus=5:178557107) | 0.0301518 | 0.0579981 | 0.0483913 | 0.056 | splice_region_variant | - | - | - |
| *ADAMTS2* | ADAM Metallopeptidase With Thrombospondin Type 1 Motif 2 | [rs35462609](http://www.ncbi.nlm.nih.gov/projects/SNP/snp_ref.cgi?rs=rs35462609) | [5:178608112](genomebrowse://api/zoom?locus=5:178608112) | 0.0107827 | 0.0270211 | 0.0290254 | 0.026 | synonymous_variant | - | - | - |
| *ADAMTS5* | ADAM Metallopeptidase With Thrombospondin Type 1 Motif 5 | [rs61088614](http://www.ncbi.nlm.nih.gov/projects/SNP/snp_ref.cgi?rs=rs61088614) | [21:28315841](genomebrowse://api/zoom?locus=21:28315841) | 0.0501198 | 0.0135878 | 0.041454 | 0.016 | synonymous_variant | - | - | - |
| *ADAT1* | Adenosine Deaminase TRNA Specific 1 | rs141222568 | 16:75646553 | 0.00539137 | 0.00211198 | 0.00319809 | 0.002323 | missense_variant | Damaging | Possibly damaging | Tolerated |
| *ADAT1* | Adenosine Deaminase TRNA Specific 1 | [rs62619985](http://www.ncbi.nlm.nih.gov/projects/SNP/snp_ref.cgi?rs=rs62619985) | [16:75646423](genomebrowse://api/zoom?locus=16:75646423) | 0.061901 | 0.043162 | 0.0672505 | 0.046 | missense_variant | Tolerated | Benign | Tolerated |
| *ADCK4* | AarF Domain-Containing Protein Kinase 4 | [rs4803357](http://www.ncbi.nlm.nih.gov/projects/SNP/snp_ref.cgi?rs=rs4803357) | [19:41209689](genomebrowse://api/zoom?locus=19:41209689) | 0.0153754 | 0.0248632 | 0.0232137 | 0.025 | synonymous_variant | - | - | - |
| *ADCY1* | Adenylate Cyclase 1 | rs78087585 | 7:45650085 | 0.00279553 | 0.00314226 | 0.00449199 | 0.003328 | synonymous_variant | - | - | - |
| *ADCY10* | Adenylate Cyclase 10 | [rs75383572](http://www.ncbi.nlm.nih.gov/projects/SNP/snp_ref.cgi?rs=rs75383572) | [1:167839679](genomebrowse://api/zoom?locus=1:167839679) | 0.0539137 | 0.0712026 | 0.0582263 | 0.068 | intron_variant | - | - | - |
| *ADCY9* | Adenylate Cyclase 9 | [rs11543317](http://www.ncbi.nlm.nih.gov/projects/SNP/snp_ref.cgi?rs=rs11543317) | [16:4016049](genomebrowse://api/zoom?locus=16:4016049) | 0.0297524 | 0.0553787 | 0.0484788 | 0.052 | synonymous_variant | - | - | - |
| *ADCY9* | Adenylate Cyclase 9 | [rs61731445](http://www.ncbi.nlm.nih.gov/projects/SNP/snp_ref.cgi?rs=rs61731445) | [16:4016377](genomebrowse://api/zoom?locus=16:4016377) | 0.0325479 | 0.0558109 | 0.0507078 | 0.053 | missense_variant | Tolerated | Benign | Damaging |
| *ADGB* | Androglobin | [rs41285871](http://www.ncbi.nlm.nih.gov/projects/SNP/snp_ref.cgi?rs=rs41285871) | [6:147022227](genomebrowse://api/zoom?locus=6:147022227) | 0.0467252 | 0.0511045 | 0.0467193 | 0.051 | intron_variant | - | - | - |
| *ADPGK* | ADP Dependent Glucokinase | [rs34149613](http://www.ncbi.nlm.nih.gov/projects/SNP/snp_ref.cgi?rs=rs34149613) | [15:73044863](genomebrowse://api/zoom?locus=15:73044863) | 0.0549121 | 0.0810007 | 0.0757302 | 0.081 | missense_variant | Tolerated | Benign | Tolerated |
| *AFAP1* | Actin Filament Associated Protein 1 | [rs10516189](http://www.ncbi.nlm.nih.gov/projects/SNP/snp_ref.cgi?rs=rs10516189) | [4:7783286](genomebrowse://api/zoom?locus=4:7783286) | 0.205072 | 0.142948 | 0.11221 | 0.134 | synonymous_variant | - | - | - |
| *AFAP1* | Actin Filament Associated Protein 1 | [rs2240053](http://www.ncbi.nlm.nih.gov/projects/SNP/snp_ref.cgi?rs=rs2240053) | [4:7765495](genomebrowse://api/zoom?locus=4:7765495) | 0.241414 | 0.150201 | 0.149233 | 0.08 | synonymous_variant | - | - | - |
| *AFF1* | AF4/FMR2 Family Member 1 | [rs61009470](http://www.ncbi.nlm.nih.gov/projects/SNP/snp_ref.cgi?rs=rs61009470) | [4:87968780](genomebrowse://api/zoom?locus=4:87968780) | 0.034345 | 0.0196723 | 0.0290908 | 0.021 | intron_variant | - | - | - |
| *AFM* | Afamin | [rs78055812](http://www.ncbi.nlm.nih.gov/projects/SNP/snp_ref.cgi?rs=rs78055812) | [4:74361163](genomebrowse://api/zoom?locus=4:74361163) | 0.0447284 | 0.0318646 | 0.0306867 | 0.032 | intron_variant | - | - | - |
| *AFP* | Alpha Fetoprotein | [rs35920062](http://www.ncbi.nlm.nih.gov/projects/SNP/snp_ref.cgi?rs=rs35920062) | [4:74318177](genomebrowse://api/zoom?locus=4:74318177) | 0.0255591 | 0.0501178 | 0.0499386 | 0.05 | synonymous_variant | - | - | - |
| *AFP* | Alpha Fetoprotein | [rs7790](http://www.ncbi.nlm.nih.gov/projects/SNP/snp_ref.cgi?rs=rs7790) | [4:74319538](genomebrowse://api/zoom?locus=4:74319538) | 0.0587061 | 0.0176658 | 0.0446879 | 0.021 | missense_variant | Tolerated | Possibly damaging | Tolerated |
| *AGA* | Aspartylglucosaminidase | rs138699617 | 4:178357453 | 0.000798722 | 0.000247841 | 0.00100207 | 0.0002883 | synonymous_variant | - | - | - |
| *AGAP1* | ArfGAP With GTPase Domain, Ankyrin Repeat And PH Domain 1 | [rs8178993](http://www.ncbi.nlm.nih.gov/projects/SNP/snp_ref.cgi?rs=rs8178993) | [2:236403462](genomebrowse://api/zoom?locus=2:236403462) | 0.0123802 | 0.0189324 | 0.0178808 | 0.019 | synonymous_variant | - | - | - |
| *AGAP7* | Arf-GAP With GTPase, ANK Repeat And PH Domain-Containing Protein 7 Pseudogene | [rs200366013](http://www.ncbi.nlm.nih.gov/projects/SNP/snp_ref.cgi?rs=rs200366013) | [10:51465131](genomebrowse://api/zoom?locus=10:51465131) | 0.0926518 | 0.0212242 | 0.0727953 | 0.017 | missense_variant | - | - | - |
| *AGAP8* | ArfGAP With GTPase Domain, Ankyrin Repeat And PH Domain 8 | [rs587657613](http://www.ncbi.nlm.nih.gov/projects/SNP/snp_ref.cgi?rs=rs587657613) | [10:51225767](genomebrowse://api/zoom?locus=10:51225767) | 0.0549121 | 0.0257104 | 0.0836799 | 0.024 | synonymous_variant | - | - | - |
| *AGAP8* | ArfGAP With GTPase Domain, Ankyrin Repeat And PH Domain 9 | [rs201187723](http://www.ncbi.nlm.nih.gov/projects/SNP/snp_ref.cgi?rs=rs201187723) | [10:51225877](genomebrowse://api/zoom?locus=10:51225877) | 0.0103834 | 0.0470565 | 0.0963499 | 0.03 | missense_variant | - | - | - |
| *AGBL2* | ATP/GTP Binding Protein Like 2 | rs144429450 | 11:47721008 | 0.00838658 | 0.00846599 | 0.00287375 | 0.006787 | synonymous_variant | - | - | - |
| *AGL* | Amylo-Alpha-1, 6-Glucosidase, 4-Alpha-Glucanotransferase | rs147038780 | 1:100340870 | 0.00119808 | 0.000354131 | 0.00146019 | 0.0004201 | intron_variant | - | - | - |
| *AGL* | Amylo-Alpha-1, 6-Glucosidase, 4-Alpha-Glucanotransferase | [rs3753494](http://www.ncbi.nlm.nih.gov/projects/SNP/snp_ref.cgi?rs=rs3753494) | [1:100358103](genomebrowse://api/zoom?locus=1:100358103) | 0.110623 | 0.13492 | 0.136422 | 0.136 | missense_variant | Damaging | Benign | Damaging |
| *AGPAT2* | 1-Acylglycerol-3-Phosphate O-Acyltransferase 2 | rs376439157 | 9:139571149 | 0.00119808 | 0.000655543 | 0.00304168 | 0.0007664 | intron_variant | - | - | - |
| *AGPAT4* | 1-Acylglycerol-3-Phosphate O-Acyltransferase 4 | [rs2277092](http://www.ncbi.nlm.nih.gov/projects/SNP/snp_ref.cgi?rs=rs2277092) | [6:161575165](genomebrowse://api/zoom?locus=6:161575165) | 0.0441294 | 0.079268 | 0.068095 | 0.078 | intron_variant | - | - | - |
| *AGRN* | Agrin | rs148430436 | 1:982969 | 0.000798722 | 0.000597823 | 0.00297446 | 0.0007002 | missense_variant | Tolerated | Possibly damaging | Damaging |
| *AGRN* | Agrin | rs17778478 | 1:987142 | 0.00958466 | 0.00492919 | 0.00598086 | 0.005183 | synonymous_variant | - | - | - |
| *AGRN* | Agrin | rs112917612 | 1:984847 | 0.0611022 | 0.0199093 | 0.0583106 | 0.022 | intron_variant | - | - | - |
| *AGRP* | Agouti Related Neuropeptide | [rs5030980](http://www.ncbi.nlm.nih.gov/projects/SNP/snp_ref.cgi?rs=rs5030980) | [16:67516945](genomebrowse://api/zoom?locus=16:67516945) | 0.0147764 | 0.0318766 | 0.0273503 | 0.032 | missense_variant | Tolerated | Benign | Tolerated |
| *AHNAK* | AHNAK Nucleoprotein | [rs75436331](http://www.ncbi.nlm.nih.gov/projects/SNP/snp_ref.cgi?rs=rs75436331) | [11:62297447](genomebrowse://api/zoom?locus=11:62297447) | 0.0325479 | 0.0282211 | 0.0370071 | 0.032 | missense_variant | Damaging | Benign | Damaging |
| *AHNAK2* | AHNAK Nucleoprotein 2 | rs151077903 | 14:105407931 | 0.00698882 | 0.0017332 | 0.00616606 | 0.00225 | synonymous_variant | - | - | - |
| *AHNAK2* | AHNAK Nucleoprotein 2 | rs201544991 | 14:105412333 | 0.000199681 | 0.000306235 | 0.000163733 | 0.0003332 | missense_variant | Tolerated | Benign | Tolerated |
| *AHNAK2* | AHNAK Nucleoprotein 2 | rs184602855 | 14:105414303 | 0.00579073 | 0.00143942 | 0.00584491 | 0.001869 | synonymous_variant | - | - | - |
| *AHNAK2* | AHNAK Nucleoprotein 2 | rs200641835 | 14:105417229 | 0.00459265 | 0.00111052 | 0.000495238 | 0.001015 | missense_variant | Tolerated | Benign | Tolerated |
| *AHNAK2* | AHNAK Nucleoprotein 2 | [rs61316883](http://www.ncbi.nlm.nih.gov/projects/SNP/snp_ref.cgi?rs=rs61316883) | [14:105411162](genomebrowse://api/zoom?locus=14:105411162) | 0.0251597 | 0.0297211 | 0.0229055 | 0.03 | synonymous_variant | - | - | - |
| *AHNAK2* | AHNAK Nucleoprotein 2 | [rs2819435](http://www.ncbi.nlm.nih.gov/projects/SNP/snp_ref.cgi?rs=rs2819435) | [14:105416220](genomebrowse://api/zoom?locus=14:105416220) | 0.0752796 | 0.0234728 | 0.068302 | 0.028 | missense_variant | Tolerated | Benign | Tolerated |
| *AHNAK2* | AHNAK Nucleoprotein 2 | [rs2819435](http://www.ncbi.nlm.nih.gov/projects/SNP/snp_ref.cgi?rs=rs2819435) | [14:105416220](genomebrowse://api/zoom?locus=14:105416220) | 0.721845,0.0752796 | 0.757618,0.0234728 | 0.768098,0.068302 | 0.759,0.028 | missense_variant | Tolerated | Benign | Tolerated |
| *AHNAK2* | AHNAK Nucleoprotein 2 | [rs11848082](http://www.ncbi.nlm.nih.gov/projects/SNP/snp_ref.cgi?rs=rs11848082) | [14:105416621](genomebrowse://api/zoom?locus=14:105416621) | 0.0433307 | 0.010479 | 0.0404026 | 0.013 | missense_variant | Tolerated | Benign | Tolerated |
| *AHNAK2* | AHNAK Nucleoprotein 2 | [rs143480617](http://www.ncbi.nlm.nih.gov/projects/SNP/snp_ref.cgi?rs=rs143480617) | [14:105416790](genomebrowse://api/zoom?locus=14:105416790) | 0.038139 | 0.0147556 | 0.0262381 | 0.017 | synonymous_variant | - | - | - |
| *AHNAK2* | AHNAK Nucleoprotein 2 | [rs149840830](http://www.ncbi.nlm.nih.gov/projects/SNP/snp_ref.cgi?rs=rs149840830) | [14:105417286](genomebrowse://api/zoom?locus=14:105417286) | 0.0543131 | 0.0127962 | 0.0599103 | 0.015 | missense_variant | Damaging | Probably damaging | Damaging |
| *AHNAK2* | AHNAK Nucleoprotein 2 | [rs77667449](http://www.ncbi.nlm.nih.gov/projects/SNP/snp_ref.cgi?rs=rs77667449) | [14:105417353](genomebrowse://api/zoom?locus=14:105417353) | 0.126797 | 0.0453631 | 0.013009 | 0.142 | missense_variant | Tolerated | Benign | Tolerated |
| *AHNAK2* | AHNAK Nucleoprotein 2 | [rs75990247](http://www.ncbi.nlm.nih.gov/projects/SNP/snp_ref.cgi?rs=rs75990247) | [14:105417358](genomebrowse://api/zoom?locus=14:105417358) | 0.1252 | 0.0257321 | 0.0122281 | 0.107 | missense_variant | Tolerated | Benign | Tolerated |
| *AHNAK2* | AHNAK Nucleoprotein 2 | [rs141283979](http://www.ncbi.nlm.nih.gov/projects/SNP/snp_ref.cgi?rs=rs141283979) | [14:105417581](genomebrowse://api/zoom?locus=14:105417581) | 0.0217652 | 0.0526906 | 0.0402176 | 0.053 | missense_variant | Tolerated | Possibly damaging | Damaging |
| *AIFM1* | Apoptosis Inducing Factor Mitochondria Associated 1 | rs73556209 | X:129263541 | 0.00529801 | 0.00146556 | 0.00454904 | 0.001779 | synonymous_variant | - | - | - |
| *AIFM2* | Apoptosis Inducing Factor Mitochondria Associated 2 | [rs2271694](http://www.ncbi.nlm.nih.gov/projects/SNP/snp_ref.cgi?rs=rs2271694) | [10:71874784](genomebrowse://api/zoom?locus=10:71874784) | 0.0658946 | 0.0453851 | 0.0229651 | 0.04 | missense_variant | Tolerated | Benign | Tolerated |
| *AIMP1* | Aminoacyl TRNA Synthetase Complex Interacting Multifunctional Protein 1 | [rs1134648](http://www.ncbi.nlm.nih.gov/projects/SNP/snp_ref.cgi?rs=rs1134648) | [4:107249244](genomebrowse://api/zoom?locus=4:107249244) | 0.115216 | 0.0674964 | 0.0252166 | 0.062 | missense_variant | Tolerated | Benign | Tolerated |
| *AIMP1* | Aminoacyl TRNA Synthetase Complex Interacting Multifunctional Protein 1 | [rs3109956](http://www.ncbi.nlm.nih.gov/projects/SNP/snp_ref.cgi?rs=rs3109956) | [4:107253063](genomebrowse://api/zoom?locus=4:107253063) | 0.115016 | 0.0676031 | 0.0251696 | 0.062 | intron_variant | - | - | - |
| *AIMP1* | Aminoacyl TRNA Synthetase Complex Interacting Multifunctional Protein 1 | [rs3109954](http://www.ncbi.nlm.nih.gov/projects/SNP/snp_ref.cgi?rs=rs3109954) | [4:107258227](genomebrowse://api/zoom?locus=4:107258227) | 0.129792 | 0.0855217 | 0.0436146 | 0.08 | intron_variant | - | - | - |
| *AIPL1* | Aryl Hydrocarbon Receptor Interacting Protein Like 1 | rs188779461 | 17:6330187 | 0.00658946 | 0.0015729 | 0.00581433 | 0.002249 | intron_variant | - | - | - |
| *AK2* | Adenylate Kinase 2 | rs370568856 | 1:33475962 | 0.00199681 | 0.00045367 | 0.000129132 | 0.0006328 | 3_prime_UTR_variant | - | - | - |
| *AK2* | Adenylate Kinase 2 | rs2335940 | 1:33502433 | 0.00199681 | 0.000578477 | 0.00300465 | 0.0006062 | 5_prime_UTR_variant | - | - | - |
| *AK7* | Adenylate Kinase 7 | [rs80304258](http://www.ncbi.nlm.nih.gov/projects/SNP/snp_ref.cgi?rs=rs80304258) | [14:96953412](genomebrowse://api/zoom?locus=14:96953412) | 0.0555112 | 0.0550681 | 0.050758 | 0.054 | intron_variant | - | - | - |
| *AK9* | Adenylate Kinase 9 | rs150078049 | 6:109837147 | 0.0061901 | 0.00940756 | 0.0075273 | 0.01 | synonymous_variant | - | - | - |
| *AK9* | Adenylate Kinase 9 | [rs75733561](http://www.ncbi.nlm.nih.gov/projects/SNP/snp_ref.cgi?rs=rs75733561) | [6:109935606](genomebrowse://api/zoom?locus=6:109935606) | 0.0541134 | 0.037688 | 0.026076 | 0.042 | missense_variant | Tolerated | Benign | Tolerated |
| *AKAP13* | A-Kinase Anchoring Protein 13 | rs75785836 | 15:86270398 | 0.00738818 | 0.00126755 | 0.00461558 | 0.001614 | synonymous_variant | - | - | - |
| *AKAP13* | A-Kinase Anchoring Protein 13 | [rs61731243](http://www.ncbi.nlm.nih.gov/projects/SNP/snp_ref.cgi?rs=rs61731243) | [15:86124616](genomebrowse://api/zoom?locus=15:86124616) | 0.0690895 | 0.0415106 | 0.0429218 | 0.043 | missense_variant | Damaging | Benign | Tolerated |
| *AKAP5* | A-Kinase Anchoring Protein 5 | [rs2230491](http://www.ncbi.nlm.nih.gov/projects/SNP/snp_ref.cgi?rs=rs2230491) | [14:64935411](genomebrowse://api/zoom?locus=14:64935411) | 0.071885 | 0.117563 | 0.0963676 | 0.118 | missense_variant | Tolerated | Benign | Tolerated |
| *AKAP9* | A-Kinase Anchoring Protein 9 | [rs59090575](http://www.ncbi.nlm.nih.gov/projects/SNP/snp_ref.cgi?rs=rs59090575) | [7:91695717](genomebrowse://api/zoom?locus=7:91695717) | 0.0503195 | 0.0118873 | 0.0342054 | 0.015 | intron_variant | - | - | - |
| *AKR1A1* | Aldo-Keto Reductase Family 1 Member A1 | [rs2229540](http://www.ncbi.nlm.nih.gov/projects/SNP/snp_ref.cgi?rs=rs2229540) | [1:46032311](genomebrowse://api/zoom?locus=1:46032311) | 0.0181709 | 0.0452806 | 0.0458126 | 0.045 | missense_variant | Damaging | Probably damaging | Damaging |
| *AKR1B10* | Aldo-Keto Reductase Family 1 Member B10 | [rs2303312](http://www.ncbi.nlm.nih.gov/projects/SNP/snp_ref.cgi?rs=rs2303312) | [7:134216684](genomebrowse://api/zoom?locus=7:134216684) | 0.0656949 | 0.0364433 | 0.0242201 | 0.037 | missense_variant | Tolerated | Benign | Tolerated |
| *AKR1C1* | Aldo-Keto Reductase Family 1 Member C1 | [rs5001362](http://www.ncbi.nlm.nih.gov/projects/SNP/snp_ref.cgi?rs=rs5001362) | [10:5018132](genomebrowse://api/zoom?locus=10:5018132) | 0.6873 | 0.645977 | 0.667646 | 0.497 | splice_region_variant | - | - | - |
| *AKR1C2* | Aldo-Keto Reductase Family 1 Member C2 | [rs200698968](http://www.ncbi.nlm.nih.gov/projects/SNP/snp_ref.cgi?rs=rs200698968) | [10:5034016](genomebrowse://api/zoom?locus=10:5034016) | 0.321286 | 0.301084 | 0.228435 | 0.131 | splice_region_variant | - | - | - |
| *AKR1CL1* | Aldo-Keto Reductase Family 1, Member C-Like 1 | [rs77303730](http://www.ncbi.nlm.nih.gov/projects/SNP/snp_ref.cgi?rs=rs77303730) | [10:5203772](genomebrowse://api/zoom?locus=10:5203772) | 0.0365415 | 0.0663256 | 0.0635864 | 0.066 | intron_variant | - | - | - |
| *AKR1E2* | Aldo-Keto Reductase Family 1 Member E2 | [rs17133693](http://www.ncbi.nlm.nih.gov/projects/SNP/snp_ref.cgi?rs=rs17133693) | [10:4875591](genomebrowse://api/zoom?locus=10:4875591) | 0.0421326 | 0.0395128 | 0.0403721 | 0.041 | missense_variant | Tolerated | Benign | Tolerated |
| *AKR1E2* | Aldo-Keto Reductase Family 1 Member E2 | [rs12240276](http://www.ncbi.nlm.nih.gov/projects/SNP/snp_ref.cgi?rs=rs12240276) | [10:4889403](genomebrowse://api/zoom?locus=10:4889403) | 0.0784744 | 0.0949951 | 0.112224 | 0.097 | stop_gained | - | - | Damaging |
| *AKT2* | AKT Serine/Threonine Kinase 2 | [rs3730259](http://www.ncbi.nlm.nih.gov/projects/SNP/snp_ref.cgi?rs=rs3730259) | [19:40747820](genomebrowse://api/zoom?locus=19:40747820) | 0.0345447 | 0.0126533 | 0.0292369 | 0.014 | intron_variant | - | - | - |
| *ALAD* | Aminolevulinate Dehydratase | [rs1805312](http://www.ncbi.nlm.nih.gov/projects/SNP/snp_ref.cgi?rs=rs1805312) | [9:116152990](genomebrowse://api/zoom?locus=9:116152990) | 0.0978435 | 0.113577 | 0.0749451 | 0.1 | intron_variant | - | - | - |
| *ALAD* | Aminolevulinate Dehydratase | [rs1800435](http://www.ncbi.nlm.nih.gov/projects/SNP/snp_ref.cgi?rs=rs1800435) | [9:116153891](genomebrowse://api/zoom?locus=9:116153891) | 0.0634984 | 0.0829994 | 0.0557099 | 0.083 | missense_variant | Tolerated | Benign | Tolerated |
| *ALCAM* | Activated Leukocyte Cell Adhesion Molecule | [rs34926152](http://www.ncbi.nlm.nih.gov/projects/SNP/snp_ref.cgi?rs=rs34926152) | [3:105264176](genomebrowse://api/zoom?locus=3:105264176) | 0.0209665 | 0.0346475 | 0.0331257 | 0.034 | missense_variant | Tolerated | Benign | Damaging |
| *ALDH18A1* | Aldehyde Dehydrogenase 18 Family Member A1 | [rs1804934](http://www.ncbi.nlm.nih.gov/projects/SNP/snp_ref.cgi?rs=rs1804934) | [10:97371146](genomebrowse://api/zoom?locus=10:97371146) | 0.0249601 | 0.0229924 | 0.0260642 | 0.022 | synonymous_variant | - | - | - |
| *ALDH1A3* | Aldehyde Dehydrogenase 1 Family Member A3 | [rs3809521](http://www.ncbi.nlm.nih.gov/projects/SNP/snp_ref.cgi?rs=rs3809521) | [15:101432681](genomebrowse://api/zoom?locus=15:101432681) | 0.0601038 | 0.0174164 | 0.0470223 | 0.02 | intron_variant | - | - | - |
| *ALDH1L1* | Aldehyde Dehydrogenase 1 Family Member L1 | rs147597514 | 3:125836862 | 0.00119808 | 0.00433974 | 0.00520227 | 0.004143 | synonymous_variant | - | - | - |
| *ALDH3B2* | Aldehyde Dehydrogenase 3 Family Member B2 | [rs17856219](http://www.ncbi.nlm.nih.gov/projects/SNP/snp_ref.cgi?rs=rs17856219) | [11:67431914](genomebrowse://api/zoom?locus=11:67431914) | 0.0708866 | 0.0785366 | 0.0753837 | 0.079 | missense_variant | Damaging | Probably damaging | Tolerated |
| *ALDH4A1* | Aldehyde Dehydrogenase 4 Family Member A1 | rs138788183 | 1:19209615 | 0.00219649 | 0.0048412 | 0.00371303 | 0.004638 | splice_region_variant | - | - | - |
| *ALDH5A1* | Aldehyde Dehydrogenase 5 Family Member A1 | rs113591366 | 6:24505165 | 0.00579073 | 0.00179099 | 0.00674411 | 0.00238 | synonymous_variant | - | - | - |
| *ALDH7A1* | Aldehyde Dehydrogenase 7 Family Member A1 | rs113775968 | 5:125890105 | 0.00499201 | 0.0014021 | 0.0058117 | 0.001845 | intron_variant | - | - | - |
| *ALDH7A1* | Aldehyde Dehydrogenase 7 Family Member A1 | [rs57902950](http://www.ncbi.nlm.nih.gov/projects/SNP/snp_ref.cgi?rs=rs57902950) | [5:125911132](genomebrowse://api/zoom?locus=5:125911132) | 0.0359425 | 0.0119014 | 0.0177729 | 0.013 | synonymous_variant | - | - | - |
| *ALDH7A1* | Aldehyde Dehydrogenase 7 Family Member A1 | [rs79544459](http://www.ncbi.nlm.nih.gov/projects/SNP/snp_ref.cgi?rs=rs79544459) | [5:125912915](genomebrowse://api/zoom?locus=5:125912915) | 0.0265575 | 0.0193355 | 0.031906 | 0.02 | intron_variant | - | - | - |
| *ALDH8A1* | Aldehyde Dehydrogenase 8 Family Member A1 | rs61731731 | 6:135239816 | 0.00139776 | 0.00438236 | 0.00342355 | 0.005115 | missense_variant | Damaging | Possibly damaging | Damaging |
| *ALG12* | ALG12 Alpha-1,6-Mannosyltransferase | rs114264124 | 22:50302962 | 0.00299521 | 0.00123039 | 0.00488073 | 0.001623 | missense_variant | Tolerated | Benign | Tolerated |
| *ALG12* | ALG12 Alpha-1,6-Mannosyltransferase | [rs3922872](http://www.ncbi.nlm.nih.gov/projects/SNP/snp_ref.cgi?rs=rs3922872) | [22:50297888](genomebrowse://api/zoom?locus=22:50297888) | 0.0916534 | 0.110961 | 0.0966332 | 0.112 | missense_variant | Damaging | Probably damaging | Damaging |
| *ALG12* | ALG12 Alpha-1,6-Mannosyltransferase | [rs11705497](http://www.ncbi.nlm.nih.gov/projects/SNP/snp_ref.cgi?rs=rs11705497) | [22:50307184](genomebrowse://api/zoom?locus=22:50307184) | 0.0702875 | 0.093623 | 0.0852713 | 0.093 | intron_variant | - | - | - |
| *ALG8* | ALG8 Alpha-1,3-Glucosyltransferase | [rs61995924](http://www.ncbi.nlm.nih.gov/projects/SNP/snp_ref.cgi?rs=rs61995924) | [11:77823732](genomebrowse://api/zoom?locus=11:77823732) | 0.0451278 | 0.0113952 | 0.0418388 | 0.014 | synonymous_variant | - | - | - |
| *ALK* | ALK Receptor Tyrosine Kinase | rs200110390 | 2:29430186 | 0.000399361 | 7.84502e-05 | 0.000355114 | 0.0001236 | intron_variant | - | - | - |
| *ALK* | ALK Receptor Tyrosine Kinase | [rs35093491](http://www.ncbi.nlm.nih.gov/projects/SNP/snp_ref.cgi?rs=rs35093491) | [2:29543736](genomebrowse://api/zoom?locus=2:29543736) | 0.0159744 | 0.0285806 | 0.021291 | 0.029 | missense_variant | Tolerated | Benign | Damaging |
| *ALK* | ALK Receptor Tyrosine Kinase | [rs948241251](http://www.ncbi.nlm.nih.gov/projects/SNP/snp_ref.cgi?rs=rs948241251) | [2:29940530](genomebrowse://api/zoom?locus=2:29940530) | - | - | - | - | missense_variant | Damaging | Benign | Damaging |
| *ALKBH1* | AlkB Homolog 1, Histone H2A Dioxygenase | [rs6494](http://www.ncbi.nlm.nih.gov/projects/SNP/snp_ref.cgi?rs=rs6494) | [14:78140355](genomebrowse://api/zoom?locus=14:78140355) | 0.11861 | 0.175561 | 0.176433 | 0.176 | missense_variant | Tolerated | Benign | Tolerated |
| *ALMS1* | ALMS1 Centrosome And Basal Body Associated Protein | [rs112034360](http://www.ncbi.nlm.nih.gov/projects/SNP/snp_ref.cgi?rs=rs112034360) | [2:73677548](genomebrowse://api/zoom?locus=2:73677548) | 0.0421326 | 0.0453204 | 0.0671341 | 0.047 | synonymous_variant | - | - | - |
| *ALMS1* | ALMS1 Centrosome And Basal Body Associated Protein | [rs116733486](http://www.ncbi.nlm.nih.gov/projects/SNP/snp_ref.cgi?rs=rs116733486) | [2:73830454](genomebrowse://api/zoom?locus=2:73830454) | 0.08127 | 0.029611 | 0.0667895 | 0.038 | intron_variant | - | - | - |
| *ALMS1* | ALMS1 Centrosome And Basal Body Associated Protein | [rs114375547](http://www.ncbi.nlm.nih.gov/projects/SNP/snp_ref.cgi?rs=rs114375547) | [2:73830455](genomebrowse://api/zoom?locus=2:73830455) | 0.08127 | 0.0293187 | 0.0669123 | 0.038 | intron_variant | - | - | - |
| *ALMS1* | ALMS1 Centrosome And Basal Body Associated Protein | [rs17848872](http://www.ncbi.nlm.nih.gov/projects/SNP/snp_ref.cgi?rs=rs17848872) | [2:73830458](genomebrowse://api/zoom?locus=2:73830458) | 0.08127 | 0.0317242 | 0.0668347 | 0.038 | intron_variant | - | - | - |
| *ALOX15* | Arachidonate 15-Lipoxygenase | [rs743646](http://www.ncbi.nlm.nih.gov/projects/SNP/snp_ref.cgi?rs=rs743646) | [17:4536241](genomebrowse://api/zoom?locus=17:4536241) | 0.0463259 | 0.0846762 | 0.0766092 | 0.085 | synonymous_variant | - | - | - |
| *ALPK2* | Alpha Kinase 2 | [rs114432784](http://www.ncbi.nlm.nih.gov/projects/SNP/snp_ref.cgi?rs=rs114432784) | [18:56246682](genomebrowse://api/zoom?locus=18:56246682) | 0.0335463 | 0.0186933 | 0.0165407 | 0.019 | synonymous_variant | - | - | - |
| *ALPP* | Alkaline Phosphatase, Placental | [rs2853378](http://www.ncbi.nlm.nih.gov/projects/SNP/snp_ref.cgi?rs=rs2853378) | [2:233245026](genomebrowse://api/zoom?locus=2:233245026) | 0.10004 | 0.0791305 | 0.0730955 | 0.091 | missense_variant | Tolerated | Benign | Tolerated |
| *ALS2* | Alsin Rho Guanine Nucleotide Exchange Factor ALS2 | rs147284131 | 2:202619288 | 0.000199681 | 0.000272183 | 9.68992e-05 | 0.0002316 | synonymous_variant | - | - | - |
| *ALS2* | Alsin Rho Guanine Nucleotide Exchange Factor ALS2 | [rs3219161](http://www.ncbi.nlm.nih.gov/projects/SNP/snp_ref.cgi?rs=rs3219161) | [2:202593280](genomebrowse://api/zoom?locus=2:202593280) | 0.115016 | 0.0954824 | 0.0914441 | 0.098 | synonymous_variant | - | - | - |
| *ALS2CL* | ALS2 C-Terminal Like | rs142971127 | 3:46717175 | 0.00339457 | 0.00757872 | 0.00711008 | 0.008006 | missense_variant | Tolerated | Possibly damaging | Damaging |
| *ALS2CL* | ALS2 C-Terminal Like | [rs57097145](http://www.ncbi.nlm.nih.gov/projects/SNP/snp_ref.cgi?rs=rs57097145) | [3:46722721](genomebrowse://api/zoom?locus=3:46722721) | 0.0495208 | 0.0181417 | 0.0505837 | 0.02 | intron_variant | - | - | - |
| *AMBN* | Ameloblastin | [rs72654387](http://www.ncbi.nlm.nih.gov/projects/SNP/snp_ref.cgi?rs=rs72654387) | [4:71472164](genomebrowse://api/zoom?locus=4:71472164) | 0.0175719 | 0.0403054 | 0.0372598 | 0.042 | missense_variant | Damaging | Probably damaging | Damaging |
| *AMDHD2* | Amidohydrolase Domain Containing 2 | [rs13331643](http://www.ncbi.nlm.nih.gov/projects/SNP/snp_ref.cgi?rs=rs13331643) | [16:2580912](genomebrowse://api/zoom?locus=16:2580912) | 0.0714856 | 0.0172809 | 0.0638325 | 0.021 | missense_variant | - | Benign | Tolerated |
| *AMH* | Anti-Mullerian Hormone | rs140765565 | 19:2251247 | 0.00479233 | 0.00471114 | 0.0084878 | 0.00459 | missense_variant | Tolerated | Possibly damaging | Tolerated |
| *AMPD1* | Adenosine Monophosphate Deaminase 1 | [rs34526199](http://www.ncbi.nlm.nih.gov/projects/SNP/snp_ref.cgi?rs=rs34526199) | [1:115222237](genomebrowse://api/zoom?locus=1:115222237) | 0.0109824 | 0.0288208 | 0.0326596 | 0.028 | missense_variant | Damaging | Probably damaging | Damaging |
| *AMPD3* | Adenosine Monophosphate Deaminase 3 | [rs73409951](http://www.ncbi.nlm.nih.gov/projects/SNP/snp_ref.cgi?rs=rs73409951) | [11:10500056](genomebrowse://api/zoom?locus=11:10500056) | 0.135583 | 0.0931728 | 0.12601 | 0.098 | intron_variant | - | - | - |
| *AMY2B* | Amylase Alpha 2B | [rs140978983](http://www.ncbi.nlm.nih.gov/projects/SNP/snp_ref.cgi?rs=rs140978983) | [1:104117921](genomebrowse://api/zoom?locus=1:104117921) | 0.0173722 | 0.023403 | 0.0175961 | 0.023 | missense_variant | Damaging | Probably damaging | Damaging |
| *ANAPC7* | Anaphase Promoting Complex Subunit 7 | [rs141147170](http://www.ncbi.nlm.nih.gov/projects/SNP/snp_ref.cgi?rs=rs141147170) | [12:110841437](genomebrowse://api/zoom?locus=12:110841437) | 0.0109824 | 0.0303608 | 0.0316133 | 0.025 | missense_variant | Damaging | Benign | Tolerated |
| *ANGPT2* | Angiopoietin 2 | rs201078106 | 8:6357431 | 0.000399361 | 8.12222e-05 | 0.000193673 | 0.0001076 | missense_variant | Damaging | Benign | Tolerated |
| *ANGPTL4* | Angiopoietin Like 4 | [rs11672433](http://www.ncbi.nlm.nih.gov/projects/SNP/snp_ref.cgi?rs=rs11672433) | [19:8438716](genomebrowse://api/zoom?locus=19:8438716) | 0.0555112 | 0.102733 | 0.0953351 | 0.103 | synonymous_variant | - | - | - |
| *ANGPTL6* | Angiopoietin Like 6 | rs76783850 | 19:10204143 | 0.00359425 | 0.00694016 | 0.0057527 | 0.007223 | synonymous_variant | - | - | - |
| *ANK1* | Ankyrin 1 | [rs10093583](http://www.ncbi.nlm.nih.gov/projects/SNP/snp_ref.cgi?rs=rs10093583) | [8:41548003](genomebrowse://api/zoom?locus=8:41548003) | 0.0403355 | 0.0132075 | 0.0397907 | 0.015 | missense_variant | Tolerated | Benign | Tolerated |
| *ANK2* | Ankyrin 2 | rs150878494 | 4:114277676 | 0.000399361 | 0.00143402 | 0.00090451 | 0.00131 | synonymous_variant | - | - | - |
| *ANKAR* | Ankyrin And Armadillo Repeat Containing | [rs16831887](http://www.ncbi.nlm.nih.gov/projects/SNP/snp_ref.cgi?rs=rs16831887) | [2:190571776](genomebrowse://api/zoom?locus=2:190571776) | 0.0323482 | 0.0395978 | 0.0355366 | 0.041 | missense_variant | Tolerated | - | Tolerated |
| *ANKEF1* | Ankyrin Repeat And EF-Hand Domain Containing 1 | [rs6087119](http://www.ncbi.nlm.nih.gov/projects/SNP/snp_ref.cgi?rs=rs6087119) | [20:10036202](genomebrowse://api/zoom?locus=20:10036202) | 0.0491214 | 0.0895899 | 0.106192 | 0.09 | missense_variant | Tolerated | Benign | Damaging |
| *ANKH* | ANKH Inorganic Pyrophosphate Transport Regulator | rs74355706 | 5:14769139 | 0.00698882 | 0.00199964 | 0.00787554 | 0.002487 | synonymous_variant | - | - | - |
| *ANKH* | ANKH Inorganic Pyrophosphate Transport Regulator | [rs78431233](http://www.ncbi.nlm.nih.gov/projects/SNP/snp_ref.cgi?rs=rs78431233) | [5:14871560](genomebrowse://api/zoom?locus=5:14871560) | 0.0215655 | 0.0543847 | 0.0616081 | 0.055 | 5_prime_UTR_variant | - | - | - |
| *ANKIB1* | Ankyrin Repeat And IBR Domain Containing 1 | rs371452332 | 7:92025726 | 0.00239617 | 0.000694605 | 0.00219525 | 0.0007541 | synonymous_variant | - | - | - |
| *ANKMY1* | Ankyrin Repeat And MYND Domain Containing 1 | rs150525285 | 2:241468750 | 0.000998403 | 0.00743978 | 0.00746317 | 0.007536 | synonymous_variant | - | - | - |
| *ANKMY1* | Ankyrin Repeat And MYND Domain Containing 1 | rs571488029 | 2:241492305 | 0.00599042 | 0.00127355 | 0.00371519 | 0.001499 | intron_variant | - | - | - |
| *ANKMY1* | Ankyrin Repeat And MYND Domain Containing 1 | [rs3821348](http://www.ncbi.nlm.nih.gov/projects/SNP/snp_ref.cgi?rs=rs3821348) | [2:241463453](genomebrowse://api/zoom?locus=2:241463453) | 0.163339 | 0.170246 | 0.161458 | 0.173 | missense_variant | Tolerated | Benign | Tolerated |
| *ANKMY2* | Ankyrin Repeat And MYND Domain Containing 2 | [rs11540037](http://www.ncbi.nlm.nih.gov/projects/SNP/snp_ref.cgi?rs=rs11540037) | [7:16655387](genomebrowse://api/zoom?locus=7:16655387) | 0.176318 | 0.203811 | 0.171409 | 0.205 | synonymous_variant | - | - | - |
| *ANKRD12* | Ankyrin Repeat Domain 12 | [rs34996750](http://www.ncbi.nlm.nih.gov/projects/SNP/snp_ref.cgi?rs=rs34996750) | [18:9256258](genomebrowse://api/zoom?locus=18:9256258) | 0.0133786 | 0.0285378 | 0.0299028 | 0.028 | missense_variant | Tolerated | Benign | Tolerated |
| *ANKRD17* | Ankyrin Repeat Domain 17 | rs146616951 | 4:73951003 | 0.000399361 | 0.000986994 | 0.0013272 | 0.001079 | synonymous_variant | - | - | - |
| *ANKRD20A1* | Ankyrin Repeat Domain 20 Family Member A1 | [rs56839104](http://www.ncbi.nlm.nih.gov/projects/SNP/snp_ref.cgi?rs=rs56839104) | [9:67964999](genomebrowse://api/zoom?locus=9:67964999) | 0.061901 | 0.0360042 | 0.0593705 | 0.02 | splice_region_variant | - | - | - |
| *ANKRD20A5P* | Ankyrin Repeat Domain 20 Family Member A5, Pseudogene | [rs7230059](http://www.ncbi.nlm.nih.gov/projects/SNP/snp_ref.cgi?rs=rs7230059) | [18:14186431](genomebrowse://api/zoom?locus=18:14186431) | 0.0976438 | 0.0472033 | 0.34249 | 0.063 | intron_variant | - | - | - |
| *ANKRD20A8P* | Ankyrin Repeat Domain 20 Family Member A8, Pseudogene | [rs2633908](http://www.ncbi.nlm.nih.gov/projects/SNP/snp_ref.cgi?rs=rs2633908) | [2:95481396](genomebrowse://api/zoom?locus=2:95481396) | 0.159744 | 0.0528653 | 0.180214 | 0.07 | non_coding_exon_variant | - | - | - |
| *ANKRD26* | Ankyrin Repeat Domain 26 | rs61730102 | 10:27366407 | 0.00339457 | 0.000548379 | 0.00258431 | 0.0007863 | missense_variant | Damaging | Possibly damaging | Tolerated |
| *ANKRD26* | Ankyrin Repeat Domain 26 | [rs11015496](http://www.ncbi.nlm.nih.gov/projects/SNP/snp_ref.cgi?rs=rs11015496) | [10:27350056](genomebrowse://api/zoom?locus=10:27350056) | 0.0972444 | 0.0689852 | 0.0857956 | 0.071 | intron_variant | - | - | - |
| *ANKRD26* | Ankyrin Repeat Domain 26 | [rs1050767212](http://www.ncbi.nlm.nih.gov/projects/SNP/snp_ref.cgi?rs=rs1050767212) | [10:27356054](genomebrowse://api/zoom?locus=10:27356054) | - | - | - | - | intron_variant | - | - | - |
| *ANKRD30A* | Ankyrin Repeat Domain 30A | [rs61737412](http://www.ncbi.nlm.nih.gov/projects/SNP/snp_ref.cgi?rs=rs61737412) | [10:37419218](genomebrowse://api/zoom?locus=10:37419218) | 0.0413339 | 0.0609301 | 0.0606384 | 0.066 | missense_variant | Damaging | Possibly damaging | Tolerated |
| *ANKRD33B* | Ankyrin Repeat Domain 33B | rs570742268 | 5:10564732 | 0.000199681 | 0.000235291 | 0.000291526 | 0.000342 | synonymous_variant | - | - | - |
| *ANKRD36* | Ankyrin Repeat Domain 36 | [rs112213180](http://www.ncbi.nlm.nih.gov/projects/SNP/snp_ref.cgi?rs=rs112213180) | [2:97830053](genomebrowse://api/zoom?locus=2:97830053) | - | 0.110413 | 0.407568 | 0.175 | splice_region_variant | - | - | - |
| *ANKRD36* | Ankyrin Repeat Domain 36 | [rs954163560](http://www.ncbi.nlm.nih.gov/projects/SNP/snp_ref.cgi?rs=rs954163560) | [2:97851107](genomebrowse://api/zoom?locus=2:97851107) | - | - | 0.114542 | - | intron_variant | - | - | - |
| *ANKRD39* | Ankyrin Repeat Domain 39 | rs138950031 | 2:97523635 | 0.00299521 | 0.000739132 | 0.00222998 | 0.0007825 | synonymous_variant | - | - | - |
| *ANKRD44* | Ankyrin Repeat Domain 44 | [rs35539834](http://www.ncbi.nlm.nih.gov/projects/SNP/snp_ref.cgi?rs=rs35539834) | [2:197954717](genomebrowse://api/zoom?locus=2:197954717) | 0.0117812 | 0.0117228 | 0.0148198 | 0.012 | synonymous_variant | - | - | - |
| *ANKRD7* | Ankyrin Repeat Domain 7 | rs189882379 | 7:117879968 | 0.000798722 | 0.00167667 | 0.00109826 | 0.001573 | missense_variant | Damaging | Benign | Damaging |
| *ANKUB1* | Ankyrin Repeat And Ubiquitin Domain Containing 1 | rs77152758 | 3:149498132 | 0.00379393 | 0.00768281 | 0.00839522 | 0.007263 | synonymous_variant | - | - | - |
| *ANKZF1* | Ankyrin Repeat And Zinc Finger Peptidyl TRNA Hydrolase 1 | [rs2293079](http://www.ncbi.nlm.nih.gov/projects/SNP/snp_ref.cgi?rs=rs2293079) | [2:220100787](genomebrowse://api/zoom?locus=2:220100787) | 0.076278 | 0.104556 | 0.0959165 | 0.105 | missense_variant | Damaging | Benign | Tolerated |
| *ANO3* | Anoctamin 3 | rs72877805 | 11:26586990 | 0.000399361 | 0.000252176 | 0.00019425 | 0.0002306 | missense_variant | Damaging | Benign | Tolerated |
| *ANO5* | Anoctamin 5 | rs115750596 | 11:22249088 | 0.00938498 | 0.00235226 | 0.00879576 | 0.002851 | missense_variant | Tolerated | Benign | Damaging |
| *ANO5* | Anoctamin 5 | [rs773561937](http://www.ncbi.nlm.nih.gov/projects/SNP/snp_ref.cgi?rs=rs773561937) | [11:22297607](genomebrowse://api/zoom?locus=11:22297607) | - | 0.0305276 | 0.0180986 | 0.062 | intron_variant | - | - | - |
| *ANO6* | Anoctamin 6 | [rs7299561](http://www.ncbi.nlm.nih.gov/projects/SNP/snp_ref.cgi?rs=rs7299561) | [12:45695828](genomebrowse://api/zoom?locus=12:45695828) | 0.0447284 | 0.0117856 | 0.0429642 | 0.014 | synonymous_variant | - | - | - |
| *ANO6* | Anoctamin 6 | [rs36059278](http://www.ncbi.nlm.nih.gov/projects/SNP/snp_ref.cgi?rs=rs36059278) | [12:45771902](genomebrowse://api/zoom?locus=12:45771902) | - | 0.259503 | 0.0701034 | 0.33 | splice_region_variant | - | - | - |
| *ANO6* | Anoctamin 6 | [rs117316516](http://www.ncbi.nlm.nih.gov/projects/SNP/snp_ref.cgi?rs=rs117316516) | [12:45797209](genomebrowse://api/zoom?locus=12:45797209) | 0.0221645 | 0.0252863 | 0.014497 | 0.026 | intron_variant | - | - | - |
| *ANO7* | Anoctamin 7 | [rs74804606](http://www.ncbi.nlm.nih.gov/projects/SNP/snp_ref.cgi?rs=rs74804606) | [2:242157184](genomebrowse://api/zoom?locus=2:242157184) | 0.0561102 | 0.0127074 | 0.0486287 | 0.016 | missense_variant | Damaging | Possibly damaging | Tolerated |
| *ANPEP* | Alanyl Aminopeptidase, Membrane | rs115627096 | 15:90340872 | 0.00738818 | 0.0016446 | 0.0066658 | 0.002199 | synonymous_variant | - | - | - |
| *ANPEP* | Alanyl Aminopeptidase, Membrane | [rs41276922](http://www.ncbi.nlm.nih.gov/projects/SNP/snp_ref.cgi?rs=rs41276922) | [15:90349461](genomebrowse://api/zoom?locus=15:90349461) | 0.0425319 | 0.0606912 | 0.063858 | 0.062 | synonymous_variant | - | - | - |
| *ANTXR1* | ANTXR Cell Adhesion Molecule 1 | [rs747343458](http://www.ncbi.nlm.nih.gov/projects/SNP/snp_ref.cgi?rs=rs747343458) | [2:69420592](genomebrowse://api/zoom?locus=2:69420592) | - | - | 0.249434 | 0.202 | intron_variant | - | - | - |
| *ANTXRL* | ANTXR Like | [rs148597284](http://www.ncbi.nlm.nih.gov/projects/SNP/snp_ref.cgi?rs=rs148597284) | [10:47674009](genomebrowse://api/zoom?locus=10:47674009) | 0.0233626 | 0.0417873 | 0.0457339 | 0.046 | missense_variant | - | - | Tolerated |
| *ANXA11* | Annexin A11 | [rs2229554](http://www.ncbi.nlm.nih.gov/projects/SNP/snp_ref.cgi?rs=rs2229554) | [10:81927059](genomebrowse://api/zoom?locus=10:81927059) | 0.0219649 | 0.0486292 | 0.0422731 | 0.05 | missense_variant | Tolerated | Probably damaging | Damaging |
| *ANXA13* | Annexin A13 | [rs55688638](http://www.ncbi.nlm.nih.gov/projects/SNP/snp_ref.cgi?rs=rs55688638) | [8:124701193](genomebrowse://api/zoom?locus=8:124701193) | 0.0115815 | 0.0228643 | 0.020451 | 0.022 | splice_region_variant | - | - | - |
| *ANXA2* | Annexin A2 | [rs17845226](http://www.ncbi.nlm.nih.gov/projects/SNP/snp_ref.cgi?rs=rs17845226) | [15:60653205](genomebrowse://api/zoom?locus=15:60653205) | 0.0485224 | 0.0922939 | 0.0862737 | 0.094 | missense_variant | Tolerated | Benign | Damaging |
| *ANXA5* | Annexin A5 | rs147775952 | 4:122592742 | 0.00339457 | 0.00376938 | 0.00151838 | 0.003707 | synonymous_variant | - | - | - |
| *ANXA7* | Annexin A7 | [rs2234965](http://www.ncbi.nlm.nih.gov/projects/SNP/snp_ref.cgi?rs=rs2234965) | [10:75148116](genomebrowse://api/zoom?locus=10:75148116) | 0.176518 | 0.108457 | 0.102751 | 0.113 | synonymous_variant | - | - | - |
| *AOC3* | Amine Oxidase Copper Containing 3 | rs2229595 | 17:41004346 | 0.00339457 | 0.00652968 | 0.0063332 | 0.006474 | missense_variant | Tolerated | Benign | Tolerated |
| *AOC3* | Amine Oxidase Copper Containing 3 | [rs2228470](http://www.ncbi.nlm.nih.gov/projects/SNP/snp_ref.cgi?rs=rs2228470) | [17:41003859](genomebrowse://api/zoom?locus=17:41003859) | 0.0455272 | 0.0116397 | 0.0432602 | 0.014 | missense_variant | Tolerated | Benign | Tolerated |
| *AOC3* | Amine Oxidase Copper Containing 3 | [rs33916389](http://www.ncbi.nlm.nih.gov/projects/SNP/snp_ref.cgi?rs=rs33916389) | [17:41004503](genomebrowse://api/zoom?locus=17:41004503) | 0.0451278 | 0.0118003 | 0.0433152 | 0.015 | synonymous_variant | - | - | - |
| *AP000688.14* | - | [rs2835265](http://www.ncbi.nlm.nih.gov/projects/SNP/snp_ref.cgi?rs=rs2835265) | [21:37444696](genomebrowse://api/zoom?locus=21:37444696) | 0.126398 | 0.135264 | 0.104238 | 0.132 | 3_prime_UTR_variant | Damaging | - | Tolerated |
| *AP1AR* | Adaptor Related Protein Complex 1 Associated Regulatory Protein | [rs75171772](http://www.ncbi.nlm.nih.gov/projects/SNP/snp_ref.cgi?rs=rs75171772) | [4:113187731](genomebrowse://api/zoom?locus=4:113187731) | 0.01877 | 0.0354381 | 0.0317949 | 0.034 | intron_variant | - | - | - |
| *AP2A2* | Adaptor Related Protein Complex 2 Subunit Alpha 2 | [rs72842410](http://www.ncbi.nlm.nih.gov/projects/SNP/snp_ref.cgi?rs=rs72842410) | [11:1010649](genomebrowse://api/zoom?locus=11:1010649) | 0.166733 | 0.158169 | 0.0962645 | 0.131 | 3_prime_UTR_variant | Tolerated | - | Tolerated |
| *AP3M2* | Adaptor Related Protein Complex 3 Subunit Mu 2 | rs199664300 | 8:42012310 | 0.000399361 | 0.000158388 | 0.000129458 | 0.0001977 | synonymous_variant | - | - | - |
| *AP4B1* | Adaptor Related Protein Complex 4 Subunit Beta 1 | [rs34751342](http://www.ncbi.nlm.nih.gov/projects/SNP/snp_ref.cgi?rs=rs34751342) | [1:114444444](genomebrowse://api/zoom?locus=1:114444444) | 0.0215655 | 0.0364754 | 0.0298411 | 0.037 | synonymous_variant | - | - | - |
| *AP5M1* | Adaptor Related Protein Complex 5 Subunit Mu 1 | [rs35759976](http://www.ncbi.nlm.nih.gov/projects/SNP/snp_ref.cgi?rs=rs35759976) | [14:57755564](genomebrowse://api/zoom?locus=14:57755564) | 0.034345 | 0.063757 | 0.0503853 | 0.061 | missense_variant | Damaging | Benign | Damaging |
| *APBA2* | Amyloid Beta Precursor Protein Binding Family A Member 2 | rs111870710 | 15:29346705 | 0.00419329 | 0.000736347 | 0.00319912 | 0.0009968 | synonymous_variant | - | - | - |
| *APBA3* | Amyloid Beta Precursor Protein Binding Family A Member 3 | [rs147130540](http://www.ncbi.nlm.nih.gov/projects/SNP/snp_ref.cgi?rs=rs147130540) | [19:3752601](genomebrowse://api/zoom?locus=19:3752601) | 0.0113818 | 0.0170895 | 0.0154953 | 0.014 | missense_variant | Damaging | Probably damaging | Damaging |
| *APBB2* | Amyloid Beta Precursor Protein Binding Family B Member 2 | rs34875102 | 4:41015942 | 0.000199681 | 0.00165391 | 0.00171144 | 0.001581 | missense_variant | Tolerated | Probably damaging | Damaging |
| *APBB2* | Amyloid Beta Precursor Protein Binding Family B Member 2 | [rs11942640](http://www.ncbi.nlm.nih.gov/projects/SNP/snp_ref.cgi?rs=rs11942640) | [4:41015742](genomebrowse://api/zoom?locus=4:41015742) | 0.0461262 | 0.0194824 | 0.0431352 | 0.021 | synonymous_variant | - | - | - |
| *APOA4* | Apolipoprotein A4 | [rs5093](http://www.ncbi.nlm.nih.gov/projects/SNP/snp_ref.cgi?rs=rs5093) | [11:116693354](genomebrowse://api/zoom?locus=11:116693354) | 0.0634984 | 0.0497234 | 0.0372285 | 0.048 | intron_variant | - | - | - |
| *APOB* | Apolipoprotein B | [rs1801695](http://www.ncbi.nlm.nih.gov/projects/SNP/snp_ref.cgi?rs=rs1801695) | [2:21224853](genomebrowse://api/zoom?locus=2:21224853) | 0.0141773 | 0.025157 | 0.0210244 | 0.024 | missense_variant | Tolerated | Benign | Tolerated |
| *APOBEC1* | Apolipoprotein B MRNA Editing Enzyme Catalytic Subunit 1 | rs139646668 | 12:7818468 | 0.000998403 | 0.00155931 | 0.000873108 | 0.00154 | initiator_codon_variant | Damaging | Possibly damaging | Damaging |
| *APOBEC4* | Apolipoprotein B MRNA Editing Enzyme Catalytic Polypeptide Like 4 | rs147640407 | 1:183617288 | 0.00299521 | 0.00121439 | 0.00216352 | 0.001194 | missense_variant | Tolerated | Benign | Tolerated |
| *APOBEC4* | Apolipoprotein B MRNA Editing Enzyme Catalytic Polypeptide Like 4 | [rs10911391](http://www.ncbi.nlm.nih.gov/projects/SNP/snp_ref.cgi?rs=rs10911391) | [1:183617094](genomebrowse://api/zoom?locus=1:183617094) | 0.0944489 | 0.0427804 | 0.0672978 | 0.046 | missense_variant | Tolerated | Benign | Tolerated |
| *APOBEC4* | Apolipoprotein B MRNA Editing Enzyme Catalytic Polypeptide Like 4 | [rs10911392](http://www.ncbi.nlm.nih.gov/projects/SNP/snp_ref.cgi?rs=rs10911392) | [1:183617311](genomebrowse://api/zoom?locus=1:183617311) | 0.110823 | 0.0459851 | 0.0808515 | 0.05 | synonymous_variant | - | - | - |
| *APOH* | Apolipoprotein H | rs114459902 | 17:64216797 | 0.00579073 | 0.00145831 | 0.00549131 | 0.00187 | missense_variant | Tolerated | Benign | Tolerated |
| *APOH* | Apolipoprotein H | [rs8178847](http://www.ncbi.nlm.nih.gov/projects/SNP/snp_ref.cgi?rs=rs8178847) | [17:64216815](genomebrowse://api/zoom?locus=17:64216815) | 0.0702875 | 0.0639205 | 0.0782586 | 0.066 | missense_variant | Tolerated | Benign | Tolerated |
| *APOL5* | Apolipoprotein L5 | [rs4821441](http://www.ncbi.nlm.nih.gov/projects/SNP/snp_ref.cgi?rs=rs4821441) | [22:36122811](genomebrowse://api/zoom?locus=22:36122811) | 0.0519169 | 0.0492451 | 0.0531771 | 0.051 | synonymous_variant | - | - | - |
| *APTX* | Aprataxin | rs141195622 | 9:32984657 | 0.000998403 | 0.00136132 | 0.00122739 | 0.001384 | missense_variant | Damaging | Possibly damaging | Damaging |
| *APTX* | Aprataxin | [rs10813920](http://www.ncbi.nlm.nih.gov/projects/SNP/snp_ref.cgi?rs=rs10813920) | [9:33001441](genomebrowse://api/zoom?locus=9:33001441) | 0.160543 | 0.120242 | 0.126382 | 0.11 | 5_prime_UTR_variant | - | - | - |
| *AQP12A* | Aquaporin 12A | [rs11889147](http://www.ncbi.nlm.nih.gov/projects/SNP/snp_ref.cgi?rs=rs11889147) | [2:241631616](genomebrowse://api/zoom?locus=2:241631616) | 0.0808706 | 0.0173548 | 0.044516 | 0.021 | synonymous_variant | - | - | - |
| *AQP12A* | Aquaporin 12A | [rs12465023](http://www.ncbi.nlm.nih.gov/projects/SNP/snp_ref.cgi?rs=rs12465023) | [2:241631886](genomebrowse://api/zoom?locus=2:241631886) | - | 0.276475 | 0.267706 | 0.171 | synonymous_variant | - | - | - |
| *AQP12A* | Aquaporin 12A | [rs58669952](http://www.ncbi.nlm.nih.gov/projects/SNP/snp_ref.cgi?rs=rs58669952) | [2:241631901](genomebrowse://api/zoom?locus=2:241631901) | - | 0.276336 | 0.264378 | 0.168 | synonymous_variant | - | - | - |
| *AQP12B* | Aquaporin 12B | [rs184125501](http://www.ncbi.nlm.nih.gov/projects/SNP/snp_ref.cgi?rs=rs184125501) | [2:241622034](genomebrowse://api/zoom?locus=2:241622034) | 0.0265575 | 0.0574743 | 0.0664892 | 0.056 | missense_variant | Damaging | Probably damaging | Damaging |
| *AQP4-AS1* | AQP4 Antisense RNA 1 | [rs17694469](http://www.ncbi.nlm.nih.gov/projects/SNP/snp_ref.cgi?rs=rs17694469) | [18:24497190](genomebrowse://api/zoom?locus=18:24497190) | 0.0113818 | 0.0181682 | 0.0144169 | 0.019 | missense_variant | - | Benign | Tolerated |
| *AQP8* | Aquaporin 8 | [rs111840156](http://www.ncbi.nlm.nih.gov/projects/SNP/snp_ref.cgi?rs=rs111840156) | [16:25239809](genomebrowse://api/zoom?locus=16:25239809) | 0.0159744 | 0.0234029 | 0.0205719 | 0.023 | missense_variant | Tolerated | Benign | Tolerated |
| *AQP9* | Aquaporin 9 | rs140188159 | 15:58467196 | 0.00339457 | 0.0044587 | 0.00100052 | 0.003187 | synonymous_variant | - | - | - |
| *ARAP2* | ArfGAP With RhoGAP Domain, Ankyrin Repeat And PH Domain 2 | [rs13148785](http://www.ncbi.nlm.nih.gov/projects/SNP/snp_ref.cgi?rs=rs13148785) | [4:36152584](genomebrowse://api/zoom?locus=4:36152584) | 0.0549121 | 0.0729892 | 0.0877618 | 0.074 | synonymous_variant | - | - | - |
| *ARAP3* | ArfGAP With RhoGAP Domain, Ankyrin Repeat And PH Domain 3 | [rs2306340](http://www.ncbi.nlm.nih.gov/projects/SNP/snp_ref.cgi?rs=rs2306340) | [5:141035248](genomebrowse://api/zoom?locus=5:141035248) | 0.0696885 | 0.0675184 | 0.0609843 | 0.068 | synonymous_variant | - | - | - |
| *AREG* | Amphiregulin | [rs62314511](http://www.ncbi.nlm.nih.gov/projects/SNP/snp_ref.cgi?rs=rs62314511) | [4:75316206](genomebrowse://api/zoom?locus=4:75316206) | - | 0.0947423 | 0.0964912 | 0.085 | missense_variant | Tolerated | Benign | Tolerated |
| *ARFGEF2* | ADP Ribosylation Factor Guanine Nucleotide Exchange Factor 2 | rs146305858 | 20:47641829 | 0.00319489 | 0.0011091 | 0.00487474 | 0.001401 | intron_variant | - | - | - |
| *ARFGEF2* | ADP Ribosylation Factor Guanine Nucleotide Exchange Factor 2 | [rs41304647](http://www.ncbi.nlm.nih.gov/projects/SNP/snp_ref.cgi?rs=rs41304647) | [20:47569226](genomebrowse://api/zoom?locus=20:47569226) | 0.0539137 | 0.0599438 | 0.0457833 | 0.061 | intron_variant | - | - | - |
| *ARFGEF2* | ADP Ribosylation Factor Guanine Nucleotide Exchange Factor 2 | c.2070+10G>A | [20:47601387](genomebrowse://api/zoom?locus=20:47601387) | - | - | - | - | intron_variant | - | - | - |
| *ARG1* | Arginase 1 | [rs41285336](http://www.ncbi.nlm.nih.gov/projects/SNP/snp_ref.cgi?rs=rs41285336) | [6:131902329](genomebrowse://api/zoom?locus=6:131902329) | 0.0227636 | 0.0526536 | 0.0563703 | 0.052 | intron_variant | - | - | - |
| *ARHGAP15* | Rho GTPase Activating Protein 15 | [rs12621011](http://www.ncbi.nlm.nih.gov/projects/SNP/snp_ref.cgi?rs=rs12621011) | [2:144193293](genomebrowse://api/zoom?locus=2:144193293) | 0.115615 | 0.0964821 | 0.040121 | 0.087 | intron_variant | - | - | - |
| *ARHGAP24* | Rho GTPase Activating Protein 24 | [rs346518](http://www.ncbi.nlm.nih.gov/projects/SNP/snp_ref.cgi?rs=rs346518) | [4:86844948](genomebrowse://api/zoom?locus=4:86844948) | 0.0734824 | 0.105249 | 0.0915115 | 0.107 | intron_variant | - | - | - |
| *ARHGAP32* | Rho GTPase Activating Protein 32 | [rs78856194](http://www.ncbi.nlm.nih.gov/projects/SNP/snp_ref.cgi?rs=rs78856194) | [11:128839405](genomebrowse://api/zoom?locus=11:128839405) | 0.0147764 | 0.0334476 | 0.0391181 | 0.032 | synonymous_variant | - | - | - |
| *ARHGAP39* | Rho GTPase Activating Protein 39 | [rs61734975](http://www.ncbi.nlm.nih.gov/projects/SNP/snp_ref.cgi?rs=rs61734975) | [8:145830988](genomebrowse://api/zoom?locus=8:145830988) | 0.0313498 | 0.0380584 | 0.0231416 | 0.035 | synonymous_variant | - | - | - |
| *ARHGAP42* | Rho GTPase Activating Protein 42 | rs187096137 | 11:100792211 | 0.00319489 | 0.000679665 | 0.00298547 | 0.001298 | intron_variant | - | - | - |
| *ARHGAP8* | Rho GTPase Activating Protein 8 | [rs41278891](http://www.ncbi.nlm.nih.gov/projects/SNP/snp_ref.cgi?rs=rs41278891) | [22:45258373](genomebrowse://api/zoom?locus=22:45258373) | 0.0113818 | 0.0247834 | 0.022598 | 0.026 | missense_variant | Tolerated | Benign | Tolerated |
| *ARHGEF10* | Rho Guanine Nucleotide Exchange Factor 10 | [rs2280886](http://www.ncbi.nlm.nih.gov/projects/SNP/snp_ref.cgi?rs=rs2280886) | [8:1830794](genomebrowse://api/zoom?locus=8:1830794) | 0.167532 | 0.160459 | 0.115772 | 0.078 | splice_region_variant | - | - | - |
| *ARHGEF10* | Rho Guanine Nucleotide Exchange Factor 10 | [rs9657362](http://www.ncbi.nlm.nih.gov/projects/SNP/snp_ref.cgi?rs=rs9657362) | [8:1833801](genomebrowse://api/zoom?locus=8:1833801) | 0.168331 | 0.159511 | 0.117596 | 0.159 | missense_variant | Tolerated | Possibly damaging | Tolerated |
| *ARHGEF10* | Rho Guanine Nucleotide Exchange Factor 10 | [rs34655804](http://www.ncbi.nlm.nih.gov/projects/SNP/snp_ref.cgi?rs=rs34655804) | [8:1833804](genomebrowse://api/zoom?locus=8:1833804) | 0.0459265 | 0.0114085 | 0.0427046 | 0.015 | synonymous_variant | - | - | - |
| *ARHGEF10* | Rho Guanine Nucleotide Exchange Factor 10 | [rs112914148](http://www.ncbi.nlm.nih.gov/projects/SNP/snp_ref.cgi?rs=rs112914148) | [8:1871114](genomebrowse://api/zoom?locus=8:1871114) | 0.0341454 | 0.0615872 | 0.0617611 | 0.062 | intron_variant | - | - | - |
| *ARHGEF12* | Rho Guanine Nucleotide Exchange Factor 12 | [rs34172482](http://www.ncbi.nlm.nih.gov/projects/SNP/snp_ref.cgi?rs=rs34172482) | [11:120278477](genomebrowse://api/zoom?locus=11:120278477) | 0.0139776 | 0.0287049 | 0.0296492 | 0.028 | synonymous_variant | - | - | - |
| *ARHGEF16* | Rho Guanine Nucleotide Exchange Factor 16 | rs56309807 | 1:3397062 | 0.0191693 | 0.018467 | 0.023631 | 0.018 | missense_variant | Tolerated | Benign | Damaging |
| *ARHGEF17* | Rho Guanine Nucleotide Exchange Factor 17 | [rs76764824](http://www.ncbi.nlm.nih.gov/projects/SNP/snp_ref.cgi?rs=rs76764824) | [11:73073580](genomebrowse://api/zoom?locus=11:73073580) | 0.0477236 | 0.0793447 | 0.0869467 | 0.08 | synonymous_variant | - | - | - |
| *ARHGEF34P* | Rho Guanine Nucleotide Exchange Factor 34, Pseudogene | [rs199675416](http://www.ncbi.nlm.nih.gov/projects/SNP/snp_ref.cgi?rs=rs199675416) | [7:143964428](genomebrowse://api/zoom?locus=7:143964428) | - | 0.0895147 | 0.0601194 | - | missense_variant | Damaging | Probably damaging | Tolerated |
| *ARHGEF34P* | Rho Guanine Nucleotide Exchange Factor 34, Pseudogene | [rs201948019](http://www.ncbi.nlm.nih.gov/projects/SNP/snp_ref.cgi?rs=rs201948019) | [7:143956181](genomebrowse://api/zoom?locus=7:143956181) | - | 0.0130671 | 0.0267962 | 0.024 | missense_variant | Tolerated | Benign | Tolerated |
| *ARHGEF39* | Rho Guanine Nucleotide Exchange Factor 39 | [rs10441685](http://www.ncbi.nlm.nih.gov/projects/SNP/snp_ref.cgi?rs=rs10441685) | [9:35661057](genomebrowse://api/zoom?locus=9:35661057) | 0.0714856 | 0.0319224 | 0.0622173 | 0.036 | missense_variant | Damaging | Possibly damaging | Tolerated |
| *ARHGEF5* | Rho Guanine Nucleotide Exchange Factor 5 | [rs142720995](http://www.ncbi.nlm.nih.gov/projects/SNP/snp_ref.cgi?rs=rs142720995) | [7:144061117](genomebrowse://api/zoom?locus=7:144061117) | - | 0.0269327 | 0.0311992 | 0.015 | missense_variant | Tolerated | Benign | Tolerated |
| *ARHGEF7* | Rho Guanine Nucleotide Exchange Factor 7 | rs146121341 | 13:111932967 | 0.000599042 | 0.00302807 | 0.00313348 | 0.003336 | synonymous_variant | - | - | - |
| *ARL6IP5* | ADP Ribosylation Factor Like GTPase 6 Interacting Protein 5 | [rs10489](http://www.ncbi.nlm.nih.gov/projects/SNP/snp_ref.cgi?rs=rs10489) | [3:69151152](genomebrowse://api/zoom?locus=3:69151152) | 0.165335 | 0.0825394 | 0.0967242 | 0.086 | synonymous_variant | - | - | - |
| *ARMC4* | Armadillo Repeat Containing 4 | [rs3737184](http://www.ncbi.nlm.nih.gov/projects/SNP/snp_ref.cgi?rs=rs3737184) | [10:28101455](genomebrowse://api/zoom?locus=10:28101455) | 0.0764776 | 0.0504114 | 0.0238726 | 0.048 | missense_variant | Tolerated | Benign | Damaging |
| *ARMC4* | Armadillo Repeat Containing 4 | [rs7920186](http://www.ncbi.nlm.nih.gov/projects/SNP/snp_ref.cgi?rs=rs7920186) | [10:28257852](genomebrowse://api/zoom?locus=10:28257852) | 0.111222 | 0.0982821 | 0.122096 | 0.056 | missense_variant | Tolerated | Benign | Tolerated |
| *ARNT2* | Aryl Hydrocarbon Receptor Nuclear Translocator 2 | [rs76861207](http://www.ncbi.nlm.nih.gov/projects/SNP/snp_ref.cgi?rs=rs76861207) | [15:80889548](genomebrowse://api/zoom?locus=15:80889548) | 0.0766773 | 0.0322042 | 0.0509742 | 0.03 | 3_prime_UTR_variant | - | - | Tolerated |
| *ARNTL* | Aryl Hydrocarbon Receptor Nuclear Translocator Like | [rs62620045](http://www.ncbi.nlm.nih.gov/projects/SNP/snp_ref.cgi?rs=rs62620045) | [11:13378457](genomebrowse://api/zoom?locus=11:13378457) | 0.0477236 | 0.0402086 | 0.0325602 | 0.036 | intron_variant | Damaging | Benign | Tolerated |
| *ARPC2* | Actin Related Protein 2/3 Complex Subunit 2 | rs571789910 | 2:219114050 | 0.000199681 | 0.000146739 | 9.68992e-05 | 0.0001647 | intron_variant | - | - | - |
| *ARPC3* | Actin Related Protein 2/3 Complex Subunit 3 | [rs766067249](http://www.ncbi.nlm.nih.gov/projects/SNP/snp_ref.cgi?rs=rs766067249) | [12:110874502](genomebrowse://api/zoom?locus=12:110874502) | - | 0.0228212 | 0.0266017 | 0.035 | intron_variant | - | - | - |
| *ARPC4-TTLL3* | ARPC4-TTLL3 Readthrough | [rs115917139](http://www.ncbi.nlm.nih.gov/projects/SNP/snp_ref.cgi?rs=rs115917139) | [3:9874914](genomebrowse://api/zoom?locus=3:9874914) | 0.0229633 | 0.0373009 | 0.0268723 | 0.037 | stop_gained | - | - | Damaging |
| *ARPC5* | Actin Related Protein 2/3 Complex Subunit 5 | [rs2231241](http://www.ncbi.nlm.nih.gov/projects/SNP/snp_ref.cgi?rs=rs2231241) | [1:183596703](genomebrowse://api/zoom?locus=1:183596703) | 0.100639 | 0.0438851 | 0.0731132 | 0.048 | synonymous_variant | - | - | - |
| *ARRDC4* | Arrestin Domain Containing 4 | [rs61741967](http://www.ncbi.nlm.nih.gov/projects/SNP/snp_ref.cgi?rs=rs61741967) | [15:98513169](genomebrowse://api/zoom?locus=15:98513169) | 0.0495208 | 0.0155062 | 0.0429528 | 0.018 | synonymous_variant | - | - | - |
| *ARVCF* | ARVCF Delta Catenin Family Member | [rs74544696](http://www.ncbi.nlm.nih.gov/projects/SNP/snp_ref.cgi?rs=rs74544696) | [22:19967480](genomebrowse://api/zoom?locus=22:19967480) | 0.0363419 | 0.0242355 | 0.0250243 | 0.022 | synonymous_variant | - | - | - |
| *ASAH1* | N-Acylsphingosine Amidohydrolase 1 | [rs2472205](http://www.ncbi.nlm.nih.gov/projects/SNP/snp_ref.cgi?rs=rs2472205) | [8:17924739](genomebrowse://api/zoom?locus=8:17924739) | 0.0227636 | 0.0392419 | 0.0297635 | 0.041 | missense_variant | Tolerated | Benign | Damaging |
| *ASB14* | Ankyrin Repeat And SOCS Box Containing 14 | rs61747702 | 3:57317300 | 0.00658946 | 0.00153465 | 0.00613537 | 0.002859 | missense_variant | Damaging | Probably damaging | Tolerated |
| *ASB2* | Ankyrin Repeat And SOCS Box Containing 2 | rs77273556 | 14:94417479 | 0.00698882 | 0.00187639 | 0.00733015 | 0.002265 | missense_variant | Tolerated | Probably damaging | Damaging |
| *ASCC1* | Activating Signal Cointegrator 1 Complex Subunit 1 | [rs41282254](http://www.ncbi.nlm.nih.gov/projects/SNP/snp_ref.cgi?rs=rs41282254) | [10:73963170](genomebrowse://api/zoom?locus=10:73963170) | 0.0177716 | 0.0239825 | 0.0215384 | 0.025 | intron_variant | - | - | - |
| *ASCC3* | Activating Signal Cointegrator 1 Complex Subunit 3 | rs77182320 | 6:101110420 | 0.00379393 | 0.00579968 | 0.00417395 | 0.005224 | splice_region_variant | - | - | - |
| *ASCC3* | Activating Signal Cointegrator 1 Complex Subunit 3 | [rs79637308](http://www.ncbi.nlm.nih.gov/projects/SNP/snp_ref.cgi?rs=rs79637308) | [6:101078974](genomebrowse://api/zoom?locus=6:101078974) | 0.0115815 | 0.0260558 | 0.0248351 | 0.024 | intron_variant | - | - | - |
| *ASIC3* | Acid Sensing Ion Channel Subunit 3 | rs114024820 | 7:150747896 | 0.00379393 | 0.00876241 | 0.00653722 | 0.008888 | missense_variant | Tolerated | Benign | Damaging |
| *ASMTL* | Acetylserotonin O-Methyltransferase Like | [rs79968027](http://www.ncbi.nlm.nih.gov/projects/SNP/snp_ref.cgi?rs=rs79968027) | [X:1531687](genomebrowse://api/zoom?locus=X:1531687) | 0.013778 | 0.0515269 | 0.0557744 | 0.055 | missense_variant | Damaging | Probably damaging | Damaging |
| *ASPHD2* | Aspartate Beta-Hydroxylase Domain Containing 2 | [rs34902186](http://www.ncbi.nlm.nih.gov/projects/SNP/snp_ref.cgi?rs=rs34902186) | [22:26830285](genomebrowse://api/zoom?locus=22:26830285) | 0.0263578 | 0.0606683 | 0.0591617 | 0.061 | missense_variant | Tolerated | Benign | Damaging |
| *ASPM* | Abnormal Spindle Microtubule Assembly | rs112647911 | 1:197070464 | 0.00978434 | 0.0030369 | 0.00855088 | 0.003282 | synonymous_variant | - | - | - |
| *ASPM* | Abnormal Spindle Microtubule Assembly | rs111487086 | 1:197071028 | 0.00978434 | 0.00302751 | 0.0085207 | 0.003287 | synonymous_variant | - | - | - |
| *ASPM* | Abnormal Spindle Microtubule Assembly | rs113325473 | 1:197072640 | 0.00479233 | 0.000893265 | 0.00385338 | 0.001128 | missense_variant | Tolerated | Benign | Tolerated |
| *ASPM* | Abnormal Spindle Microtubule Assembly | rs41299625 | 1:197072929 | 0.00159744 | 0.00114095 | 0.000777152 | 0.00131 | missense_variant | Damaging | Probably damaging | Damaging |
| *ASPM* | Abnormal Spindle Microtubule Assembly | c.10332-6delT | [1:197053562](genomebrowse://api/zoom?locus=1:197053562) | - | 0.206156 | 0.0105118 | 0.177 | splice_region_variant | - | - | - |
| *ASPM* | Abnormal Spindle Microtubule Assembly | [rs12138336](http://www.ncbi.nlm.nih.gov/projects/SNP/snp_ref.cgi?rs=rs12138336) | [1:197070521](genomebrowse://api/zoom?locus=1:197070521) | 0.028754 | 0.0508133 | 0.0464476 | 0.05 | missense_variant | Tolerated | Benign | Tolerated |
| *ASS1* | Argininosuccinate Synthase 1 | [rs1057484](http://www.ncbi.nlm.nih.gov/projects/SNP/snp_ref.cgi?rs=rs1057484) | [9:133364757](genomebrowse://api/zoom?locus=9:133364757) | 0.0766773 | 0.0921777 | 0.113166 | 0.094 | synonymous_variant | - | - | - |
| *ATAD2B* | ATPase Family AAA Domain Containing 2B | [rs2712089](http://www.ncbi.nlm.nih.gov/projects/SNP/snp_ref.cgi?rs=rs2712089) | [2:24080274](genomebrowse://api/zoom?locus=2:24080274) | 0.0680911 | 0.0434029 | 0.0630529 | 0.037 | intron_variant | - | - | - |
| *ATAD3A* | ATPase Family AAA Domain Containing 3A | rs139994378 | 1:1452641 | 0.00838658 | 0.00162481 | 0.0078094 | 0.001672 | missense_variant | Tolerated | Benign | Tolerated |
| *ATAD3A* | ATPase Family AAA Domain Containing 3A | rs113640550 | 1:1459323 | 0.00758786 | 0.0017638 | 0.0072431 | 0.002356 | synonymous_variant | - | - | - |
| *ATAD5* | ATPase Family AAA Domain Containing 5 | [rs11655623](http://www.ncbi.nlm.nih.gov/projects/SNP/snp_ref.cgi?rs=rs11655623) | [17:29162173](genomebrowse://api/zoom?locus=17:29162173) | 0.180112 | 0.138639 | 0.129202 | 0.141 | synonymous_variant | - | - | - |
| *ATAD5* | ATPase Family AAA Domain Containing 5 | [rs9896095](http://www.ncbi.nlm.nih.gov/projects/SNP/snp_ref.cgi?rs=rs9896095) | [17:29187497](genomebrowse://api/zoom?locus=17:29187497) | 0.172524 | 0.134558 | 0.124491 | 0.14 | synonymous_variant | - | - | - |
| *ATAD5* | ATPase Family AAA Domain Containing 5 | [rs11657270](http://www.ncbi.nlm.nih.gov/projects/SNP/snp_ref.cgi?rs=rs11657270) | [17:29214387](genomebrowse://api/zoom?locus=17:29214387) | 0.144369 | 0.126081 | 0.0975846 | 0.131 | missense_variant | Tolerated | Benign | Tolerated |
| *ATCAY* | ATCAY Kinesin Light Chain Interacting Caytaxin | [rs61551618](http://www.ncbi.nlm.nih.gov/projects/SNP/snp_ref.cgi?rs=rs61551618) | [19:3924517](genomebrowse://api/zoom?locus=19:3924517) | 0.0329473 | - | 0.0264843 | - | intron_variant | - | - | - |
| *ATG12* | Autophagy Related 12 | rs149148380 | 5:115177382 | 0.000998403 | 0.000426628 | 0.00216366 | 0.0006095 | 5_prime_UTR_variant | - | - | - |
| *ATG14* | Autophagy Related 14 | [rs61743178](http://www.ncbi.nlm.nih.gov/projects/SNP/snp_ref.cgi?rs=rs61743178) | [14:55864146](genomebrowse://api/zoom?locus=14:55864146) | 0.0249601 | 0.0409238 | 0.0303197 | 0.042 | synonymous_variant | - | - | - |
| *ATG4C* | Autophagy Related 4C Cysteine Peptidase | [rs17123872](http://www.ncbi.nlm.nih.gov/projects/SNP/snp_ref.cgi?rs=rs17123872) | [1:63284830](genomebrowse://api/zoom?locus=1:63284830) | 0.0265575 | 0.0474442 | 0.0249273 | 0.042 | synonymous_variant | - | - | - |
| *ATIC* | 5-Aminoimidazole-4-Carboxamide Ribonucleotide Formyltransferase/IMP Cyclohydrolase | rs79239006 | 2:216214029 | 0.00519169 | 0.00137354 | 0.00513632 | 0.001664 | intron_variant | - | - | - |
| *ATL1* | Atlastin GTPase 1 | rs139720661 | 14:51080039 | 0.00998403 | 0.00187418 | 0.00784833 | 0.002364 | synonymous_variant | - | - | - |
| *ATMIN* | ATM Interactor | [rs36040615](http://www.ncbi.nlm.nih.gov/projects/SNP/snp_ref.cgi?rs=rs36040615) | [16:81078081](genomebrowse://api/zoom?locus=16:81078081) | 0.0305511 | 0.0139443 | 0.0233204 | 0.014 | missense_variant | Tolerated | Benign | Tolerated |
| *ATP13A5* | ATPase 13A5 | rs201387347 | 3:193081090 | 0.000798722 | 0.0022173 | 0.00190421 | 0.002487 | missense_variant | Tolerated | Benign | Tolerated |
| *ATP1A1* | ATPase Na+/K+ Transporting Subunit Alpha 1 | [rs11540956](http://www.ncbi.nlm.nih.gov/projects/SNP/snp_ref.cgi?rs=rs11540956) | [1:116932921](genomebrowse://api/zoom?locus=1:116932921) | 0.0810703 | 0.0350191 | 0.0758096 | 0.039 | synonymous_variant | - | - | - |
| *ATP1A2* | ATPase Na+/K+ Transporting Subunit Alpha 2 | [rs2295623](http://www.ncbi.nlm.nih.gov/projects/SNP/snp_ref.cgi?rs=rs2295623) | [1:160097315](genomebrowse://api/zoom?locus=1:160097315) | 0.0814696 | 0.0932897 | 0.0870605 | 0.092 | intron_variant | - | - | - |
| *ATP2A2* | ATPase Sarcoplasmic/Endoplasmic Reticulum Ca2+ Transporting 2 | rs191476843 | 12:110764149 | 0.000599042 | 0.000224901 | 0.000129224 | 0.0001813 | intron_variant | - | - | - |
| *ATP2A3* | ATPase Sarcoplasmic/Endoplasmic Reticulum Ca2+ Transporting 3 | rs145977939 | 17:3845941 | 0.000199681 | 0.000183414 | 9.70371e-05 | 0.0002306 | missense_variant | Damaging | Benign | Damaging |
| *ATP2A3* | ATPase Sarcoplasmic/Endoplasmic Reticulum Ca2+ Transporting 3 | [rs55837933](http://www.ncbi.nlm.nih.gov/projects/SNP/snp_ref.cgi?rs=rs55837933) | [17:3850910](genomebrowse://api/zoom?locus=17:3850910) | 0.0710863 | 0.016785 | 0.0696697 | 0.022 | synonymous_variant | - | - | - |
| *ATP2B1* | ATPase Plasma Membrane Ca2+ Transporting 1 | rs35349730 | 12:89998019 | 0.00778754 | 0.00847768 | 0.00707593 | 0.008253 | synonymous_variant | - | - | - |
| *ATP2B2* | ATPase Plasma Membrane Ca2+ Transporting 2 | [rs9826066](http://www.ncbi.nlm.nih.gov/projects/SNP/snp_ref.cgi?rs=rs9826066) | [3:10491230](genomebrowse://api/zoom?locus=3:10491230) | 0.0623003 | 0.0148991 | 0.0550672 | 0.018 | 5_prime_UTR_variant | - | - | - |
| *ATP2B4* | ATPase Plasma Membrane Ca2+ Transporting 4 | [rs41264257](http://www.ncbi.nlm.nih.gov/projects/SNP/snp_ref.cgi?rs=rs41264257) | [1:203708772](genomebrowse://api/zoom?locus=1:203708772) | 0.0189696 | 0.041014 | 0.0386578 | 0.041 | synonymous_variant | - | - | - |
| *ATP5J2-PTCD1* | ATP5J2-PTCD1 Readthrough | rs150504114 | 7:99032559 | 0.0071885 | 0.00954237 | 0.00552183 | 0.009274 | missense_variant | Damaging | Possibly damaging | Tolerated |
| *ATP5J2-PTCD1* | ATP5J2-PTCD1 Readthrough | [rs114658645](http://www.ncbi.nlm.nih.gov/projects/SNP/snp_ref.cgi?rs=rs114658645) | [7:99017737](genomebrowse://api/zoom?locus=7:99017737) | 0.033147 | 0.0459951 | 0.0369735 | 0.045 | synonymous_variant | - | - | - |
| *ATP5J2-PTCD1* | ATP5J2-PTCD1 Readthrough | [rs2240385](http://www.ncbi.nlm.nih.gov/projects/SNP/snp_ref.cgi?rs=rs2240385) | [7:99026837](genomebrowse://api/zoom?locus=7:99026837) | 0.165535 | 0.0887386 | 0.063491 | 0.09 | intron_variant | - | - | - |
| *ATP5J2-PTCD1* | ATP5J2-PTCD1 Readthrough | [rs940336](http://www.ncbi.nlm.nih.gov/projects/SNP/snp_ref.cgi?rs=rs940336) | [7:99032458](genomebrowse://api/zoom?locus=7:99032458) | 0.159744 | 0.087399 | 0.0588711 | 0.088 | synonymous_variant | - | - | - |
| *ATP5J2-PTCD1* | ATP5J2-PTCD1 Readthrough | [rs34943973](http://www.ncbi.nlm.nih.gov/projects/SNP/snp_ref.cgi?rs=rs34943973) | [7:99032517](genomebrowse://api/zoom?locus=7:99032517) | 0.0427316 | 0.046717 | 0.0443504 | 0.047 | missense_variant | Damaging | Benign | Tolerated |
| *ATP5O* | ATP Synthase Peripheral Stalk Subunit OSCP | [rs78234759](http://www.ncbi.nlm.nih.gov/projects/SNP/snp_ref.cgi?rs=rs78234759) | [21:35284683](genomebrowse://api/zoom?locus=21:35284683) | 0.0305511 | 0.0400906 | 0.0326656 | 0.04 | synonymous_variant | - | - | - |
| *ATP6V0A2* | ATPase H+ Transporting V0 Subunit A2 | rs146967928 | 12:124228362 | 0.00119808 | 0.000203069 | 0.000678207 | 0.0002141 | missense_variant | Tolerated | Benign | Damaging |
| *ATP6V0A2* | ATPase H+ Transporting V0 Subunit A2 | [rs74922060](http://www.ncbi.nlm.nih.gov/projects/SNP/snp_ref.cgi?rs=rs74922060) | [12:124221796](genomebrowse://api/zoom?locus=12:124221796) | 0.0265575 | 0.0116612 | 0.0182828 | 0.011 | missense_variant | Damaging | Probably damaging | Damaging |
| *ATP6V0A2* | ATPase H+ Transporting V0 Subunit A2 | [rs17883456](http://www.ncbi.nlm.nih.gov/projects/SNP/snp_ref.cgi?rs=rs17883456) | [12:124241506](genomebrowse://api/zoom?locus=12:124241506) | 0.0147764 | 0.0284167 | 0.0294156 | 0.028 | missense_variant | Damaging | Probably damaging | Damaging |
| *ATP6V0A4* | ATPase H+ Transporting V0 Subunit A4 | rs138627775 | 7:138394395 | 0.000599042 | 0.00130433 | 0.00129383 | 0.001392 | synonymous_variant | - | - | - |
| *ATP6V0A4* | ATPase H+ Transporting V0 Subunit A4 | [rs3807153](http://www.ncbi.nlm.nih.gov/projects/SNP/snp_ref.cgi?rs=rs3807153) | [7:138417791](genomebrowse://api/zoom?locus=7:138417791) | 0.10603 | 0.0654627 | 0.0877193 | 0.069 | missense_variant | Damaging | Benign | Damaging |
| *ATP6V0A4* | ATPase H+ Transporting V0 Subunit A4 | [rs58568563](http://www.ncbi.nlm.nih.gov/projects/SNP/snp_ref.cgi?rs=rs58568563) | [7:138433937](genomebrowse://api/zoom?locus=7:138433937) | 0.0690895 | 0.0212057 | 0.0518202 | 0.024 | synonymous_variant | - | - | - |
| *ATP6V1B1* | ATPase H+ Transporting V1 Subunit B1 | [rs17720303](http://www.ncbi.nlm.nih.gov/projects/SNP/snp_ref.cgi?rs=rs17720303) | [2:71163173](genomebrowse://api/zoom?locus=2:71163173) | 0.158546 | 0.184959 | 0.166526 | 0.18 | missense_variant | Tolerated | Benign | Damaging |
| *ATP7B* | ATPase Copper Transporting Beta | rs181250704 | 13:52509155 | 0.00119808 | 0.00107515 | 0.000971062 | 0.001083 | missense_variant | Damaging | Probably damaging | Damaging |
| *ATP7B* | ATPase Copper Transporting Beta | [rs7334118](http://www.ncbi.nlm.nih.gov/projects/SNP/snp_ref.cgi?rs=rs7334118) | [13:52513266](genomebrowse://api/zoom?locus=13:52513266) | 0.0315495 | 0.0294672 | 0.0204971 | 0.026 | missense_variant | Damaging | Possibly damaging | Damaging |
| *ATP7B* | ATPase Copper Transporting Beta | [rs7325983](http://www.ncbi.nlm.nih.gov/projects/SNP/snp_ref.cgi?rs=rs7325983) | [13:52520627](genomebrowse://api/zoom?locus=13:52520627) | 0.113618 | 0.0692928 | 0.0999289 | 0.07 | intron_variant | - | - | - |
| *ATP9B* | ATPase Phospholipid Transporting 9B (Putative) | [rs34331564](http://www.ncbi.nlm.nih.gov/projects/SNP/snp_ref.cgi?rs=rs34331564) | [18:76873288](genomebrowse://api/zoom?locus=18:76873288) | 0.067492 | 0.084223 | 0.0773952 | 0.085 | synonymous_variant | - | - | - |
| *ATPAF1* | ATP Synthase Mitochondrial F1 Complex Assembly Factor 1 | c.496-15A>T | [1:47123876](genomebrowse://api/zoom?locus=1:47123876) | - | - | - | - | intron_variant | - | - | - |
| *ATPAF2* | ATP Synthase Mitochondrial F1 Complex Assembly Factor 2 | [rs80251201](http://www.ncbi.nlm.nih.gov/projects/SNP/snp_ref.cgi?rs=rs80251201) | [17:17932008](genomebrowse://api/zoom?locus=17:17932008) | 0.141773 | 0.131211 | 0.0925866 | 0.134 | intron_variant | - | - | - |
| *ATXN7L1* | Ataxin 7 Like 1 | rs576111136 | 7:105305631 | 0.000199681 | 4.62419e-05 | 3.22997e-05 | 3.893e-05 | missense_variant | Tolerated | Benign | Damaging |
| *AUP1* | AUP1 Lipid Droplet Regulating VLDL Assembly Factor | [rs1183739](http://www.ncbi.nlm.nih.gov/projects/SNP/snp_ref.cgi?rs=rs1183739) | [2:74756548](genomebrowse://api/zoom?locus=2:74756548) | 0.0467252 | 0.113418 | 0.125016 | 0.098 | synonymous_variant | - | - | - |
| *AURKC* | Aurora Kinase C | rs113118234 | 19:57744819 | 0.00698882 | 0.00139698 | 0.00487632 | 0.001598 | intron_variant | - | - | - |
| *AVPR1A* | Arginine Vasopressin Receptor 1A | [rs2228153](http://www.ncbi.nlm.nih.gov/projects/SNP/snp_ref.cgi?rs=rs2228153) | [12:63544326](genomebrowse://api/zoom?locus=12:63544326) | 0.0593051 | 0.0132584 | 0.0524191 | 0.017 | synonymous_variant | - | - | - |
| *AXDND1* | Axonemal Dynein Light Chain Domain Containing 1 | rs5005771 | 1:179520569 | 0.00559105 | 0.00122184 | 0.00552183 | 0.001532 | synonymous_variant | - | - | - |
| *AXIN2* | Axin 2 | rs141697521 | 17:63534420 | 0.00139776 | 0.000809344 | 0.000517364 | 0.0007166 | synonymous_variant | - | - | - |
| *AZI1* | 5-Azacytidine-Induced Protein 1 | [rs4613097](http://www.ncbi.nlm.nih.gov/projects/SNP/snp_ref.cgi?rs=rs4613097) | [17:79170578](genomebrowse://api/zoom?locus=17:79170578) | 0.111422 | 0.0654858 | 0.0803571 | 0.062 | missense_variant | Tolerated | Benign | Damaging |
| *B3GALNT1* | Beta-1,3-N-Acetylgalactosaminyltransferase 1 (Globoside Blood Group) | [rs2231257](http://www.ncbi.nlm.nih.gov/projects/SNP/snp_ref.cgi?rs=rs2231257) | [3:160804167](genomebrowse://api/zoom?locus=3:160804167) | 0.0519169 | 0.0450673 | 0.0243327 | 0.046 | missense_variant | Tolerated | Probably damaging | Damaging |
| *B3GNT3* | UDP-GlcNAc:BetaGal Beta-1,3-N-Acetylglucosaminyltransferase 3 | rs114463415 | 19:17922769 | 0.00579073 | 0.00145817 | 0.00552504 | 0.001812 | synonymous_variant | - | - | - |
| *B3GNT3* | UDP-GlcNAc:BetaGal Beta-1,3-N-Acetylglucosaminyltransferase 4 | [rs77134098](http://www.ncbi.nlm.nih.gov/projects/SNP/snp_ref.cgi?rs=rs77134098) | [19:17922666](genomebrowse://api/zoom?locus=19:17922666) | 0.0109824 | 0.014488 | 0.0145368 | 0.014 | missense_variant | Tolerated | Benign | Damaging |
| *B3GNT8* | UDP-GlcNAc:BetaGal Beta-1,3-N-Acetylglucosaminyltransferase 8 | [rs45563938](http://www.ncbi.nlm.nih.gov/projects/SNP/snp_ref.cgi?rs=rs45563938) | [19:41932063](genomebrowse://api/zoom?locus=19:41932063) | 0.0626997 | 0.103095 | 0.0828473 | 0.103 | synonymous_variant | - | - | - |
| *B4GALNT4* | Beta-1,4-N-Acetyl-Galactosaminyltransferase 4 | rs150333376 | 11:375496 | 0.00559105 | 0.0011059 | 0.00424718 | 0.001369 | synonymous_variant | - | - | - |
| *BAALCOS* | BAALC Antisense RNA 2 | rs144964359 | 8:104145356 | 0.00379393 | 0.000627875 | 0.00410764 | 0.000572 | non_coding_exon_variant | - | - | - |
| *BAIAP2L1* | BAR/IMD Domain Containing Adaptor Protein 2 Like 1 | [rs11540936](http://www.ncbi.nlm.nih.gov/projects/SNP/snp_ref.cgi?rs=rs11540936) | [7:97937201](genomebrowse://api/zoom?locus=7:97937201) | 0.0313498 | 0.0180102 | 0.037868 | 0.019 | synonymous_variant | - | - | - |
| *BATF2* | Basic Leucine Zipper ATF-Like Transcription Factor 2 | rs143335222 | 11:64761971 | 0.00579073 | 0.000966603 | 0.00487663 | 0.001336 | synonymous_variant | - | - | - |
| *BAZ2A* | Bromodomain Adjacent To Zinc Finger Domain 2A | [rs2230580](http://www.ncbi.nlm.nih.gov/projects/SNP/snp_ref.cgi?rs=rs2230580) | [12:57003964](genomebrowse://api/zoom?locus=12:57003964) | 0.047524 | 0.0110789 | 0.0424892 | 0.014 | synonymous_variant | - | - | - |
| *BAZ2A* | Bromodomain Adjacent To Zinc Finger Domain 2A | [rs34393899](http://www.ncbi.nlm.nih.gov/projects/SNP/snp_ref.cgi?rs=rs34393899) | [12:57004268](genomebrowse://api/zoom?locus=12:57004268) | 0.0147764 | 0.0318293 | 0.0325595 | 0.032 | synonymous_variant | - | - | - |
| *BAZ2A* | Bromodomain Adjacent To Zinc Finger Domain 2A | [rs7974600](http://www.ncbi.nlm.nih.gov/projects/SNP/snp_ref.cgi?rs=rs7974600) | [12:57006998](genomebrowse://api/zoom?locus=12:57006998) | 0.0830671 | 0.0325007 | 0.0640189 | 0.036 | synonymous_variant | - | - | - |
| *BAZ2B* | Bromodomain Adjacent To Zinc Finger Domain 2B | [rs10202670](http://www.ncbi.nlm.nih.gov/projects/SNP/snp_ref.cgi?rs=rs10202670) | [2:160310246](genomebrowse://api/zoom?locus=2:160310246) | 0.0714856 | 0.106781 | 0.0895315 | 0.108 | missense_variant | Damaging | Benign | Tolerated |
| *BBS1* | Bardet-Biedl Syndrome 1 | rs55848325 | 11:66278154 | 0.00439297 | 0.00400257 | 0.00316456 | 0.004291 | synonymous_variant | - | - | - |
| *BBS10* | Bardet-Biedl Syndrome 10 | [rs35676114](http://www.ncbi.nlm.nih.gov/projects/SNP/snp_ref.cgi?rs=rs35676114) | [12:76740149](genomebrowse://api/zoom?locus=12:76740149) | 0.0335463 | 0.0639454 | 0.0520483 | 0.065 | missense_variant | Tolerated | Benign | Tolerated |
| *BBS4* | Bardet-Biedl Syndrome 4 | rs75295839 | 15:73002101 | 0.00319489 | 0.00689212 | 0.0056499 | 0.006861 | missense_variant | Tolerated | Benign | Damaging |
| *BBS9* | Bardet-Biedl Syndrome 9 | rs115809567 | 7:33545179 | 0.0081869 | 0.00187298 | 0.00690679 | 0.002454 | synonymous_variant | - | - | - |
| *BCAS1* | Breast Carcinoma Amplified Sequence 1 | [rs60878965](http://www.ncbi.nlm.nih.gov/projects/SNP/snp_ref.cgi?rs=rs60878965) | [20:52611552](genomebrowse://api/zoom?locus=20:52611552) | 0.0896565 | 0.0203556 | 0.0778553 | 0.026 | missense_variant | Damaging | Probably damaging | Tolerated |
| *BCHE* | Butyrylcholinesterase | [rs16849700](http://www.ncbi.nlm.nih.gov/projects/SNP/snp_ref.cgi?rs=rs16849700) | [3:165547973](genomebrowse://api/zoom?locus=3:165547973) | 0.0227636 | 0.0146167 | 0.0217785 | 0.016 | missense_variant | Tolerated | Benign | Damaging |
| *BCKDHA* | Branched Chain Keto Acid Dehydrogenase E1 Subunit Alpha | [rs11549936](http://www.ncbi.nlm.nih.gov/projects/SNP/snp_ref.cgi?rs=rs11549936) | [19:41916549](genomebrowse://api/zoom?locus=19:41916549) | 0.0553115 | 0.0991933 | 0.0776074 | 0.098 | missense_variant | Tolerated | Benign | Tolerated |
| *BCKDHB* | Branched Chain Keto Acid Dehydrogenase E1 Subunit Beta | [rs73479953](http://www.ncbi.nlm.nih.gov/projects/SNP/snp_ref.cgi?rs=rs73479953) | [6:80877371](genomebrowse://api/zoom?locus=6:80877371) | 0.129792 | 0.0776319 | 0.0681319 | 0.082 | intron_variant | - | - | - |
| *BCL2L2-PABPN1* | BCL2L2-PABPN1 Readthrough | [rs2295126](http://www.ncbi.nlm.nih.gov/projects/SNP/snp_ref.cgi?rs=rs2295126) | [14:23792215](genomebrowse://api/zoom?locus=14:23792215) | 0.0561102 | 0.0230316 | 0.053441 | 0.027 | synonymous_variant | - | - | - |
| *BCL9L* | BCL9 Like | [rs78780803](http://www.ncbi.nlm.nih.gov/projects/SNP/snp_ref.cgi?rs=rs78780803) | [11:118772825](genomebrowse://api/zoom?locus=11:118772825) | 0.0443291 | 0.0214747 | 0.0282781 | 0.021 | missense_variant | Tolerated | Benign | Tolerated |
| *BCMO1* | Beta-Carotene 15,15'-Monooxygenase 1 | [rs7501331](http://www.ncbi.nlm.nih.gov/projects/SNP/snp_ref.cgi?rs=rs7501331) | [16:81314496](genomebrowse://api/zoom?locus=16:81314496) | 0.152157 | 0.212198 | 0.185665 | 0.212 | missense_variant | Tolerated | Benign | Tolerated |
| *BDH2* | 3-Hydroxybutyrate Dehydrogenase 2 | [rs79460377](http://www.ncbi.nlm.nih.gov/projects/SNP/snp_ref.cgi?rs=rs79460377) | [4:104004064](genomebrowse://api/zoom?locus=4:104004064) | 0.0225639 | 0.055589 | 0.0666494 | 0.052 | synonymous_variant | - | - | - |
| *BDKRB1* | Bradykinin Receptor B1 | [rs33925361](http://www.ncbi.nlm.nih.gov/projects/SNP/snp_ref.cgi?rs=rs33925361) | [14:96730718](genomebrowse://api/zoom?locus=14:96730718) | 0.117212 | 0.0745543 | 0.0991142 | 0.077 | synonymous_variant | - | - | - |
| *BDKRB2* | Bradykinin Receptor B2 | [rs1046248](http://www.ncbi.nlm.nih.gov/projects/SNP/snp_ref.cgi?rs=rs1046248) | [14:96703484](genomebrowse://api/zoom?locus=14:96703484) | 0.0425319 | 0.0694132 | 0.0754102 | 0.069 | missense_variant | Damaging | Benign | Tolerated |
| *BDP1* | B Double Prime 1, Subunit Of RNA Polymerase III Transcription Initiation Factor IIIB | rs199669834 | 5:70810878 | 0.000199681 | 0.00138828 | 0.00151809 | 0.001573 | synonymous_variant | - | - | - |
| *BEST1* | Bestrophin 1 | [rs1109748](http://www.ncbi.nlm.nih.gov/projects/SNP/snp_ref.cgi?rs=rs1109748) | [11:61722645](genomebrowse://api/zoom?locus=11:61722645) | 0.317492 | 0.204598 | 0.113886 | 0.202 | synonymous_variant | - | - | - |
| *BEST3* | Bestrophin 3 | [rs17106884](http://www.ncbi.nlm.nih.gov/projects/SNP/snp_ref.cgi?rs=rs17106884) | [12:70048829](genomebrowse://api/zoom?locus=12:70048829) | 0.0433307 | 0.031804 | 0.0427731 | 0.032 | missense_variant | Damaging | Benign | Tolerated |
| *BICD1* | BICD Cargo Adaptor 1 | [rs34447944](http://www.ncbi.nlm.nih.gov/projects/SNP/snp_ref.cgi?rs=rs34447944) | [12:32481369](genomebrowse://api/zoom?locus=12:32481369) | 0.0463259 | 0.0752639 | 0.0557817 | 0.075 | synonymous_variant | - | - | - |
| *BID* | BH3 Interacting Domain Death Agonist | [rs147461488](http://www.ncbi.nlm.nih.gov/projects/SNP/snp_ref.cgi?rs=rs147461488) | [22:18222869](genomebrowse://api/zoom?locus=22:18222869) | 0.167532 | 0.117206 | 0.200395 | 0.121 | intron_variant | - | - | - |
| *BIRC6* | Baculoviral IAP Repeat Containing 6 | [rs34757266](http://www.ncbi.nlm.nih.gov/projects/SNP/snp_ref.cgi?rs=rs34757266) | [2:32740138](genomebrowse://api/zoom?locus=2:32740138) | 0.0353434 | 0.0743465 | 0.0665569 | 0.075 | synonymous_variant | - | - | - |
| *BIRC6* | Baculoviral IAP Repeat Containing 6 | [rs72800834](http://www.ncbi.nlm.nih.gov/projects/SNP/snp_ref.cgi?rs=rs72800834) | [2:32757349](genomebrowse://api/zoom?locus=2:32757349) | 0.033147 | 0.0715534 | 0.0666968 | 0.051 | intron_variant | - | - | - |
| *BLK* | BLK Proto-Oncogene, Src Family Tyrosine Kinase | [rs112254800](http://www.ncbi.nlm.nih.gov/projects/SNP/snp_ref.cgi?rs=rs112254800) | [8:11418678](genomebrowse://api/zoom?locus=8:11418678) | 0.0728834 | 0.0272962 | 0.0585361 | 0.017 | intron_variant | - | - | - |
| *BLM* | BLM RecQ Like Helicase | [rs7167216](http://www.ncbi.nlm.nih.gov/projects/SNP/snp_ref.cgi?rs=rs7167216) | [15:91354521](genomebrowse://api/zoom?locus=15:91354521) | 0.0670926 | 0.0663972 | 0.0854808 | 0.066 | missense_variant | Tolerated | Benign | Tolerated |
| *BLNK* | B Cell Linker | rs148612299 | 10:97983635 | 0.00299521 | 0.00609137 | 0.00374725 | 0.005477 | missense_variant | Tolerated | Benign | Tolerated |
| *BLNK* | B Cell Linker | [rs2242136](http://www.ncbi.nlm.nih.gov/projects/SNP/snp_ref.cgi?rs=rs2242136) | [10:97964261](genomebrowse://api/zoom?locus=10:97964261) | 0.109625 | 0.043503 | 0.0500678 | 0.046 | intron_variant | - | - | - |
| *BLVRA* | Biliverdin Reductase A | rs146389616 | 7:43840060 | 0.000399361 | 0.00031712 | 0.000290848 | 0.0002965 | splice_region_variant | - | - | - |
| *BMS1* | BMS1 Ribosome Biogenesis Factor | [rs7921071](http://www.ncbi.nlm.nih.gov/projects/SNP/snp_ref.cgi?rs=rs7921071) | [10:43289407](genomebrowse://api/zoom?locus=10:43289407) | 0.0986422 | 0.0639637 | 0.0693915 | 0.061 | synonymous_variant | - | - | - |
| *BMS1* | BMS1 Ribosome Biogenesis Factor | [rs11239786](http://www.ncbi.nlm.nih.gov/projects/SNP/snp_ref.cgi?rs=rs11239786) | [10:43312807](genomebrowse://api/zoom?locus=10:43312807) | 0.205072 | 0.202029 | 0.157252 | 0.203 | synonymous_variant | - | - | - |
| *BNC2* | Basonuclin 2 | [rs3739715](http://www.ncbi.nlm.nih.gov/projects/SNP/snp_ref.cgi?rs=rs3739715) | [9:16435714](genomebrowse://api/zoom?locus=9:16435714) | 0.0788738 | 0.074568 | 0.0884178 | 0.077 | synonymous_variant | - | - | - |
| *BNC2* | Basonuclin 2 | [rs76485966](http://www.ncbi.nlm.nih.gov/projects/SNP/snp_ref.cgi?rs=rs76485966) | [9:16738403](genomebrowse://api/zoom?locus=9:16738403) | 0.0493211 | 0.0197588 | 0.0288872 | 0.021 | synonymous_variant | - | - | - |
| *BNIPL* | BCL2 Interacting Protein Like | [rs12068365](http://www.ncbi.nlm.nih.gov/projects/SNP/snp_ref.cgi?rs=rs12068365) | [1:151015868](genomebrowse://api/zoom?locus=1:151015868) | 0.0583067 | 0.0586611 | 0.090915 | 0.061 | missense_variant | Tolerated | Benign | Tolerated |
| *BOLA1* | BolA Family Member 1 | [rs1044808](http://www.ncbi.nlm.nih.gov/projects/SNP/snp_ref.cgi?rs=rs1044808) | [1:149871905](genomebrowse://api/zoom?locus=1:149871905) | 0.0231629 | 0.0467268 | 0.0490044 | 0.047 | missense_variant | Tolerated | Benign | Tolerated |
| *BPI* | Bactericidal Permeability Increasing Protein | rs5743498 | 20:36932648 | 0.00299521 | 0.000801595 | 0.00323332 | 0.001046 | missense_variant | Tolerated | Benign | Tolerated |
| *BPI* | Bactericidal Permeability Increasing Protein | rs5743502 | 20:36937431 | 0.00419329 | 0.0063029 | 0.00597044 | 0.006161 | synonymous_variant | - | - | - |
| *BPIFB1* | BPI Fold Containing Family B Member 1 | [rs34548457](http://www.ncbi.nlm.nih.gov/projects/SNP/snp_ref.cgi?rs=rs34548457) | [20:31889150](genomebrowse://api/zoom?locus=20:31889150) | 0.0690895 | 0.0155983 | 0.0601822 | 0.02 | missense_variant | Damaging | Probably damaging | Tolerated |
| *BPIFB1* | BPI Fold Containing Family B Member 1 | [rs41311304](http://www.ncbi.nlm.nih.gov/projects/SNP/snp_ref.cgi?rs=rs41311304) | [20:31894766](genomebrowse://api/zoom?locus=20:31894766) | 0.0145767 | 0.0341266 | 0.0281044 | 0.033 | synonymous_variant | - | - | - |
| *BPIFB3* | BPI Fold Containing Family B Member 3 | rs150087753 | 20:31654638 | 0.00199681 | 0.00443028 | 0.00410074 | 0.004859 | synonymous_variant | - | - | - |
| *BPNT1* | 3'(2'), 5'-Bisphosphate Nucleotidase 1 | [rs34887640](http://www.ncbi.nlm.nih.gov/projects/SNP/snp_ref.cgi?rs=rs34887640) | [1:220231181](genomebrowse://api/zoom?locus=1:220231181) | 0.207069 | 0.289125 | 0.251669 | 0.264 | 3_prime_UTR_variant | - | - | - |
| *BRD1* | Bromodomain Containing 1 | [rs6009883](http://www.ncbi.nlm.nih.gov/projects/SNP/snp_ref.cgi?rs=rs6009883) | [22:50216739](genomebrowse://api/zoom?locus=22:50216739) | 0.0613019 | 0.0171641 | 0.0510052 | 0.02 | synonymous_variant | - | - | - |
| *BRD2* | Bromodomain Containing 2 | [rs2071876](http://www.ncbi.nlm.nih.gov/projects/SNP/snp_ref.cgi?rs=rs2071876) | [6:32948426](genomebrowse://api/zoom?locus=6:32948426) | 0.0605032 | 0.0694692 | 0.055882 | 0.069 | synonymous_variant | - | - | - |
| *BRD9* | Bromodomain Containing 9 | [rs73733976](http://www.ncbi.nlm.nih.gov/projects/SNP/snp_ref.cgi?rs=rs73733976) | [5:887533](genomebrowse://api/zoom?locus=5:887533) | 0.0365415 | 0.0112711 | 0.0368863 | 0.013 | synonymous_variant | - | - | - |
| *BRF1* | BRF1 RNA Polymerase III Transcription Initiation Factor Subunit | [rs28406206](http://www.ncbi.nlm.nih.gov/projects/SNP/snp_ref.cgi?rs=rs28406206) | [14:105688082](genomebrowse://api/zoom?locus=14:105688082) | 0.0489217 | 0.0675953 | 0.0785946 | 0.068 | synonymous_variant | - | - | - |
| *BRPF1* | Bromodomain And PHD Finger Containing 1 | rs143857877 | 3:9788910 | 0.00379393 | 0.000661918 | 0.00251922 | 0.0007578 | synonymous_variant | - | - | - |
| *BRSK1* | BR Serine/Threonine Kinase 1 | [rs12973169](http://www.ncbi.nlm.nih.gov/projects/SNP/snp_ref.cgi?rs=rs12973169) | [19:55817676](genomebrowse://api/zoom?locus=19:55817676) | 0.0900559 | 0.0842238 | 0.117485 | 0.084 | synonymous_variant | - | - | - |
| *BST1* | Bone Marrow Stromal Cell Antigen 1 | [rs2302464](http://www.ncbi.nlm.nih.gov/projects/SNP/snp_ref.cgi?rs=rs2302464) | [4:15709252](genomebrowse://api/zoom?locus=4:15709252) | 0.0790735 | 0.0470787 | 0.0386384 | 0.049 | missense_variant | Tolerated | Possibly damaging | Damaging |
| *BTBD3* | BTB Domain Containing 3 | [rs35364034](http://www.ncbi.nlm.nih.gov/projects/SNP/snp_ref.cgi?rs=rs35364034) | [20:11903654](genomebrowse://api/zoom?locus=20:11903654) | 0.0954473 | 0.0727416 | 0.0542355 | 0.072 | synonymous_variant | - | - | - |
| *BTBD7* | BTB Domain Containing 7 | rs146841523 | 14:93708881 | 0.00259585 | 0.000710602 | 0.0024543 | 0.0009142 | missense_variant | Tolerated | Benign | Tolerated |
| *BTD* | Biotinidase | rs145388314 | 3:15686008 | 0.00159744 | 0.00801618 | 0.00564954 | 0.00766 | synonymous_variant | - | - | - |
| *BTN2A2* | Butyrophilin Subfamily 2 Member A2 | rs62617840 | 6:26392905 | 0.0061901 | 0.00162059 | 0.00559074 | 0.002059 | missense_variant | Tolerated | Benign | Tolerated |
| *BTN3A1* | Butyrophilin Subfamily 3 Member A1 | [rs2393650](http://www.ncbi.nlm.nih.gov/projects/SNP/snp_ref.cgi?rs=rs2393650) | [6:26406396](genomebrowse://api/zoom?locus=6:26406396) | 0.173722 | 0.0666334 | 0.141094 | 0.047 | synonymous_variant | - | - | - |
| *BTN3A2* | Butyrophilin Subfamily 3 Member A2 | [rs148971714](http://www.ncbi.nlm.nih.gov/projects/SNP/snp_ref.cgi?rs=rs148971714) | [6:26369052](genomebrowse://api/zoom?locus=6:26369052) | - | 0.032691 | 0.0306668 | 0.047 | synonymous_variant | - | - | - |
| *BTNL10* | Butyrophilin Like 10 | [rs115476782](http://www.ncbi.nlm.nih.gov/projects/SNP/snp_ref.cgi?rs=rs115476782) | [1:228698311](genomebrowse://api/zoom?locus=1:228698311) | 0.0297524 | 0.0372057 | 0.029978 | 0.037 | missense_variant | - | - | - |
| *BTNL2* | Butyrophilin Like 2 | [rs41399051](http://www.ncbi.nlm.nih.gov/projects/SNP/snp_ref.cgi?rs=rs41399051) | [6:32369601](genomebrowse://api/zoom?locus=6:32369601) | 0.0159744 | 0.0267979 | 0.0221805 | 0.027 | intron_variant | - | - | - |
| *BTNL2* | Butyrophilin Like 2 | [rs28362681](http://www.ncbi.nlm.nih.gov/projects/SNP/snp_ref.cgi?rs=rs28362681) | [6:32370879](genomebrowse://api/zoom?locus=6:32370879) | 0.134185 | 0.114549 | 0.113095 | 0.112 | missense_variant | Tolerated | Benign | Tolerated |
| *BTNL8* | Butyrophilin Like 8 | rs145199317 | 5:180335720 | 0.00299521 | 0.00571329 | 0.0074916 | 0.005831 | missense_variant | Damaging | Possibly damaging | Tolerated |
| *C10orf128* | Chromosome 10 Open Reading Frame 128 | [rs17009534](http://www.ncbi.nlm.nih.gov/projects/SNP/snp_ref.cgi?rs=rs17009534) | [10:50396388](genomebrowse://api/zoom?locus=10:50396388) | 0.0465256 | 0.0205582 | 0.0254571 | 0.023 | 5_prime_UTR_variant | - | - | - |
| *C10orf71* | Chromosome 10 Open Reading Frame 71 | [rs11101093](http://www.ncbi.nlm.nih.gov/projects/SNP/snp_ref.cgi?rs=rs11101093) | [10:50532812](genomebrowse://api/zoom?locus=10:50532812) | 0.0954473 | 0.115602 | 0.101893 | 0.077 | missense_variant | Damaging | - | Tolerated |
| *C11orf35* | Chromosome 11 Open Reading Frame 35 | [rs11822657](http://www.ncbi.nlm.nih.gov/projects/SNP/snp_ref.cgi?rs=rs11822657) | [11:557584](genomebrowse://api/zoom?locus=11:557584) | 0.0543131 | 0.0526463 | 0.0540471 | 0.053 | synonymous_variant | - | - | - |
| *C11orf65* | Chromosome 11 Open Reading Frame 65 | rs137901968 | 11:108264068 | 0.00159744 | 0.00312388 | 0.00251954 | 0.003064 | missense_variant | Tolerated | Benign | Tolerated |
| *C11orf82* | Chromosome 11 Open Reading Frame 82 | [rs11826199](http://www.ncbi.nlm.nih.gov/projects/SNP/snp_ref.cgi?rs=rs11826199) | [11:82644764](genomebrowse://api/zoom?locus=11:82644764) | 0.0926518 | 0.0513973 | 0.0604719 | 0.05 | missense_variant | Damaging | Possibly damaging | Damaging |
| *C12orf10* | Chromosome 12 Open Reading Frame 10 | rs150319735 | 12:53700067 | 0.00299521 | 0.000422332 | 0.00109791 | 0.0005271 | missense_variant | Damaging | Probably damaging | Damaging |
| *C12orf40* | Chromosome 12 Open Reading Frame 40 | [rs73270414](http://www.ncbi.nlm.nih.gov/projects/SNP/snp_ref.cgi?rs=rs73270414) | [12:40085906](genomebrowse://api/zoom?locus=12:40085906) | 0.129792 | 0.132188 | 0.117689 | 0.135 | missense_variant | Tolerated | Benign | Tolerated |
| *C12orf56* | Chromosome 12 Open Reading Frame 56 | [rs113411861](http://www.ncbi.nlm.nih.gov/projects/SNP/snp_ref.cgi?rs=rs113411861) | [12:64712547](genomebrowse://api/zoom?locus=12:64712547) | 0.438099 | 0.407758 | 0.456724 | 0.39 | inframe_insertion | - | - | - |
| *C12orf56* | Chromosome 12 Open Reading Frame 56 | [rs73118352](http://www.ncbi.nlm.nih.gov/projects/SNP/snp_ref.cgi?rs=rs73118352) | [12:64746833](genomebrowse://api/zoom?locus=12:64746833) | 0.165335 | 0.140963 | 0.124584 | 0.14 | missense_variant | Tolerated | Benign | Tolerated |
| *C12orf66* | Chromosome 12 Open Reading Frame 66 | rs188114016 | 12:64587684 | 0.0071885 | 0.00255885 | 0.00723234 | 0.003021 | missense_variant | Tolerated | Benign | Tolerated |
| *C12orf73* | Chromosome 12 Open Reading Frame 73 | rs116667612 | 12:104347196 | 0.00778754 | 0.00163982 | 0.00697539 | 0.0009705 | intron_variant | - | - | - |
| *C12orf77* | Chromosome 12 Open Reading Frame 77 | [rs16928315](http://www.ncbi.nlm.nih.gov/projects/SNP/snp_ref.cgi?rs=rs16928315) | [12:25147192](genomebrowse://api/zoom?locus=12:25147192) | 0.157947 | 0.115841 | 0.078463 | 0.117 | 3_prime_UTR_variant | - | - | - |
| *C14orf37* | Chromosome 14 Open Reading Frame 37 | rs140057603 | 14:58563642 | 0.00219649 | 0.00416419 | 0.00633239 | 0.004242 | missense_variant | Tolerated | Probably damaging | Damaging |
| *C14orf37* | Chromosome 14 Open Reading Frame 38 | [rs11626667](http://www.ncbi.nlm.nih.gov/projects/SNP/snp_ref.cgi?rs=rs11626667) | [14:58604850](genomebrowse://api/zoom?locus=14:58604850) | 0.0253594 | 0.0373905 | 0.0412388 | 0.038 | synonymous_variant | - | - | - |
| *C14orf37* | Chromosome 14 Open Reading Frame 39 | [rs61741193](http://www.ncbi.nlm.nih.gov/projects/SNP/snp_ref.cgi?rs=rs61741193) | [14:58605072](genomebrowse://api/zoom?locus=14:58605072) | 0.0169728 | 0.0269437 | 0.0237357 | 0.026 | missense_variant | Damaging | Benign | Tolerated |
| *C15orf41* | Chromosome 15 Open Reading Frame 41 | c.716+24439delT | [15:37026601](genomebrowse://api/zoom?locus=15:37026601) | 0.239217 | 0.188916 | 0.173354 | 0.101 | intron_variant | - | - | - |
| *C15orf43* | Chromosome 15 Open Reading Frame 43 | [rs11638723](http://www.ncbi.nlm.nih.gov/projects/SNP/snp_ref.cgi?rs=rs11638723) | [15:45249121](genomebrowse://api/zoom?locus=15:45249121) | 0.0117812 | 0.0170309 | 0.0128729 | 0.016 | missense_variant | Tolerated | Benign | Tolerated |
| *C16orf52* | Chromosome 16 Open Reading Frame 52 | [rs201044196](http://www.ncbi.nlm.nih.gov/projects/SNP/snp_ref.cgi?rs=rs201044196) | [16:22019647](genomebrowse://api/zoom?locus=16:22019647) | 0.128195 | 0.109972 | 0.0687663 | 0.112 | frameshift_variant | - | - | - |
| *C16orf62* | Chromosome 16 Open Reading Frame 62 | [rs61738005](http://www.ncbi.nlm.nih.gov/projects/SNP/snp_ref.cgi?rs=rs61738005) | [16:19711717](genomebrowse://api/zoom?locus=16:19711717) | 0.0111821 | 0.0303476 | 0.0386753 | 0.032 | synonymous_variant | - | - | - |
| *C16orf89* | Chromosome 16 Open Reading Frame 89 | c.956-8dupT | [16:5094845](genomebrowse://api/zoom?locus=16:5094845) | - | 0.244577 | 0.0162072 | 0.166 | splice_region_variant | - | - | - |
| *C16orf96* | Chromosome 16 Open Reading Frame 96 | rs185475621 | 16:4625931 | 0.00359425 | 0.00343668 | 0.0068941 | 0.005678 | missense_variant | Damaging | Possibly damaging | Tolerated |
| *C17orf105* | Chromosome 17 Open Reading Frame 105 | [rs73987086](http://www.ncbi.nlm.nih.gov/projects/SNP/snp_ref.cgi?rs=rs73987086) | [17:41857869](genomebrowse://api/zoom?locus=17:41857869) | 0.0523163 | 0.0112544 | 0.0467317 | 0.019 | synonymous_variant | - | - | - |
| *C17orf53* | Chromosome 17 Open Reading Frame 53 | c.1074T>C | [17:42226245](genomebrowse://api/zoom?locus=17:42226245) | - | - | - | - | synonymous_variant | - | - | - |
| *C17orf77* | Chromosome 17 Open Reading Frame 77 | [rs58253413](http://www.ncbi.nlm.nih.gov/projects/SNP/snp_ref.cgi?rs=rs58253413) | [17:72588778](genomebrowse://api/zoom?locus=17:72588778) | 0.109625 | 0.0683017 | 0.0706666 | 0.07 | missense_variant | - | - | - |
| *C18orf54* | Chromosome 18 Open Reading Frame 54 | [rs1657904](http://www.ncbi.nlm.nih.gov/projects/SNP/snp_ref.cgi?rs=rs1657904) | [18:51887123](genomebrowse://api/zoom?locus=18:51887123) | 0.0609026 | 0.05557 | 0.0664942 | 0.057 | synonymous_variant | - | - | - |
| *C18orf64* | Chromosome 18 Open Reading Frame 64 | [rs12326052](http://www.ncbi.nlm.nih.gov/projects/SNP/snp_ref.cgi?rs=rs12326052) | [18:6588865](genomebrowse://api/zoom?locus=18:6588865) | 0.200879 | 0.119672 | 0.110018 | 0.116 | missense_variant | - | - | - |
| *C19orf12* | Chromosome 19 Open Reading Frame 12 | rs535994327 | 19:30191397 | 0.000199681 | 0.000294548 | 0.000355022 | 0.0004276 | 3_prime_UTR_variant | - | - | - |
| *C1orf101* | Chromosome 1 Open Reading Frame 101 | [rs34510134](http://www.ncbi.nlm.nih.gov/projects/SNP/snp_ref.cgi?rs=rs34510134) | [1:244715882](genomebrowse://api/zoom?locus=1:244715882) | 0.0613019 | 0.014937 | 0.0587514 | 0.019 | synonymous_variant | - | - | - |
| *C1orf101* | Chromosome 1 Open Reading Frame 101 | [rs35587664](http://www.ncbi.nlm.nih.gov/projects/SNP/snp_ref.cgi?rs=rs35587664) | [1:244715966](genomebrowse://api/zoom?locus=1:244715966) | 0.117612 | 0.0677074 | 0.125251 | 0.071 | synonymous_variant | - | - | - |
| *C1orf127* | Chromosome 1 Open Reading Frame 127 | rs1281016 | 1:11008594 | 0.0690895 | 0.0427693 | 0.0799806 | 0.046 | missense_variant | Tolerated | Benign | Tolerated |
| *C1orf173* | Chromosome 1 Open Reading Frame 173 | [rs2305549](http://www.ncbi.nlm.nih.gov/projects/SNP/snp_ref.cgi?rs=rs2305549) | [1:75055419](genomebrowse://api/zoom?locus=1:75055419) | 0.117612 | 0.111272 | 0.0921078 | 0.109 | missense_variant | Tolerated | Benign | Tolerated |
| *C1orf174* | Chromosome 1 Open Reading Frame 174 | rs12036962 | 1:3807258 | 0.0465256 | 0.0320274 | 0.0134777 | 0.028 | missense_variant | Tolerated | Benign | Tolerated |
| *C1orf195* | Chromosome 1 Open Reading Frame 195 | [rs78615461](http://www.ncbi.nlm.nih.gov/projects/SNP/snp_ref.cgi?rs=rs78615461) | [1:15495110](genomebrowse://api/zoom?locus=1:15495110) | 0.285343 | 0.21232 | 0.232469 | 0.197 | missense_variant | - | - | Tolerated |
| *C1orf52* | Chromosome 1 Open Reading Frame 52 | rs143779356 | 1:85718340 | 0.000199681 | 0.000602091 | 0.000646454 | 0.0006837 | missense_variant | Tolerated | Benign | Damaging |
| *C1QC* | Complement C1q C Chain | [rs36049190](http://www.ncbi.nlm.nih.gov/projects/SNP/snp_ref.cgi?rs=rs36049190) | [1:22973699](genomebrowse://api/zoom?locus=1:22973699) | 0.0263578 | 0.0495683 | 0.0489721 | 0.049 | intron_variant | - | - | - |
| *C1R* | Complement C1r | [rs75380747](http://www.ncbi.nlm.nih.gov/projects/SNP/snp_ref.cgi?rs=rs75380747) | [12:7188562](genomebrowse://api/zoom?locus=12:7188562) | 0.0617013 | 0.0390019 | 0.0354254 | 0.039 | stop_gained | - | - | - |
| *C2* | Complement C2 | [rs9332704](http://www.ncbi.nlm.nih.gov/projects/SNP/snp_ref.cgi?rs=rs9332704) | [6:31895619](genomebrowse://api/zoom?locus=6:31895619) | 0.0145767 | 0.0139647 | 0.0123433 | 0.014 | intron_variant | - | - | - |
| *C2* | Complement C2 | [rs9394078](http://www.ncbi.nlm.nih.gov/projects/SNP/snp_ref.cgi?rs=rs9394078) | [6:31868938](genomebrowse://api/zoom?locus=6:31868938) | 0.0585064 | 0.0439688 | 0.044402 | 0.042 | missense_variant | Tolerated | Benign | Damaging |
| *C20orf173* | Chromosome 20 Open Reading Frame 173 | [rs35756561](http://www.ncbi.nlm.nih.gov/projects/SNP/snp_ref.cgi?rs=rs35756561) | [20:34116307](genomebrowse://api/zoom?locus=20:34116307) | 0.0613019 | 0.05419 | 0.0279196 | 0.055 | synonymous_variant | - | - | - |
| *C20orf26* | Chromosome 20 Open Reading Frame 26 | [rs6075630](http://www.ncbi.nlm.nih.gov/projects/SNP/snp_ref.cgi?rs=rs6075630) | [20:20150027](genomebrowse://api/zoom?locus=20:20150027) | 0.139776 | 0.21512 | 0.193858 | 0.214 | synonymous_variant | - | - | - |
| *C20orf26* | Chromosome 20 Open Reading Frame 26 | [rs78741533](http://www.ncbi.nlm.nih.gov/projects/SNP/snp_ref.cgi?rs=rs78741533) | [20:20257962](genomebrowse://api/zoom?locus=20:20257962) | 0.0415335 | 0.0100072 | 0.0383919 | 0.013 | missense_variant | Tolerated | Possibly damaging | Tolerated |
| *C20orf26* | Chromosome 20 Open Reading Frame 26 | [rs2273056](http://www.ncbi.nlm.nih.gov/projects/SNP/snp_ref.cgi?rs=rs2273056) | [20:20033223](genomebrowse://api/zoom?locus=20:20033223) | 0.0726837 | 0.0601275 | 0.053958 | 0.059 | missense_variant | Damaging | Benign | Tolerated |
| *C20orf78* | Chromosome 20 Open Reading Frame 78 | rs556133893 | 20:18794612 | 0.000199681 | 4.06709e-05 | 3.22935e-05 | 3.295e-05 | synonymous_variant | - | - | - |
| *C21orf2* | Chromosome 21 Open Reading Frame 2 | [rs11552066](http://www.ncbi.nlm.nih.gov/projects/SNP/snp_ref.cgi?rs=rs11552066) | [21:45750145](genomebrowse://api/zoom?locus=21:45750145) | 0.086262 | 0.105507 | 0.0889104 | 0.097 | missense_variant | Tolerated | Benign | Tolerated |
| *C22orf42* | Chromosome 22 Open Reading Frame 42 | [rs8143121](http://www.ncbi.nlm.nih.gov/projects/SNP/snp_ref.cgi?rs=rs8143121) | [22:32550293](genomebrowse://api/zoom?locus=22:32550293) | 0.0299521 | 0.0426247 | 0.0345841 | 0.043 | missense_variant | Tolerated | Benign | Tolerated |
| *C22orf46* | Chromosome 22 Open Reading Frame 46 | c.535G>T | [22:42089785](genomebrowse://api/zoom?locus=22:42089785) | - | - | - | - | missense_variant | Damaging | Probably damaging | Tolerated |
| *C2CD2* | C2 Calcium Dependent Domain Containing 2 | [rs61735797](http://www.ncbi.nlm.nih.gov/projects/SNP/snp_ref.cgi?rs=rs61735797) | [21:43342111](genomebrowse://api/zoom?locus=21:43342111) | 0.077476 | 0.016101 | 0.0622251 | 0.02 | 5_prime_UTR_premature_start_codon_gain_variant | - | - | - |
| *C2CD5* | C2 Calcium Dependent Domain Containing 5 | [rs10842027](http://www.ncbi.nlm.nih.gov/projects/SNP/snp_ref.cgi?rs=rs10842027) | [12:22625661](genomebrowse://api/zoom?locus=12:22625661) | 0.187101 | 0.0892465 | 0.157987 | 0.098 | intron_variant | - | - | - |
| *C2orf16* | Chromosome 2 Open Reading Frame 16 | rs139381292 | 2:27802145 | 0.00499201 | 0.00739661 | 0.00474776 | 0.006867 | synonymous_variant | - | - | - |
| *C2orf57* | Chromosome 2 Open Reading Frame 57 | [rs17199614](http://www.ncbi.nlm.nih.gov/projects/SNP/snp_ref.cgi?rs=rs17199614) | [2:232457857](genomebrowse://api/zoom?locus=2:232457857) | 0.0145767 | 0.0234301 | 0.019696 | 0.023 | synonymous_variant | - | - | - |
| *C2orf71* | Chromosome 2 Open Reading Frame 71 | rs189042259 | 2:29294710 | 0.00259585 | 0.000365524 | 0.00142091 | 0.0004881 | synonymous_variant | - | - | - |
| *C2orf71* | Chromosome 2 Open Reading Frame 71 | [rs144569618](http://www.ncbi.nlm.nih.gov/projects/SNP/snp_ref.cgi?rs=rs144569618) | [2:29294239](genomebrowse://api/zoom?locus=2:29294239) | 0.0313498 | 0.0486596 | 0.0220032 | 0.041 | synonymous_variant | - | - | - |
| *C2orf74* | Chromosome 2 Open Reading Frame 74 | rs528746738 | 2:61385081 | 0.000399361 | 0.00135225 | 0.000613537 | 0.002452 | intron_variant | - | - | - |
| *C3* | Complement C3 | [rs11569471](http://www.ncbi.nlm.nih.gov/projects/SNP/snp_ref.cgi?rs=rs11569471) | [19:6697590](genomebrowse://api/zoom?locus=19:6697590) | 0.0477236 | 0.0108879 | 0.0417395 | 0.014 | intron_variant | - | - | - |
| *C3* | Complement C3 | [rs2230203](http://www.ncbi.nlm.nih.gov/projects/SNP/snp_ref.cgi?rs=rs2230203) | [19:6710782](genomebrowse://api/zoom?locus=19:6710782) | 0.0636981 | 0.126604 | 0.121692 | 0.128 | synonymous_variant | - | - | - |
| *C3* | Complement C3 | [rs1047286](http://www.ncbi.nlm.nih.gov/projects/SNP/snp_ref.cgi?rs=rs1047286) | [19:6713262](genomebrowse://api/zoom?locus=19:6713262) | 0.0698882 | 0.142323 | 0.135023 | 0.143 | missense_variant | Damaging | Benign | Tolerated |
| *C3orf22* | Chromosome 3 Open Reading Frame 22 | [rs34760151](http://www.ncbi.nlm.nih.gov/projects/SNP/snp_ref.cgi?rs=rs34760151) | [3:126270923](genomebrowse://api/zoom?locus=3:126270923) | 0.0960463 | 0.0426396 | 0.0776643 | 0.047 | synonymous_variant | - | - | - |
| *C3orf35* | Chromosome 3 Open Reading Frame 35 | [rs17266511](http://www.ncbi.nlm.nih.gov/projects/SNP/snp_ref.cgi?rs=rs17266511) | [3:37476427](genomebrowse://api/zoom?locus=3:37476427) | 0.0621006 | 0.083783 | 0.0876672 | 0.084 | missense_variant | - | - | - |
| *C3orf62* | Chromosome 3 Open Reading Frame 62 | [rs13077498](http://www.ncbi.nlm.nih.gov/projects/SNP/snp_ref.cgi?rs=rs13077498) | [3:49313978](genomebrowse://api/zoom?locus=3:49313978) | 0.0650958 | 0.088177 | 0.0956011 | 0.088 | missense_variant | Tolerated | Benign | Tolerated |
| *C4A* | Complement C4A (Rodgers Blood Group) | rs375745191 | 6:31964140 | 0.00539137 | 0.00218665 | 0.00663507 | 0.002338 | intron_variant | - | - | - |
| *C4A* | Complement C4A (Rodgers Blood Group) | [rs7751519](http://www.ncbi.nlm.nih.gov/projects/SNP/snp_ref.cgi?rs=rs7751519) | [6:31994742](genomebrowse://api/zoom?locus=6:31994742) | 0.132188 | 0.095439 | 0.158447 | 0.087 | intron_variant | - | - | - |
| *C4A* | Complement C4A (Rodgers Blood Group) | [rs7771952](http://www.ncbi.nlm.nih.gov/projects/SNP/snp_ref.cgi?rs=rs7771952) | [6:31994750](genomebrowse://api/zoom?locus=6:31994750) | - | 0.0954741 | 0.156295 | 0.087 | intron_variant | - | - | - |
| *C4orf21* | Chromosome 4 Open Reading Frame 21 | [rs61745597](http://www.ncbi.nlm.nih.gov/projects/SNP/snp_ref.cgi?rs=rs61745597) | [4:113544993](genomebrowse://api/zoom?locus=4:113544993) | 0.0203674 | 0.0121954 | 0.011196 | 0.012 | missense_variant | Tolerated | Benign | Tolerated |
| *C4orf29* | Chromosome 4 Open Reading Frame 29 | [rs35763794](http://www.ncbi.nlm.nih.gov/projects/SNP/snp_ref.cgi?rs=rs35763794) | [4:128949850](genomebrowse://api/zoom?locus=4:128949850) | 0.0734824 | 0.0528169 | 0.0312399 | 0.046 | missense_variant | Tolerated | Benign | Tolerated |
| *C5* | Complement C5 | rs34552775 | 9:123785738 | 0.000998403 | 0.00542927 | 0.00787707 | 0.005156 | missense_variant | Damaging | Probably damaging | Damaging |
| *C5* | Complement C5 | [rs12237774](http://www.ncbi.nlm.nih.gov/projects/SNP/snp_ref.cgi?rs=rs12237774) | [9:123725971](genomebrowse://api/zoom?locus=9:123725971) | 0.106629 | 0.0440728 | 0.0784402 | 0.048 | synonymous_variant | - | - | - |
| *C5AR2* | Complement Component 5a Receptor 2 | rs138219631 | 19:47844720 | 0.00399361 | 0.000862678 | 0.00291055 | 0.001014 | missense_variant | - | Benign | Tolerated |
| *C5AR2* | Complement Component 5a Receptor 2 | [rs36046934](http://www.ncbi.nlm.nih.gov/projects/SNP/snp_ref.cgi?rs=rs36046934) | [19:47844653](genomebrowse://api/zoom?locus=19:47844653) | 0.0171725 | 0.0147509 | 0.0142959 | 0.015 | synonymous_variant | - | - | - |
| *C5orf47* | Chromosome 5 Open Reading Frame 47 | [rs62378684](http://www.ncbi.nlm.nih.gov/projects/SNP/snp_ref.cgi?rs=rs62378684) | [5:173428228](genomebrowse://api/zoom?locus=5:173428228) | 0.0305511 | 0.0484721 | 0.0428442 | 0.053 | 3_prime_UTR_variant | - | - | - |
| *C5orf49* | Chromosome 5 Open Reading Frame 49 | rs143664639 | 5:7832056 | 0.00259585 | 0.00342786 | 0.00226113 | 0.00344 | missense_variant | Tolerated | Benign | Tolerated |
| *C5orf52* | Chromosome 5 Open Reading Frame 52 | [rs10051838](http://www.ncbi.nlm.nih.gov/projects/SNP/snp_ref.cgi?rs=rs10051838) | [5:157102159](genomebrowse://api/zoom?locus=5:157102159) | 0.182308 | 0.134875 | 0.178227 | 0.151 | missense_variant | Tolerated | Benign | Tolerated |
| *C6* | Complement C6 | rs28651920 | 5:41155108 | 0.00319489 | 0.000826372 | 0.00258348 | 0.001095 | synonymous_variant | - | - | - |
| *C6orf10* | Chromosome 6 Open Reading Frame 10 | [rs7751028](http://www.ncbi.nlm.nih.gov/projects/SNP/snp_ref.cgi?rs=rs7751028) | [6:32261014](genomebrowse://api/zoom?locus=6:32261014) | 0.0503195 | 0.0231243 | 0.0442693 | 0.024 | missense_variant | Damaging | Probably damaging | Tolerated |
| *C6orf10* | Chromosome 6 Open Reading Frame 10 | [rs114543649](http://www.ncbi.nlm.nih.gov/projects/SNP/snp_ref.cgi?rs=rs114543649) | [6:32261158](genomebrowse://api/zoom?locus=6:32261158) | 0.0503195 | 0.0230917 | 0.0439617 | 0.024 | missense_variant | Tolerated | Benign | Tolerated |
| *C6orf10* | Chromosome 6 Open Reading Frame 10 | [rs61995678](http://www.ncbi.nlm.nih.gov/projects/SNP/snp_ref.cgi?rs=rs61995678) | [6:32261291](genomebrowse://api/zoom?locus=6:32261291) | 0.0503195 | 0.023146 | 0.0446053 | 0.024 | missense_variant | Tolerated | Benign | Tolerated |
| *C6orf10* | Chromosome 6 Open Reading Frame 10 | [rs4947338](http://www.ncbi.nlm.nih.gov/projects/SNP/snp_ref.cgi?rs=rs4947338) | [6:32261653](genomebrowse://api/zoom?locus=6:32261653) | 0.0503195 | 0.0231315 | 0.04413 | 0.024 | missense_variant | Damaging | Probably damaging | Tolerated |
| *C6orf10* | Chromosome 6 Open Reading Frame 10 | [rs115212456](http://www.ncbi.nlm.nih.gov/projects/SNP/snp_ref.cgi?rs=rs115212456) | [6:32303154](genomebrowse://api/zoom?locus=6:32303154) | 0.0207668 | - | 0.0216318 | - | intron_variant | - | - | - |
| *C6orf132* | Chromosome 6 Open Reading Frame 132 | [rs9394863](http://www.ncbi.nlm.nih.gov/projects/SNP/snp_ref.cgi?rs=rs9394863) | [6:42072989](genomebrowse://api/zoom?locus=6:42072989) | 0.217053 | 0.125269 | 0.128668 | 0.137 | synonymous_variant | - | - | - |
| *C6orf141* | Chromosome 6 Open Reading Frame 141 | [rs41273692](http://www.ncbi.nlm.nih.gov/projects/SNP/snp_ref.cgi?rs=rs41273692) | [6:49519169](genomebrowse://api/zoom?locus=6:49519169) | 0.061901 | 0.0308302 | 0.059379 | 0.043 | missense_variant | Tolerated | Benign | Tolerated |
| *C6orf163* | Chromosome 6 Open Reading Frame 163 | [rs41273289](http://www.ncbi.nlm.nih.gov/projects/SNP/snp_ref.cgi?rs=rs41273289) | [6:88074904](genomebrowse://api/zoom?locus=6:88074904) | 0.061901 | 0.0120017 | 0.0517676 | 0.018 | synonymous_variant | - | - | - |
| *C7* | Complement C7 | [rs60714178](http://www.ncbi.nlm.nih.gov/projects/SNP/snp_ref.cgi?rs=rs60714178) | [5:40964885](genomebrowse://api/zoom?locus=5:40964885) | 0.0660942 | 0.116436 | 0.119952 | 0.117 | missense_variant | Tolerated | Benign | Tolerated |
| *C7orf13* | Chromosome 7 Open Reading Frame 13 | [rs76141520](http://www.ncbi.nlm.nih.gov/projects/SNP/snp_ref.cgi?rs=rs76141520) | [7:156432793](genomebrowse://api/zoom?locus=7:156432793) | 0.0235623 | 0.0477432 | 0.0524598 | 0.029 | non_coding_exon_variant | - | - | - |
| *C7orf33* | Chromosome 7 Open Reading Frame 33 | rs145588023 | 7:148288054 | 0.000399361 | 0.00173814 | 0.00226347 | 0.001713 | missense_variant | Tolerated | Benign | Tolerated |
| *C7orf33* | Chromosome 7 Open Reading Frame 33 | [rs62624492](http://www.ncbi.nlm.nih.gov/projects/SNP/snp_ref.cgi?rs=rs62624492) | [7:148288020](genomebrowse://api/zoom?locus=7:148288020) | 0.0273562 | 0.0420685 | 0.0216197 | 0.039 | initiator_codon_variant | Damaging | Benign | Damaging |
| *C7orf33* | Chromosome 7 Open Reading Frame 33 | [rs111320068](http://www.ncbi.nlm.nih.gov/projects/SNP/snp_ref.cgi?rs=rs111320068) | [7:148288135](genomebrowse://api/zoom?locus=7:148288135) | 0.0261581 | 0.0425777 | 0.0215693 | 0.039 | synonymous_variant | - | - | - |
| *C7orf63* | Chromosome 7 Open Reading Frame 63 | [rs17863057](http://www.ncbi.nlm.nih.gov/projects/SNP/snp_ref.cgi?rs=rs17863057) | [7:89897703](genomebrowse://api/zoom?locus=7:89897703) | 0.0650958 | 0.0392513 | 0.0378621 | 0.041 | splice_region_variant | - | - | - |
| *C7orf69* | Chromosome 7 Open Reading Frame 69 | [rs146576726](http://www.ncbi.nlm.nih.gov/projects/SNP/snp_ref.cgi?rs=rs146576726) | [7:47840310](genomebrowse://api/zoom?locus=7:47840310) | 0.0259585 | 0.0299127 | 0.0153022 | 0.029 | missense_variant | Tolerated | Benign | Tolerated |
| *C8A* | Complement C8 Alpha Chain | rs114723546 | 1:57351559 | 0.00439297 | 0.0012482 | 0.00465267 | 0.001557 | intron_variant | - | - | - |
| *C8A* | Complement C8 Alpha Chain | [rs1620075](http://www.ncbi.nlm.nih.gov/projects/SNP/snp_ref.cgi?rs=rs1620075) | [1:57378149](genomebrowse://api/zoom?locus=1:57378149) | 0.136182 | 0.0755517 | 0.0959785 | 0.077 | missense_variant | Damaging | Possibly damaging | Tolerated |
| *C8A* | Complement C8 Alpha Chain | [rs1620073](http://www.ncbi.nlm.nih.gov/projects/SNP/snp_ref.cgi?rs=rs1620073) | [1:57378150](genomebrowse://api/zoom?locus=1:57378150) | 0.136382 | 0.075638 | 0.095986 | 0.077 | synonymous_variant | - | - | - |
| *C8B* | Complement C8 Beta Chain | [rs72670382](http://www.ncbi.nlm.nih.gov/projects/SNP/snp_ref.cgi?rs=rs72670382) | [1:57431629](genomebrowse://api/zoom?locus=1:57431629) | 0.0113818 | 0.0192093 | 0.0176113 | 0.018 | 5_prime_UTR_variant | - | - | - |
| *C8orf48* | Chromosome 8 Open Reading Frame 48 | rs184032075 | 8:13424840 | 0.000199681 | 0.00126596 | 0.00138871 | 0.00104 | missense_variant | Tolerated | Benign | Tolerated |
| *C9orf171* | Chromosome 9 Open Reading Frame 171 | [rs61308567](http://www.ncbi.nlm.nih.gov/projects/SNP/snp_ref.cgi?rs=rs61308567) | [9:135418389](genomebrowse://api/zoom?locus=9:135418389) | 0.0489217 | 0.010288 | 0.0405318 | 0.013 | synonymous_variant | - | - | - |
| *C9orf173* | Chromosome 9 Open Reading Frame 173 | [rs61759822](http://www.ncbi.nlm.nih.gov/projects/SNP/snp_ref.cgi?rs=rs61759822) | [9:140147273](genomebrowse://api/zoom?locus=9:140147273) | 0.142772 | 0.131579 | 0.103697 | 0.105 | missense_variant | Tolerated | Possibly damaging | Tolerated |
| *C9orf72* | C9orf72-SMCR8 Complex Subunit | [rs17696570](http://www.ncbi.nlm.nih.gov/projects/SNP/snp_ref.cgi?rs=rs17696570) | [9:27558437](genomebrowse://api/zoom?locus=9:27558437) | 0.0155751 | - | 0.0262613 | - | intron_variant | - | - | - |
| *C9orf84* | Chromosome 9 Open Reading Frame 84 | [rs12683681](http://www.ncbi.nlm.nih.gov/projects/SNP/snp_ref.cgi?rs=rs12683681) | [9:114470127](genomebrowse://api/zoom?locus=9:114470127) | 0.0814696 | 0.0467816 | 0.0653172 | 0.05 | splice_region_variant | - | - | - |
| *C9orf84* | Chromosome 9 Open Reading Frame 84 | [rs7868266](http://www.ncbi.nlm.nih.gov/projects/SNP/snp_ref.cgi?rs=rs7868266) | [9:114470138](genomebrowse://api/zoom?locus=9:114470138) | 0.0752796 | 0.025818 | 0.0575393 | 0.033 | missense_variant | Tolerated | Benign | Tolerated |
| *CA6* | Carbonic Anhydrase 6 | rs2274329 | 1:9009451 | 0.0946486 | 0.0930587 | 0.0696215 | 0.092 | missense_variant | Damaging | Probably damaging | Damaging |
| *CA8* | Carbonic Anhydrase 8 | [rs61743130](http://www.ncbi.nlm.nih.gov/projects/SNP/snp_ref.cgi?rs=rs61743130) | [8:61178567](genomebrowse://api/zoom?locus=8:61178567) | 0.0551118 | 0.0191173 | 0.0518801 | 0.022 | synonymous_variant | - | - | - |
| *CAAP1* | Caspase Activity And Apoptosis Inhibitor 1 | [rs10967558](http://www.ncbi.nlm.nih.gov/projects/SNP/snp_ref.cgi?rs=rs10967558) | [9:26842506](genomebrowse://api/zoom?locus=9:26842506) | 0.0483227 | 0.0106901 | 0.0417447 | 0.013 | synonymous_variant | - | - | - |
| *CAAP1* | Caspase Activity And Apoptosis Inhibitor 1 | [rs12342214](http://www.ncbi.nlm.nih.gov/projects/SNP/snp_ref.cgi?rs=rs12342214) | [9:26861106](genomebrowse://api/zoom?locus=9:26861106) | 0.0513179 | 0.0108166 | 0.0422053 | 0.014 | missense_variant | Tolerated | Benign | Damaging |
| *CABLES2* | Cdk5 And Abl Enzyme Substrate 2 | [rs13042761](http://www.ncbi.nlm.nih.gov/projects/SNP/snp_ref.cgi?rs=rs13042761) | [20:60966374](genomebrowse://api/zoom?locus=20:60966374) | 0.0892572 | 0.0709661 | 0.0961489 | 0.074 | synonymous_variant | - | - | - |
| *CACNA1D* | Calcium Voltage-Gated Channel Subunit Alpha1 D | [rs2306865](http://www.ncbi.nlm.nih.gov/projects/SNP/snp_ref.cgi?rs=rs2306865) | [3:53761071](genomebrowse://api/zoom?locus=3:53761071) | 0.0439297 | 0.0316314 | 0.0256667 | 0.032 | intron_variant | - | - | - |
| *CACNA1D* | Calcium Voltage-Gated Channel Subunit Alpha1 D | [rs955905417](http://www.ncbi.nlm.nih.gov/projects/SNP/snp_ref.cgi?rs=rs955905417) | [3:53836195](genomebrowse://api/zoom?locus=3:53836195) | - | - | - | - | synonymous_variant | - | - | - |
| *CACNA1E* | Calcium Voltage-Gated Channel Subunit Alpha1 E | rs183395195 | 1:181689970 | 0.000399361 | 0.000961743 | 0.000807129 | 0.0008854 | synonymous_variant | - | - | - |
| *CACNA1S* | Calcium Voltage-Gated Channel Subunit Alpha1 S | [rs16847623](http://www.ncbi.nlm.nih.gov/projects/SNP/snp_ref.cgi?rs=rs16847623) | [1:201035429](genomebrowse://api/zoom?locus=1:201035429) | 0.0347444 | 0.0107324 | 0.0242469 | 0.012 | synonymous_variant | - | - | - |
| *CACNA1S* | Calcium Voltage-Gated Channel Subunit Alpha1 S | [rs16847674](http://www.ncbi.nlm.nih.gov/projects/SNP/snp_ref.cgi?rs=rs16847674) | [1:201047135](genomebrowse://api/zoom?locus=1:201047135) | 0.122005 | 0.0866048 | 0.0965366 | 0.085 | synonymous_variant | - | - | - |
| *CACNA2D1* | Calcium Voltage-Gated Channel Auxiliary Subunit Alpha2delta 1 | rs148573351 | 7:81714063 | 0.0091853 | 0.00155571 | 0.00619835 | 0.002018 | intron_variant | - | - | - |
| *CACNG6* | Calcium Voltage-Gated Channel Auxiliary Subunit Gamma 6 | [rs34604090](http://www.ncbi.nlm.nih.gov/projects/SNP/snp_ref.cgi?rs=rs34604090) | [19:54501527](genomebrowse://api/zoom?locus=19:54501527) | 0.090655 | 0.0635941 | 0.109242 | 0.068 | synonymous_variant | - | - | - |
| *CALB1* | Calbindin 1 | [rs78054626](http://www.ncbi.nlm.nih.gov/projects/SNP/snp_ref.cgi?rs=rs78054626) | [8:91090553](genomebrowse://api/zoom?locus=8:91090553) | 0.0758786 | 0.0204894 | 0.0634186 | 0.025 | intron_variant | - | - | - |
| *CALB2* | Calbindin 2 | rs201550865 | 16:71417236 | 0.000199681 | 0.000800236 | 0.000710778 | 0.0007578 | intron_variant | - | - | - |
| *CALCA* | Calcitonin Related Polypeptide Alpha | [rs5241](http://www.ncbi.nlm.nih.gov/projects/SNP/snp_ref.cgi?rs=rs5241) | [11:14990543](genomebrowse://api/zoom?locus=11:14990543) | 0.0547125 | 0.0205763 | 0.0486874 | 0.023 | missense_variant | Tolerated | Benign | Damaging |
| *CAMTA2* | Calmodulin Binding Transcription Activator 2 | c.2480G>T | [17:4876156](genomebrowse://api/zoom?locus=17:4876156) | - | - | - | - | missense_variant | Tolerated | Probably damaging | Tolerated |
| *CAPN1* | Calpain 1 | rs17880800 | 11:64950331 | 0.0061901 | 0.001321 | 0.0053126 | 0.001414 | synonymous_variant | - | - | - |
| *CAPN13* | Calpain 13 | rs80066698 | 2:30966294 | 0.00399361 | 0.00139335 | 0.00535864 | 0.001597 | missense_variant | Tolerated | Benign | Tolerated |
| *CAPN3* | Calpain 3 | [rs28364393](http://www.ncbi.nlm.nih.gov/projects/SNP/snp_ref.cgi?rs=rs28364393) | [15:42677959](genomebrowse://api/zoom?locus=15:42677959) | 0.115615 | 0.0672468 | 0.0525363 | 0.058 | intron_variant | - | - | - |
| *CAPN6* | Calpain 6 | [rs17882737](http://www.ncbi.nlm.nih.gov/projects/SNP/snp_ref.cgi?rs=rs17882737) | [X:110496391](genomebrowse://api/zoom?locus=X:110496391) | 0.0831788 | 0.0251108 | 0.0723092 | 0.029 | synonymous_variant | - | - | - |
| *CAPN9* | Calpain 9 | [rs7517389](http://www.ncbi.nlm.nih.gov/projects/SNP/snp_ref.cgi?rs=rs7517389) | [1:230910339](genomebrowse://api/zoom?locus=1:230910339) | 0.0944489 | 0.069558 | 0.0606531 | 0.069 | synonymous_variant | - | - | - |
| *CARD14* | Caspase Recruitment Domain Family Member 14 | rs150536049 | 17:78165166 | 0.000399361 | 0.000374626 | 0.000161426 | 0.0003212 | missense_variant | Tolerated | Benign | Tolerated |
| *CARD14* | Caspase Recruitment Domain Family Member 14 | [rs11658460](http://www.ncbi.nlm.nih.gov/projects/SNP/snp_ref.cgi?rs=rs11658460) | [17:78166385](genomebrowse://api/zoom?locus=17:78166385) | 0.121406 | 0.108408 | 0.10647 | 0.112 | synonymous_variant | - | - | - |
| *CARD8* | Caspase Recruitment Domain Family Member 8 | rs369625179 | 19:48722214 | 0.000199681 | 4.46897e-05 | 6.45786e-05 | 4.118e-05 | missense_variant | Damaging | Possibly damaging | Tolerated |
| *CARD8* | Caspase Recruitment Domain Family Member 8 | [rs8112588](http://www.ncbi.nlm.nih.gov/projects/SNP/snp_ref.cgi?rs=rs8112588) | [19:48715036](genomebrowse://api/zoom?locus=19:48715036) | 0.0493211 | 0.0106504 | 0.0389535 | 0.014 | synonymous_variant | - | - | - |
| *CARNS1* | Carnosine Synthase 1 | rs41302427 | 11:67186995 | 0.00399361 | 0.00378596 | 0.00300912 | 0.003724 | missense_variant | Damaging | Benign | Tolerated |
| *CASP2* | Caspase 2 | [rs4647288](http://www.ncbi.nlm.nih.gov/projects/SNP/snp_ref.cgi?rs=rs4647288) | [7:142988726](genomebrowse://api/zoom?locus=7:142988726) | 0.0115815 | 0.0229803 | 0.0209033 | 0.023 | synonymous_variant | - | - | - |
| *CASP2* | Caspase 2 | [rs4647297](http://www.ncbi.nlm.nih.gov/projects/SNP/snp_ref.cgi?rs=rs4647297) | [7:142991361](genomebrowse://api/zoom?locus=7:142991361) | 0.0391374 | 0.0482388 | 0.0489829 | 0.049 | missense_variant | Tolerated | Benign | Tolerated |
| *CASP7* | Caspase 7 | rs61755278 | 10:115489187 | 0.000599042 | 0.000852937 | 0.000387372 | 0.0007825 | missense_variant | Tolerated | Benign | Tolerated |
| *CASP7* | Caspase 7 | [rs12358524](http://www.ncbi.nlm.nih.gov/projects/SNP/snp_ref.cgi?rs=rs12358524) | [10:115451781](genomebrowse://api/zoom?locus=10:115451781) | 0.0708866 | 0.0895057 | 0.0804022 | 0.089 | synonymous_variant | - | - | - |
| *CASP8* | Caspase 8 | [rs3769824](http://www.ncbi.nlm.nih.gov/projects/SNP/snp_ref.cgi?rs=rs3769824) | [2:202122956](genomebrowse://api/zoom?locus=2:202122956) | 0.0345447 | 0.0491842 | 0.0372747 | 0.045 | initiator_codon_variant | Damaging | Benign | Damaging |
| *CASP8* | Caspase 8 | [rs1045485](http://www.ncbi.nlm.nih.gov/projects/SNP/snp_ref.cgi?rs=rs1045485) | [2:202149589](genomebrowse://api/zoom?locus=2:202149589) | 0.0527157 | 0.0898428 | 0.0973354 | 0.091 | missense_variant | Tolerated | Benign | Tolerated |
| *CASP9* | Caspase 9 | rs370730070 | 1:15850672 | 0.00399361 | 0.000550912 | 0.00350263 | 0.000285 | synonymous_variant | - | - | - |
| *CASR* | Calcium Sensing Receptor | [rs1042636](http://www.ncbi.nlm.nih.gov/projects/SNP/snp_ref.cgi?rs=rs1042636) | [3:122003769](genomebrowse://api/zoom?locus=3:122003769) | 0.20647 | 0.150325 | 0.104064 | 0.145 | missense_variant | Damaging | Benign | Damaging |
| *CAT* | Catalase | rs61752922 | 11:34472623 | 0.000399361 | 0.00191675 | 0.00145236 | 0.002084 | synonymous_variant | - | - | - |
| *CATSPER1* | Cation Channel Sperm Associated 1 | rs147398525 | 11:65789120 | 0.00678914 | 0.00672515 | 0.00559726 | 0.006536 | splice_region_variant | - | - | - |
| *CATSPER2* | Cation Channel Sperm Associated 2 | rs201425755 | 15:43924899 | 0.000199681 | 0.00116477 | 0.0011978 | 0.001137 | intron_variant | - | - | - |
| *CATSPER3* | Cation Channel Sperm Associated 3 | [rs112278352](http://www.ncbi.nlm.nih.gov/projects/SNP/snp_ref.cgi?rs=rs112278352) | [5:134344706](genomebrowse://api/zoom?locus=5:134344706) | 0.0577077 | 0.0142087 | 0.0550382 | 0.018 | intron_variant | - | - | - |
| *CATSPER3* | Cation Channel Sperm Associated 3 | [rs7719874](http://www.ncbi.nlm.nih.gov/projects/SNP/snp_ref.cgi?rs=rs7719874) | [5:134347241](genomebrowse://api/zoom?locus=5:134347241) | 0.0585064 | 0.0142285 | 0.0551067 | 0.018 | synonymous_variant | - | - | - |
| *CATSPER4* | Cation Channel Sperm Associated 4 | rs142583631 | 1:26517811 | 0.00339457 | 0.00673793 | 0.00446342 | 0.006622 | missense_variant | Damaging | Benign | Tolerated |
| *CATSPER4* | Cation Channel Sperm Associated 4 | [rs41310785](http://www.ncbi.nlm.nih.gov/projects/SNP/snp_ref.cgi?rs=rs41310785) | [1:26527884](genomebrowse://api/zoom?locus=1:26527884) | 0.0227636 | 0.0555528 | 0.0664055 | 0.053 | synonymous_variant | - | - | - |
| *CATSPERB* | Cation Channel Sperm Associated Auxiliary Subunit Beta | [rs78613025](http://www.ncbi.nlm.nih.gov/projects/SNP/snp_ref.cgi?rs=rs78613025) | [14:92174423](genomebrowse://api/zoom?locus=14:92174423) | 0.0417332 | - | 0.0410848 | - | intron_variant | - | - | - |
| *CATSPERD* | Cation Channel Sperm Associated Auxiliary Subunit Delta | [rs73544757](http://www.ncbi.nlm.nih.gov/projects/SNP/snp_ref.cgi?rs=rs73544757) | [19:5733874](genomebrowse://api/zoom?locus=19:5733874) | 0.0642971 | 0.0292863 | 0.0366279 | 0.029 | missense_variant | Damaging | Benign | Tolerated |
| *CATSPERD* | Cation Channel Sperm Associated Auxiliary Subunit Delta | [rs73544759](http://www.ncbi.nlm.nih.gov/projects/SNP/snp_ref.cgi?rs=rs73544759) | [19:5733908](genomebrowse://api/zoom?locus=19:5733908) | 0.0654952 | 0.0301681 | 0.0376139 | 0.03 | synonymous_variant | - | - | - |
| *CATSPERD* | Cation Channel Sperm Associated Auxiliary Subunit Delta | [rs57680462](http://www.ncbi.nlm.nih.gov/projects/SNP/snp_ref.cgi?rs=rs57680462) | [19:5744499](genomebrowse://api/zoom?locus=19:5744499) | 0.0617013 | 0.0189536 | 0.0407438 | 0.022 | missense_variant | Damaging | Benign | Tolerated |
| *CBFA2T2* | CBFA2/RUNX1 Partner Transcriptional Co-Repressor 2 | rs143207038 | 20:32199069 | 0.000199681 | 0.000609588 | 0.000419951 | 0.000626 | synonymous_variant | - | - | - |
| *CBFB* | Core-Binding Factor Subunit Beta | [rs79560117](http://www.ncbi.nlm.nih.gov/projects/SNP/snp_ref.cgi?rs=rs79560117) | [16:67063612](genomebrowse://api/zoom?locus=16:67063612) | 0.0595048 | 0.047403 | 0.0665089 | 0.049 | intron_variant | - | - | - |
| *CBL* | Cbl Proto-Oncogene | rs34732429 | 11:119149351 | 0.00259585 | 0.000438828 | 0.0015834 | 0.0004942 | synonymous_variant | - | - | - |
| *CBR3* | Carbonyl Reductase 3 | [rs17849671](http://www.ncbi.nlm.nih.gov/projects/SNP/snp_ref.cgi?rs=rs17849671) | [21:37518582](genomebrowse://api/zoom?locus=21:37518582) | 0.0219649 | 0.0436061 | 0.0395528 | 0.044 | synonymous_variant | - | - | - |
| *CBX2* | Chromobox 2 | [rs8080971](http://www.ncbi.nlm.nih.gov/projects/SNP/snp_ref.cgi?rs=rs8080971) | [17:77757845](genomebrowse://api/zoom?locus=17:77757845) | 0.058107 | 0.0147526 | 0.0512763 | 0.018 | synonymous_variant | - | - | - |
| *CCAR1* | Cell Division Cycle And Apoptosis Regulator 1 | [rs113255973](http://www.ncbi.nlm.nih.gov/projects/SNP/snp_ref.cgi?rs=rs113255973) | [10:70482238](genomebrowse://api/zoom?locus=10:70482238) | 0.0179712 | 0.0303121 | 0.0212409 | 0.029 | 5_prime_UTR_variant | Damaging | - | Tolerated |
| *CCAR2* | Cell Cycle And Apoptosis Regulator 2 | rs75182495 | 8:22476754 | 0.00459265 | 0.00849377 | 0.00771914 | 0.006663 | synonymous_variant | - | - | - |
| *CCDC104* | Coiled-Coil Domain Containing 104 | [rs1045920](http://www.ncbi.nlm.nih.gov/projects/SNP/snp_ref.cgi?rs=rs1045920) | [2:55771169](genomebrowse://api/zoom?locus=2:55771169) | 0.148762 | 0.0748605 | 0.139588 | 0.084 | missense_variant | Tolerated | Benign | Tolerated |
| *CCDC108* | Coiled-Coil Domain Containing 108 | [rs3736403](http://www.ncbi.nlm.nih.gov/projects/SNP/snp_ref.cgi?rs=rs3736403) | [2:219905247](genomebrowse://api/zoom?locus=2:219905247) | 0.114217 | 0.103034 | 0.108191 | 0.083 | missense_variant | Tolerated | Benign | Tolerated |
| *CCDC114* | Coiled-Coil Domain Containing 114 | [rs16981988](http://www.ncbi.nlm.nih.gov/projects/SNP/snp_ref.cgi?rs=rs16981988) | [19:48821757](genomebrowse://api/zoom?locus=19:48821757) | 0.0319489 | 0.0257573 | 0.026699 | 0.029 | missense_variant | Damaging | Probably damaging | Damaging |
| *CCDC116* | Coiled-Coil Domain Containing 116 | [rs730882](http://www.ncbi.nlm.nih.gov/projects/SNP/snp_ref.cgi?rs=rs730882) | [22:21989325](genomebrowse://api/zoom?locus=22:21989325) | 0.0577077 | 0.0300292 | 0.0523504 | 0.032 | missense_variant | Damaging | Possibly damaging | Tolerated |
| *CCDC129* | Coiled-Coil Domain Containing 129 | [rs2286711](http://www.ncbi.nlm.nih.gov/projects/SNP/snp_ref.cgi?rs=rs2286711) | [7:31594508](genomebrowse://api/zoom?locus=7:31594508) | 0.228834 | 0.225858 | 0.233034 | 0.202 | synonymous_variant | - | - | - |
| *CCDC129* | Coiled-Coil Domain Containing 129 | [rs7799540](http://www.ncbi.nlm.nih.gov/projects/SNP/snp_ref.cgi?rs=rs7799540) | [7:31690831](genomebrowse://api/zoom?locus=7:31690831) | 0.0489217 | 0.011284 | 0.0417744 | 0.014 | missense_variant | Damaging | Probably damaging | Tolerated |
| *CCDC132* | Coiled-Coil Domain Containing 132 | [rs750985350](http://www.ncbi.nlm.nih.gov/projects/SNP/snp_ref.cgi?rs=rs750985350) | [7:92900513](genomebrowse://api/zoom?locus=7:92900513) | - | 0.152541 | 0.064305 | 0.143 | intron_variant | - | - | - |
| *CCDC138* | Coiled-Coil Domain Containing 138 | [rs35794776](http://www.ncbi.nlm.nih.gov/projects/SNP/snp_ref.cgi?rs=rs35794776) | [2:109408159](genomebrowse://api/zoom?locus=2:109408159) | 0.0720847 | 0.0584063 | 0.050126 | 0.062 | missense_variant | Damaging | Benign | Tolerated |
| *CCDC144CP* | Coiled-Coil Domain Containing 144C, Pseudogene | rs540490614 | 17:20242989 | 0.000399361 | 0.000259212 | 0.000261986 | 4.838e-05 | non_coding_exon_variant | - | - | - |
| *CCDC146* | Coiled-Coil Domain Containing 146 | [rs17230060](http://www.ncbi.nlm.nih.gov/projects/SNP/snp_ref.cgi?rs=rs17230060) | [7:76871113](genomebrowse://api/zoom?locus=7:76871113) | 0.0704872 | 0.11418 | 0.105182 | 0.116 | synonymous_variant | - | - | - |
| *CCDC147* | Coiled-Coil Domain Containing 147 | rs146524252 | 10:106124620 | 0.000199681 | 0.00143686 | 0.0015177 | 0.001508 | synonymous_variant | - | - | - |
| *CCDC152* | Coiled-Coil Domain Containing 152 | rs28919922 | 5:42801278 | 0.00898562 | 0.00200927 | 0.00784782 | 0.002344 | synonymous_variant | - | - | Tolerated |
| *CCDC157* | Coiled-Coil Domain Containing 157 | [rs41281641](http://www.ncbi.nlm.nih.gov/projects/SNP/snp_ref.cgi?rs=rs41281641) | [22:30762139](genomebrowse://api/zoom?locus=22:30762139) | 0.0599042 | 0.0713582 | 0.0886043 | 0.073 | synonymous_variant | - | - | - |
| *CCDC157* | Coiled-Coil Domain Containing 157 | [rs41281643](http://www.ncbi.nlm.nih.gov/projects/SNP/snp_ref.cgi?rs=rs41281643) | [22:30762178](genomebrowse://api/zoom?locus=22:30762178) | 0.0233626 | 0.0209108 | 0.0254511 | 0.021 | synonymous_variant | - | - | - |
| *CCDC168* | Coiled-Coil Domain Containing 168 | rs141879232 | 13:103390384 | 0.00239617 | 0.00358787 | 0.00239715 | 0.003827 | synonymous_variant | - | - | - |
| *CCDC168* | Coiled-Coil Domain Containing 168 | [rs17507841](http://www.ncbi.nlm.nih.gov/projects/SNP/snp_ref.cgi?rs=rs17507841) | [13:103388015](genomebrowse://api/zoom?locus=13:103388015) | 0.0195687 | 0.0290968 | 0.0196586 | 0.034 | missense_variant | - | Benign | Tolerated |
| *CCDC168* | Coiled-Coil Domain Containing 168 | [rs17592459](http://www.ncbi.nlm.nih.gov/projects/SNP/snp_ref.cgi?rs=rs17592459) | [13:103388880](genomebrowse://api/zoom?locus=13:103388880) | 0.024361 | 0.0363864 | 0.0234537 | 0.047 | missense_variant | - | Benign | Tolerated |
| *CCDC168* | Coiled-Coil Domain Containing 168 | [rs74435572](http://www.ncbi.nlm.nih.gov/projects/SNP/snp_ref.cgi?rs=rs74435572) | [13:103392127](genomebrowse://api/zoom?locus=13:103392127) | 0.067492 | 0.0955099 | 0.0845645 | 0.106 | missense_variant | - | - | - |
| *CCDC168* | Coiled-Coil Domain Containing 168 | [rs79592880](http://www.ncbi.nlm.nih.gov/projects/SNP/snp_ref.cgi?rs=rs79592880) | [13:103392709](genomebrowse://api/zoom?locus=13:103392709) | 0.0882588 | 0.103584 | 0.10109 | 0.123 | missense_variant | - | - | - |
| *CCDC168* | Coiled-Coil Domain Containing 168 | [rs75285783](http://www.ncbi.nlm.nih.gov/projects/SNP/snp_ref.cgi?rs=rs75285783) | [13:103397890](genomebrowse://api/zoom?locus=13:103397890) | 0.0748802 | 0.0965749 | 0.0896291 | 0.108 | synonymous_variant | - | - | - |
| *CCDC171* | Coiled-Coil Domain Containing 171 | rs191749178 | 9:15587692 | 0.00219649 | 0.00333611 | 0.00280863 | 0.00272 | intron_variant | - | - | - |
| *CCDC171* | Coiled-Coil Domain Containing 171 | rs537403155 | 9:15587693 | 0.00219649 | 0.00332849 | 0.00280917 | 0.002721 | intron_variant | - | - | - |
| *CCDC171* | Coiled-Coil Domain Containing 171 | [rs34816651](http://www.ncbi.nlm.nih.gov/projects/SNP/snp_ref.cgi?rs=rs34816651) | [9:15744683](genomebrowse://api/zoom?locus=9:15744683) | 0.0986422 | 0.0423277 | 0.0412954 | 0.045 | missense_variant | Tolerated | Benign | Damaging |
| *CCDC175* | Coiled-Coil Domain Containing 175 | [rs112952088](http://www.ncbi.nlm.nih.gov/projects/SNP/snp_ref.cgi?rs=rs112952088) | [14:60027907](genomebrowse://api/zoom?locus=14:60027907) | 0.0223642 | 0.0380829 | 0.0468639 | 0.032 | missense_variant | Tolerated | - | Tolerated |
| *CCDC18* | Coiled-Coil Domain Containing 18 | [rs2255722](http://www.ncbi.nlm.nih.gov/projects/SNP/snp_ref.cgi?rs=rs2255722) | [1:93646433](genomebrowse://api/zoom?locus=1:93646433) | 0.145767 | 0.135037 | 0.090974 | 0.128 | 5_prime_UTR_variant | - | - | - |
| *CCDC19* | Coiled-Coil Domain Containing 19 | [rs41264831](http://www.ncbi.nlm.nih.gov/projects/SNP/snp_ref.cgi?rs=rs41264831) | [1:159863028](genomebrowse://api/zoom?locus=1:159863028) | 0.053115 | 0.0184731 | 0.0399341 | 0.02 | missense_variant | Damaging | Benign | Damaging |
| *CCDC27* | Coiled-Coil Domain Containing 27 | rs41315312 | 1:3669172 | 0.0778754 | 0.0747791 | 0.0616483 | 0.073 | missense_variant | Tolerated | Benign | Tolerated |
| *CCDC30* | Coiled-Coil Domain Containing 30 | [rs16829829](http://www.ncbi.nlm.nih.gov/projects/SNP/snp_ref.cgi?rs=rs16829829) | [1:43119661](genomebrowse://api/zoom?locus=1:43119661) | 0.0746805 | 0.0423988 | 0.0654858 | 0.045 | missense_variant | Damaging | Benign | Tolerated |
| *CCDC38* | Coiled-Coil Domain Containing 38 | rs141524716 | 12:96263275 | 0.00559105 | 0.00173358 | 0.00649015 | 0.002331 | missense_variant | Damaging | Benign | Damaging |
| *CCDC38* | Coiled-Coil Domain Containing 38 | [rs56236436](http://www.ncbi.nlm.nih.gov/projects/SNP/snp_ref.cgi?rs=rs56236436) | [12:96263376](genomebrowse://api/zoom?locus=12:96263376) | 0.0319489 | 0.0544077 | 0.0536904 | 0.055 | intron_variant | - | - | - |
| *CCDC39* | Coiled-Coil Domain Containing 39 | rs183413880 | 3:180369283 | 0.00239617 | 0.00373008 | 0.00223388 | 0.001572 | missense_variant | Tolerated | Benign | Tolerated |
| *CCDC40* | Coiled-Coil Domain Containing 40 | [rs2289531](http://www.ncbi.nlm.nih.gov/projects/SNP/snp_ref.cgi?rs=rs2289531) | [17:78023722](genomebrowse://api/zoom?locus=17:78023722) | 0.11222 | 0.0990033 | 0.108545 | 0.1 | synonymous_variant | - | - | - |
| *CCDC57* | Coiled-Coil Domain Containing 57 | [rs11653662](http://www.ncbi.nlm.nih.gov/projects/SNP/snp_ref.cgi?rs=rs11653662) | [17:80071450](genomebrowse://api/zoom?locus=17:80071450) | 0.101038 | 0.167829 | 0.163776 | 0.148 | intron_variant | - | - | Damaging |
| *CCDC60* | Coiled-Coil Domain Containing 60 | [rs78218795](http://www.ncbi.nlm.nih.gov/projects/SNP/snp_ref.cgi?rs=rs78218795) | [12:119961587](genomebrowse://api/zoom?locus=12:119961587) | 0.0311502 | 0.0189109 | 0.0332515 | 0.021 | missense_variant | Damaging | Probably damaging | Damaging |
| *CCDC7* | Coiled-Coil Domain Containing 7 | [rs56391924](http://www.ncbi.nlm.nih.gov/projects/SNP/snp_ref.cgi?rs=rs56391924) | [10:32745248](genomebrowse://api/zoom?locus=10:32745248) | 0.140974 | 0.12521 | 0.0739628 | 0.121 | missense_variant | Tolerated | Benign | Tolerated |
| *CCDC7* | Coiled-Coil Domain Containing 7 | [rs12268559](http://www.ncbi.nlm.nih.gov/projects/SNP/snp_ref.cgi?rs=rs12268559) | [10:32856746](genomebrowse://api/zoom?locus=10:32856746) | 0.165335 | 0.131668 | 0.0927375 | 0.127 | missense_variant | Tolerated | Benign | Tolerated |
| *CCDC73* | Coiled-Coil Domain Containing 73 | rs117530589 | 11:32781773 | 0.00359425 | 0.00527588 | 0.00500614 | 0.00487 | missense_variant | Tolerated | Benign | Tolerated |
| *CCDC81* | Coiled-Coil Domain Containing 81 | [rs17210546](http://www.ncbi.nlm.nih.gov/projects/SNP/snp_ref.cgi?rs=rs17210546) | [11:86103767](genomebrowse://api/zoom?locus=11:86103767) | 0.0543131 | 0.0359901 | 0.012362 | 0.021 | synonymous_variant | - | - | - |
| *CCDC83* | Coiled-Coil Domain Containing 83 | [rs3213934](http://www.ncbi.nlm.nih.gov/projects/SNP/snp_ref.cgi?rs=rs3213934) | [11:85627039](genomebrowse://api/zoom?locus=11:85627039) | 0.111022 | 0.1306 | 0.128747 | 0.129 | intron_variant | - | - | - |
| *CCIN* | Calicin | rs75354691 | 9:36169517 | 0.000798722 | 0.000446704 | 0.00216255 | 0.0006177 | synonymous_variant | - | - | - |
| *CCIN* | Calicin | [rs34789048](http://www.ncbi.nlm.nih.gov/projects/SNP/snp_ref.cgi?rs=rs34789048) | [9:36169723](genomebrowse://api/zoom?locus=9:36169723) | 0.0555112 | 0.0703912 | 0.0821542 | 0.071 | missense_variant | Tolerated | Benign | Tolerated |
| *CCIN* | Calicin | [rs113985677](http://www.ncbi.nlm.nih.gov/projects/SNP/snp_ref.cgi?rs=rs113985677) | [9:36170290](genomebrowse://api/zoom?locus=9:36170290) | 0.0165735 | 0.0301137 | 0.0281276 | 0.03 | missense_variant | Tolerated | Benign | Tolerated |
| *CCM2L* | CCM2 Like Scaffold Protein | [rs6089151](http://www.ncbi.nlm.nih.gov/projects/SNP/snp_ref.cgi?rs=rs6089151) | [20:30616835](genomebrowse://api/zoom?locus=20:30616835) | 0.01877 | 0.0363632 | 0.0403502 | 0.036 | synonymous_variant | Tolerated | - | Damaging |
| *CCNB2* | Cyclin B2 | rs140527154 | 15:59399630 | 0.000199681 | 6.09276e-05 | 0.000290904 | 9.06e-05 | missense_variant | Tolerated | Benign | Damaging |
| *CCND2* | Cyclin D2 | rs142170178 | 12:4409090 | 0.000998403 | 0.00116196 | 0.0013888 | 0.00112 | missense_variant | Tolerated | Benign | Damaging |
| *CCNE2* | Cyclin E2 | [rs2467670](http://www.ncbi.nlm.nih.gov/projects/SNP/snp_ref.cgi?rs=rs2467670) | [8:95896106](genomebrowse://api/zoom?locus=8:95896106) | 0.0433307 | 0.0506569 | 0.0435471 | 0.048 | intron_variant | - | - | - |
| *CCNJL* | Cyclin J Like | rs377173221 | 5:159686633 | 0.000798722 | 0.00020308 | 0.000937177 | 0.0002808 | synonymous_variant | - | - | - |
| *CCR7* | C-C Motif Chemokine Receptor 7 | [rs2229095](http://www.ncbi.nlm.nih.gov/projects/SNP/snp_ref.cgi?rs=rs2229095) | [17:38711222](genomebrowse://api/zoom?locus=17:38711222) | 0.0595048 | 0.0246855 | 0.0595338 | 0.028 | synonymous_variant | - | - | - |
| *CCRL2* | C-C Motif Chemokine Receptor Like 2 | [rs11574440](http://www.ncbi.nlm.nih.gov/projects/SNP/snp_ref.cgi?rs=rs11574440) | [3:46449164](genomebrowse://api/zoom?locus=3:46449164) | 0.0555112 | 0.0106235 | 0.054424 | 0.017 | initiator_codon_variant | Tolerated | Benign | Tolerated |
| *CCRL2* | C-C Motif Chemokine Receptor Like 2 | [rs11574441](http://www.ncbi.nlm.nih.gov/projects/SNP/snp_ref.cgi?rs=rs11574441) | [3:46449175](genomebrowse://api/zoom?locus=3:46449175) | 0.0704872 | 0.0131935 | 0.0668714 | 0.021 | missense_variant | Tolerated | Benign | Tolerated |
| *CCRN4L* | Carbon Catabolite Repression 4-Like Protein | [rs61731324](http://www.ncbi.nlm.nih.gov/projects/SNP/snp_ref.cgi?rs=rs61731324) | [4:139964337](genomebrowse://api/zoom?locus=4:139964337) | 0.0662939 | 0.019553 | 0.0440587 | 0.022 | synonymous_variant | - | - | - |
| *CCSER1* | Coiled-Coil Serine Rich Protein 1 | rs72659490 | 4:91230480 | 0.00399361 | 0.00680344 | 0.00562016 | 0.006605 | missense_variant | Tolerated | Benign | Tolerated |
| *CCT2* | Chaperonin Containing TCP1 Subunit 2 | rs369840085 | 12:69986814 | 0.000399361 | 8.13068e-05 | 3.25119e-05 | 9.884e-05 | missense_variant | Damaging | Probably damaging | Damaging |
| *CCT5* | Chaperonin Containing TCP1 Subunit 5 | rs554376123 | 5:10250393 | 0.00239617 | 0.000647029 | 0.00154929 | 0.000758 | 5_prime_UTR_variant | - | - | - |
| *CCT5* | Chaperonin Containing TCP1 Subunit 5 | rs142127415 | 5:10263278 | 0.00199681 | 0.000438668 | 0.00138835 | 0.0006095 | synonymous_variant | - | - | - |
| *CCT5* | Chaperonin Containing TCP1 Subunit 5 | [rs10474867](http://www.ncbi.nlm.nih.gov/projects/SNP/snp_ref.cgi?rs=rs10474867) | [5:10262770](genomebrowse://api/zoom?locus=5:10262770) | 0.0459265 | 0.0106491 | 0.0416021 | 0.013 | intron_variant | - | - | - |
| *CCT6A* | Chaperonin Containing TCP1 Subunit 6A | [rs33922584](http://www.ncbi.nlm.nih.gov/projects/SNP/snp_ref.cgi?rs=rs33922584) | [7:56125757](genomebrowse://api/zoom?locus=7:56125757) | 0.0491214 | 0.0375824 | 0.0347868 | 0.036 | missense_variant | Damaging | Benign | Damaging |
| *CCT7* | Chaperonin Containing TCP1 Subunit 7 | [rs41285977](http://www.ncbi.nlm.nih.gov/projects/SNP/snp_ref.cgi?rs=rs41285977) | [2:73461553](genomebrowse://api/zoom?locus=2:73461553) | 0.0365415 | 0.0576818 | 0.07093 | 0.059 | intron_variant | - | - | - |
| *CCZ1* | CCZ1 Homolog, Vacuolar Protein Trafficking And Biogenesis Associated | [rs71524059](http://www.ncbi.nlm.nih.gov/projects/SNP/snp_ref.cgi?rs=rs71524059) | [7:5942269](genomebrowse://api/zoom?locus=7:5942269) | 0.264577 | 0.1133 | 0.159438 | 0.17 | intron_variant | - | - | - |
| *CCZ1B* | CCZ1 Homolog B, Vacuolar Protein Trafficking And Biogenesis Associated | [rs80105188](http://www.ncbi.nlm.nih.gov/projects/SNP/snp_ref.cgi?rs=rs80105188) | [7:6838915](genomebrowse://api/zoom?locus=7:6838915) | - | 0.0274962 | 0.0117025 | 0.021 | splice_region_variant | - | - | - |
| *CD109* | CD109 Molecule | rs114463962 | 6:74502308 | 0.00299521 | 0.000717809 | 0.00284091 | 0.0009307 | intron_variant | - | - | - |
| *CD1A* | CD1a Molecule | [rs2269714](http://www.ncbi.nlm.nih.gov/projects/SNP/snp_ref.cgi?rs=rs2269714) | [1:158224904](genomebrowse://api/zoom?locus=1:158224904) | 0.164337 | 0.110432 | 0.0749208 | 0.11 | missense_variant | Tolerated | Benign | Tolerated |
| *CD1A* | CD1a Molecule | [rs2269715](http://www.ncbi.nlm.nih.gov/projects/SNP/snp_ref.cgi?rs=rs2269715) | [1:158225019](genomebrowse://api/zoom?locus=1:158225019) | 0.164337 | 0.110458 | 0.0752052 | 0.11 | missense_variant | Tolerated | Benign | Tolerated |
| *CD1B* | CD1b Molecule | [rs57016335](http://www.ncbi.nlm.nih.gov/projects/SNP/snp_ref.cgi?rs=rs57016335) | [1:158299766](genomebrowse://api/zoom?locus=1:158299766) | 0.0567093 | 0.0149622 | 0.0468932 | 0.019 | synonymous_variant | - | - | - |
| *CD1C* | CD1c Molecule | [rs3138099](http://www.ncbi.nlm.nih.gov/projects/SNP/snp_ref.cgi?rs=rs3138099) | [1:158261015](genomebrowse://api/zoom?locus=1:158261015) | 0.0601038 | 0.0448904 | 0.0750535 | 0.048 | synonymous_variant | - | - | - |
| *CD1E* | CD1e Molecule | [rs61734681](http://www.ncbi.nlm.nih.gov/projects/SNP/snp_ref.cgi?rs=rs61734681) | [1:158326581](genomebrowse://api/zoom?locus=1:158326581) | 0.057508 | 0.0132887 | 0.0538063 | 0.016 | synonymous_variant | - | - | - |
| *CD1E* | CD1e Molecule | [rs61734680](http://www.ncbi.nlm.nih.gov/projects/SNP/snp_ref.cgi?rs=rs61734680) | [1:158326645](genomebrowse://api/zoom?locus=1:158326645) | 0.0395367 | 0.010064 | 0.0405545 | 0.012 | missense_variant | Tolerated | Benign | Tolerated |
| *CD200R1* | CD200 Receptor 1 | [rs72491121](http://www.ncbi.nlm.nih.gov/projects/SNP/snp_ref.cgi?rs=rs72491121) | [3:112643952](genomebrowse://api/zoom?locus=3:112643952) | 0.0832668 | 0.0541765 | 0.0398797 | 0.055 | synonymous_variant | - | - | - |
| *CD207* | CD207 Molecule | [rs57302492](http://www.ncbi.nlm.nih.gov/projects/SNP/snp_ref.cgi?rs=rs57302492) | [2:71058230](genomebrowse://api/zoom?locus=2:71058230) | 0.129593 | 0.0698547 | 0.0814262 | 0.073 | missense_variant | Tolerated | Benign | Tolerated |
| *CD22* | CD22 Molecule | [rs25677](http://www.ncbi.nlm.nih.gov/projects/SNP/snp_ref.cgi?rs=rs25677) | [19:35831986](genomebrowse://api/zoom?locus=19:35831986) | 0.0587061 | 0.0201394 | 0.0536407 | 0.023 | synonymous_variant | - | - | - |
| *CD247* | CD247 Molecule | [rs33937946](http://www.ncbi.nlm.nih.gov/projects/SNP/snp_ref.cgi?rs=rs33937946) | [1:167407858](genomebrowse://api/zoom?locus=1:167407858) | 0.0595048 | 0.0381011 | 0.0666365 | 0.041 | synonymous_variant | - | - | - |
| *CD300LF* | CD300 Molecule Like Family Member F | rs61738885 | 17:72701356 | 0.00359425 | 0.000961728 | 0.00442506 | 0.0009051 | synonymous_variant | - | - | - |
| *CD300LF* | CD300 Molecule Like Family Member F | [rs59859657](http://www.ncbi.nlm.nih.gov/projects/SNP/snp_ref.cgi?rs=rs59859657) | [17:72691885](genomebrowse://api/zoom?locus=17:72691885) | 0.0712859 | 0.0302305 | 0.0425828 | 0.031 | synonymous_variant | - | - | - |
| *CD44* | CD44 Molecule (Indian Blood Group) | rs11607491 | 11:35226083 | 0.00359425 | 0.00768348 | 0.00600659 | 0.007273 | missense_variant | Tolerated | Benign | Tolerated |
| *CD48* | CD48 Molecule | [rs11541827](http://www.ncbi.nlm.nih.gov/projects/SNP/snp_ref.cgi?rs=rs11541827) | [1:160681508](genomebrowse://api/zoom?locus=1:160681508) | 0.0183706 | 0.0206154 | 0.0150359 | 0.022 | synonymous_variant | - | - | - |
| *CD58* | CD58 Molecule | [rs35768283](http://www.ncbi.nlm.nih.gov/projects/SNP/snp_ref.cgi?rs=rs35768283) | [1:117078729](genomebrowse://api/zoom?locus=1:117078729) | 0.0778754 | 0.0313452 | 0.0681334 | 0.035 | synonymous_variant | - | - | - |
| *CD83* | CD83 Molecule | [rs35118414](http://www.ncbi.nlm.nih.gov/projects/SNP/snp_ref.cgi?rs=rs35118414) | [6:14131854](genomebrowse://api/zoom?locus=6:14131854) | 0.038738 | 0.0379515 | 0.0459484 | 0.04 | missense_variant | Tolerated | Benign | Tolerated |
| *CD8A* | CD8a Molecule | [rs7563485](http://www.ncbi.nlm.nih.gov/projects/SNP/snp_ref.cgi?rs=rs7563485) | [2:87012706](genomebrowse://api/zoom?locus=2:87012706) | 0.0479233 | 0.05631 | 0.0838343 | 0.026 | 3_prime_UTR_variant | - | - | - |
| *CDAN1* | Codanin 1 | rs77191722 | 15:43016707 | 0.00239617 | 0.000612989 | 0.00326017 | 0.000774 | synonymous_variant | - | - | - |
| *CDAN1* | Codanin 1 | rs61745955 | 15:43027745 | 0.0061901 | 0.00114257 | 0.00458656 | 0.001532 | synonymous_variant | - | - | - |
| *CDAN1* | Codanin 1 | c.3551G>A | [15:43017349](genomebrowse://api/zoom?locus=15:43017349) | - | - | - | - | stop_gained | - | - | Damaging |
| *CDAN1* | Codanin 1 | [rs28645106](http://www.ncbi.nlm.nih.gov/projects/SNP/snp_ref.cgi?rs=rs28645106) | [15:43023304](genomebrowse://api/zoom?locus=15:43023304) | 0.196086 | 0.0824677 | 0.119944 | 0.088 | intron_variant | - | - | - |
| *CDC27* | Cell Division Cycle 27 | [rs731790](http://www.ncbi.nlm.nih.gov/projects/SNP/snp_ref.cgi?rs=rs731790) | [17:45198343](genomebrowse://api/zoom?locus=17:45198343) | 0.0567093 | 0.0733108 | 0.085935 | 0.071 | synonymous_variant | - | - | - |
| *CDC37L1* | Cell Division Cycle 37 Like 1 | [rs2295967](http://www.ncbi.nlm.nih.gov/projects/SNP/snp_ref.cgi?rs=rs2295967) | [9:4685008](genomebrowse://api/zoom?locus=9:4685008) | 0.179513 | 0.129126 | 0.101914 | 0.131 | synonymous_variant | - | - | - |
| *CDC42BPA* | CDC42 Binding Protein Kinase Alpha | rs112760723 | 1:227239626 | 0.00359425 | 0.000860809 | 0.00335896 | 0.0009308 | intron_variant | - | Possibly damaging | Damaging |
| *CDC42BPA* | CDC42 Binding Protein Kinase Alpha | rs181994567 | 1:227387209 | 0.000199681 | 0.000124599 | 0.000452401 | 0.000173 | intron_variant | - | - | - |
| *CDC42BPB* | CDC42 Binding Protein Kinase Beta | rs34750098 | 14:103447257 | 0.00299521 | 0.00612331 | 0.00506844 | 0.005164 | synonymous_variant | - | - | - |
| *CDCA7L* | Cell Division Cycle Associated 7 Like | [rs10238000](http://www.ncbi.nlm.nih.gov/projects/SNP/snp_ref.cgi?rs=rs10238000) | [7:21985383](genomebrowse://api/zoom?locus=7:21985383) | 0.0591054 | 0.0124878 | 0.0514116 | 0.017 | intron_variant | - | - | - |
| *CDH12* | Cadherin 12 | rs143459013 | 5:21760715 | 0.000998403 | 0.000199703 | 0.000549451 | 0.0002471 | missense_variant | Tolerated | Benign | Damaging |
| *CDH20* | Cadherin 20 | rs17068463 | 18:59195354 | 0.00658946 | 0.00153972 | 0.00562161 | 0.001919 | missense_variant | Tolerated | Benign | Damaging |
| *CDH23* | Cadherin Related 23 | [rs1227049](http://www.ncbi.nlm.nih.gov/projects/SNP/snp_ref.cgi?rs=rs1227049) | [10:73434888](genomebrowse://api/zoom?locus=10:73434888) | 0.185304 | 0.201184 | 0.161969 | 0.199 | missense_variant | - | Probably damaging | Damaging |
| *CDH23* | Cadherin Related 23 | [rs1045482647](http://www.ncbi.nlm.nih.gov/projects/SNP/snp_ref.cgi?rs=rs1045482647) | [10:73468882](genomebrowse://api/zoom?locus=10:73468882) | - | - | - | - | missense_variant | Damaging | Possibly damaging | Damaging |
| *CDH26* | Cadherin 26 | rs73916223 | 20:58571606 | 0.00778754 | 0.00154024 | 0.00500517 | 0.001861 | 5_prime_UTR_variant | - | - | - |
| *CDH26* | Cadherin 26 | [rs41314912](http://www.ncbi.nlm.nih.gov/projects/SNP/snp_ref.cgi?rs=rs41314912) | [20:58576449](genomebrowse://api/zoom?locus=20:58576449) | 0.0135783 | 0.0164992 | 0.0131095 | 0.017 | missense_variant | Tolerated | Benign | Tolerated |
| *CDH3* | Cadherin 3 | rs201352240 | 16:68732174 | 0.000199681 | 0.000154313 | 0.000225981 | 0.0001565 | synonymous_variant | - | - | - |
| *CDH9* | Cadherin 9 | rs147997829 | 5:26885795 | 0.000399361 | 0.000688554 | 0.000516996 | 0.0007332 | missense_variant | Damaging | Probably damaging | Damaging |
| *CDH9* | Cadherin 9 | rs111992148 | 5:26903653 | 0.00998403 | 0.00201322 | 0.00666192 | 0.002251 | intron_variant | - | - | - |
| *CDHR3* | Cadherin Related Family Member 3 | [rs6967330](http://www.ncbi.nlm.nih.gov/projects/SNP/snp_ref.cgi?rs=rs6967330) | [7:105658451](genomebrowse://api/zoom?locus=7:105658451) | 0.184704 | 0.18604 | 0.203219 | 0.189 | missense_variant | Tolerated | Benign | Damaging |
| *CDHR3* | Cadherin Related Family Member 3 | [rs73195662](http://www.ncbi.nlm.nih.gov/projects/SNP/snp_ref.cgi?rs=rs73195662) | [7:105658460](genomebrowse://api/zoom?locus=7:105658460) | 0.0672923 | 0.07218 | 0.0607177 | 0.074 | missense_variant | Tolerated | Benign | Tolerated |
| *CDHR3* | Cadherin Related Family Member 3 | c.2273-892_2273-891insA | [7:105668106](genomebrowse://api/zoom?locus=7:105668106) | - | 0.134266 | 0.0476171 | 0.11 | intron_variant | - | - | - |
| *CDK11A* | Cyclin Dependent Kinase 11A | rs55699379 | 1:1643740 | 0.0259585 | 0.0987743 | 0.0424743 | 0.106 | synonymous_variant | - | - | - |
| *CDK12* | Cyclin Dependent Kinase 12 | rs56362165 | 17:37682375 | 0.00678914 | 0.00173505 | 0.00749112 | 0.002199 | missense_variant | Tolerated | Benign | Tolerated |
| *CDK12* | Cyclin Dependent Kinase 12 | [rs35896550](http://www.ncbi.nlm.nih.gov/projects/SNP/snp_ref.cgi?rs=rs35896550) | [17:37619038](genomebrowse://api/zoom?locus=17:37619038) | 0.058107 | 0.0136111 | 0.0552863 | 0.017 | synonymous_variant | - | - | - |
| *CDK16* | Cyclin Dependent Kinase 16 | rs727503843 | X:47082616 | 0.00397351 | 0.00137153 | 0.0051082 | 0.00186 | missense_variant | Tolerated | Benign | Tolerated |
| *CDK5RAP2* | CDK5 Regulatory Subunit Associated Protein 2 | [rs3780679](http://www.ncbi.nlm.nih.gov/projects/SNP/snp_ref.cgi?rs=rs3780679) | [9:123205912](genomebrowse://api/zoom?locus=9:123205912) | 0.0752796 | 0.0366097 | 0.0718207 | 0.041 | missense_variant | Tolerated | Benign | Tolerated |
| *CDK5RAP2* | CDK5 Regulatory Subunit Associated Protein 2 | [rs34523498](http://www.ncbi.nlm.nih.gov/projects/SNP/snp_ref.cgi?rs=rs34523498) | [9:123205981](genomebrowse://api/zoom?locus=9:123205981) | 0.0269569 | 0.0250126 | 0.0291648 | 0.026 | missense_variant | Damaging | Probably damaging | Damaging |
| *CDKAL1* | CDK5 Regulatory Subunit Associated Protein 1 Like 1 | [rs77152992](http://www.ncbi.nlm.nih.gov/projects/SNP/snp_ref.cgi?rs=rs77152992) | [6:21065449](genomebrowse://api/zoom?locus=6:21065449) | 0.0860623 | 0.0639369 | 0.0569637 | 0.065 | missense_variant | Damaging | Benign | Damaging |
| *CDKN1A* | Cyclin Dependent Kinase Inhibitor 1A | [rs1801270](http://www.ncbi.nlm.nih.gov/projects/SNP/snp_ref.cgi?rs=rs1801270) | [6:36651971](genomebrowse://api/zoom?locus=6:36651971) | 0.25619 | 0.152389 | 0.1458 | 0.146 | missense_variant | Tolerated | Benign | Damaging |
| *CDKN2A* | Cyclin Dependent Kinase Inhibitor 2A | rs200863613 | 9:21971060 | 0.000199681 | 8.64409e-05 | 0.000226142 | 0.000142 | missense_variant | Tolerated | Probably damaging | Tolerated |
| *CDKN3* | Cyclin Dependent Kinase Inhibitor 3 | rs147340627 | 14:54880330 | 0.0061901 | 0.00122633 | 0.00672139 | 0.0005647 | intron_variant | - | - | - |
| *CDNF* | Cerebral Dopamine Neurotrophic Factor | [rs61738953](http://www.ncbi.nlm.nih.gov/projects/SNP/snp_ref.cgi?rs=rs61738953) | [10:14862082](genomebrowse://api/zoom?locus=10:14862082) | 0.0135783 | 0.0309806 | 0.0295409 | 0.031 | missense_variant | Damaging | Probably damaging | Damaging |
| *CDRT4* | CMT1A Duplicated Region Transcript 4 | rs61742154 | 17:15341181 | 0.00898562 | 0.00192578 | 0.00823271 | 0.002578 | missense_variant | - | Benign | Tolerated |
| *CDSN* | Corneodesmosin | [rs1042127](http://www.ncbi.nlm.nih.gov/projects/SNP/snp_ref.cgi?rs=rs1042127) | [6:31084170](genomebrowse://api/zoom?locus=6:31084170) | 0.195887 | 0.208016 | 0.16565 | 0.201 | missense_variant | Tolerated | Possibly damaging | Tolerated |
| *CDYL* | Chromodomain Y Like | [rs734783](http://www.ncbi.nlm.nih.gov/projects/SNP/snp_ref.cgi?rs=rs734783) | [6:4892381](genomebrowse://api/zoom?locus=6:4892381) | 0.0499201 | 0.0120454 | 0.0464763 | 0.015 | synonymous_variant | - | - | - |
| *CEBPZ* | CCAAT Enhancer Binding Protein Zeta | [rs1803251](http://www.ncbi.nlm.nih.gov/projects/SNP/snp_ref.cgi?rs=rs1803251) | [2:37454998](genomebrowse://api/zoom?locus=2:37454998) | 0.0900559 | 0.0212663 | 0.0826444 | 0.027 | synonymous_variant | - | - | - |
| *CEL* | Carboxyl Ester Lipase | rs200157916 | 9:135940503 | 0.00239617 | 0.000458954 | 0.0016154 | 0.0005951 | synonymous_variant | - | - | - |
| *CELSR1* | Cadherin EGF LAG Seven-Pass G-Type Receptor 1 | [rs6008778](http://www.ncbi.nlm.nih.gov/projects/SNP/snp_ref.cgi?rs=rs6008778) | [22:46760102](genomebrowse://api/zoom?locus=22:46760102) | 0.0543131 | 0.0140917 | 0.0492365 | 0.017 | synonymous_variant | - | - | - |
| *CELSR1* | Cadherin EGF LAG Seven-Pass G-Type Receptor 1 | [rs11090875](http://www.ncbi.nlm.nih.gov/projects/SNP/snp_ref.cgi?rs=rs11090875) | [22:46785301](genomebrowse://api/zoom?locus=22:46785301) | 0.0716853 | 0.0726676 | 0.0911176 | 0.073 | synonymous_variant | - | - | - |
| *CELSR1* | Cadherin EGF LAG Seven-Pass G-Type Receptor 1 | [rs35717986](http://www.ncbi.nlm.nih.gov/projects/SNP/snp_ref.cgi?rs=rs35717986) | [22:46805007](genomebrowse://api/zoom?locus=22:46805007) | 0.11222 | 0.0624253 | 0.115565 | 0.066 | synonymous_variant | - | - | - |
| *CELSR1* | Cadherin EGF LAG Seven-Pass G-Type Receptor 1 | [rs3747251](http://www.ncbi.nlm.nih.gov/projects/SNP/snp_ref.cgi?rs=rs3747251) | [22:46860088](genomebrowse://api/zoom?locus=22:46860088) | 0.194489 | 0.0671161 | 0.147044 | 0.076 | synonymous_variant | - | - | - |
| *CELSR1* | Cadherin EGF LAG Seven-Pass G-Type Receptor 1 | c.3263A>C | [22:46929805](genomebrowse://api/zoom?locus=22:46929805) | - | - | - | - | missense_variant | Damaging | Probably damaging | Damaging |
| *CEMIP* | Cell Migration Inducing Hyaluronidase 1 | [rs35541581](http://www.ncbi.nlm.nih.gov/projects/SNP/snp_ref.cgi?rs=rs35541581) | [15:81172064](genomebrowse://api/zoom?locus=15:81172064) | 0.0712859 | 0.0178946 | 0.0640057 | 0.022 | synonymous_variant | - | - | - |
| *CEMIP* | Cell Migration Inducing Hyaluronidase 1 | [rs35092028](http://www.ncbi.nlm.nih.gov/projects/SNP/snp_ref.cgi?rs=rs35092028) | [15:81221492](genomebrowse://api/zoom?locus=15:81221492) | 0.0189696 | 0.0195806 | 0.0237849 | 0.02 | synonymous_variant | - | - | - |
| *CENPE* | Centromere Protein E | [rs61744934](http://www.ncbi.nlm.nih.gov/projects/SNP/snp_ref.cgi?rs=rs61744934) | [4:104081920](genomebrowse://api/zoom?locus=4:104081920) | 0.0740815 | 0.017086 | 0.0642154 | 0.021 | synonymous_variant | - | - | - |
| *CENPF* | Centromere Protein F | [rs753058863](http://www.ncbi.nlm.nih.gov/projects/SNP/snp_ref.cgi?rs=rs753058863) | [1:214818766](genomebrowse://api/zoom?locus=1:214818766) | - | - | - | - | synonymous_variant | - | - | - |
| *CENPJ* | Centromere Protein J | [rs9511510](http://www.ncbi.nlm.nih.gov/projects/SNP/snp_ref.cgi?rs=rs9511510) | [13:25486911](genomebrowse://api/zoom?locus=13:25486911) | 0.0609026 | 0.110388 | 0.103057 | 0.109 | missense_variant | Tolerated | Benign | Tolerated |
| *CENPN* | Centromere Protein N | c.938-16_938-15insT | [16:81066160](genomebrowse://api/zoom?locus=16:81066160) | - | 0.190057 | 0.0849215 | 0.051 | intron_variant | - | - | - |
| *CENPP* | Centromere Protein P | rs115116400 | 9:95179589 | 0.00938498 | 0.00199893 | 0.00794471 | 0.002389 | missense_variant | Damaging | Probably damaging | Damaging |
| *CEP152* | Centrosomal Protein 152 | rs113266652 | 15:49059226 | 0.00479233 | 0.000976034 | 0.00439333 | 0.0011 | intron_variant | - | - | - |
| *CEP152* | Centrosomal Protein 152 | rs199862615 | 15:49083481 | 0.00119808 | 0.000256485 | 0.00132437 | 0.0002732 | missense_variant | Damaging | Probably damaging | Tolerated |
| *CEP152* | Centrosomal Protein 152 | [rs77732888](http://www.ncbi.nlm.nih.gov/projects/SNP/snp_ref.cgi?rs=rs77732888) | [15:49088198](genomebrowse://api/zoom?locus=15:49088198) | 0.0415335 | 0.0712403 | 0.0674811 | 0.071 | intron_variant | - | - | - |
| *CEP170* | Centrosomal Protein 170 | [rs12047774](http://www.ncbi.nlm.nih.gov/projects/SNP/snp_ref.cgi?rs=rs12047774) | [1:243299436](genomebrowse://api/zoom?locus=1:243299436) | - | 0.0978182 | 0.0855092 | 0.053 | intron_variant | - | - | - |
| *CEP192* | Centrosomal Protein 192 | [rs2027698](http://www.ncbi.nlm.nih.gov/projects/SNP/snp_ref.cgi?rs=rs2027698) | [18:13092424](genomebrowse://api/zoom?locus=18:13092424) | 0.063099 | 0.0451766 | 0.0419734 | 0.047 | missense_variant | Tolerated | Benign | Tolerated |
| *CEP57L1* | Centrosomal Protein 57 Like 1 | rs372596886 | 6:109480445 | 0.00119808 | 9.7634e-05 | 0.000387272 | 9.061e-05 | intron_variant | Tolerated | - | Tolerated |
| *CEP68* | Centrosomal Protein 68 | [rs2723088](http://www.ncbi.nlm.nih.gov/projects/SNP/snp_ref.cgi?rs=rs2723088) | [2:65299298](genomebrowse://api/zoom?locus=2:65299298) | 0.0740815 | 0.100046 | 0.0971034 | 0.1 | missense_variant | Tolerated | Possibly damaging | Tolerated |
| *CEP68* | Centrosomal Protein 68 | [rs2723089](http://www.ncbi.nlm.nih.gov/projects/SNP/snp_ref.cgi?rs=rs2723089) | [2:65299300](genomebrowse://api/zoom?locus=2:65299300) | 0.0740815 | 0.100054 | 0.0971258 | 0.1 | missense_variant | Damaging | Possibly damaging | Damaging |
| *CEP70* | Centrosomal Protein 70 | [rs35340237](http://www.ncbi.nlm.nih.gov/projects/SNP/snp_ref.cgi?rs=rs35340237) | [3:138218994](genomebrowse://api/zoom?locus=3:138218994) | 0.195487 | 0.0800427 | 0.121187 | 0.085 | missense_variant | Tolerated | Benign | Tolerated |
| *CERCAM* | Cerebral Endothelial Cell Adhesion Molecule | [rs61732491](http://www.ncbi.nlm.nih.gov/projects/SNP/snp_ref.cgi?rs=rs61732491) | [9:131186783](genomebrowse://api/zoom?locus=9:131186783) | 0.0545128 | 0.0132889 | 0.0510541 | 0.017 | missense_variant | Tolerated | Benign | Tolerated |
| *CERCAM* | Cerebral Endothelial Cell Adhesion Molecule | [rs34878035](http://www.ncbi.nlm.nih.gov/projects/SNP/snp_ref.cgi?rs=rs34878035) | [9:131186856](genomebrowse://api/zoom?locus=9:131186856) | 0.052516 | 0.0129432 | 0.0496442 | 0.017 | synonymous_variant | - | - | - |
| *CERKL* | Ceramide Kinase Like | [rs61750041](http://www.ncbi.nlm.nih.gov/projects/SNP/snp_ref.cgi?rs=rs61750041) | [2:182468803](genomebrowse://api/zoom?locus=2:182468803) | 0.15595 | 0.147898 | 0.15111 | 0.14 | missense_variant | Damaging | Benign | Tolerated |
| *CERKL* | Ceramide Kinase Like | [rs11553356](http://www.ncbi.nlm.nih.gov/projects/SNP/snp_ref.cgi?rs=rs11553356) | [2:182401957](genomebrowse://api/zoom?locus=2:182401957) | 0.16873 | 0.134116 | 0.159818 | 0.048 | 3_prime_UTR_variant | - | - | - |
| *CERS3* | Ceramide Synthase 3 | [rs1566775](http://www.ncbi.nlm.nih.gov/projects/SNP/snp_ref.cgi?rs=rs1566775) | [15:101013123](genomebrowse://api/zoom?locus=15:101013123) | 0.0291534 | 0.0494453 | 0.0452459 | 0.048 | splice_region_variant | - | - | - |
| *CES1* | Carboxylesterase 1 | rs371537606 | 16:55850922 | 0.000199681 | 0.00192556 | 0.00161603 | 0.001903 | intron_variant | - | - | - |
| *CES1P1* | Carboxylesterase 1 Pseudogene 1 | rs201139605 | 16:55807350 | 0.00179712 | 0.00612787 | 0.00512963 | 0.005024 | non_coding_exon_variant | - | - | - |
| *CES3* | Carboxylesterase 3 | [rs3848289](http://www.ncbi.nlm.nih.gov/projects/SNP/snp_ref.cgi?rs=rs3848289) | [16:67006838](genomebrowse://api/zoom?locus=16:67006838) | 0.043131 | 0.0614976 | 0.0394932 | 0.057 | synonymous_variant | - | - | - |
| *CETN1* | Centrin 1 | rs114739741 | 18:580528 | 0.00978434 | 0.00549509 | 0.00245589 | 0.004645 | synonymous_variant | - | - | - |
| *CETP* | Cholesteryl Ester Transfer Protein | rs34680782 | 16:56996060 | 0.00319489 | 0.000747562 | 0.00388174 | 0.0009719 | intron_variant | - | - | - |
| *CETP* | Cholesteryl Ester Transfer Protein | [rs5883](http://www.ncbi.nlm.nih.gov/projects/SNP/snp_ref.cgi?rs=rs5883) | [16:57007353](genomebrowse://api/zoom?locus=16:57007353) | 0.0547125 | 0.0511931 | 0.0631178 | 0.054 | synonymous_variant | - | - | - |
| *CETP* | Cholesteryl Ester Transfer Protein | [rs1800777](http://www.ncbi.nlm.nih.gov/projects/SNP/snp_ref.cgi?rs=rs1800777) | [16:57017319](genomebrowse://api/zoom?locus=16:57017319) | 0.0263578 | 0.037451 | 0.0226876 | 0.035 | missense_variant | Tolerated | Benign | Tolerated |
| *CFB* | Complement Factor B | [rs4151651](http://www.ncbi.nlm.nih.gov/projects/SNP/snp_ref.cgi?rs=rs4151651) | [6:31915614](genomebrowse://api/zoom?locus=6:31915614) | 0.0101837 | 0.0221058 | 0.0241758 | 0.022 | missense_variant | Tolerated | Benign | Tolerated |
| *CFD* | Complement Factor D | rs193047313 | 19:861692 | 0.00319489 | 0.000859788 | 0.00359223 | 0.001036 | splice_region_variant | - | - | - |
| *CFH* | Complement Factor H | [rs515299](http://www.ncbi.nlm.nih.gov/projects/SNP/snp_ref.cgi?rs=rs515299) | [1:196706677](genomebrowse://api/zoom?locus=1:196706677) | 0.0623003 | 0.0163555 | 0.0569931 | 0.02 | missense_variant | Tolerated | Benign | Tolerated |
| *CFHR1* | Complement Factor H Related 1 | rs186492664 | 1:196799600 | 0.00179712 | 0.000309997 | 0.00115674 | 0.0004596 | intron_variant | - | - | - |
| *CFHR1* | Complement Factor H Related 1 | rs139136156 | 1:196801046 | 0.000798722 | 0.000249727 | 0.0010093 | 0.0003386 | missense_variant | Damaging | Benign | Tolerated |
| *CFHR4* | Complement Factor H Related 4 | c.1600A>G | [1:196887399](genomebrowse://api/zoom?locus=1:196887399) | - | - | - | - | missense_variant | Tolerated | Benign | Tolerated |
| *CFHR5* | Complement Factor H Related 5 | [rs57960694](http://www.ncbi.nlm.nih.gov/projects/SNP/snp_ref.cgi?rs=rs57960694) | [1:196963213](genomebrowse://api/zoom?locus=1:196963213) | 0.0407348 | 0.0108962 | 0.033706 | 0.013 | missense_variant | Tolerated | Benign | Tolerated |
| *CFHR5* | Complement Factor H Related 5 | [rs35662416](http://www.ncbi.nlm.nih.gov/projects/SNP/snp_ref.cgi?rs=rs35662416) | [1:196967354](genomebrowse://api/zoom?locus=1:196967354) | 0.0103834 | 0.0163255 | 0.0152731 | 0.016 | missense_variant | Tolerated | Possibly damaging | Tolerated |
| *CFI* | Complement Factor I | rs80173133 | 4:110662042 | 0.00878594 | 0.00211315 | 0.00859506 | 0.00266 | 3_prime_UTR_variant | - | - | - |
| *CFL2* | Cofilin 2 | rs139087926 | 14:35180564 | 0.000399361 | 0.00120581 | 0.0010011 | 0.0008276 | 3_prime_UTR_variant | - | - | - |
| *CGREF1* | Cell Growth Regulator With EF-Hand Domain 1 | rs114353144 | 2:27322424 | 0.00119808 | 0.00119902 | 0.00103513 | 0.00103 | missense_variant | Tolerated | Benign | Damaging |
| *CHAF1B* | Chromatin Assembly Factor 1 Subunit B | [rs62229404](http://www.ncbi.nlm.nih.gov/projects/SNP/snp_ref.cgi?rs=rs62229404) | [21:37759957](genomebrowse://api/zoom?locus=21:37759957) | 0.0401358 | 0.0700902 | 0.0719225 | 0.069 | missense_variant | Tolerated | Benign | Damaging |
| *CHCHD3* | Coiled-Coil-Helix-Coiled-Coil-Helix Domain Containing 3 | rs111673548 | 7:132719655 | 0.00658946 | 0.0022484 | 0.00649309 | 0.002557 | intron_variant | - | - | - |
| *CHD8* | Chromodomain Helicase DNA Binding Protein 8 | rs61744173 | 14:21896192 | 0.00219649 | 0.00893858 | 0.00968242 | 0.008422 | synonymous_variant | - | - | - |
| *CHIA* | Chitinase Acidic | [rs41282492](http://www.ncbi.nlm.nih.gov/projects/SNP/snp_ref.cgi?rs=rs41282492) | [1:111854889](genomebrowse://api/zoom?locus=1:111854889) | 0.108626 | 0.105233 | 0.124806 | 0.107 | missense_variant | Tolerated | Benign | Damaging |
| *CHIA* | Chitinase Acidic | [rs41282494](http://www.ncbi.nlm.nih.gov/projects/SNP/snp_ref.cgi?rs=rs41282494) | [1:111854895](genomebrowse://api/zoom?locus=1:111854895) | 0.107827 | 0.105515 | 0.123657 | 0.107 | missense_variant | Damaging | Possibly damaging | Damaging |
| *CHIA* | Chitinase Acidic | [rs41282496](http://www.ncbi.nlm.nih.gov/projects/SNP/snp_ref.cgi?rs=rs41282496) | [1:111854938](genomebrowse://api/zoom?locus=1:111854938) | 0.107827 | 0.10493 | 0.123658 | 0.107 | missense_variant | Tolerated | Benign | Damaging |
| *CHID1* | Chitinase Domain Containing 1 | rs115994760 | 11:870136 | 0.00259585 | 0.000687238 | 0.00320368 | 0.0008567 | synonymous_variant | - | - | - |
| *CHIT1* | Chitinase 1 | rs140728916 | 1:203185737 | 0.000599042 | 0.000142796 | 0.000968679 | 5.131e-05 | 3_prime_UTR_variant | - | - | - |
| *CHIT1* | Chitinase 1 | rs143518872 | 1:203188942 | 0.000199681 | 0.000611879 | 0.000258598 | 0.0006095 | synonymous_variant | - | - | - |
| *CHMP1A* | Charged Multivesicular Body Protein 1A | rs114931496 | 16:89715801 | 0.00439297 | 0.00107712 | 0.00416667 | 0.001245 | missense_variant | - | - | - |
| *CHMP4C* | Charged Multivesicular Body Protein 4C | rs114287276 | 8:82667691 | 0.00259585 | 0.000801902 | 0.00300407 | 0.0009884 | missense_variant | Damaging | Benign | Tolerated |
| *CHRFAM7A* | CHRNA7 (Exons 5-10) And FAM7A (Exons A-E) Fusion | [rs201471858](http://www.ncbi.nlm.nih.gov/projects/SNP/snp_ref.cgi?rs=rs201471858) | [15:30654961](genomebrowse://api/zoom?locus=15:30654961) | - | 0.0231525 | 0.0280165 | 0.012 | synonymous_variant | - | - | - |
| *CHRM3* | Cholinergic Receptor Muscarinic 3 | rs16839102 | 1:240072043 | 0.00798722 | 0.00126808 | 0.00413463 | 0.00154 | missense_variant | Tolerated | Benign | Damaging |
| *CHRNA4* | Cholinergic Receptor Nicotinic Alpha 4 Subunit | [rs45456294](http://www.ncbi.nlm.nih.gov/projects/SNP/snp_ref.cgi?rs=rs45456294) | [20:61974832](genomebrowse://api/zoom?locus=20:61974832) | 0.0495208 | 0.0512549 | 0.0438171 | 0.064 | 3_prime_UTR_variant | - | - | - |
| *CHRNA4* | Cholinergic Receptor Nicotinic Alpha 4 Subunit | [rs6090378](http://www.ncbi.nlm.nih.gov/projects/SNP/snp_ref.cgi?rs=rs6090378) | [20:61975378](genomebrowse://api/zoom?locus=20:61975378) | 0.0676917 | 0.0634986 | 0.0539789 | 0.078 | 3_prime_UTR_variant | - | - | - |
| *CHRNA4* | Cholinergic Receptor Nicotinic Alpha 4 Subunit | c.*2243dupT | [20:61975847](genomebrowse://api/zoom?locus=20:61975847) | 0.288538 | 0.408708 | 0.297544 | 0.326 | 3_prime_UTR_variant | - | - | - |
| *CHRNA4* | Cholinergic Receptor Nicotinic Alpha 4 Subunit | [rs45607838](http://www.ncbi.nlm.nih.gov/projects/SNP/snp_ref.cgi?rs=rs45607838) | [20:61977318](genomebrowse://api/zoom?locus=20:61977318) | 0.0129792 | 0.0203891 | 0.0177785 | 0.012 | 3_prime_UTR_variant | - | - | - |
| *CHST12* | Carbohydrate Sulfotransferase 12 | [rs3735099](http://www.ncbi.nlm.nih.gov/projects/SNP/snp_ref.cgi?rs=rs3735099) | [7:2472429](genomebrowse://api/zoom?locus=7:2472429) | 0.0754792 | 0.0617381 | 0.0431531 | 0.061 | missense_variant | Damaging | Benign | Tolerated |
| *CHST12* | Carbohydrate Sulfotransferase 12 | [rs3735100](http://www.ncbi.nlm.nih.gov/projects/SNP/snp_ref.cgi?rs=rs3735100) | [7:2472455](genomebrowse://api/zoom?locus=7:2472455) | 0.0758786 | 0.0617391 | 0.0430041 | 0.062 | missense_variant | Tolerated | Benign | Tolerated |
| *CHST6* | Carbohydrate Sulfotransferase 6 | [rs117435647](http://www.ncbi.nlm.nih.gov/projects/SNP/snp_ref.cgi?rs=rs117435647) | [16:75513243](genomebrowse://api/zoom?locus=16:75513243) | 0.0175719 | 0.0352644 | 0.0348453 | 0.035 | missense_variant | Damaging | Benign | Tolerated |
| *CHST9* | Carbohydrate Sulfotransferase 9 | [rs17703902](http://www.ncbi.nlm.nih.gov/projects/SNP/snp_ref.cgi?rs=rs17703902) | [18:24628487](genomebrowse://api/zoom?locus=18:24628487) | 0.0447284 | 0.0413942 | 0.0314541 | 0.04 | intron_variant | - | - | - |
| *CHSY3* | Chondroitin Sulfate Synthase 3 | [rs10068403](http://www.ncbi.nlm.nih.gov/projects/SNP/snp_ref.cgi?rs=rs10068403) | [5:129520679](genomebrowse://api/zoom?locus=5:129520679) | 0.170527 | 0.0710639 | 0.0892049 | 0.07 | missense_variant | Tolerated | Benign | Tolerated |
| *CHTF18* | Chromosome Transmission Fidelity Factor 18 | [rs742319](http://www.ncbi.nlm.nih.gov/projects/SNP/snp_ref.cgi?rs=rs742319) | [16:841966](genomebrowse://api/zoom?locus=16:841966) | 0.141374 | 0.157904 | 0.140365 | 0.148 | intron_variant | - | - | - |
| *CHTF18* | Chromosome Transmission Fidelity Factor 18 | [rs2277897](http://www.ncbi.nlm.nih.gov/projects/SNP/snp_ref.cgi?rs=rs2277897) | [16:842450](genomebrowse://api/zoom?locus=16:842450) | 0.141773 | 0.156809 | 0.140394 | 0.122 | synonymous_variant | - | - | - |
| *CHTF8* | Chromosome Transmission Fidelity Factor 8 | [rs79051270](http://www.ncbi.nlm.nih.gov/projects/SNP/snp_ref.cgi?rs=rs79051270) | [16:69153251](genomebrowse://api/zoom?locus=16:69153251) | 0.0593051 | 0.050206 | 0.0395893 | 0.031 | 3_prime_UTR_variant | Tolerated | Benign | Damaging |
| *CIAO1* | Cytosolic Iron-Sulfur Assembly Component 1 | rs148682902 | 2:96933377 | 0.00459265 | 0.00859786 | 0.00856496 | 0.008797 | synonymous_variant | - | - | - |
| *CIC* | Capicua Transcriptional Repressor | [rs10410185](http://www.ncbi.nlm.nih.gov/projects/SNP/snp_ref.cgi?rs=rs10410185) | [19:42795554](genomebrowse://api/zoom?locus=19:42795554) | 0.086861 | 0.0795181 | 0.060663 | 0.078 | synonymous_variant | - | - | - |
| *CIDEC* | Cell Death Inducing DFFA Like Effector C | [rs73118319](http://www.ncbi.nlm.nih.gov/projects/SNP/snp_ref.cgi?rs=rs73118319) | [3:9912097](genomebrowse://api/zoom?locus=3:9912097) | 0.028754 | 0.0144701 | 0.0141186 | 0.014 | intron_variant | - | - | - |
| *CIITA* | Class II Major Histocompatibility Complex Transactivator | [rs78108426](http://www.ncbi.nlm.nih.gov/projects/SNP/snp_ref.cgi?rs=rs78108426) | [16:11001421](genomebrowse://api/zoom?locus=16:11001421) | 0.0447284 | 0.0219081 | 0.0161624 | 0.022 | missense_variant | Tolerated | Possibly damaging | Tolerated |
| *CIRBP* | Cold Inducible RNA Binding Protein | rs370909081 | 19:1269948 | 0.000399361 | 0.000302741 | 0.000129241 | 0.0002849 | intron_variant | - | - | - |
| *CIRH1A* | Cirrhosis, Autosomal Recessive 1A (Cirhin) | [rs2288036](http://www.ncbi.nlm.nih.gov/projects/SNP/snp_ref.cgi?rs=rs2288036) | [16:69177268](genomebrowse://api/zoom?locus=16:69177268) | 0.0433307 | 0.0490496 | 0.0425229 | 0.049 | synonymous_variant | - | - | - |
| *CISH* | Cytokine Inducible SH2 Containing Protein | [rs2239752](http://www.ncbi.nlm.nih.gov/projects/SNP/snp_ref.cgi?rs=rs2239752) | [3:50645413](genomebrowse://api/zoom?locus=3:50645413) | 0.141973 | 0.0930101 | 0.0683849 | 0.081 | synonymous_variant | - | - | - |
| *CKAP2* | Cytoskeleton Associated Protein 2 | rs149254206 | 13:53035478 | 0.000998403 | 6.90535e-05 | 0.000226084 | 9.06e-05 | missense_variant | Tolerated | Benign | Tolerated |
| *CKAP5* | Cytoskeleton Associated Protein 5 | rs138443179 | 11:46837930 | 0.00319489 | 0.000390035 | 0.00148531 | 0.0004612 | missense_variant | Tolerated | Benign | Damaging |
| *CKLF* | Chemokine Like Factor | [rs59529247](http://www.ncbi.nlm.nih.gov/projects/SNP/snp_ref.cgi?rs=rs59529247) | [16:66596936](genomebrowse://api/zoom?locus=16:66596936) | 0.0992412 | 0.0704218 | 0.0625162 | 0.06 | intron_variant | - | - | - |
| *CLASRP* | CLK4 Associating Serine/Arginine Rich Protein | rs117912158 | 19:45559799 | 0.00878594 | 0.00792585 | 0.00336134 | 0.008162 | splice_region_variant | - | - | - |
| *CLCA2* | Chloride Channel Accessory 2 | [rs1413426](http://www.ncbi.nlm.nih.gov/projects/SNP/snp_ref.cgi?rs=rs1413426) | [1:86909582](genomebrowse://api/zoom?locus=1:86909582) | 0.102436 | 0.0843829 | 0.0854289 | 0.087 | missense_variant | Tolerated | Benign | Tolerated |
| *CLCA4* | Chloride Channel Accessory 4 | [rs2231592](http://www.ncbi.nlm.nih.gov/projects/SNP/snp_ref.cgi?rs=rs2231592) | [1:87031656](genomebrowse://api/zoom?locus=1:87031656) | 0.0696885 | 0.0367452 | 0.0371617 | 0.037 | missense_variant | Tolerated | Benign | Tolerated |
| *CLCC1* | Chloride Channel CLIC Like 1 | [rs41336244](http://www.ncbi.nlm.nih.gov/projects/SNP/snp_ref.cgi?rs=rs41336244) | [1:109486269](genomebrowse://api/zoom?locus=1:109486269) | 0.0409345 | 0.0548608 | 0.0368132 | 0.053 | intron_variant | - | - | - |
| *CLCN1* | Chloride Voltage-Gated Channel 1 | [rs78085922](http://www.ncbi.nlm.nih.gov/projects/SNP/snp_ref.cgi?rs=rs78085922) | [7:143043304](genomebrowse://api/zoom?locus=7:143043304) | 0.013778 | 0.0128567 | 0.0155906 | 0.014 | synonymous_variant | - | - | - |
| *CLCN5* | Chloride Voltage-Gated Channel 5 | [rs34173954](http://www.ncbi.nlm.nih.gov/projects/SNP/snp_ref.cgi?rs=rs34173954) | [X:49854942](genomebrowse://api/zoom?locus=X:49854942) | 0.0630464 | 0.0162869 | 0.0598546 | 0.019 | synonymous_variant | - | - | - |
| *CLCN6* | Chloride Voltage-Gated Channel 6 | rs2272803 | 1:11898789 | 0.0650958 | 0.0520404 | 0.0411955 | 0.052 | intron_variant | Damaging | - | Tolerated |
| *CLCN7* | Chloride Voltage-Gated Channel 7 | [rs115071340](http://www.ncbi.nlm.nih.gov/projects/SNP/snp_ref.cgi?rs=rs115071340) | [16:1510800](genomebrowse://api/zoom?locus=16:1510800) | 0.0159744 | 0.0168644 | 0.0238788 | 0.017 | intron_variant | - | - | - |
| *CLCNKB* | Chloride Voltage-Gated Channel Kb | rs140218299 | 1:16376125 | 0.000998403 | 0.000194709 | 0.000812057 | 0.0002654 | missense_variant | Damaging | Possibly damaging | Tolerated |
| *CLDN1* | Claudin 1 | [rs56271702](http://www.ncbi.nlm.nih.gov/projects/SNP/snp_ref.cgi?rs=rs56271702) | [3:190030683](genomebrowse://api/zoom?locus=3:190030683) | 0.0533147 | 0.0114261 | 0.0440302 | 0.014 | synonymous_variant | - | - | - |
| *CLEC10A* | C-Type Lectin Domain Containing 10A | [rs35318160](http://www.ncbi.nlm.nih.gov/projects/SNP/snp_ref.cgi?rs=rs35318160) | [17:6980105](genomebrowse://api/zoom?locus=17:6980105) | 0.0179712 | 0.0187322 | 0.018545 | 0.019 | missense_variant | Tolerated | Benign | Tolerated |
| *CLEC10A* | C-Type Lectin Domain Containing 10A | [rs16956478](http://www.ncbi.nlm.nih.gov/projects/SNP/snp_ref.cgi?rs=rs16956478) | [17:6980273](genomebrowse://api/zoom?locus=17:6980273) | 0.0449281 | 0.0261856 | 0.0439421 | 0.028 | missense_variant | Tolerated | Possibly damaging | Tolerated |
| *CLEC17A* | C-Type Lectin Domain Containing 17A | rs114453977 | 19:14705316 | 0.00838658 | 0.00126941 | 0.00391586 | 0.00125 | intron_variant | - | - | - |
| *CLEC18A* | C-Type Lectin Domain Family 18 Member A | [rs199537784](http://www.ncbi.nlm.nih.gov/projects/SNP/snp_ref.cgi?rs=rs199537784) | [16:69985274](genomebrowse://api/zoom?locus=16:69985274) | - | 0.0239966 | 0.022269 | 0.011 | missense_variant | Damaging | Possibly damaging | Tolerated |
| *CLEC18B* | C-Type Lectin Domain Family 18 Member B | [rs146555914](http://www.ncbi.nlm.nih.gov/projects/SNP/snp_ref.cgi?rs=rs146555914) | [16:74443542](genomebrowse://api/zoom?locus=16:74443542) | - | 0.138267 | 0.108771 | 0.136 | splice_region_variant | - | - | - |
| *CLEC18C* | C-Type Lectin Domain Family 18 Member C | [rs149087931](http://www.ncbi.nlm.nih.gov/projects/SNP/snp_ref.cgi?rs=rs149087931) | [16:70211218](genomebrowse://api/zoom?locus=16:70211218) | - | 0.121145 | 0.0639481 | 0.105 | synonymous_variant | - | - | Tolerated |
| *CLEC19A* | C-Type Lectin Domain Containing 19A | [rs179196](http://www.ncbi.nlm.nih.gov/projects/SNP/snp_ref.cgi?rs=rs179196) | [16:19297219](genomebrowse://api/zoom?locus=16:19297219) | 0.211262 | 0.180433 | 0.100511 | 0.134 | missense_variant | Tolerated | Benign | Tolerated |
| *CLEC1B* | C-Type Lectin Domain Family 1 Member B | [rs2273987](http://www.ncbi.nlm.nih.gov/projects/SNP/snp_ref.cgi?rs=rs2273987) | [12:10150961](genomebrowse://api/zoom?locus=12:10150961) | 0.0898562 | 0.0804388 | 0.0749806 | 0.084 | missense_variant | Damaging | Possibly damaging | Damaging |
| *CLEC4F* | C-Type Lectin Domain Family 4 Member F | [rs17006361](http://www.ncbi.nlm.nih.gov/projects/SNP/snp_ref.cgi?rs=rs17006361) | [2:71046536](genomebrowse://api/zoom?locus=2:71046536) | 0.0395367 | 0.0102979 | 0.0411871 | 0.013 | synonymous_variant | - | - | - |
| *CLEC7A* | C-Type Lectin Domain Containing 7A | rs144190589 | 12:10277991 | 0.00638978 | 0.00734229 | 0.00662616 | 0.00766 | synonymous_variant | - | - | - |
| *CLGN* | Calmegin | [rs2175563](http://www.ncbi.nlm.nih.gov/projects/SNP/snp_ref.cgi?rs=rs2175563) | [4:141320021](genomebrowse://api/zoom?locus=4:141320021) | 0.0626997 | 0.0567085 | 0.0539536 | 0.057 | missense_variant | Tolerated | Benign | Damaging |
| *CLGN* | Calmegin | [rs114046582](http://www.ncbi.nlm.nih.gov/projects/SNP/snp_ref.cgi?rs=rs114046582) | [4:141320081](genomebrowse://api/zoom?locus=4:141320081) | 0.0123802 | 0.0179989 | 0.0150937 | 0.016 | missense_variant | Damaging | Benign | Damaging |
| *CLGN* | Calmegin | [rs2567241](http://www.ncbi.nlm.nih.gov/projects/SNP/snp_ref.cgi?rs=rs2567241) | [4:141323162](genomebrowse://api/zoom?locus=4:141323162) | 0.0626997 | 0.0524114 | 0.0540295 | 0.057 | missense_variant | Tolerated | Benign | Damaging |
| *CLHC1* | Clathrin Heavy Chain Linker Domain Containing 1 | [rs17852670](http://www.ncbi.nlm.nih.gov/projects/SNP/snp_ref.cgi?rs=rs17852670) | [2:55449464](genomebrowse://api/zoom?locus=2:55449464) | 0.228035 | 0.217654 | 0.178927 | 0.216 | synonymous_variant | - | - | - |
| *CLK2P* | CDC-Like Kinase 2, Pseudogene 1 | rs148318746 | 7:23625310 | 0.00778754 | 0.00118975 | 0.0052618 | 0.001614 | non_coding_exon_variant | - | - | - |
| *CLMP* | CXADR Like Membrane Protein | [rs2276348](http://www.ncbi.nlm.nih.gov/projects/SNP/snp_ref.cgi?rs=rs2276348) | [11:122955402](genomebrowse://api/zoom?locus=11:122955402) | 0.048123 | 0.0347348 | 0.0241586 | 0.034 | missense_variant | Tolerated | Benign | Tolerated |
| *CLN5* | CLN5 Intracellular Trafficking Protein | [rs34481987](http://www.ncbi.nlm.nih.gov/projects/SNP/snp_ref.cgi?rs=rs34481987) | [13:77570078](genomebrowse://api/zoom?locus=13:77570078) | 0.0275559 | 0.0200278 | 0.0139472 | 0.02 | synonymous_variant | - | - | - |
| *CLPX* | Caseinolytic Mitochondrial Matrix Peptidase Chaperone Subunit X | rs149450217 | 15:65445835 | 0.00319489 | 0.00455686 | 0.00235819 | 0.003863 | intron_variant | - | - | - |
| *CLRN1* | Clarin 1 | [rs111033422](http://www.ncbi.nlm.nih.gov/projects/SNP/snp_ref.cgi?rs=rs111033422) | [3:150690490](genomebrowse://api/zoom?locus=3:150690490) | 0.0441294 | 0.0131072 | 0.0343169 | 0.016 | synonymous_variant | - | - | - |
| *CLRN2* | Clarin 2 | [rs13147559](http://www.ncbi.nlm.nih.gov/projects/SNP/snp_ref.cgi?rs=rs13147559) | [4:17524570](genomebrowse://api/zoom?locus=4:17524570) | 0.095647 | 0.129904 | 0.102805 | 0.128 | missense_variant | Tolerated | Possibly damaging | Damaging |
| *CLRN2* | Clarin 2 | [rs2597792](http://www.ncbi.nlm.nih.gov/projects/SNP/snp_ref.cgi?rs=rs2597792) | [4:17528570](genomebrowse://api/zoom?locus=4:17528570) | 0.0613019 | 0.0457636 | 0.0602557 | 0.048 | synonymous_variant | - | - | - |
| *CLU* | Clusterin | [rs10503814](http://www.ncbi.nlm.nih.gov/projects/SNP/snp_ref.cgi?rs=rs10503814) | [8:27454575](genomebrowse://api/zoom?locus=8:27454575) | 0.057508 | 0.0355256 | 0.0602986 | 0.019 | 3_prime_UTR_variant | - | - | - |
| *CLUAP1* | Clusterin Associated Protein 1 | [rs59492947](http://www.ncbi.nlm.nih.gov/projects/SNP/snp_ref.cgi?rs=rs59492947) | [16:3558283](genomebrowse://api/zoom?locus=16:3558283) | 0.0557109 | 0.0303583 | 0.0474331 | 0.033 | splice_region_variant | - | - | - |
| *CLUAP1* | Clusterin Associated Protein 1 | [rs79684678](http://www.ncbi.nlm.nih.gov/projects/SNP/snp_ref.cgi?rs=rs79684678) | [16:3580565](genomebrowse://api/zoom?locus=16:3580565) | 0.0752796 | 0.0557511 | 0.0680233 | 0.058 | splice_region_variant | - | - | - |
| *CLUH* | Clustered Mitochondria Homolog | rs139530964 | 17:2594895 | 0.00658946 | 0.0021647 | 0.00840934 | 0.002575 | splice_region_variant | - | - | - |
| *CLYBL* | Citrate Lyase Beta Like | [rs3831038](http://www.ncbi.nlm.nih.gov/projects/SNP/snp_ref.cgi?rs=rs3831038) | [13:100517196](genomebrowse://api/zoom?locus=13:100517196) | - | - | 0.250523 | 0.237 | intron_variant | - | - | - |
| *CMAHP* | Cytidine Monophospho-N-Acetylneuraminic Acid Hydroxylase, Pseudogene | [rs2273671](http://www.ncbi.nlm.nih.gov/projects/SNP/snp_ref.cgi?rs=rs2273671) | [6:25115296](genomebrowse://api/zoom?locus=6:25115296) | 0.166134 | 0.189248 | 0.141873 | 0.133 | non_coding_exon_variant | - | - | - |
| *CMC1* | C-X9-C Motif Containing 1 | rs139180372 | 3:28304801 | 0.000798722 | 0.00353469 | 0.00360798 | 0.003773 | synonymous_variant | - | - | - |
| *CNBD2* | Cyclic Nucleotide Binding Domain Containing 2 | rs79513483 | 20:34575395 | 0.00199681 | 0.000727116 | 0.00232588 | 0.0009307 | synonymous_variant | - | - | - |
| *CNBD2* | Cyclic Nucleotide Binding Domain Containing 2 | [rs17347958](http://www.ncbi.nlm.nih.gov/projects/SNP/snp_ref.cgi?rs=rs17347958) | [20:34560609](genomebrowse://api/zoom?locus=20:34560609) | 0.0505192 | 0.0551833 | 0.0332429 | 0.059 | missense_variant | Damaging | Probably damaging | Damaging |
| *CNGA3* | Cyclic Nucleotide Gated Channel Subunit Alpha 3 | rs150759499 | 2:99013260 | 0.00139776 | 0.000223521 | 0.00103319 | 0.0002965 | missense_variant | Damaging | Probably damaging | Damaging |
| *CNGA4* | Cyclic Nucleotide Gated Channel Subunit Alpha 4 | [rs61745230](http://www.ncbi.nlm.nih.gov/projects/SNP/snp_ref.cgi?rs=rs61745230) | [11:6262909](genomebrowse://api/zoom?locus=11:6262909) | 0.0285543 | 0.0228118 | 0.0308985 | 0.025 | missense_variant | Damaging | Benign | Damaging |
| *CNGB1* | Cyclic Nucleotide Gated Channel Subunit Beta 1 | rs192843629 | 16:57950041 | 0.000199681 | 0.000686317 | 0.000420521 | 0.0006865 | missense_variant | Damaging | Probably damaging | Damaging |
| *CNGB1* | Cyclic Nucleotide Gated Channel Subunit Beta 1 | [rs35424571](http://www.ncbi.nlm.nih.gov/projects/SNP/snp_ref.cgi?rs=rs35424571) | [16:57953010](genomebrowse://api/zoom?locus=16:57953010) | 0.0483227 | 0.0443613 | 0.0648256 | 0.046 | synonymous_variant | - | - | - |
| *CNGB3* | Cyclic Nucleotide Gated Channel Subunit Beta 3 | rs112671789 | 8:87638186 | 0.000199681 | 0.00011176 | 0.000613576 | 0.0001895 | intron_variant | - | - | - |
| *CNGB3* | Cyclic Nucleotide Gated Channel Subunit Beta 3 | rs79126074 | 8:87738888 | 0.00758786 | 0.00307462 | 0.00594661 | 0.003673 | splice_region_variant | - | - | - |
| *CNGB3* | Cyclic Nucleotide Gated Channel Subunit Beta 3 | [rs3735970](http://www.ncbi.nlm.nih.gov/projects/SNP/snp_ref.cgi?rs=rs3735970) | [8:87588248](genomebrowse://api/zoom?locus=8:87588248) | 0.0822684 | 0.0874398 | 0.0886763 | 0.087 | synonymous_variant | - | - | - |
| *CNGB3* | Cyclic Nucleotide Gated Channel Subunit Beta 3 | [rs7000747](http://www.ncbi.nlm.nih.gov/projects/SNP/snp_ref.cgi?rs=rs7000747) | [8:87616311](genomebrowse://api/zoom?locus=8:87616311) | 0.0607029 | 0.0119397 | 0.0466822 | 0.015 | intron_variant | - | - | - |
| *CNGB3* | Cyclic Nucleotide Gated Channel Subunit Beta 3 | [rs16916632](http://www.ncbi.nlm.nih.gov/projects/SNP/snp_ref.cgi?rs=rs16916632) | [8:87680282](genomebrowse://api/zoom?locus=8:87680282) | 0.0555112 | 0.0144075 | 0.0454457 | 0.017 | missense_variant | Tolerated | Benign | Tolerated |
| *CNKSR1* | Connector Enhancer Of Kinase Suppressor Of Ras 1 | rs148237566 | 1:26510932 | 0.00239617 | 0.00456859 | 0.00292094 | 0.004482 | synonymous_variant | - | - | - |
| *CNOT10* | CCR4-NOT Transcription Complex Subunit 10 | [rs6781308](http://www.ncbi.nlm.nih.gov/projects/SNP/snp_ref.cgi?rs=rs6781308) | [3:32804185](genomebrowse://api/zoom?locus=3:32804185) | 0.0591054 | 0.0527717 | 0.0560328 | 0.054 | intron_variant | - | - | - |
| *CNOT6L* | CCR4-NOT Transcription Complex Subunit 6 Like | rs199946358 | 4:78650048 | 0.000599042 | 0.000211647 | 0.000581358 | 0.00024 | synonymous_variant | - | - | - |
| *CNTN4* | Contactin 4 | rs181977811 | 3:3079034 | 0.000199681 | 0.000150866 | 0.000677988 | 0.0001812 | intron_variant | - | - | - |
| *CNTN4* | Contactin 4 | [rs78860599](http://www.ncbi.nlm.nih.gov/projects/SNP/snp_ref.cgi?rs=rs78860599) | [3:2787408](genomebrowse://api/zoom?locus=3:2787408) | 0.0275559 | 0.0443989 | 0.039381 | 0.044 | intron_variant | - | - | - |
| *CNTN5* | Contactin 5 | [rs12222337](http://www.ncbi.nlm.nih.gov/projects/SNP/snp_ref.cgi?rs=rs12222337) | [11:99690450](genomebrowse://api/zoom?locus=11:99690450) | - | 0.0985171 | 0.103975 | 0.135 | synonymous_variant | - | - | - |
| *CNTN6* | Contactin 6 | [rs146722842](http://www.ncbi.nlm.nih.gov/projects/SNP/snp_ref.cgi?rs=rs146722842) | [3:1269489](genomebrowse://api/zoom?locus=3:1269489) | 0.0199681 | 0.0183397 | 0.0107874 | 0.019 | intron_variant | - | - | - |
| *CNTNAP2* | Contactin Associated Protein 2 | [rs75858942](http://www.ncbi.nlm.nih.gov/projects/SNP/snp_ref.cgi?rs=rs75858942) | [7:147600642](genomebrowse://api/zoom?locus=7:147600642) | 0.0399361 | 0.0102831 | 0.0413896 | 0.013 | intron_variant | - | - | - |
| *CNTNAP2* | Contactin Associated Protein 2 | [rs10240482](http://www.ncbi.nlm.nih.gov/projects/SNP/snp_ref.cgi?rs=rs10240482) | [7:147674948](genomebrowse://api/zoom?locus=7:147674948) | 0.125799 | 0.0769715 | 0.108271 | 0.08 | splice_region_variant | - | - | - |
| *CNTNAP2* | Contactin Associated Protein 2 | [rs10240503](http://www.ncbi.nlm.nih.gov/projects/SNP/snp_ref.cgi?rs=rs10240503) | [7:147674978](genomebrowse://api/zoom?locus=7:147674978) | 0.257788 | 0.141649 | 0.216461 | 0.152 | synonymous_variant | - | - | - |
| *CNTNAP3* | Contactin Associated Protein Family Member 3 | [rs72721583](http://www.ncbi.nlm.nih.gov/projects/SNP/snp_ref.cgi?rs=rs72721583) | [9:39103775](genomebrowse://api/zoom?locus=9:39103775) | 0.0241613 | 0.035015 | 0.0283337 | 0.031 | synonymous_variant | - | - | - |
| *CNTROB* | Centrobin, Centriole Duplication And Spindle Assembly Protein | [rs62623385](http://www.ncbi.nlm.nih.gov/projects/SNP/snp_ref.cgi?rs=rs62623385) | [17:7847837](genomebrowse://api/zoom?locus=17:7847837) | 0.0121805 | 0.0262647 | 0.0290848 | 0.026 | missense_variant | Tolerated | Benign | Tolerated |
| *COBL* | Cordon-Bleu WH2 Repeat Protein | [rs142060269](http://www.ncbi.nlm.nih.gov/projects/SNP/snp_ref.cgi?rs=rs142060269) | [7:51098568](genomebrowse://api/zoom?locus=7:51098568) | - | 0.373945 | 0.353886 | 0.373 | disruptive_inframe_deletion | - | - | - |
| *COCH* | Cochlin | rs28362777 | 14:31355067 | 0.00159744 | 0.000505223 | 0.00142045 | 0.0006507 | synonymous_variant | - | - | - |
| *COG1* | Component Of Oligomeric Golgi Complex 1 | [rs62621249](http://www.ncbi.nlm.nih.gov/projects/SNP/snp_ref.cgi?rs=rs62621249) | [17:71197323](genomebrowse://api/zoom?locus=17:71197323) | 0.0503195 | 0.0682259 | 0.0603203 | 0.067 | missense_variant | Tolerated | Benign | Damaging |
| *COG5* | Component Of Oligomeric Golgi Complex 5 | [rs34087251](http://www.ncbi.nlm.nih.gov/projects/SNP/snp_ref.cgi?rs=rs34087251) | [7:107002504](genomebrowse://api/zoom?locus=7:107002504) | 0.0375399 | 0.043338 | 0.0617898 | 0.043 | missense_variant | Tolerated | Benign | Tolerated |
| *COG7* | Component Of Oligomeric Golgi Complex 7 | rs140637718 | 16:23454008 | 0.00798722 | 0.00212246 | 0.00774893 | 0.002652 | intron_variant | - | - | - |
| *COG7* | Component Of Oligomeric Golgi Complex 7 | [rs8057712](http://www.ncbi.nlm.nih.gov/projects/SNP/snp_ref.cgi?rs=rs8057712) | [16:23403744](genomebrowse://api/zoom?locus=16:23403744) | 0.107827 | 0.054843 | 0.0826064 | 0.056 | synonymous_variant | - | - | - |
| *COL14A1* | Collagen Type XIV Alpha 1 Chain | [rs17833744](http://www.ncbi.nlm.nih.gov/projects/SNP/snp_ref.cgi?rs=rs17833744) | [8:121292947](genomebrowse://api/zoom?locus=8:121292947) | 0.0321486 | 0.0628575 | 0.0727778 | 0.063 | splice_region_variant | - | - | - |
| *COL17A1* | Collagen Type XVII Alpha 1 Chain | rs72821450 | 10:105812932 | 0.00199681 | 0.00529555 | 0.00429836 | 0.005313 | intron_variant | - | - | - |
| *COL18A1* | Collagen Type XVIII Alpha 1 Chain | rs9975166 | 21:46931004 | 0.00179712 | 0.000202749 | 7.55287e-05 | 0.0002067 | intron_variant | - | - | - |
| *COL18A1* | Collagen Type XVIII Alpha 1 Chain | rs113315760 | 21:46932129 | 0.000998403 | 0.00100961 | 0.000970058 | 0.001111 | synonymous_variant | - | - | - |
| *COL18A1* | Collagen Type XVIII Alpha 1 Chain | [rs17338853](http://www.ncbi.nlm.nih.gov/projects/SNP/snp_ref.cgi?rs=rs17338853) | [21:46896381](genomebrowse://api/zoom?locus=21:46896381) | 0.0285543 | 0.0323142 | 0.0274132 | 0.031 | synonymous_variant | - | - | - |
| *COL18A1* | Collagen Type XVIII Alpha 1 Chain | [rs73370824](http://www.ncbi.nlm.nih.gov/projects/SNP/snp_ref.cgi?rs=rs73370824) | [21:46907338](genomebrowse://api/zoom?locus=21:46907338) | 0.135783 | 0.0729753 | 0.103341 | 0.077 | intron_variant | - | - | - |
| *COL18A1* | Collagen Type XVIII Alpha 1 Chain | [rs79980197](http://www.ncbi.nlm.nih.gov/projects/SNP/snp_ref.cgi?rs=rs79980197) | [21:46911188](genomebrowse://api/zoom?locus=21:46911188) | 0.0688898 | 0.0833491 | 0.078507 | 0.083 | missense_variant | Damaging | Probably damaging | Damaging |
| *COL1A1* | Collagen Type I Alpha 1 Chain | rs62637627 | 17:48273519 | 0.00299521 | 0.000540558 | 0.00152242 | 0.0007083 | synonymous_variant | - | - | - |
| *COL1A1* | Collagen Type I Alpha 1 Chain | rs141726413 | 17:48273737 | 0.00239617 | 0.00754999 | 0.00873447 | 0.008261 | intron_variant | - | - | - |
| *COL1A1* | Collagen Type I Alpha 1 Chain | [rs1057297](http://www.ncbi.nlm.nih.gov/projects/SNP/snp_ref.cgi?rs=rs1057297) | [17:48277235](genomebrowse://api/zoom?locus=17:48277235) | 0.0453275 | 0.0204306 | 0.0392322 | 0.024 | synonymous_variant | - | - | - |
| *COL28A1* | Collagen Type XXVIII Alpha 1 Chain | [rs11984435](http://www.ncbi.nlm.nih.gov/projects/SNP/snp_ref.cgi?rs=rs11984435) | [7:7476098](genomebrowse://api/zoom?locus=7:7476098) | 0.0411342 | 0.0122478 | 0.0319183 | 0.014 | splice_region_variant | - | - | - |
| *COL4A5* | Collagen Type IV Alpha 5 Chain | [rs2272946](http://www.ncbi.nlm.nih.gov/projects/SNP/snp_ref.cgi?rs=rs2272946) | [X:107834453](genomebrowse://api/zoom?locus=X:107834453) | 0.149404 | 0.0434731 | 0.101754 | 0.047 | missense_variant | Tolerated | Benign | Damaging |
| *COL4A5* | Collagen Type IV Alpha 5 Chain | [rs7884085](http://www.ncbi.nlm.nih.gov/projects/SNP/snp_ref.cgi?rs=rs7884085) | [X:107845128](genomebrowse://api/zoom?locus=X:107845128) | 0.149934 | 0.0431482 | 0.100341 | 0.041 | synonymous_variant | - | - | - |
| *COL4A5* | Collagen Type IV Alpha 5 Chain | [rs3747408](http://www.ncbi.nlm.nih.gov/projects/SNP/snp_ref.cgi?rs=rs3747408) | [X:107850076](genomebrowse://api/zoom?locus=X:107850076) | 0.148874 | 0.0438653 | 0.103442 | 0.047 | synonymous_variant | - | - | - |
| *COL4A5* | Collagen Type IV Alpha 5 Chain | [rs61746140](http://www.ncbi.nlm.nih.gov/projects/SNP/snp_ref.cgi?rs=rs61746140) | [X:107929337](genomebrowse://api/zoom?locus=X:107929337) | 0.127417 | 0.0355565 | 0.0909005 | 0.039 | missense_variant | - | - | - |
| *COL5A1* | Collagen Type V Alpha 1 Chain | rs147008954 | 9:137593122 | 0.000399361 | 0.000346032 | 0.000516829 | 0.0003708 | missense_variant | Damaging | Possibly damaging | Tolerated |
| *COL5A1* | Collagen Type V Alpha 1 Chain | rs61737941 | 9:137642459 | 0.00738818 | 0.00158533 | 0.0058147 | 0.001977 | synonymous_variant | - | - | - |
| *COL5A3* | Collagen Type V Alpha 3 Chain | rs62638750 | 19:10084460 | 0.00279553 | 0.00858585 | 0.00810441 | 0.007858 | missense_variant | Tolerated | Benign | Tolerated |
| *COL5A3* | Collagen Type V Alpha 3 Chain | [rs62104313](http://www.ncbi.nlm.nih.gov/projects/SNP/snp_ref.cgi?rs=rs62104313) | [19:10078125](genomebrowse://api/zoom?locus=19:10078125) | 0.248003 | 0.151147 | 0.154325 | 0.127 | intron_variant | - | - | - |
| *COL5A3* | Collagen Type V Alpha 3 Chain | [rs11882346](http://www.ncbi.nlm.nih.gov/projects/SNP/snp_ref.cgi?rs=rs11882346) | [19:10090758](genomebrowse://api/zoom?locus=19:10090758) | 0.0666933 | 0.051337 | 0.0717335 | 0.048 | intron_variant | - | - | - |
| *COL5A3* | Collagen Type V Alpha 3 Chain | [rs114143093](http://www.ncbi.nlm.nih.gov/projects/SNP/snp_ref.cgi?rs=rs114143093) | [19:10097443](genomebrowse://api/zoom?locus=19:10097443) | 0.1248 | 0.0680046 | - | 0.111 | intron_variant | - | - | - |
| *COL5A3* | Collagen Type V Alpha 3 Chain | [rs76321360](http://www.ncbi.nlm.nih.gov/projects/SNP/snp_ref.cgi?rs=rs76321360) | [19:10114759](genomebrowse://api/zoom?locus=19:10114759) | 0.0183706 | 0.0398707 | 0.0424553 | 0.04 | synonymous_variant | - | - | - |
| *COL6A1* | Collagen Type VI Alpha 1 Chain | [rs13051496](http://www.ncbi.nlm.nih.gov/projects/SNP/snp_ref.cgi?rs=rs13051496) | [21:47423509](genomebrowse://api/zoom?locus=21:47423509) | 0.108826 | 0.161847 | 0.157566 | 0.161 | missense_variant | Damaging | Benign | Tolerated |
| *COL6A2* | Collagen Type VI Alpha 2 Chain | rs61735835 | 21:47535816 | 0.0081869 | 0.00223085 | 0.00911913 | 0.002525 | missense_variant | Tolerated | Possibly damaging | Tolerated |
| *COL6A3* | Collagen Type VI Alpha 3 Chain | rs115387170 | 2:238287313 | 0.00219649 | 0.00238809 | 0.00180844 | 0.002446 | synonymous_variant | - | - | - |
| *COL6A3* | Collagen Type VI Alpha 3 Chain | [rs36104025](http://www.ncbi.nlm.nih.gov/projects/SNP/snp_ref.cgi?rs=rs36104025) | [2:238247734](genomebrowse://api/zoom?locus=2:238247734) | 0.0325479 | 0.0658182 | 0.0542994 | 0.067 | missense_variant | Damaging | Probably damaging | Damaging |
| *COL6A3* | Collagen Type VI Alpha 3 Chain | [rs59232721](http://www.ncbi.nlm.nih.gov/projects/SNP/snp_ref.cgi?rs=rs59232721) | [2:238250839](genomebrowse://api/zoom?locus=2:238250839) | 0.038738 | 0.0674127 | 0.0615429 | 0.068 | intron_variant | - | - | - |
| *COL6A3* | Collagen Type VI Alpha 3 Chain | [rs34181055](http://www.ncbi.nlm.nih.gov/projects/SNP/snp_ref.cgi?rs=rs34181055) | [2:238253152](genomebrowse://api/zoom?locus=2:238253152) | 0.0195687 | 0.0289343 | 0.026559 | 0.03 | synonymous_variant | - | - | - |
| *COL6A6* | Collagen Type VI Alpha 6 Chain | rs140872639 | 3:130311412 | 0.00219649 | 0.00038781 | 0.00142165 | 0.0004224 | stop_gained | - | - | Damaging |
| *COL6A6* | Collagen Type VI Alpha 6 Chain | [rs61629992](http://www.ncbi.nlm.nih.gov/projects/SNP/snp_ref.cgi?rs=rs61629992) | [3:130360527](genomebrowse://api/zoom?locus=3:130360527) | 0.0710863 | 0.0746084 | 0.0551626 | 0.078 | missense_variant | Damaging | Benign | Tolerated |
| *COL6A6* | Collagen Type VI Alpha 6 Chain | [rs16830494](http://www.ncbi.nlm.nih.gov/projects/SNP/snp_ref.cgi?rs=rs16830494) | [3:130361856](genomebrowse://api/zoom?locus=3:130361856) | 0.121206 | 0.12771 | 0.0951919 | 0.123 | missense_variant | Damaging | Benign | Tolerated |
| *COLGALT2* | Collagen Beta(1-O)Galactosyltransferase 2 | [rs16861794](http://www.ncbi.nlm.nih.gov/projects/SNP/snp_ref.cgi?rs=rs16861794) | [1:183920191](genomebrowse://api/zoom?locus=1:183920191) | 0.0397364 | 0.032426 | 0.0338589 | 0.034 | synonymous_variant | - | - | - |
| *COLQ* | Collagen Like Tail Subunit Of Asymmetric Acetylcholinesterase | rs116373583 | 3:15497493 | 0.0071885 | 0.0013777 | 0.00604122 | 0.001779 | missense_variant | Tolerated | Benign | Damaging |
| *COLQ* | Collagen Like Tail Subunit Of Asymmetric Acetylcholinesterase | [rs73033051](http://www.ncbi.nlm.nih.gov/projects/SNP/snp_ref.cgi?rs=rs73033051) | [3:15563178](genomebrowse://api/zoom?locus=3:15563178) | 0.158147 | 0.106955 | 0.0975223 | 0.105 | 5_prime_UTR_variant | - | - | - |
| *COMP* | Cartilage Oligomeric Matrix Protein | rs74432818 | 19:18896470 | 0.00479233 | 0.00256882 | 0.00220336 | 0.002669 | intron_variant | - | - | - |
| *COMP* | Cartilage Oligomeric Matrix Protein | [rs61752496](http://www.ncbi.nlm.nih.gov/projects/SNP/snp_ref.cgi?rs=rs61752496) | [19:18893732](genomebrowse://api/zoom?locus=19:18893732) | 0.0654952 | 0.0163158 | 0.0573011 | 0.02 | missense_variant | Tolerated | Benign | Tolerated |
| *COPB2* | COPI Coat Complex Subunit Beta 2 | rs373775494 | 3:139102117 | 0.000399361 | 0.000109772 | 0.000193748 | 9.884e-05 | intron_variant | - | - | - |
| *COPS8* | COP9 Signalosome Subunit 8 | [rs73999622](http://www.ncbi.nlm.nih.gov/projects/SNP/snp_ref.cgi?rs=rs73999622) | [2:238005457](genomebrowse://api/zoom?locus=2:238005457) | 0.0277556 | 0.0171864 | 0.028343 | 0.018 | splice_region_variant | - | - | - |
| *COQ5* | Coenzyme Q5, Methyltransferase | [rs503335](http://www.ncbi.nlm.nih.gov/projects/SNP/snp_ref.cgi?rs=rs503335) | [12:120960057](genomebrowse://api/zoom?locus=12:120960057) | 0.0720847 | 0.0775954 | 0.0791702 | 0.08 | synonymous_variant | - | - | - |
| *COQ6* | Coenzyme Q6, Monooxygenase | [rs17552038](http://www.ncbi.nlm.nih.gov/projects/SNP/snp_ref.cgi?rs=rs17552038) | [14:74416945](genomebrowse://api/zoom?locus=14:74416945) | 0.0976438 | 0.156516 | 0.112069 | 0.074 | 5_prime_UTR_variant | - | - | - |
| *COX10* | Cytochrome C Oxidase Assembly Factor Heme A:Farnesyltransferase COX10 | [rs2230351](http://www.ncbi.nlm.nih.gov/projects/SNP/snp_ref.cgi?rs=rs2230351) | [17:13980058](genomebrowse://api/zoom?locus=17:13980058) | 0.0615016 | 0.0795701 | 0.0750872 | 0.076 | missense_variant | Tolerated | Benign | Damaging |
| *COX10* | Cytochrome C Oxidase Assembly Factor Heme A:Farnesyltransferase COX10 | [rs144352489](http://www.ncbi.nlm.nih.gov/projects/SNP/snp_ref.cgi?rs=rs144352489) | [17:14005395](genomebrowse://api/zoom?locus=17:14005395) | 0.0203674 | 0.0157073 | 0.0158705 | 0.014 | intron_variant | - | - | - |
| *CP* | Ceruloplasmin | rs73866999 | 3:148930285 | 0.0071885 | 0.00145493 | 0.00539894 | 0.002001 | missense_variant | Damaging | Probably damaging | Tolerated |
| *CP* | Ceruloplasmin | [rs34394958](http://www.ncbi.nlm.nih.gov/projects/SNP/snp_ref.cgi?rs=rs34394958) | [3:148895654](genomebrowse://api/zoom?locus=3:148895654) | 0.019369 | 0.033841 | 0.0319465 | 0.033 | synonymous_variant | - | - | - |
| *CP* | Ceruloplasmin | [rs61733458](http://www.ncbi.nlm.nih.gov/projects/SNP/snp_ref.cgi?rs=rs61733458) | [3:148916215](genomebrowse://api/zoom?locus=3:148916215) | 0.0211661 | 0.0287447 | 0.0202546 | 0.029 | missense_variant | Damaging | Possibly damaging | Damaging |
| *CP* | Ceruloplasmin | [rs57192685](http://www.ncbi.nlm.nih.gov/projects/SNP/snp_ref.cgi?rs=rs57192685) | [3:148885066](genomebrowse://api/zoom?locus=3:148885066) | 0.127995 | 0.0892813 | 0.124159 | 0.078 | intron_variant | - | - | - |
| *CPA3* | Carboxypeptidase A3 | rs143184657 | 3:148601439 | 0.00379393 | 0.00620572 | 0.00510171 | 0.006309 | missense_variant | Damaging | Possibly damaging | Tolerated |
| *CPA4* | Carboxypeptidase A4 | rs73721864 | 7:129950667 | 0.00519169 | 0.00125892 | 0.00458331 | 0.00154 | synonymous_variant | - | - | - |
| *CPB2* | Carboxypeptidase B2 | [rs2277440](http://www.ncbi.nlm.nih.gov/projects/SNP/snp_ref.cgi?rs=rs2277440) | [13:46638826](genomebrowse://api/zoom?locus=13:46638826) | 0.143371 | 0.107768 | 0.0940348 | 0.105 | synonymous_variant | - | - | - |
| *CPED1* | Cadherin Like And PC-Esterase Domain Containing 1 | rs147902277 | 7:120655691 | 0.00419329 | 0.000727911 | 0.00261611 | 0.001021 | intron_variant | - | - | - |
| *CPN1* | Carboxypeptidase N Subunit 1 | [rs61733667](http://www.ncbi.nlm.nih.gov/projects/SNP/snp_ref.cgi?rs=rs61733667) | [10:101802262](genomebrowse://api/zoom?locus=10:101802262) | 0.0225639 | 0.0355395 | 0.02684 | 0.035 | synonymous_variant | - | - | - |
| *CPN1* | Carboxypeptidase N Subunit 1 | [rs61751507](http://www.ncbi.nlm.nih.gov/projects/SNP/snp_ref.cgi?rs=rs61751507) | [10:101829514](genomebrowse://api/zoom?locus=10:101829514) | 0.0265575 | 0.0423289 | 0.0325285 | 0.042 | missense_variant | Tolerated | Possibly damaging | Damaging |
| *CPNE1* | Copine 1 | rs573636405 | 20:34214217 | 0.000599042 | 0.000300693 | 0.00012955 | 0.0002553 | synonymous_variant | - | - | - |
| *CPNE1* | Copine 1 | [rs11543239](http://www.ncbi.nlm.nih.gov/projects/SNP/snp_ref.cgi?rs=rs11543239) | [20:34214173](genomebrowse://api/zoom?locus=20:34214173) | 0.0896565 | 0.0699765 | 0.0661079 | 0.075 | missense_variant | Damaging | Benign | Tolerated |
| *CPNE1* | Copine 1 | [rs2230219](http://www.ncbi.nlm.nih.gov/projects/SNP/snp_ref.cgi?rs=rs2230219) | [20:34219480](genomebrowse://api/zoom?locus=20:34219480) | 0.0473243 | 0.0561037 | 0.0354995 | 0.059 | synonymous_variant | - | - | - |
| *CPNE2* | Copine 2 | [rs75543218](http://www.ncbi.nlm.nih.gov/projects/SNP/snp_ref.cgi?rs=rs75543218) | [16:57153441](genomebrowse://api/zoom?locus=16:57153441) | 0.105631 | 0.0541317 | 0.0441348 | 0.049 | intron_variant | - | - | - |
| *CPNE3* | Copine 3 | [rs41333046](http://www.ncbi.nlm.nih.gov/projects/SNP/snp_ref.cgi?rs=rs41333046) | [8:87558846](genomebrowse://api/zoom?locus=8:87558846) | 0.114018 | 0.0244442 | 0.094584 | 0.03 | missense_variant | Damaging | Benign | Damaging |
| *CPNE6* | Copine 6 | rs200941113 | 14:24545788 | 0.000199681 | 9.75285e-05 | 6.4771e-05 | 7.413e-05 | intron_variant | - | - | - |
| *CPS1* | Carbamoyl-Phosphate Synthase 1 | [rs2287600](http://www.ncbi.nlm.nih.gov/projects/SNP/snp_ref.cgi?rs=rs2287600) | [2:211473070](genomebrowse://api/zoom?locus=2:211473070) | 0.136581 | 0.126215 | 0.0991829 | 0.124 | intron_variant | - | - | - |
| *CPS1* | Carbamoyl-Phosphate Synthase 1 | [rs41272671](http://www.ncbi.nlm.nih.gov/projects/SNP/snp_ref.cgi?rs=rs41272671) | [2:211525420](genomebrowse://api/zoom?locus=2:211525420) | 0.0191693 | 0.0352597 | 0.0284773 | 0.035 | intron_variant | - | - | - |
| *CPSF2* | Cleavage And Polyadenylation Specific Factor 2 | [rs932551439](http://www.ncbi.nlm.nih.gov/projects/SNP/snp_ref.cgi?rs=rs932551439) | [14:92621643](genomebrowse://api/zoom?locus=14:92621643) | - | - | - | - | stop_gained | - | - | Damaging |
| *CPT1C* | Carnitine Palmitoyltransferase 1C | rs144722270 | 19:50210832 | 0.000599042 | 0.0018664 | 0.00119494 | 0.001812 | synonymous_variant | - | - | - |
| *CPT2* | Carnitine Palmitoyltransferase 2 | rs147276580 | 1:53676718 | 0.000998403 | 0.000227542 | 0.00087153 | 0.0003377 | missense_variant | Tolerated | Benign | Damaging |
| *CPVL* | Carboxypeptidase Vitellogenic Like | [rs323182](http://www.ncbi.nlm.nih.gov/projects/SNP/snp_ref.cgi?rs=rs323182) | [7:29152371](genomebrowse://api/zoom?locus=7:29152371) | 0.0798722 | 0.100468 | 0.110017 | 0.101 | synonymous_variant | - | - | - |
| *CPXCR1* | CPX Chromosome Region Candidate 1 | [rs78677921](http://www.ncbi.nlm.nih.gov/projects/SNP/snp_ref.cgi?rs=rs78677921) | [X:88008713](genomebrowse://api/zoom?locus=X:88008713) | 0.0638411 | 0.019944 | 0.0518925 | 0.023 | synonymous_variant | - | - | - |
| *CR1L* | Complement C3b/C4b Receptor 1 Like | [rs41303261](http://www.ncbi.nlm.nih.gov/projects/SNP/snp_ref.cgi?rs=rs41303261) | [1:207867854](genomebrowse://api/zoom?locus=1:207867854) | 0.0393371 | 0.0492348 | 0.041306 | 0.051 | missense_variant | Damaging | Probably damaging | Tolerated |
| *CR1L* | Complement C3b/C4b Receptor 1 Like | [rs72468038](http://www.ncbi.nlm.nih.gov/projects/SNP/snp_ref.cgi?rs=rs72468038) | [1:207868022](genomebrowse://api/zoom?locus=1:207868022) | 0.0395367 | 0.0492605 | 0.0414514 | 0.051 | missense_variant | Tolerated | Benign | Tolerated |
| *CRBN* | Cereblon | rs141122257 | 3:3196410 | 0.00219649 | 0.000455915 | 0.00135598 | 0.0006178 | intron_variant | - | - | - |
| *CREB5* | CAMP Responsive Element Binding Protein 5 | rs115746904 | 7:28450075 | 0.00878594 | 0.00138705 | 0.00714147 | 0.001115 | intron_variant | - | - | - |
| *CRELD2* | Cysteine Rich With EGF Like Domains 2 | [rs111939483](http://www.ncbi.nlm.nih.gov/projects/SNP/snp_ref.cgi?rs=rs111939483) | [22:50316015](genomebrowse://api/zoom?locus=22:50316015) | 0.0497204 | 0.0219963 | 0.0342417 | 0.024 | synonymous_variant | - | - | - |
| *CRELD2* | Cysteine Rich With EGF Like Domains 2 | [rs11545763](http://www.ncbi.nlm.nih.gov/projects/SNP/snp_ref.cgi?rs=rs11545763) | [22:50319170](genomebrowse://api/zoom?locus=22:50319170) | 0.067492 | 0.0931697 | 0.0833549 | 0.093 | missense_variant | Damaging | Probably damaging | Damaging |
| *CRISP2* | Cysteine Rich Secretory Protein 2 | [rs76570254](http://www.ncbi.nlm.nih.gov/projects/SNP/snp_ref.cgi?rs=rs76570254) | [6:49663519](genomebrowse://api/zoom?locus=6:49663519) | 0.0313498 | 0.04752 | 0.0438013 | 0.047 | intron_variant | - | - | - |
| *CRISP2* | Cysteine Rich Secretory Protein 2 | [rs36069724](http://www.ncbi.nlm.nih.gov/projects/SNP/snp_ref.cgi?rs=rs36069724) | [6:49663567](genomebrowse://api/zoom?locus=6:49663567) | 0.0141773 | 0.0169049 | 0.0134638 | 0.018 | missense_variant | Damaging | Probably damaging | Damaging |
| *CROCC* | Ciliary Rootlet Coiled-Coil, Rootletin | [rs57442576](http://www.ncbi.nlm.nih.gov/projects/SNP/snp_ref.cgi?rs=rs57442576) | [1:17263290](genomebrowse://api/zoom?locus=1:17263290) | 0.0926518 | 0.0837916 | 0.106993 | 0.091 | missense_variant | Tolerated | Benign | Tolerated |
| *CROT* | Carnitine O-Octanoyltransferase | [rs7785206](http://www.ncbi.nlm.nih.gov/projects/SNP/snp_ref.cgi?rs=rs7785206) | [7:87021023](genomebrowse://api/zoom?locus=7:87021023) | 0.0872604 | 0.0800391 | 0.0839418 | 0.083 | missense_variant | Tolerated | Benign | Tolerated |
| *CRTC3* | CREB Regulated Transcription Coactivator 3 | [rs71407320](http://www.ncbi.nlm.nih.gov/projects/SNP/snp_ref.cgi?rs=rs71407320) | [15:91185291](genomebrowse://api/zoom?locus=15:91185291) | 0.0317492 | 0.0604207 | 0.0586334 | 0.06 | synonymous_variant | - | - | - |
| *CRYBA1* | Crystallin Beta A1 | [rs72819448](http://www.ncbi.nlm.nih.gov/projects/SNP/snp_ref.cgi?rs=rs72819448) | [17:27577334](genomebrowse://api/zoom?locus=17:27577334) | 0.0375399 | 0.0260917 | 0.038668 | 0.027 | intron_variant | - | - | - |
| *CRYBB1* | Crystallin Beta B1 | rs147361174 | 22:27003813 | 0.00279553 | 0.000700417 | 0.00216492 | 0.0008484 | intron_variant | - | - | - |
| *CRYBB2* | Crystallin Beta B2 | rs137852878 | 22:25627539 | 0.000399361 | 0.000106621 | 9.69869e-05 | 8.236e-05 | intron_variant | - | - | - |
| *CSAG1* | Chondrosarcoma Associated Gene 1 | [rs1894359](http://www.ncbi.nlm.nih.gov/projects/SNP/snp_ref.cgi?rs=rs1894359) | [X:151908844](genomebrowse://api/zoom?locus=X:151908844) | 0.0998676 | 0.139309 | 0.154414 | 0.14 | missense_variant | - | - | - |
| *CSAG1* | Chondrosarcoma Associated Gene 1 | [rs796367357](http://www.ncbi.nlm.nih.gov/projects/SNP/snp_ref.cgi?rs=rs796367357) | [X:151908845](genomebrowse://api/zoom?locus=X:151908845) | 0.0998676 | 0.139314 | 0.154298 | 0.14 | stop_gained | - | - | - |
| *CSDE1* | Cold Shock Domain Containing E1 | rs189058114 | 1:115260842 | 0.00159744 | 0.00169861 | 0.00131372 | 0.001343 | splice_region_variant | - | - | - |
| *CSE1L* | Chromosome Segregation 1 Like | rs143358483 | 20:47704595 | 0.00279553 | 0.00142175 | 0.000161363 | 0.001211 | synonymous_variant | - | - | - |
| *CSF2* | Colony Stimulating Factor 2 | rs185490125 | 5:131410485 | 0.000599042 | 0.000134131 | 0.000420521 | 0.0001483 | intron_variant | - | - | - |
| *CSF2RB* | Colony Stimulating Factor 2 Receptor Subunit Beta | rs61736839 | 22:37322101 | 0.00838658 | 0.00855312 | 0.00516562 | 0.008599 | synonymous_variant | - | - | - |
| *CSF3R* | Colony Stimulating Factor 3 Receptor | rs4376694 | 1:36936994 | 0.000599042 | 0.000556597 | 0.000452196 | 0.0004612 | intron_variant | - | - | - |
| *CSGALNACT1* | Chondroitin Sulfate N-Acetylgalactosaminyltransferase 1 | rs61910741 | 8:19263328 | 0.00139776 | 0.0038545 | 0.00319664 | 0.004126 | missense_variant | Damaging | Probably damaging | Damaging |
| *CSGALNACT2* | Chondroitin Sulfate N-Acetylgalactosaminyltransferase 2 | [rs534822228](http://www.ncbi.nlm.nih.gov/projects/SNP/snp_ref.cgi?rs=rs534822228) | [10:43671379](genomebrowse://api/zoom?locus=10:43671379) | 0.129393 | 0.0523365 | 0.0109886 | 0.106 | intron_variant | - | - | - |
| *CSMD1* | CUB And Sushi Multiple Domains 1 | [rs11984691](http://www.ncbi.nlm.nih.gov/projects/SNP/snp_ref.cgi?rs=rs11984691) | [8:2800096](genomebrowse://api/zoom?locus=8:2800096) | 0.0605032 | 0.0264059 | 0.0564714 | 0.028 | missense_variant | Tolerated | Benign | Tolerated |
| *CSMD1* | CUB And Sushi Multiple Domains 1 | [rs7015423](http://www.ncbi.nlm.nih.gov/projects/SNP/snp_ref.cgi?rs=rs7015423) | [8:3000033](genomebrowse://api/zoom?locus=8:3000033) | 0.0748802 | 0.0177359 | 0.0653227 | 0.022 | synonymous_variant | - | - | - |
| *CSMD3* | CUB And Sushi Multiple Domains 3 | rs78926727 | 8:113331015 | 0.00599042 | 0.00158887 | 0.00656491 | 0.001976 | intron_variant | - | - | - |
| *CSMD3* | CUB And Sushi Multiple Domains 3 | [rs16883328](http://www.ncbi.nlm.nih.gov/projects/SNP/snp_ref.cgi?rs=rs16883328) | [8:113277868](genomebrowse://api/zoom?locus=8:113277868) | 0.0848642 | 0.0577094 | 0.0622173 | 0.061 | intron_variant | - | - | - |
| *CSMD3* | CUB And Sushi Multiple Domains 3 | [rs12155677](http://www.ncbi.nlm.nih.gov/projects/SNP/snp_ref.cgi?rs=rs12155677) | [8:113484935](genomebrowse://api/zoom?locus=8:113484935) | 0.0347444 | 0.0627862 | 0.0511611 | 0.061 | synonymous_variant | - | - | - |
| *CSNK1E* | Casein Kinase 1 Epsilon | rs188847511 | 22:38694257 | 0.00219649 | 0.000445947 | 0.00281389 | 0.0004805 | intron_variant | - | - | - |
| *CSNK1G2* | Casein Kinase 1 Gamma 2 | [rs148408595](http://www.ncbi.nlm.nih.gov/projects/SNP/snp_ref.cgi?rs=rs148408595) | [19:1979811](genomebrowse://api/zoom?locus=19:1979811) | 0.0349441 | 0.0232784 | 0.0204711 | 0.02 | missense_variant | Tolerated | Benign | Tolerated |
| *CSPG4* | Chondroitin Sulfate Proteoglycan 4 | [rs112660485](http://www.ncbi.nlm.nih.gov/projects/SNP/snp_ref.cgi?rs=rs112660485) | [15:75985469](genomebrowse://api/zoom?locus=15:75985469) | - | 0.0158855 | 0.0352024 | 0.016 | missense_variant | Damaging | Possibly damaging | Damaging |
| *CSRNP1* | Cysteine And Serine Rich Nuclear Protein 1 | rs147162469 | 3:39184884 | 0.00579073 | 0.00657814 | 0.00326333 | 0.006227 | missense_variant | Tolerated | Benign | Tolerated |
| *CST8* | Cystatin 8 | rs114856939 | 20:23472390 | 0.00579073 | 0.00138087 | 0.00416855 | 0.001656 | missense_variant | Tolerated | Benign | Tolerated |
| *CSTL1* | Cystatin Like 1 | rs76182203 | 20:23425489 | 0.00798722 | 0.0019738 | 0.00678295 | 0.002389 | missense_variant | Damaging | Benign | Tolerated |
| *CT47B1* | Cancer/Testis Antigen Family 47 Member B1 | rs200243472 | X:120009263 | 0.000794702 | 0.000174971 | 0.000375869 | 0.0002251 | missense_variant | Tolerated | Benign | Tolerated |
| *CTAGE10P* | CTAGE Family Member 10, Pseudogene | [rs75540113](http://www.ncbi.nlm.nih.gov/projects/SNP/snp_ref.cgi?rs=rs75540113) | [13:50465577](genomebrowse://api/zoom?locus=13:50465577) | 0.0858626 | 0.0574113 | 0.0459299 | 0.059 | non_coding_exon_variant | - | - | - |
| *CTAGE10P* | CTAGE Family Member 10, Pseudogene | [rs35551290](http://www.ncbi.nlm.nih.gov/projects/SNP/snp_ref.cgi?rs=rs35551290) | [13:50466633](genomebrowse://api/zoom?locus=13:50466633) | 0.0363419 | 0.0681692 | 0.0683169 | 0.069 | non_coding_exon_variant | - | - | - |
| *CTAGE9* | CTAGE Family Member 9 | [rs201694043](http://www.ncbi.nlm.nih.gov/projects/SNP/snp_ref.cgi?rs=rs201694043) | [6:132029857](genomebrowse://api/zoom?locus=6:132029857) | 0.133187 | 0.0458417 | 0.0709436 | 0.029 | synonymous_variant | - | - | - |
| *CTAGE9* | CTAGE Family Member 9 | [rs9398993](http://www.ncbi.nlm.nih.gov/projects/SNP/snp_ref.cgi?rs=rs9398993) | [6:132030037](genomebrowse://api/zoom?locus=6:132030037) | 0.0788738 | 0.0353999 | 0.0369698 | 0.036 | synonymous_variant | - | - | - |
| *CTAGE9* | CTAGE Family Member 9 | [rs200752094](http://www.ncbi.nlm.nih.gov/projects/SNP/snp_ref.cgi?rs=rs200752094) | [6:132030137](genomebrowse://api/zoom?locus=6:132030137) | 0.0726837 | 0.0258545 | 0.0504277 | 0.027 | missense_variant | Damaging | Benign | Tolerated |
| *CTBP1-AS2* | CTBP1 Antisense RNA 2 (Head To Head) | rs79317050 | 4:1244605 | 0.00419329 | 0.00457825 | 0.00397698 | 0.004711 | non_coding_exon_variant | - | - | - |
| *CTDSPL* | CTD Small Phosphatase Like | rs184292727 | 3:38025735 | 0.000399361 | 0.00334022 | 0.0027959 | 0.001533 | 3_prime_UTR_variant | - | - | - |
| *CTDSPL* | CTD Small Phosphatase Like | [rs73058970](http://www.ncbi.nlm.nih.gov/projects/SNP/snp_ref.cgi?rs=rs73058970) | [3:37998566](genomebrowse://api/zoom?locus=3:37998566) | 0.0511182 | 0.0493613 | 0.0610806 | 0.052 | intron_variant | - | - | - |
| *CTDSPL* | CTD Small Phosphatase Like | [rs60500371](http://www.ncbi.nlm.nih.gov/projects/SNP/snp_ref.cgi?rs=rs60500371) | [3:38025751](genomebrowse://api/zoom?locus=3:38025751) | 0.17512 | 0.159779 | 0.177091 | 0.168 | 3_prime_UTR_variant | - | - | - |
| *CTGLF12P* | Centaurin, Gamma-Like Family, Member 12 Pseudogene | [rs77534235](http://www.ncbi.nlm.nih.gov/projects/SNP/snp_ref.cgi?rs=rs77534235) | [10:49218451](genomebrowse://api/zoom?locus=10:49218451) | 0.0836661 | 0.0547124 | 0.0558157 | 0.054 | non_coding_exon_variant | - | - | - |
| *CTNNBL1* | Catenin Beta Like 1 | rs199857944 | 20:36500350 | 0.000199681 | 6.10148e-05 | 0.000194037 | 4.942e-05 | missense_variant | Tolerated | Benign | Damaging |
| *CTNS* | Cystinosin, Lysosomal Cystine Transporter | [rs77453839](http://www.ncbi.nlm.nih.gov/projects/SNP/snp_ref.cgi?rs=rs77453839) | [17:3559781](genomebrowse://api/zoom?locus=17:3559781) | 0.0698882 | 0.0180596 | 0.0599892 | 0.023 | synonymous_variant | - | - | - |
| *CTRC* | Chymotrypsin C | [rs497078](http://www.ncbi.nlm.nih.gov/projects/SNP/snp_ref.cgi?rs=rs497078) | [1:15767036](genomebrowse://api/zoom?locus=1:15767036) | 0.0828674 | 0.0912638 | 0.11589 | 0.093 | synonymous_variant | - | - | - |
| *CTSA* | Cathepsin A | rs368697828 | 20:44523526 | 0.000399361 | 0.000211164 | 6.46078e-05 | 0.0002306 | missense_variant | Damaging | Benign | Tolerated |
| *CTSC* | Cathepsin C | [rs17594](http://www.ncbi.nlm.nih.gov/projects/SNP/snp_ref.cgi?rs=rs17594) | [11:88027393](genomebrowse://api/zoom?locus=11:88027393) | 0.0856629 | 0.0629117 | 0.0605277 | 0.063 | synonymous_variant | - | - | - |
| *CTSL3P* | Cathepsin L Family Member 3, Pseudogene | [rs11141967](http://www.ncbi.nlm.nih.gov/projects/SNP/snp_ref.cgi?rs=rs11141967) | [9:90388501](genomebrowse://api/zoom?locus=9:90388501) | 0.0363419 | 0.0321733 | 0.0410662 | 0.033 | non_coding_exon_variant | - | - | - |
| *CTTNBP2* | Cortactin Binding Protein 2 | [rs62617115](http://www.ncbi.nlm.nih.gov/projects/SNP/snp_ref.cgi?rs=rs62617115) | [7:117375374](genomebrowse://api/zoom?locus=7:117375374) | 0.0165735 | 0.0283086 | 0.026705 | 0.028 | missense_variant | Damaging | Possibly damaging | Tolerated |
| *CTTNBP2NL* | CTTNBP2 N-Terminal Like | rs144647165 | 1:112958740 | 0.00978434 | 0.00296056 | 0.00943701 | 0.003426 | intron_variant | - | - | - |
| *CUBN* | Cubilin | rs117128556 | 10:16955978 | 0.00259585 | 0.00557011 | 0.00558822 | 0.00565 | missense_variant | Tolerated | Benign | Damaging |
| *CUBN* | Cubilin | [rs1801238](http://www.ncbi.nlm.nih.gov/projects/SNP/snp_ref.cgi?rs=rs1801238) | [10:16932490](genomebrowse://api/zoom?locus=10:16932490) | 0.0139776 | 0.0282264 | 0.0266098 | 0.029 | missense_variant | Damaging | Probably damaging | Damaging |
| *CUBN* | Cubilin | [rs1873469](http://www.ncbi.nlm.nih.gov/projects/SNP/snp_ref.cgi?rs=rs1873469) | [10:17087156](genomebrowse://api/zoom?locus=10:17087156) | 0.0936502 | 0.0254274 | 0.0849264 | 0.03 | synonymous_variant | - | - | - |
| *CUBN* | Cubilin | [rs12571671](http://www.ncbi.nlm.nih.gov/projects/SNP/snp_ref.cgi?rs=rs12571671) | [10:17146590](genomebrowse://api/zoom?locus=10:17146590) | 0.116813 | 0.0728818 | 0.0727332 | 0.071 | synonymous_variant | - | - | - |
| *CUBN* | Cubilin | [rs41289313](http://www.ncbi.nlm.nih.gov/projects/SNP/snp_ref.cgi?rs=rs41289313) | [10:17157572](genomebrowse://api/zoom?locus=10:17157572) | 0.0846645 | 0.0878173 | 0.065894 | 0.09 | synonymous_variant | - | - | - |
| *CUBN* | Cubilin | [rs41289315](http://www.ncbi.nlm.nih.gov/projects/SNP/snp_ref.cgi?rs=rs41289315) | [10:17165545](genomebrowse://api/zoom?locus=10:17165545) | 0.0884585 | 0.0968323 | 0.0753717 | 0.098 | intron_variant | - | - | - |
| *CUBN* | Cubilin | [rs2273737](http://www.ncbi.nlm.nih.gov/projects/SNP/snp_ref.cgi?rs=rs2273737) | [10:17165552](genomebrowse://api/zoom?locus=10:17165552) | 0.0820687 | 0.0903325 | 0.0722086 | 0.09 | intron_variant | - | - | - |
| *CUL7* | Cullin 7 | rs201972320 | 6:43014211 | 0.000199681 | 0.000207132 | 0.00116407 | 0.0002389 | intron_variant | - | - | - |
| *CUL7* | Cullin 7 | [rs373031091](http://www.ncbi.nlm.nih.gov/projects/SNP/snp_ref.cgi?rs=rs373031091) | [6:43006229](genomebrowse://api/zoom?locus=6:43006229) | - | - | - | - | intron_variant | - | - | - |
| *CUL7* | Cullin 7 | [rs111320577](http://www.ncbi.nlm.nih.gov/projects/SNP/snp_ref.cgi?rs=rs111320577) | [6:43014177](genomebrowse://api/zoom?locus=6:43014177) | 0.0405351 | 0.0263051 | 0.0390413 | 0.027 | intron_variant | - | - | - |
| *CUL9* | Cullin 9 | c.2907T>A | [6:43166450](genomebrowse://api/zoom?locus=6:43166450) | - | - | - | - | synonymous_variant | - | - | - |
| *CWF19L1* | CWF19 Like Cell Cycle Control Factor 1 | [rs7073610](http://www.ncbi.nlm.nih.gov/projects/SNP/snp_ref.cgi?rs=rs7073610) | [10:102006625](genomebrowse://api/zoom?locus=10:102006625) | 0.0800719 | 0.051611 | 0.06273 | 0.053 | missense_variant | Tolerated | Benign | Damaging |
| *CWF19L1* | CWF19 Like Cell Cycle Control Factor 1 | [rs7072367](http://www.ncbi.nlm.nih.gov/projects/SNP/snp_ref.cgi?rs=rs7072367) | [10:102009980](genomebrowse://api/zoom?locus=10:102009980) | 0.0934505 | 0.0546023 | 0.0750468 | 0.056 | intron_variant | - | - | - |
| *CWF19L2* | CWF19 Like Cell Cycle Control Factor 2 | [rs35543671](http://www.ncbi.nlm.nih.gov/projects/SNP/snp_ref.cgi?rs=rs35543671) | [11:107224415](genomebrowse://api/zoom?locus=11:107224415) | 0.0133786 | 0.0226478 | 0.0197067 | 0.02 | synonymous_variant | - | - | - |
| *CXCR6* | C-X-C Motif Chemokine Receptor 6 | rs61751654 | 3:45988525 | 0.00199681 | 0.00415089 | 0.00303539 | 0.004077 | synonymous_variant | - | - | - |
| *CXorf30* | Chromosome X Open Reading Frame 30 | [rs16987461](http://www.ncbi.nlm.nih.gov/projects/SNP/snp_ref.cgi?rs=rs16987461) | [X:36317165](genomebrowse://api/zoom?locus=X:36317165) | 0.0447682 | 0.0100397 | 0.0367849 | 0.016 | missense_variant | Tolerated | Benign | Tolerated |
| *CXorf40B* | Chromosome X Open Reading Frame 40B | rs146445817 | X:149101900 | 0.00211921 | 0.00059949 | 0.00295289 | 0.0007002 | missense_variant | Tolerated | Benign | Tolerated |
| *CXorf58* | Chromosome X Open Reading Frame 58 | [rs113507987](http://www.ncbi.nlm.nih.gov/projects/SNP/snp_ref.cgi?rs=rs113507987) | [X:23928482](genomebrowse://api/zoom?locus=X:23928482) | 0.0890066 | 0.0324133 | 0.068254 | 0.033 | synonymous_variant | - | - | - |
| *CXorf58* | Chromosome X Open Reading Frame 58 | [rs111886191](http://www.ncbi.nlm.nih.gov/projects/SNP/snp_ref.cgi?rs=rs111886191) | [X:23934485](genomebrowse://api/zoom?locus=X:23934485) | 0.107285 | 0.0391708 | 0.0802534 | 0.037 | intron_variant | - | - | - |
| *CXorf64* | Chromosome X Open Reading Frame 64 | [rs41309538](http://www.ncbi.nlm.nih.gov/projects/SNP/snp_ref.cgi?rs=rs41309538) | [X:125954753](genomebrowse://api/zoom?locus=X:125954753) | 0.0516556 | 0.0619114 | 0.0544483 | 0.081 | synonymous_variant | - | - | - |
| *CXorf66* | Chromosome X Open Reading Frame 66 | rs149552852 | X:139038160 | 0.00476821 | 0.000879867 | 0.00280873 | 0.001054 | missense_variant | Damaging | Benign | Tolerated |
| *CYB5R3* | Cytochrome B5 Reductase 3 | [rs1800457](http://www.ncbi.nlm.nih.gov/projects/SNP/snp_ref.cgi?rs=rs1800457) | [22:43024271](genomebrowse://api/zoom?locus=22:43024271) | 0.0926518 | 0.0201818 | 0.0817752 | 0.026 | missense_variant | Tolerated | Benign | Tolerated |
| *CYBB* | Cytochrome B-245 Beta Chain | [rs34834015](http://www.ncbi.nlm.nih.gov/projects/SNP/snp_ref.cgi?rs=rs34834015) | [X:37664440](genomebrowse://api/zoom?locus=X:37664440) | - | 0.0115682 | 0.0408107 | 0.013 | intron_variant | - | - | - |
| *CYFIP2* | Cytoplasmic FMR1 Interacting Protein 2 | [rs11551374](http://www.ncbi.nlm.nih.gov/projects/SNP/snp_ref.cgi?rs=rs11551374) | [5:156753257](genomebrowse://api/zoom?locus=5:156753257) | 0.0644968 | 0.0958166 | 0.0802585 | 0.094 | synonymous_variant | - | - | - |
| *CYLD* | CYLD Lysine 63 Deubiquitinase | [rs17222902](http://www.ncbi.nlm.nih.gov/projects/SNP/snp_ref.cgi?rs=rs17222902) | [16:50813997](genomebrowse://api/zoom?locus=16:50813997) | 0.0788738 | 0.0893739 | 0.0546113 | 0.089 | intron_variant | - | - | - |
| *CYP11A1* | Cytochrome P450 Family 11 Subfamily A Member 1 | rs58174009 | 15:74637475 | 0.00359425 | 0.00203884 | 0.00468558 | 0.002207 | missense_variant | Tolerated | Benign | Damaging |
| *CYP11B1* | Cytochrome P450 Family 11 Subfamily B Member 1 | rs371436758 | 8:143958340 | 0.00359425 | 0.00103641 | 0.00307563 | 0.001285 | intron_variant | - | - | - |
| *CYP11B1* | Cytochrome P450 Family 11 Subfamily B Member 1 | [rs34570566](http://www.ncbi.nlm.nih.gov/projects/SNP/snp_ref.cgi?rs=rs34570566) | [8:143957738](genomebrowse://api/zoom?locus=8:143957738) | 0.113618 | 0.0529925 | 0.0887323 | 0.057 | synonymous_variant | - | - | - |
| *CYP11B2* | Cytochrome P450 Family 11 Subfamily B Member 2 | [rs5313](http://www.ncbi.nlm.nih.gov/projects/SNP/snp_ref.cgi?rs=rs5313) | [8:143994253](genomebrowse://api/zoom?locus=8:143994253) | 0.133986 | 0.0403271 | 0.104153 | 0.048 | synonymous_variant | - | - | - |
| *CYP11B2* | Cytochrome P450 Family 11 Subfamily B Member 2 | [rs4543](http://www.ncbi.nlm.nih.gov/projects/SNP/snp_ref.cgi?rs=rs4543) | [8:143995743](genomebrowse://api/zoom?locus=8:143995743) | 0.103435 | 0.0852111 | 0.115185 | 0.09 | synonymous_variant | - | - | - |
| *CYP19A1* | Cytochrome P450 Family 19 Subfamily A Member 1 | [rs700519](http://www.ncbi.nlm.nih.gov/projects/SNP/snp_ref.cgi?rs=rs700519) | [15:51507968](genomebrowse://api/zoom?locus=15:51507968) | 0.139976 | 0.0760705 | 0.0813555 | 0.08 | missense_variant | Damaging | Benign | Damaging |
| *CYP21A1P* | Cytochrome P450 Family 21 Subfamily A Member 1, Pseudogene | rs186792335 | 6:31974185 | 0.000998403 | 0.000367956 | 0.00147543 | 0.0004761 | missense_variant | - | - | - |
| *CYP24A1* | Cytochrome P450 Family 24 Subfamily A Member 1 | [rs6068816](http://www.ncbi.nlm.nih.gov/projects/SNP/snp_ref.cgi?rs=rs6068816) | [20:52781091](genomebrowse://api/zoom?locus=20:52781091) | 0.165535 | 0.123169 | 0.090324 | 0.123 | synonymous_variant | - | - | - |
| *CYP2B6* | Cytochrome P450 Family 2 Subfamily B Member 6 | rs58871670 | 19:41512872 | 0.00139776 | 0.00322892 | 0.00449257 | 0.003245 | missense_variant | Tolerated | Benign | Damaging |
| *CYP2B6* | Cytochrome P450 Family 2 Subfamily B Member 6 | rs45482602 | 19:41515255 | 0.000798722 | 0.0025263 | 0.00124142 | 0.002482 | missense_variant | Damaging | Probably damaging | Tolerated |
| *CYP2C8* | Cytochrome P450 Family 2 Subfamily C Member 8 | [rs1058930](http://www.ncbi.nlm.nih.gov/projects/SNP/snp_ref.cgi?rs=rs1058930) | [10:96818119](genomebrowse://api/zoom?locus=10:96818119) | 0.0165735 | 0.0376294 | 0.0382227 | 0.039 | missense_variant | Damaging | Probably damaging | Damaging |
| *CYP2D6* | Cytochrome P450 Family 2 Subfamily D Member 6 | rs28371696 | 22:42526717 | 0.00978434 | 0.00217384 | 0.00569782 | 0.002494 | missense_variant | Tolerated | Benign | Damaging |
| *CYP2W1* | Cytochrome P450 Family 2 Subfamily W Member 1 | rs57806492 | 7:1024179 | 0.00419329 | 0.00341137 | 0.00442793 | 0.002343 | synonymous_variant | - | - | - |
| *CYP3A43* | Cytochrome P450 Family 3 Subfamily A Member 43 | rs190638933 | 7:99454406 | 0.00139776 | 0.00226139 | 0.00142045 | 0.002347 | intron_variant | - | - | - |
| *CYP3A7-CYP3AP1* | CYP3A7-CYP3AP1 Readthrough | [rs72494453](http://www.ncbi.nlm.nih.gov/projects/SNP/snp_ref.cgi?rs=rs72494453) | [7:99293358](genomebrowse://api/zoom?locus=7:99293358) | 0.123602 | 0.0752695 | 0.0336778 | 0.074 | intron_variant | - | - | - |
| *CYP4F24P* | Cytochrome P450 Family 4 Subfamily F Member 24, Pseudogene | [rs4807985](http://www.ncbi.nlm.nih.gov/projects/SNP/snp_ref.cgi?rs=rs4807985) | [19:15881909](genomebrowse://api/zoom?locus=19:15881909) | 0.15655 | 0.179172 | 0.177345 | 0.182 | non_coding_exon_variant | - | - | - |
| *CYP4F8* | Cytochrome P450 Family 4 Subfamily F Member 8 | rs146385529 | 19:15728931 | 0.00259585 | 0.0044587 | 0.00678777 | 0.003429 | missense_variant | - | Benign | - |
| *CYP4V2* | Cytochrome P450 Family 4 Subfamily V Member 2 | [rs62350517](http://www.ncbi.nlm.nih.gov/projects/SNP/snp_ref.cgi?rs=rs62350517) | [4:187115777](genomebrowse://api/zoom?locus=4:187115777) | 0.0225639 | 0.0538267 | 0.0443762 | 0.052 | intron_variant | - | - | - |
| *CYP4V2* | Cytochrome P450 Family 4 Subfamily V Member 2 | [rs10029149](http://www.ncbi.nlm.nih.gov/projects/SNP/snp_ref.cgi?rs=rs10029149) | [4:187126449](genomebrowse://api/zoom?locus=4:187126449) | 0.077476 | 0.0175578 | 0.071327 | 0.023 | synonymous_variant | - | - | - |
| *CYP7B1* | Cytochrome P450 Family 7 Subfamily B Member 1 | [rs59035258](http://www.ncbi.nlm.nih.gov/projects/SNP/snp_ref.cgi?rs=rs59035258) | [8:65527669](genomebrowse://api/zoom?locus=8:65527669) | 0.0177716 | 0.0344633 | 0.0251971 | 0.033 | missense_variant | Tolerated | Benign | Tolerated |
| *CYTH1* | Cytohesin 1 | [rs7210648](http://www.ncbi.nlm.nih.gov/projects/SNP/snp_ref.cgi?rs=rs7210648) | [17:76676989](genomebrowse://api/zoom?locus=17:76676989) | 0.0726837 | - | 0.0322351 | - | intron_variant | - | - | - |
| *DAAM2* | Dishevelled Associated Activator Of Morphogenesis 2 | [rs34432477](http://www.ncbi.nlm.nih.gov/projects/SNP/snp_ref.cgi?rs=rs34432477) | [6:39828815](genomebrowse://api/zoom?locus=6:39828815) | 0.0461262 | 0.0406348 | 0.0488846 | 0.039 | intron_variant | Damaging | Benign | Tolerated |
| *DAAM2* | Dishevelled Associated Activator Of Morphogenesis 2 | [rs2504797](http://www.ncbi.nlm.nih.gov/projects/SNP/snp_ref.cgi?rs=rs2504797) | [6:39847279](genomebrowse://api/zoom?locus=6:39847279) | 0.126398 | 0.163731 | 0.102352 | 0.161 | intron_variant | - | - | - |
| *DAB2* | DAB Adaptor Protein 2 | rs145888939 | 5:39383058 | 0.000399361 | 0.000536782 | 0.000548777 | 0.0005107 | missense_variant | Tolerated | Benign | Damaging |
| *DAB2* | DAB Adaptor Protein 2 | [rs61742957](http://www.ncbi.nlm.nih.gov/projects/SNP/snp_ref.cgi?rs=rs61742957) | [5:39376840](genomebrowse://api/zoom?locus=5:39376840) | 0.0171725 | 0.0306669 | 0.0317788 | 0.029 | synonymous_variant | - | - | - |
| *DAB2* | DAB Adaptor Protein 2 | [rs61744160](http://www.ncbi.nlm.nih.gov/projects/SNP/snp_ref.cgi?rs=rs61744160) | [5:39376858](genomebrowse://api/zoom?locus=5:39376858) | 0.0171725 | 0.030627 | 0.0317486 | 0.029 | synonymous_variant | - | - | - |
| *DACT2* | Dishevelled Binding Antagonist Of Beta Catenin 2 | rs139037320 | 6:168710902 | 0.00539137 | 0.00132716 | 0.00484747 | 0.001065 | missense_variant | Tolerated | Benign | Tolerated |
| *DACT2* | Dishevelled Binding Antagonist Of Beta Catenin 2 | [rs41266321](http://www.ncbi.nlm.nih.gov/projects/SNP/snp_ref.cgi?rs=rs41266321) | [6:168709147](genomebrowse://api/zoom?locus=6:168709147) | 0.0441294 | 0.0731032 | 0.0671999 | 0.065 | synonymous_variant | - | - | - |
| *DACT3-AS1* | DACT3 Antisense RNA 1 | rs138656141 | 19:47177915 | 0.00139776 | 0.000579173 | 0.00236414 | 0.0006754 | synonymous_variant | - | - | - |
| *DARS2* | Aspartyl-TRNA Synthetase 2, Mitochondrial | [rs115051769](http://www.ncbi.nlm.nih.gov/projects/SNP/snp_ref.cgi?rs=rs115051769) | [1:173800667](genomebrowse://api/zoom?locus=1:173800667) | 0.0101837 | 0.0180022 | 0.0178583 | 0.018 | splice_region_variant | - | - | - |
| *DBH* | Dopamine Beta-Hydroxylase | rs3025400 | 9:136509351 | 0.00279553 | 0.000556341 | 0.00229432 | 0.0007825 | synonymous_variant | - | - | - |
| *DBH* | Dopamine Beta-Hydroxylase | [rs1611119](http://www.ncbi.nlm.nih.gov/projects/SNP/snp_ref.cgi?rs=rs1611119) | [9:136505127](genomebrowse://api/zoom?locus=9:136505127) | 0.0279553 | 0.0488125 | 0.0451694 | 0.049 | intron_variant | - | - | - |
| *DBH* | Dopamine Beta-Hydroxylase | [rs1611120](http://www.ncbi.nlm.nih.gov/projects/SNP/snp_ref.cgi?rs=rs1611120) | [9:136507301](genomebrowse://api/zoom?locus=9:136507301) | 0.0730831 | 0.0586822 | 0.059611 | 0.059 | intron_variant | - | - | - |
| *DBH* | Dopamine Beta-Hydroxylase | [rs1611126](http://www.ncbi.nlm.nih.gov/projects/SNP/snp_ref.cgi?rs=rs1611126) | [9:136509448](genomebrowse://api/zoom?locus=9:136509448) | 0.124401 | 0.0748593 | 0.130127 | 0.081 | splice_region_variant | - | - | - |
| *DBH* | Dopamine Beta-Hydroxylase | [rs7862391](http://www.ncbi.nlm.nih.gov/projects/SNP/snp_ref.cgi?rs=rs7862391) | [9:136516912](genomebrowse://api/zoom?locus=9:136516912) | 0.103035 | 0.0454621 | 0.0899915 | 0.04 | intron_variant | - | - | - |
| *DBH* | Dopamine Beta-Hydroxylase | [rs6271](http://www.ncbi.nlm.nih.gov/projects/SNP/snp_ref.cgi?rs=rs6271) | [9:136522274](genomebrowse://api/zoom?locus=9:136522274) | 0.0207668 | 0.0462664 | 0.0497672 | 0.045 | missense_variant | Tolerated | Possibly damaging | Tolerated |
| *DBR1* | Debranching RNA Lariats 1 | rs6803524 | 3:137880872 | 0.00798722 | 0.00225377 | 0.00878723 | 0.002767 | synonymous_variant | - | - | - |
| *DBR1* | Debranching RNA Lariats 1 | rs35147674 | 3:137892429 | 0.00419329 | 0.00628624 | 0.00287709 | 0.006622 | synonymous_variant | - | - | - |
| *DCAF12* | DDB1 And CUL4 Associated Factor 12 | [rs11557154](http://www.ncbi.nlm.nih.gov/projects/SNP/snp_ref.cgi?rs=rs11557154) | [9:34107505](genomebrowse://api/zoom?locus=9:34107505) | 0.227037 | 0.185661 | 0.124014 | 0.18 | missense_variant | Damaging | Possibly damaging | Damaging |
| *DCAF17* | DDB1 And CUL4 Associated Factor 17 | [rs61731491](http://www.ncbi.nlm.nih.gov/projects/SNP/snp_ref.cgi?rs=rs61731491) | [2:172325498](genomebrowse://api/zoom?locus=2:172325498) | 0.0397364 | 0.0106655 | 0.038146 | 0.013 | synonymous_variant | - | - | - |
| *DCBLD2* | Discoidin, CUB And LCCL Domain Containing 2 | [rs9838238](http://www.ncbi.nlm.nih.gov/projects/SNP/snp_ref.cgi?rs=rs9838238) | [3:98600385](genomebrowse://api/zoom?locus=3:98600385) | 0.0295527 | 0.0406036 | 0.0394533 | 0.041 | missense_variant | Tolerated | Probably damaging | Damaging |
| *DCC* | DCC Netrin 1 Receptor | [rs35891220](http://www.ncbi.nlm.nih.gov/projects/SNP/snp_ref.cgi?rs=rs35891220) | [18:50976898](genomebrowse://api/zoom?locus=18:50976898) | 0.0405351 | 0.0190511 | 0.0377645 | 0.019 | synonymous_variant | - | - | - |
| *DCDC2C* | Doublecortin Domain Containing 2C | [rs79406267](http://www.ncbi.nlm.nih.gov/projects/SNP/snp_ref.cgi?rs=rs79406267) | [2:3816957](genomebrowse://api/zoom?locus=2:3816957) | 0.0185703 | 0.0368984 | 0.040558 | 0.024 | missense_variant | Tolerated | - | - |
| *DCHS1* | Dachsous Cadherin-Related 1 | [rs35599968](http://www.ncbi.nlm.nih.gov/projects/SNP/snp_ref.cgi?rs=rs35599968) | [11:6644427](genomebrowse://api/zoom?locus=11:6644427) | 0.0267572 | 0.0517571 | 0.0572758 | 0.048 | missense_variant | Tolerated | Benign | Tolerated |
| *DCHS1* | Dachsous Cadherin-Related 1 | [rs11040936](http://www.ncbi.nlm.nih.gov/projects/SNP/snp_ref.cgi?rs=rs11040936) | [11:6648573](genomebrowse://api/zoom?locus=11:6648573) | 0.0421326 | 0.022069 | 0.0132208 | 0.02 | synonymous_variant | - | - | - |
| *DCHS2* | Dachsous Cadherin-Related 2 | rs141221497 | 4:155157977 | 0.000798722 | 0.000468615 | 6.45828e-05 | 0.0004366 | synonymous_variant | - | - | - |
| *DCHS2* | Dachsous Cadherin-Related 2 | [rs61741046](http://www.ncbi.nlm.nih.gov/projects/SNP/snp_ref.cgi?rs=rs61741046) | [4:155156542](genomebrowse://api/zoom?locus=4:155156542) | 0.0459265 | 0.0268673 | 0.0369975 | 0.028 | missense_variant | - | Possibly damaging | Tolerated |
| *DCHS2* | Dachsous Cadherin-Related 2 | [rs6824133](http://www.ncbi.nlm.nih.gov/projects/SNP/snp_ref.cgi?rs=rs6824133) | [4:155157531](genomebrowse://api/zoom?locus=4:155157531) | 0.04373 | 0.0102064 | 0.0373289 | 0.013 | missense_variant | - | Benign | Tolerated |
| *DCK* | Deoxycytidine Kinase | [rs11544786](http://www.ncbi.nlm.nih.gov/projects/SNP/snp_ref.cgi?rs=rs11544786) | [4:71888176](genomebrowse://api/zoom?locus=4:71888176) | 0.0319489 | 0.0495504 | 0.0421138 | 0.051 | synonymous_variant | - | - | - |
| *DCP1B* | Decapping MRNA 1B | [rs715146](http://www.ncbi.nlm.nih.gov/projects/SNP/snp_ref.cgi?rs=rs715146) | [12:2062075](genomebrowse://api/zoom?locus=12:2062075) | 0.0247604 | 0.0364029 | 0.0291171 | 0.036 | missense_variant | Tolerated | Benign | Tolerated |
| *DCPS* | Decapping Enzyme, Scavenger | [rs3740915](http://www.ncbi.nlm.nih.gov/projects/SNP/snp_ref.cgi?rs=rs3740915) | [11:126174038](genomebrowse://api/zoom?locus=11:126174038) | 0.0798722 | 0.0737529 | 0.0496057 | 0.069 | synonymous_variant | - | - | - |
| *DDB1* | Damage Specific DNA Binding Protein 1 | [rs2230356](http://www.ncbi.nlm.nih.gov/projects/SNP/snp_ref.cgi?rs=rs2230356) | [11:61099072](genomebrowse://api/zoom?locus=11:61099072) | 0.0473243 | 0.0715597 | 0.0776837 | 0.071 | synonymous_variant | - | - | - |
| *DDX10* | DEAD-Box Helicase 10 | rs201962995 | 11:108811090 | 0.000199681 | 8.12301e-06 | 3.22893e-05 | 8.236e-06 | synonymous_variant | - | - | - |
| *DDX11* | DEAD/H-Box Helicase 11 | [rs71455622](http://www.ncbi.nlm.nih.gov/projects/SNP/snp_ref.cgi?rs=rs71455622) | [12:31249708](genomebrowse://api/zoom?locus=12:31249708) | 0.0397364 | 0.0718488 | 0.0717334 | 0.071 | intron_variant | - | - | - |
| *DDX11* | DEAD/H-Box Helicase 11 | [rs77340729](http://www.ncbi.nlm.nih.gov/projects/SNP/snp_ref.cgi?rs=rs77340729) | [12:31255405](genomebrowse://api/zoom?locus=12:31255405) | 0.0792732 | 0.0421488 | 0.0462538 | 0.044 | synonymous_variant | - | - | - |
| *DDX20* | DEAD-Box Helicase 20 | [rs197414](http://www.ncbi.nlm.nih.gov/projects/SNP/snp_ref.cgi?rs=rs197414) | [1:112309123](genomebrowse://api/zoom?locus=1:112309123) | 0.165935 | 0.120875 | 0.196387 | 0.127 | missense_variant | Tolerated | Benign | Tolerated |
| *DDX20* | DEAD-Box Helicase 20 | [rs85276](http://www.ncbi.nlm.nih.gov/projects/SNP/snp_ref.cgi?rs=rs85276) | [1:112309331](genomebrowse://api/zoom?locus=1:112309331) | 0.165935 | 0.120904 | 0.196253 | 0.127 | missense_variant | Tolerated | Benign | Tolerated |
| *DDX4* | DEAD-Box Helicase 4 | c.-14-22_-14-21insT | [5:55034757](genomebrowse://api/zoom?locus=5:55034757) | - | 0.169936 | 0.0897507 | 0.103 | intron_variant | - | - | - |
| *DDX49* | DEAD-Box Helicase 49 | rs34332105 | 19:19031444 | 0.000599042 | 0.00205985 | 0.00184109 | 0.001911 | synonymous_variant | - | - | - |
| *DDX58* | DExD/H-Box Helicase 58 | rs116612738 | 9:32480254 | 0.00658946 | 0.00125737 | 0.00487695 | 0.001614 | synonymous_variant | - | - | - |
| *DDX58* | DExD/H-Box Helicase 58 | rs138048526 | 9:32488794 | 0.00658946 | 0.00127751 | 0.00516066 | 0.001647 | synonymous_variant | - | - | - |
| *DDX60* | DExD/H-Box Helicase 60 | rs2684369 | 4:169227695 | 0.00758786 | 0.00153961 | 0.00536314 | 0.001911 | synonymous_variant | - | - | - |
| *DDX60L* | DExD/H-Box 60 Like | [rs62334143](http://www.ncbi.nlm.nih.gov/projects/SNP/snp_ref.cgi?rs=rs62334143) | [4:169341537](genomebrowse://api/zoom?locus=4:169341537) | 0.0301518 | 0.0453097 | 0.0490234 | 0.046 | splice_region_variant | - | - | - |
| *DECR1* | 2,4-Dienoyl-CoA Reductase 1 | [rs16902273](http://www.ncbi.nlm.nih.gov/projects/SNP/snp_ref.cgi?rs=rs16902273) | [8:91018507](genomebrowse://api/zoom?locus=8:91018507) | 0.135783 | 0.0855081 | 0.0913297 | 0.055 | intron_variant | - | - | Tolerated |
| *DEF6* | DEF6 Guanine Nucleotide Exchange Factor | [rs61734578](http://www.ncbi.nlm.nih.gov/projects/SNP/snp_ref.cgi?rs=rs61734578) | [6:35287755](genomebrowse://api/zoom?locus=6:35287755) | 0.0205671 | 0.0186983 | 0.0246818 | 0.014 | synonymous_variant | - | - | - |
| *DEFB105A* | Defensin Beta 105A | [rs199678025](http://www.ncbi.nlm.nih.gov/projects/SNP/snp_ref.cgi?rs=rs199678025) | [8:7347054](genomebrowse://api/zoom?locus=8:7347054) | - | 0.178475 | 0.584906 | 0.045 | missense_variant | Damaging | Probably damaging | Damaging |
| *DEFB126* | Defensin Beta 126 | [rs11467497](http://www.ncbi.nlm.nih.gov/projects/SNP/snp_ref.cgi?rs=rs11467497) | [20:126156](genomebrowse://api/zoom?locus=20:126156) | - | 0.146205 | 0.16617 | 0.152 | frameshift_variant | - | - | - |
| *DEFB128* | Defensin Beta 128 | [rs35089290](http://www.ncbi.nlm.nih.gov/projects/SNP/snp_ref.cgi?rs=rs35089290) | [20:168781](genomebrowse://api/zoom?locus=20:168781) | 0.0283546 | 0.0639666 | 0.0556525 | 0.065 | intron_variant | - | - | - |
| *DENND2C* | DENN Domain Containing 2C | rs142662286 | 1:115151373 | 0.0091853 | 0.00795269 | 0.00700814 | 0.007256 | synonymous_variant | - | - | - |
| *DENND2C* | DENN Domain Containing 2C | [rs61752477](http://www.ncbi.nlm.nih.gov/projects/SNP/snp_ref.cgi?rs=rs61752477) | [1:115168440](genomebrowse://api/zoom?locus=1:115168440) | 0.0109824 | 0.0288296 | 0.0324851 | 0.028 | missense_variant | Tolerated | Benign | Tolerated |
| *DENND3* | DENN Domain Containing 3 | rs77142252 | 8:142161819 | 0.0061901 | 0.00150648 | 0.00585117 | 0.001804 | synonymous_variant | - | - | - |
| *DENND4A* | DENN Domain Containing 4A | [rs8036725](http://www.ncbi.nlm.nih.gov/projects/SNP/snp_ref.cgi?rs=rs8036725) | [15:65962190](genomebrowse://api/zoom?locus=15:65962190) | 0.0587061 | 0.0129598 | 0.0512249 | 0.016 | missense_variant | Tolerated | Benign | Damaging |
| *DENND4B* | DENN Domain Containing 4B | [rs2297895](http://www.ncbi.nlm.nih.gov/projects/SNP/snp_ref.cgi?rs=rs2297895) | [1:153913684](genomebrowse://api/zoom?locus=1:153913684) | 0.177117 | 0.147063 | 0.126222 | 0.143 | intron_variant | - | - | - |
| *DENND5B* | DENN Domain Containing 5B | rs138735047 | 12:31613214 | 0.00239617 | 0.00398368 | 0.00658447 | 0.003788 | missense_variant | Damaging | Benign | Damaging |
| *DEPDC1* | DEP Domain Containing 1 | [rs3790479](http://www.ncbi.nlm.nih.gov/projects/SNP/snp_ref.cgi?rs=rs3790479) | [1:68948280](genomebrowse://api/zoom?locus=1:68948280) | 0.0772764 | 0.0575936 | 0.0748544 | 0.06 | missense_variant | Damaging | Benign | Damaging |
| *DEPDC1* | DEP Domain Containing 1 | [rs78030459](http://www.ncbi.nlm.nih.gov/projects/SNP/snp_ref.cgi?rs=rs78030459) | [1:68960349](genomebrowse://api/zoom?locus=1:68960349) | 0.0760783 | 0.0575147 | 0.0746206 | 0.06 | synonymous_variant | - | - | - |
| *DEPDC7* | DEP Domain Containing 7 | [rs34161108](http://www.ncbi.nlm.nih.gov/projects/SNP/snp_ref.cgi?rs=rs34161108) | [11:33049341](genomebrowse://api/zoom?locus=11:33049341) | 0.0702875 | 0.0823802 | 0.063984 | 0.079 | missense_variant | Tolerated | Benign | Tolerated |
| *DEPDC7* | DEP Domain Containing 7 | [rs36077875](http://www.ncbi.nlm.nih.gov/projects/SNP/snp_ref.cgi?rs=rs36077875) | [11:33053005](genomebrowse://api/zoom?locus=11:33053005) | 0.0111821 | 0.0237008 | 0.0213123 | 0.024 | synonymous_variant | - | - | - |
| *DEPDC7* | DEP Domain Containing 7 | [rs17852859](http://www.ncbi.nlm.nih.gov/projects/SNP/snp_ref.cgi?rs=rs17852859) | [11:33054856](genomebrowse://api/zoom?locus=11:33054856) | 0.0139776 | 0.0285554 | 0.029927 | 0.03 | missense_variant | Tolerated | Benign | Tolerated |
| *DERL1* | Derlin 1 | [rs2272722](http://www.ncbi.nlm.nih.gov/projects/SNP/snp_ref.cgi?rs=rs2272722) | [8:124031541](genomebrowse://api/zoom?locus=8:124031541) | 0.0529153 | 0.0630077 | 0.0645349 | 0.063 | missense_variant | Tolerated | Benign | Damaging |
| *DET1* | DET1 Partner Of COP1 E3 Ubiquitin Ligase | [rs75714837](http://www.ncbi.nlm.nih.gov/projects/SNP/snp_ref.cgi?rs=rs75714837) | [15:89056292](genomebrowse://api/zoom?locus=15:89056292) | 0.0263578 | 0.0436614 | 0.0425058 | 0.045 | synonymous_variant | - | - | - |
| *DFNB59* | Deafness, Autosomal Recessive 59 | [rs17304212](http://www.ncbi.nlm.nih.gov/projects/SNP/snp_ref.cgi?rs=rs17304212) | [2:179325735](genomebrowse://api/zoom?locus=2:179325735) | 0.0393371 | 0.056115 | 0.0574237 | 0.055 | missense_variant | Damaging | Benign | Tolerated |
| *DGAT2L6* | Diacylglycerol O-Acyltransferase 2 Like 6 | rs140170619 | X:69424308 | 0.000529801 | 0.000145525 | 0.000417944 | 0.0001977 | synonymous_variant | - | - | - |
| *DGKB* | Diacylglycerol Kinase Beta | [rs34616903](http://www.ncbi.nlm.nih.gov/projects/SNP/snp_ref.cgi?rs=rs34616903) | [7:14188806](genomebrowse://api/zoom?locus=7:14188806) | 0.0399361 | 0.0221715 | 0.0240223 | 0.022 | missense_variant | Tolerated | Benign | Damaging |
| *DGKD* | Diacylglycerol Kinase Delta | [rs10208953](http://www.ncbi.nlm.nih.gov/projects/SNP/snp_ref.cgi?rs=rs10208953) | [2:234356807](genomebrowse://api/zoom?locus=2:234356807) | 0.0103834 | 0.0234845 | 0.0210434 | 0.025 | synonymous_variant | - | - | - |
| *DGKH* | Diacylglycerol Kinase Eta | rs35357520 | 13:42752288 | 0.00778754 | 0.00213326 | 0.00904627 | 0.002759 | synonymous_variant | - | - | - |
| *DGKI* | Diacylglycerol Kinase Iota | [rs863082](http://www.ncbi.nlm.nih.gov/projects/SNP/snp_ref.cgi?rs=rs863082) | [7:137255996](genomebrowse://api/zoom?locus=7:137255996) | 0.206869 | 0.10671 | 0.154691 | 0.119 | synonymous_variant | - | - | - |
| *DGKI* | Diacylglycerol Kinase Iota | [rs834431](http://www.ncbi.nlm.nih.gov/projects/SNP/snp_ref.cgi?rs=rs834431) | [7:137261940](genomebrowse://api/zoom?locus=7:137261940) | 0.27476 | 0.155043 | 0.196444 | 0.143 | intron_variant | - | - | - |
| *DGUOK* | Deoxyguanosine Kinase | [rs10186730](http://www.ncbi.nlm.nih.gov/projects/SNP/snp_ref.cgi?rs=rs10186730) | [2:74153990](genomebrowse://api/zoom?locus=2:74153990) | 0.0435304 | 0.010763 | 0.045164 | 0.014 | 5_prime_UTR_variant | - | - | - |
| *DHRS3* | Dehydrogenase/Reductase 3 | rs6670337 | 1:12639309 | 0.126398 | 0.0572616 | 0.0874085 | 0.062 | intron_variant | - | - | - |
| *DHRSX* | Dehydrogenase/Reductase X-Linked | [rs3813162](http://www.ncbi.nlm.nih.gov/projects/SNP/snp_ref.cgi?rs=rs3813162) | [X:2407207](genomebrowse://api/zoom?locus=X:2407207) | 0.0415335 | 0.0216968 | 0.0126341 | 0.02 | synonymous_variant | - | - | - |
| *DIDO1* | Death Inducer-Obliterator 1 | [rs2295000](http://www.ncbi.nlm.nih.gov/projects/SNP/snp_ref.cgi?rs=rs2295000) | [20:61536938](genomebrowse://api/zoom?locus=20:61536938) | 0.189297 | 0.201081 | 0.18482 | 0.103 | 3_prime_UTR_variant | Damaging | Benign | Tolerated |
| *DIP2C* | Disco Interacting Protein 2 Homolog C | [rs12358220](http://www.ncbi.nlm.nih.gov/projects/SNP/snp_ref.cgi?rs=rs12358220) | [10:402317](genomebrowse://api/zoom?locus=10:402317) | 0.0265575 | 0.0619671 | 0.0653705 | 0.064 | intron_variant | - | - | - |
| *DIP2C* | Disco Interacting Protein 2 Homolog C | [rs11252681](http://www.ncbi.nlm.nih.gov/projects/SNP/snp_ref.cgi?rs=rs11252681) | [10:530775](genomebrowse://api/zoom?locus=10:530775) | 0.0107827 | 0.0274333 | 0.0324541 | 0.028 | intron_variant | Damaging | - | Damaging |
| *DKK2* | Dickkopf WNT Signaling Pathway Inhibitor 2 | [rs34830201](http://www.ncbi.nlm.nih.gov/projects/SNP/snp_ref.cgi?rs=rs34830201) | [4:107845198](genomebrowse://api/zoom?locus=4:107845198) | 0.0145767 | 0.0270844 | 0.0263158 | 0.027 | synonymous_variant | - | - | - |
| *DLEC1* | DLEC1 Cilia And Flagella Associated Protein | [rs114701641](http://www.ncbi.nlm.nih.gov/projects/SNP/snp_ref.cgi?rs=rs114701641) | [3:38158122](genomebrowse://api/zoom?locus=3:38158122) | 0.0155751 | 0.0148476 | 0.0187318 | 0.015 | synonymous_variant | - | - | - |
| *DMBT1* | Deleted In Malignant Brain Tumors 1 | rs572813390 | 10:124358528 | 0.000199681 | 0.000166688 | 9.69243e-05 | 0.0001985 | synonymous_variant | - | - | - |
| *DMBX1* | Diencephalon/Mesencephalon Homeobox 1 | [rs61751016](http://www.ncbi.nlm.nih.gov/projects/SNP/snp_ref.cgi?rs=rs61751016) | [1:46977812](genomebrowse://api/zoom?locus=1:46977812) | 0.038738 | 0.0287177 | 0.0334242 | 0.03 | synonymous_variant | - | - | - |
| *DMP1* | Dentin Matrix Acidic Phosphoprotein 1 | rs145237146 | 4:88583745 | 0.00119808 | 0.00192901 | 0.00161467 | 0.001812 | missense_variant | Tolerated | Benign | Tolerated |
| *DMTF1* | Cyclin D Binding Myb Like Transcription Factor 1 | [rs11974238](http://www.ncbi.nlm.nih.gov/projects/SNP/snp_ref.cgi?rs=rs11974238) | [7:86813831](genomebrowse://api/zoom?locus=7:86813831) | 0.034345 | 0.0280806 | 0.038469 | 0.03 | synonymous_variant | - | - | - |
| *DNAAF1* | Dynein Axonemal Assembly Factor 1 | [rs76108116](http://www.ncbi.nlm.nih.gov/projects/SNP/snp_ref.cgi?rs=rs76108116) | [16:84209688](genomebrowse://api/zoom?locus=16:84209688) | 0.0491214 | 0.0123901 | 0.0493591 | 0.016 | synonymous_variant | - | - | - |
| *DNAAF1* | Dynein Axonemal Assembly Factor 1 | [rs2288024](http://www.ncbi.nlm.nih.gov/projects/SNP/snp_ref.cgi?rs=rs2288024) | [16:84211465](genomebrowse://api/zoom?locus=16:84211465) | 0.0257588 | 0.0338755 | 0.0314498 | 0.033 | 3_prime_UTR_variant | - | - | - |
| *DNAH10* | Dynein Axonemal Heavy Chain 10 | [rs34934281](http://www.ncbi.nlm.nih.gov/projects/SNP/snp_ref.cgi?rs=rs34934281) | [12:124330311](genomebrowse://api/zoom?locus=12:124330311) | 0.0509185 | 0.0867077 | 0.0751196 | 0.087 | missense_variant | Tolerated | Benign | Damaging |
| *DNAH11* | Dynein Axonemal Heavy Chain 11 | rs371793247 | 7:21658670 | 0.000399361 | 0.000110552 | 0.000290698 | 0.0001407 | intron_variant | - | - | - |
| *DNAH11* | Dynein Axonemal Heavy Chain 11 | rs72657336 | 7:21730517 | 0.00319489 | 0.0042767 | 0.00345429 | 0.004071 | intron_variant | - | - | - |
| *DNAH11* | Dynein Axonemal Heavy Chain 11 | rs28370951 | 7:21765494 | 0.00539137 | 0.00161386 | 0.00626494 | 0.001896 | synonymous_variant | - | - | - |
| *DNAH11* | Dynein Axonemal Heavy Chain 11 | rs28549882 | 7:21784089 | 0.00658946 | 0.00177295 | 0.00681348 | 0.002136 | missense_variant | Tolerated | Benign | Tolerated |
| *DNAH11* | Dynein Axonemal Heavy Chain 11 | rs116368970 | 7:21840745 | 0.00539137 | 0.00123578 | 0.00406635 | 0.001375 | intron_variant | - | - | - |
| *DNAH11* | Dynein Axonemal Heavy Chain 11 | rs114257197 | 7:21894071 | 0.00798722 | 0.00190851 | 0.0069808 | 0.002401 | missense_variant | Damaging | Benign | Damaging |
| *DNAH11* | Dynein Axonemal Heavy Chain 11 | [rs17144747](http://www.ncbi.nlm.nih.gov/projects/SNP/snp_ref.cgi?rs=rs17144747) | [7:21639572](genomebrowse://api/zoom?locus=7:21639572) | 0.0758786 | 0.0470726 | 0.0707486 | 0.05 | synonymous_variant | - | - | - |
| *DNAH11* | Dynein Axonemal Heavy Chain 11 | [rs12666072](http://www.ncbi.nlm.nih.gov/projects/SNP/snp_ref.cgi?rs=rs12666072) | [7:21901593](genomebrowse://api/zoom?locus=7:21901593) | 0.0938498 | 0.0911643 | 0.060655 | 0.084 | synonymous_variant | - | - | - |
| *DNAH14* | Dynein Axonemal Heavy Chain 14 | [rs112172758](http://www.ncbi.nlm.nih.gov/projects/SNP/snp_ref.cgi?rs=rs112172758) | [1:225495168](genomebrowse://api/zoom?locus=1:225495168) | 0.0229633 | 0.0440344 | 0.040357 | 0.044 | missense_variant | Damaging | Benign | Tolerated |
| *DNAH14* | Dynein Axonemal Heavy Chain 14 | [rs41268715](http://www.ncbi.nlm.nih.gov/projects/SNP/snp_ref.cgi?rs=rs41268715) | [1:225521101](genomebrowse://api/zoom?locus=1:225521101) | 0.0265575 | 0.0459911 | 0.0409778 | 0.047 | missense_variant | Tolerated | Possibly damaging | Damaging |
| *DNAH14* | Dynein Axonemal Heavy Chain 14 | [rs12184324](http://www.ncbi.nlm.nih.gov/projects/SNP/snp_ref.cgi?rs=rs12184324) | [1:225528340](genomebrowse://api/zoom?locus=1:225528340) | 0.0872604 | 0.0497045 | 0.0933299 | 0.049 | missense_variant | Damaging | Possibly damaging | Tolerated |
| *DNAH14* | Dynein Axonemal Heavy Chain 14 | [rs73136911](http://www.ncbi.nlm.nih.gov/projects/SNP/snp_ref.cgi?rs=rs73136911) | [1:225534324](genomebrowse://api/zoom?locus=1:225534324) | 0.0623003 | 0.0131243 | 0.052635 | 0.022 | missense_variant | Tolerated | Benign | Tolerated |
| *DNAH17* | Dynein Axonemal Heavy Chain 17 | [rs3209030](http://www.ncbi.nlm.nih.gov/projects/SNP/snp_ref.cgi?rs=rs3209030) | [17:76423151](genomebrowse://api/zoom?locus=17:76423151) | 0.0830671 | 0.082314 | 0.0848697 | 0.075 | synonymous_variant | - | - | - |
| *DNAH2* | Dynein Axonemal Heavy Chain 2 | rs185730860 | 17:7671337 | 0.00159744 | 0.00389838 | 0.000969368 | 0.003188 | synonymous_variant | - | - | - |
| *DNAH2* | Dynein Axonemal Heavy Chain 2 | [rs78354379](http://www.ncbi.nlm.nih.gov/projects/SNP/snp_ref.cgi?rs=rs78354379) | [17:7736480](genomebrowse://api/zoom?locus=17:7736480) | 0.0467252 | 0.0298846 | 0.0185317 | 0.028 | missense_variant | Damaging | Possibly damaging | Damaging |
| *DNAH3* | Dynein Axonemal Heavy Chain 3 | rs144845767 | 16:20975877 | 0.00579073 | 0.00156806 | 0.00435906 | 0.00196 | missense_variant | Damaging | Benign | Tolerated |
| *DNAH3* | Dynein Axonemal Heavy Chain 3 | [rs144617499](http://www.ncbi.nlm.nih.gov/projects/SNP/snp_ref.cgi?rs=rs144617499) | [16:21073933](genomebrowse://api/zoom?locus=16:21073933) | 0.0117812 | 0.0195343 | 0.0159842 | 0.019 | missense_variant | Damaging | Probably damaging | Damaging |
| *DNAH5* | Dynein Axonemal Heavy Chain 5 | rs146696580 | 5:13862646 | 0.00179712 | 0.000666791 | 0.00287635 | 0.0008484 | intron_variant | - | - | - |
| *DNAH5* | Dynein Axonemal Heavy Chain 5 | rs143567667 | 5:13864592 | 0.00179712 | 0.000670361 | 0.00284146 | 0.0008484 | missense_variant | Tolerated | Possibly damaging | Damaging |
| *DNAH5* | Dynein Axonemal Heavy Chain 5 | rs146318478 | 5:13902113 | 0.00219649 | 0.000702614 | 0.00294251 | 0.0006288 | intron_variant | - | - | - |
| *DNAH5* | Dynein Axonemal Heavy Chain 5 | [rs2277046](http://www.ncbi.nlm.nih.gov/projects/SNP/snp_ref.cgi?rs=rs2277046) | [5:13717471](genomebrowse://api/zoom?locus=5:13717471) | 0.158946 | 0.148571 | 0.0995735 | 0.141 | missense_variant | Damaging | Benign | Damaging |
| *DNAH5* | Dynein Axonemal Heavy Chain 5 | [rs17263496](http://www.ncbi.nlm.nih.gov/projects/SNP/snp_ref.cgi?rs=rs17263496) | [5:13737444](genomebrowse://api/zoom?locus=5:13737444) | 0.0656949 | 0.104926 | 0.111531 | 0.101 | missense_variant | Damaging | Probably damaging | Damaging |
| *DNAH5* | Dynein Axonemal Heavy Chain 5 | [rs11958022](http://www.ncbi.nlm.nih.gov/projects/SNP/snp_ref.cgi?rs=rs11958022) | [5:13866054](genomebrowse://api/zoom?locus=5:13866054) | 0.120407 | 0.0466871 | 0.0910354 | 0.052 | intron_variant | - | - | - |
| *DNAH6* | Dynein Axonemal Heavy Chain 6 | [rs12992282](http://www.ncbi.nlm.nih.gov/projects/SNP/snp_ref.cgi?rs=rs12992282) | [2:84880695](genomebrowse://api/zoom?locus=2:84880695) | 0.0401358 | 0.0967206 | 0.106847 | 0.078 | synonymous_variant | - | - | - |
| *DNAH6* | Dynein Axonemal Heavy Chain 6 | [rs1192307](http://www.ncbi.nlm.nih.gov/projects/SNP/snp_ref.cgi?rs=rs1192307) | [2:84913588](genomebrowse://api/zoom?locus=2:84913588) | 0.028754 | 0.0387445 | 0.0317927 | 0.042 | intron_variant | - | - | - |
| *DNAH6* | Dynein Axonemal Heavy Chain 6 | [rs1192395](http://www.ncbi.nlm.nih.gov/projects/SNP/snp_ref.cgi?rs=rs1192395) | [2:85024731](genomebrowse://api/zoom?locus=2:85024731) | 0.104233 | 0.123502 | 0.124814 | 0.107 | synonymous_variant | - | - | - |
| *DNAH7* | Dynein Axonemal Heavy Chain 7 | rs146463525 | 2:196728931 | 0.00359425 | 0.00299275 | 0.00203423 | 0.002953 | missense_variant | Damaging | Probably damaging | Damaging |
| *DNAH8* | Dynein Axonemal Heavy Chain 8 | rs141289823 | 6:38754671 | 0.00299521 | 0.000666285 | 0.00284091 | 0.000972 | synonymous_variant | - | - | - |
| *DNAH8* | Dynein Axonemal Heavy Chain 8 | rs145272110 | 6:38805735 | 0.00139776 | 0.00322959 | 0.00222724 | 0.003039 | synonymous_variant | - | - | - |
| *DNAH9* | Dynein Axonemal Heavy Chain 9 | rs116202848 | 17:11757440 | 0.00678914 | 0.00222553 | 0.00797752 | 0.002685 | missense_variant | Tolerated | Possibly damaging | Damaging |
| *DNAI2* | Dynein Axonemal Intermediate Chain 2 | rs35636875 | 17:72285855 | 0.0061901 | 0.00225864 | 0.00916273 | 0.00294 | missense_variant | Damaging | Possibly damaging | Damaging |
| *DNAI2* | Dynein Axonemal Intermediate Chain 2 | rs144035254 | 17:72306239 | 0.00539137 | 0.0013577 | 0.00585117 | 0.00168 | synonymous_variant | - | - | - |
| *DNAJB12* | DnaJ Heat Shock Protein Family (Hsp40) Member B12 | rs41282266 | 10:74100831 | 0.00279553 | 0.00912326 | 0.00794728 | 0.008492 | synonymous_variant | - | - | - |
| *DNAJB12* | DnaJ Heat Shock Protein Family (Hsp40) Member B12 | rs148088395 | 10:74100836 | 0.00199681 | 0.00276223 | 0.00290773 | 0.002644 | missense_variant | Tolerated | Benign | Damaging |
| *DNAJB3* | DnaJ Heat Shock Protein Family (Hsp40) Member B3 | rs146398257 | 2:234652268 | 0.00539137 | 0.00145435 | 0.00622205 | 0.001705 | missense_variant | - | - | - |
| *DNAJB5* | DnaJ Heat Shock Protein Family (Hsp40) Member B5 | rs543735348 | 9:34990449 | 0.00419329 | 0.00108662 | 0.00530127 | 0.001104 | 5_prime_UTR_variant | - | - | - |
| *DNAJC11* | DnaJ Heat Shock Protein Family (Hsp40) Member C11 | rs12137794 | 1:6705944 | 0.0441294 | 0.0734792 | 0.0685729 | 0.069 | missense_variant | Tolerated | Benign | Damaging |
| *DNAJC13* | DnaJ Heat Shock Protein Family (Hsp40) Member C13 | rs61748101 | 3:132213034 | 0.00299521 | 0.00472685 | 0.00268105 | 0.004406 | missense_variant | Damaging | Possibly damaging | Damaging |
| *DNAJC13* | DnaJ Heat Shock Protein Family (Hsp40) Member C13 | [rs62292959](http://www.ncbi.nlm.nih.gov/projects/SNP/snp_ref.cgi?rs=rs62292959) | [3:132224128](genomebrowse://api/zoom?locus=3:132224128) | 0.0932508 | 0.115999 | 0.0915028 | 0.113 | intron_variant | - | - | - |
| *DNAJC13* | DnaJ Heat Shock Protein Family (Hsp40) Member C13 | [rs80120242](http://www.ncbi.nlm.nih.gov/projects/SNP/snp_ref.cgi?rs=rs80120242) | [3:132235344](genomebrowse://api/zoom?locus=3:132235344) | 0.0249601 | 0.0444574 | 0.0416048 | 0.044 | splice_region_variant | - | - | - |
| *DNAJC19* | DnaJ Heat Shock Protein Family (Hsp40) Member C19 | rs527471494 | 3:180707476 | 0.000399361 | 2.73232e-05 | 3.23394e-05 | 1.869e-05 | 5_prime_UTR_variant | - | - | - |
| *DNAJC21* | DnaJ Heat Shock Protein Family (Hsp40) Member C21 | [rs17244979](http://www.ncbi.nlm.nih.gov/projects/SNP/snp_ref.cgi?rs=rs17244979) | [5:34954079](genomebrowse://api/zoom?locus=5:34954079) | 0.0493211 | 0.0706977 | 0.0572933 | 0.073 | synonymous_variant | - | - | - |
| *DNAJC25* | DnaJ Heat Shock Protein Family (Hsp40) Member C25 | [rs1322259](http://www.ncbi.nlm.nih.gov/projects/SNP/snp_ref.cgi?rs=rs1322259) | [9:114411891](genomebrowse://api/zoom?locus=9:114411891) | 0.0533147 | 0.0344661 | 0.0555591 | 0.036 | synonymous_variant | - | - | - |
| *DNAJC5* | DnaJ Heat Shock Protein Family (Hsp40) Member C5 | [rs6089780](http://www.ncbi.nlm.nih.gov/projects/SNP/snp_ref.cgi?rs=rs6089780) | [20:62550880](genomebrowse://api/zoom?locus=20:62550880) | - | 0.0770508 | 0.109516 | 0.089 | intron_variant | - | - | - |
| *DNHD1* | Dynein Heavy Chain Domain 1 | [rs16915277](http://www.ncbi.nlm.nih.gov/projects/SNP/snp_ref.cgi?rs=rs16915277) | [11:6567857](genomebrowse://api/zoom?locus=11:6567857) | 0.0511182 | 0.0242705 | 0.0525669 | 0.028 | missense_variant | Tolerated | Benign | Tolerated |
| *DNM1P46* | Dynamin 1 Pseudogene 46 | [rs202154374](http://www.ncbi.nlm.nih.gov/projects/SNP/snp_ref.cgi?rs=rs202154374) | [15:100339984](genomebrowse://api/zoom?locus=15:100339984) | - | - | 0.0591612 | 0.021 | non_coding_exon_variant | - | - | - |
| *DNM1P46* | Dynamin 1 Pseudogene 46 | [rs202231117](http://www.ncbi.nlm.nih.gov/projects/SNP/snp_ref.cgi?rs=rs202231117) | [15:100345137](genomebrowse://api/zoom?locus=15:100345137) | 0.145767 | 0.219467 | 0.205246 | 0.193 | splice_region_variant | - | - | - |
| *DNMBP* | Dynamin Binding Protein | rs138530279 | 10:101648658 | 0.00179712 | 0.000767475 | 0.00148502 | 0.0007907 | missense_variant | Tolerated | Benign | Tolerated |
| *DNMBP* | Dynamin Binding Protein | [rs61757225](http://www.ncbi.nlm.nih.gov/projects/SNP/snp_ref.cgi?rs=rs61757225) | [10:101639682](genomebrowse://api/zoom?locus=10:101639682) | 0.0369409 | 0.0355197 | 0.0275733 | 0.036 | synonymous_variant | - | - | - |
| *DNMBP* | Dynamin Binding Protein | rs111969797 | 10:101715453 | 0.00159744 | 0.000703001 | 0.00206572 | 0.0007248 | missense_variant | Damaging | Benign | Tolerated |
| *DNMBP* | Dynamin Binding Protein | [rs35924554](http://www.ncbi.nlm.nih.gov/projects/SNP/snp_ref.cgi?rs=rs35924554) | [10:101716112](genomebrowse://api/zoom?locus=10:101716112) | 0.0495208 | 0.0438777 | 0.0496247 | 0.045 | missense_variant | Tolerated | Benign | Tolerated |
| *DNMT1* | DNA Methyltransferase 1 | [rs773760005](http://www.ncbi.nlm.nih.gov/projects/SNP/snp_ref.cgi?rs=rs773760005) | [19:10251049](genomebrowse://api/zoom?locus=19:10251049) | - | 0.272866 | 0.0254289 | 0.149 | intron_variant | - | - | - |
| *DNMT1* | DNA Methyltransferase 1 | [rs2228613](http://www.ncbi.nlm.nih.gov/projects/SNP/snp_ref.cgi?rs=rs2228613) | [19:10265593](genomebrowse://api/zoom?locus=19:10265593) | 0.0397364 | 0.0709941 | 0.0701924 | 0.073 | synonymous_variant | - | - | - |
| *DNPEP* | Aspartyl Aminopeptidase | [rs11539909](http://www.ncbi.nlm.nih.gov/projects/SNP/snp_ref.cgi?rs=rs11539909) | [2:220251685](genomebrowse://api/zoom?locus=2:220251685) | 0.0121805 | 0.0234633 | 0.020685 | 0.022 | missense_variant | Damaging | Benign | Tolerated |
| *DNTT* | DNA Nucleotidylexotransferase | rs41291616 | 10:98084110 | 0.00359425 | 0.00443118 | 0.00335961 | 0.004225 | missense_variant | Damaging | Probably damaging | Damaging |
| *DNTTIP2* | Deoxynucleotidyltransferase Terminal Interacting Protein 2 | [rs35650636](http://www.ncbi.nlm.nih.gov/projects/SNP/snp_ref.cgi?rs=rs35650636) | [1:94342202](genomebrowse://api/zoom?locus=1:94342202) | 0.0557109 | 0.0112631 | 0.0431959 | 0.013 | missense_variant | Damaging | Benign | Tolerated |
| *DOCK1* | Dedicator Of Cytokinesis 1 | rs61733299 | 10:128923768 | 0.000399361 | 0.000211296 | 0.000710365 | 0.0002972 | missense_variant | Tolerated | Benign | Damaging |
| *DOCK1* | Dedicator Of Cytokinesis 1 | [rs35541322](http://www.ncbi.nlm.nih.gov/projects/SNP/snp_ref.cgi?rs=rs35541322) | [10:128807018](genomebrowse://api/zoom?locus=10:128807018) | 0.0185703 | 0.0408624 | 0.0424763 | 0.038 | synonymous_variant | - | - | - |
| *DOCK2* | Dedicator Of Cytokinesis 2 | [rs3734099](http://www.ncbi.nlm.nih.gov/projects/SNP/snp_ref.cgi?rs=rs3734099) | [5:169122803](genomebrowse://api/zoom?locus=5:169122803) | 0.169329 | 0.110015 | 0.172995 | 0.117 | splice_region_variant | - | - | - |
| *DOCK8* | Dedicator Of Cytokinesis 8 | rs141335439 | 9:368164 | 0.00319489 | 0.000865375 | 0.00365034 | 0.00112 | intron_variant | Damaging | Benign | Tolerated |
| *DOCK8* | Dedicator Of Cytokinesis 8 | rs16937932 | 9:396836 | 0.00379393 | 0.00101547 | 0.00377956 | 0.001252 | missense_variant | Damaging | Probably damaging | Damaging |
| *DOCK8* | Dedicator Of Cytokinesis 8 | [rs34722895](http://www.ncbi.nlm.nih.gov/projects/SNP/snp_ref.cgi?rs=rs34722895) | [9:289482](genomebrowse://api/zoom?locus=9:289482) | - | 0.33395 | 0.379126 | 0.336 | intron_variant | - | - | - |
| *DOCK8* | Dedicator Of Cytokinesis 8 | [rs10972587](http://www.ncbi.nlm.nih.gov/projects/SNP/snp_ref.cgi?rs=rs10972587) | [9:368030](genomebrowse://api/zoom?locus=9:368030) | 0.130192 | 0.0559163 | 0.128343 | 0.063 | synonymous_variant | - | - | - |
| *DOCK8* | Dedicator Of Cytokinesis 8 | [rs17673268](http://www.ncbi.nlm.nih.gov/projects/SNP/snp_ref.cgi?rs=rs17673268) | [9:368128](genomebrowse://api/zoom?locus=9:368128) | 0.029353 | 0.0772103 | 0.0844961 | 0.075 | missense_variant | Damaging | Probably damaging | Damaging |
| *DOK1* | Docking Protein 1 | [rs10208552](http://www.ncbi.nlm.nih.gov/projects/SNP/snp_ref.cgi?rs=rs10208552) | [2:74781955](genomebrowse://api/zoom?locus=2:74781955) | 0.0760783 | 0.0236604 | 0.069272 | 0.028 | intron_variant | - | - | - |
| *DOPEY2* | Dopey Family Member 2 | rs77696046 | 21:37603028 | 0.00239617 | 0.00520753 | 0.00613378 | 0.004851 | missense_variant | Tolerated | Benign | Tolerated |
| *DPF3* | Double PHD Fingers 3 | [rs12433339](http://www.ncbi.nlm.nih.gov/projects/SNP/snp_ref.cgi?rs=rs12433339) | [14:73240509](genomebrowse://api/zoom?locus=14:73240509) | 0.0946486 | 0.137655 | 0.110623 | 0.114 | intron_variant | - | - | - |
| *DPY19L2P2* | DPY19L2 Pseudogene 2 | [rs17136078](http://www.ncbi.nlm.nih.gov/projects/SNP/snp_ref.cgi?rs=rs17136078) | [7:102850726](genomebrowse://api/zoom?locus=7:102850726) | 0.123602 | 0.120978 | 0.123424 | 0.119 | non_coding_exon_variant | - | - | - |
| *DPYD* | Dihydropyrimidine Dehydrogenase | rs72977734 | 1:98186465 | 0.00539137 | 0.00129227 | 0.0060323 | 0.001589 | missense_variant | Damaging | Benign | Tolerated |
| *DPYD* | Dihydropyrimidine Dehydrogenase | [rs56293913](http://www.ncbi.nlm.nih.gov/projects/SNP/snp_ref.cgi?rs=rs56293913) | [1:98039541](genomebrowse://api/zoom?locus=1:98039541) | 0.0636981 | 0.0963088 | 0.106951 | 0.098 | intron_variant | - | - | - |
| *DPYD* | Dihydropyrimidine Dehydrogenase | [rs12137711](http://www.ncbi.nlm.nih.gov/projects/SNP/snp_ref.cgi?rs=rs12137711) | [1:97700589](genomebrowse://api/zoom?locus=1:97700589) | 0.0880591 | 0.119881 | 0.0979005 | 0.118 | intron_variant | - | - | - |
| *DPYD* | Dihydropyrimidine Dehydrogenase | [rs1801160](http://www.ncbi.nlm.nih.gov/projects/SNP/snp_ref.cgi?rs=rs1801160) | [1:97770920](genomebrowse://api/zoom?locus=1:97770920) | 0.0439297 | 0.0468797 | 0.0324158 | 0.046 | missense_variant | Tolerated | Probably damaging | Damaging |
| *DR1* | Down-Regulator Of Transcription 1 | [rs3088371](http://www.ncbi.nlm.nih.gov/projects/SNP/snp_ref.cgi?rs=rs3088371) | [1:93826178](genomebrowse://api/zoom?locus=1:93826178) | 0.0734824 | 0.0881962 | 0.084513 | 0.092 | missense_variant | Tolerated | Benign | Damaging |
| *DRD2* | Dopamine Receptor D2 | rs71653614 | 11:113283437 | 0.00279553 | 0.00391688 | 0.00729833 | 0.003813 | missense_variant | Tolerated | Benign | Damaging |
| *DRD2* | Dopamine Receptor D2 | rs200846690 | 11:113288904 | 0.000199681 | 0.000456454 | 0.000161718 | 0.0004283 | intron_variant | - | - | - |
| *DSC1* | Desmocollin 1 | [rs35338395](http://www.ncbi.nlm.nih.gov/projects/SNP/snp_ref.cgi?rs=rs35338395) | [18:28737407](genomebrowse://api/zoom?locus=18:28737407) | 0.0207668 | 0.0394057 | 0.039113 | 0.039 | missense_variant | Damaging | Benign | Damaging |
| *DSC2* | Desmocollin 2 | [rs1790682](http://www.ncbi.nlm.nih.gov/projects/SNP/snp_ref.cgi?rs=rs1790682) | [18:28648200](genomebrowse://api/zoom?locus=18:28648200) | 0.0199681 | 0.0363127 | 0.0350174 | 0.035 | intron_variant | - | - | - |
| *DSC2* | Desmocollin 2 | [rs61731921](http://www.ncbi.nlm.nih.gov/projects/SNP/snp_ref.cgi?rs=rs61731921) | [18:28648975](genomebrowse://api/zoom?locus=18:28648975) | 0.0275559 | 0.0481471 | 0.0442346 | 0.049 | missense_variant | Tolerated | Benign | Tolerated |
| *DSC3* | Desmocollin 3 | rs142985439 | 18:28588188 | 0.00319489 | 0.00357872 | 0.00290604 | 0.003205 | intron_variant | - | - | - |
| *DSCAML1* | DS Cell Adhesion Molecule Like 1 | [rs11216382](http://www.ncbi.nlm.nih.gov/projects/SNP/snp_ref.cgi?rs=rs11216382) | [11:117303858](genomebrowse://api/zoom?locus=11:117303858) | 0.140974 | 0.144369 | 0.182209 | 0.146 | synonymous_variant | - | - | - |
| *DSG1* | Desmoglein 1 | rs145643174 | 18:28934982 | 0.000599042 | 0.000722913 | 0.000355205 | 0.0005765 | synonymous_variant | - | - | - |
| *DSG2* | Desmoglein 2 | rs121913013 | 18:29099850 | 0.000199681 | 0.00186884 | 0.00155009 | 0.001871 | missense_variant | Damaging | Possibly damaging | Tolerated |
| *DSG4* | Desmoglein 4 | rs35378785 | 18:28979427 | 0.00439297 | 0.00453706 | 0.00245446 | 0.004316 | missense_variant | Tolerated | Probably damaging | Damaging |
| *DSG4* | Desmoglein 4 | [rs1020839](http://www.ncbi.nlm.nih.gov/projects/SNP/snp_ref.cgi?rs=rs1020839) | [18:28989674](genomebrowse://api/zoom?locus=18:28989674) | 0.166334 | 0.151066 | 0.144782 | 0.149 | intron_variant | - | - | - |
| *DSP* | Desmoplakin | rs41302885 | 6:7583703 | 0.00259585 | 0.004102 | 0.00284219 | 0.003904 | missense_variant | Damaging | Probably damaging | Damaging |
| *DSPP* | Dentin Sialophosphoprotein | rs191967636 | 4:88535073 | 0.00119808 | 0.00682672 | 0.00935699 | 0.006072 | missense_variant | - | Benign | Tolerated |
| *DST* | Dystonin | rs79225819 | 6:56483404 | 0.00599042 | 0.00156842 | 0.00636099 | 0.002142 | missense_variant | Tolerated | Possibly damaging | - |
| *DST* | Dystonin | rs57439200 | 6:56501329 | 0.00599042 | 0.00165209 | 0.00695253 | 0.002232 | intron_variant | - | - | - |
| *DST* | Dystonin | rs75671065 | 6:56507562 | 0.00279553 | 0.003531 | 0.00290716 | 0.003509 | missense_variant | Damaging | Possibly damaging | - |
| *DST* | Dystonin | [rs45564536](http://www.ncbi.nlm.nih.gov/projects/SNP/snp_ref.cgi?rs=rs45564536) | [6:56471650](genomebrowse://api/zoom?locus=6:56471650) | 0.0650958 | 0.0499457 | 0.0829992 | 0.053 | intron_variant | - | - | - |
| *DST* | Dystonin | [rs16888053](http://www.ncbi.nlm.nih.gov/projects/SNP/snp_ref.cgi?rs=rs16888053) | [6:56471798](genomebrowse://api/zoom?locus=6:56471798) | 0.0369409 | 0.0101945 | 0.0325989 | 0.012 | intron_variant | Damaging | Possibly damaging | - |
| *DST* | Dystonin | c.4831-1648C>T | [6:56480930](genomebrowse://api/zoom?locus=6:56480930) | - | - | - | - | synonymous_variant | - | - | - |
| *DST* | Dystonin | [rs2230863](http://www.ncbi.nlm.nih.gov/projects/SNP/snp_ref.cgi?rs=rs2230863) | [6:56483852](genomebrowse://api/zoom?locus=6:56483852) | 0.0369409 | 0.0101449 | 0.0314195 | 0.012 | synonymous_variant | - | - | - |
| *DTNA* | Dystrobrevin Alpha | [rs6650658](http://www.ncbi.nlm.nih.gov/projects/SNP/snp_ref.cgi?rs=rs6650658) | [18:32335915](genomebrowse://api/zoom?locus=18:32335915) | 0.0479233 | 0.0600327 | 0.0830802 | 0.061 | intron_variant | - | - | - |
| *DTNA* | Dystrobrevin Alpha | [rs541157](http://www.ncbi.nlm.nih.gov/projects/SNP/snp_ref.cgi?rs=rs541157) | [18:32455379](genomebrowse://api/zoom?locus=18:32455379) | 0.128195 | 0.119247 | 0.169425 | 0.123 | intron_variant | - | - | - |
| *DTNBP1* | Dystrobrevin Binding Protein 1 | [rs76702672](http://www.ncbi.nlm.nih.gov/projects/SNP/snp_ref.cgi?rs=rs76702672) | [6:15524048](genomebrowse://api/zoom?locus=6:15524048) | 0.0241613 | 0.0292011 | 0.0229373 | 0.028 | 3_prime_UTR_variant | - | - | - |
| *DTNBP1* | Dystrobrevin Binding Protein 1 | [rs3829893](http://www.ncbi.nlm.nih.gov/projects/SNP/snp_ref.cgi?rs=rs3829893) | [6:15615637](genomebrowse://api/zoom?locus=6:15615637) | 0.172125 | 0.169312 | 0.142714 | 0.166 | splice_region_variant | - | - | - |
| *DUOX1* | Dual Oxidase 1 | rs150755159 | 15:45436303 | 0.000399361 | 0.000959724 | 0.000807233 | 0.0008236 | missense_variant | Tolerated | Benign | Tolerated |
| *DUOX2* | Dual Oxidase 2 | rs143471358 | 15:45389453 | 0.000998403 | 0.00217496 | 0.0016798 | 0.002281 | missense_variant | Damaging | Possibly damaging | Damaging |
| *DUOX2* | Dual Oxidase 2 | [rs56323146](http://www.ncbi.nlm.nih.gov/projects/SNP/snp_ref.cgi?rs=rs56323146) | [15:45386806](genomebrowse://api/zoom?locus=15:45386806) | 0.0704872 | 0.0652431 | 0.0832794 | 0.066 | synonymous_variant | - | - | - |
| *DUOX2* | Dual Oxidase 2 | [rs61730032](http://www.ncbi.nlm.nih.gov/projects/SNP/snp_ref.cgi?rs=rs61730032) | [15:45388140](genomebrowse://api/zoom?locus=15:45388140) | 0.029353 | 0.0147201 | 0.0273351 | 0.017 | synonymous_variant | - | - | - |
| *DUOX2* | Dual Oxidase 2 | [rs73406334](http://www.ncbi.nlm.nih.gov/projects/SNP/snp_ref.cgi?rs=rs73406334) | [15:45397889](genomebrowse://api/zoom?locus=15:45397889) | 0.0628994 | 0.0674732 | 0.0758859 | 0.067 | synonymous_variant | - | - | - |
| *DUOX2* | Dual Oxidase 2 | [rs113400262](http://www.ncbi.nlm.nih.gov/projects/SNP/snp_ref.cgi?rs=rs113400262) | [15:45398369](genomebrowse://api/zoom?locus=15:45398369) | 0.033147 | 0.0526089 | 0.048599 | 0.05 | missense_variant | Tolerated | Benign | Tolerated |
| *DUX2* | Double Homeobox 2 | [rs797024536](http://www.ncbi.nlm.nih.gov/projects/SNP/snp_ref.cgi?rs=rs797024536) | [4:190989567](genomebrowse://api/zoom?locus=4:190989567) | - | - | 0.0414583 | - | missense_variant | - | - | - |
| *DUX2* | Double Homeobox 2 | [rs797038574](http://www.ncbi.nlm.nih.gov/projects/SNP/snp_ref.cgi?rs=rs797038574) | [4:190989574](genomebrowse://api/zoom?locus=4:190989574) | - | - | 0.0397797 | - | missense_variant | - | - | - |
| *DUX4* | Double Homeobox 4 | [rs775307903](http://www.ncbi.nlm.nih.gov/projects/SNP/snp_ref.cgi?rs=rs775307903) | [10:135490839](genomebrowse://api/zoom?locus=10:135490839) | - | 0.234091 | 0.0238095 | 0.118 | intron_variant | - | - | - |
| *DUX4* | Double Homeobox 4 | [rs370060335](http://www.ncbi.nlm.nih.gov/projects/SNP/snp_ref.cgi?rs=rs370060335) | [10:135491028](genomebrowse://api/zoom?locus=10:135491028) | 0.220447 | 0.0347231 | 0.0951026 | 0.052 | intron_variant | - | - | - |
| *DUX4* | Double Homeobox 4 | [rs374542577](http://www.ncbi.nlm.nih.gov/projects/SNP/snp_ref.cgi?rs=rs374542577) | [10:135491154](genomebrowse://api/zoom?locus=10:135491154) | - | 0.133308 | 0.107387 | 0.03 | intron_variant | - | - | - |
| *DYNC1H1* | Dynein Cytoplasmic 1 Heavy Chain 1 | rs75094258 | 14:102469273 | 0.00539137 | 0.000971956 | 0.0033895 | 0.001277 | synonymous_variant | - | - | - |
| *DYNC1H1* | Dynein Cytoplasmic 1 Heavy Chain 1 | [rs3830915](http://www.ncbi.nlm.nih.gov/projects/SNP/snp_ref.cgi?rs=rs3830915) | [14:102483882](genomebrowse://api/zoom?locus=14:102483882) | 0.196685 | 0.138383 | 0.142552 | 0.14 | intron_variant | - | - | - |
| *DYNC1H1* | Dynein Cytoplasmic 1 Heavy Chain 1 | [rs17541366](http://www.ncbi.nlm.nih.gov/projects/SNP/snp_ref.cgi?rs=rs17541366) | [14:102496651](genomebrowse://api/zoom?locus=14:102496651) | 0.0153754 | 0.030861 | 0.0308746 | 0.033 | intron_variant | - | - | - |
| *DYNC1H1* | Dynein Cytoplasmic 1 Heavy Chain 1 | [rs10147313](http://www.ncbi.nlm.nih.gov/projects/SNP/snp_ref.cgi?rs=rs10147313) | [14:102506783](genomebrowse://api/zoom?locus=14:102506783) | 0.0429313 | 0.0183592 | 0.0472606 | 0.02 | intron_variant | - | - | - |
| *DYRK4* | Dual Specificity Tyrosine Phosphorylation Regulated Kinase 4 | [rs2255588](http://www.ncbi.nlm.nih.gov/projects/SNP/snp_ref.cgi?rs=rs2255588) | [12:4716513](genomebrowse://api/zoom?locus=12:4716513) | 0.15615 | 0.0756204 | 0.0978408 | 0.081 | synonymous_variant | - | - | - |
| *DYSF* | Dysferlin | [rs74384941](http://www.ncbi.nlm.nih.gov/projects/SNP/snp_ref.cgi?rs=rs74384941) | [2:71743411](genomebrowse://api/zoom?locus=2:71743411) | 0.0203674 | 0.0359396 | 0.0357535 | 0.034 | intron_variant | - | - | - |
| *DYTN* | Dystrotelin | rs368214018 | 2:207575198 | 0.000599042 | 8.87154e-05 | 0.000419924 | 0.0001076 | intron_variant | - | - | - |
| *DYTN* | Dystrotelin | [rs116768218](http://www.ncbi.nlm.nih.gov/projects/SNP/snp_ref.cgi?rs=rs116768218) | [2:207569623](genomebrowse://api/zoom?locus=2:207569623) | 0.0131789 | 0.0333803 | 0.038628 | 0.032 | missense_variant | Tolerated | Benign | Tolerated |
| *E2F7* | E2F Transcription Factor 7 | [rs310830](http://www.ncbi.nlm.nih.gov/projects/SNP/snp_ref.cgi?rs=rs310830) | [12:77419593](genomebrowse://api/zoom?locus=12:77419593) | 0.0423323 | 0.08308 | 0.0775946 | 0.081 | synonymous_variant | - | - | - |
| *ECE1* | Endothelin Converting Enzyme 1 | [rs1076669](http://www.ncbi.nlm.nih.gov/projects/SNP/snp_ref.cgi?rs=rs1076669) | [1:21573855](genomebrowse://api/zoom?locus=1:21573855) | 0.0285543 | 0.054701 | 0.0508945 | 0.055 | missense_variant | Tolerated | Benign | Tolerated |
| *ECE2* | Endothelin Converting Enzyme 2 | rs56132089 | 3:183995069 | 0.000599042 | 0.000605056 | 0.000678251 | 0.0005848 | missense_variant | Tolerated | Benign | Damaging |
| *ECM1* | Extracellular Matrix Protein 1 | [rs75772057](http://www.ncbi.nlm.nih.gov/projects/SNP/snp_ref.cgi?rs=rs75772057) | [1:150481982](genomebrowse://api/zoom?locus=1:150481982) | 0.0141773 | 0.0200765 | 0.0106376 | 0.019 | intron_variant | - | - | - |
| *ECM1* | Extracellular Matrix Protein 1 | [rs41264469](http://www.ncbi.nlm.nih.gov/projects/SNP/snp_ref.cgi?rs=rs41264469) | [1:150482255](genomebrowse://api/zoom?locus=1:150482255) | 0.0485224 | 0.0973082 | 0.0999153 | 0.096 | intron_variant | - | - | - |
| *EDEM1* | ER Degradation Enhancing Alpha-Mannosidase Like Protein 1 | [rs144177905](http://www.ncbi.nlm.nih.gov/projects/SNP/snp_ref.cgi?rs=rs144177905) | [3:5229643](genomebrowse://api/zoom?locus=3:5229643) | 0.0167732 | 0.0334399 | 0.0262815 | 0.027 | synonymous_variant | - | - | - |
| *EEF1A2* | Eukaryotic Translation Elongation Factor 1 Alpha 2 | [rs3818681](http://www.ncbi.nlm.nih.gov/projects/SNP/snp_ref.cgi?rs=rs3818681) | [20:62127326](genomebrowse://api/zoom?locus=20:62127326) | 0.0976438 | 0.0681716 | 0.0358414 | 0.067 | synonymous_variant | - | - | - |
| *EEFSEC* | Eukaryotic Elongation Factor, Selenocysteine-TRNA Specific | [rs11711710](http://www.ncbi.nlm.nih.gov/projects/SNP/snp_ref.cgi?rs=rs11711710) | [3:127872506](genomebrowse://api/zoom?locus=3:127872506) | 0.136182 | 0.115178 | 0.144592 | 0.117 | synonymous_variant | - | - | - |
| *EFCAB13* | EF-Hand Calcium Binding Domain 13 | [rs76299620](http://www.ncbi.nlm.nih.gov/projects/SNP/snp_ref.cgi?rs=rs76299620) | [17:45447802](genomebrowse://api/zoom?locus=17:45447802) | 0.0563099 | 0.0327067 | 0.0220664 | 0.033 | splice_acceptor_variant | - | - | Damaging |
| *EFCAB13* | EF-Hand Calcium Binding Domain 13 | [rs72825679](http://www.ncbi.nlm.nih.gov/projects/SNP/snp_ref.cgi?rs=rs72825679) | [17:45468842](genomebrowse://api/zoom?locus=17:45468842) | 0.0892572 | 0.0720291 | 0.0571549 | 0.073 | missense_variant | Damaging | Probably damaging | Damaging |
| *EFCAB13* | EF-Hand Calcium Binding Domain 13 | [rs118004742](http://www.ncbi.nlm.nih.gov/projects/SNP/snp_ref.cgi?rs=rs118004742) | [17:45468858](genomebrowse://api/zoom?locus=17:45468858) | 0.0231629 | 0.0440804 | 0.0355987 | 0.044 | stop_gained | - | - | Damaging |
| *EFCAB4A* | EF-Hand Calcium-Binding Domain-Containing Protein 4A | [rs72847210](http://www.ncbi.nlm.nih.gov/projects/SNP/snp_ref.cgi?rs=rs72847210) | [11:830383](genomebrowse://api/zoom?locus=11:830383) | 0.0165735 | 0.0319032 | 0.0318639 | 0.016 | intron_variant | - | - | - |
| *EFCAB6* | EF-Hand Calcium Binding Domain 6 | rs146421624 | 22:44168935 | 0.00319489 | 0.00542556 | 0.00303422 | 0.005609 | missense_variant | Tolerated | Possibly damaging | Damaging |
| *EFCAB6* | EF-Hand Calcium Binding Domain 6 | [rs137731](http://www.ncbi.nlm.nih.gov/projects/SNP/snp_ref.cgi?rs=rs137731) | [22:44031042](genomebrowse://api/zoom?locus=22:44031042) | 0.0161741 | 0.0306231 | 0.0360122 | 0.03 | missense_variant | Tolerated | Benign | Tolerated |
| *EFCAB6* | EF-Hand Calcium Binding Domain 6 | [rs137794](http://www.ncbi.nlm.nih.gov/projects/SNP/snp_ref.cgi?rs=rs137794) | [22:44079680](genomebrowse://api/zoom?locus=22:44079680) | 0.0758786 | 0.107111 | 0.114709 | 0.103 | missense_variant | Tolerated | Benign | Tolerated |
| *EFCAB7* | EF-Hand Calcium Binding Domain 7 | rs41311162 | 1:64022923 | 0.000399361 | 0.0015215 | 0.00142238 | 0.00154 | splice_region_variant | - | - | - |
| *EFCAB7* | EF-Hand Calcium Binding Domain 7 | [rs6693255](http://www.ncbi.nlm.nih.gov/projects/SNP/snp_ref.cgi?rs=rs6693255) | [1:63999826](genomebrowse://api/zoom?locus=1:63999826) | 0.144968 | 0.161127 | 0.128462 | 0.158 | missense_variant | Damaging | Benign | Tolerated |
| *EFHB* | EF-Hand Domain Family Member B | [rs17180771](http://www.ncbi.nlm.nih.gov/projects/SNP/snp_ref.cgi?rs=rs17180771) | [3:19975139](genomebrowse://api/zoom?locus=3:19975139) | 0.105431 | 0.18769 | 0.17727 | 0.185 | synonymous_variant | - | - | - |
| *EFHC1* | EF-Hand Domain Containing 1 | rs116338308 | 6:52317844 | 0.0081869 | 0.00109555 | 0.00545584 | 0.0007781 | intron_variant | - | - | - |
| *EFHC1* | EF-Hand Domain Containing 1 | rs35648306 | 6:52357109 | 0.00878594 | 0.00171937 | 0.00762028 | 0.00224 | synonymous_variant | - | - | - |
| *EFHC1* | EF-Hand Domain Containing 1 | rs74543871 | 6:52358475 | 0.00838658 | 0.0013674 | 0.0052141 | 0.0006307 | 3_prime_UTR_variant | - | - | - |
| *EFHC1* | EF-Hand Domain Containing 1 | c.*2368C>T | [6:52359507](genomebrowse://api/zoom?locus=6:52359507) | - | - | - | - | 3_prime_UTR_variant | - | - | - |
| *EFHC2* | EF-Hand Domain Containing 2 | rs374228583 | X:44023153 | 0.000529801 | 0.000948552 | 0.00120276 | 0.0007417 | intron_variant | - | - | - |
| *EFHD2* | EF-Hand Domain Family Member D2 | rs191564157 | 1:15754460 | 0.000399361 | 0.00180515 | 0.00151789 | 0.0008039 | intron_variant | - | - | - |
| *EFNA4* | Ephrin A4 | rs115929779 | 1:155036437 | 0.0071885 | 0.00121669 | 0.00690841 | 0.001177 | intron_variant | - | - | - |
| *EGF* | Epidermal Growth Factor | rs11569111 | 4:110925738 | 0.00399361 | 0.00104853 | 0.00416586 | 0.001343 | missense_variant | Damaging | Benign | Tolerated |
| *EGF* | Epidermal Growth Factor | [rs11568937](http://www.ncbi.nlm.nih.gov/projects/SNP/snp_ref.cgi?rs=rs11568937) | [4:110882051](genomebrowse://api/zoom?locus=4:110882051) | 0.0165735 | 0.0324379 | 0.0283988 | 0.032 | synonymous_variant | - | - | - |
| *EGFL7* | EGF Like Domain Multiple 7 | rs78568191 | 9:139566748 | 0.00339457 | 0.00747905 | 0.00481982 | 0.00645 | 3_prime_UTR_variant | - | - | - |
| *EGLN1* | Egl-9 Family Hypoxia Inducible Factor 1 | rs61734647 | 1:231502166 | 0.00259585 | 0.000467062 | 0.00197271 | 0.000593 | synonymous_variant | - | - | - |
| *EHBP1* | EH Domain Binding Protein 1 | [rs17432615](http://www.ncbi.nlm.nih.gov/projects/SNP/snp_ref.cgi?rs=rs17432615) | [2:63176139](genomebrowse://api/zoom?locus=2:63176139) | 0.0613019 | 0.101754 | 0.101713 | 0.105 | missense_variant | Damaging | Possibly damaging | Damaging |
| *EHHADH* | Enoyl-CoA Hydratase And 3-Hydroxyacyl CoA Dehydrogenase | [rs2302819](http://www.ncbi.nlm.nih.gov/projects/SNP/snp_ref.cgi?rs=rs2302819) | [3:184922294](genomebrowse://api/zoom?locus=3:184922294) | 0.0894569 | 0.0479045 | 0.0592968 | 0.048 | missense_variant | Damaging | Probably damaging | Damaging |
| *EHMT1* | Euchromatic Histone Lysine Methyltransferase 1 | [rs12341990](http://www.ncbi.nlm.nih.gov/projects/SNP/snp_ref.cgi?rs=rs12341990) | [9:140669541](genomebrowse://api/zoom?locus=9:140669541) | 0.0577077 | 0.0139997 | 0.0506341 | 0.016 | intron_variant | - | - | - |
| *EIF2B3* | Eukaryotic Translation Initiation Factor 2B Subunit Gamma | [rs72887005](http://www.ncbi.nlm.nih.gov/projects/SNP/snp_ref.cgi?rs=rs72887005) | [1:45444136](genomebrowse://api/zoom?locus=1:45444136) | 0.0682907 | 0.015867 | 0.0652188 | 0.02 | splice_region_variant | - | - | - |
| *EIF3A* | Eukaryotic Translation Initiation Factor 3 Subunit A | rs61729179 | 10:120829071 | 0.00838658 | 0.00191292 | 0.00852108 | 0.002619 | synonymous_variant | - | - | - |
| *EIF3D* | Eukaryotic Translation Initiation Factor 3 Subunit D | rs61730261 | 22:36921733 | 0.00519169 | 0.00173661 | 0.00723187 | 0.002125 | synonymous_variant | - | - | - |
| *EIF4ENIF1* | Eukaryotic Translation Initiation Factor 4E Nuclear Import Factor 1 | [rs5753627](http://www.ncbi.nlm.nih.gov/projects/SNP/snp_ref.cgi?rs=rs5753627) | [22:31859859](genomebrowse://api/zoom?locus=22:31859859) | 0.139177 | 0.10456 | 0.0755363 | 0.109 | synonymous_variant | - | - | - |
| *EIF4G1* | Eukaryotic Translation Initiation Factor 4 Gamma 1 | rs144580979 | 3:184040527 | 0.00179712 | 0.000511629 | 0.0018729 | 0.0005354 | intron_variant | - | - | - |
| *EIF4G1* | Eukaryotic Translation Initiation Factor 4 Gamma 1 | rs74627110 | 3:184049766 | 0.00499201 | 0.00121868 | 0.00468467 | 0.001408 | synonymous_variant | - | - | - |
| *EIF4G1* | Eukaryotic Translation Initiation Factor 4 Gamma 1 | [rs2293605](http://www.ncbi.nlm.nih.gov/projects/SNP/snp_ref.cgi?rs=rs2293605) | [3:184044433](genomebrowse://api/zoom?locus=3:184044433) | 0.063099 | 0.10746 | 0.0975775 | 0.106 | intron_variant | - | - | - |
| *EIF5AL1* | Eukaryotic Translation Initiation Factor 5A Like 1 | [rs200745296](http://www.ncbi.nlm.nih.gov/projects/SNP/snp_ref.cgi?rs=rs200745296) | [10:81272720](genomebrowse://api/zoom?locus=10:81272720) | 0.0191693 | 0.0287562 | 0.0107422 | 0.058 | synonymous_variant | - | - | - |
| *ELANE* | Elastase, Neutrophil Expressed | [rs17223045](http://www.ncbi.nlm.nih.gov/projects/SNP/snp_ref.cgi?rs=rs17223045) | [19:855587](genomebrowse://api/zoom?locus=19:855587) | 0.0249601 | 0.0147925 | 0.0263328 | 0.016 | synonymous_variant | - | - | - |
| *ELAVL2* | ELAV Like RNA Binding Protein 2 | [rs73654364](http://www.ncbi.nlm.nih.gov/projects/SNP/snp_ref.cgi?rs=rs73654364) | [9:23762176](genomebrowse://api/zoom?locus=9:23762176) | 0.0557109 | 0.0465648 | 0.0377175 | 0.043 | synonymous_variant | - | - | - |
| *ELF1* | E74 Like ETS Transcription Factor 1 | c.1348A>G | [13:41508073](genomebrowse://api/zoom?locus=13:41508073) | - | - | - | - | missense_variant | Tolerated | Probably damaging | Damaging |
| *ELMO3* | Engulfment And Cell Motility 3 | [rs73586989](http://www.ncbi.nlm.nih.gov/projects/SNP/snp_ref.cgi?rs=rs73586989) | [16:67234741](genomebrowse://api/zoom?locus=16:67234741) | 0.122005 | 0.0780929 | 0.124805 | 0.083 | intron_variant | - | - | - |
| *ELMOD2* | ELMO Domain Containing 2 | c.143-24delT | [4:141448596](genomebrowse://api/zoom?locus=4:141448596) | - | 0.336199 | 0.0103132 | 0.268 | intron_variant | - | - | - |
| *ELP2* | Elongator Acetyltransferase Complex Subunit 2 | rs77729794 | 18:33713185 | 0.00519169 | 0.00105179 | 0.00351999 | 0.001227 | intron_variant | - | - | - |
| *ELP2* | Elongator Acetyltransferase Complex Subunit 2 | [rs16967474](http://www.ncbi.nlm.nih.gov/projects/SNP/snp_ref.cgi?rs=rs16967474) | [18:33726295](genomebrowse://api/zoom?locus=18:33726295) | 0.0700879 | 0.0191629 | 0.0435372 | 0.023 | missense_variant | Damaging | Possibly damaging | Damaging |
| *ELP2* | Elongator Acetyltransferase Complex Subunit 2 | [rs73428961](http://www.ncbi.nlm.nih.gov/projects/SNP/snp_ref.cgi?rs=rs73428961) | [18:33736418](genomebrowse://api/zoom?locus=18:33736418) | 0.0702875 | 0.0191768 | 0.0436639 | 0.023 | intron_variant | - | - | - |
| *ELP6* | Elongator Acetyltransferase Complex Subunit 6 | rs116174668 | 3:47537594 | 0.0091853 | 0.00179519 | 0.00668259 | 0.002166 | missense_variant | Damaging | Possibly damaging | Damaging |
| *ELP6* | Elongator Acetyltransferase Complex Subunit 6 | [rs72913126](http://www.ncbi.nlm.nih.gov/projects/SNP/snp_ref.cgi?rs=rs72913126) | [3:47537515](genomebrowse://api/zoom?locus=3:47537515) | 0.0842652 | 0.0197403 | 0.0771296 | 0.024 | 3_prime_UTR_variant | - | - | - |
| *EMR4P* | Egf-Like Module Containing, Mucin-Like, Hormone Receptor-Like 4 | [rs12984813](http://www.ncbi.nlm.nih.gov/projects/SNP/snp_ref.cgi?rs=rs12984813) | [19:6990724](genomebrowse://api/zoom?locus=19:6990724) | 0.11222 | - | 0.0966291 | - | intron_variant | - | - | - |
| *EN1* | Engrailed Homeobox 1 | [rs79126103](http://www.ncbi.nlm.nih.gov/projects/SNP/snp_ref.cgi?rs=rs79126103) | [2:119604255](genomebrowse://api/zoom?locus=2:119604255) | 0.063099 | 0.0771612 | 0.0491036 | 0.065 | synonymous_variant | - | - | - |
| *ENAM* | Enamelin | [rs144929717](http://www.ncbi.nlm.nih.gov/projects/SNP/snp_ref.cgi?rs=rs144929717) | [4:71498303](genomebrowse://api/zoom?locus=4:71498303) | 0.103834 | - | 0.0906001 | - | intron_variant | - | - | - |
| *ENAM* | Enamelin | [rs2609428](http://www.ncbi.nlm.nih.gov/projects/SNP/snp_ref.cgi?rs=rs2609428) | [4:71508869](genomebrowse://api/zoom?locus=4:71508869) | 0.0393371 | 0.0122614 | 0.0361672 | 0.014 | missense_variant | Tolerated | Benign | Tolerated |
| *ENDOD1* | Endonuclease Domain Containing 1 | rs184192678 | 11:94861636 | 0.00139776 | 0.00233958 | 0.0025502 | 0.002315 | synonymous_variant | - | - | - |
| *ENGASE* | Endo-Beta-N-Acetylglucosaminidase | [rs62063818](http://www.ncbi.nlm.nih.gov/projects/SNP/snp_ref.cgi?rs=rs62063818) | [17:77073776](genomebrowse://api/zoom?locus=17:77073776) | 0.0409345 | 0.059341 | 0.0522663 | 0.059 | synonymous_variant | - | - | - |
| *ENPEP* | Glutamyl Aminopeptidase | [rs56280650](http://www.ncbi.nlm.nih.gov/projects/SNP/snp_ref.cgi?rs=rs56280650) | [4:111452312](genomebrowse://api/zoom?locus=4:111452312) | 0.0121805 | 0.0238672 | 0.0216716 | 0.02 | intron_variant | - | - | - |
| *ENPP1* | Ectonucleotide Pyrophosphatase/Phosphodiesterase 1 | [rs1804025](http://www.ncbi.nlm.nih.gov/projects/SNP/snp_ref.cgi?rs=rs1804025) | [6:132211534](genomebrowse://api/zoom?locus=6:132211534) | 0.0790735 | 0.0337463 | 0.070695 | 0.039 | synonymous_variant | - | - | - |
| *ENPP3* | Ectonucleotide Pyrophosphatase/Phosphodiesterase 3 | [rs9483324](http://www.ncbi.nlm.nih.gov/projects/SNP/snp_ref.cgi?rs=rs9483324) | [6:132022419](genomebrowse://api/zoom?locus=6:132022419) | 0.299521 | 0.190121 | 0.221892 | 0.188 | synonymous_variant | - | - | - |
| *ENPP4* | Ectonucleotide Pyrophosphatase/Phosphodiesterase 4 | [rs9472696](http://www.ncbi.nlm.nih.gov/projects/SNP/snp_ref.cgi?rs=rs9472696) | [6:46108916](genomebrowse://api/zoom?locus=6:46108916) | 0.0934505 | 0.0830024 | 0.107576 | 0.083 | synonymous_variant | - | - | - |
| *ENTPD3* | Ectonucleoside Triphosphate Diphosphohydrolase 3 | rs35071108 | 3:40464469 | 0.00599042 | 0.00176446 | 0.00549167 | 0.001969 | synonymous_variant | - | - | - |
| *EOMES* | Eomesodermin | rs539715811 | 3:27763687 | 0.000199681 | 5.21871e-05 | 0.000390041 | 5.917e-05 | synonymous_variant | - | - | - |
| *EP400* | E1A Binding Protein P400 | [rs11833839](http://www.ncbi.nlm.nih.gov/projects/SNP/snp_ref.cgi?rs=rs11833839) | [12:132560957](genomebrowse://api/zoom?locus=12:132560957) | 0.132987 | 0.0802393 | 0.133758 | 0.059 | intron_variant | - | - | - |
| *EPB41L2* | Erythrocyte Membrane Protein Band 4.1 Like 2 | rs138467790 | 6:131276437 | 0.000399361 | 6.09835e-05 | 0.000163324 | 8.236e-05 | missense_variant | Damaging | Possibly damaging | Tolerated |
| *EPB42* | Erythrocyte Membrane Protein Band 4.2 | [rs1042168](http://www.ncbi.nlm.nih.gov/projects/SNP/snp_ref.cgi?rs=rs1042168) | [15:43507393](genomebrowse://api/zoom?locus=15:43507393) | 0.0425319 | 0.0117477 | 0.0369999 | 0.014 | synonymous_variant | - | - | - |
| *EPC1* | Enhancer Of Polycomb Homolog 1 | rs147945675 | 10:32581429 | 0.00199681 | 0.00110216 | 0.00116324 | 0.000972 | synonymous_variant | - | - | - |
| *EPCAM* | Epithelial Cell Adhesion Molecule | rs146480420 | 2:47601029 | 0.000998403 | 0.00268832 | 0.00280917 | 0.002578 | missense_variant | Damaging | Probably damaging | Damaging |
| *EPCAM* | Epithelial Cell Adhesion Molecule | [rs78608315](http://www.ncbi.nlm.nih.gov/projects/SNP/snp_ref.cgi?rs=rs78608315) | [2:47604148](genomebrowse://api/zoom?locus=2:47604148) | 0.0101837 | 0.0249027 | 0.0283518 | 0.025 | splice_region_variant | - | - | - |
| *EPHA10* | EPH Receptor A10 | [rs2170809](http://www.ncbi.nlm.nih.gov/projects/SNP/snp_ref.cgi?rs=rs2170809) | [1:38185647](genomebrowse://api/zoom?locus=1:38185647) | 0.0555112 | 0.0702711 | 0.0715163 | 0.069 | synonymous_variant | - | - | - |
| *EPHA10* | EPH Receptor A10 | [rs16824420](http://www.ncbi.nlm.nih.gov/projects/SNP/snp_ref.cgi?rs=rs16824420) | [1:38188697](genomebrowse://api/zoom?locus=1:38188697) | 0.0565096 | 0.0706911 | 0.0712528 | 0.07 | intron_variant | - | - | - |
| *EPHA2* | EPH Receptor A2 | rs55655135 | 1:16459832 | 0.00239617 | 0.00561259 | 0.00447152 | 0.004991 | synonymous_variant | - | - | - |
| *EPHA2* | EPH Receptor A2 | rs142079142 | 1:16464376 | 0.000998403 | 0.000180102 | 0.000969055 | 0.0002142 | synonymous_variant | - | - | - |
| *EPHA7* | EPH Receptor A7 | rs41273629 | 6:94120318 | 0.00119808 | 0.00155673 | 0.000840228 | 0.001598 | missense_variant | Tolerated | Benign | Tolerated |
| *EPHA7* | EPH Receptor A7 | [rs2278107](http://www.ncbi.nlm.nih.gov/projects/SNP/snp_ref.cgi?rs=rs2278107) | [6:94120639](genomebrowse://api/zoom?locus=6:94120639) | 0.0650958 | 0.0523881 | 0.0394932 | 0.05 | missense_variant | Tolerated | Benign | Damaging |
| *EPHB6* | EPH Receptor B6 | [rs8177146](http://www.ncbi.nlm.nih.gov/projects/SNP/snp_ref.cgi?rs=rs8177146) | [7:142563253](genomebrowse://api/zoom?locus=7:142563253) | 0.0682907 | 0.038549 | 0.0478089 | 0.039 | missense_variant | - | - | - |
| *EPHB6* | EPH Receptor B6 | [rs8177153](http://www.ncbi.nlm.nih.gov/projects/SNP/snp_ref.cgi?rs=rs8177153) | [7:142565385](genomebrowse://api/zoom?locus=7:142565385) | 0.0435304 | 0.0322052 | 0.025416 | 0.032 | synonymous_variant | - | - | - |
| *EPHB6* | EPH Receptor B6 | [rs8177158](http://www.ncbi.nlm.nih.gov/projects/SNP/snp_ref.cgi?rs=rs8177158) | [7:142565743](genomebrowse://api/zoom?locus=7:142565743) | 0.0664936 | 0.0285043 | 0.066561 | 0.026 | intron_variant | - | - | - |
| *EPHB6* | EPH Receptor B6 | [rs2229873](http://www.ncbi.nlm.nih.gov/projects/SNP/snp_ref.cgi?rs=rs2229873) | [7:142565776](genomebrowse://api/zoom?locus=7:142565776) | 0.04373 | 0.0323645 | 0.0252153 | 0.028 | synonymous_variant | - | - | - |
| *EPHB6* | EPH Receptor B6 | [rs4987690](http://www.ncbi.nlm.nih.gov/projects/SNP/snp_ref.cgi?rs=rs4987690) | [7:142568518](genomebrowse://api/zoom?locus=7:142568518) | 0.0660942 | 0.0296989 | 0.066345 | 0.033 | intron_variant | - | - | - |
| *EPN3* | Epsin 3 | [rs113914602](http://www.ncbi.nlm.nih.gov/projects/SNP/snp_ref.cgi?rs=rs113914602) | [17:48619175](genomebrowse://api/zoom?locus=17:48619175) | 0.0441294 | 0.0105951 | 0.0390989 | 0.013 | intron_variant | - | - | - |
| *EPPK1* | Epiplakin 1 | rs139952490 | 8:144943223 | 0.0081869 | 0.00162927 | 0.0050974 | 0.001886 | missense_variant | - | Benign | Tolerated |
| *EPRS* | Glutamyl-Prolyl-TRNA Synthetase | [rs5030751](http://www.ncbi.nlm.nih.gov/projects/SNP/snp_ref.cgi?rs=rs5030751) | [1:220162029](genomebrowse://api/zoom?locus=1:220162029) | 0.0433307 | 0.0100723 | 0.0359082 | 0.013 | missense_variant | Damaging | Benign | Tolerated |
| *EPS8L1* | EPS8 Like 1 | [rs17700138](http://www.ncbi.nlm.nih.gov/projects/SNP/snp_ref.cgi?rs=rs17700138) | [19:55593417](genomebrowse://api/zoom?locus=19:55593417) | 0.126797 | 0.173768 | 0.166602 | 0.121 | intron_variant | - | - | - |
| *EPYC* | Epiphycan | [rs17784152](http://www.ncbi.nlm.nih.gov/projects/SNP/snp_ref.cgi?rs=rs17784152) | [12:91366649](genomebrowse://api/zoom?locus=12:91366649) | 0.0259585 | 0.0382666 | 0.0188655 | 0.035 | missense_variant | Damaging | Probably damaging | Damaging |
| *ERAP1* | Endoplasmic Reticulum Aminopeptidase 1 | [rs3734016](http://www.ncbi.nlm.nih.gov/projects/SNP/snp_ref.cgi?rs=rs3734016) | [5:96139464](genomebrowse://api/zoom?locus=5:96139464) | 0.0858626 | 0.0703995 | 0.0647831 | 0.066 | missense_variant | Tolerated | Benign | Tolerated |
| *ERBB2* | Erb-B2 Receptor Tyrosine Kinase 2 | [rs2230698](http://www.ncbi.nlm.nih.gov/projects/SNP/snp_ref.cgi?rs=rs2230698) | [17:37872050](genomebrowse://api/zoom?locus=17:37872050) | 0.0451278 | 0.0109967 | 0.0396856 | 0.013 | synonymous_variant | - | - | - |
| *ERBB4* | Erb-B2 Receptor Tyrosine Kinase 4 | [rs139728050](http://www.ncbi.nlm.nih.gov/projects/SNP/snp_ref.cgi?rs=rs139728050) | [2:212426588](genomebrowse://api/zoom?locus=2:212426588) | 0.0127796 | 0.0375314 | 0.0473606 | 0.037 | intron_variant | - | - | - |
| *ERC2* | ELKS/RAB6-Interacting/CAST Family Member 2 | rs201790802 | 3:56207545 | 0.000998403 | 0.000410579 | 0.00184097 | 0.0004707 | synonymous_variant | - | - | - |
| *ERC2* | ELKS/RAB6-Interacting/CAST Family Member 2 | [rs56227347](http://www.ncbi.nlm.nih.gov/projects/SNP/snp_ref.cgi?rs=rs56227347) | [3:56330368](genomebrowse://api/zoom?locus=3:56330368) | 0.057508 | 0.02429 | 0.0591294 | 0.027 | synonymous_variant | - | - | - |
| *ERCC3* | ERCC Excision Repair 3, TFIIH Core Complex Helicase Subunit | rs200833462 | 2:128046899 | 0.000798722 | 0.00110548 | 0.00074275 | 0.001227 | intron_variant | - | - | - |
| *ERCC4* | ERCC Excision Repair 4, Endonuclease Catalytic Subunit | [rs2020955](http://www.ncbi.nlm.nih.gov/projects/SNP/snp_ref.cgi?rs=rs2020955) | [16:14038659](genomebrowse://api/zoom?locus=16:14038659) | 0.0611022 | 0.0153673 | 0.0593478 | 0.019 | missense_variant | Tolerated | Benign | Tolerated |
| *ERCC6* | ERCC Excision Repair 6, Chromatin Remodeling Factor | rs4253227 | 10:50667229 | 0.00479233 | 0.00101314 | 0.00322872 | 0.00117 | missense_variant | Damaging | Probably damaging | Damaging |
| *ERG* | ETS Transcription Factor ERG | [rs17853963](http://www.ncbi.nlm.nih.gov/projects/SNP/snp_ref.cgi?rs=rs17853963) | [21:39775468](genomebrowse://api/zoom?locus=21:39775468) | 0.0145767 | 0.0242475 | 0.0213206 | 0.024 | synonymous_variant | - | - | - |
| *ERMARD* | ER Membrane Associated RNA Degradation | rs559633528 | 6:170176651 | 0.000199681 | 3.25598e-05 | 6.45703e-05 | 1.647e-05 | missense_variant | Tolerated | Benign | Tolerated |
| *ESPL1* | Extra Spindle Pole Bodies Like 1, Separase | rs200442902 | 12:53683027 | 0.000399361 | 0.000142871 | 0.000129207 | 0.0001318 | missense_variant | Damaging | Benign | Tolerated |
| *ESPNP* | Espin Pseudogene | [rs201754604](http://www.ncbi.nlm.nih.gov/projects/SNP/snp_ref.cgi?rs=rs201754604) | [1:17017747](genomebrowse://api/zoom?locus=1:17017747) | - | 0.0201694 | 0.024535 | 0.031 | non_coding_exon_variant | - | - | - |
| *ESRRB* | Estrogen Related Receptor Beta | [rs61742642](http://www.ncbi.nlm.nih.gov/projects/SNP/snp_ref.cgi?rs=rs61742642) | [14:76964655](genomebrowse://api/zoom?locus=14:76964655) | 0.0700879 | 0.0766487 | 0.0564464 | 0.071 | missense_variant | Tolerated | Benign | Damaging |
| *ESRRG* | Estrogen Related Receptor Gamma | [rs10863247](http://www.ncbi.nlm.nih.gov/projects/SNP/snp_ref.cgi?rs=rs10863247) | [1:216692741](genomebrowse://api/zoom?locus=1:216692741) | 0.0932508 | 0.0226607 | 0.0837373 | 0.028 | synonymous_variant | - | - | - |
| *ESYT3* | Extended Synaptotagmin 3 | [rs35537868](http://www.ncbi.nlm.nih.gov/projects/SNP/snp_ref.cgi?rs=rs35537868) | [3:138191448](genomebrowse://api/zoom?locus=3:138191448) | 0.141973 | 0.0667146 | 0.0682935 | 0.068 | missense_variant | Tolerated | Benign | Tolerated |
| *ETNK2* | Ethanolamine Kinase 2 | [rs45596637](http://www.ncbi.nlm.nih.gov/projects/SNP/snp_ref.cgi?rs=rs45596637) | [1:204116624](genomebrowse://api/zoom?locus=1:204116624) | 0.0103834 | 0.0240412 | 0.0253567 | 0.024 | intron_variant | Tolerated | - | Tolerated |
| *ETNPPL* | Ethanolamine-Phosphate Phospho-Lyase | [rs10035050](http://www.ncbi.nlm.nih.gov/projects/SNP/snp_ref.cgi?rs=rs10035050) | [4:109665016](genomebrowse://api/zoom?locus=4:109665016) | 0.121206 | 0.0517693 | 0.109675 | 0.057 | splice_region_variant | - | - | - |
| *ETS2* | ETS Proto-Oncogene 2, Transcription Factor | rs61735785 | 21:40190408 | 0.00339457 | 0.00736131 | 0.00662273 | 0.007265 | missense_variant | Damaging | Possibly damaging | Damaging |
| *ETV2* | ETS Variant Transcription Factor 2 | [rs2285419](http://www.ncbi.nlm.nih.gov/projects/SNP/snp_ref.cgi?rs=rs2285419) | [19:36134208](genomebrowse://api/zoom?locus=19:36134208) | 0.122204 | 0.113965 | 0.107996 | 0.105 | missense_variant | Damaging | Probably damaging | Damaging |
| *EVC* | EvC Ciliary Complex Subunit 1 | rs113870218 | 4:5731158 | 0.0623003 | 0.0908995 | 0.0821918 | 0.091 | intron_variant | - | - | - |
| *EVC* |  | rs35953626 | 4:5755524 | 0.0728834 | 0.0171529 | 0.0655589 | 0.021 | missense_variant | Tolerated | Benign | Tolerated |
| *EVC2* | EvC Ciliary Complex Subunit 2 | rs57901231 | 4:5567110 | 0.00279553 | 0.000877792 | 0.00245668 | 0.0008154 | intron_variant | - | - | - |
| *EVC2* |  | rs60121553 | 4:5624530 | 0.0878594 | 0.0242489 | 0.0743703 | 0.029 | synonymous_variant | - | - | - |
| *EVPL* | Envoplakin | [rs7225323](http://www.ncbi.nlm.nih.gov/projects/SNP/snp_ref.cgi?rs=rs7225323) | [17:74005476](genomebrowse://api/zoom?locus=17:74005476) | 0.175519 | 0.0752243 | 0.173219 | 0.083 | synonymous_variant | - | - | - |
| *EVX1* | Even-Skipped Homeobox 1 | c.563C>T | [7:27284802](genomebrowse://api/zoom?locus=7:27284802) | - | - | - | - | missense_variant | Damaging | Probably damaging | Damaging |
| *EXO5* | Exonuclease 5 | [rs35672330](http://www.ncbi.nlm.nih.gov/projects/SNP/snp_ref.cgi?rs=rs35672330) | [1:40980668](genomebrowse://api/zoom?locus=1:40980668) | 0.0197684 | 0.0437098 | 0.0420247 | 0.041 | missense_variant | Damaging | Probably damaging | Tolerated |
| *EXOC6B* | Exocyst Complex Component 6B | rs75680051 | 2:72802618 | 0.00998403 | 0.00256939 | 0.00982102 | 0.003211 | splice_region_variant | - | - | - |
| *EXOC7* | Exocyst Complex Component 7 | rs140797843 | 17:74078113 | 0.00658946 | 0.00939888 | 0.00797614 | 0.009132 | missense_variant | Damaging | Benign | Damaging |
| *EXOSC10* | Exosome Component 10 | rs3737621 | 1:11150682 | 0.11242 | 0.0608716 | 0.0649225 | 0.061 | synonymous_variant | - | - | - |
| *EXT2* | Exostosin Glycosyltransferase 2 | rs138187791 | 11:44193165 | 0.000998403 | 0.00106399 | 0.000484246 | 0.0008154 | missense_variant | Tolerated | Benign | Damaging |
| *EXTL2* | Exostosin Like Glycosyltransferase 2 | [rs2207735](http://www.ncbi.nlm.nih.gov/projects/SNP/snp_ref.cgi?rs=rs2207735) | [1:101343264](genomebrowse://api/zoom?locus=1:101343264) | 0.04373 | 0.0112953 | 0.0362708 | 0.014 | synonymous_variant | - | - | - |
| *EYS* | Eyes Shut Homolog | rs74848648 | 6:64694325 | 0.00459265 | 0.000871863 | 0.00369267 | 0.001786 | missense_variant | Tolerated | Benign | Tolerated |
| *EYS* | Eyes Shut Homolog | rs200935518 | 6:65300869 | 0.000199681 | 0.000837928 | 0.000776347 | 0.0005568 | missense_variant | Damaging | Benign | Tolerated |
| *F10* | Coagulation Factor X | rs115112448 | 13:113783806 | 0.00738818 | 0.00169328 | 0.00614131 | 0.002092 | synonymous_variant | - | - | - |
| *F11* | Coagulation Factor XI | [rs5975](http://www.ncbi.nlm.nih.gov/projects/SNP/snp_ref.cgi?rs=rs5975) | [4:187208968](genomebrowse://api/zoom?locus=4:187208968) | 0.0377396 | 0.0128153 | 0.0290909 | 0.013 | synonymous_variant | - | - | - |
| *F11* | Coagulation Factor XI | [rs5971](http://www.ncbi.nlm.nih.gov/projects/SNP/snp_ref.cgi?rs=rs5971) | [4:187209702](genomebrowse://api/zoom?locus=4:187209702) | 0.0894569 | 0.0605589 | 0.0666236 | 0.061 | synonymous_variant | - | - | - |
| *F13B* | Coagulation Factor XIII B Chain | rs17549671 | 1:197019979 | 0.00638978 | 0.00196428 | 0.00652286 | 0.002414 | missense_variant | Tolerated | Benign | Tolerated |
| *F2* | Coagulation Factor II, Thrombin | rs5899 | 11:46747662 | 0.00638978 | 0.00861817 | 0.00526724 | 0.008582 | synonymous_variant | - | - | - |
| *F3* | Coagulation Factor III, Tissue Factor | rs143237190 | 1:95001655 | 0.00638978 | 0.00145371 | 0.00442449 | 0.001828 | missense_variant | Damaging | Probably damaging | Tolerated |
| *F5* | Coagulation Factor V | rs6026 | 1:169497292 | 0.00758786 | 0.00205549 | 0.0068471 | 0.002487 | missense_variant | Damaging | Probably damaging | Damaging |
| *F5* | Coagulation Factor V | [rs9332701](http://www.ncbi.nlm.nih.gov/projects/SNP/snp_ref.cgi?rs=rs9332701) | [1:169484767](genomebrowse://api/zoom?locus=1:169484767) | 0.0113818 | 0.0305438 | 0.0320786 | 0.031 | missense_variant | Damaging | Probably damaging | Damaging |
| *F5* | Coagulation Factor V | [rs6035](http://www.ncbi.nlm.nih.gov/projects/SNP/snp_ref.cgi?rs=rs6035) | [1:169521849](genomebrowse://api/zoom?locus=1:169521849) | 0.0830671 | 0.0877282 | 0.0796137 | 0.086 | synonymous_variant | - | - | - |
| *F5* | Coagulation Factor V | [rs6033](http://www.ncbi.nlm.nih.gov/projects/SNP/snp_ref.cgi?rs=rs6033) | [1:169521853](genomebrowse://api/zoom?locus=1:169521853) | 0.0501198 | 0.0757197 | 0.0682495 | 0.072 | missense_variant | Tolerated | Benign | Tolerated |
| *F5* | Coagulation Factor V | [rs6023](http://www.ncbi.nlm.nih.gov/projects/SNP/snp_ref.cgi?rs=rs6023) | [1:169528384](genomebrowse://api/zoom?locus=1:169528384) | 0.0323482 | 0.0585166 | 0.0517498 | 0.058 | splice_region_variant | - | - | - |
| *FAH* | Fumarylacetoacetate Hydrolase | [rs60585303](http://www.ncbi.nlm.nih.gov/projects/SNP/snp_ref.cgi?rs=rs60585303) | [15:80473505](genomebrowse://api/zoom?locus=15:80473505) | 0.0459265 | 0.011489 | 0.0439141 | 0.015 | splice_region_variant | - | - | - |
| *FAM107A* | Family With Sequence Similarity 107 Member A | rs11539082 | 3:58553039 | 0.00379393 | 0.000817416 | 0.0037475 | 0.001104 | missense_variant | Damaging | Probably damaging | Damaging |
| *FAM107A* | Family With Sequence Similarity 107 Member A | [rs1139701](http://www.ncbi.nlm.nih.gov/projects/SNP/snp_ref.cgi?rs=rs1139701) | [3:58552950](genomebrowse://api/zoom?locus=3:58552950) | 0.0173722 | 0.037157 | 0.0420304 | 0.037 | synonymous_variant | - | - | - |
| *FAM115C* | Family With Sequence Similarity 115 Member C | [rs1464828](http://www.ncbi.nlm.nih.gov/projects/SNP/snp_ref.cgi?rs=rs1464828) | [7:143321094](genomebrowse://api/zoom?locus=7:143321094) | 0.518371 | 0.121675 | 0.21678 | 0.158 | intron_variant | - | - | - |
| *FAM120A* | Family With Sequence Similarity 120A | [rs77023914](http://www.ncbi.nlm.nih.gov/projects/SNP/snp_ref.cgi?rs=rs77023914) | [9:96278474](genomebrowse://api/zoom?locus=9:96278474) | 0.151957 | 0.0655128 | 0.126611 | 0.07 | synonymous_variant | - | - | - |
| *FAM126A* | Family With Sequence Similarity 126 Member A | rs59318769 | 7:22999843 | 0.00119808 | 0.00038283 | 0.00132446 | 0.000453 | intron_variant | - | - | - |
| *FAM134B* | Family With Sequence Similarity 134 Member B | rs61741225 | 5:16565892 | 0.00698882 | 0.00217473 | 0.00887841 | 0.002558 | synonymous_variant | - | - | - |
| *FAM135B* | Family With Sequence Similarity 135 Member B | [rs2280848](http://www.ncbi.nlm.nih.gov/projects/SNP/snp_ref.cgi?rs=rs2280848) | [8:139144914](genomebrowse://api/zoom?locus=8:139144914) | 0.0746805 | 0.0527095 | 0.0409508 | 0.05 | synonymous_variant | - | - | - |
| *FAM13A* | Family With Sequence Similarity 13 Member A | rs60527761 | 4:89941657 | 0.0091853 | 0.00237677 | 0.00899909 | 0.003113 | synonymous_variant | - | - | - |
| *FAM153C* | Family With Sequence Similarity 153 Member C | [rs200475370](http://www.ncbi.nlm.nih.gov/projects/SNP/snp_ref.cgi?rs=rs200475370) | [5:177457628](genomebrowse://api/zoom?locus=5:177457628) | 0.0301518 | 0.0523704 | 0.0334515 | 0.052 | intron_variant | - | - | - |
| *FAM153C* | Family With Sequence Similarity 153 Member C | [rs534078519](http://www.ncbi.nlm.nih.gov/projects/SNP/snp_ref.cgi?rs=rs534078519) | [5:177462077](genomebrowse://api/zoom?locus=5:177462077) | 0.0786741 | 0.0804919 | 0.161576 | 0.031 | intron_variant | - | - | - |
| *FAM153C* | Family With Sequence Similarity 153 Member C | [rs200007598](http://www.ncbi.nlm.nih.gov/projects/SNP/snp_ref.cgi?rs=rs200007598) | [5:177462960](genomebrowse://api/zoom?locus=5:177462960) | - | - | 0.190958 | - | intron_variant | - | - | - |
| *FAM178B* | Family With Sequence Similarity 178 Member B | [rs146972264](http://www.ncbi.nlm.nih.gov/projects/SNP/snp_ref.cgi?rs=rs146972264) | [2:97638311](genomebrowse://api/zoom?locus=2:97638311) | 0.0221645 | 0.0350562 | 0.0253925 | 0.026 | missense_variant | Tolerated | - | Tolerated |
| *FAM179B* | Family With Sequence Similarity 179 Member B | [rs3742591](http://www.ncbi.nlm.nih.gov/projects/SNP/snp_ref.cgi?rs=rs3742591) | [14:45433155](genomebrowse://api/zoom?locus=14:45433155) | 0.0543131 | 0.0584848 | 0.0440692 | 0.058 | missense_variant | Tolerated | Probably damaging | Damaging |
| *FAM182B* | Family With Sequence Similarity 182 Member B | rs535435761 | 20:25755737 | 0.000798722 | 0.000577895 | 0.000161865 | 0.0006652 | intron_variant | - | - | - |
| *FAM184B* | Family With Sequence Similarity 184 Member B | rs559118491 | 4:17660012 | 0.000798722 | 0.000188372 | 0.00103386 | 0.0001547 | synonymous_variant | - | - | - |
| *FAM184B* | Family With Sequence Similarity 184 Member B | [rs2302392](http://www.ncbi.nlm.nih.gov/projects/SNP/snp_ref.cgi?rs=rs2302392) | [4:17660051](genomebrowse://api/zoom?locus=4:17660051) | 0.086861 | 0.0338826 | 0.0685273 | 0.034 | synonymous_variant | - | - | - |
| *FAM189A1* | Family With Sequence Similarity 189 Member A1 | [rs61741714](http://www.ncbi.nlm.nih.gov/projects/SNP/snp_ref.cgi?rs=rs61741714) | [15:29429347](genomebrowse://api/zoom?locus=15:29429347) | 0.0123802 | 0.0253468 | 0.0228697 | 0.022 | synonymous_variant | - | - | - |
| *FAM205B* | Family With Sequence Similarity 205 Member B | [rs146266330](http://www.ncbi.nlm.nih.gov/projects/SNP/snp_ref.cgi?rs=rs146266330) | [9:34834134](genomebrowse://api/zoom?locus=9:34834134) | 0.0169728 | 0.0237166 | 0.024045 | 0.02 | non_coding_exon_variant | - | - | - |
| *FAM208A* | Family With Sequence Similarity 208 Member A | [rs56758331](http://www.ncbi.nlm.nih.gov/projects/SNP/snp_ref.cgi?rs=rs56758331) | [3:56661586](genomebrowse://api/zoom?locus=3:56661586) | 0.0569089 | 0.049236 | 0.0522542 | 0.052 | intron_variant | - | - | - |
| *FAM208B* | Family With Sequence Similarity 208 Member B | rs369448239 | 10:5781699 | 0.000798722 | 0.000300583 | 0.00116212 | 0.0003144 | synonymous_variant | - | - | - |
| *FAM20A* | FAM20A Golgi Associated Secretory Pathway Pseudokinase | [rs77669268](http://www.ncbi.nlm.nih.gov/projects/SNP/snp_ref.cgi?rs=rs77669268) | [17:66532545](genomebrowse://api/zoom?locus=17:66532545) | 0.0139776 | 0.0267671 | 0.0229855 | 0.025 | 3_prime_UTR_variant | - | - | - |
| *FAM211A* | Leucine-Rich Repeat-Containing Protein FAM211A | [rs61745139](http://www.ncbi.nlm.nih.gov/projects/SNP/snp_ref.cgi?rs=rs61745139) | [17:16347325](genomebrowse://api/zoom?locus=17:16347325) | 0.048123 | 0.0924169 | 0.0783413 | 0.096 | missense_variant | Damaging | Benign | Damaging |
| *FAM213A* | Family With Sequence Similarity 213 Member A | [rs17617713](http://www.ncbi.nlm.nih.gov/projects/SNP/snp_ref.cgi?rs=rs17617713) | [10:82191700](genomebrowse://api/zoom?locus=10:82191700) | 0.013778 | 0.0234189 | 0.0173405 | 0.022 | intron_variant | - | - | - |
| *FAM216A* | Family With Sequence Similarity 216 Member A | [rs17851852](http://www.ncbi.nlm.nih.gov/projects/SNP/snp_ref.cgi?rs=rs17851852) | [12:110927882](genomebrowse://api/zoom?locus=12:110927882) | 0.0103834 | 0.0223541 | 0.0169141 | 0.021 | synonymous_variant | - | - | - |
| *FAM21B* | Family With Sequence Similarity 21 Member B | [rs201672329](http://www.ncbi.nlm.nih.gov/projects/SNP/snp_ref.cgi?rs=rs201672329) | [10:47911549](genomebrowse://api/zoom?locus=10:47911549) | 0.047524 | 0.0401138 | 0.0455196 | 0.038 | synonymous_variant | - | - | - |
| *FAM26F* | Family With Sequence Similarity 26 Member F | rs73767784 | 6:116784759 | 0.00499201 | 0.00139717 | 0.00607078 | 0.00173 | missense_variant | Damaging | Possibly damaging | Damaging |
| *FAM27E1* | Family With Sequence Similarity 27 Member E1 | [rs75215693](http://www.ncbi.nlm.nih.gov/projects/SNP/snp_ref.cgi?rs=rs75215693) | [9:46386688](genomebrowse://api/zoom?locus=9:46386688) | - | 0.619252 | 0.497766 | 0.213 | non_coding_exon_variant | - | - | - |
| *FAM35DP* | Family With Sequence Similarity 35 Member DP | [rs201541056](http://www.ncbi.nlm.nih.gov/projects/SNP/snp_ref.cgi?rs=rs201541056) | [10:47400681](genomebrowse://api/zoom?locus=10:47400681) | - | 0.0828663 | 0.131189 | 0.016 | non_coding_exon_variant | - | - | - |
| *FAM3B* | FAM3 Metabolism Regulating Signaling Molecule B | [rs111988437](http://www.ncbi.nlm.nih.gov/projects/SNP/snp_ref.cgi?rs=rs111988437) | [21:42720579](genomebrowse://api/zoom?locus=21:42720579) | 0.04373 | 0.0313452 | 0.0293795 | 0.031 | missense_variant | Tolerated | Benign | Tolerated |
| *FAM3D* | FAM3 Metabolism Regulating Signaling Molecule D | [rs33966924](http://www.ncbi.nlm.nih.gov/projects/SNP/snp_ref.cgi?rs=rs33966924) | [3:58625875](genomebrowse://api/zoom?locus=3:58625875) | 0.0794728 | 0.134354 | 0.138706 | 0.133 | missense_variant | Tolerated | Benign | Tolerated |
| *FAM50B* | Family With Sequence Similarity 50 Member B | rs80012120 | 6:3850730 | 0.00379393 | 0.000830267 | 0.00320264 | 0.001054 | missense_variant | Tolerated | Benign | Tolerated |
| *FAM63A* | Family With Sequence Similarity 63 Member A | [rs41310885](http://www.ncbi.nlm.nih.gov/projects/SNP/snp_ref.cgi?rs=rs41310885) | [1:150974971](genomebrowse://api/zoom?locus=1:150974971) | 0.0533147 | 0.0513353 | 0.0783896 | 0.053 | missense_variant | Tolerated | Benign | Tolerated |
| *FAM63B* | Family With Sequence Similarity 63 Member B | c.899-17delT | [15:59094495](genomebrowse://api/zoom?locus=15:59094495) | - | 0.301552 | 0.0136044 | 0.155 | intron_variant | - | - | - |
| *FAM65C* | Family With Sequence Similarity 65 Member C | rs146773676 | 20:49224995 | 0.00379393 | 0.00557411 | 0.00822273 | 0.005395 | missense_variant | Tolerated | Benign | Damaging |
| *FAM65C* | Family With Sequence Similarity 65 Member C | [rs35976890](http://www.ncbi.nlm.nih.gov/projects/SNP/snp_ref.cgi?rs=rs35976890) | [20:49214188](genomebrowse://api/zoom?locus=20:49214188) | 0.024361 | 0.0507883 | 0.0475236 | 0.05 | synonymous_variant | - | - | - |
| *FAM66B* | Family With Sequence Similarity 66 Member B | [rs199562878](http://www.ncbi.nlm.nih.gov/projects/SNP/snp_ref.cgi?rs=rs199562878) | [8:7194685](genomebrowse://api/zoom?locus=8:7194685) | 0.0135783 | 0.0256962 | 0.0177665 | 0.022 | missense_variant | - | - | - |
| *FAM66D* | Family With Sequence Similarity 66 Member D | [rs71237667](http://www.ncbi.nlm.nih.gov/projects/SNP/snp_ref.cgi?rs=rs71237667) | [8:11996252](genomebrowse://api/zoom?locus=8:11996252) | 0.0231629 | 0.0620562 | 0.0670412 | 0.062 | synonymous_variant | - | - | - |
| *FAM74A3* | Family With Sequence Similarity 74 Member A3 | [rs62566171](http://www.ncbi.nlm.nih.gov/projects/SNP/snp_ref.cgi?rs=rs62566171) | [9:40715926](genomebrowse://api/zoom?locus=9:40715926) | 0.134984 | - | - | - | non_coding_exon_variant | - | - | - |
| *FAM81B* | Family With Sequence Similarity 81 Member B | rs184777811 | 5:94759910 | 0.00439297 | 0.000732347 | 0.00393802 | 0.0009214 | intron_variant | - | - | - |
| *FAM81B* | Family With Sequence Similarity 81 Member B | [rs57231561](http://www.ncbi.nlm.nih.gov/projects/SNP/snp_ref.cgi?rs=rs57231561) | [5:94731761](genomebrowse://api/zoom?locus=5:94731761) | 0.0131789 | - | 0.0136557 | - | intron_variant | - | - | - |
| *FAM83H* | Family With Sequence Similarity 83 Member H | rs117987215 | 8:144808572 | 0.00299521 | 0.0061205 | 0.00541013 | 0.005901 | missense_variant | Tolerated | Benign | Tolerated |
| *FAM86B1* | Family With Sequence Similarity 86 Member B1 | [rs201429262](http://www.ncbi.nlm.nih.gov/projects/SNP/snp_ref.cgi?rs=rs201429262) | [8:12041157](genomebrowse://api/zoom?locus=8:12041157) | - | 0.051914 | 0.109158 | 0.016 | synonymous_variant | - | - | - |
| *FAM86B1* | Family With Sequence Similarity 86 Member B1 | [rs201223071](http://www.ncbi.nlm.nih.gov/projects/SNP/snp_ref.cgi?rs=rs201223071) | [8:12043950](genomebrowse://api/zoom?locus=8:12043950) | - | 0.0175994 | 0.0945274 | 0.02 | missense_variant | Tolerated | Benign | Tolerated |
| *FAM90A1* | Family With Sequence Similarity 90 Member A1 | rs117679735 | 12:8376723 | 0.00199681 | 0.00578804 | 0.00613497 | 0.006251 | missense_variant | Tolerated | Benign | Tolerated |
| *FAM90A27P* | Family With Sequence Similarity 90 Member A27, Pseudogene | [rs111531670](http://www.ncbi.nlm.nih.gov/projects/SNP/snp_ref.cgi?rs=rs111531670) | [19:53787375](genomebrowse://api/zoom?locus=19:53787375) | 0.123203 | 0.112016 | 0.103455 | 0.095 | non_coding_exon_variant | - | - | - |
| *FAM98B* | Family With Sequence Similarity 98 Member B | rs35671060 | 15:38746444 | 0.00199681 | 0.00487266 | 0.00536141 | 0.003848 | intron_variant | - | - | - |
| *FANCG* | FA Complementation Group G | [rs2237857](http://www.ncbi.nlm.nih.gov/projects/SNP/snp_ref.cgi?rs=rs2237857) | [9:35076755](genomebrowse://api/zoom?locus=9:35076755) | 0.0495208 | 0.0151257 | 0.0280597 | 0.018 | missense_variant | Tolerated | Benign | Tolerated |
| *FANCI* | FA Complementation Group I | [rs11857960](http://www.ncbi.nlm.nih.gov/projects/SNP/snp_ref.cgi?rs=rs11857960) | [15:89837139](genomebrowse://api/zoom?locus=15:89837139) | 0.0788738 | 0.0243242 | 0.0734144 | 0.028 | synonymous_variant | - | - | - |
| *FANCI* | FA Complementation Group I | [rs3087374](http://www.ncbi.nlm.nih.gov/projects/SNP/snp_ref.cgi?rs=rs3087374) | [15:89859994](genomebrowse://api/zoom?locus=15:89859994) | 0.0269569 | 0.0620257 | 0.0684578 | 0.061 | missense_variant | Damaging | Benign | Damaging |
| *FANCM* | FA Complementation Group M | rs7142192 | 14:45658415 | 0.00998403 | 0.00224485 | 0.00862459 | 0.002693 | synonymous_variant | - | - | - |
| *FANCM* | FA Complementation Group M | rs79343837 | 14:45665690 | 0.00998403 | 0.00215738 | 0.00807546 | 0.002627 | missense_variant | Tolerated | Benign | Tolerated |
| *FANCM* | FA Complementation Group M | [rs78410784](http://www.ncbi.nlm.nih.gov/projects/SNP/snp_ref.cgi?rs=rs78410784) | [14:45639985](genomebrowse://api/zoom?locus=14:45639985) | 0.0593051 | 0.0119976 | 0.0442584 | 0.014 | intron_variant | - | - | - |
| *FARP2* | FERM, ARH/RhoGEF And Pleckstrin Domain Protein 2 | [rs41342147](http://www.ncbi.nlm.nih.gov/projects/SNP/snp_ref.cgi?rs=rs41342147) | [2:242407588](genomebrowse://api/zoom?locus=2:242407588) | 0.0842652 | 0.105489 | 0.10234 | 0.103 | missense_variant | Tolerated | Probably damaging | Damaging |
| *FARS2* | Phenylalanyl-TRNA Synthetase 2, Mitochondrial | rs73718083 | 6:5369271 | 0.00439297 | 0.00100398 | 0.00368193 | 0.001277 | synonymous_variant | - | - | - |
| *FASN* | Fatty Acid Synthase | rs141141382 | 17:80039510 | 0.00159744 | 0.000911059 | 0.00103453 | 0.0008674 | missense_variant | Damaging | Possibly damaging | Tolerated |
| *FASTKD1* | FAST Kinase Domains 1 | [rs16857030](http://www.ncbi.nlm.nih.gov/projects/SNP/snp_ref.cgi?rs=rs16857030) | [2:170403106](genomebrowse://api/zoom?locus=2:170403106) | 0.0986422 | 0.0725283 | 0.105216 | 0.073 | synonymous_variant | - | - | - |
| *FASTKD5* | FAST Kinase Domains 5 | [rs3746698](http://www.ncbi.nlm.nih.gov/projects/SNP/snp_ref.cgi?rs=rs3746698) | [20:3128403](genomebrowse://api/zoom?locus=20:3128403) | 0.0241613 | 0.0132232 | 0.0104624 | 0.014 | synonymous_variant | - | - | - |
| *FAT1* | FAT Atypical Cadherin 1 | [rs114279527](http://www.ncbi.nlm.nih.gov/projects/SNP/snp_ref.cgi?rs=rs114279527) | [4:187628054](genomebrowse://api/zoom?locus=4:187628054) | 0.0241613 | 0.0342239 | 0.0266761 | 0.034 | synonymous_variant | - | - | - |
| *FAT3* | FAT Atypical Cadherin 3 | rs80074298 | 11:92616021 | 0.00698882 | 0.00176261 | 0.00669253 | 0.002265 | synonymous_variant | - | - | - |
| *FAT3* | FAT Atypical Cadherin 3 | rs181251382 | 11:92623068 | 0.00179712 | 0.00125837 | 0.00054906 | 0.001135 | intron_variant | - | - | - |
| *FAT3* | FAT Atypical Cadherin 3 | [rs75649640](http://www.ncbi.nlm.nih.gov/projects/SNP/snp_ref.cgi?rs=rs75649640) | [11:92600266](genomebrowse://api/zoom?locus=11:92600266) | 0.0656949 | 0.077138 | 0.064538 | 0.073 | synonymous_variant | - | - | - |
| *FAXDC2* | Fatty Acid Hydroxylase Domain Containing 2 | [rs62382170](http://www.ncbi.nlm.nih.gov/projects/SNP/snp_ref.cgi?rs=rs62382170) | [5:154214232](genomebrowse://api/zoom?locus=5:154214232) | 0.0145767 | 0.0349729 | 0.0345028 | 0.036 | missense_variant | Tolerated | Benign | Tolerated |
| *FBF1* | Fas Binding Factor 1 | [rs3826274](http://www.ncbi.nlm.nih.gov/projects/SNP/snp_ref.cgi?rs=rs3826274) | [17:73910395](genomebrowse://api/zoom?locus=17:73910395) | 0.110024 | 0.0983969 | 0.104427 | 0.088 | intron_variant | - | - | - |
| *FBLN1* | Fibulin 1 | [rs76711905](http://www.ncbi.nlm.nih.gov/projects/SNP/snp_ref.cgi?rs=rs76711905) | [22:45914541](genomebrowse://api/zoom?locus=22:45914541) | 0.0385383 | 0.0571909 | 0.0667184 | 0.05 | intron_variant | - | - | - |
| *FBLN1* | Fibulin 1 | [rs77035925](http://www.ncbi.nlm.nih.gov/projects/SNP/snp_ref.cgi?rs=rs77035925) | [22:45914692](genomebrowse://api/zoom?locus=22:45914692) | 0.0369409 | 0.057472 | 0.0663457 | 0.053 | intron_variant | - | - | - |
| *FBLN1* | Fibulin 1 | [rs2239844](http://www.ncbi.nlm.nih.gov/projects/SNP/snp_ref.cgi?rs=rs2239844) | [22:45927112](genomebrowse://api/zoom?locus=22:45927112) | 0.109026 | 0.107675 | 0.0872236 | 0.105 | intron_variant | - | - | - |
| *FBLN2* | Fibulin 2 | [rs41283996](http://www.ncbi.nlm.nih.gov/projects/SNP/snp_ref.cgi?rs=rs41283996) | [3:13670508](genomebrowse://api/zoom?locus=3:13670508) | 0.048123 | 0.0813742 | 0.0681332 | 0.08 | synonymous_variant | - | - | - |
| *FBN3* | Fibrillin 3 | [rs7258713](http://www.ncbi.nlm.nih.gov/projects/SNP/snp_ref.cgi?rs=rs7258713) | [19:8171068](genomebrowse://api/zoom?locus=19:8171068) | 0.0730831 | 0.0195559 | 0.0609654 | 0.023 | synonymous_variant | - | - | - |
| *FBN3* | Fibrillin 3 | [rs35840170](http://www.ncbi.nlm.nih.gov/projects/SNP/snp_ref.cgi?rs=rs35840170) | [19:8188812](genomebrowse://api/zoom?locus=19:8188812) | 0.0834665 | 0.0718464 | 0.0458481 | 0.064 | missense_variant | Tolerated | Benign | Tolerated |
| *FBRSL1* | Fibrosin Like 1 | [rs61529021](http://www.ncbi.nlm.nih.gov/projects/SNP/snp_ref.cgi?rs=rs61529021) | [12:133102403](genomebrowse://api/zoom?locus=12:133102403) | 0.0347444 | 0.0214295 | 0.0166203 | 0.02 | synonymous_variant | - | - | - |
| *FBXL13* | F-Box And Leucine Rich Repeat Protein 13 | [rs3800939](http://www.ncbi.nlm.nih.gov/projects/SNP/snp_ref.cgi?rs=rs3800939) | [7:102574715](genomebrowse://api/zoom?locus=7:102574715) | 0.0678914 | 0.0634837 | 0.0463118 | 0.066 | missense_variant | Tolerated | Possibly damaging | Damaging |
| *FBXL18* | F-Box And Leucine Rich Repeat Protein 18 | [rs11975313](http://www.ncbi.nlm.nih.gov/projects/SNP/snp_ref.cgi?rs=rs11975313) | [7:5530954](genomebrowse://api/zoom?locus=7:5530954) | 0.179712 | 0.15835 | 0.173382 | 0.154 | synonymous_variant | - | - | Damaging |
| *FBXL21* | F-Box And Leucine Rich Repeat Protein 21 | rs148275750 | 5:135272498 | 0.000399361 | 8.12757e-05 | 0.000323164 | 0.0001159 | stop_gained | - | - | Damaging |
| *FBXL21* | F-Box And Leucine Rich Repeat Protein 21 | rs79738762 | 5:135277062 | 0.00958466 | 0.00192209 | 0.00804056 | 0.002573 | missense_variant | - | Benign | Damaging |
| *FBXL4* | F-Box And Leucine Rich Repeat Protein 4 | rs61744041 | 6:99353427 | 0.00439297 | 0.00555429 | 0.00461707 | 0.005691 | synonymous_variant | - | - | - |
| *FBXL4* | F-Box And Leucine Rich Repeat Protein 4 | [rs34316889](http://www.ncbi.nlm.nih.gov/projects/SNP/snp_ref.cgi?rs=rs34316889) | [6:99374760](genomebrowse://api/zoom?locus=6:99374760) | 0.0133786 | 0.0218939 | 0.0211791 | 0.021 | missense_variant | Tolerated | Benign | Tolerated |
| *FBXL6* | F-Box And Leucine Rich Repeat Protein 6 | rs139662377 | 8:145579785 | 0.000399361 | 0.00016269 | 0.000614489 | 0.0001977 | missense_variant | Tolerated | Benign | Damaging |
| *FBXL7* | F-Box And Leucine Rich Repeat Protein 7 | rs35368404 | 5:15928420 | 0.00758786 | 0.00182383 | 0.00739282 | 0.002089 | synonymous_variant | - | - | - |
| *FBXO16* | F-Box Protein 16 | rs199665828 | 8:28314368 | 0.000199681 | 5.68597e-05 | 6.45745e-05 | 4.942e-05 | missense_variant | Tolerated | Benign | Tolerated |
| *FBXO2* | F-Box Protein 2 | rs141266599 | 1:11708856 | 0.000599042 | 0.00234077 | 0.00190827 | 0.002331 | synonymous_variant | - | - | - |
| *FBXO38* | F-Box Protein 38 | [rs74863106](http://www.ncbi.nlm.nih.gov/projects/SNP/snp_ref.cgi?rs=rs74863106) | [5:147778631](genomebrowse://api/zoom?locus=5:147778631) | 0.0375399 | 0.0274035 | 0.0127998 | 0.024 | synonymous_variant | - | - | - |
| *FBXO39* | F-Box Protein 39 | [rs10401001](http://www.ncbi.nlm.nih.gov/projects/SNP/snp_ref.cgi?rs=rs10401001) | [17:6683871](genomebrowse://api/zoom?locus=17:6683871) | 0.0706869 | 0.0185173 | 0.0618613 | 0.023 | synonymous_variant | - | - | - |
| *FBXO44* | F-Box Protein 44 | rs150816569 | 1:11718859 | 0.00479233 | 0.00514436 | 0.00458805 | 0.004588 | missense_variant | Tolerated | Benign | Tolerated |
| *FBXW10* | F-Box And WD Repeat Domain Containing 10 | rs117585894 | 17:18673345 | 0.000798722 | 0.00152273 | 0.000968679 | 0.001425 | synonymous_variant | - | - | - |
| *FBXW10* | F-Box And WD Repeat Domain Containing 10 | [rs200711350](http://www.ncbi.nlm.nih.gov/projects/SNP/snp_ref.cgi?rs=rs200711350) | [17:18653276](genomebrowse://api/zoom?locus=17:18653276) | 0.0579073 | 0.0689126 | 0.0551115 | 0.069 | intron_variant | - | - | - |
| *FCGBP* | Fc Fragment Of IgG Binding Protein | [rs150027077](http://www.ncbi.nlm.nih.gov/projects/SNP/snp_ref.cgi?rs=rs150027077) | [19:40368619](genomebrowse://api/zoom?locus=19:40368619) | - | 0.107295 | 0.0832883 | 0.179 | synonymous_variant | - | - | - |
| *FCGBP* | Fc Fragment Of IgG Binding Protein | [rs368994804](http://www.ncbi.nlm.nih.gov/projects/SNP/snp_ref.cgi?rs=rs368994804) | [19:40368697](genomebrowse://api/zoom?locus=19:40368697) | - | 0.0168241 | 0.0345658 | 0.06 | synonymous_variant | - | - | - |
| *FCGBP* | Fc Fragment Of IgG Binding Protein | [rs775561740](http://www.ncbi.nlm.nih.gov/projects/SNP/snp_ref.cgi?rs=rs775561740) | [19:40368733](genomebrowse://api/zoom?locus=19:40368733) | - | 0.0320968 | 0.0854105 | 0.052 | synonymous_variant | - | - | - |
| *FCGR3B* | Fc Fragment Of IgG Receptor IIIb | [rs114169903](http://www.ncbi.nlm.nih.gov/projects/SNP/snp_ref.cgi?rs=rs114169903) | [1:161596032](genomebrowse://api/zoom?locus=1:161596032) | 0.0247604 | 0.0172106 | 0.0366118 | 0.019 | synonymous_variant | - | - | - |
| *FCGR3B* | Fc Fragment Of IgG Receptor IIIb | [rs5030738](http://www.ncbi.nlm.nih.gov/projects/SNP/snp_ref.cgi?rs=rs5030738) | [1:161599654](genomebrowse://api/zoom?locus=1:161599654) | 0.0828674 | 0.0375163 | 0.0857066 | 0.042 | missense_variant | Tolerated | Benign | Tolerated |
| *FCGR3B* | Fc Fragment Of IgG Receptor IIIb | [rs111402007](http://www.ncbi.nlm.nih.gov/projects/SNP/snp_ref.cgi?rs=rs111402007) | [1:161600189](genomebrowse://api/zoom?locus=1:161600189) | 0.0225639 | 0.0434682 | 0.0354175 | 0.044 | intron_variant | - | - | - |
| *FCHSD1* | FCH And Double SH3 Domains 1 | rs140791780 | 5:141023864 | 0.00858626 | 0.00201789 | 0.0084477 | 0.002367 | missense_variant | Tolerated | Benign | Tolerated |
| *FCN1* | Ficolin 1 | rs141017404 | 9:137804383 | 0.000599042 | 0.000544419 | 0.000258916 | 0.0004695 | synonymous_variant | - | - | - |
| *FCRL3* | Fc Receptor Like 3 | [rs2282284](http://www.ncbi.nlm.nih.gov/projects/SNP/snp_ref.cgi?rs=rs2282284) | [1:157648543](genomebrowse://api/zoom?locus=1:157648543) | 0.0573083 | 0.054591 | 0.0575419 | 0.056 | missense_variant | Tolerated | Benign | Tolerated |
| *FCRL4* | Fc Receptor Like 4 | rs78796764 | 1:157559076 | 0.00439297 | 0.00298917 | 0.00213123 | 0.003015 | synonymous_variant | - | - | - |
| *FCRL5* | Fc Receptor Like 5 | [rs73011567](http://www.ncbi.nlm.nih.gov/projects/SNP/snp_ref.cgi?rs=rs73011567) | [1:157504422](genomebrowse://api/zoom?locus=1:157504422) | 0.1252 | 0.0390746 | 0.0877352 | 0.045 | missense_variant | Damaging | Possibly damaging | Tolerated |
| *FCRL5* | Fc Receptor Like 5 | [rs73011568](http://www.ncbi.nlm.nih.gov/projects/SNP/snp_ref.cgi?rs=rs73011568) | [1:157504433](genomebrowse://api/zoom?locus=1:157504433) | 0.125399 | 0.0391183 | 0.0877981 | 0.045 | missense_variant | Damaging | Probably damaging | Tolerated |
| *FCRLA* | Fc Receptor Like A | [rs61801161](http://www.ncbi.nlm.nih.gov/projects/SNP/snp_ref.cgi?rs=rs61801161) | [1:161680518](genomebrowse://api/zoom?locus=1:161680518) | 0.0489217 | 0.0508177 | 0.0567983 | 0.053 | intron_variant | - | - | - |
| *FDFT1* | Farnesyl-Diphosphate Farnesyltransferase 1 | [rs9205](http://www.ncbi.nlm.nih.gov/projects/SNP/snp_ref.cgi?rs=rs9205) | [8:11689119](genomebrowse://api/zoom?locus=8:11689119) | 0.306909 | 0.278498 | 0.229199 | 0.275 | synonymous_variant | - | - | - |
| *FDXR* | Ferredoxin Reductase | [rs2070921](http://www.ncbi.nlm.nih.gov/projects/SNP/snp_ref.cgi?rs=rs2070921) | [17:72860654](genomebrowse://api/zoom?locus=17:72860654) | 0.0553115 | 0.0354951 | 0.0160026 | 0.033 | synonymous_variant | - | - | - |
| *FERD3L* | Fer3 Like BHLH Transcription Factor | rs149352998 | 7:19184920 | 0.000798722 | 0.000205643 | 0.000710365 | 0.0002471 | synonymous_variant | - | - | - |
| *FERMT1* | Fermitin Family Member 1 | [rs6076938](http://www.ncbi.nlm.nih.gov/projects/SNP/snp_ref.cgi?rs=rs6076938) | [20:6077726](genomebrowse://api/zoom?locus=20:6077726) | 0.234625 | 0.273776 | 0.216712 | 0.317 | intron_variant | - | - | - |
| *FETUB* | Fetuin B | [rs1047115](http://www.ncbi.nlm.nih.gov/projects/SNP/snp_ref.cgi?rs=rs1047115) | [3:186358366](genomebrowse://api/zoom?locus=3:186358366) | 0.115815 | 0.110819 | 0.135036 | 0.114 | synonymous_variant | - | - | - |
| *FFAR3* | Free Fatty Acid Receptor 3 | [rs146211359](http://www.ncbi.nlm.nih.gov/projects/SNP/snp_ref.cgi?rs=rs146211359) | [19:35850416](genomebrowse://api/zoom?locus=19:35850416) | - | 0.0286665 | 0.0192158 | 0.029 | synonymous_variant | - | - | - |
| *FGF18* | Fibroblast Growth Factor 18 | [rs34347344](http://www.ncbi.nlm.nih.gov/projects/SNP/snp_ref.cgi?rs=rs34347344) | [5:170883734](genomebrowse://api/zoom?locus=5:170883734) | 0.0557109 | 0.061318 | 0.034944 | 0.056 | synonymous_variant | - | - | - |
| *FH* | Fumarate Hydratase | [rs61737760](http://www.ncbi.nlm.nih.gov/projects/SNP/snp_ref.cgi?rs=rs61737760) | [1:241667523](genomebrowse://api/zoom?locus=1:241667523) | 0.0375399 | 0.0347332 | 0.026547 | 0.035 | synonymous_variant | - | - | - |
| *FHAD1* | Forkhead Associated Phosphopeptide Binding Domain 1 | rs200196291 | 1:15623268 | 0.000798722 | 0.0046883 | 0.00513367 | 0.002866 | synonymous_variant | - | - | - |
| *FHAD1* | Forkhead Associated Phosphopeptide Binding Domain 1 | rs144834032 | 1:15679403 | 0.000798722 | 0.000363785 | 0.00206972 | 0.0003826 | missense_variant | Damaging | Benign | Tolerated |
| *FHAD1* | Forkhead Associated Phosphopeptide Binding Domain 1 | [rs113966492](http://www.ncbi.nlm.nih.gov/projects/SNP/snp_ref.cgi?rs=rs113966492) | [1:15707775](genomebrowse://api/zoom?locus=1:15707775) | 0.0121805 | 0.0262904 | 0.0195502 | 0.017 | missense_variant | Tolerated | Probably damaging | Tolerated |
| *FHAD1* | Forkhead Associated Phosphopeptide Binding Domain 1 | [rs6685602](http://www.ncbi.nlm.nih.gov/projects/SNP/snp_ref.cgi?rs=rs6685602) | [1:15709641](genomebrowse://api/zoom?locus=1:15709641) | 0.0335463 | 0.031092 | 0.0417987 | 0.023 | intron_variant | - | - | - |
| *FHIT* | Fragile Histidine Triad Diadenosine Triphosphatase | rs73842465 | 3:59999799 | 0.00998403 | 0.00252815 | 0.00884899 | 0.003179 | synonymous_variant | - | - | - |
| *FHOD3* | Formin Homology 2 Domain Containing 3 | rs150630527 | 18:34273358 | 0.00179712 | 0.00333363 | 0.00255151 | 0.003295 | synonymous_variant | - | - | - |
| *FHOD3* | Formin Homology 2 Domain Containing 3 | [rs9304162](http://www.ncbi.nlm.nih.gov/projects/SNP/snp_ref.cgi?rs=rs9304162) | [18:34320630](genomebrowse://api/zoom?locus=18:34320630) | 0.0654952 | 0.0161658 | 0.0610615 | 0.021 | synonymous_variant | - | - | - |
| *FJX1* | Four-Jointed Box Kinase 1 | [rs12286850](http://www.ncbi.nlm.nih.gov/projects/SNP/snp_ref.cgi?rs=rs12286850) | [11:35641428](genomebrowse://api/zoom?locus=11:35641428) | 0.0714856 | 0.0141759 | 0.0655674 | 0.015 | missense_variant | Tolerated | Benign | Tolerated |
| *FKBP10* | FKBP Prolyl Isomerase 10 | [rs34764749](http://www.ncbi.nlm.nih.gov/projects/SNP/snp_ref.cgi?rs=rs34764749) | [17:39974642](genomebrowse://api/zoom?locus=17:39974642) | 0.0742811 | 0.0478513 | 0.0712207 | 0.049 | missense_variant | Tolerated | Benign | Tolerated |
| *FKBP15* | FKBP Prolyl Isomerase 15 | [rs45559933](http://www.ncbi.nlm.nih.gov/projects/SNP/snp_ref.cgi?rs=rs45559933) | [9:115950114](genomebrowse://api/zoom?locus=9:115950114) | 0.0119808 | 0.0303469 | 0.0358066 | 0.03 | missense_variant | Damaging | Possibly damaging | Tolerated |
| *FLCN* | Folliculin | [rs61750032](http://www.ncbi.nlm.nih.gov/projects/SNP/snp_ref.cgi?rs=rs61750032) | [17:17119761](genomebrowse://api/zoom?locus=17:17119761) | 0.0329473 | 0.0101713 | 0.0313936 | 0.012 | synonymous_variant | - | - | - |
| *FLCN* | Folliculin | [rs3744123](http://www.ncbi.nlm.nih.gov/projects/SNP/snp_ref.cgi?rs=rs3744123) | [17:17127470](genomebrowse://api/zoom?locus=17:17127470) | 0.0652955 | 0.0323189 | 0.0620638 | 0.03 | intron_variant | - | - | - |
| *FLG* | Filaggrin | rs76330665 | 1:152275273 | 0.0091853 | 0.00242622 | 0.00888071 | 0.003171 | missense_variant | Tolerated | Benign | Tolerated |
| *FLG* | Filaggrin | [rs7518080](http://www.ncbi.nlm.nih.gov/projects/SNP/snp_ref.cgi?rs=rs7518080) | [1:152276671](genomebrowse://api/zoom?locus=1:152276671) | 0.0163738 | 0.0434257 | 0.0426025 | 0.033 | missense_variant | Damaging | Possibly damaging | Tolerated |
| *FLG* | Filaggrin | [rs34806697](http://www.ncbi.nlm.nih.gov/projects/SNP/snp_ref.cgi?rs=rs34806697) | [1:152281621](genomebrowse://api/zoom?locus=1:152281621) | 0.0369409 | 0.0678601 | 0.0685737 | 0.067 | missense_variant | Tolerated | Benign | Tolerated |
| *FLII* | FLII Actin Remodeling Protein | [rs113208618](http://www.ncbi.nlm.nih.gov/projects/SNP/snp_ref.cgi?rs=rs113208618) | [17:18148944](genomebrowse://api/zoom?locus=17:18148944) | 0.0417332 | 0.0117768 | 0.0354326 | 0.014 | synonymous_variant | - | - | - |
| *FLJ10038* | - | [rs8552](http://www.ncbi.nlm.nih.gov/projects/SNP/snp_ref.cgi?rs=rs8552) | [15:50646783](genomebrowse://api/zoom?locus=15:50646783) | 0.113019 | 0.0583109 | 0.0746877 | 0.032 | intron_variant | - | - | - |
| *FLJ23867* | - | rs192185246 | 1:180167380 | 0.00279553 | 0.00576842 | 0.0061292 | 0.004474 | non_coding_exon_variant | - | - | - |
| *FLJ43681* | - | rs544696302 | 17:81188023 | 0.000998403 | 0.0027672 | 0.00759878 | 0.003046 | non_coding_exon_variant | - | - | - |
| *FLJ44313* | - | rs561387578 | 18:74208371 | 0.00459265 | 0.000704674 | 0.00410472 | 0.000437 | missense_variant | - | Benign | Tolerated |
| *FLNC* | Filamin C | rs34932223 | 7:128480629 | 0.00299521 | 0.00231893 | 0.00122834 | 0.002319 | missense_variant | Damaging | Benign | Tolerated |
| *FLNC* | Filamin C | [rs76046880](http://www.ncbi.nlm.nih.gov/projects/SNP/snp_ref.cgi?rs=rs76046880) | [7:128480666](genomebrowse://api/zoom?locus=7:128480666) | 0.0159744 | 0.0314081 | 0.0343033 | 0.031 | synonymous_variant | - | - | - |
| *FLNC* | Filamin C | [rs111958241](http://www.ncbi.nlm.nih.gov/projects/SNP/snp_ref.cgi?rs=rs111958241) | [7:128482806](genomebrowse://api/zoom?locus=7:128482806) | 0.0147764 | 0.0313871 | 0.0331001 | 0.031 | intron_variant | - | - | - |
| *FLNC* | Filamin C | [rs78086167](http://www.ncbi.nlm.nih.gov/projects/SNP/snp_ref.cgi?rs=rs78086167) | [7:128482835](genomebrowse://api/zoom?locus=7:128482835) | 0.0147764 | 0.0314039 | 0.0332643 | 0.031 | intron_variant | - | - | - |
| *FLNC* | Filamin C | [rs34180031](http://www.ncbi.nlm.nih.gov/projects/SNP/snp_ref.cgi?rs=rs34180031) | [7:128486091](genomebrowse://api/zoom?locus=7:128486091) | 0.0197684 | 0.0326152 | 0.0390939 | 0.033 | synonymous_variant | - | - | - |
| *FLNC* | Filamin C | [rs74811088](http://www.ncbi.nlm.nih.gov/projects/SNP/snp_ref.cgi?rs=rs74811088) | [7:128488800](genomebrowse://api/zoom?locus=7:128488800) | 0.0147764 | 0.0313163 | 0.0331672 | 0.031 | intron_variant | - | - | - |
| *FLNC* | Filamin C | [rs79790270](http://www.ncbi.nlm.nih.gov/projects/SNP/snp_ref.cgi?rs=rs79790270) | [7:128491497](genomebrowse://api/zoom?locus=7:128491497) | 0.0147764 | 0.0313582 | 0.0332277 | 0.031 | intron_variant | - | - | - |
| *FLRT1* | Fibronectin Leucine Rich Transmembrane Protein 1 | [rs947939](http://www.ncbi.nlm.nih.gov/projects/SNP/snp_ref.cgi?rs=rs947939) | [11:63885287](genomebrowse://api/zoom?locus=11:63885287) | 0.0692891 | 0.112361 | 0.103543 | 0.112 | synonymous_variant | - | - | - |
| *FLT1* | Fms Related Receptor Tyrosine Kinase 1 | rs61763178 | 13:29001430 | 0.00738818 | 0.00166212 | 0.00597622 | 0.002125 | synonymous_variant | - | - | - |
| *FLT1* | Fms Related Receptor Tyrosine Kinase 1 | [rs56314249](http://www.ncbi.nlm.nih.gov/projects/SNP/snp_ref.cgi?rs=rs56314249) | [13:28896979](genomebrowse://api/zoom?locus=13:28896979) | 0.0447284 | 0.0582154 | 0.0264738 | 0.05 | synonymous_variant | - | - | - |
| *FLT4* | Fms Related Receptor Tyrosine Kinase 4 | [rs3736062](http://www.ncbi.nlm.nih.gov/projects/SNP/snp_ref.cgi?rs=rs3736062) | [5:180052946](genomebrowse://api/zoom?locus=5:180052946) | 0.0341454 | 0.0313836 | 0.0242766 | 0.03 | synonymous_variant | - | - | - |
| *FLT4* | Fms Related Receptor Tyrosine Kinase 4 | [rs56188706](http://www.ncbi.nlm.nih.gov/projects/SNP/snp_ref.cgi?rs=rs56188706) | [5:180057356](genomebrowse://api/zoom?locus=5:180057356) | 0.171725 | 0.129881 | 0.134841 | 0.128 | intron_variant | - | - | - |
| *FLVCR1* | FLVCR Heme Transporter 1 | [rs17677416](http://www.ncbi.nlm.nih.gov/projects/SNP/snp_ref.cgi?rs=rs17677416) | [1:213061308](genomebrowse://api/zoom?locus=1:213061308) | 0.0119808 | 0.0276863 | 0.0256733 | 0.027 | synonymous_variant | - | - | - |
| *FMN2* | Formin 2 | [rs6677726](http://www.ncbi.nlm.nih.gov/projects/SNP/snp_ref.cgi?rs=rs6677726) | [1:240492414](genomebrowse://api/zoom?locus=1:240492414) | 0.15635 | 0.115984 | 0.1076 | 0.112 | synonymous_variant | - | - | - |
| *FMNL1* | Formin Like 1 | rs75160160 | 17:43307953 | 0.00579073 | 0.00571219 | 0.00132686 | 0.004967 | intron_variant | - | - | - |
| *FMNL1* | Formin Like 1 | [rs9898221](http://www.ncbi.nlm.nih.gov/projects/SNP/snp_ref.cgi?rs=rs9898221) | [17:43311620](genomebrowse://api/zoom?locus=17:43311620) | 0.0692891 | 0.0655107 | 0.0663645 | 0.062 | intron_variant | - | - | - |
| *FMNL2* | Formin Like 2 | [rs35776654](http://www.ncbi.nlm.nih.gov/projects/SNP/snp_ref.cgi?rs=rs35776654) | [2:153468107](genomebrowse://api/zoom?locus=2:153468107) | 0.0519169 | 0.105196 | 0.116708 | 0.1 | synonymous_variant | - | - | - |
| *FMO3* | Flavin Containing Dimethylaniline Monoxygenase 3 | rs151271991 | 1:171070298 | 0.00139776 | 0.000259176 | 0.00132369 | 6.932e-05 | intron_variant | - | - | - |
| *FMR1* | FMRP Translational Regulator 1 | [rs25714](http://www.ncbi.nlm.nih.gov/projects/SNP/snp_ref.cgi?rs=rs25714) | [X:147018146](genomebrowse://api/zoom?locus=X:147018146) | 0.261192 | 0.158525 | 0.121127 | 0.153 | intron_variant | - | - | - |
| *FN1* | Fibronectin 1 | rs151174151 | 2:216229671 | 0.00299521 | 0.00265949 | 0.00219624 | 0.002038 | synonymous_variant | - | - | - |
| *FN1* | Fibronectin 1 | rs139078629 | 2:216251538 | 0.00279553 | 0.00474386 | 0.00504952 | 0.004892 | missense_variant | Damaging | Probably damaging | Damaging |
| *FN1* | Fibronectin 1 | rs138144264 | 2:216285445 | 0.000199681 | 0.00104806 | 0.000838926 | 0.001211 | synonymous_variant | - | - | - |
| *FN1* | Fibronectin 1 | [rs10498037](http://www.ncbi.nlm.nih.gov/projects/SNP/snp_ref.cgi?rs=rs10498037) | [2:216230216](genomebrowse://api/zoom?locus=2:216230216) | 0.0535144 | 0.0734998 | 0.0744571 | 0.071 | intron_variant | - | - | - |
| *FN1* | Fibronectin 1 | [rs17458018](http://www.ncbi.nlm.nih.gov/projects/SNP/snp_ref.cgi?rs=rs17458018) | [2:216285375](genomebrowse://api/zoom?locus=2:216285375) | 0.0453275 | 0.0598391 | 0.0365058 | 0.06 | intron_variant | - | - | - |
| *FN1* | Fibronectin 1 | [rs10648691](http://www.ncbi.nlm.nih.gov/projects/SNP/snp_ref.cgi?rs=rs10648691) | [2:216299572](genomebrowse://api/zoom?locus=2:216299572) | 0.56889 | 0.511916 | 0.524257 | 0.504 | intron_variant | - | - | - |
| *FN3K* | Fructosamine 3 Kinase | [rs72318398](http://www.ncbi.nlm.nih.gov/projects/SNP/snp_ref.cgi?rs=rs72318398) | [17:80706720](genomebrowse://api/zoom?locus=17:80706720) | 0.184105 | 0.151158 | 0.153109 | 0.16 | intron_variant | - | - | - |
| *FNBP1L* | Formin Binding Protein 1 Like | [rs237426](http://www.ncbi.nlm.nih.gov/projects/SNP/snp_ref.cgi?rs=rs237426) | [1:94012379](genomebrowse://api/zoom?locus=1:94012379) | 0.0449281 | 0.0109098 | 0.0380361 | 0.013 | intron_variant | - | - | - |
| *FNDC3B* | Fibronectin Type III Domain Containing 3B | rs191259545 | 3:172034254 | 0.00179712 | 0.000584264 | 0.00371928 | 0.0001481 | intron_variant | - | - | - |
| *FNDC7* | Fibronectin Type III Domain Containing 7 | [rs17553619](http://www.ncbi.nlm.nih.gov/projects/SNP/snp_ref.cgi?rs=rs17553619) | [1:109261509](genomebrowse://api/zoom?locus=1:109261509) | 0.0491214 | 0.0640556 | 0.0512721 | 0.075 | missense_variant | Tolerated | Benign | Tolerated |
| *FOCAD* | Focadhesin | [rs60488560](http://www.ncbi.nlm.nih.gov/projects/SNP/snp_ref.cgi?rs=rs60488560) | [9:20916865](genomebrowse://api/zoom?locus=9:20916865) | 0.0223642 | 0.0233889 | 0.0241034 | 0.024 | intron_variant | - | - | - |
| *FOLR3* | Folate Receptor Gamma | [rs1802608](http://www.ncbi.nlm.nih.gov/projects/SNP/snp_ref.cgi?rs=rs1802608) | [11:71850722](genomebrowse://api/zoom?locus=11:71850722) | 0.0826677 | 0.0620049 | 0.105234 | 0.065 | missense_variant | - | - | - |
| *FOXA3* | Forkhead Box A3 | [rs3810327](http://www.ncbi.nlm.nih.gov/projects/SNP/snp_ref.cgi?rs=rs3810327) | [19:46376217](genomebrowse://api/zoom?locus=19:46376217) | 0.0696885 | 0.0902441 | 0.0880088 | 0.091 | synonymous_variant | - | - | - |
| *FOXI1* | Forkhead Box I1 | rs35678180 | 5:169535204 | 0.00139776 | 0.00530796 | 0.00481577 | 0.005329 | synonymous_variant | - | - | - |
| *FOXK1* | Forkhead Box K1 | [rs79357656](http://www.ncbi.nlm.nih.gov/projects/SNP/snp_ref.cgi?rs=rs79357656) | [7:4799141](genomebrowse://api/zoom?locus=7:4799141) | 0.0199681 | 0.0374956 | 0.0392931 | 0.031 | synonymous_variant | - | - | - |
| *FPGT-TNNI3K* | FPGT-TNNI3K Readthrough | rs547775196 | 1:74946404 | 0.000399361 | 2.09861e-05 | 9.69117e-05 | 4.679e-05 | intron_variant | Tolerated | - | Damaging |
| *FREM1* | FRAS1 Related Extracellular Matrix 1 | [rs16932323](http://www.ncbi.nlm.nih.gov/projects/SNP/snp_ref.cgi?rs=rs16932323) | [9:14823218](genomebrowse://api/zoom?locus=9:14823218) | 0.0455272 | 0.0102749 | 0.0396472 | 0.012 | synonymous_variant | - | - | - |
| *FREM1* | FRAS1 Related Extracellular Matrix 1 | [rs41313784](http://www.ncbi.nlm.nih.gov/projects/SNP/snp_ref.cgi?rs=rs41313784) | [9:14851594](genomebrowse://api/zoom?locus=9:14851594) | 0.038139 | 0.013924 | 0.0233467 | 0.014 | synonymous_variant | - | - | - |
| *FREM3* | FRAS1 Related Extracellular Matrix 3 | rs144048977 | 4:144620134 | 0.000998403 | 0.00341282 | 0.00278011 | 0.001318 | synonymous_variant | - | - | - |
| *FREM3* | FRAS1 Related Extracellular Matrix 3 | [rs72940301](http://www.ncbi.nlm.nih.gov/projects/SNP/snp_ref.cgi?rs=rs72940301) | [4:144619921](genomebrowse://api/zoom?locus=4:144619921) | 0.102037 | 0.0406819 | 0.0689666 | 0.026 | synonymous_variant | - | - | - |
| *FRG2B* | FSHD Region Gene 2 Family Member B | [rs200661929](http://www.ncbi.nlm.nih.gov/projects/SNP/snp_ref.cgi?rs=rs200661929) | [10:135440203](genomebrowse://api/zoom?locus=10:135440203) | 0.173123 | 0.0875782 | 0.103539 | 0.105 | missense_variant | Damaging | Benign | Tolerated |
| *FRMD1* | FERM Domain Containing 1 | rs73262843 | 6:168461534 | 0.0081869 | 0.00185839 | 0.0070459 | 0.002265 | missense_variant | Damaging | Benign | Tolerated |
| *FRMD1* | FERM Domain Containing 1 | [rs902393](http://www.ncbi.nlm.nih.gov/projects/SNP/snp_ref.cgi?rs=rs902393) | [6:168463624](genomebrowse://api/zoom?locus=6:168463624) | 0.0696885 | 0.0709963 | 0.0732387 | 0.073 | missense_variant | Damaging | Benign | Tolerated |
| *FRMD7* | FERM Domain Containing 7 | [rs5930546](http://www.ncbi.nlm.nih.gov/projects/SNP/snp_ref.cgi?rs=rs5930546) | [X:131234733](genomebrowse://api/zoom?locus=X:131234733) | 0.0569536 | 0.0844724 | 0.0868254 | 0.087 | synonymous_variant | - | - | - |
| *FRMPD1* | FERM And PDZ Domain Containing 1 | rs150491654 | 9:37744678 | 0.000399361 | 0.00147524 | 0.002553 | 0.001359 | synonymous_variant | - | - | - |
| *FRMPD1* | FERM And PDZ Domain Containing 1 | [rs2274324](http://www.ncbi.nlm.nih.gov/projects/SNP/snp_ref.cgi?rs=rs2274324) | [9:37731007](genomebrowse://api/zoom?locus=9:37731007) | 0.0880591 | 0.0484686 | 0.0422754 | 0.049 | synonymous_variant | - | - | - |
| *FRRS1* | Ferric Chelate Reductase 1 | rs143957192 | 1:100206478 | 0.00159744 | 0.00321239 | 0.00242436 | 0.003772 | missense_variant | Tolerated | Benign | Damaging |
| *FRRS1* | Ferric Chelate Reductase 1 | [rs41285734](http://www.ncbi.nlm.nih.gov/projects/SNP/snp_ref.cgi?rs=rs41285734) | [1:100207818](genomebrowse://api/zoom?locus=1:100207818) | 0.0607029 | 0.0438002 | 0.0316194 | 0.047 | synonymous_variant | - | - | - |
| *FRY* | FRY Microtubule Binding Protein | rs202082890 | 13:32735264 | 0.000399361 | 0.000134229 | 0.000193711 | 9.934e-05 | intron_variant | - | - | - |
| *FRY* | FRY Microtubule Binding Protein | [rs12428144](http://www.ncbi.nlm.nih.gov/projects/SNP/snp_ref.cgi?rs=rs12428144) | [13:32676096](genomebrowse://api/zoom?locus=13:32676096) | 0.0952476 | 0.0661537 | 0.0438273 | 0.064 | splice_region_variant | - | - | - |
| *FRY* | FRY Microtubule Binding Protein | [rs2301386](http://www.ncbi.nlm.nih.gov/projects/SNP/snp_ref.cgi?rs=rs2301386) | [13:32868498](genomebrowse://api/zoom?locus=13:32868498) | 0.0660942 | 0.0568203 | 0.0335185 | 0.055 | splice_region_variant | - | - | - |
| *FRYL* | FRY Like Transcription Coactivator | rs146633085 | 4:48517232 | 0.00399361 | 0.000762071 | 0.00261425 | 0.0009603 | missense_variant | Tolerated | Benign | Damaging |
| *FSCN3* | Fascin Actin-Bundling Protein 3 | [rs3779536](http://www.ncbi.nlm.nih.gov/projects/SNP/snp_ref.cgi?rs=rs3779536) | [7:127233977](genomebrowse://api/zoom?locus=7:127233977) | 0.110623 | 0.0596436 | 0.0864142 | 0.061 | missense_variant | Tolerated | Probably damaging | Damaging |
| *FSIP2* | Fibrous Sheath Interacting Protein 2 | [rs17826666](http://www.ncbi.nlm.nih.gov/projects/SNP/snp_ref.cgi?rs=rs17826666) | [2:186678633](genomebrowse://api/zoom?locus=2:186678633) | 0.0529153 | 0.0729326 | 0.0596503 | 0.075 | missense_variant | Tolerated | - | Tolerated |
| *FSTL4* | Follistatin Like 4 | rs141195594 | 5:132535129 | 0.000599042 | 0.000446715 | 0.00051703 | 0.0003542 | synonymous_variant | - | - | - |
| *FSTL5* | Follistatin Like 5 | [rs3749598](http://www.ncbi.nlm.nih.gov/projects/SNP/snp_ref.cgi?rs=rs3749598) | [4:162307312](genomebrowse://api/zoom?locus=4:162307312) | 0.0910543 | 0.112024 | 0.113151 | 0.109 | missense_variant | Damaging | Probably damaging | Damaging |
| *FTX* | FTX Transcript, XIST Regulator | rs372055341 | X:73438224 | 0.00264901 | 0.00582645 | 0.00645341 | 0.005244 | intron_variant | - | - | - |
| *FUK* | Fucose Kinase | [rs17880268](http://www.ncbi.nlm.nih.gov/projects/SNP/snp_ref.cgi?rs=rs17880268) | [16:70500070](genomebrowse://api/zoom?locus=16:70500070) | 0.0840655 | 0.0175511 | 0.0720939 | 0.021 | synonymous_variant | - | - | - |
| *FURIN* | Furin, Paired Basic Amino Acid Cleaving Enzyme | c.1729G>A | [15:91424207](genomebrowse://api/zoom?locus=15:91424207) | - | - | - | - | missense_variant | Tolerated | Benign | Damaging |
| *FUS* | FUS RNA Binding Protein | rs140875749 | 16:31202973 | 0.00279553 | 0.00383401 | 0.00258582 | 0.001434 | 3_prime_UTR_variant | - | - | - |
| *FUS* | FUS RNA Binding Protein | rs184109677 | 16:31206057 | 0.00399361 | 0.000796062 | 0.00329351 | 0.0001482 | 3_prime_UTR_variant | - | - | - |
| *FUS* | FUS RNA Binding Protein | c.1542-21delT | [16:31202699](genomebrowse://api/zoom?locus=16:31202699) | - | 0.334737 | 0.0146112 | 0.328 | intron_variant | - | - | - |
| *FUS* | FUS RNA Binding Protein | [rs118018900](http://www.ncbi.nlm.nih.gov/projects/SNP/snp_ref.cgi?rs=rs118018900) | [16:31203669](genomebrowse://api/zoom?locus=16:31203669) | 0.0159744 | 0.0339202 | 0.0251664 | 0.036 | 3_prime_UTR_variant | - | - | - |
| *FUT1* | Fucosyltransferase 1 (H Blood Group) | [rs2071699](http://www.ncbi.nlm.nih.gov/projects/SNP/snp_ref.cgi?rs=rs2071699) | [19:49254504](genomebrowse://api/zoom?locus=19:49254504) | 0.0998403 | 0.0587204 | 0.0356427 | 0.057 | missense_variant | Tolerated | Benign | Tolerated |
| *FUT2* | Fucosyltransferase 2 | rs149356814 | 19:49206524 | 0.00319489 | 0.00356947 | 0.000581959 | 0.00386 | missense_variant | Damaging | Probably damaging | Damaging |
| *FUT2* | Fucosyltransferase 2 | [rs1800027](http://www.ncbi.nlm.nih.gov/projects/SNP/snp_ref.cgi?rs=rs1800027) | [19:49206726](genomebrowse://api/zoom?locus=19:49206726) | 0.0435304 | 0.0598806 | 0.0594722 | 0.062 | synonymous_variant | - | - | - |
| *FUT3* | Fucosyltransferase 3 (Lewis Blood Group) | rs28381969 | 19:5843877 | 0.00738818 | 0.00142419 | 0.00477162 | 0.002603 | missense_variant | Tolerated | Benign | Tolerated |
| *FUT3* | Fucosyltransferase 3 (Lewis Blood Group) | [rs3745635](http://www.ncbi.nlm.nih.gov/projects/SNP/snp_ref.cgi?rs=rs3745635) | [19:5844343](genomebrowse://api/zoom?locus=19:5844343) | 0.153554 | 0.0847992 | 0.107059 | 0.082 | missense_variant | Damaging | Probably damaging | Damaging |
| *FUT3* | Fucosyltransferase 3 (Lewis Blood Group) | [rs145362171](http://www.ncbi.nlm.nih.gov/projects/SNP/snp_ref.cgi?rs=rs145362171) | [19:5844804](genomebrowse://api/zoom?locus=19:5844804) | 0.0101837 | 0.0112666 | 0.0108323 | 0.011 | missense_variant | Tolerated | Possibly damaging | Tolerated |
| *FUT5* | Fucosyltransferase 5 | [rs61730513](http://www.ncbi.nlm.nih.gov/projects/SNP/snp_ref.cgi?rs=rs61730513) | [19:5867053](genomebrowse://api/zoom?locus=19:5867053) | 0.0543131 | 0.0228326 | 0.0450937 | 0.026 | synonymous_variant | - | - | - |
| *FUT5* | Fucosyltransferase 5 | [rs140776824](http://www.ncbi.nlm.nih.gov/projects/SNP/snp_ref.cgi?rs=rs140776824) | [19:5867154](genomebrowse://api/zoom?locus=19:5867154) | 0.0207668 | 0.0509375 | 0.0558982 | 0.05 | missense_variant | Tolerated | Benign | Tolerated |
| *FUT6* | Fucosyltransferase 6 | [rs61740561](http://www.ncbi.nlm.nih.gov/projects/SNP/snp_ref.cgi?rs=rs61740561) | [19:5831577](genomebrowse://api/zoom?locus=19:5831577) | 0.0738818 | 0.0465658 | 0.0763458 | 0.05 | synonymous_variant | - | - | - |
| *FUT6* | Fucosyltransferase 6 | [rs61739552](http://www.ncbi.nlm.nih.gov/projects/SNP/snp_ref.cgi?rs=rs61739552) | [19:5831602](genomebrowse://api/zoom?locus=19:5831602) | 0.0339457 | 0.0375533 | 0.0413274 | 0.039 | missense_variant | Tolerated | Benign | Tolerated |
| *FUT6* | Fucosyltransferase 6 | [rs141349215](http://www.ncbi.nlm.nih.gov/projects/SNP/snp_ref.cgi?rs=rs141349215) | [19:5831841](genomebrowse://api/zoom?locus=19:5831841) | 0.0233626 | 0.0353449 | 0.031533 | 0.036 | synonymous_variant | - | - | - |
| *FXN* | Frataxin | rs112826541 | 9:71661426 | 0.000599042 | 0.000591701 | 0.00048459 | 0.0004203 | intron_variant | - | - | - |
| *FYCO1* | FYVE And Coiled-Coil Domain Autophagy Adaptor 1 | rs140002692 | 3:46010179 | 0.00399361 | 0.000942147 | 0.00284054 | 0.001244 | missense_variant | Damaging | Probably damaging | Damaging |
| *FYCO1* | FYVE And Coiled-Coil Domain Autophagy Adaptor 1 | c.4277A>G | [3:45965232](genomebrowse://api/zoom?locus=3:45965232) | - | - | - | - | missense_variant | Tolerated | Benign | Damaging |
| *FYCO1* | FYVE And Coiled-Coil Domain Autophagy Adaptor 1 | [rs34147726](http://www.ncbi.nlm.nih.gov/projects/SNP/snp_ref.cgi?rs=rs34147726) | [3:46009620](genomebrowse://api/zoom?locus=3:46009620) | 0.0976438 | 0.0216044 | 0.0881231 | 0.028 | synonymous_variant | - | - | - |
| *GAA* | Glucosidase Alpha, Acid | [rs1800303](http://www.ncbi.nlm.nih.gov/projects/SNP/snp_ref.cgi?rs=rs1800303) | [17:78081661](genomebrowse://api/zoom?locus=17:78081661) | 0.110224 | 0.0717446 | 0.111792 | 0.075 | synonymous_variant | - | - | - |
| *GABBR2* | Gamma-Aminobutyric Acid Type B Receptor Subunit 2 | [rs3205936](http://www.ncbi.nlm.nih.gov/projects/SNP/snp_ref.cgi?rs=rs3205936) | [9:101147997](genomebrowse://api/zoom?locus=9:101147997) | 0.0519169 | 0.0121761 | 0.0489727 | 0.016 | synonymous_variant | - | - | - |
| *GABPB2* | GA Binding Protein Transcription Factor Subunit Beta 2 | [rs11204774](http://www.ncbi.nlm.nih.gov/projects/SNP/snp_ref.cgi?rs=rs11204774) | [1:151062957](genomebrowse://api/zoom?locus=1:151062957) | 0.0549121 | 0.0576928 | 0.0890641 | 0.06 | missense_variant | Tolerated | Probably damaging | Damaging |
| *GABRB2* | Gamma-Aminobutyric Acid Type A Receptor Subunit Beta2 | [rs2229944](http://www.ncbi.nlm.nih.gov/projects/SNP/snp_ref.cgi?rs=rs2229944) | [5:160721319](genomebrowse://api/zoom?locus=5:160721319) | 0.117812 | 0.114405 | 0.101694 | 0.106 | synonymous_variant | - | - | - |
| *GABRG3* | Gamma-Aminobutyric Acid Type A Receptor Subunit Gamma3 | rs28399526 | 15:27222234 | 0.000599042 | 0.00183586 | 0.00271178 | 0.001788 | missense_variant | Damaging | Possibly damaging | Damaging |
| *GADL1* | Glutamate Decarboxylase Like 1 | [rs6763895](http://www.ncbi.nlm.nih.gov/projects/SNP/snp_ref.cgi?rs=rs6763895) | [3:30891615](genomebrowse://api/zoom?locus=3:30891615) | 0.0451278 | 0.0110923 | 0.0412115 | 0.014 | intron_variant | - | - | - |
| *GAGE2A* | G Antigen 2A | rs782042171 | X:49355212 | 0.00847682 | 0.00203506 | 0.00337223 | 0.001878 | intron_variant | - | - | - |
| *GAK* | Cyclin G Associated Kinase | [rs41286651](http://www.ncbi.nlm.nih.gov/projects/SNP/snp_ref.cgi?rs=rs41286651) | [4:870941](genomebrowse://api/zoom?locus=4:870941) | 0.0115815 | 0.0227943 | 0.0209641 | 0.022 | synonymous_variant | - | - | - |
| *GAL3ST4* | Galactose-3-O-Sulfotransferase 4 | rs114072073 | 7:99764358 | 0.000199681 | 0.00026932 | 0.000840553 | 0.0003295 | missense_variant | Damaging | Possibly damaging | Damaging |
| *GALC* | Galactosylceramidase | [rs74337989](http://www.ncbi.nlm.nih.gov/projects/SNP/snp_ref.cgi?rs=rs74337989) | [14:88454469](genomebrowse://api/zoom?locus=14:88454469) | 0.0794728 | 0.126611 | 0.119595 | 0.124 | intron_variant | - | - | - |
| *GALC* | Galactosylceramidase | [rs78187751](http://www.ncbi.nlm.nih.gov/projects/SNP/snp_ref.cgi?rs=rs78187751) | [14:88458745](genomebrowse://api/zoom?locus=14:88458745) | 0.0790735 | 0.120682 | 0.117325 | 0.085 | intron_variant | - | - | - |
| *GALE* | UDP-Galactose-4-Epimerase | [rs1006253988](http://www.ncbi.nlm.nih.gov/projects/SNP/snp_ref.cgi?rs=rs1006253988) | [1:24124271](genomebrowse://api/zoom?locus=1:24124271) | - | - | - | - | missense_variant | Tolerated | Probably damaging | Damaging |
| *GALNS* | Galactosamine (N-Acetyl)-6-Sulfatase | rs143793386 | 16:88898513 | 0.00978434 | 0.0017591 | 0.0076529 | 0.002298 | splice_region_variant | - | - | - |
| *GALNS* | Galactosamine (N-Acetyl)-6-Sulfatase | rs148565559 | 16:88904097 | 0.000399361 | 0.00101937 | 0.00139006 | 0.0008978 | missense_variant | Tolerated | Possibly damaging | Damaging |
| *GALNS* | Galactosamine (N-Acetyl)-6-Sulfatase | [rs7187889](http://www.ncbi.nlm.nih.gov/projects/SNP/snp_ref.cgi?rs=rs7187889) | [16:88902643](genomebrowse://api/zoom?locus=16:88902643) | 0.0439297 | 0.0104989 | 0.0416209 | 0.013 | missense_variant | Damaging | Benign | Damaging |
| *GALNS* | Galactosamine (N-Acetyl)-6-Sulfatase | [rs34278797](http://www.ncbi.nlm.nih.gov/projects/SNP/snp_ref.cgi?rs=rs34278797) | [16:88908306](genomebrowse://api/zoom?locus=16:88908306) | 0.0115815 | 0.018441 | 0.0181565 | 0.018 | synonymous_variant | - | - | - |
| *GALNS* | Galactosamine (N-Acetyl)-6-Sulfatase | [rs11862754](http://www.ncbi.nlm.nih.gov/projects/SNP/snp_ref.cgi?rs=rs11862754) | [16:88909159](genomebrowse://api/zoom?locus=16:88909159) | 0.103834 | 0.0233939 | 0.0937803 | 0.03 | missense_variant | Tolerated | Benign | Damaging |
| *GALNT12* | Polypeptide N-Acetylgalactosaminyltransferase 12 | [rs3216734](http://www.ncbi.nlm.nih.gov/projects/SNP/snp_ref.cgi?rs=rs3216734) | [9:101599212](genomebrowse://api/zoom?locus=9:101599212) | 0.124601 | 0.202846 | 0.169213 | 0.197 | intron_variant | - | - | - |
| *GALNT14* | Polypeptide N-Acetylgalactosaminyltransferase 14 | [rs2288100](http://www.ncbi.nlm.nih.gov/projects/SNP/snp_ref.cgi?rs=rs2288100) | [2:31178818](genomebrowse://api/zoom?locus=2:31178818) | 0.127995 | 0.152996 | 0.135779 | 0.154 | synonymous_variant | - | - | - |
| *GAMT* | Guanidinoacetate N-Methyltransferase | [rs17851582](http://www.ncbi.nlm.nih.gov/projects/SNP/snp_ref.cgi?rs=rs17851582) | [19:1397443](genomebrowse://api/zoom?locus=19:1397443) | 0.0365415 | 0.0766035 | 0.0733657 | 0.075 | missense_variant | Tolerated | Possibly damaging | Damaging |
| *GANC* | Glucosidase Alpha, Neutral C | [rs2412688](http://www.ncbi.nlm.nih.gov/projects/SNP/snp_ref.cgi?rs=rs2412688) | [15:42575956](genomebrowse://api/zoom?locus=15:42575956) | 0.121805 | 0.0794772 | 0.0546042 | 0.043 | intron_variant | Tolerated | Benign | Damaging |
| *GANC* | Glucosidase Alpha, Neutral C | [rs16973008](http://www.ncbi.nlm.nih.gov/projects/SNP/snp_ref.cgi?rs=rs16973008) | [15:42579984](genomebrowse://api/zoom?locus=15:42579984) | 0.147165 | 0.0950175 | 0.0914386 | 0.092 | synonymous_variant | - | - | - |
| *GANC* | Glucosidase Alpha, Neutral C | [rs112976629](http://www.ncbi.nlm.nih.gov/projects/SNP/snp_ref.cgi?rs=rs112976629) | [15:42585029](genomebrowse://api/zoom?locus=15:42585029) | 0.0145767 | 0.0249011 | 0.0230357 | 0.025 | synonymous_variant | - | - | - |
| *GANC* | Glucosidase Alpha, Neutral C | [rs36023127](http://www.ncbi.nlm.nih.gov/projects/SNP/snp_ref.cgi?rs=rs36023127) | [15:42631928](genomebrowse://api/zoom?locus=15:42631928) | 0.0944489 | 0.0916539 | 0.0898082 | 0.093 | synonymous_variant | - | - | - |
| *GANC* | Glucosidase Alpha, Neutral C | [rs35039186](http://www.ncbi.nlm.nih.gov/projects/SNP/snp_ref.cgi?rs=rs35039186) | [15:42631976](genomebrowse://api/zoom?locus=15:42631976) | 0.0944489 | 0.0915976 | 0.0895836 | 0.093 | synonymous_variant | - | - | - |
| *GAREM* | GRB2-Associated And Regulator Of MAPK Protein 1 | [rs16962974](http://www.ncbi.nlm.nih.gov/projects/SNP/snp_ref.cgi?rs=rs16962974) | [18:29867091](genomebrowse://api/zoom?locus=18:29867091) | 0.0613019 | 0.0322818 | 0.0646557 | 0.035 | missense_variant | Damaging | Benign | Tolerated |
| *GARS* | Glycyl-TRNA Synthetase 1 | [rs4593](http://www.ncbi.nlm.nih.gov/projects/SNP/snp_ref.cgi?rs=rs4593) | [7:30673401](genomebrowse://api/zoom?locus=7:30673401) | 0.0764776 | 0.0363355 | 0.0814427 | 0.039 | synonymous_variant | - | - | - |
| *GBA3* | Glucosylceramidase Beta 3 (Gene/Pseudogene) | rs76988396 | 4:22749175 | 0.00199681 | 0.00742141 | 0.00862125 | 0.007242 | synonymous_variant | - | - | - |
| *GBE1* | 1,4-Alpha-Glucan Branching Enzyme 1 | [rs2228389](http://www.ncbi.nlm.nih.gov/projects/SNP/snp_ref.cgi?rs=rs2228389) | [3:81627175](genomebrowse://api/zoom?locus=3:81627175) | 0.0760783 | 0.04201 | 0.0610554 | 0.043 | missense_variant | Tolerated | Benign | Tolerated |
| *GBE1* | 1,4-Alpha-Glucan Branching Enzyme 1 | [rs17019144](http://www.ncbi.nlm.nih.gov/projects/SNP/snp_ref.cgi?rs=rs17019144) | [3:81698119](genomebrowse://api/zoom?locus=3:81698119) | 0.109824 | 0.0467394 | 0.0825086 | 0.049 | synonymous_variant | - | - | - |
| *GBE1* | 1,4-Alpha-Glucan Branching Enzyme 1 | [rs13320194](http://www.ncbi.nlm.nih.gov/projects/SNP/snp_ref.cgi?rs=rs13320194) | [3:81720076](genomebrowse://api/zoom?locus=3:81720076) | 0.105232 | 0.0396813 | 0.0797528 | 0.044 | synonymous_variant | - | - | - |
| *GBP6* | Guanylate Binding Protein Family Member 6 | [rs35837853](http://www.ncbi.nlm.nih.gov/projects/SNP/snp_ref.cgi?rs=rs35837853) | [1:89844035](genomebrowse://api/zoom?locus=1:89844035) | 0.102037 | 0.0844678 | 0.0563817 | 0.079 | missense_variant | Tolerated | Benign | Tolerated |
| *GBP7* | Guanylate Binding Protein 7 | [rs115671591](http://www.ncbi.nlm.nih.gov/projects/SNP/snp_ref.cgi?rs=rs115671591) | [1:89599013](genomebrowse://api/zoom?locus=1:89599013) | 0.0205671 | 0.0252711 | 0.018588 | 0.026 | synonymous_variant | - | - | - |
| *GBP7* | Guanylate Binding Protein 7 | [rs115454072](http://www.ncbi.nlm.nih.gov/projects/SNP/snp_ref.cgi?rs=rs115454072) | [1:89613280](genomebrowse://api/zoom?locus=1:89613280) | 0.0229633 | 0.0257861 | 0.0202519 | 0.026 | synonymous_variant | - | - | - |
| *GCKR* | Glucokinase Regulator | rs146175795 | 2:27721143 | 0.00439297 | 0.00270903 | 0.00100187 | 0.002281 | missense_variant | Damaging | Probably damaging | Damaging |
| *GCKR* | Glucokinase Regulator | [rs8179219](http://www.ncbi.nlm.nih.gov/projects/SNP/snp_ref.cgi?rs=rs8179219) | [2:27730817](genomebrowse://api/zoom?locus=2:27730817) | 0.0497204 | 0.0483423 | 0.0591527 | 0.051 | intron_variant | - | - | - |
| *GCSAML* | Germinal Center Associated Signaling And Motility Like | [rs56043070](http://www.ncbi.nlm.nih.gov/projects/SNP/snp_ref.cgi?rs=rs56043070) | [1:247719769](genomebrowse://api/zoom?locus=1:247719769) | 0.0389377 | 0.0518778 | 0.0509373 | 0.051 | splice_donor_variant | - | - | Damaging |
| *GCSH* | Glycine Cleavage System Protein H | [rs8177910](http://www.ncbi.nlm.nih.gov/projects/SNP/snp_ref.cgi?rs=rs8177910) | [16:81121183](genomebrowse://api/zoom?locus=16:81121183) | 0.0465256 | 0.0119652 | 0.0361395 | 0.014 | intron_variant | - | - | - |
| *GCSH* | Glycine Cleavage System Protein H | [rs8177908](http://www.ncbi.nlm.nih.gov/projects/SNP/snp_ref.cgi?rs=rs8177908) | [16:81121237](genomebrowse://api/zoom?locus=16:81121237) | 0.0465256 | 0.0119684 | 0.0362127 | 0.014 | synonymous_variant | - | - | - |
| *GCSH* | Glycine Cleavage System Protein H | [rs8177907](http://www.ncbi.nlm.nih.gov/projects/SNP/snp_ref.cgi?rs=rs8177907) | [16:81121246](genomebrowse://api/zoom?locus=16:81121246) | 0.0465256 | 0.0119772 | 0.0361317 | 0.014 | synonymous_variant | - | - | - |
| *GCSH* | Glycine Cleavage System Protein H | [rs8177877](http://www.ncbi.nlm.nih.gov/projects/SNP/snp_ref.cgi?rs=rs8177877) | [16:81124216](genomebrowse://api/zoom?locus=16:81124216) | 0.0435304 | 0.0114845 | 0.0339684 | 0.014 | missense_variant | Tolerated | Benign | Damaging |
| *GDF3* | Growth Differentiation Factor 3 | [rs17727707](http://www.ncbi.nlm.nih.gov/projects/SNP/snp_ref.cgi?rs=rs17727707) | [12:7848202](genomebrowse://api/zoom?locus=12:7848202) | 0.0267572 | 0.0679898 | 0.0733243 | 0.068 | synonymous_variant | - | - | - |
| *GDPD4* | Glycerophosphodiester Phosphodiesterase Domain Containing 4 | [rs61689775](http://www.ncbi.nlm.nih.gov/projects/SNP/snp_ref.cgi?rs=rs61689775) | [11:76928153](genomebrowse://api/zoom?locus=11:76928153) | 0.0573083 | 0.0124873 | 0.0534813 | 0.013 | 3_prime_UTR_variant | - | - | - |
| *GEMIN4* | Gem Nuclear Organelle Associated Protein 4 | [rs12942598](http://www.ncbi.nlm.nih.gov/projects/SNP/snp_ref.cgi?rs=rs12942598) | [17:648916](genomebrowse://api/zoom?locus=17:648916) | 0.0147764 | 0.0304429 | 0.028674 | 0.028 | synonymous_variant | - | - | - |
| *GEMIN8* | Gem Nuclear Organelle Associated Protein 8 | rs150567066 | X:14038534 | 0.00927152 | 0.00208776 | 0.00738162 | 0.002553 | synonymous_variant | - | - | - |
| *GFER* | Growth Factor, Augmenter Of Liver Regeneration | [rs1802834](http://www.ncbi.nlm.nih.gov/projects/SNP/snp_ref.cgi?rs=rs1802834) | [16:2035999](genomebrowse://api/zoom?locus=16:2035999) | 0.0333466 | 0.0687585 | 0.0737498 | 0.067 | synonymous_variant | - | - | - |
| *GFI1* | Growth Factor Independent 1 Transcriptional Repressor | [rs200902533](http://www.ncbi.nlm.nih.gov/projects/SNP/snp_ref.cgi?rs=rs200902533) | [1:92944315](genomebrowse://api/zoom?locus=1:92944315) | - | - | 0.125315 | 0.1 | splice_region_variant | - | - | - |
| *GFI1B* | Growth Factor Independent 1B Transcriptional Repressor | [rs60757417](http://www.ncbi.nlm.nih.gov/projects/SNP/snp_ref.cgi?rs=rs60757417) | [9:135864436](genomebrowse://api/zoom?locus=9:135864436) | 0.0666933 | 0.0637026 | 0.0639591 | 0.066 | intron_variant | - | - | - |
| *GFM1* | G Elongation Factor Mitochondrial 1 | rs147759114 | 3:158386886 | 0.00519169 | 0.00145576 | 0.00597931 | 0.001969 | missense_variant | Tolerated | Benign | Damaging |
| *GFPT1* | Glutamine--Fructose-6-Phosphate Transaminase 1 | [rs201268947](http://www.ncbi.nlm.nih.gov/projects/SNP/snp_ref.cgi?rs=rs201268947) | [2:69553376](genomebrowse://api/zoom?locus=2:69553376) | - | 0.0662111 | 0.0348448 | 0.081 | intron_variant | - | - | - |
| *GFPT2* | Glutamine-Fructose-6-Phosphate Transaminase 2 | [rs2112594](http://www.ncbi.nlm.nih.gov/projects/SNP/snp_ref.cgi?rs=rs2112594) | [5:179744009](genomebrowse://api/zoom?locus=5:179744009) | 0.142372 | 0.17338 | 0.151199 | 0.168 | synonymous_variant | - | - | - |
| *GGCX* | Gamma-Glutamyl Carboxylase | [rs11676382](http://www.ncbi.nlm.nih.gov/projects/SNP/snp_ref.cgi?rs=rs11676382) | [2:85777633](genomebrowse://api/zoom?locus=2:85777633) | 0.0255591 | 0.0574289 | 0.0635618 | 0.057 | intron_variant | - | - | - |
| *GGNBP1* | Gametogenetin Binding Protein 1 (Pseudogene) | rs115275555 | 6:33553439 | 0.00359425 | 0.000775477 | 0.00371567 | 0.0008518 | non_coding_exon_variant | - | - | - |
| *GGT1* | Gamma-Glutamyltransferase 1 | rs45519234 | 22:25024172 | 0.000399361 | 0.000618168 | 0.000583582 | 0.0005518 | intron_variant | - | - | - |
| *GGTLC2* | Gamma-Glutamyltransferase Light Chain 2 | [rs74553569](http://www.ncbi.nlm.nih.gov/projects/SNP/snp_ref.cgi?rs=rs74553569) | [22:22989622](genomebrowse://api/zoom?locus=22:22989622) | 0.141973 | 0.102671 | 0.0831062 | 0.107 | synonymous_variant | - | - | - |
| *GH1* | Growth Hormone 1 | rs71640274 | 17:61995743 | 0.000199681 | 0.000129949 | 0.000581583 | 0.0001483 | missense_variant | Tolerated | Benign | Damaging |
| *GHR* | Growth Hormone Receptor | rs149475648 | 5:42688957 | 0.00139776 | 0.00377484 | 0.00338972 | 0.003781 | intron_variant | - | - | - |
| *GHRHR* | Growth Hormone Releasing Hormone Receptor | rs10227922 | 7:31011625 | 0.000798722 | 0.000235531 | 0.00119571 | 0.000313 | missense_variant | Tolerated | Probably damaging | Damaging |
| *GHRHR* | Growth Hormone Releasing Hormone Receptor | [rs4988496](http://www.ncbi.nlm.nih.gov/projects/SNP/snp_ref.cgi?rs=rs4988496) | [7:31008686](genomebrowse://api/zoom?locus=7:31008686) | 0.127396 | 0.0635728 | 0.110755 | 0.058 | missense_variant | Tolerated | Benign | Tolerated |
| *GHRHR* | Growth Hormone Releasing Hormone Receptor | [rs4988498](http://www.ncbi.nlm.nih.gov/projects/SNP/snp_ref.cgi?rs=rs4988498) | [7:31009576](genomebrowse://api/zoom?locus=7:31009576) | 0.0640974 | 0.0450289 | 0.0635864 | 0.047 | missense_variant | Tolerated | Benign | Damaging |
| *GHRHR* | Growth Hormone Releasing Hormone Receptor | [rs2228078](http://www.ncbi.nlm.nih.gov/projects/SNP/snp_ref.cgi?rs=rs2228078) | [7:31018852](genomebrowse://api/zoom?locus=7:31018852) | 0.0371406 | 0.0505061 | 0.0204754 | 0.041 | missense_variant | Tolerated | Benign | Damaging |
| *GIGYF2* | GRB10 Interacting GYF Protein 2 | rs114498122 | 2:233671277 | 0.00259585 | 0.00491813 | 0.00287282 | 0.005074 | synonymous_variant | - | - | - |
| *GIMAP1-GIMAP5* | GIMAP1-GIMAP5 Readthrough | [rs61751040](http://www.ncbi.nlm.nih.gov/projects/SNP/snp_ref.cgi?rs=rs61751040) | [7:150439554](genomebrowse://api/zoom?locus=7:150439554) | 0.0593051 | 0.0149467 | 0.0440939 | 0.018 | synonymous_variant | - | - | - |
| *GIMAP2* | GTPase, IMAP Family Member 2 | [rs1860871](http://www.ncbi.nlm.nih.gov/projects/SNP/snp_ref.cgi?rs=rs1860871) | [7:150389593](genomebrowse://api/zoom?locus=7:150389593) | 0.0990415 | 0.0965244 | 0.0877295 | 0.094 | synonymous_variant | - | - | - |
| *GINM1* | Glycoprotein Integral Membrane 1 | [rs1137086](http://www.ncbi.nlm.nih.gov/projects/SNP/snp_ref.cgi?rs=rs1137086) | [6:149903597](genomebrowse://api/zoom?locus=6:149903597) | 0.0155751 | 0.0301609 | 0.0281831 | 0.031 | missense_variant | Tolerated | Benign | Tolerated |
| *GJA3* | Gap Junction Protein Alpha 3 | [rs74607195](http://www.ncbi.nlm.nih.gov/projects/SNP/snp_ref.cgi?rs=rs74607195) | [13:20716885](genomebrowse://api/zoom?locus=13:20716885) | 0.0121805 | 0.0349743 | 0.0463131 | 0.035 | synonymous_variant | - | - | - |
| *GJA8* | Gap Junction Protein Alpha 8 | [rs3766503](http://www.ncbi.nlm.nih.gov/projects/SNP/snp_ref.cgi?rs=rs3766503) | [1:147380886](genomebrowse://api/zoom?locus=1:147380886) | 0.0491214 | 0.0403897 | 0.049334 | 0.043 | synonymous_variant | - | - | - |
| *GJB3* | Gap Junction Protein Beta 3 | rs61744512 | 1:35250942 | 0.00519169 | 0.00149896 | 0.00443452 | 0.001664 | synonymous_variant | - | - | - |
| *GJB4* | Gap Junction Protein Beta 4 | [rs78499418](http://www.ncbi.nlm.nih.gov/projects/SNP/snp_ref.cgi?rs=rs78499418) | [1:35227306](genomebrowse://api/zoom?locus=1:35227306) | 0.0447284 | 0.0260416 | 0.0306215 | 0.024 | missense_variant | Tolerated | Benign | Tolerated |
| *GJB7* | Gap Junction Protein Beta 7 | [rs35259282](http://www.ncbi.nlm.nih.gov/projects/SNP/snp_ref.cgi?rs=rs35259282) | [6:87994504](genomebrowse://api/zoom?locus=6:87994504) | 0.0353434 | 0.0616981 | 0.0588597 | 0.063 | missense_variant | Damaging | Probably damaging | Damaging |
| *GK* | Glycerol Kinase | [rs34795481](http://www.ncbi.nlm.nih.gov/projects/SNP/snp_ref.cgi?rs=rs34795481) | [X:30686141](genomebrowse://api/zoom?locus=X:30686141) | 0.0312583 | 0.0519518 | 0.0544677 | 0.052 | synonymous_variant | - | - | - |
| *GK* | Glycerol Kinase | [rs41305205](http://www.ncbi.nlm.nih.gov/projects/SNP/snp_ref.cgi?rs=rs41305205) | [X:30714817](genomebrowse://api/zoom?locus=X:30714817) | 0.0312583 | 0.0509708 | 0.0543824 | 0.052 | intron_variant | - | - | - |
| *GK* | Glycerol Kinase | [rs41311819](http://www.ncbi.nlm.nih.gov/projects/SNP/snp_ref.cgi?rs=rs41311819) | [X:30738878](genomebrowse://api/zoom?locus=X:30738878) | 0.0317881 | 0.0528539 | 0.054485 | 0.052 | intron_variant | - | - | - |
| *GK3P* | Glycerol Kinase 3 Pseudogene | [rs7655933](http://www.ncbi.nlm.nih.gov/projects/SNP/snp_ref.cgi?rs=rs7655933) | [4:166200045](genomebrowse://api/zoom?locus=4:166200045) | 0.0471246 | 0.0102371 | 0.0382305 | 0.013 | intron_variant | - | - | - |
| *GKN1* | Gastrokine 1 | [rs35624281](http://www.ncbi.nlm.nih.gov/projects/SNP/snp_ref.cgi?rs=rs35624281) | [2:69204629](genomebrowse://api/zoom?locus=2:69204629) | 0.134585 | 0.0853092 | 0.0646892 | 0.085 | synonymous_variant | - | - | - |
| *GLB1L2* | Galactosidase Beta 1 Like 2 | [rs3802928](http://www.ncbi.nlm.nih.gov/projects/SNP/snp_ref.cgi?rs=rs3802928) | [11:134226278](genomebrowse://api/zoom?locus=11:134226278) | 0.126597 | 0.0728185 | 0.0992115 | 0.076 | synonymous_variant | - | - | - |
| *GLDC* | Glycine Decarboxylase | [rs3215923](http://www.ncbi.nlm.nih.gov/projects/SNP/snp_ref.cgi?rs=rs3215923) | [9:6553515](genomebrowse://api/zoom?locus=9:6553515) | 0.242612 | 0.296597 | 0.26456 | 0.296 | splice_region_variant | - | - | - |
| *GLDC* | Glycine Decarboxylase | [rs2228095](http://www.ncbi.nlm.nih.gov/projects/SNP/snp_ref.cgi?rs=rs2228095) | [9:6606645](genomebrowse://api/zoom?locus=9:6606645) | 0.0814696 | 0.0201948 | 0.0669683 | 0.024 | synonymous_variant | - | - | - |
| *GLIS2* | GLIS Family Zinc Finger 2 | [rs28449480](http://www.ncbi.nlm.nih.gov/projects/SNP/snp_ref.cgi?rs=rs28449480) | [16:4382271](genomebrowse://api/zoom?locus=16:4382271) | 0.0555112 | 0.0138473 | 0.0530543 | 0.017 | 5_prime_UTR_variant | - | - | - |
| *GLRB* | Glycine Receptor Beta | [rs1801154](http://www.ncbi.nlm.nih.gov/projects/SNP/snp_ref.cgi?rs=rs1801154) | [4:158073913](genomebrowse://api/zoom?locus=4:158073913) | 0.0621006 | 0.0458964 | 0.0482496 | 0.047 | synonymous_variant | - | - | - |
| *GLRX5* | Glutaredoxin 5 | [rs11628901](http://www.ncbi.nlm.nih.gov/projects/SNP/snp_ref.cgi?rs=rs11628901) | [14:96010424](genomebrowse://api/zoom?locus=14:96010424) | 0.0830671 | 0.123153 | 0.105381 | 0.119 | missense_variant | Damaging | Benign | Damaging |
| *GLT8D2* | Glycosyltransferase 8 Domain Containing 2 | [rs79806135](http://www.ncbi.nlm.nih.gov/projects/SNP/snp_ref.cgi?rs=rs79806135) | [12:104387138](genomebrowse://api/zoom?locus=12:104387138) | 0.120807 | 0.113093 | 0.0898612 | 0.11 | intron_variant | - | - | - |
| *GLTSCR1* | Glioma Tumor Suppressor Candidate Region Gene 1 Protein | rs181534456 | 19:48205456 | 0.0061901 | 0.00288014 | 0.00692915 | 0.002859 | synonymous_variant | - | - | - |
| *GLTSCR1L* | Glioma Tumor Suppressor Candidate Region Gene 1 Protein-Like | rs76373914 | 6:42823669 | 0.00259585 | 0.000739152 | 0.00164814 | 0.000733 | intron_variant | - | - | - |
| *GLTSCR2* | Glioma Tumor Suppressor Candidate Region Gene 2 Protein | [rs11538669](http://www.ncbi.nlm.nih.gov/projects/SNP/snp_ref.cgi?rs=rs11538669) | [19:48254799](genomebrowse://api/zoom?locus=19:48254799) | 0.0153754 | 0.0186447 | 0.0215674 | 0.019 | synonymous_variant | - | - | - |
| *GLUD2* | Glutamate Dehydrogenase 2 | [rs9697983](http://www.ncbi.nlm.nih.gov/projects/SNP/snp_ref.cgi?rs=rs9697983) | [X:120183030](genomebrowse://api/zoom?locus=X:120183030) | 0.0328477 | 0.0265981 | 0.0355199 | 0.028 | missense_variant | Tolerated | Benign | Damaging |
| *GLUL* | Glutamate-Ammonia Ligase | [rs17462824](http://www.ncbi.nlm.nih.gov/projects/SNP/snp_ref.cgi?rs=rs17462824) | [1:182356247](genomebrowse://api/zoom?locus=1:182356247) | 0.0427316 | 0.0732256 | 0.0790613 | 0.072 | intron_variant | - | - | - |
| *GM2A* | GM2 Ganglioside Activator | [rs1048719](http://www.ncbi.nlm.nih.gov/projects/SNP/snp_ref.cgi?rs=rs1048719) | [5:150632832](genomebrowse://api/zoom?locus=5:150632832) | 0.0800719 | 0.0604002 | 0.0407596 | 0.057 | missense_variant | Tolerated | Benign | Tolerated |
| *GMPPA* | GDP-Mannose Pyrophosphorylase A | rs150386940 | 2:220367115 | 0.00199681 | 0.0084108 | 0.00986736 | 0.008368 | synonymous_variant | - | - | - |
| *GNAI1* | G Protein Subunit Alpha I1 | [rs17153555](http://www.ncbi.nlm.nih.gov/projects/SNP/snp_ref.cgi?rs=rs17153555) | [7:79818277](genomebrowse://api/zoom?locus=7:79818277) | 0.0307508 | 0.013444 | 0.0294707 | 0.015 | synonymous_variant | - | - | - |
| *GNAT1* | G Protein Subunit Alpha Transducin 1 | c.766G>T | [3:50231991](genomebrowse://api/zoom?locus=3:50231991) | - | - | - | - | missense_variant | Tolerated | Benign | Damaging |
| *GNAT2* | G Protein Subunit Alpha Transducin 2 | [rs3738766](http://www.ncbi.nlm.nih.gov/projects/SNP/snp_ref.cgi?rs=rs3738766) | [1:110151395](genomebrowse://api/zoom?locus=1:110151395) | 0.0886581 | 0.0500536 | 0.0324529 | 0.05 | missense_variant | Tolerated | Probably damaging | Damaging |
| *GNB3* | G Protein Subunit Beta 3 | [rs5442](http://www.ncbi.nlm.nih.gov/projects/SNP/snp_ref.cgi?rs=rs5442) | [12:6954864](genomebrowse://api/zoom?locus=12:6954864) | 0.0195687 | 0.0488952 | 0.045337 | 0.05 | missense_variant | Damaging | Probably damaging | Damaging |
| *GNE* | Glucosamine (UDP-N-Acetyl)-2-Epimerase/N-Acetylmannosamine Kinase | rs115052162 | 9:36227218 | 0.0071885 | 0.00181938 | 0.00713502 | 0.002438 | intron_variant | - | - | - |
| *GNL3* | G Protein Nucleolar 3 | rs183506718 | 3:52723293 | 0.000599042 | 0.000144881 | 0.000516729 | 0.0001895 | intron_variant | - | - | - |
[truncated: 715,111 more chars]
