# Supplementary material for: Identification of Novel Variants in Cleft Palate-Associated Genes in Brazilian Patients With Non-syndromic Cleft Palate Only
Source: Front Cell Dev Biol. 2021 Jul 8;9:638522. doi: 10.3389/fcell.2021.638522 (PMC8297955; doi:10.3389/fcell.2021.638522)
Supplement: Supplementary file 7 [file Data_Sheet_5.docx]

**Supplementary Table 5.** List of the biological processes for the dataset of variant-containing-genes identified in patients with nonsyndromic cleft palate only (NSCPO).

| **GO ID** | **Term description** | **Observed gene count** | **Background gene count** | **False discovery rate** | **Matching proteins in your network** |
| --- | --- | --- | --- | --- | --- |
| **GO:0001501** | skeletal system development | 12 | 457 | 1.96e-10 | MTHFD1, SOX9, LRP6, TP63, TYMS, JAG2, TBX1, COL11A1, COL11A2, TCOF1, COL2A1, MSX1 |
| **GO:0035999*** | tetrahydrofolate interconversion | 5 | 9 | 3.57e-09 | MTHFD1, FTCD, TYMS, SHMT1, MTHFR |
| **GO:0042558*** | pteridine-containing compound metabolic process | 6 | 33 | 3.88e-09 | MTHFD1, FTCD, TYMS, SHMT1, MTR, MTHFR |
| **GO:0048598** | embryonic morphogenesis | 11 | 545 | 1.10e-08 | MTHFD1, SOX9, LRP6, CREBBP, TP63, GRHL3, JAG2, TBX1, COL11A1, COL2A1, MSX1 |
| **GO:0009790** | embryo development | 12 | 890 | 6.83e-08 | MTHFD1, SOX9, LRP6, CREBBP, TP63, GRHL3, JAG2, TBX1, COL11A1, TCOF1, COL2A1, MSX1 |
| **GO:0043009** | chordate embryonic development | 10 | 550 | 1.25e-07 | MTHFD1, SOX9, LRP6, GRHL3, JAG2, TBX1, COL11A1, TCOF1, COL2A1, MSX1 |
| **GO:0051216** | cartilage development | 7 | 147 | 1.25e-07 | SOX9, LRP6, TYMS, COL11A1, COL11A2, COL2A1, MSX1 |
| **GO:0060021** | roof of mouth development | 6 | 87 | 2.17e-07 | LRP6, JAG2, TBX1, COL11A2, COL2A1, MSX1 |
| **GO:0043583** | ear development | 7 | 204 | 7.20e-07 | SOX9, GRHL3, JAG2, TBX1, COL11A1, COL2A1, MSX1 |
| **GO:0042471*** | ear morphogenesis | 6 | 112 | 7.34e-07 | SOX9, GRHL3, TBX1, COL11A1, COL2A1, MSX1 |
| **GO:0048729** | tissue morphogenesis | 9 | 522 | 1.04e-06 | MTHFD1, SOX9, LRP6, TP63, GRHL3, JAG2, TBX1, COL11A1, MSX1 |
| **GO:0048863** | stem cell differentiation | 6 | 131 | 1.59e-06 | SOX9, LRP6, TP63, TBX1, TCOF1, MSX1 |
| **GO:0009888** | tissue development | 13 | 1626 | 1.69e-06 | MTHFD1, SOX9, LRP6, TP63, GRHL3, TYMS, JAG2, TBX1, COL11A1, COL11A2, TCOF1, COL2A1, MSX1 |
| **GO:0090596** | sensory organ morphogenesis | 7 | 248 | 1.96e-06 | SOX9, LRP6, GRHL3, TBX1, COL11A1, COL2A1, MSX1 |
| **GO:0016331*** | morphogenesis of embryonic epithelium | 6 | 141 | 2.06e-06 | MTHFD1, SOX9, LRP6, TP63, GRHL3, JAG2 |
| **GO:0048646** | anatomical structure formation involved in morphogenesis | 10 | 831 | 2.83e-06 | MTHFD1, SOX9, LRP6, TP63, GRHL3, TBX1, COL11A1, TCOF1, COL2A1, MSX1 |
| **GO:0048562** | embryonic organ morphogenesis | 7 | 279 | 3.72e-06 | MTHFD1, SOX9, GRHL3, TBX1, COL11A1, COL2A1, MSX1 |
| **GO:0009887** | animal organ morphogenesis | 10 | 865 | 3.76e-06 | MTHFD1, SOX9, LRP6, TP63, GRHL3, JAG2, TBX1, COL11A1, COL2A1, MSX1 |
| **GO:0042475** | odontogenesis of dentin-containing tooth | 5 | 78 | 4.07e-06 | LRP6, TP63, JAG2, TBX1, MSX1 |
| **GO:0006767*** | water-soluble vitamin metabolic process | 5 | 81 | 4.67e-06 | TCN2, MTHFD1, SHMT1, MTR, MTHFR |
| **GO:0007275** | multicellular organism development | 19 | 4726 | 4.67e-06 | MTHFD1, SOX9, LRP6, CDH1, CREBBP, TP63, GRHL3, TYMS, JAG2, TBX1, POMGNT2, OFD1, MTR, COL11A1, TBX22, COL11A2, TCOF1, COL2A1, MSX1 |
| **GO:0051186*** | cofactor metabolic process | 8 | 467 | 5.10e-06 | TCN2, MTHFD1, FTCD, TYMS, SHMT1, ACACB, MTR, MTHFR |
| **GO:0048839** | inner ear development | 6 | 177 | 5.43e-06 | SOX9, GRHL3, JAG2, TBX1, COL11A1, COL2A1 |
| **GO:0042472** | inner ear morphogenesis | 5 | 92 | 7.38e-06 | SOX9, GRHL3, TBX1, COL11A1, COL2A1 |
| **GO:0007423** | sensory organ development | 8 | 515 | 9.60e-06 | SOX9, LRP6, GRHL3, JAG2, TBX1, COL11A1, COL2A1, MSX1 |
| **GO:0048705** | skeletal system morphogenesis | 6 | 204 | 1.11e-05 | MTHFD1, SOX9, TBX1, COL11A1, COL2A1, MSX1 |
| **GO:0032501** | multicellular organismal process | 21 | 6507 | 1.28e-05 | MTHFD1, SOX9, LRP6, CDH1, CREBBP, TP63, GRHL3, TYMS, JAG2, TBX1, ACACB, POMGNT2, OFD1, MTR, COL11A1, TBX22, COL11A2, MTHFR, TCOF1, COL2A1, MSX1 |
| **GO:0060429** | epithelium development | 10 | 1055 | 1.60e-05 | MTHFD1, SOX9, LRP6, TP63, GRHL3, TYMS, JAG2, TBX1, COL2A1, MSX1 |
| **GO:0014029*** | neural crest formation | 3 | 11 | 2.91e-05 | SOX9, LRP6, TCOF1 |
| **GO:0048762** | mesenchymal cell differentiation | 5 | 130 | 2.93e-05 | SOX9, LRP6, TBX1, TCOF1, MSX1 |
| **GO:2001053*** | regulation of mesenchymal cell apoptotic process | 3 | 12 | 3.28e-05 | SOX9, TBX1, MSX1 |
| **GO:0035108** | limb morphogenesis | 5 | 147 | 4.77e-05 | SOX9, CREBBP, TP63, COL2A1, MSX1 |
| **GO:0006732*** | coenzyme metabolic process | 6 | 297 | 6.60e-05 | MTHFD1, FTCD, TYMS, SHMT1, ACACB, MTHFR |
| **GO:0009653** | anatomical structure morphogenesis | 12 | 1992 | 6.60e-05 | MTHFD1, SOX9, LRP6, CREBBP, TP63, GRHL3, JAG2, TBX1, COL11A1, TCOF1, COL2A1, MSX1 |
| **GO:0014033*** | neural crest cell differentiation | 4 | 66 | 6.60e-05 | SOX9, LRP6, TBX1, TCOF1 |
| **GO:0001837** | epithelial to mesenchymal transition | 4 | 68 | 6.78e-05 | SOX9, LRP6, TCOF1, MSX1 |
| **GO:0007507** | heart development | 7 | 485 | 6.78e-05 | MTHFD1, SOX9, LRP6, TBX1, COL11A1, COL2A1, MSX1 |
| **GO:0046655*** | folic acid metabolic process | 3 | 18 | 7.97e-05 | MTHFD1, SHMT1, MTHFR |
| **GO:0048513** | animal organ development | 14 | 2926 | 7.99e-05 | MTHFD1, SOX9, LRP6, CDH1, TP63, GRHL3, TYMS, JAG2, TBX1, COL11A1, COL11A2, TCOF1, COL2A1, MSX1 |
| **GO:0001502*** | cartilage condensation | 3 | 20 | 9.80e-05 | SOX9, COL11A1, COL2A1 |
| **GO:0006555*** | methionine metabolic process | 3 | 20 | 9.80e-05 | MTHFD1, MTR, MTHFR |
| **GO:0035295** | tube development | 8 | 793 | 0.00012 | MTHFD1, SOX9, LRP6, TP63, GRHL3, TYMS, TBX1, COL2A1 |
| **GO:0048731** | system development | 16 | 4144 | 0.00013 | MTHFD1, SOX9, LRP6, CDH1, TP63, GRHL3, TYMS, JAG2, TBX1, POMGNT2, MTR, COL11A1, COL11A2, TCOF1, COL2A1, MSX1 |
| **GO:0062009*** | secondary palate development | 3 | 23 | 0.00013 | JAG2, TBX1, COL11A2 |
| **GO:0007399** | nervous system development | 12 | 2206 | 0.00014 | MTHFD1, SOX9, LRP6, CDH1, TP63, GRHL3, JAG2, TBX1, POMGNT2, MTR, COL2A1, MSX1 |
| **GO:0090103*** | cochlea morphogenesis | 3 | 24 | 0.00014 | SOX9, GRHL3, TBX1 |
| **GO:0048704** | embryonic skeletal system morphogenesis | 4 | 92 | 0.00016 | MTHFD1, TBX1, COL11A1, COL2A1 |
| **GO:1901605*** | alpha-amino acid metabolic process | 5 | 209 | 0.00017 | MTHFD1, FTCD, SHMT1, MTR, MTHFR |
| **GO:0043648*** | dicarboxylic acid metabolic process | 4 | 97 | 0.00019 | MTHFD1, FTCD, SHMT1, MTHFR |
| **GO:0002053*** | positive regulation of mesenchymal cell proliferation | 3 | 28 | 0.00020 | SOX9, TP63, TBX1 |
| **GO:0048732** | gland development | 6 | 395 | 0.00022 | SOX9, CDH1, TP63, TYMS, TBX1, MSX1 |
| **GO:0017144*** | drug metabolic process | 7 | 622 | 0.00024 | TCN2, MTHFD1, FTCD, TYMS, SHMT1, MTR, MTHFR |
| **GO:0007389** | pattern specification process | 6 | 409 | 0.00025 | LRP6, TP63, GRHL3, TBX1, OFD1, MSX1 |
| **GO:0042493** | response to drug | 8 | 900 | 0.00025 | TCN2, SOX9, CDH1, TYMS, ACACB, MTR, MTHFR, MSX1 |
| **GO:0060325** | face morphogenesis | 3 | 31 | 0.00025 | LRP6, TBX1, MSX1 |
| **GO:0003007** | heart morphogenesis | 5 | 235 | 0.00026 | SOX9, TBX1, COL11A1, COL2A1, MSX1 |
| **GO:0006231*** | dTMP biosynthetic process | 2 | 3 | 0.00034 | TYMS, SHMT1 |
| **GO:0007219** | Notch signaling pathway | 4 | 116 | 0.00034 | SOX9, CREBBP, TP63, JAG2 |
| **GO:0001838** | embryonic epithelial tube formation | 4 | 120 | 0.00036 | MTHFD1, SOX9, LRP6, GRHL3 |
| **GO:0035270** | endocrine system development | 4 | 120 | 0.00036 | SOX9, CDH1, TBX1, MSX1 |
| **GO:0009112*** | nucleobase metabolic process | 3 | 38 | 0.00038 | MTHFD1, TYMS, SHMT1 |
| **GO:0030199** | collagen fibril organization | 3 | 39 | 0.00040 | COL11A1, COL11A2, COL2A1 |
| **GO:0046483** | heterocycle metabolic process | 16 | 4716 | 0.00050 | TCN2, MTHFD1, SOX9, CREBBP, TP63, GRHL3, FTCD, TYMS, SHMT1, TBX1, ACACB, MTR, TBX22, MTHFR, RFC1, MSX1 |
| **GO:0048871** | multicellular organismal homeostasis | 5 | 283 | 0.00051 | SOX9, TP63, GRHL3, ACACB, COL2A1 |
| **GO:0006725** | cellular aromatic compound metabolic process | 16 | 4754 | 0.00054 | TCN2, MTHFD1, SOX9, CREBBP, TP63, GRHL3, FTCD, TYMS, SHMT1, TBX1, ACACB, MTR, TBX22, MTHFR, RFC1, MSX1 |
| **GO:0030198** | extracellular matrix organization | 5 | 296 | 0.00058 | SOX9, CDH1, COL11A1, COL11A2, COL2A1 |
| **GO:0060023*** | soft palate development | 2 | 5 | 0.00058 | TBX1, COL11A2 |
| **GO:0097065*** | anterior head development | 2 | 5 | 0.00058 | SOX9, COL2A1 |
| **GO:0007605*** | sensory perception of sound | 4 | 144 | 0.00059 | TBX1, COL11A1, COL11A2, COL2A1 |
| **GO:0045747** | positive regulation of Notch signaling pathway | 3 | 48 | 0.00061 | CREBBP, TP63, JAG2 |
| **GO:0045893** | positive regulation of transcription, DNA-templated | 9 | 1435 | 0.00070 | SOX9, LRP6, CDH1, CREBBP, TP63, GRHL3, TBX1, RFC1, MSX1 |
| **GO:0001101** | response to acid chemical | 5 | 323 | 0.00082 | SOX9, TYMS, SHMT1, TBX1, MTHFR |
| **GO:1901360** | organic cyclic compound metabolic process | 16 | 4963 | 0.00083 | TCN2, MTHFD1, SOX9, CREBBP, TP63, GRHL3, FTCD, TYMS, SHMT1, TBX1, ACACB, MTR, TBX22, MTHFR, RFC1, MSX1 |
| **GO:0042592** | homeostatic process | 9 | 1491 | 0.00088 | MTHFD1, SOX9, LRP6, CREBBP, TP63, GRHL3, ACACB, COL2A1, RFC1 |
| **GO:0014032*** | neural crest cell development | 3 | 58 | 0.00094 | SOX9, TBX1, TCOF1 |
| **GO:0002062*** | chondrocyte differentiation | 3 | 60 | 0.00100 | SOX9, COL11A1, COL2A1 |
| **GO:0019752*** | carboxylic acid metabolic process | 7 | 854 | 0.0010 | MTHFD1, FTCD, TYMS, SHMT1, ACACB, MTR, MTHFR |
| **GO:0060534*** | trachea cartilage development | 2 | 8 | 0.00100 | SOX9, LRP6 |
| **GO:1901607*** | alpha-amino acid biosynthetic process | 3 | 60 | 0.00100 | MTHFD1, SHMT1, MTR |
| **GO:0007417** | central nervous system development | 7 | 861 | 0.0011 | SOX9, LRP6, CDH1, GRHL3, TBX1, COL2A1, MSX1 |
| **GO:0010468** | regulation of gene expression | 15 | 4533 | 0.0012 | SOX9, LRP6, CDH1, CREBBP, TP63, GRHL3, TYMS, SHMT1, TBX1, ACACB, TBX22, TCOF1, COL2A1, RFC1, MSX1 |
| **GO:1904888*** | cranial skeletal system development | 3 | 66 | 0.0012 | MTHFD1, TP63, TBX1 |
| **GO:0006547*** | histidine metabolic process | 2 | 10 | 0.0013 | MTHFD1, FTCD |
| **GO:0030154** | cell differentiation | 13 | 3457 | 0.0013 | SOX9, LRP6, CDH1, TP63, TYMS, JAG2, TBX1, POMGNT2, MTR, COL11A1, TCOF1, COL2A1, MSX1 |
| **GO:0035239** | tube morphogenesis | 6 | 615 | 0.0013 | MTHFD1, SOX9, LRP6, TP63, GRHL3, TBX1 |
| **GO:2001054*** | negative regulation of mesenchymal cell apoptotic process | 2 | 10 | 0.0013 | SOX9, TBX1 |
| **GO:0048703*** | embryonic viscerocranium morphogenesis | 2 | 11 | 0.0015 | MTHFD1, TBX1 |
| **GO:0051593*** | response to folic acid | 2 | 11 | 0.0015 | TYMS, MTHFR |
| **GO:0060174*** | limb bud formation | 2 | 11 | 0.0015 | SOX9, COL2A1 |
| **GO:0060536*** | cartilage morphogenesis | 2 | 11 | 0.0015 | LRP6, MSX1 |
| **GO:0009086*** | methionine biosynthetic process | 2 | 12 | 0.0016 | MTHFD1, MTR |
| **GO:0009113*** | purine nucleobase biosynthetic process | 2 | 12 | 0.0016 | MTHFD1, SHMT1 |
| **GO:0010629** | negative regulation of gene expression | 9 | 1670 | 0.0016 | SOX9, CREBBP, TP63, TYMS, SHMT1, ACACB, TBX22, RFC1, MSX1 |
| **GO:0021536** | diencephalon development | 3 | 75 | 0.0016 | LRP6, CDH1, MSX1 |
| **GO:0034654** | nucleobase-containing compound biosynthetic process | 12 | 3031 | 0.0016 | MTHFD1, SOX9, CREBBP, TP63, GRHL3, TYMS, SHMT1, TBX1, ACACB, TBX22, RFC1, MSX1 |
| **GO:0051289*** | protein homotetramerization | 3 | 78 | 0.0016 | TP63, SHMT1, ACACB |
| **GO:2000027** | regulation of animal organ morphogenesis | 4 | 207 | 0.0016 | SOX9, GRHL3, TBX1, MSX1 |
| **GO:0050667*** | homocysteine metabolic process | 2 | 13 | 0.0018 | MTHFD1, MTHFR |
| **GO:0001843** | neural tube closure | 3 | 86 | 0.0020 | MTHFD1, LRP6, GRHL3 |
| **GO:0009719** | response to endogenous stimulus | 8 | 1353 | 0.0020 | SOX9, LRP6, CDH1, TYMS, SHMT1, TBX1, COL2A1, MSX1 |
| **GO:0035112** | genitalia morphogenesis | 2 | 14 | 0.0020 | LRP6, TP63 |
| **GO:0060322** | head development | 6 | 692 | 0.0020 | SOX9, LRP6, CDH1, TBX1, COL2A1, MSX1 |
| **GO:0060349** | bone morphogenesis | 3 | 86 | 0.0020 | SOX9, COL2A1, MSX1 |
| **GO:0071599** | otic vesicle development | 2 | 14 | 0.0020 | SOX9, COL2A1 |
| **GO:1901698** | response to nitrogen compound | 7 | 988 | 0.0020 | SOX9, LRP6, CDH1, TYMS, SHMT1, MTR, MTHFR |
| **GO:2000113** | negative regulation of cellular macromolecule biosynthetic process | 8 | 1348 | 0.0020 | SOX9, CREBBP, TP63, TYMS, SHMT1, TBX22, RFC1, MSX1 |
| **GO:0002065** | columnar/cuboidal epithelial cell differentiation | 3 | 91 | 0.0022 | TP63, TYMS, JAG2 |
| **GO:0043933** | protein-containing complex subunit organization | 9 | 1770 | 0.0022 | SOX9, LRP6, CREBBP, TP63, SHMT1, ACACB, COL11A1, COL11A2, COL2A1 |
| **GO:0009889*** | regulation of biosynthetic process | 14 | 4337 | 0.0024 | SOX9, LRP6, CDH1, CREBBP, TP63, GRHL3, TYMS, SHMT1, TBX1, ACACB, TBX22, TCOF1, RFC1, MSX1 |
| **GO:0009070** | serine family amino acid biosynthetic process | 2 | 17 | 0.0025 | MTHFD1, SHMT1 |
| **GO:0043603*** | cellular amide metabolic process | 6 | 732 | 0.0025 | MTHFD1, FTCD, TYMS, SHMT1, ACACB, MTHFR |
| **GO:1901700** | response to oxygen-containing compound | 8 | 1427 | 0.0026 | SOX9, LRP6, CDH1, TYMS, SHMT1, TBX1, MTR, MTHFR |
| **GO:0030903*** | notochord development | 2 | 18 | 0.0027 | SOX9, COL2A1 |
| **GO:0048745** | smooth muscle tissue development | 2 | 19 | 0.0029 | SOX9, TP63 |
| **GO:0061436*** | establishment of skin barrier | 2 | 19 | 0.0029 | TP63, GRHL3 |
| **GO:0042474*** | middle ear morphogenesis | 2 | 20 | 0.0031 | TBX1, MSX1 |
| **GO:0045944** | positive regulation of transcription by RNA polymerase II | 7 | 1104 | 0.0032 | SOX9, LRP6, CREBBP, TP63, GRHL3, TBX1, MSX1 |
| **GO:0071773** | cellular response to BMP stimulus | 3 | 107 | 0.0032 | SOX9, COL2A1, MSX1 |
| **GO:0001736** | establishment of planar polarity | 2 | 21 | 0.0033 | TP63, GRHL3 |
| **GO:0009235*** | cobalamin metabolic process | 2 | 21 | 0.0033 | TCN2, MTR |
| **GO:0048468** | cell development | 8 | 1493 | 0.0033 | SOX9, CDH1, TP63, TYMS, TBX1, MTR, COL11A1, TCOF1 |
| **GO:0016043** | cellular component organization | 15 | 5163 | 0.0034 | SOX9, LRP6, CDH1, CREBBP, TP63, SHMT1, ACACB, OFD1, MTR, COL11A1, COL11A2, MTHFR, COL2A1, RFC1, MSX1 |
| **GO:0002063*** | chondrocyte development | 2 | 22 | 0.0035 | SOX9, COL11A1 |
| **GO:0060740** | prostate gland epithelium morphogenesis | 2 | 22 | 0.0035 | SOX9, TP63 |
| **GO:0044249** | cellular biosynthetic process | 14 | 4567 | 0.0036 | MTHFD1, SOX9, CREBBP, TP63, GRHL3, TYMS, SHMT1, TBX1, ACACB, POMGNT2, MTR, TBX22, RFC1, MSX1 |
| **GO:0000122** | negative regulation of transcription by RNA polymerase II | 6 | 809 | 0.0037 | SOX9, CREBBP, TP63, TBX22, RFC1, MSX1 |
| **GO:0060351*** | cartilage development involved in endochondral bone morphogenesis | 2 | 23 | 0.0037 | SOX9, COL2A1 |
| **GO:0009165*** | nucleotide biosynthetic process | 4 | 291 | 0.0040 | MTHFD1, TYMS, SHMT1, ACACB |
| **GO:0031324** | negative regulation of cellular metabolic process | 10 | 2463 | 0.0042 | SOX9, LRP6, CREBBP, TP63, TYMS, SHMT1, ACACB, TBX22, RFC1, MSX1 |
| **GO:0032365*** | intracellular lipid transport | 2 | 25 | 0.0042 | LRP6, ACACB |
| **GO:1901576** | organic substance biosynthetic process | 14 | 4656 | 0.0042 | MTHFD1, SOX9, CREBBP, TP63, GRHL3, TYMS, SHMT1, TBX1, ACACB, POMGNT2, MTR, TBX22, RFC1, MSX1 |
| **GO:2000112** | regulation of cellular macromolecule biosynthetic process | 13 | 4050 | 0.0042 | SOX9, LRP6, CDH1, CREBBP, TP63, GRHL3, TYMS, SHMT1, TBX1, TBX22, TCOF1, RFC1, MSX1 |
| **GO:0030326** | embryonic limb morphogenesis | 3 | 126 | 0.0044 | CREBBP, TP63, MSX1 |
| **GO:0003401*** | axis elongation | 2 | 27 | 0.0047 | SOX9, LRP6 |
| **GO:0035690** | cellular response to drug | 4 | 310 | 0.0047 | SOX9, CDH1, MTR, MSX1 |
| **GO:0043066** | negative regulation of apoptotic process | 6 | 859 | 0.0047 | SOX9, LRP6, TP63, TBX1, COL2A1, MSX1 |
| **GO:0046394*** | carboxylic acid biosynthetic process | 4 | 311 | 0.0047 | MTHFD1, SHMT1, ACACB, MTR |
| **GO:0003002** | regionalization | 4 | 313 | 0.0048 | LRP6, TP63, TBX1, MSX1 |
| **GO:0035116** | embryonic hindlimb morphogenesis | 2 | 28 | 0.0048 | TP63, MSX1 |
| **GO:0010243** | response to organonitrogen compound | 6 | 876 | 0.0049 | SOX9, LRP6, CDH1, TYMS, SHMT1, MTHFR |
| **GO:0014070** | response to organic cyclic compound | 6 | 873 | 0.0049 | LRP6, CDH1, TYMS, SHMT1, ACACB, MTHFR |
| **GO:0042221** | response to chemical | 13 | 4153 | 0.0049 | TCN2, SOX9, LRP6, CDH1, CREBBP, TYMS, SHMT1, TBX1, ACACB, MTR, MTHFR, COL2A1, MSX1 |
| **GO:0060255** | regulation of macromolecule metabolic process | 16 | 6072 | 0.0049 | SOX9, LRP6, CDH1, CREBBP, TP63, GRHL3, TYMS, SHMT1, TBX1, ACACB, TBX22, MTHFR, TCOF1, COL2A1, RFC1, MSX1 |
| **GO:0009987** | cellular process | 25 | 14652 | 0.0051 | TCN2, MTHFD1, SOX9, LRP6, CDH1, CREBBP, TP63, GRHL3, FTCD, TYMS, SHMT1, JAG2, TBX1, ACACB, POMGNT2, OFD1, MTR, COL11A1, TBX22, COL11A2, MTHFR, TCOF1, COL2A1, RFC1, MSX1 |
| **GO:0010605** | negative regulation of macromolecule metabolic process | 10 | 2558 | 0.0052 | SOX9, LRP6, CREBBP, TP63, TYMS, SHMT1, ACACB, TBX22, RFC1, MSX1 |
| **GO:0006807** | nitrogen compound metabolic process | 19 | 8349 | 0.0053 | TCN2, MTHFD1, SOX9, CREBBP, TP63, GRHL3, FTCD, TYMS, SHMT1, TBX1, ACACB, POMGNT2, MTR, COL11A1, TBX22, MTHFR, COL2A1, RFC1, MSX1 |
| **GO:1901701** | cellular response to oxygen-containing compound | 6 | 896 | 0.0054 | SOX9, LRP6, CDH1, SHMT1, TBX1, MTR |
| **GO:0035115** | embryonic forelimb morphogenesis | 2 | 31 | 0.0056 | TP63, MSX1 |
| **GO:0003203*** | endocardial cushion morphogenesis | 2 | 32 | 0.0059 | SOX9, MSX1 |
| **GO:0006790*** | sulfur compound metabolic process | 4 | 343 | 0.0062 | MTHFD1, ACACB, MTR, MTHFR |
| **GO:0050896** | response to stimulus | 18 | 7824 | 0.0080 | TCN2, SOX9, LRP6, CDH1, CREBBP, TP63, GRHL3, TYMS, SHMT1, JAG2, TBX1, ACACB, MTR, COL11A1, MTHFR, COL2A1, RFC1, MSX1 |
| **GO:0030855** | epithelial cell differentiation | 5 | 649 | 0.0081 | SOX9, TP63, TYMS, JAG2, TBX1 |
| **GO:0030857** | negative regulation of epithelial cell differentiation | 2 | 39 | 0.0081 | SOX9, TP63 |
| **GO:0044281*** | small molecule metabolic process | 8 | 1779 | 0.0081 | TCN2, MTHFD1, FTCD, TYMS, SHMT1, ACACB, MTR, MTHFR |
| **GO:0042398*** | cellular modified amino acid biosynthetic process | 2 | 41 | 0.0087 | MTHFD1, SHMT1 |
| **GO:0048523** | negative regulation of cellular process | 13 | 4454 | 0.0087 | SOX9, LRP6, CDH1, CREBBP, TP63, TYMS, SHMT1, TBX1, ACACB, TBX22, COL2A1, RFC1, MSX1 |
| **GO:0044272*** | sulfur compound biosynthetic process | 3 | 172 | 0.0088 | MTHFD1, ACACB, MTR |
| **GO:0008283** | cell population proliferation | 5 | 676 | 0.0093 | SOX9, LRP6, TP63, TBX1, MSX1 |
| **GO:0021983*** | pituitary gland development | 2 | 43 | 0.0093 | CDH1, MSX1 |
| **GO:0048483*** | autonomic nervous system development | 2 | 43 | 0.0093 | TP63, TBX1 |
| **GO:0034641** | cellular nitrogen compound metabolic process | 14 | 5126 | 0.0094 | MTHFD1, SOX9, CREBBP, TP63, GRHL3, FTCD, TYMS, SHMT1, TBX1, ACACB, TBX22, MTHFR, RFC1, MSX1 |
| **GO:0002064** | epithelial cell development | 3 | 179 | 0.0095 | SOX9, TP63, TYMS |
| **GO:0010033** | response to organic substance | 10 | 2815 | 0.0095 | SOX9, LRP6, CDH1, TYMS, SHMT1, TBX1, ACACB, MTHFR, COL2A1, MSX1 |
| **GO:0050679** | positive regulation of epithelial cell proliferation | 3 | 178 | 0.0095 | SOX9, TP63, TBX1 |
| **GO:0051172** | negative regulation of nitrogen compound metabolic process | 9 | 2307 | 0.0095 | SOX9, LRP6, CREBBP, TP63, TYMS, SHMT1, TBX22, RFC1, MSX1 |
| **GO:0008544** | epidermis development | 4 | 403 | 0.0099 | SOX9, TP63, GRHL3, JAG2 |
| **GO:0044237** | cellular metabolic process | 19 | 8797 | 0.0099 | TCN2, MTHFD1, SOX9, CREBBP, TP63, GRHL3, FTCD, TYMS, SHMT1, TBX1, ACACB, POMGNT2, MTR, COL11A1, TBX22, MTHFR, COL2A1, RFC1, MSX1 |
| **GO:0030901*** | midbrain development | 2 | 46 | 0.0102 | LRP6, MSX1 |
| **GO:2000026** | regulation of multicellular organismal development | 8 | 1876 | 0.0104 | SOX9, LRP6, CREBBP, TP63, GRHL3, TBX1, ACACB, MSX1 |
| **GO:0060042*** | retina morphogenesis in camera-type eye | 2 | 49 | 0.0112 | SOX9, LRP6 |
| **GO:0071229** | cellular response to acid chemical | 3 | 196 | 0.0117 | SOX9, SHMT1, TBX1 |
| **GO:0009952** | anterior/posterior pattern specification | 3 | 197 | 0.0118 | LRP6, TBX1, MSX1 |
| **GO:1901135** | carbohydrate derivative metabolic process | 6 | 1083 | 0.0119 | TYMS, SHMT1, ACACB, POMGNT2, COL11A1, COL2A1 |
| **GO:0009967** | positive regulation of signal transduction | 7 | 1493 | 0.0123 | SOX9, LRP6, CREBBP, TP63, JAG2, TBX1, MSX1 |
| **GO:0042981** | regulation of apoptotic process | 7 | 1501 | 0.0126 | SOX9, LRP6, CREBBP, TP63, TBX1, COL2A1, MSX1 |
| **GO:0080090** | regulation of primary metabolic process | 15 | 5982 | 0.0126 | SOX9, LRP6, CDH1, CREBBP, TP63, GRHL3, TYMS, SHMT1, TBX1, ACACB, TBX22, MTHFR, TCOF1, RFC1, MSX1 |
| **GO:0071495** | cellular response to endogenous stimulus | 6 | 1106 | 0.0131 | SOX9, CDH1, SHMT1, TBX1, COL2A1, MSX1 |
| **GO:0072522*** | purine-containing compound biosynthetic process | 3 | 206 | 0.0131 | MTHFD1, SHMT1, ACACB |
| **GO:0061061** | muscle structure development | 4 | 457 | 0.0144 | SOX9, TBX1, COL11A1, MSX1 |
| **GO:0065008** | regulation of biological quality | 11 | 3559 | 0.0144 | MTHFD1, SOX9, LRP6, CREBBP, TP63, GRHL3, SHMT1, ACACB, COL2A1, RFC1, MSX1 |
| **GO:0001503** | ossification | 3 | 215 | 0.0145 | SOX9, COL11A1, COL2A1 |
| **GO:0031323** | regulation of cellular metabolic process | 15 | 6082 | 0.0147 | SOX9, LRP6, CDH1, CREBBP, TP63, GRHL3, TYMS, SHMT1, TBX1, ACACB, TBX22, MTHFR, TCOF1, RFC1, MSX1 |
| **GO:0006366** | transcription by RNA polymerase II | 5 | 784 | 0.0155 | SOX9, CREBBP, TP63, GRHL3, MSX1 |
| **GO:0071704** | organic substance metabolic process | 19 | 9135 | 0.0155 | TCN2, MTHFD1, SOX9, CREBBP, TP63, GRHL3, FTCD, TYMS, SHMT1, TBX1, ACACB, POMGNT2, MTR, COL11A1, TBX22, MTHFR, COL2A1, RFC1, MSX1 |
| **GO:0031325** | positive regulation of cellular metabolic process | 10 | 3060 | 0.0162 | SOX9, LRP6, CDH1, CREBBP, TP63, GRHL3, TBX1, ACACB, RFC1, MSX1 |
| **GO:0071363** | cellular response to growth factor stimulus | 4 | 477 | 0.0163 | SOX9, TBX1, COL2A1, MSX1 |
| **GO:0045165** | cell fate commitment | 3 | 230 | 0.0169 | SOX9, JAG2, TBX1 |
| **GO:0042733** | embryonic digit morphogenesis | 2 | 64 | 0.0171 | CREBBP, MSX1 |
| **GO:0006355** | regulation of transcription, DNA-templated | 11 | 3661 | 0.0174 | SOX9, LRP6, CDH1, CREBBP, TP63, GRHL3, TYMS, TBX1, TBX22, RFC1, MSX1 |
| **GO:0097305*** | response to alcohol | 3 | 233 | 0.0174 | LRP6, CDH1, TYMS |
| **GO:0071300*** | cellular response to retinoic acid | 2 | 68 | 0.0187 | SOX9, TBX1 |
| **GO:2000243*** | positive regulation of reproductive process | 2 | 70 | 0.0194 | SOX9, MSX1 |
| **GO:0006357** | regulation of transcription by RNA polymerase II | 9 | 2633 | 0.0197 | SOX9, LRP6, CREBBP, TP63, GRHL3, TBX1, TBX22, RFC1, MSX1 |
| **GO:0001708** | cell fate specification | 2 | 72 | 0.0203 | SOX9, TBX1 |
| **GO:0001942** | hair follicle development | 2 | 72 | 0.0203 | SOX9, TP63 |
| **GO:0007548** | sex differentiation | 3 | 252 | 0.0205 | SOX9, LRP6, TP63 |
| **GO:0060415*** | muscle tissue morphogenesis | 2 | 74 | 0.0212 | TBX1, COL11A1 |
| **GO:0070887** | cellular response to chemical stimulus | 9 | 2672 | 0.0214 | SOX9, LRP6, CDH1, CREBBP, SHMT1, TBX1, MTR, COL2A1, MSX1 |
| **GO:0034644*** | cellular response to UV | 2 | 78 | 0.0230 | CREBBP, TP63 |
| **GO:0061053** | somite development | 2 | 79 | 0.0235 | MTHFD1, LRP6 |
| **GO:0006029*** | proteoglycan metabolic process | 2 | 82 | 0.0250 | COL11A1, COL2A1 |
| **GO:0050673*** | epithelial cell proliferation | 2 | 83 | 0.0254 | SOX9, TP63 |
| **GO:0097306** | cellular response to alcohol | 2 | 83 | 0.0254 | LRP6, CDH1 |
| **GO:0071214** | cellular response to abiotic stimulus | 3 | 282 | 0.0266 | SOX9, CREBBP, TP63 |
| **GO:0051171** | regulation of nitrogen compound metabolic process | 14 | 5827 | 0.0267 | SOX9, LRP6, CDH1, CREBBP, TP63, GRHL3, TYMS, SHMT1, TBX1, TBX22, MTHFR, TCOF1, RFC1, MSX1 |
| **GO:0060537** | muscle tissue development | 3 | 284 | 0.0267 | SOX9, TP63, COL11A1 |
| **GO:1901699** | cellular response to nitrogen compound | 4 | 568 | 0.0267 | SOX9, CDH1, SHMT1, MTR |
| **GO:0060249** | anatomical structure homeostasis | 3 | 285 | 0.0269 | SOX9, COL2A1, RFC1 |
| **GO:0007517** | muscle organ development | 3 | 287 | 0.0272 | TBX1, COL11A1, MSX1 |
| **GO:0044238** | primary metabolic process | 18 | 8808 | 0.0275 | MTHFD1, SOX9, CREBBP, TP63, GRHL3, FTCD, TYMS, SHMT1, TBX1, ACACB, POMGNT2, MTR, COL11A1, TBX22, MTHFR, COL2A1, RFC1, MSX1 |
| **GO:0043010** | camera-type eye development | 3 | 292 | 0.0279 | SOX9, LRP6, GRHL3 |
| **GO:0030182** | neuron differentiation | 5 | 940 | 0.0281 | SOX9, LRP6, CDH1, JAG2, MTR |
| **GO:0003008*** | system process | 7 | 1827 | 0.0301 | SOX9, JAG2, TBX1, COL11A1, COL11A2, MTHFR, COL2A1 |
| **GO:1901566** | organonitrogen compound biosynthetic process | 6 | 1370 | 0.0301 | MTHFD1, TYMS, SHMT1, ACACB, POMGNT2, MTR |
| **GO:0071897*** | DNA biosynthetic process | 2 | 95 | 0.0305 | TYMS, RFC1 |
| **GO:0051153*** | regulation of striated muscle cell differentiation | 2 | 96 | 0.0310 | TBX1, MSX1 |
| **GO:1901564** | organonitrogen compound metabolic process | 13 | 5281 | 0.0310 | TCN2, MTHFD1, SOX9, CREBBP, FTCD, TYMS, SHMT1, ACACB, POMGNT2, MTR, COL11A1, MTHFR, COL2A1 |
| **GO:1901606*** | alpha-amino acid catabolic process | 2 | 98 | 0.0320 | FTCD, SHMT1 |
| **GO:1901137** | carbohydrate derivative biosynthetic process | 4 | 625 | 0.0345 | TYMS, SHMT1, ACACB, POMGNT2 |
| **GO:0048699** | generation of neurons | 6 | 1422 | 0.0348 | SOX9, LRP6, CDH1, JAG2, POMGNT2, MTR |
| **GO:0051276** | chromosome organization | 5 | 999 | 0.0348 | SOX9, CREBBP, TP63, MTHFR, RFC1 |
| **GO:0006417*** | regulation of translation | 3 | 327 | 0.0361 | TYMS, SHMT1, TCOF1 |
| **GO:0034645** | cellular macromolecule biosynthetic process | 10 | 3518 | 0.0361 | SOX9, CREBBP, TP63, GRHL3, TYMS, TBX1, POMGNT2, TBX22, RFC1, MSX1 |
| **GO:0048589** | developmental growth | 3 | 340 | 0.0393 | SOX9, LRP6, TYMS |
| **GO:0007368** | determination of left/right symmetry | 2 | 115 | 0.0408 | TBX1, OFD1 |
| **GO:0007498*** | mesoderm development | 2 | 115 | 0.0408 | TP63, TBX1 |
| **GO:0009628** | response to abiotic stimulus | 5 | 1052 | 0.0408 | SOX9, CREBBP, TP63, COL11A1, MTHFR |
| **GO:0009100** | glycoprotein metabolic process | 3 | 349 | 0.0412 | POMGNT2, COL11A1, COL2A1 |
| **GO:0017148*** | negative regulation of translation | 2 | 117 | 0.0417 | TYMS, SHMT1 |
| **GO:0006325** | chromatin organization | 4 | 683 | 0.0436 | SOX9, CREBBP, TP63, MTHFR |
| **GO:0010564** | regulation of cell cycle process | 4 | 684 | 0.0436 | SOX9, TP63, OFD1, MSX1 |
| **GO:0045596** | negative regulation of cell differentiation | 4 | 683 | 0.0436 | SOX9, TP63, TBX1, MSX1 |
| **GO:0001889** | liver development | 2 | 123 | 0.0448 | SOX9, TYMS |
| **GO:0030879** | mammary gland development | 2 | 123 | 0.0448 | SOX9, MSX1 |
| **GO:0090090** | negative regulation of canonical Wnt signaling pathway | 2 | 123 | 0.0448 | SOX9, LRP6 |
| **GO:0048565** | digestive tract development | 2 | 126 | 0.0463 | TP63, TYMS |
| **GO:0043588** | skin development | 3 | 373 | 0.0472 | SOX9, TP63, GRHL3 |
| **GO:0003206** | cardiac chamber morphogenesis | 2 | 128 | 0.0474 | TBX1, COL11A1 |
| **GO:0051240** | positive regulation of multicellular organismal process | 6 | 1551 | 0.0474 | SOX9, LRP6, CREBBP, TP63, TBX1, ACACB |
| **GO:0006351** | transcription, DNA-templated | 8 | 2569 | 0.0478 | SOX9, CREBBP, TP63, GRHL3, TBX1, TBX22, RFC1, MSX1 |
| **GO:0045787** | positive regulation of cell cycle | 3 | 376 | 0.0478 | LRP6, TP63, MSX1 |
| **GO:1901796** | regulation of signal transduction by p53 class mediator | 2 | 129 | 0.0478 | TP63, MSX1 |
| **GO:1905330** | regulation of morphogenesis of an epithelium | 2 | 130 | 0.0481 | SOX9, GRHL3 |

*Biological process found only in NSCPO.
