# Supplementary material for: Identification of Novel Variants in Cleft Palate-Associated Genes in Brazilian Patients With Non-syndromic Cleft Palate Only
Source: Front Cell Dev Biol. 2021 Jul 8;9:638522. doi: 10.3389/fcell.2021.638522 (PMC8297955; doi:10.3389/fcell.2021.638522)
Supplement: Supplementary file 8 [file Data_Sheet_6.docx]

| **Supplementary Table 6.** Biological processes characterized with the list of altered genes in patients with nonsyndromic cleft palate only (NSCPO) from syndromic cleft palate-associated genes. | | | | | |
| --- | --- | --- | --- | --- | --- |
| **GO ID** | **Term description** | **Observed gene count** | **Background gene count** | **False discovery rate** | **Matching proteins in your network** |
| **GO:0032502** | developmental process | 65 | 5401 | 6.42e-12 | CD4,KCNQ1,WFS1,GMNN,SLC35D1,CHSY1,NOTCH2,BRIP1,NOTCH3,STAT3,HYAL1,GATA6,DUSP6,GDF6,FANCD2,BRAF,TBC1D24,HPGD,BMPER,ANKRD11,KMT2D,COL3A1,UBB,PLXND1,CEP57,FAM20C,POLE,RAI1,WT1,COL7A1,ALX4,RERE,PTPN11,COL9A3,ATR,POMGNT2,ARID1B,COMT,SLC39A13,SMC3,DMD,LMNA,L1CAM,SOX3,NSMF,BMPR1A,COL9A2,ATRX,MED12,HSPG2,COL5A2,LRP4,CDKL5,FANCA,GPC3,GLI3,FOXP2,SMARCA4,TRPV4,FTO,FBN2,ZEB2,STRA6,RECQL4,NEB |
| **GO:0048856** | anatomical structure development | 63 | 5085 | 6.42e-12 | CD4,KCNQ1,WFS1,GMNN,SLC35D1,CHSY1,NOTCH2,BRIP1,NOTCH3,STAT3,HYAL1,GATA6,DUSP6,GDF6,FANCD2,BRAF,TBC1D24,HPGD,BMPER,ANKRD11,KMT2D,COL3A1,UBB,PLXND1,CEP57,FAM20C,POLE,RAI1,WT1,COL7A1,ALX4,RERE,PTPN11,COL9A3,ATR,POMGNT2,ARID1B,SLC39A13,DMD,LMNA,L1CAM,SOX3,NSMF,BMPR1A,COL9A2,ATRX,MED12,HSPG2,COL5A2,LRP4,CDKL5,FANCA,GPC3,GLI3,FOXP2,SMARCA4,TRPV4,FTO,FBN2,ZEB2,STRA6,RECQL4,NEB |
| **GO:0007275** | multicellular organism development | 60 | 4726 | 8.60e-12 | CD4,KCNQ1,WFS1,GMNN,SLC35D1,CHSY1,NOTCH2,BRIP1,NOTCH3,STAT3,HYAL1,GATA6,DUSP6,GDF6,FANCD2,BRAF,TBC1D24,HPGD,BMPER,ANKRD11,COL3A1,UBB,PLXND1,FAM20C,POLE,RAI1,WT1,COL7A1,ALX4,RERE,PTPN11,COL9A3,ATR,POMGNT2,ARID1B,DMD,LMNA,L1CAM,SOX3,NSMF,BMPR1A,COL9A2,ATRX,MED12,HSPG2,COL5A2,LRP4,CDKL5,FANCA,GPC3,GLI3,FOXP2,SMARCA4,TRPV4,FTO,FBN2,ZEB2,STRA6,RECQL4,NEB |
| **GO:0048731** | system development | 56 | 4144 | 8.80e-12 | CD4,KCNQ1,WFS1,GMNN,SLC35D1,CHSY1,NOTCH2,BRIP1,NOTCH3,STAT3,HYAL1,GATA6,GDF6,FANCD2,BRAF,TBC1D24,HPGD,BMPER,ANKRD11,COL3A1,UBB,PLXND1,FAM20C,POLE,RAI1,WT1,ALX4,RERE,PTPN11,COL9A3,POMGNT2,ARID1B,DMD,LMNA,L1CAM,SOX3,NSMF,BMPR1A,COL9A2,ATRX,MED12,HSPG2,COL5A2,LRP4,CDKL5,FANCA,GPC3,GLI3,FOXP2,SMARCA4,TRPV4,FTO,FBN2,ZEB2,STRA6,NEB |
| **GO:0048513** | animal organ development | 45 | 2926 | 2.32e-10 | CD4,KCNQ1,WFS1,GMNN,CHSY1,NOTCH2,BRIP1,NOTCH3,STAT3,HYAL1,GATA6,FANCD2,BRAF,HPGD,BMPER,ANKRD11,COL3A1,PLXND1,FAM20C,POLE,WT1,ALX4,RERE,PTPN11,COL9A3,DMD,LMNA,SOX3,BMPR1A,ATRX,MED12,HSPG2,COL5A2,LRP4,FANCA,GPC3,GLI3,FOXP2,SMARCA4,TRPV4,FTO,FBN2,ZEB2,STRA6,NEB |
| **GO:0009653** | anatomical structure morphogenesis | 35 | 1992 | 7.51e-09 | GMNN,CHSY1,NOTCH2,NOTCH3,STAT3,HYAL1,GATA6,FANCD2,BRAF,HPGD,BMPER,ANKRD11,COL3A1,UBB,PLXND1,FAM20C,WT1,COL7A1,ALX4,RERE,PTPN11,L1CAM,BMPR1A,ATRX,MED12,HSPG2,COL5A2,LRP4,GPC3,GLI3,FOXP2,TRPV4,FBN2,ZEB2,STRA6 |
| **GO:0032501** | multicellular organismal process | 65 | 6507 | 1.15e-08 | CD4,KCNQ1,WFS1,GMNN,SLC35D1,CHSY1,NOTCH2,BRIP1,NOTCH3,STAT3,HYAL1,GATA6,DUSP6,GDF6,FANCD2,BRAF,TBC1D24,HPGD,BMPER,ANKRD11,KMT2D,COL3A1,UBB,PLXND1,CEP57,FAM20C,POLE,RAI1,WT1,COL7A1,ALX4,RERE,PTPN11,COL9A3,ATR,POMGNT2,ARID1B,COMT,SMC3,DMD,LMNA,L1CAM,SOX3,NSMF,BMPR1A,COL9A2,ATRX,MED12,HSPG2,COL5A2,LRP4,CDKL5,FANCA,GPC3,GLI3,FOXP2,SMARCA4,TRPV4,FTO,PIEZO2,FBN2,ZEB2,STRA6,RECQL4,NEB |
| **GO:0051239** | regulation of multicellular organismal process | 41 | 2788 | 1.26e-08 | CD4,KCNQ1,POLR3B,CHSY1,NOTCH2,NOTCH3,STAT3,HYAL1,GATA6,DUSP6,GDF6,FANCD2,BMPER,KMT2D,COL3A1,PLXND1,FAM20C,RAI1,WT1,PTPN11,COMT,DMD,LMNA,L1CAM,SOX3,NSMF,BMPR1A,POLR3A,MED12,HSPG2,COL5A2,LRP4,CDKL5,FANCA,GPC3,GLI3,FOXP2,TRPV4,FTO,FBN2,ZEB2 |
| **GO:0009887** | animal organ morphogenesis | 23 | 865 | 1.71e-08 | GMNN,CHSY1,NOTCH2,STAT3,HYAL1,GATA6,FANCD2,BRAF,ANKRD11,COL3A1,PLXND1,FAM20C,WT1,ALX4,BMPR1A,MED12,COL5A2,LRP4,GPC3,GLI3,TRPV4,FBN2,STRA6 |
| **GO:0048518** | positive regulation of biological process | 58 | 5459 | 3.31e-08 | CD4,KCNQ1,CDC6,WFS1,POLR3B,CHSY1,NOTCH2,MAP2K2,NOTCH3,STAT3,B3GAT3,HYAL1,GATA6,DUSP6,GDF6,KAT6B,BRAF,HPGD,BMPER,RAD21,CDT1,KMT2D,COL3A1,UBB,PLXND1,FAM20C,RAI1,WT1,ALX4,RERE,PTPN11,ATR,DOK7,ARID1B,POX2,ACTB,COMT,SMC3,DMD,LMNA,L1CAM,NSMF,BMPR1A,POLR3A,POLR1C,ATRX,MED12,LRP4,CDKL5,GPC3,GLI3,FOXP2,SEPT9,SMARCA4,TRPV4,FBN2,ZEB2,STRA6 |
| **GO:2000026** | regulation of multicellular organismal development | 32 | 1876 | 9.04e-08 | CD4,NOTCH2,NOTCH3,STAT3,HYAL1,GATA6,DUSP6,GDF6,FANCD2,BMPER,KMT2D,COL3A1,PLXND1,FAM20C,WT1,DMD,L1CAM,SOX3,NSMF,BMPR1A,MED12,HSPG2,COL5A2,LRP4,CDKL5,FANCA,GPC3,GLI3,FOXP2,TRPV4,FBN2,ZEB2 |
| **GO:0050793** | regulation of developmental process | 36 | 2416 | 1.71e-07 | CD4,NOTCH2,NOTCH3,STAT3,HYAL1,GATA6,DUSP6,GDF6,FANCD2,BMPER,KMT2D,COL3A1,PLXND1,FAM20C,RAI1,WT1,PTPN11,DMD,LMNA,L1CAM,SOX3,NSMF,BMPR1A,MED12,HSPG2,COL5A2,LRP4,CDKL5,FANCA,GPC3,GLI3,FOXP2,TRPV4,FTO,FBN2,ZEB2 |
| **GO:0001501** | skeletal system development | 16 | 457 | 3.53e-07 | SLC35D1,CHSY1,HYAL1,ANKRD11,COL3A1,FAM20C,RAI1,ALX4,PTPN11,BMPR1A,COL9A2,MED12,COL5A2,GLI3,TRPV4,FBN2 |
| **GO:0048519** | negative regulation of biological process | 52 | 4953 | 1.00e-06 | KCNQ1,CDC6,WFS1,GMNN,CHSY1,NOTCH2,BRIP1,MAP2K2,NOTCH3,STAT3,HYAL1,GATA6,DUSP6,KAT6B,BUB1B,BRAF,HPGD,BMPER,RAD21,CDT1,KMT2D,COL3A1,UBB,PLXND1,SHMT1,RAI1,WT1,COL7A1,RERE,PTPN11,ATR,TTC37,COMT,SMC3,DMD,LMNA,SOX3,BMPR1A,ATRX,MED12,HSPG2,COL5A2,LRP4,GPC3,GLI3,FOXP2,TMEM67,PEX5,SMARCA4,TRPV4,FBN2,ZEB2 |
| **GO:0009719** | response to endogenous stimulus | 25 | 1353 | 2.07e-06 | CD4,KCNQ1,CDC6,BRIP1,LTBP2,STAT3,HYAL1,GATA6,GDF6,HPGD,COL3A1,UBB,CEP57,SHMT1,WT1,PTPN11,ARID1B,ACTB,NSMF,BMPR1A,MED12,COL5A2,LRP4,FOXP2,TRPV4 |
| **GO:0010604** | positive regulation of macromolecule metabolic process | 39 | 3081 | 2.07e-06 | CD4,CDC6,WFS1,NOTCH2,MAP2K2,NOTCH3,STAT3,GATA6,DUSP6,GDF6,KAT6B,BRAF,BMPER,RAD21,CDT1,KMT2D,UBB,PLXND1,RAI1,WT1,ALX4,RERE,PTPN11,ATR,DOK7,ARID1B,ACTB,LMNA,NSMF,BMPR1A,POLR1C,ATRX,MED12,LRP4,GPC3,GLI3,SMARCA4,TRPV4,ZEB2 |
| **GO:0048522** | positive regulation of cellular process | 51 | 4898 | 2.07e-06 | CD4,KCNQ1,CDC6,WFS1,CHSY1,NOTCH2,MAP2K2,NOTCH3,STAT3,HYAL1,GATA6,DUSP6,GDF6,KAT6B,BRAF,HPGD,BMPER,RAD21,CDT1,KMT2D,COL3A1,UBB,PLXND1,FAM20C,RAI1,WT1,ALX4,RERE,PTPN11,ATR,DOK7,ARID1B,POX2,COMT,DMD,LMNA,L1CAM,NSMF,BMPR1A,ATRX,MED12,LRP4,CDKL5,GPC3,GLI3,FOXP2,SEPT9,SMARCA4,TRPV4,FBN2,ZEB2 |
| **GO:0048523** | negative regulation of cellular process | 48 | 4454 | 2.29e-06 | KCNQ1,CDC6,WFS1,GMNN,NOTCH2,BRIP1,NOTCH3,STAT3,HYAL1,GATA6,DUSP6,KAT6B,BUB1B,BRAF,HPGD,BMPER,RAD21,CDT1,KMT2D,COL3A1,UBB,PLXND1,SHMT1,WT1,COL7A1,RERE,PTPN11,ATR,COMT,SMC3,DMD,LMNA,SOX3,BMPR1A,ATRX,MED12,HSPG2,COL5A2,LRP4,GPC3,GLI3,FOXP2,TMEM67,PEX5,SMARCA4,TRPV4,FBN2,ZEB2 |
| **GO:0009893** | positive regulation of metabolic process | 40 | 3280 | 2.74e-06 | CD4,CDC6,WFS1,NOTCH2,MAP2K2,NOTCH3,STAT3,GATA6,DUSP6,GDF6,KAT6B,BRAF,BMPER,RAD21,CDT1,KMT2D,UBB,PLXND1,RAI1,WT1,ALX4,RERE,PTPN11,ATR,DOK7,ARID1B,ACTB,COMT,LMNA,NSMF,BMPR1A,POLR1C,ATRX,MED12,LRP4,GPC3,GLI3,SMARCA4,TRPV4,ZEB2 |
| **GO:0044260** | cellular macromolecule metabolic process | 59 | 6413 | 3.94e-06 | CDC6,WFS1,POLR3B,GMNN,SLC35D1,CHSY1,NOTCH2,BRIP1,XYLT1,MAP2K2,NOTCH3,STAT3,B3GAT3,HYAL1,GATA6,DUSP6,MED13L,GDF6,KAT6B,BUB1B,FANCD2,BRAF,RAD21,CDT1,KMT2D,COL3A1,UBB,FAM20C,POLE,WT1,ALX4,RERE,PTPN11,ATR,POMGNT2,ARID1B,ACTB,TTC37,SMC3,SOX3,POMGNT1,BMPR1A,POLR3A,POLR1C,ATRX,MED12,HSPG2,CDKL5,FANCA,GPC3,GLI3,FOXP2,TMEM67,PEX5,EARS2,SMARCA4,FTO,ZEB2,RECQL4 |
| **GO:0071495** | cellular response to endogenous stimulus | 22 | 1106 | 3.94e-06 | KCNQ1,CDC6,BRIP1,LTBP2,STAT3,HYAL1,GATA6,GDF6,HPGD,COL3A1,UBB,CEP57,SHMT1,WT1,PTPN11,ARID1B,ACTB,NSMF,BMPR1A,MED12,COL5A2,LRP4 |
| **GO:0051173** | positive regulation of nitrogen compound metabolic process | 37 | 2946 | 5.14e-06 | CD4,CDC6,WFS1,NOTCH2,MAP2K2,NOTCH3,STAT3,GATA6,DUSP6,GDF6,KAT6B,BRAF,BMPER,RAD21,CDT1,KMT2D,UBB,PLXND1,RAI1,WT1,ALX4,RERE,PTPN11,ATR,DOK7,ARID1B,COMT,NSMF,BMPR1A,ATRX,MED12,LRP4,GPC3,GLI3,SMARCA4,TRPV4,ZEB2 |
| **GO:0035295** | tube development | 18 | 793 | 1.20e-05 | BRIP1,NOTCH3,GATA6,HPGD,BMPER,COL3A1,PLXND1,WT1,ALX4,BMPR1A,ATRX,MED12,HSPG2,GPC3,GLI3,FOXP2,ZEB2,STRA6 |
| **GO:0044237** | cellular metabolic process | 70 | 8797 | 1.40e-05 | KCNQ1,CDC6,WFS1,POLR3B,GMNN,SLC35D1,CHSY1,NOTCH2,BRIP1,XYLT1,MAP2K2,NOTCH3,STAT3,B3GAT3,HYAL1,GATA6,DUSP6,MED13L,GDF6,KAT6B,BUB1B,FANCD2,BRAF,HPGD,RAD21,CDT1,KMT2D,GUSB,COL3A1,UBB,SHMT1,FAM20C,POLE,DDX59,WT1,ALX4,RERE,PTPN11,ATR,POMGNT2,ARID1B,POX2,ACTB,TTC37,COMT,SMC3,DMD,SOX3,POMGNT1,BMPR1A,POLR3A,POLR1C,ATRX,MED12,HSPG2,PSAT1,PLCB4,CDKL5,FANCA,GPC3,GLI3,FOXP2,TMEM67,PEX5,EARS2,SMARCA4,FTO,ZEB2,STRA6,RECQL4 |
| **GO:0048869** | cellular developmental process | 40 | 3533 | 1.67e-05 | CD4,CHSY1,NOTCH2,BRIP1,NOTCH3,STAT3,GATA6,DUSP6,GDF6,BRAF,TBC1D24,KMT2D,COL3A1,UBB,PLXND1,CEP57,FAM20C,WT1,COL7A1,RERE,PTPN11,ATR,POMGNT2,ARID1B,DMD,LMNA,L1CAM,SOX3,NSMF,BMPR1A,ATRX,MED12,HSPG2,LRP4,CDKL5,GPC3,GLI3,FOXP2,TRPV4,ZEB2 |
| **GO:0009059** | macromolecule biosynthetic process | 40 | 3576 | 2.24e-05 | CDC6,POLR3B,GMNN,SLC35D1,CHSY1,NOTCH2,BRIP1,XYLT1,NOTCH3,STAT3,B3GAT3,HYAL1,GATA6,MED13L,KAT6B,RAD21,CDT1,KMT2D,UBB,POLE,WT1,ALX4,RERE,ATR,POMGNT2,ARID1B,SOX3,POMGNT1,POLR3A,POLR1C,ATRX,MED12,HSPG2,GPC3,GLI3,FOXP2,EARS2,SMARCA4,ZEB2,RECQL4 |
| **GO:0045595** | regulation of cell differentiation | 26 | 1695 | 2.24e-05 | CD4,NOTCH2,NOTCH3,STAT3,GATA6,DUSP6,GDF6,FANCD2,KMT2D,COL3A1,PLXND1,FAM20C,DMD,L1CAM,SOX3,NSMF,BMPR1A,COL5A2,LRP4,CDKL5,FANCA,GLI3,TRPV4,FTO,FBN2,ZEB2 |
| **GO:0030154** | cell differentiation | 39 | 3457 | 2.61e-05 | CD4,CHSY1,NOTCH2,BRIP1,NOTCH3,STAT3,GATA6,DUSP6,GDF6,BRAF,TBC1D24,KMT2D,COL3A1,UBB,PLXND1,CEP57,FAM20C,WT1,COL7A1,RERE,PTPN11,POMGNT2,ARID1B,DMD,LMNA,L1CAM,SOX3,NSMF,BMPR1A,ATRX,MED12,HSPG2,LRP4,CDKL5,GPC3,GLI3,FOXP2,TRPV4,ZEB2 |
| **GO:0006807** | nitrogen compound metabolic process | 67 | 8349 | 2.82e-05 | KCNQ1,CDC6,WFS1,POLR3B,GMNN,SLC35D1,CHSY1,NOTCH2,BRIP1,XYLT1,MAP2K2,NOTCH3,STAT3,B3GAT3,HYAL1,GATA6,DUSP6,MED13L,GDF6,KAT6B,BUB1B,FANCD2,BRAF,RAD21,CDT1,KMT2D,GUSB,COL3A1,UBB,SHMT1,FAM20C,POLE,DDX59,WT1,ALX4,RERE,PTPN11,ATR,POMGNT2,ARID1B,POX2,ACTB,TTC37,COMT,SMC3,DMD,SOX3,POMGNT1,BMPR1A,POLR3A,POLR1C,ATRX,MED12,HSPG2,PSAT1,CDKL5,FANCA,GPC3,GLI3,FOXP2,TMEM67,PEX5,EARS2,SMARCA4,FTO,ZEB2,RECQL4 |
| **GO:0060840** | artery development | 7 | 81 | 2.84e-05 | NOTCH3,HPGD,COL3A1,PLXND1,BMPR1A,GLI3,STRA6 |
| **GO:0031325** | positive regulation of cellular metabolic process | 36 | 3060 | 3.20e-05 | CD4,CDC6,WFS1,NOTCH2,MAP2K2,NOTCH3,STAT3,GATA6,DUSP6,GDF6,KAT6B,BRAF,BMPER,RAD21,CDT1,KMT2D,UBB,PLXND1,RAI1,WT1,ALX4,RERE,PTPN11,ATR,DOK7,ARID1B,COMT,NSMF,BMPR1A,ATRX,MED12,LRP4,GLI3,SMARCA4,TRPV4,ZEB2 |
| **GO:0044238** | primary metabolic process | 69 | 8808 | 3.35e-05 | KCNQ1,CDC6,WFS1,POLR3B,GMNN,SLC35D1,CHSY1,NOTCH2,BRIP1,XYLT1,MAP2K2,NOTCH3,STAT3,B3GAT3,HYAL1,GATA6,DUSP6,MED13L,GDF6,KAT6B,BUB1B,FANCD2,BRAF,HPGD,RAD21,CDT1,KMT2D,GUSB,COL3A1,UBB,SHMT1,FAM20C,POLE,DDX59,WT1,ALX4,RERE,PTPN11,ATR,POMGNT2,ARID1B,POX2,ACTB,TTC37,COMT,SMC3,SOX3,POMGNT1,BMPR1A,POLR3A,POLR1C,ATRX,MED12,HSPG2,PSAT1,PLCB4,CDKL5,FANCA,GPC3,GLI3,FOXP2,TMEM67,PEX5,EARS2,SMARCA4,FTO,ZEB2,STRA6,RECQL4 |
| **GO:0065007** | biological regulation | 82 | 11740 | 3.56e-05 | CD4,KCNQ1,CDC6,WFS1,POLR3B,GMNN,CHSY1,NOTCH2,BRIP1,LTBP2,MAP2K2,NOTCH3,STAT3,B3GAT3,HYAL1,GATA6,DUSP6,MED13L,GDF6,KAT6B,BUB1B,FANCD2,BRAF,TBC1D24,HPGD,BMPER,RAD21,CDT1,ANKRD11,KMT2D,COL3A1,UBB,PLXND1,CEP57,SHMT1,FAM20C,RAI1,WT1,COL7A1,ALX4,RERE,PTPN11,ATR,DOK7,ARID1B,POX2,ACTB,TTC37,COMT,SLC39A13,SMC3,DMD,LMNA,L1CAM,SOX3,NSMF,BMPR1A,POLR3A,POLR1C,ATRX,MED12,HSPG2,COL5A2,PLCB4,LRP4,CDKL5,FANCA,GPC3,GLI3,FOXP2,TMEM67,SEPT9,PEX5,SMARCA4,TRPV4,FTO,PIEZO2,FBN2,ZEB2,STRA6,RECQL4,NEB |
| **GO:0010033** | response to organic substance | 34 | 2815 | 3.97e-05 | CD4,KCNQ1,CDC6,WFS1,BRIP1,LTBP2,STAT3,HYAL1,GATA6,DUSP6,GDF6,KAT6B,HPGD,CDT1,COL3A1,UBB,CEP57,SHMT1,WT1,PTPN11,ARID1B,ACTB,COMT,LMNA,NSMF,BMPR1A,ATRX,MED12,COL5A2,LRP4,FOXP2,TMEM67,SMARCA4,TRPV4 |
| **GO:0072359** | circulatory system development | 17 | 807 | 5.26e-05 | KCNQ1,NOTCH2,NOTCH3,GATA6,HPGD,BMPER,COL3A1,PLXND1,WT1,PTPN11,LMNA,BMPR1A,MED12,HSPG2,GPC3,GLI3,STRA6 |
| **GO:0071704** | organic substance metabolic process | 70 | 9135 | 5.48e-05 | KCNQ1,CDC6,WFS1,POLR3B,GMNN,SLC35D1,CHSY1,NOTCH2,BRIP1,XYLT1,MAP2K2,NOTCH3,STAT3,B3GAT3,HYAL1,GATA6,DUSP6,MED13L,GDF6,KAT6B,BUB1B,FANCD2,BRAF,HPGD,RAD21,CDT1,KMT2D,GUSB,COL3A1,UBB,SHMT1,FAM20C,POLE,DDX59,WT1,ALX4,RERE,PTPN11,ATR,POMGNT2,ARID1B,POX2,ACTB,TTC37,COMT,SMC3,DMD,SOX3,POMGNT1,BMPR1A,POLR3A,POLR1C,ATRX,MED12,HSPG2,PSAT1,PLCB4,CDKL5,FANCA,GPC3,GLI3,FOXP2,TMEM67,PEX5,EARS2,SMARCA4,FTO,ZEB2,STRA6,RECQL4 |
| **GO:0008152** | metabolic process | 72 | 9569 | 5.62e-05 | KCNQ1,CDC6,WFS1,POLR3B,GMNN,SLC35D1,CHSY1,NOTCH2,BRIP1,XYLT1,MAP2K2,NOTCH3,STAT3,B3GAT3,HYAL1,GATA6,DUSP6,MED13L,GDF6,KAT6B,BUB1B,FANCD2,BRAF,HPGD,RAD21,CDT1,KMT2D,GUSB,COL3A1,UBB,SHMT1,FAM20C,POLE,DDX59,WT1,ALX4,RERE,PTPN11,ATR,POMGNT2,ARID1B,POX2,ACTB,TTC37,COMT,SMC3,DMD,SOX3,POMGNT1,BMPR1A,POLR3A,POLR1C,ATRX,MED12,HSPG2,PSAT1,PLCB4,CDKL5,FANCA,GPC3,GLI3,FOXP2,TMEM67,PEX5,EARS2,SMARCA4,TRPV4,TNXB,FTO,ZEB2,STRA6,RECQL4 |
| **GO:0060255** | regulation of macromolecule metabolic process | 54 | 6072 | 5.85e-05 | CD4,KCNQ1,CDC6,WFS1,GMNN,NOTCH2,BRIP1,MAP2K2,NOTCH3,STAT3,B3GAT3,GATA6,DUSP6,MED13L,GDF6,KAT6B,FANCD2,BRAF,BMPER,RAD21,CDT1,KMT2D,UBB,PLXND1,SHMT1,RAI1,WT1,COL7A1,ALX4,RERE,PTPN11,ATR,DOK7,ARID1B,ACTB,TTC37,SMC3,DMD,LMNA,SOX3,NSMF,BMPR1A,POLR1C,ATRX,MED12,HSPG2,LRP4,FANCA,GPC3,GLI3,FOXP2,SMARCA4,TRPV4,ZEB2 |
| **GO:0010628** | positive regulation of gene expression | 26 | 1826 | 6.39e-05 | CD4,MAP2K2,NOTCH3,STAT3,GATA6,GDF6,KAT6B,BRAF,RAD21,KMT2D,UBB,PLXND1,RAI1,WT1,ALX4,ARID1B,ACTB,LMNA,BMPR1A,POLR1C,ATRX,MED12,GLI3,SMARCA4,TRPV4,ZEB2 |
| **GO:0009987** | cellular process | 92 | 14652 | 7.18e-05 | CD4,KCNQ1,CDC6,WFS1,POLR3B,GMNN,SLC35D1,CHSY1,NOTCH2,BRIP1,XYLT1,LTBP2,MAP2K2,NOTCH3,STAT3,B3GAT3,HYAL1,GATA6,DUSP6,MED13L,GDF6,KAT6B,BUB1B,FANCD2,BRAF,TBC1D24,HPGD,BMPER,RAD21,CDT1,KMT2D,GUSB,COL3A1,UBB,PLXND1,CEP57,SHMT1,FAM20C,POLE,DDX59,WT1,COL7A1,ALX4,RERE,PTPN11,COL9A3,ATR,POMGNT2,ARID1B,POX2,ACTB,TTC37,COMT,SLC39A13,SMC3,DMD,LMNA,L1CAM,SOX3,NSMF,POMGNT1,BMPR1A,POLR3A,POLR1C,COL9A2,ATRX,MED12,HSPG2,COL5A2,PSAT1,PLCB4,LRP4,CDKL5,FANCA,GPC3,GLI3,FOXP2,TMEM67,SEPT9,PEX5,EARS2,SMARCA4,TRPV4,TNXB,FTO,PIEZO2,FBN2,TBCE,ZEB2,STRA6,RECQL4,NEB |
| **GO:0006024** | glycosaminoglycan biosynthetic process | 7 | 101 | 8.42e-05 | SLC35D1,CHSY1,XYLT1,B3GAT3,HYAL1,HSPG2,GPC3 |
| **GO:0019222** | regulation of metabolic process | 56 | 6516 | 8.46e-05 | CD4,KCNQ1,CDC6,WFS1,GMNN,NOTCH2,BRIP1,MAP2K2,NOTCH3,STAT3,B3GAT3,GATA6,DUSP6,MED13L,GDF6,KAT6B,FANCD2,BRAF,BMPER,RAD21,CDT1,KMT2D,UBB,PLXND1,SHMT1,FAM20C,RAI1,WT1,COL7A1,ALX4,RERE,PTPN11,ATR,DOK7,ARID1B,ACTB,TTC37,COMT,SMC3,DMD,LMNA,SOX3,NSMF,BMPR1A,POLR1C,ATRX,MED12,HSPG2,LRP4,FANCA,GPC3,GLI3,FOXP2,SMARCA4,TRPV4,ZEB2 |
| **GO:0043170** | macromolecule metabolic process | 61 | 7453 | 8.46e-05 | CDC6,WFS1,POLR3B,GMNN,SLC35D1,CHSY1,NOTCH2,BRIP1,XYLT1,MAP2K2,NOTCH3,STAT3,B3GAT3,HYAL1,GATA6,DUSP6,MED13L,GDF6,KAT6B,BUB1B,FANCD2,BRAF,RAD21,CDT1,KMT2D,GUSB,COL3A1,UBB,FAM20C,POLE,DDX59,WT1,ALX4,RERE,PTPN11,ATR,POMGNT2,ARID1B,ACTB,TTC37,SMC3,SOX3,POMGNT1,BMPR1A,POLR3A,POLR1C,ATRX,MED12,HSPG2,CDKL5,FANCA,GPC3,GLI3,FOXP2,TMEM67,PEX5,EARS2,SMARCA4,FTO,ZEB2,RECQL4 |
| **GO:0040008** | regulation of growth | 15 | 663 | 8.60e-05 | WFS1,NOTCH2,STAT3,HYAL1,GATA6,DUSP6,RAI1,WT1,PTPN11,L1CAM,BMPR1A,CDKL5,GPC3,SMARCA4,FTO |
| **GO:0051171** | regulation of nitrogen compound metabolic process | 52 | 5827 | 8.94e-05 | CD4,CDC6,WFS1,GMNN,NOTCH2,BRIP1,MAP2K2,NOTCH3,STAT3,B3GAT3,GATA6,DUSP6,MED13L,GDF6,KAT6B,FANCD2,BRAF,BMPER,RAD21,CDT1,KMT2D,UBB,PLXND1,SHMT1,RAI1,WT1,COL7A1,ALX4,RERE,PTPN11,ATR,DOK7,ARID1B,ACTB,COMT,SMC3,DMD,LMNA,SOX3,NSMF,BMPR1A,ATRX,MED12,HSPG2,LRP4,FANCA,GPC3,GLI3,FOXP2,SMARCA4,TRPV4,ZEB2 |
| **GO:0022603** | regulation of anatomical structure morphogenesis | 18 | 961 | 9.23e-05 | STAT3,HYAL1,GATA6,DUSP6,BMPER,PLXND1,WT1,L1CAM,NSMF,BMPR1A,MED12,HSPG2,COL5A2,LRP4,CDKL5,GPC3,FOXP2,ZEB2 |
| **GO:0030203** | glycosaminoglycan metabolic process | 8 | 153 | 9.23e-05 | SLC35D1,CHSY1,XYLT1,B3GAT3,HYAL1,GUSB,HSPG2,GPC3 |
| **GO:0072358** | cardiovascular system development | 13 | 496 | 9.23e-05 | KCNQ1,NOTCH3,GATA6,HPGD,BMPER,COL3A1,PLXND1,WT1,BMPR1A,HSPG2,GPC3,GLI3,STRA6 |
| **GO:1901576** | organic substance biosynthetic process | 45 | 4656 | 9.23e-05 | CDC6,POLR3B,GMNN,SLC35D1,CHSY1,NOTCH2,BRIP1,XYLT1,NOTCH3,STAT3,B3GAT3,HYAL1,GATA6,MED13L,KAT6B,HPGD,RAD21,CDT1,KMT2D,UBB,SHMT1,POLE,WT1,ALX4,RERE,PTPN11,ATR,POMGNT2,ARID1B,DMD,SOX3,POMGNT1,POLR3A,POLR1C,ATRX,MED12,HSPG2,PSAT1,GPC3,GLI3,FOXP2,EARS2,SMARCA4,ZEB2,RECQL4 |
| **GO:0010605** | negative regulation of macromolecule metabolic process | 31 | 2558 | 9.97e-05 | KCNQ1,CDC6,WFS1,GMNN,NOTCH2,BRIP1,MAP2K2,NOTCH3,STAT3,GATA6,DUSP6,KAT6B,KMT2D,UBB,SHMT1,WT1,COL7A1,RERE,ATR,TTC37,SMC3,DMD,SOX3,ATRX,HSPG2,GPC3,GLI3,FOXP2,SMARCA4,TRPV4,ZEB2 |
| **GO:0010468** | regulation of gene expression | 44 | 4533 | 0.00011 | CD4,KCNQ1,CDC6,WFS1,GMNN,NOTCH2,BRIP1,MAP2K2,NOTCH3,STAT3,GATA6,MED13L,GDF6,KAT6B,FANCD2,BRAF,BMPER,RAD21,CDT1,KMT2D,UBB,PLXND1,SHMT1,RAI1,WT1,ALX4,RERE,ARID1B,ACTB,TTC37,LMNA,SOX3,BMPR1A,POLR1C,ATRX,MED12,LRP4,FANCA,GPC3,GLI3,FOXP2,SMARCA4,TRPV4,ZEB2 |
| **GO:0040007** | growth | 11 | 357 | 0.00012 | ANKRD11,KMT2D,PTPN11,BMPR1A,ATRX,MED12,LRP4,GLI3,FOXP2,ZEB2,STRA6 |
| **GO:0045786** | negative regulation of cell cycle | 13 | 517 | 0.00012 | CDC6,GMNN,NOTCH2,BRIP1,BUB1B,HPGD,RAD21,CDT1,PTPN11,ATR,SMC3,ATRX,TMEM67 |
| **GO:0007399** | nervous system development | 28 | 2206 | 0.00014 | NOTCH2,NOTCH3,STAT3,GDF6,FANCD2,TBC1D24,COL3A1,UBB,PLXND1,RERE,PTPN11,POMGNT2,ARID1B,DMD,L1CAM,SOX3,NSMF,BMPR1A,ATRX,MED12,HSPG2,LRP4,CDKL5,GLI3,FOXP2,SMARCA4,TRPV4,ZEB2 |
| **GO:0009892** | negative regulation of metabolic process | 32 | 2762 | 0.00015 | KCNQ1,CDC6,WFS1,GMNN,NOTCH2,BRIP1,MAP2K2,NOTCH3,STAT3,GATA6,DUSP6,KAT6B,KMT2D,UBB,SHMT1,WT1,COL7A1,RERE,ATR,TTC37,COMT,SMC3,DMD,SOX3,ATRX,HSPG2,GPC3,GLI3,FOXP2,SMARCA4,TRPV4,ZEB2 |
| **GO:0035239** | tube morphogenesis | 14 | 615 | 0.00015 | NOTCH3,GATA6,HPGD,BMPER,COL3A1,PLXND1,WT1,BMPR1A,MED12,HSPG2,GPC3,GLI3,ZEB2,STRA6 |
| **GO:0030204** | chondroitin sulfate metabolic process | 5 | 41 | 0.00016 | SLC35D1,CHSY1,XYLT1,B3GAT3,HYAL1 |
| **GO:0034645** | cellular macromolecule biosynthetic process | 37 | 3518 | 0.00016 | CDC6,POLR3B,GMNN,SLC35D1,CHSY1,NOTCH2,BRIP1,XYLT1,NOTCH3,STAT3,B3GAT3,GATA6,MED13L,KAT6B,RAD21,CDT1,KMT2D,UBB,POLE,WT1,ALX4,RERE,ATR,POMGNT2,ARID1B,SOX3,POMGNT1,POLR3A,POLR1C,ATRX,MED12,GLI3,FOXP2,EARS2,SMARCA4,ZEB2,RECQL4 |
| **GO:0051726** | regulation of cell cycle | 19 | 1129 | 0.00016 | CDC6,GMNN,NOTCH2,BRIP1,MAP2K2,STAT3,HYAL1,GATA6,BUB1B,HPGD,RAD21,CDT1,CEP57,PTPN11,ATR,ACTB,SMC3,ATRX,TMEM67 |
| **GO:0080090** | regulation of primary metabolic process | 52 | 5982 | 0.00016 | CD4,CDC6,WFS1,GMNN,NOTCH2,BRIP1,MAP2K2,NOTCH3,STAT3,B3GAT3,GATA6,DUSP6,MED13L,GDF6,KAT6B,FANCD2,BRAF,BMPER,RAD21,CDT1,KMT2D,UBB,PLXND1,SHMT1,RAI1,WT1,COL7A1,ALX4,RERE,PTPN11,ATR,DOK7,ARID1B,ACTB,COMT,SMC3,DMD,LMNA,SOX3,NSMF,BMPR1A,ATRX,MED12,HSPG2,LRP4,FANCA,GPC3,GLI3,FOXP2,SMARCA4,TRPV4,ZEB2 |
| **GO:0042221** | response to chemical | 41 | 4153 | 0.00017 | CD4,KCNQ1,CDC6,WFS1,BRIP1,LTBP2,STAT3,HYAL1,GATA6,DUSP6,GDF6,KAT6B,FANCD2,BRAF,HPGD,CDT1,COL3A1,UBB,CEP57,SHMT1,WT1,PTPN11,ATR,ARID1B,POX2,ACTB,COMT,SLC39A13,LMNA,L1CAM,NSMF,BMPR1A,ATRX,MED12,COL5A2,LRP4,GLI3,FOXP2,TMEM67,SMARCA4,TRPV4 |
| **GO:0048638** | regulation of developmental growth | 10 | 302 | 0.00017 | STAT3,GATA6,DUSP6,RAI1,WT1,PTPN11,L1CAM,BMPR1A,CDKL5,FTO |
| **GO:0050767** | regulation of neurogenesis | 15 | 730 | 0.00018 | NOTCH3,STAT3,GDF6,COL3A1,PLXND1,DMD,L1CAM,SOX3,NSMF,BMPR1A,LRP4,CDKL5,GLI3,TRPV4,ZEB2 |
| **GO:0001568** | blood vessel development | 12 | 464 | 0.00019 | NOTCH3,GATA6,HPGD,BMPER,COL3A1,PLXND1,WT1,BMPR1A,HSPG2,GPC3,GLI3,STRA6 |
| **GO:0016043** | cellular component organization | 47 | 5163 | 0.00019 | CD4,GMNN,BRIP1,LTBP2,STAT3,KAT6B,BUB1B,FANCD2,TBC1D24,RAD21,CDT1,KMT2D,COL3A1,UBB,PLXND1,CEP57,SHMT1,WT1,COL7A1,RERE,PTPN11,COL9A3,ARID1B,ACTB,SMC3,DMD,LMNA,L1CAM,COL9A2,ATRX,HSPG2,COL5A2,LRP4,CDKL5,FANCA,GLI3,TMEM67,SEPT9,PEX5,SMARCA4,TRPV4,TNXB,FBN2,TBCE,ZEB2,RECQL4,NEB |
| **GO:0070887** | cellular response to chemical stimulus | 31 | 2672 | 0.00019 | CD4,KCNQ1,CDC6,WFS1,BRIP1,LTBP2,STAT3,HYAL1,GATA6,GDF6,KAT6B,FANCD2,BRAF,HPGD,COL3A1,UBB,CEP57,SHMT1,WT1,PTPN11,ARID1B,POX2,ACTB,LMNA,NSMF,BMPR1A,ATRX,MED12,COL5A2,LRP4,SMARCA4 |
| **GO:0003002** | regionalization | 10 | 313 | 0.00020 | CHSY1,DUSP6,WT1,ALX4,BMPR1A,MED12,LRP4,GPC3,GLI3,ZEB2 |
| **GO:0009888** | tissue development | 23 | 1626 | 0.00020 | CHSY1,HYAL1,GATA6,BMPER,COL3A1,PLXND1,FAM20C,WT1,COL7A1,ALX4,SLC39A13,LMNA,BMPR1A,ATRX,MED12,LRP4,GPC3,GLI3,FOXP2,TRPV4,FTO,ZEB2,STRA6 |
| **GO:0010557** | positive regulation of macromolecule biosynthetic process | 24 | 1758 | 0.00021 | CD4,MAP2K2,NOTCH3,STAT3,GATA6,GDF6,KAT6B,RAD21,CDT1,KMT2D,UBB,PLXND1,RAI1,WT1,ALX4,RERE,ATR,ARID1B,BMPR1A,ATRX,MED12,GLI3,SMARCA4,ZEB2 |
| **GO:0060284** | regulation of cell development | 16 | 846 | 0.00021 | NOTCH2,NOTCH3,STAT3,GDF6,COL3A1,PLXND1,DMD,L1CAM,SOX3,NSMF,BMPR1A,LRP4,CDKL5,GLI3,TRPV4,ZEB2 |
| **GO:2000112** | regulation of cellular macromolecule biosynthetic process | 40 | 4050 | 0.00021 | CD4,CDC6,WFS1,GMNN,NOTCH2,BRIP1,MAP2K2,NOTCH3,STAT3,GATA6,MED13L,GDF6,KAT6B,FANCD2,BMPER,RAD21,CDT1,KMT2D,UBB,PLXND1,SHMT1,RAI1,WT1,ALX4,RERE,ATR,ARID1B,SMC3,SOX3,BMPR1A,ATRX,MED12,LRP4,FANCA,GPC3,GLI3,FOXP2,SMARCA4,TRPV4,ZEB2 |
| **GO:0001822** | kidney development | 9 | 251 | 0.00022 | WFS1,NOTCH3,HPGD,BMPER,WT1,LRP4,GPC3,GLI3,STRA6 |
| **GO:0022008** | neurogenesis | 22 | 1519 | 0.00022 | NOTCH3,STAT3,GDF6,TBC1D24,COL3A1,UBB,PLXND1,RERE,PTPN11,POMGNT2,ARID1B,DMD,L1CAM,SOX3,NSMF,BMPR1A,MED12,LRP4,CDKL5,GLI3,TRPV4,ZEB2 |
| **GO:0031323** | regulation of cellular metabolic process | 52 | 6082 | 0.00022 | CD4,CDC6,WFS1,GMNN,NOTCH2,BRIP1,MAP2K2,NOTCH3,STAT3,B3GAT3,GATA6,DUSP6,MED13L,GDF6,KAT6B,FANCD2,BRAF,BMPER,RAD21,CDT1,KMT2D,UBB,PLXND1,SHMT1,FAM20C,RAI1,WT1,COL7A1,ALX4,RERE,PTPN11,ATR,DOK7,ARID1B,ACTB,COMT,SMC3,DMD,LMNA,SOX3,NSMF,BMPR1A,ATRX,MED12,LRP4,FANCA,GPC3,GLI3,FOXP2,SMARCA4,TRPV4,ZEB2 |
| **GO:0045935** | positive regulation of nucleobase-containing compound metabolic process | 24 | 1770 | 0.00022 | CD4,MAP2K2,NOTCH3,STAT3,GATA6,GDF6,KAT6B,RAD21,CDT1,KMT2D,UBB,PLXND1,RAI1,WT1,ALX4,RERE,ATR,ARID1B,BMPR1A,ATRX,MED12,GLI3,SMARCA4,ZEB2 |
| **GO:0050789** | regulation of biological process | 77 | 11116 | 0.00022 | CD4,KCNQ1,CDC6,WFS1,POLR3B,GMNN,CHSY1,NOTCH2,BRIP1,LTBP2,MAP2K2,NOTCH3,STAT3,B3GAT3,HYAL1,GATA6,DUSP6,MED13L,GDF6,KAT6B,BUB1B,FANCD2,BRAF,HPGD,BMPER,RAD21,CDT1,KMT2D,COL3A1,UBB,PLXND1,CEP57,SHMT1,FAM20C,RAI1,WT1,COL7A1,ALX4,RERE,PTPN11,ATR,DOK7,ARID1B,POX2,ACTB,TTC37,COMT,SMC3,DMD,LMNA,L1CAM,SOX3,NSMF,BMPR1A,POLR3A,POLR1C,ATRX,MED12,HSPG2,COL5A2,PLCB4,LRP4,CDKL5,FANCA,GPC3,GLI3,FOXP2,TMEM67,SEPT9,PEX5,SMARCA4,TRPV4,FTO,FBN2,ZEB2,STRA6,NEB |
| **GO:1903508** | positive regulation of nucleic acid-templated transcription | 22 | 1520 | 0.00022 | CD4,MAP2K2,NOTCH3,STAT3,GATA6,GDF6,KAT6B,RAD21,KMT2D,UBB,PLXND1,RAI1,WT1,ALX4,RERE,ARID1B,BMPR1A,ATRX,MED12,GLI3,SMARCA4,ZEB2 |
| **GO:0007548** | sex differentiation | 9 | 252 | 0.00023 | BRIP1,GATA6,WT1,PTPN11,COL9A3,BMPR1A,ATRX,FANCA,STRA6 |
| **GO:0044249** | cellular biosynthetic process | 43 | 4567 | 0.00023 | CDC6,POLR3B,GMNN,SLC35D1,CHSY1,NOTCH2,BRIP1,XYLT1,NOTCH3,STAT3,B3GAT3,HYAL1,GATA6,MED13L,KAT6B,HPGD,RAD21,CDT1,KMT2D,UBB,SHMT1,POLE,WT1,ALX4,RERE,PTPN11,ATR,POMGNT2,ARID1B,DMD,SOX3,POMGNT1,POLR3A,POLR1C,ATRX,MED12,PSAT1,GLI3,FOXP2,EARS2,SMARCA4,ZEB2,RECQL4 |
| **GO:0007507** | heart development | 12 | 485 | 0.00024 | NOTCH2,GATA6,COL3A1,PLXND1,WT1,PTPN11,LMNA,BMPR1A,MED12,GPC3,GLI3,STRA6 |
| **GO:0048699** | generation of neurons | 21 | 1422 | 0.00024 | NOTCH3,STAT3,GDF6,TBC1D24,COL3A1,UBB,PLXND1,RERE,PTPN11,POMGNT2,ARID1B,DMD,L1CAM,SOX3,NSMF,BMPR1A,LRP4,CDKL5,GLI3,TRPV4,ZEB2 |
| **GO:0010558** | negative regulation of macromolecule biosynthetic process | 21 | 1425 | 0.00025 | CDC6,WFS1,GMNN,NOTCH3,STAT3,GATA6,KAT6B,KMT2D,UBB,SHMT1,WT1,RERE,ATR,SMC3,SOX3,ATRX,GLI3,FOXP2,SMARCA4,TRPV4,ZEB2 |
| **GO:0050896** | response to stimulus | 61 | 7824 | 0.00026 | CD4,KCNQ1,CDC6,WFS1,POLR3B,CHSY1,NOTCH2,BRIP1,LTBP2,MAP2K2,NOTCH3,STAT3,HYAL1,GATA6,DUSP6,GDF6,KAT6B,FANCD2,BRAF,HPGD,RAD21,CDT1,KMT2D,GUSB,COL3A1,UBB,PLXND1,CEP57,SHMT1,POLE,WT1,PTPN11,ATR,ARID1B,POX2,ACTB,COMT,SLC39A13,SMC3,DMD,LMNA,L1CAM,NSMF,BMPR1A,POLR3A,ATRX,MED12,HSPG2,COL5A2,PLCB4,LRP4,FANCA,GLI3,FOXP2,TMEM67,SMARCA4,TRPV4,FTO,PIEZO2,STRA6,RECQL4 |
| **GO:0051240** | positive regulation of multicellular organismal process | 22 | 1551 | 0.00026 | CD4,KCNQ1,POLR3B,STAT3,HYAL1,GATA6,GDF6,BMPER,PLXND1,FAM20C,WT1,DMD,L1CAM,NSMF,BMPR1A,POLR3A,CDKL5,GLI3,FOXP2,TRPV4,FBN2,ZEB2 |
| **GO:0045893** | positive regulation of transcription, DNA-templated | 21 | 1435 | 0.00027 | CD4,MAP2K2,NOTCH3,STAT3,GATA6,GDF6,KAT6B,RAD21,KMT2D,UBB,PLXND1,RAI1,WT1,ALX4,ARID1B,BMPR1A,ATRX,MED12,GLI3,SMARCA4,ZEB2 |
| **GO:0006355** | regulation of transcription, DNA-templated | 37 | 3661 | 0.00028 | CD4,CDC6,WFS1,GMNN,NOTCH2,BRIP1,MAP2K2,NOTCH3,STAT3,GATA6,MED13L,GDF6,KAT6B,FANCD2,BMPER,RAD21,CDT1,KMT2D,UBB,PLXND1,RAI1,WT1,ALX4,RERE,ARID1B,SOX3,BMPR1A,ATRX,MED12,LRP4,FANCA,GPC3,GLI3,FOXP2,SMARCA4,TRPV4,ZEB2 |
| **GO:0019219** | regulation of nucleobase-containing compound metabolic process | 40 | 4133 | 0.00028 | CD4,CDC6,WFS1,GMNN,NOTCH2,BRIP1,MAP2K2,NOTCH3,STAT3,GATA6,MED13L,GDF6,KAT6B,FANCD2,BMPER,RAD21,CDT1,KMT2D,UBB,PLXND1,RAI1,WT1,ALX4,RERE,ATR,ARID1B,SMC3,LMNA,SOX3,BMPR1A,ATRX,MED12,LRP4,FANCA,GPC3,GLI3,FOXP2,SMARCA4,TRPV4,ZEB2 |
| **GO:0071310** | cellular response to organic substance | 27 | 2219 | 0.00030 | CD4,KCNQ1,CDC6,WFS1,BRIP1,LTBP2,STAT3,HYAL1,GATA6,GDF6,KAT6B,HPGD,COL3A1,UBB,CEP57,SHMT1,WT1,PTPN11,ARID1B,ACTB,LMNA,NSMF,BMPR1A,MED12,COL5A2,LRP4,SMARCA4 |
| **GO:0006260** | DNA replication | 8 | 203 | 0.00031 | CDC6,GMNN,BRIP1,CDT1,POLE,ATR,ATRX,RECQL4 |
| **GO:0009790** | embryo development | 16 | 890 | 0.00031 | SLC35D1,HYAL1,GATA6,ANKRD11,POLE,WT1,COL7A1,ALX4,BMPR1A,MED12,LRP4,GPC3,GLI3,FBN2,ZEB2,STRA6 |
| **GO:0048589** | developmental growth | 10 | 340 | 0.00031 | ANKRD11,KMT2D,PTPN11,BMPR1A,ATRX,MED12,LRP4,GLI3,ZEB2,STRA6 |
| **GO:0060322** | head development | 14 | 692 | 0.00031 | NOTCH3,FANCD2,ANKRD11,COL3A1,RERE,PTPN11,SOX3,BMPR1A,ATRX,HSPG2,GLI3,FOXP2,ZEB2,STRA6 |
| **GO:0070848** | response to growth factor | 12 | 507 | 0.00031 | LTBP2,HYAL1,GATA6,DUSP6,GDF6,HPGD,COL3A1,UBB,CEP57,PTPN11,BMPR1A,LRP4 |
| **GO:0048705** | skeletal system morphogenesis | 8 | 204 | 0.00032 | CHSY1,HYAL1,ANKRD11,ALX4,MED12,GLI3,TRPV4,FBN2 |
| **GO:0006275** | regulation of DNA replication | 6 | 96 | 0.00033 | CDC6,GMNN,CDT1,ATR,SMC3,ATRX |
| **GO:0007423** | sensory organ development | 12 | 515 | 0.00033 | KCNQ1,STAT3,BMPER,WT1,PTPN11,SOX3,COL5A2,GLI3,FOXP2,SMARCA4,FBN2,STRA6 |
| **GO:0022414** | reproductive process | 20 | 1350 | 0.00033 | BRIP1,STAT3,GATA6,BUB1B,FANCD2,HPGD,RAD21,KMT2D,CEP57,WT1,PTPN11,COL9A3,COMT,SMC3,SOX3,BMPR1A,ATRX,FANCA,GLI3,STRA6 |
| **GO:0031327** | negative regulation of cellular biosynthetic process | 21 | 1479 | 0.00033 | CDC6,WFS1,GMNN,NOTCH3,STAT3,GATA6,KAT6B,KMT2D,UBB,SHMT1,WT1,RERE,ATR,SMC3,SOX3,ATRX,GLI3,FOXP2,SMARCA4,TRPV4,ZEB2 |
| **GO:0031328** | positive regulation of cellular biosynthetic process | 24 | 1846 | 0.00033 | CD4,MAP2K2,NOTCH3,STAT3,GATA6,GDF6,KAT6B,RAD21,CDT1,KMT2D,UBB,PLXND1,RAI1,WT1,ALX4,RERE,ATR,ARID1B,BMPR1A,ATRX,MED12,GLI3,SMARCA4,ZEB2 |
| **GO:0035108** | limb morphogenesis | 7 | 147 | 0.00033 | ALX4,BMPR1A,ATRX,LRP4,GPC3,GLI3,FBN2 |
| **GO:0048468** | cell development | 21 | 1493 | 0.00038 | CHSY1,BRIP1,GDF6,TBC1D24,KMT2D,UBB,CEP57,FAM20C,WT1,RERE,PTPN11,ARID1B,DMD,LMNA,L1CAM,BMPR1A,ATRX,MED12,LRP4,GLI3,ZEB2 |
| **GO:0046483** | heterocycle metabolic process | 43 | 4716 | 0.00039 | KCNQ1,CDC6,POLR3B,GMNN,SLC35D1,NOTCH2,BRIP1,NOTCH3,STAT3,GATA6,MED13L,KAT6B,FANCD2,RAD21,CDT1,KMT2D,UBB,SHMT1,FAM20C,POLE,DDX59,WT1,ALX4,RERE,ATR,ARID1B,POX2,TTC37,SMC3,SOX3,POLR3A,POLR1C,ATRX,MED12,PSAT1,FANCA,GLI3,FOXP2,EARS2,SMARCA4,FTO,ZEB2,RECQL4 |
| **GO:0003006** | developmental process involved in reproduction | 13 | 622 | 0.00040 | BRIP1,GATA6,KMT2D,CEP57,WT1,PTPN11,COL9A3,SOX3,BMPR1A,ATRX,FANCA,GLI3,STRA6 |
| **GO:0031401** | positive regulation of protein modification process | 18 | 1149 | 0.00041 | CD4,CDC6,WFS1,NOTCH2,MAP2K2,STAT3,DUSP6,GDF6,BRAF,BMPER,UBB,PTPN11,DOK7,NSMF,BMPR1A,LRP4,TRPV4,ZEB2 |
| **GO:0007517** | muscle organ development | 9 | 287 | 0.00042 | COL3A1,WT1,ALX4,DMD,LMNA,BMPR1A,FOXP2,STRA6,NEB |
| **GO:0046661** | male sex differentiation | 7 | 155 | 0.00042 | BRIP1,GATA6,WT1,COL9A3,BMPR1A,ATRX,FANCA |
| **GO:0048844** | artery morphogenesis | 5 | 59 | 0.00042 | NOTCH3,HPGD,COL3A1,BMPR1A,STRA6 |
| **GO:0050794** | regulation of cellular process | 73 | 10484 | 0.00043 | CD4,KCNQ1,CDC6,WFS1,GMNN,CHSY1,NOTCH2,BRIP1,LTBP2,MAP2K2,NOTCH3,STAT3,B3GAT3,HYAL1,GATA6,DUSP6,MED13L,GDF6,KAT6B,BUB1B,FANCD2,BRAF,HPGD,BMPER,RAD21,CDT1,KMT2D,COL3A1,UBB,PLXND1,CEP57,SHMT1,FAM20C,RAI1,WT1,COL7A1,ALX4,RERE,PTPN11,ATR,DOK7,ARID1B,POX2,ACTB,COMT,SMC3,DMD,LMNA,L1CAM,SOX3,NSMF,BMPR1A,ATRX,MED12,HSPG2,COL5A2,PLCB4,LRP4,CDKL5,FANCA,GPC3,GLI3,FOXP2,TMEM67,SEPT9,PEX5,SMARCA4,TRPV4,FTO,FBN2,ZEB2,STRA6,NEB |
| **GO:0006725** | cellular aromatic compound metabolic process | 43 | 4754 | 0.00044 | KCNQ1,CDC6,POLR3B,GMNN,SLC35D1,NOTCH2,BRIP1,NOTCH3,STAT3,GATA6,MED13L,KAT6B,FANCD2,RAD21,CDT1,KMT2D,UBB,SHMT1,FAM20C,POLE,DDX59,WT1,ALX4,RERE,ATR,ARID1B,TTC37,COMT,SMC3,SOX3,POLR3A,POLR1C,ATRX,MED12,PSAT1,FANCA,GLI3,FOXP2,EARS2,SMARCA4,FTO,ZEB2,RECQL4 |
| **GO:0048646** | anatomical structure formation involved in morphogenesis | 15 | 831 | 0.00044 | NOTCH3,GATA6,BMPER,PLXND1,FAM20C,WT1,COL7A1,PTPN11,BMPR1A,MED12,HSPG2,GLI3,FBN2,ZEB2,STRA6 |
| **GO:0051128** | regulation of cellular component organization | 27 | 2306 | 0.00044 | CDC6,MAP2K2,HYAL1,BUB1B,RAD21,CDT1,PLXND1,WT1,PTPN11,ATR,SMC3,DMD,LMNA,L1CAM,NSMF,ATRX,HSPG2,LRP4,CDKL5,GPC3,TMEM67,SEPT9,PEX5,SMARCA4,TRPV4,ZEB2,NEB |
| **GO:0051172** | negative regulation of nitrogen compound metabolic process | 27 | 2307 | 0.00044 | CDC6,WFS1,GMNN,NOTCH3,STAT3,GATA6,DUSP6,KAT6B,KMT2D,UBB,SHMT1,WT1,COL7A1,RERE,ATR,COMT,SMC3,DMD,SOX3,ATRX,HSPG2,GPC3,GLI3,FOXP2,SMARCA4,TRPV4,ZEB2 |
| **GO:0001934** | positive regulation of protein phosphorylation | 16 | 941 | 0.00045 | CD4,CDC6,NOTCH2,MAP2K2,STAT3,DUSP6,GDF6,BRAF,BMPER,UBB,PTPN11,DOK7,BMPR1A,LRP4,TRPV4,ZEB2 |
| **GO:0045937** | positive regulation of phosphate metabolic process | 17 | 1052 | 0.00045 | CD4,CDC6,NOTCH2,MAP2K2,STAT3,DUSP6,GDF6,BRAF,BMPER,UBB,PTPN11,DOK7,NSMF,BMPR1A,LRP4,TRPV4,ZEB2 |
| **GO:0051094** | positive regulation of developmental process | 19 | 1286 | 0.00046 | CD4,STAT3,HYAL1,GATA6,GDF6,BMPER,PLXND1,FAM20C,WT1,DMD,LMNA,L1CAM,NSMF,BMPR1A,CDKL5,GLI3,FOXP2,FBN2,ZEB2 |
| **GO:0048598** | embryonic morphogenesis | 12 | 545 | 0.00047 | HYAL1,GATA6,COL7A1,ALX4,BMPR1A,MED12,LRP4,GPC3,GLI3,FBN2,ZEB2,STRA6 |
| **GO:0030198** | extracellular matrix organization | 9 | 296 | 0.00048 | COL3A1,WT1,COL7A1,COL9A3,COL9A2,HSPG2,COL5A2,TNXB,FBN2 |
| **GO:0033047** | regulation of mitotic sister chromatid segregation | 5 | 63 | 0.00050 | CDC6,BUB1B,RAD21,CDT1,ATRX |
| **GO:1901360** | organic cyclic compound metabolic process | 44 | 4963 | 0.00050 | KCNQ1,CDC6,POLR3B,GMNN,SLC35D1,NOTCH2,BRIP1,NOTCH3,STAT3,GATA6,MED13L,KAT6B,FANCD2,RAD21,CDT1,KMT2D,UBB,SHMT1,FAM20C,POLE,DDX59,WT1,ALX4,RERE,ATR,ARID1B,POX2,TTC37,COMT,SMC3,SOX3,POLR3A,POLR1C,ATRX,MED12,PSAT1,FANCA,GLI3,FOXP2,EARS2,SMARCA4,FTO,ZEB2,RECQL4 |
| **GO:0050650** | chondroitin sulfate proteoglycan biosynthetic process | 4 | 30 | 0.00052 | SLC35D1,CHSY1,XYLT1,B3GAT3 |
| **GO:1903510** | mucopolysaccharide metabolic process | 6 | 109 | 0.00052 | SLC35D1,CHSY1,XYLT1,B3GAT3,HYAL1,GUSB |
| **GO:0045934** | negative regulation of nucleobase-containing compound metabolic process | 20 | 1424 | 0.00053 | CDC6,WFS1,GMNN,NOTCH3,STAT3,GATA6,KAT6B,KMT2D,UBB,WT1,RERE,ATR,SMC3,SOX3,ATRX,GLI3,FOXP2,SMARCA4,TRPV4,ZEB2 |
| **GO:0051716** | cellular response to stimulus | 51 | 6212 | 0.00055 | CD4,KCNQ1,CDC6,WFS1,NOTCH2,BRIP1,LTBP2,MAP2K2,NOTCH3,STAT3,HYAL1,GATA6,DUSP6,GDF6,KAT6B,FANCD2,BRAF,HPGD,RAD21,COL3A1,UBB,PLXND1,CEP57,SHMT1,POLE,WT1,PTPN11,ATR,ARID1B,POX2,ACTB,COMT,SMC3,DMD,LMNA,NSMF,BMPR1A,ATRX,MED12,COL5A2,PLCB4,LRP4,FANCA,GLI3,TMEM67,SMARCA4,TRPV4,FTO,PIEZO2,STRA6,RECQL4 |
| **GO:0071363** | cellular response to growth factor stimulus | 11 | 477 | 0.00065 | LTBP2,HYAL1,GATA6,GDF6,HPGD,COL3A1,UBB,CEP57,PTPN11,BMPR1A,LRP4 |
| **GO:1901699** | cellular response to nitrogen compound | 12 | 568 | 0.00065 | KCNQ1,CDC6,BRIP1,STAT3,COL3A1,SHMT1,WT1,ARID1B,ACTB,NSMF,ATRX,COL5A2 |
| **GO:0006259** | DNA metabolic process | 14 | 773 | 0.00069 | CDC6,GMNN,BRIP1,FANCD2,RAD21,CDT1,UBB,POLE,ATR,SMC3,ATRX,FANCA,FTO,RECQL4 |
| **GO:0051241** | negative regulation of multicellular organismal process | 17 | 1098 | 0.00069 | CHSY1,NOTCH3,STAT3,GATA6,COL3A1,RAI1,WT1,PTPN11,COMT,LMNA,SOX3,BMPR1A,HSPG2,COL5A2,LRP4,GLI3,TRPV4 |
| **GO:0006139** | nucleobase-containing compound metabolic process | 41 | 4551 | 0.00072 | KCNQ1,CDC6,POLR3B,GMNN,SLC35D1,NOTCH2,BRIP1,NOTCH3,STAT3,GATA6,MED13L,KAT6B,FANCD2,RAD21,CDT1,KMT2D,UBB,SHMT1,FAM20C,POLE,DDX59,WT1,ALX4,RERE,ATR,ARID1B,TTC37,SMC3,SOX3,POLR3A,POLR1C,ATRX,MED12,FANCA,GLI3,FOXP2,EARS2,SMARCA4,FTO,ZEB2,RECQL4 |
| **GO:0019827** | stem cell population maintenance | 6 | 118 | 0.00072 | NOTCH2,STAT3,FANCD2,SMC3,BMPR1A,MED12 |
| **GO:0071417** | cellular response to organonitrogen compound | 11 | 485 | 0.00072 | KCNQ1,CDC6,BRIP1,STAT3,COL3A1,SHMT1,WT1,ARID1B,ACTB,NSMF,COL5A2 |
| **GO:0008156** | negative regulation of DNA replication | 4 | 34 | 0.00073 | CDC6,GMNN,ATR,SMC3 |
| **GO:2000113** | negative regulation of cellular macromolecule biosynthetic process | 19 | 1348 | 0.00075 | CDC6,WFS1,GMNN,NOTCH3,STAT3,GATA6,KAT6B,KMT2D,UBB,SHMT1,WT1,ATR,SMC3,ATRX,GLI3,FOXP2,SMARCA4,TRPV4,ZEB2 |
| **GO:0006281** | DNA repair | 11 | 491 | 0.00077 | BRIP1,FANCD2,RAD21,UBB,POLE,ATR,SMC3,ATRX,FANCA,FTO,RECQL4 |
| **GO:0022402** | cell cycle process | 15 | 890 | 0.00077 | CDC6,GMNN,NOTCH2,BRIP1,BUB1B,FANCD2,RAD21,CDT1,CEP57,POLE,SMC3,LMNA,ATRX,FANCA,TBCE |
| **GO:0090304** | nucleic acid metabolic process | 37 | 3941 | 0.00086 | CDC6,POLR3B,GMNN,NOTCH2,BRIP1,NOTCH3,STAT3,GATA6,MED13L,KAT6B,FANCD2,RAD21,CDT1,KMT2D,UBB,POLE,DDX59,WT1,ALX4,RERE,ATR,ARID1B,TTC37,SMC3,SOX3,POLR3A,POLR1C,ATRX,MED12,FANCA,GLI3,FOXP2,EARS2,SMARCA4,FTO,ZEB2,RECQL4 |
| **GO:0031399** | regulation of protein modification process | 22 | 1747 | 0.00087 | CD4,CDC6,WFS1,NOTCH2,MAP2K2,STAT3,B3GAT3,DUSP6,GDF6,BRAF,BMPER,UBB,PTPN11,DOK7,ACTB,DMD,NSMF,BMPR1A,ATRX,LRP4,TRPV4,ZEB2 |
| **GO:0009967** | positive regulation of signal transduction | 20 | 1493 | 0.00088 | CD4,CHSY1,NOTCH2,MAP2K2,STAT3,GATA6,DUSP6,GDF6,BRAF,BMPER,KMT2D,COL3A1,UBB,PTPN11,ATR,BMPR1A,GPC3,SMARCA4,TRPV4,ZEB2 |
| **GO:0045664** | regulation of neuron differentiation | 12 | 595 | 0.00089 | NOTCH3,GDF6,PLXND1,DMD,L1CAM,SOX3,NSMF,LRP4,CDKL5,GLI3,TRPV4,ZEB2 |
| **GO:0090287** | regulation of cellular response to growth factor stimulus | 8 | 254 | 0.00091 | GATA6,DUSP6,BMPER,UBB,FAM20C,DMD,GPC3,FBN2 |
| **GO:0030326** | embryonic limb morphogenesis | 6 | 126 | 0.00094 | ALX4,BMPR1A,LRP4,GPC3,GLI3,FBN2 |
| **GO:0009966** | regulation of signal transduction | 31 | 3033 | 0.00095 | CD4,WFS1,CHSY1,NOTCH2,MAP2K2,NOTCH3,STAT3,GATA6,DUSP6,GDF6,FANCD2,BRAF,BMPER,KMT2D,COL3A1,UBB,FAM20C,PTPN11,ATR,DMD,LMNA,BMPR1A,MED12,LRP4,FANCA,GPC3,GLI3,SMARCA4,TRPV4,FBN2,ZEB2 |
| **GO:0060541** | respiratory system development | 7 | 188 | 0.00097 | GATA6,WT1,BMPR1A,GPC3,GLI3,FOXP2,STRA6 |
| **GO:0007178** | transmembrane receptor protein serine/threonine kinase signaling pathway | 7 | 189 | 0.00100 | LTBP2,GDF6,HPGD,COL3A1,UBB,BMPR1A,LRP4 |
| **GO:0042475** | odontogenesis of dentin-containing tooth | 5 | 78 | 0.0010 | ANKRD11,FAM20C,BMPR1A,LRP4,GLI3 |
| **GO:0043410** | positive regulation of MAPK cascade | 11 | 512 | 0.0010 | CD4,NOTCH2,MAP2K2,DUSP6,GDF6,BRAF,BMPER,UBB,PTPN11,TRPV4,ZEB2 |
| **GO:0050768** | negative regulation of neurogenesis | 8 | 260 | 0.0010 | NOTCH3,STAT3,COL3A1,SOX3,BMPR1A,LRP4,GLI3,TRPV4 |
| **GO:0008584** | male gonad development | 6 | 132 | 0.0011 | BRIP1,GATA6,WT1,COL9A3,ATRX,FANCA |
| **GO:0051174** | regulation of phosphorus metabolic process | 21 | 1658 | 0.0011 | CD4,CDC6,NOTCH2,MAP2K2,STAT3,B3GAT3,DUSP6,GDF6,BRAF,BMPER,UBB,FAM20C,PTPN11,DOK7,ACTB,DMD,NSMF,BMPR1A,LRP4,TRPV4,ZEB2 |
| **GO:0006357** | regulation of transcription by RNA polymerase II | 28 | 2633 | 0.0012 | WFS1,BRIP1,NOTCH3,STAT3,GATA6,MED13L,GDF6,BMPER,RAD21,KMT2D,UBB,PLXND1,RAI1,WT1,ALX4,RERE,ARID1B,SOX3,BMPR1A,ATRX,MED12,LRP4,GPC3,GLI3,FOXP2,SMARCA4,TRPV4,ZEB2 |
| **GO:0009952** | anterior/posterior pattern specification | 7 | 197 | 0.0012 | WT1,ALX4,BMPR1A,MED12,GPC3,GLI3,ZEB2 |
| **GO:0010629** | negative regulation of gene expression | 21 | 1670 | 0.0012 | KCNQ1,WFS1,GMNN,NOTCH2,BRIP1,MAP2K2,NOTCH3,STAT3,GATA6,KAT6B,KMT2D,UBB,SHMT1,WT1,TTC37,ATRX,GLI3,FOXP2,SMARCA4,TRPV4,ZEB2 |
| **GO:0033554** | cellular response to stress | 20 | 1553 | 0.0014 | WFS1,BRIP1,GATA6,FANCD2,BRAF,RAD21,UBB,POLE,PTPN11,ATR,POX2,COMT,SMC3,LMNA,ATRX,FANCA,TMEM67,TRPV4,FTO,RECQL4 |
| **GO:0007179** | transforming growth factor beta receptor signaling pathway | 5 | 88 | 0.0016 | LTBP2,HPGD,COL3A1,UBB,BMPR1A |
| **GO:0010171** | body morphogenesis | 4 | 44 | 0.0016 | ANKRD11,PTPN11,GPC3,STRA6 |
| **GO:0090329** | regulation of DNA-dependent DNA replication | 4 | 44 | 0.0016 | GMNN,CDT1,SMC3,ATRX |
| **GO:0007417** | central nervous system development | 14 | 861 | 0.0017 | NOTCH3,STAT3,FANCD2,COL3A1,RERE,PTPN11,SOX3,BMPR1A,ATRX,MED12,HSPG2,GLI3,FOXP2,ZEB2 |
| **GO:0009612** | response to mechanical stimulus | 7 | 210 | 0.0017 | COL3A1,PTPN11,DMD,FOXP2,TRPV4,PIEZO2,STRA6 |
| **GO:0007420** | brain development | 12 | 650 | 0.0018 | NOTCH3,FANCD2,COL3A1,RERE,PTPN11,SOX3,BMPR1A,ATRX,HSPG2,GLI3,FOXP2,ZEB2 |
| **GO:0061061** | muscle structure development | 10 | 457 | 0.0018 | GATA6,COL3A1,WT1,ALX4,DMD,LMNA,BMPR1A,FOXP2,STRA6,NEB |
| **GO:0010646** | regulation of cell communication | 32 | 3327 | 0.0019 | CD4,WFS1,CHSY1,NOTCH2,MAP2K2,NOTCH3,STAT3,GATA6,DUSP6,GDF6,FANCD2,BRAF,BMPER,KMT2D,COL3A1,UBB,FAM20C,PTPN11,ATR,DMD,LMNA,NSMF,BMPR1A,MED12,LRP4,FANCA,GPC3,GLI3,SMARCA4,TRPV4,FBN2,ZEB2 |
| **GO:0034641** | cellular nitrogen compound metabolic process | 43 | 5126 | 0.0019 | KCNQ1,CDC6,POLR3B,GMNN,SLC35D1,NOTCH2,BRIP1,NOTCH3,STAT3,GATA6,MED13L,KAT6B,FANCD2,RAD21,CDT1,KMT2D,UBB,SHMT1,FAM20C,POLE,DDX59,WT1,ALX4,RERE,ATR,ARID1B,TTC37,SMC3,DMD,SOX3,POLR3A,POLR1C,ATRX,MED12,PSAT1,FANCA,GLI3,FOXP2,EARS2,SMARCA4,FTO,ZEB2,RECQL4 |
| **GO:0045944** | positive regulation of transcription by RNA polymerase II | 16 | 1104 | 0.0019 | NOTCH3,STAT3,GATA6,RAD21,KMT2D,UBB,PLXND1,RAI1,WT1,ALX4,BMPR1A,ATRX,MED12,GLI3,SMARCA4,ZEB2 |
| **GO:0051321** | meiotic cell cycle | 7 | 214 | 0.0019 | BRIP1,BUB1B,FANCD2,RAD21,SMC3,ATRX,FANCA |
| **GO:1901698** | response to nitrogen compound | 15 | 988 | 0.0019 | KCNQ1,CDC6,WFS1,BRIP1,STAT3,COL3A1,SHMT1,WT1,ARID1B,ACTB,NSMF,ATRX,COL5A2,TMEM67,TRPV4 |
| **GO:1903507** | negative regulation of nucleic acid-templated transcription | 17 | 1220 | 0.0019 | WFS1,GMNN,NOTCH3,STAT3,GATA6,KAT6B,KMT2D,UBB,WT1,RERE,SOX3,ATRX,GLI3,FOXP2,SMARCA4,TRPV4,ZEB2 |
| **GO:0010243** | response to organonitrogen compound | 14 | 876 | 0.0020 | KCNQ1,CDC6,WFS1,BRIP1,STAT3,COL3A1,SHMT1,WT1,ARID1B,ACTB,NSMF,COL5A2,TMEM67,TRPV4 |
| **GO:0060324** | face development | 4 | 48 | 0.0020 | ANKRD11,PTPN11,SOX3,STRA6 |
| **GO:0030071** | regulation of mitotic metaphase/anaphase transition | 4 | 49 | 0.0021 | CDC6,BUB1B,RAD21,CDT1 |
| **GO:0036297** | interstrand cross-link repair | 4 | 49 | 0.0021 | FANCD2,UBB,ATR,FANCA |
| **GO:0048514** | blood vessel morphogenesis | 9 | 381 | 0.0021 | NOTCH3,HPGD,BMPER,COL3A1,PLXND1,WT1,BMPR1A,HSPG2,STRA6 |
| **GO:0090092** | regulation of transmembrane receptor protein serine/threonine kinase signaling pathway | 7 | 220 | 0.0021 | GATA6,GDF6,BMPER,UBB,BMPR1A,GPC3,FBN2 |
| **GO:0001932** | regulation of protein phosphorylation | 18 | 1370 | 0.0022 | CD4,CDC6,NOTCH2,MAP2K2,STAT3,DUSP6,GDF6,BRAF,BMPER,UBB,PTPN11,DOK7,ACTB,DMD,BMPR1A,LRP4,TRPV4,ZEB2 |
| **GO:0023051** | regulation of signaling | 32 | 3360 | 0.0022 | CD4,WFS1,CHSY1,NOTCH2,MAP2K2,NOTCH3,STAT3,GATA6,DUSP6,GDF6,FANCD2,BRAF,BMPER,KMT2D,COL3A1,UBB,FAM20C,PTPN11,ATR,DMD,LMNA,NSMF,BMPR1A,MED12,LRP4,FANCA,GPC3,GLI3,SMARCA4,TRPV4,FBN2,ZEB2 |
| **GO:0071371** | cellular response to gonadotropin stimulus | 3 | 18 | 0.0022 | GATA6,WT1,NSMF |
| **GO:0031324** | negative regulation of cellular metabolic process | 26 | 2463 | 0.0023 | CDC6,WFS1,GMNN,NOTCH3,STAT3,GATA6,DUSP6,KAT6B,KMT2D,UBB,SHMT1,WT1,COL7A1,RERE,ATR,COMT,SMC3,DMD,SOX3,ATRX,GPC3,GLI3,FOXP2,SMARCA4,TRPV4,ZEB2 |
| **GO:0048745** | smooth muscle tissue development | 3 | 19 | 0.0024 | COL3A1,FOXP2,STRA6 |
| **GO:0007049** | cell cycle | 17 | 1263 | 0.0026 | CDC6,GMNN,NOTCH2,BRIP1,BUB1B,FANCD2,RAD21,CDT1,CEP57,POLE,PTPN11,SMC3,LMNA,ATRX,FANCA,SEPT9,TBCE |
| **GO:0007088** | regulation of mitotic nuclear division | 6 | 160 | 0.0026 | CDC6,BUB1B,RAD21,CDT1,SMC3,ATRX |
| **GO:0032268** | regulation of cellular protein metabolic process | 26 | 2486 | 0.0026 | CD4,CDC6,WFS1,NOTCH2,MAP2K2,STAT3,B3GAT3,DUSP6,GDF6,BRAF,BMPER,UBB,SHMT1,WT1,COL7A1,PTPN11,DOK7,ACTB,DMD,NSMF,BMPR1A,ATRX,LRP4,GPC3,TRPV4,ZEB2 |
| **GO:0045597** | positive regulation of cell differentiation | 14 | 908 | 0.0026 | CD4,STAT3,GATA6,GDF6,PLXND1,FAM20C,DMD,L1CAM,NSMF,BMPR1A,CDKL5,GLI3,FBN2,ZEB2 |
| **GO:1903046** | meiotic cell cycle process | 6 | 161 | 0.0026 | BRIP1,BUB1B,FANCD2,RAD21,ATRX,FANCA |
| **GO:0019220** | regulation of phosphate metabolic process | 20 | 1657 | 0.0027 | CD4,CDC6,NOTCH2,MAP2K2,STAT3,B3GAT3,DUSP6,GDF6,BRAF,BMPER,UBB,PTPN11,DOK7,ACTB,DMD,NSMF,BMPR1A,LRP4,TRPV4,ZEB2 |
| **GO:0030324** | lung development | 6 | 162 | 0.0027 | GATA6,BMPR1A,GPC3,GLI3,FOXP2,STRA6 |
| **GO:0007167** | enzyme linked receptor protein signaling pathway | 12 | 698 | 0.0029 | CD4,LTBP2,STAT3,GDF6,HPGD,COL3A1,UBB,CEP57,PTPN11,ACTB,BMPR1A,LRP4 |
| **GO:0048608** | reproductive structure development | 9 | 405 | 0.0030 | BRIP1,GATA6,WT1,PTPN11,COL9A3,ATRX,FANCA,GLI3,STRA6 |
| **GO:0051246** | regulation of protein metabolic process | 27 | 2668 | 0.0030 | CD4,CDC6,WFS1,NOTCH2,MAP2K2,STAT3,B3GAT3,DUSP6,GDF6,BRAF,BMPER,UBB,SHMT1,WT1,COL7A1,PTPN11,DOK7,ACTB,DMD,NSMF,BMPR1A,ATRX,HSPG2,LRP4,GPC3,TRPV4,ZEB2 |
| **GO:0060348** | bone development | 6 | 166 | 0.0030 | CHSY1,ANKRD11,FAM20C,PTPN11,GLI3,TRPV4 |
| **GO:0003279** | cardiac septum development | 5 | 107 | 0.0032 | NOTCH2,GATA6,PLXND1,BMPR1A,STRA6 |
| **GO:0009628** | response to abiotic stimulus | 15 | 1052 | 0.0032 | BRIP1,HYAL1,GATA6,FANCD2,COL3A1,UBB,PTPN11,ATR,DMD,LMNA,NSMF,FOXP2,TRPV4,PIEZO2,STRA6 |
| **GO:0043585** | nose morphogenesis | 2 | 3 | 0.0032 | GLI3,STRA6 |
| **GO:0071163** | DNA replication preinitiation complex assembly | 2 | 3 | 0.0032 | GMNN,CDT1 |
| **GO:0034502** | protein localization to chromosome | 4 | 58 | 0.0033 | BUB1B,RAD21,ATR,ATRX |
| **GO:0045669** | positive regulation of osteoblast differentiation | 4 | 58 | 0.0033 | FAM20C,BMPR1A,GLI3,FBN2 |
| **GO:1901700** | response to oxygen-containing compound | 18 | 1427 | 0.0033 | CD4,KCNQ1,CDC6,BRIP1,STAT3,HYAL1,HPGD,CDT1,COL3A1,SHMT1,WT1,ARID1B,ACTB,COMT,NSMF,COL5A2,FOXP2,TRPV4 |
| **GO:1904385** | cellular response to angiotensin | 3 | 22 | 0.0033 | CDC6,BRIP1,ARID1B |
| **GO:0060420** | regulation of heart growth | 4 | 60 | 0.0037 | GATA6,DUSP6,WT1,BMPR1A |
| **GO:0006027** | glycosaminoglycan catabolic process | 4 | 62 | 0.0040 | HYAL1,GUSB,HSPG2,GPC3 |
| **GO:0006261** | DNA-dependent DNA replication | 5 | 114 | 0.0040 | CDC6,GMNN,CDT1,POLE,ATRX |
| **GO:0007059** | chromosome segregation | 7 | 253 | 0.0040 | BRIP1,BUB1B,FANCD2,RAD21,CDT1,CEP57,SMC3 |
| **GO:0045927** | positive regulation of growth | 7 | 252 | 0.0040 | WFS1,HYAL1,GATA6,WT1,L1CAM,BMPR1A,CDKL5 |
| **GO:0051247** | positive regulation of protein metabolic process | 19 | 1587 | 0.0040 | CD4,CDC6,WFS1,NOTCH2,MAP2K2,STAT3,DUSP6,GDF6,BRAF,BMPER,UBB,PTPN11,DOK7,NSMF,BMPR1A,LRP4,GPC3,TRPV4,ZEB2 |
| **GO:1902533** | positive regulation of intracellular signal transduction | 14 | 959 | 0.0040 | CD4,NOTCH2,MAP2K2,STAT3,DUSP6,GDF6,BRAF,BMPER,COL3A1,UBB,PTPN11,ATR,TRPV4,ZEB2 |
| **GO:0001654** | eye development | 8 | 339 | 0.0042 | STAT3,WT1,COL5A2,GLI3,FOXP2,SMARCA4,FBN2,STRA6 |
| **GO:0030278** | regulation of ossification | 6 | 181 | 0.0042 | CHSY1,FAM20C,BMPR1A,LRP4,GLI3,FBN2 |
| **GO:0009725** | response to hormone | 13 | 854 | 0.0043 | CD4,CDC6,BRIP1,STAT3,GATA6,HPGD,WT1,PTPN11,ARID1B,NSMF,MED12,FOXP2,TRPV4 |
| **GO:0030206** | chondroitin sulfate biosynthetic process | 3 | 25 | 0.0043 | SLC35D1,CHSY1,XYLT1 |
| **GO:0051984** | positive regulation of chromosome segregation | 3 | 25 | 0.0043 | CDC6,RAD21,CDT1 |
| **GO:1902895** | positive regulation of pri-miRNA transcription by RNA polymerase II | 3 | 25 | 0.0043 | STAT3,BMPR1A,SMARCA4 |
| **GO:0042733** | embryonic digit morphogenesis | 4 | 64 | 0.0044 | ALX4,BMPR1A,LRP4,GLI3 |
| **GO:0006974** | cellular response to DNA damage stimulus | 12 | 749 | 0.0046 | BRIP1,FANCD2,RAD21,UBB,POLE,PTPN11,ATR,SMC3,ATRX,FANCA,FTO,RECQL4 |
| **GO:0035270** | endocrine system development | 5 | 120 | 0.0047 | GATA6,WT1,SOX3,BMPR1A,STRA6 |
| **GO:0000280** | nuclear division | 7 | 268 | 0.0051 | BRIP1,BUB1B,FANCD2,RAD21,CDT1,CEP57,FANCA |
| **GO:0033048** | negative regulation of mitotic sister chromatid segregation | 3 | 27 | 0.0051 | BUB1B,RAD21,ATRX |
| **GO:0048706** | embryonic skeletal system development | 5 | 122 | 0.0051 | SLC35D1,HYAL1,ALX4,MED12,GLI3 |
| **GO:0070192** | chromosome organization involved in meiotic cell cycle | 4 | 67 | 0.0051 | BRIP1,BUB1B,FANCD2,ATRX |
| **GO:0048584** | positive regulation of response to stimulus | 22 | 2054 | 0.0053 | CD4,POLR3B,CHSY1,NOTCH2,MAP2K2,STAT3,GATA6,DUSP6,GDF6,BRAF,BMPER,KMT2D,COL3A1,UBB,PTPN11,ATR,ACTB,BMPR1A,GPC3,SMARCA4,TRPV4,ZEB2 |
| **GO:0000075** | cell cycle checkpoint | 6 | 193 | 0.0054 | CDC6,BRIP1,BUB1B,CDT1,PTPN11,ATR |
| **GO:0040014** | regulation of multicellular organism growth | 4 | 69 | 0.0054 | STAT3,RAI1,PTPN11,FTO |
| **GO:0051276** | chromosome organization | 14 | 999 | 0.0054 | BRIP1,KAT6B,BUB1B,FANCD2,CDT1,KMT2D,UBB,CEP57,RERE,ARID1B,SMC3,ATRX,SMARCA4,RECQL4 |
| **GO:0006950** | response to stress | 30 | 3267 | 0.0055 | CD4,WFS1,POLR3B,BRIP1,STAT3,HYAL1,GATA6,DUSP6,FANCD2,BRAF,RAD21,COL3A1,UBB,POLE,PTPN11,ATR,ARID1B,POX2,COMT,SMC3,LMNA,POLR3A,ATRX,HSPG2,FANCA,GLI3,TMEM67,TRPV4,FTO,RECQL4 |
| **GO:0048565** | digestive tract development | 5 | 126 | 0.0055 | GATA6,COL3A1,ALX4,GLI3,STRA6 |
| **GO:0061448** | connective tissue development | 6 | 194 | 0.0055 | CHSY1,HYAL1,SLC39A13,BMPR1A,TRPV4,FTO |
| **GO:0070372** | regulation of ERK1 and ERK2 cascade | 7 | 274 | 0.0056 | CD4,NOTCH2,DUSP6,BRAF,BMPER,PTPN11,TRPV4 |
| **GO:0097070** | ductus arteriosus closure | 2 | 5 | 0.0056 | HPGD,STRA6 |
| **GO:0070374** | positive regulation of ERK1 and ERK2 cascade | 6 | 196 | 0.0057 | CD4,NOTCH2,BRAF,BMPER,PTPN11,TRPV4 |
| **GO:0098813** | nuclear chromosome segregation | 6 | 196 | 0.0057 | BRIP1,BUB1B,FANCD2,CDT1,CEP57,SMC3 |
| **GO:0030900** | forebrain development | 8 | 366 | 0.0061 | NOTCH3,COL3A1,SOX3,BMPR1A,ATRX,GLI3,FOXP2,ZEB2 |
| **GO:0043627** | response to estrogen | 4 | 74 | 0.0066 | GATA6,KMT2D,COMT,GLI3 |
| **GO:0060537** | muscle tissue development | 7 | 284 | 0.0067 | GATA6,COL3A1,WT1,LMNA,BMPR1A,FOXP2,STRA6 |
| **GO:0009954** | proximal/distal pattern formation | 3 | 31 | 0.0068 | CHSY1,LRP4,GLI3 |
| **GO:0060325** | face morphogenesis | 3 | 31 | 0.0068 | ANKRD11,PTPN11,STRA6 |
| **GO:0033043** | regulation of organelle organization | 15 | 1155 | 0.0069 | CDC6,MAP2K2,BUB1B,RAD21,CDT1,ATR,SMC3,LMNA,ATRX,CDKL5,TMEM67,SEPT9,TRPV4,ZEB2,NEB |
| **GO:0042297** | vocal learning | 2 | 6 | 0.0070 | FOXP2,STRA6 |
| **GO:1903224** | regulation of endodermal cell differentiation | 2 | 6 | 0.0070 | DUSP6,COL5A2 |
| **GO:2000348** | regulation of CD40 signaling pathway | 2 | 6 | 0.0070 | FANCD2,FANCA |
| **GO:0007492** | endoderm development | 4 | 76 | 0.0071 | GATA6,COL7A1,BMPR1A,MED12 |
| **GO:0050772** | positive regulation of axonogenesis | 4 | 76 | 0.0071 | PLXND1,L1CAM,CDKL5,ZEB2 |
| **GO:0045892** | negative regulation of transcription, DNA-templated | 15 | 1169 | 0.0076 | WFS1,GMNN,NOTCH3,STAT3,GATA6,KAT6B,KMT2D,UBB,WT1,ATRX,GLI3,FOXP2,SMARCA4,TRPV4,ZEB2 |
| **GO:0000122** | negative regulation of transcription by RNA polymerase II | 12 | 809 | 0.0079 | WFS1,NOTCH3,STAT3,GATA6,UBB,WT1,ATRX,GLI3,FOXP2,SMARCA4,TRPV4,ZEB2 |
| **GO:0060429** | epithelium development | 14 | 1055 | 0.0083 | GATA6,BMPER,PLXND1,FAM20C,WT1,ALX4,BMPR1A,ATRX,MED12,LRP4,GPC3,GLI3,ZEB2,STRA6 |
| **GO:0060430** | lung saccule development | 2 | 7 | 0.0087 | GATA6,STRA6 |
| **GO:0030501** | positive regulation of bone mineralization | 3 | 35 | 0.0089 | FAM20C,BMPR1A,FBN2 |
| **GO:0048645** | animal organ formation | 3 | 35 | 0.0089 | GATA6,BMPR1A,GLI3 |
| **GO:2001222** | regulation of neuron migration | 3 | 35 | 0.0089 | STAT3,COL3A1,NSMF |
| **GO:0042127** | regulation of cell population proliferation | 18 | 1594 | 0.0091 | CD4,CDC6,BRIP1,NOTCH3,STAT3,HYAL1,GATA6,HPGD,KMT2D,WT1,COMT,LMNA,BMPR1A,FANCA,GPC3,GLI3,FOXP2,FTO |
| **GO:0048592** | eye morphogenesis | 5 | 145 | 0.0091 | STAT3,COL5A2,GLI3,FBN2,STRA6 |
| **GO:0048583** | regulation of response to stimulus | 33 | 3882 | 0.0092 | CD4,WFS1,POLR3B,CHSY1,NOTCH2,MAP2K2,NOTCH3,STAT3,GATA6,DUSP6,GDF6,FANCD2,BRAF,BMPER,KMT2D,COL3A1,UBB,FAM20C,PTPN11,ATR,ACTB,DMD,LMNA,BMPR1A,MED12,LRP4,FANCA,GPC3,GLI3,SMARCA4,TRPV4,FBN2,ZEB2 |
| **GO:0140013** | meiotic nuclear division | 5 | 146 | 0.0092 | BRIP1,BUB1B,FANCD2,RAD21,FANCA |
| **GO:1901342** | regulation of vasculature development | 7 | 305 | 0.0092 | STAT3,HYAL1,GATA6,BMPER,PLXND1,WT1,HSPG2 |
| **GO:0010464** | regulation of mesenchymal cell proliferation | 3 | 36 | 0.0093 | LMNA,BMPR1A,FOXP2 |
| **GO:0035137** | hindlimb morphogenesis | 3 | 36 | 0.0093 | ALX4,BMPR1A,GPC3 |
| **GO:0031570** | DNA integrity checkpoint | 5 | 147 | 0.0094 | CDC6,BRIP1,CDT1,PTPN11,ATR |
| **GO:0065004** | protein-DNA complex assembly | 6 | 223 | 0.0095 | GMNN,KAT6B,CDT1,UBB,ATRX,SMARCA4 |
| **GO:0007346** | regulation of mitotic cell cycle | 10 | 608 | 0.0096 | CDC6,MAP2K2,HYAL1,BUB1B,RAD21,CDT1,CEP57,PTPN11,SMC3,ATRX |
| **GO:0045058** | T cell selection | 3 | 37 | 0.0098 | CD4,STAT3,GLI3 |
| **GO:0048286** | lung alveolus development | 3 | 37 | 0.0098 | GATA6,FOXP2,STRA6 |
| **GO:0060421** | positive regulation of heart growth | 3 | 37 | 0.0098 | GATA6,WT1,BMPR1A |
| **GO:0001657** | ureteric bud development | 4 | 86 | 0.0099 | BMPER,WT1,GPC3,GLI3 |
| **GO:0017038** | protein import | 5 | 150 | 0.0099 | STAT3,CEP57,RERE,LMNA,PEX5 |
| **GO:0009953** | dorsal/ventral pattern formation | 4 | 87 | 0.0102 | DUSP6,BMPR1A,LRP4,GLI3 |
| **GO:0010470** | regulation of gastrulation | 3 | 38 | 0.0102 | DUSP6,BMPR1A,COL5A2 |
| **GO:0033044** | regulation of chromosome organization | 7 | 313 | 0.0102 | CDC6,BUB1B,RAD21,CDT1,ATR,LMNA,ATRX |
| **GO:0045165** | cell fate commitment | 6 | 230 | 0.0107 | NOTCH2,NOTCH3,STAT3,GATA6,WT1,GLI3 |
| **GO:0009798** | axis specification | 4 | 89 | 0.0108 | WT1,BMPR1A,LRP4,GPC3 |
| **GO:0030199** | collagen fibril organization | 3 | 39 | 0.0108 | COL3A1,COL5A2,TNXB |
| **GO:0043933** | protein-containing complex subunit organization | 19 | 1770 | 0.0108 | GMNN,KAT6B,CDT1,KMT2D,COL3A1,UBB,CEP57,SHMT1,COL7A1,DMD,ATRX,COL5A2,LRP4,FANCA,SEPT9,PEX5,SMARCA4,TRPV4,TNXB |
| **GO:0045860** | positive regulation of protein kinase activity | 9 | 517 | 0.0112 | CD4,CDC6,MAP2K2,DUSP6,BRAF,UBB,PTPN11,DOK7,ZEB2 |
| **GO:1901137** | carbohydrate derivative biosynthetic process | 10 | 625 | 0.0112 | SLC35D1,CHSY1,XYLT1,B3GAT3,HYAL1,SHMT1,POMGNT2,POMGNT1,HSPG2,GPC3 |
| **GO:0006383** | transcription by RNA polymerase III | 3 | 40 | 0.0114 | POLR3B,POLR3A,POLR1C |
| **GO:0048568** | embryonic organ development | 8 | 417 | 0.0114 | HYAL1,POLE,ALX4,BMPR1A,MED12,GLI3,FBN2,STRA6 |
| **GO:0000278** | mitotic cell cycle | 10 | 628 | 0.0115 | CDC6,GMNN,BUB1B,CDT1,CEP57,POLE,PTPN11,SMC3,LMNA,TBCE |
| **GO:0045926** | negative regulation of growth | 6 | 235 | 0.0115 | NOTCH2,HYAL1,RAI1,WT1,GPC3,SMARCA4 |
| **GO:0060539** | diaphragm development | 2 | 9 | 0.0115 | WT1,STRA6 |
| **GO:0032879** | regulation of localization | 24 | 2524 | 0.0118 | CD4,KCNQ1,WFS1,MAP2K2,STAT3,B3GAT3,HYAL1,BRAF,BMPER,CDT1,COL3A1,PLXND1,PTPN11,ACTB,COMT,DMD,LMNA,NSMF,BMPR1A,LRP4,GPC3,GLI3,TRPV4,FTO |
| **GO:0048704** | embryonic skeletal system morphogenesis | 4 | 92 | 0.0118 | HYAL1,ALX4,MED12,GLI3 |
| **GO:0035282** | segmentation | 4 | 93 | 0.0122 | WT1,BMPR1A,MED12,ZEB2 |
| **GO:0050769** | positive regulation of neurogenesis | 8 | 425 | 0.0125 | GDF6,PLXND1,DMD,L1CAM,NSMF,CDKL5,GLI3,ZEB2 |
| **GO:0050770** | regulation of axonogenesis | 5 | 162 | 0.0129 | PLXND1,L1CAM,LRP4,CDKL5,ZEB2 |
| **GO:0051130** | positive regulation of cellular component organization | 14 | 1128 | 0.0133 | HYAL1,RAD21,CDT1,PLXND1,ATR,DMD,L1CAM,ATRX,LRP4,CDKL5,GPC3,SEPT9,TRPV4,ZEB2 |
| **GO:0048639** | positive regulation of developmental growth | 5 | 165 | 0.0138 | GATA6,WT1,L1CAM,BMPR1A,CDKL5 |
| **GO:0033365** | protein localization to organelle | 10 | 649 | 0.0141 | STAT3,BUB1B,RAD21,UBB,CEP57,RERE,ATR,LMNA,ATRX,PEX5 |
| **GO:0001658** | branching involved in ureteric bud morphogenesis | 3 | 45 | 0.0149 | WT1,GPC3,GLI3 |
| **GO:0060976** | coronary vasculature development | 3 | 45 | 0.0149 | GATA6,PLXND1,GPC3 |
| **GO:0071902** | positive regulation of protein serine/threonine kinase activity | 7 | 340 | 0.0149 | CDC6,MAP2K2,DUSP6,BRAF,UBB,PTPN11,ZEB2 |
| **GO:0001763** | morphogenesis of a branching structure | 5 | 169 | 0.0151 | PLXND1,WT1,RERE,GPC3,GLI3 |
| **GO:0040029** | regulation of gene expression, epigenetic | 6 | 251 | 0.0152 | KCNQ1,STAT3,KMT2D,ARID1B,ACTB,POLR1C |
| **GO:0003179** | heart valve morphogenesis | 3 | 46 | 0.0155 | NOTCH2,BMPR1A,STRA6 |
| **GO:0006563** | L-serine metabolic process | 2 | 11 | 0.0155 | SHMT1,PSAT1 |
| **GO:0006606** | protein import into nucleus | 4 | 101 | 0.0155 | STAT3,CEP57,RERE,LMNA |
| **GO:0010975** | regulation of neuron projection development | 8 | 443 | 0.0155 | PLXND1,DMD,L1CAM,NSMF,LRP4,CDKL5,TRPV4,ZEB2 |
| **GO:0043009** | chordate embryonic development | 9 | 550 | 0.0155 | SLC35D1,HYAL1,GATA6,ANKRD11,ALX4,BMPR1A,MED12,GLI3,ZEB2 |
| **GO:0072520** | seminiferous tubule development | 2 | 11 | 0.0155 | BRIP1,ATRX |
| **GO:2000105** | positive regulation of DNA-dependent DNA replication | 2 | 11 | 0.0155 | CDT1,ATRX |
| **GO:2001223** | negative regulation of neuron migration | 2 | 11 | 0.0155 | STAT3,COL3A1 |
| **GO:0044772** | mitotic cell cycle phase transition | 6 | 254 | 0.0157 | CDC6,GMNN,BUB1B,CDT1,CEP57,POLE |
| **GO:0090100** | positive regulation of transmembrane receptor protein serine/threonine kinase signaling pathway | 4 | 102 | 0.0159 | GATA6,GDF6,BMPR1A,GPC3 |
| **GO:0009100** | glycoprotein metabolic process | 7 | 349 | 0.0164 | SLC35D1,CHSY1,XYLT1,B3GAT3,HYAL1,POMGNT2,POMGNT1 |
| **GO:0044093** | positive regulation of molecular function | 18 | 1713 | 0.0168 | CD4,CDC6,GMNN,MAP2K2,STAT3,B3GAT3,DUSP6,BRAF,TBC1D24,CDT1,UBB,PLXND1,PTPN11,DOK7,DMD,CDKL5,SMARCA4,ZEB2 |
| **GO:0048806** | genitalia development | 3 | 48 | 0.0168 | WT1,PTPN11,STRA6 |
| **GO:0051093** | negative regulation of developmental process | 12 | 910 | 0.0168 | NOTCH3,STAT3,COL3A1,RAI1,WT1,SOX3,BMPR1A,HSPG2,COL5A2,LRP4,GLI3,TRPV4 |
| **GO:0035563** | positive regulation of chromatin binding | 2 | 12 | 0.0171 | GMNN,CDT1 |
| **GO:0060019** | radial glial cell differentiation | 2 | 12 | 0.0171 | STAT3,GLI3 |
| **GO:0010828** | positive regulation of glucose transmembrane transport | 3 | 49 | 0.0176 | BRAF,PTPN11,GPC3 |
| **GO:0035904** | aorta development | 3 | 49 | 0.0176 | COL3A1,PLXND1,BMPR1A |
| **GO:0018130** | heterocycle biosynthetic process | 27 | 3095 | 0.0178 | POLR3B,SLC35D1,NOTCH2,NOTCH3,STAT3,GATA6,MED13L,KAT6B,RAD21,KMT2D,UBB,SHMT1,POLE,WT1,ALX4,RERE,ARID1B,SOX3,POLR3A,POLR1C,ATRX,MED12,PSAT1,GLI3,FOXP2,SMARCA4,ZEB2 |
| **GO:0010769** | regulation of cell morphogenesis involved in differentiation | 6 | 263 | 0.0179 | PLXND1,L1CAM,NSMF,LRP4,CDKL5,ZEB2 |
| **GO:0019438** | aromatic compound biosynthetic process | 27 | 3100 | 0.0180 | POLR3B,SLC35D1,NOTCH2,NOTCH3,STAT3,GATA6,MED13L,KAT6B,RAD21,KMT2D,UBB,SHMT1,POLE,WT1,ALX4,RERE,ARID1B,SOX3,POLR3A,POLR1C,ATRX,MED12,PSAT1,GLI3,FOXP2,SMARCA4,ZEB2 |
| **GO:0043406** | positive regulation of MAP kinase activity | 6 | 264 | 0.0180 | MAP2K2,DUSP6,BRAF,UBB,PTPN11,ZEB2 |
| **GO:0071773** | cellular response to BMP stimulus | 4 | 107 | 0.0180 | GATA6,GDF6,BMPR1A,LRP4 |
| **GO:0010564** | regulation of cell cycle process | 10 | 684 | 0.0186 | CDC6,HYAL1,GATA6,BUB1B,RAD21,CDT1,CEP57,SMC3,ATRX,TMEM67 |
| **GO:0033262** | regulation of nuclear cell cycle DNA replication | 2 | 13 | 0.0190 | CDT1,ATRX |
| **GO:0035723** | interleukin-15-mediated signaling pathway | 2 | 13 | 0.0190 | CD4,STAT3 |
| **GO:0045815** | positive regulation of gene expression, epigenetic | 3 | 51 | 0.0190 | ARID1B,ACTB,POLR1C |
| **GO:0022607** | cellular component assembly | 22 | 2343 | 0.0200 | GMNN,BRIP1,KAT6B,CDT1,KMT2D,UBB,PLXND1,CEP57,SHMT1,COL7A1,ARID1B,ACTB,DMD,ATRX,LRP4,FANCA,TMEM67,SEPT9,PEX5,SMARCA4,TRPV4,TNXB |
| **GO:0010948** | negative regulation of cell cycle process | 6 | 273 | 0.0207 | BUB1B,RAD21,CDT1,SMC3,ATRX,TMEM67 |
| **GO:0032508** | DNA duplex unwinding | 3 | 53 | 0.0207 | BRIP1,ATRX,RECQL4 |
| **GO:0090068** | positive regulation of cell cycle process | 6 | 273 | 0.0207 | CDC6,HYAL1,GATA6,RAD21,CDT1,ATRX |
| **GO:0032870** | cellular response to hormone stimulus | 9 | 585 | 0.0211 | CDC6,BRIP1,STAT3,GATA6,WT1,PTPN11,ARID1B,NSMF,MED12 |
| **GO:0042659** | regulation of cell fate specification | 2 | 14 | 0.0211 | DUSP6,BMPR1A |
| **GO:0070102** | interleukin-6-mediated signaling pathway | 2 | 14 | 0.0211 | STAT3,PTPN11 |
| **GO:0045765** | regulation of angiogenesis | 6 | 277 | 0.0216 | STAT3,HYAL1,GATA6,BMPER,PLXND1,HSPG2 |
| **GO:0048562** | embryonic organ morphogenesis | 6 | 279 | 0.0223 | HYAL1,ALX4,MED12,GLI3,FBN2,STRA6 |
| **GO:0045787** | positive regulation of cell cycle | 7 | 376 | 0.0224 | CDC6,HYAL1,GATA6,RAD21,CDT1,PTPN11,ATRX |
| **GO:0010941** | regulation of cell death | 17 | 1638 | 0.0233 | WFS1,NOTCH2,MAP2K2,STAT3,GATA6,DUSP6,GDF6,BRAF,HPGD,UBB,WT1,ALX4,POX2,LMNA,NSMF,HSPG2,GLI3 |
| **GO:0034764** | positive regulation of transmembrane transport | 5 | 194 | 0.0233 | KCNQ1,BRAF,PTPN11,DMD,GPC3 |
| **GO:0070198** | protein localization to chromosome, telomeric region | 2 | 15 | 0.0233 | ATR,ATRX |
| **GO:0071214** | cellular response to abiotic stimulus | 6 | 282 | 0.0233 | HYAL1,PTPN11,ATR,NSMF,TRPV4,PIEZO2 |
| **GO:0048871** | multicellular organismal homeostasis | 6 | 283 | 0.0235 | WFS1,STAT3,ANKRD11,PTPN11,TRPV4,FTO |
| **GO:0051052** | regulation of DNA metabolic process | 7 | 381 | 0.0236 | CDC6,GMNN,CDT1,ATR,SMC3,LMNA,ATRX |
| **GO:0071900** | regulation of protein serine/threonine kinase activity | 8 | 488 | 0.0241 | CDC6,MAP2K2,DUSP6,BRAF,UBB,PTPN11,ACTB,ZEB2 |
| **GO:0120035** | regulation of plasma membrane bounded cell projection organization | 9 | 600 | 0.0241 | PLXND1,DMD,L1CAM,NSMF,LRP4,CDKL5,SEPT9,TRPV4,ZEB2 |
| **GO:0034613** | cellular protein localization | 15 | 1367 | 0.0243 | CD4,LTBP2,STAT3,BUB1B,BRAF,RAD21,UBB,CEP57,RERE,ATR,DMD,LMNA,ATRX,LRP4,PEX5 |
| **GO:0034654** | nucleobase-containing compound biosynthetic process | 26 | 3031 | 0.0250 | POLR3B,SLC35D1,NOTCH2,NOTCH3,STAT3,GATA6,MED13L,KAT6B,RAD21,KMT2D,UBB,SHMT1,POLE,WT1,ALX4,RERE,ARID1B,SOX3,POLR3A,POLR1C,ATRX,MED12,GLI3,FOXP2,SMARCA4,ZEB2 |
| **GO:0072006** | nephron development | 4 | 121 | 0.0251 | NOTCH3,WT1,GPC3,GLI3 |
| **GO:0000076** | DNA replication checkpoint | 2 | 16 | 0.0252 | CDC6,CDT1 |
| **GO:0003184** | pulmonary valve morphogenesis | 2 | 16 | 0.0252 | NOTCH2,STRA6 |
| **GO:0009101** | glycoprotein biosynthetic process | 6 | 289 | 0.0252 | SLC35D1,CHSY1,XYLT1,B3GAT3,POMGNT2,POMGNT1 |
| **GO:0009950** | dorsal/ventral axis specification | 2 | 16 | 0.0252 | BMPR1A,LRP4 |
| **GO:0030214** | hyaluronan catabolic process | 2 | 16 | 0.0252 | HYAL1,GUSB |
| **GO:0044271** | cellular nitrogen compound biosynthetic process | 29 | 3528 | 0.0252 | POLR3B,SLC35D1,NOTCH2,NOTCH3,STAT3,GATA6,MED13L,KAT6B,RAD21,KMT2D,UBB,SHMT1,POLE,WT1,ALX4,RERE,ARID1B,DMD,SOX3,POLR3A,POLR1C,ATRX,MED12,PSAT1,GLI3,FOXP2,EARS2,SMARCA4,ZEB2 |
| **GO:0065003** | protein-containing complex assembly | 16 | 1514 | 0.0252 | GMNN,KAT6B,CDT1,KMT2D,UBB,CEP57,SHMT1,COL7A1,DMD,ATRX,LRP4,FANCA,SEPT9,PEX5,SMARCA4,TRPV4 |
| **GO:0090335** | regulation of brown fat cell differentiation | 2 | 16 | 0.0252 | TRPV4,FTO |
| **GO:0000082** | G1/S transition of mitotic cell cycle | 4 | 123 | 0.0258 | CDC6,GMNN,CDT1,POLE |
| **GO:0000819** | sister chromatid segregation | 4 | 123 | 0.0258 | BUB1B,CDT1,CEP57,SMC3 |
| **GO:0043010** | camera-type eye development | 6 | 292 | 0.0259 | WT1,GLI3,FOXP2,SMARCA4,FBN2,STRA6 |
| **GO:0030111** | regulation of Wnt signaling pathway | 6 | 293 | 0.0262 | MED12,LRP4,GPC3,GLI3,SMARCA4,ZEB2 |
| **GO:0071230** | cellular response to amino acid stimulus | 3 | 60 | 0.0262 | COL3A1,NSMF,COL5A2 |
| **GO:0043583** | ear development | 5 | 204 | 0.0268 | KCNQ1,BMPER,PTPN11,GLI3,STRA6 |
| **GO:0009070** | serine family amino acid biosynthetic process | 2 | 17 | 0.0270 | SHMT1,PSAT1 |
| **GO:0031175** | neuron projection development | 9 | 616 | 0.0270 | TBC1D24,UBB,RERE,PTPN11,ARID1B,L1CAM,LRP4,GLI3,ZEB2 |
| **GO:0048732** | gland development | 7 | 395 | 0.0270 | GATA6,PLXND1,WT1,SOX3,BMPR1A,GLI3,STRA6 |
| **GO:0045665** | negative regulation of neuron differentiation | 5 | 205 | 0.0271 | NOTCH3,SOX3,LRP4,GLI3,TRPV4 |
| **GO:0072594** | establishment of protein localization to organelle | 7 | 396 | 0.0271 | STAT3,UBB,CEP57,RERE,ATR,LMNA,PEX5 |
| **GO:0010648** | negative regulation of cell communication | 14 | 1255 | 0.0272 | WFS1,NOTCH3,DUSP6,BRAF,BMPER,UBB,PTPN11,LMNA,MED12,LRP4,GPC3,GLI3,SMARCA4,FBN2 |
| **GO:0048660** | regulation of smooth muscle cell proliferation | 4 | 126 | 0.0273 | NOTCH3,HPGD,COMT,BMPR1A |
| **GO:0023057** | negative regulation of signaling | 14 | 1258 | 0.0276 | WFS1,NOTCH3,DUSP6,BRAF,BMPER,UBB,PTPN11,LMNA,MED12,LRP4,GPC3,GLI3,SMARCA4,FBN2 |
| **GO:0060562** | epithelial tube morphogenesis | 6 | 298 | 0.0277 | PLXND1,WT1,MED12,GPC3,GLI3,ZEB2 |
| **GO:1901362** | organic cyclic compound biosynthetic process | 27 | 3230 | 0.0277 | POLR3B,SLC35D1,NOTCH2,NOTCH3,STAT3,GATA6,MED13L,KAT6B,RAD21,KMT2D,UBB,SHMT1,POLE,WT1,ALX4,RERE,ARID1B,SOX3,POLR3A,POLR1C,ATRX,MED12,PSAT1,GLI3,FOXP2,SMARCA4,ZEB2 |
| **GO:2000027** | regulation of animal organ morphogenesis | 5 | 207 | 0.0278 | WT1,BMPR1A,MED12,GPC3,FOXP2 |
| **GO:0001756** | somitogenesis | 3 | 63 | 0.0289 | BMPR1A,MED12,ZEB2 |
| **GO:0007221** | positive regulation of transcription of Notch receptor target | 2 | 18 | 0.0289 | NOTCH3,PLXND1 |
| **GO:0060393** | regulation of pathway-restricted SMAD protein phosphorylation | 3 | 63 | 0.0289 | GDF6,BMPER,BMPR1A |
| **GO:0014070** | response to organic cyclic compound | 11 | 873 | 0.0295 | CD4,KCNQ1,STAT3,DUSP6,HPGD,SHMT1,WT1,ACTB,COMT,MED12,FOXP2 |
| **GO:0006352** | DNA-templated transcription, initiation | 5 | 213 | 0.0306 | NOTCH2,NOTCH3,POLR1C,MED12,SMARCA4 |
| **GO:0008284** | positive regulation of cell population proliferation | 11 | 878 | 0.0306 | CD4,CDC6,NOTCH3,STAT3,HYAL1,GATA6,HPGD,KMT2D,BMPR1A,GLI3,FOXP2 |
| **GO:0051129** | negative regulation of cellular component organization | 9 | 632 | 0.0306 | BUB1B,RAD21,LMNA,ATRX,HSPG2,LRP4,TMEM67,PEX5,TRPV4 |
| **GO:1901992** | positive regulation of mitotic cell cycle phase transition | 3 | 65 | 0.0307 | CDC6,HYAL1,CDT1 |
| **GO:0032332** | positive regulation of chondrocyte differentiation | 2 | 19 | 0.0313 | GDF6,GLI3 |
| **GO:0048729** | tissue morphogenesis | 8 | 522 | 0.0318 | COL3A1,PLXND1,WT1,BMPR1A,MED12,GPC3,GLI3,ZEB2 |
| **GO:1903706** | regulation of hemopoiesis | 7 | 412 | 0.0318 | CD4,NOTCH2,STAT3,FANCD2,KMT2D,FANCA,GLI3 |
| **GO:0048754** | branching morphogenesis of an epithelial tube | 4 | 134 | 0.0321 | PLXND1,WT1,GPC3,GLI3 |
| **GO:0051053** | negative regulation of DNA metabolic process | 4 | 134 | 0.0321 | CDC6,GMNN,ATR,SMC3 |
| **GO:0002521** | leukocyte differentiation | 6 | 313 | 0.0332 | CD4,NOTCH2,STAT3,FAM20C,GPC3,GLI3 |
| **GO:0051050** | positive regulation of transport | 11 | 892 | 0.0333 | CD4,KCNQ1,WFS1,B3GAT3,BRAF,PTPN11,ACTB,DMD,GPC3,GLI3,TRPV4 |
| **GO:0048609** | multicellular organismal reproductive process | 10 | 766 | 0.0334 | BRIP1,FANCD2,HPGD,KMT2D,CEP57,WT1,PTPN11,COMT,ATRX,FANCA |
| **GO:0060341** | regulation of cellular localization | 10 | 766 | 0.0334 | CD4,MAP2K2,B3GAT3,CDT1,PTPN11,ACTB,DMD,LMNA,LRP4,GLI3 |
| **GO:2000104** | negative regulation of DNA-dependent DNA replication | 2 | 20 | 0.0334 | GMNN,SMC3 |
| **GO:0009968** | negative regulation of signal transduction | 13 | 1160 | 0.0338 | WFS1,NOTCH3,DUSP6,BRAF,BMPER,UBB,LMNA,MED12,LRP4,GPC3,GLI3,SMARCA4,FBN2 |
| **GO:0006996** | organelle organization | 26 | 3131 | 0.0340 | BRIP1,STAT3,KAT6B,BUB1B,FANCD2,RAD21,CDT1,KMT2D,UBB,CEP57,COL7A1,RERE,ARID1B,ACTB,SMC3,DMD,LMNA,ATRX,FANCA,TMEM67,PEX5,SMARCA4,TRPV4,TNXB,TBCE,RECQL4 |
| **GO:1901701** | cellular response to oxygen-containing compound | 11 | 896 | 0.0340 | KCNQ1,CDC6,BRIP1,STAT3,COL3A1,SHMT1,WT1,ARID1B,ACTB,NSMF,COL5A2 |
| **GO:0071456** | cellular response to hypoxia | 4 | 139 | 0.0354 | BRIP1,GATA6,UBB,LMNA |
| **GO:0120036** | plasma membrane bounded cell projection organization | 12 | 1034 | 0.0356 | TBC1D24,UBB,CEP57,RERE,PTPN11,ARID1B,DMD,L1CAM,LRP4,GLI3,TMEM67,ZEB2 |
| **GO:0007063** | regulation of sister chromatid cohesion | 2 | 21 | 0.0359 | RAD21,ATRX |
| **GO:0015012** | heparan sulfate proteoglycan biosynthetic process | 2 | 21 | 0.0359 | XYLT1,B3GAT3 |
| **GO:1901798** | positive regulation of signal transduction by p53 class mediator | 2 | 21 | 0.0359 | UBB,ATR |
| **GO:2000637** | positive regulation of gene silencing by miRNA | 2 | 21 | 0.0359 | MAP2K2,STAT3 |
| **GO:0003151** | outflow tract morphogenesis | 3 | 71 | 0.0368 | GATA6,PLXND1,BMPR1A |
| **GO:0006366** | transcription by RNA polymerase II | 10 | 784 | 0.0378 | NOTCH2,NOTCH3,STAT3,GATA6,RAD21,WT1,ALX4,MED12,GLI3,ZEB2 |
| **GO:0061351** | neural precursor cell proliferation | 3 | 72 | 0.0380 | RERE,GLI3,ZEB2 |
| **GO:0007530** | sex determination | 2 | 22 | 0.0384 | WT1,SOX3 |
| **GO:0045841** | negative regulation of mitotic metaphase/anaphase transition | 2 | 22 | 0.0384 | BUB1B,RAD21 |
| **GO:0045859** | regulation of protein kinase activity | 10 | 788 | 0.0384 | CD4,CDC6,MAP2K2,DUSP6,BRAF,UBB,PTPN11,DOK7,ACTB,ZEB2 |
| **GO:0061311** | cell surface receptor signaling pathway involved in heart development | 2 | 22 | 0.0384 | NOTCH2,BMPR1A |
| **GO:0098751** | bone cell development | 2 | 22 | 0.0384 | FAM20C,PTPN11 |
| **GO:1903010** | regulation of bone development | 2 | 22 | 0.0384 | NOTCH2,GLI3 |
| **GO:0045931** | positive regulation of mitotic cell cycle | 4 | 144 | 0.0385 | CDC6,HYAL1,CDT1,PTPN11 |
| **GO:0060411** | cardiac septum morphogenesis | 3 | 74 | 0.0401 | NOTCH2,GATA6,BMPR1A |
| **GO:0006297** | nucleotide-excision repair, DNA gap filling | 2 | 23 | 0.0407 | UBB,POLE |
| **GO:0021544** | subpallium development | 2 | 23 | 0.0407 | GLI3,FOXP2 |
| **GO:0030325** | adrenal gland development | 2 | 23 | 0.0407 | WT1,STRA6 |
| **GO:0051216** | cartilage development | 4 | 147 | 0.0407 | CHSY1,HYAL1,BMPR1A,TRPV4 |
| **GO:0008589** | regulation of smoothened signaling pathway | 3 | 75 | 0.0410 | CHSY1,GPC3,GLI3 |
| **GO:0021536** | diencephalon development | 3 | 75 | 0.0410 | SOX3,BMPR1A,ZEB2 |
| **GO:0048585** | negative regulation of response to stimulus | 15 | 1483 | 0.0417 | WFS1,NOTCH3,DUSP6,BRAF,BMPER,COL3A1,UBB,PTPN11,LMNA,MED12,LRP4,GPC3,GLI3,SMARCA4,FBN2 |
| **GO:0000187** | activation of MAPK activity | 4 | 149 | 0.0422 | MAP2K2,DUSP6,UBB,PTPN11 |
| **GO:0045666** | positive regulation of neuron differentiation | 6 | 337 | 0.0430 | GDF6,PLXND1,DMD,L1CAM,CDKL5,ZEB2 |
| **GO:0045596** | negative regulation of cell differentiation | 9 | 683 | 0.0440 | NOTCH3,STAT3,COL3A1,SOX3,BMPR1A,COL5A2,LRP4,GLI3,TRPV4 |
| **GO:0030182** | neuron differentiation | 11 | 940 | 0.0443 | NOTCH3,STAT3,TBC1D24,UBB,RERE,PTPN11,ARID1B,L1CAM,LRP4,GLI3,ZEB2 |
| **GO:0048812** | neuron projection morphogenesis | 7 | 448 | 0.0443 | UBB,RERE,PTPN11,L1CAM,LRP4,GLI3,ZEB2 |
| **GO:1903047** | mitotic cell cycle process | 8 | 564 | 0.0446 | CDC6,GMNN,BUB1B,CDT1,CEP57,POLE,LMNA,TBCE |
| **GO:0006351** | transcription, DNA-templated | 22 | 2569 | 0.0450 | POLR3B,NOTCH2,NOTCH3,STAT3,GATA6,MED13L,KAT6B,RAD21,KMT2D,WT1,ALX4,RERE,ARID1B,SOX3,POLR3A,POLR1C,ATRX,MED12,GLI3,FOXP2,SMARCA4,ZEB2 |
| **GO:0043436** | oxoacid metabolic process | 11 | 943 | 0.0450 | SLC35D1,CHSY1,XYLT1,B3GAT3,HYAL1,HPGD,GUSB,SHMT1,POX2,PSAT1,EARS2 |
| **GO:0042981** | regulation of apoptotic process | 15 | 1501 | 0.0451 | WFS1,NOTCH2,MAP2K2,STAT3,GATA6,DUSP6,GDF6,BRAF,HPGD,UBB,WT1,ALX4,LMNA,NSMF,GLI3 |
| **GO:0055007** | cardiac muscle cell differentiation | 3 | 79 | 0.0452 | GATA6,WT1,LMNA |
| **GO:0060045** | positive regulation of cardiac muscle cell proliferation | 2 | 25 | 0.0452 | GATA6,BMPR1A |
| **GO:0010001** | glial cell differentiation | 4 | 154 | 0.0453 | STAT3,PTPN11,MED12,GLI3 |
| **GO:0031346** | positive regulation of cell projection organization | 6 | 343 | 0.0453 | PLXND1,DMD,L1CAM,CDKL5,SEPT9,ZEB2 |
| **GO:0048738** | cardiac muscle tissue development | 4 | 154 | 0.0453 | GATA6,WT1,LMNA,BMPR1A |
| **GO:0065008** | regulation of biological quality | 28 | 3559 | 0.0453 | CD4,KCNQ1,WFS1,STAT3,GATA6,ANKRD11,COL3A1,UBB,PLXND1,SHMT1,PTPN11,ATR,COMT,SLC39A13,DMD,LMNA,L1CAM,NSMF,ATRX,LRP4,CDKL5,SMARCA4,TRPV4,FTO,PIEZO2,FBN2,RECQL4,NEB |
| **GO:1901135** | carbohydrate derivative metabolic process | 12 | 1083 | 0.0457 | SLC35D1,CHSY1,XYLT1,B3GAT3,HYAL1,GUSB,SHMT1,FAM20C,POMGNT2,POMGNT1,HSPG2,GPC3 |
| **GO:0010631** | epithelial cell migration | 3 | 80 | 0.0459 | PLXND1,PTPN11,ZEB2 |
| **GO:0030178** | negative regulation of Wnt signaling pathway | 4 | 155 | 0.0459 | MED12,LRP4,GPC3,GLI3 |
| **GO:0048661** | positive regulation of smooth muscle cell proliferation | 3 | 80 | 0.0459 | NOTCH3,HPGD,BMPR1A |
| **GO:0006338** | chromatin remodeling | 4 | 156 | 0.0465 | RERE,ARID1B,ATRX,SMARCA4 |
| **GO:0032204** | regulation of telomere maintenance | 3 | 81 | 0.0470 | ATR,LMNA,ATRX |
| **GO:0045589** | regulation of regulatory T cell differentiation | 2 | 26 | 0.0470 | FANCD2,FANCA |
| **GO:0035264** | multicellular organism growth | 3 | 82 | 0.0479 | ANKRD11,PTPN11,ATRX |
| **GO:1901565** | organonitrogen compound catabolic process | 11 | 958 | 0.0479 | WFS1,HYAL1,BUB1B,GUSB,UBB,SHMT1,POX2,COMT,HSPG2,GPC3,TMEM67 |
| **GO:1901990** | regulation of mitotic cell cycle phase transition | 6 | 351 | 0.0482 | CDC6,HYAL1,BUB1B,RAD21,CDT1,CEP57 |
| **GO:0001656** | metanephros development | 3 | 83 | 0.0491 | WT1,GPC3,GLI3 |
| **GO:0003148** | outflow tract septum morphogenesis | 2 | 27 | 0.0491 | GATA6,BMPR1A |
| **GO:0010976** | positive regulation of neuron projection development | 5 | 251 | 0.0491 | PLXND1,DMD,L1CAM,CDKL5,ZEB2 |
| **GO:0021846** | cell proliferation in forebrain | 2 | 27 | 0.0491 | GLI3,ZEB2 |
| **GO:2001252** | positive regulation of chromosome organization | 4 | 160 | 0.0491 | RAD21,CDT1,ATR,ATRX |
| **GO:0016070** | RNA metabolic process | 27 | 3430 | 0.0496 | POLR3B,NOTCH2,NOTCH3,STAT3,GATA6,MED13L,KAT6B,RAD21,KMT2D,POLE,DDX59,WT1,ALX4,RERE,ARID1B,TTC37,SOX3,POLR3A,POLR1C,ATRX,MED12,GLI3,FOXP2,EARS2,SMARCA4,FTO,ZEB2 |
| **GO:1903827** | regulation of cellular protein localization | 7 | 465 | 0.0496 | B3GAT3,CDT1,PTPN11,ACTB,LMNA,LRP4,GLI3 |
| **GO:0070252** | actin-mediated cell contraction | 3 | 84 | 0.0498 | KCNQ1,DMD,NEB |
