# Supplementary material for: Identification of Novel Variants in Cleft Palate-Associated Genes in Brazilian Patients With Non-syndromic Cleft Palate Only
Source: Front Cell Dev Biol. 2021 Jul 8;9:638522. doi: 10.3389/fcell.2021.638522 (PMC8297955; doi:10.3389/fcell.2021.638522)
Supplement: Supplementary file 9 [file Data_Sheet_7.docx]

| **Supplementary Table 7.** Activated pathways characterized with syndromic cleft palate only variant-containing-genes identified in patients with nonsyndromic cleft palate only (NSCPO). | | | | | |
| --- | --- | --- | --- | --- | --- |
| **Pathway ID** | **Term description** | **Observed gene count** | **Background gene count** | **False discovery rate** | **Matching proteins in your network** |
| **hsa04919** | Thyroid hormone signaling pathway | 7 | 115 | 0.00047 | NOTCH2,MAP2K2,NOTCH3,MED13L,ACTB,MED12,PLCB4 |
| **hsa04974** | Protein digestion and absorption | 6 | 90 | 0.00077 | KCNQ1,COL3A1,COL7A1,COL9A3,COL9A2,COL5A2 |
| **hsa05205** | Proteoglycans in cancer | 7 | 195 | 0.0041 | MAP2K2,STAT3,BRAF,PTPN11,ACTB,HSPG2,GPC3 |
| **hsa05206** | MicroRNAs in cancer | 6 | 149 | 0.0057 | NOTCH2,MAP2K2,NOTCH3,STAT3,TNXB,ZEB2 |
| **hsa03460** | Fanconi anemia pathway | 4 | 51 | 0.0059 | BRIP1,FANCD2,ATR,FANCA |
| **hsa00532** | Glycosaminoglycan biosynthesis - chondroitin sulfate / dermatan sulfate | 3 | 20 | 0.0060 | CHSY1,XYLT1,B3GAT3 |
| **hsa01100** | Metabolic pathways | 16 | 1250 | 0.0107 | POLR3B,CHSY1,XYLT1,B3GAT3,HYAL1,GUSB,SHMT1,POLE,POMGNT2,COMT,POMGNT1,POLR3A,POLR1C,PSAT1,PLCB4,EARS2 |
| **hsa04110** | Cell cycle | 5 | 123 | 0.0107 | CDC6,BUB1B,RAD21,ATR,SMC3 |
| **hsa05221** | Acute myeloid leukemia | 4 | 66 | 0.0107 | MAP2K2,STAT3,DUSP6,BRAF |
| **hsa03020** | RNA polymerase | 3 | 31 | 0.0116 | POLR3B,POLR3A,POLR1C |
| **hsa04512** | ECM-receptor interaction | 4 | 81 | 0.0142 | COL9A3,COL9A2,HSPG2,TNXB |
| **hsa05165** | Human papillomavirus infection | 7 | 317 | 0.0179 | NOTCH2,MAP2K2,NOTCH3,COL9A3,ATR,COL9A2,TNXB |
| **hsa01522** | Endocrine resistance | 4 | 95 | 0.0212 | NOTCH2,MAP2K2,NOTCH3,BRAF |
| **hsa05225** | Hepatocellular carcinoma | 5 | 163 | 0.0212 | MAP2K2,BRAF,ARID1B,ACTB,SMARCA4 |
| **hsa00240** | Pyrimidine metabolism | 4 | 100 | 0.0220 | POLR3B,POLE,POLR3A,POLR1C |
| **hsa04510** | Focal adhesion | 5 | 197 | 0.0386 | BRAF,COL9A3,ACTB,COL9A2,TNXB |
| **hsa04730** | Long-term depression | 3 | 60 | 0.0409 | MAP2K2,BRAF,PLCB4 |
| **hsa04623** | Cytosolic DNA-sensing pathway | 3 | 62 | 0.0422 | POLR3B,POLR3A,POLR1C |
| **hsa04720** | Long-term potentiation | 3 | 64 | 0.0435 | MAP2K2,BRAF,PLCB4 |
| **hsa00531** | Glycosaminoglycan degradation | 2 | 19 | 0.0439 | HYAL1,GUSB |
| **hsa05223** | Non-small cell lung cancer | 3 | 66 | 0.0439 | MAP2K2,STAT3,BRAF |
| **hsa05211** | Renal cell carcinoma | 3 | 68 | 0.0442 | MAP2K2,BRAF,PTPN11 |
| **hsa00515** | Mannose type O-glycan biosynthesis | 2 | 22 | 0.0492 | POMGNT2,POMGNT1 |
| **hsa00534** | Glycosaminoglycan biosynthesis - heparan sulfate / heparin | 2 | 24 | 0.0492 | XYLT1,B3GAT3 |
| **hsa01521** | EGFR tyrosine kinase inhibitor resistance | 3 | 78 | 0.0492 | MAP2K2,STAT3,BRAF |
| **hsa04934** | Cushing's syndrome | 4 | 153 | 0.0492 | MAP2K2,BRAF,KMT2D,PLCB4 |
| **hsa04971** | Gastric acid secretion | 3 | 72 | 0.0492 | KCNQ1,ACTB,PLCB4 |
| **hsa05220** | Chronic myeloid leukemia | 3 | 76 | 0.0492 | MAP2K2,BRAF,PTPN11 |
| **hsa05224** | Breast cancer | 4 | 147 | 0.0492 | NOTCH2,MAP2K2,NOTCH3,BRAF |
| **hsa05412** | Arrhythmogenic right ventricular cardiomyopathy (ARVC) | 3 | 72 | 0.0492 | ACTB,DMD,LMNA |
| **hsa05410** | Hypertrophic cardiomyopathy (HCM) | 3 | 81 | 0.0499 | ACTB,DMD,LMNA |
