# Supplementary material for: Identification of Novel Variants in Cleft Palate-Associated Genes in Brazilian Patients With Non-syndromic Cleft Palate Only
Source: Front Cell Dev Biol. 2021 Jul 8;9:638522. doi: 10.3389/fcell.2021.638522 (PMC8297955; doi:10.3389/fcell.2021.638522)
Supplement: Supplementary file 10 [file Data_Sheet_8.docx]

| **Supplementary Table 8.** Biological processes characterized with the list of altered genes in patients with nonsyndromic cleft palate only (NSCPO) from cleft lip-palate-associated genes. | | | | | |
| --- | --- | --- | --- | --- | --- |
| **GO ID** | **Term description** | **Observed gene count** | **Background gene count** | **False discovery rate** | **Matching proteins in your network** |
| **GO:0048513** | animal organ development | 56 | 2926 | 2.24e-20 | TNFRSF1A,KRT14,MYH9,MMP2,DHODH,TGFB1,FKTN,TTC21B,JAG1,EDAR,EP300,WDPCP,EGFR,NOTCH1,ATM,TDGF1,IFT122,COL1A2,TSC1,FUZ,WDR35,FBN1,FRAS1,PTCH1,FGFR3,CTNNB1,LARGE,RET,RYR1,SMOC1,ACTA1,AHI1,PTCH2,FGD1,ASXL1,ROR2,FOXH1,KDM6A,SKI,BRCA2,CDON,ACTN1,BMP7,DYNC2H1,TBC1D32,PAX1,CTNND1,WDR60,CC2D2A,PAX6,ESR1,FLNB,PIK3R1,KMT2A,CEP290,MYH3 |
| **GO:0009790** | embryo development | 35 | 890 | 4.90e-20 | MYH9,MMP2,TGFB1,EP300,WDPCP,EGFR,NOTCH1,ATM,TDGF1,IFT122,TSC1,FUZ,FBN1,FRAS1,PTCH1,CTNNB1,RET,AHI1,ROR2,FOXH1,KDM6A,SKI,BRCA2,CDON,BMP7,DYNC2H1,TBC1D32,PAX1,WDR60,CC2D2A,PAX6,BRCA1,KMT2A,CEP290,MYH3 |
| **GO:0009653** | anatomical structure morphogenesis | 47 | 1992 | 8.04e-20 | MYH9,MMP2,TGFB1,JAG1,EDAR,EP300,WDPCP,EGFR,NOTCH1,ATM,TDGF1,IFT122,COL1A2,TSC1,FUZ,FBN1,FRAS1,PTCH1,FGFR3,CTNNB1,RET,RYR1,ACTA1,AHI1,STXBP1,FGD1,ASXL1,ROR2,FOXH1,KDM6A,SKI,RPGRIP1L,CDON,ACTN1,BMP7,DYNC2H1,TBC1D32,PAX1,WDR60,CC2D2A,PAX6,ESR1,FLNB,PIK3R1,CEP290,ACTG1,MYH3 |
| **GO:0009887** | animal organ morphogenesis | 32 | 865 | 1.83e-17 | MMP2,TGFB1,JAG1,EDAR,EP300,WDPCP,EGFR,NOTCH1,IFT122,COL1A2,FUZ,FBN1,FRAS1,PTCH1,FGFR3,CTNNB1,RYR1,ACTA1,AHI1,FGD1,ASXL1,ROR2,FOXH1,KDM6A,SKI,CDON,BMP7,PAX1,WDR60,PAX6,ESR1,CEP290 |
| **GO:0048598** | embryonic morphogenesis | 26 | 545 | 3.12e-16 | MMP2,TGFB1,WDPCP,NOTCH1,IFT122,TSC1,FUZ,FBN1,FRAS1,PTCH1,CTNNB1,RET,AHI1,ROR2,FOXH1,KDM6A,SKI,CDON,BMP7,DYNC2H1,TBC1D32,WDR60,CC2D2A,PAX6,CEP290,MYH3 |
| **GO:0048646** | anatomical structure formation involved in morphogenesis | 30 | 831 | 5.00e-16 | MYH9,MMP2,TGFB1,JAG1,EDAR,EP300,NOTCH1,ATM,TDGF1,IFT122,TSC1,FUZ,PTCH1,CTNNB1,RET,ACTA1,AHI1,ROR2,FOXH1,KDM6A,SKI,CDON,ACTN1,BMP7,PAX1,CC2D2A,PAX6,CEP290,ACTG1,MYH3 |
| **GO:0048731** | system development | 58 | 4144 | 1.55e-15 | TNFRSF1A,KRT14,MYH9,MMP2,DHODH,TGFB1,FKTN,TTC21B,JAG1,EDAR,SMARCB1,EP300,WDPCP,EGFR,NOTCH1,ATM,TDGF1,IFT122,COL1A2,TSC1,FUZ,WDR35,FBN1,FRAS1,PTCH1,FGFR3,CTNNB1,LARGE,RET,RYR1,SMOC1,ACTA1,AHI1,PTCH2,STXBP1,FGD1,ASXL1,ROR2,FOXH1,KDM6A,SKI,BRCA2,CDON,ACTN1,BMP7,DYNC2H1,TBC1D32,PAX1,CTNND1,WDR60,CC2D2A,PAX6,ESR1,FLNB,PIK3R1,KMT2A,CEP290,MYH3 |
| **GO:0007275** | multicellular organism development | 61 | 4726 | 3.74e-15 | TNFRSF1A,KRT14,MYH9,MMP2,DHODH,TGFB1,FKTN,TTC21B,JAG1,EDAR,SMARCB1,EP300,WDPCP,EGFR,NOTCH1,ATM,TDGF1,IFT122,COL1A2,TSC1,FUZ,WDR35,FBN1,FRAS1,PTCH1,FGFR3,CTNNB1,LARGE,RET,RYR1,SMOC1,ACTA1,AHI1,PTCH2,POMT1,STXBP1,FGD1,ASXL1,ROR2,FOXH1,KDM6A,SKI,BRCA2,CDON,ACTN1,BMP7,DYNC2H1,TBC1D32,PAX1,CTNND1,WDR60,CC2D2A,PAX6,ESR1,COLEC11,BRCA1,FLNB,PIK3R1,KMT2A,CEP290,MYH3 |
| **GO:0009888** | tissue development | 38 | 1626 | 3.74e-15 | KRT14,MMP2,TGFB1,JAG1,EDAR,EP300,WDPCP,EGFR,NOTCH1,ATM,IFT122,TSC1,FUZ,FRAS1,PTCH1,FGFR3,CTNNB1,LARGE,RET,RYR1,ACTA1,AHI1,PTCH2,ASXL1,ROR2,FOXH1,KDM6A,SKI,RPGRIP1L,CDON,BMP7,TBC1D32,PAX1,CC2D2A,PAX6,ESR1,FLNB,CEP290 |
| **GO:0048856** | anatomical structure development | 63 | 5085 | 3.74e-15 | TNFRSF1A,KRT14,MYH9,MMP2,DHODH,TGFB1,FKTN,TTC21B,JAG1,EDAR,SMARCB1,EP300,WDPCP,EGFR,NOTCH1,ATM,TDGF1,IFT122,COL1A2,TSC1,FUZ,WDR35,FBN1,FRAS1,PTCH1,FGFR3,CTNNB1,LARGE,RET,RYR1,SMOC1,ACTA1,AHI1,PTCH2,POMT1,STXBP1,FGD1,ASXL1,ROR2,FOXH1,KDM6A,SKI,RPGRIP1L,BRCA2,CDON,ACTN1,BMP7,DYNC2H1,TBC1D32,PAX1,CTNND1,WDR60,CC2D2A,PAX6,ESR1,COLEC11,BRCA1,FLNB,PIK3R1,KMT2A,CEP290,ACTG1,MYH3 |
| **GO:0060429** | epithelium development | 31 | 1055 | 1.77e-14 | KRT14,TGFB1,JAG1,EDAR,EP300,WDPCP,EGFR,NOTCH1,ATM,IFT122,TSC1,FUZ,FRAS1,PTCH1,CTNNB1,RET,AHI1,PTCH2,ROR2,FOXH1,KDM6A,SKI,RPGRIP1L,BMP7,TBC1D32,PAX1,CC2D2A,PAX6,ESR1,FLNB,CEP290 |
| **GO:0007389** | pattern specification process | 21 | 409 | 1.26e-13 | TTC21B,EP300,NOTCH1,ATM,TDGF1,IFT122,PTCH1,CTNNB1,AHI1,ROR2,FOXH1,KDM6A,SKI,CDON,BMP7,DYNC2H1,TBC1D32,PAX1,CC2D2A,PAX6,KMT2A |
| **GO:0003002** | regionalization | 19 | 313 | 1.94e-13 | TTC21B,EP300,NOTCH1,ATM,TDGF1,IFT122,PTCH1,CTNNB1,AHI1,ROR2,FOXH1,KDM6A,SKI,CDON,DYNC2H1,TBC1D32,PAX1,PAX6,KMT2A |
| **GO:0009792** | embryo development ending in birth or egg hatching | 23 | 568 | 4.81e-13 | MYH9,TGFB1,EP300,EGFR,NOTCH1,ATM,TDGF1,IFT122,TSC1,FUZ,PTCH1,CTNNB1,ROR2,KDM6A,SKI,BRCA2,BMP7,TBC1D32,PAX1,WDR60,CC2D2A,PAX6,BRCA1 |
| **GO:0035295** | tube development | 26 | 793 | 6.08e-13 | MYH9,MMP2,TGFB1,JAG1,EDAR,EP300,EGFR,NOTCH1,TDGF1,IFT122,TSC1,FUZ,PTCH1,CTNNB1,RET,AHI1,ASXL1,FOXH1,KDM6A,SKI,BMP7,TBC1D32,CC2D2A,PAX6,ESR1,CEP290 |
| **GO:0072359** | circulatory system development | 26 | 807 | 8.54e-13 | TNFRSF1A,MYH9,MMP2,TGFB1,JAG1,EP300,WDPCP,NOTCH1,ATM,TDGF1,IFT122,COL1A2,TSC1,FBN1,PTCH1,CTNNB1,RYR1,AHI1,ASXL1,FOXH1,KDM6A,BMP7,DYNC2H1,TBC1D32,CC2D2A,PAX6 |
| **GO:0002009** | morphogenesis of an epithelium | 20 | 414 | 1.50e-12 | TGFB1,JAG1,EGFR,NOTCH1,IFT122,TSC1,FUZ,FRAS1,PTCH1,CTNNB1,RET,AHI1,FOXH1,KDM6A,SKI,RPGRIP1L,BMP7,CC2D2A,ESR1,CEP290 |
| **GO:0007507** | heart development | 21 | 485 | 2.17e-12 | TNFRSF1A,TGFB1,JAG1,EP300,NOTCH1,ATM,TDGF1,IFT122,TSC1,FBN1,PTCH1,CTNNB1,RYR1,AHI1,ASXL1,FOXH1,KDM6A,BMP7,DYNC2H1,TBC1D32,CC2D2A |
| **GO:0043009** | chordate embryonic development | 22 | 550 | 2.17e-12 | MYH9,TGFB1,EP300,EGFR,NOTCH1,ATM,IFT122,TSC1,FUZ,PTCH1,CTNNB1,ROR2,KDM6A,SKI,BRCA2,BMP7,TBC1D32,PAX1,WDR60,CC2D2A,PAX6,BRCA1 |
| **GO:0035148** | tube formation | 14 | 142 | 2.33e-12 | TGFB1,EDAR,NOTCH1,IFT122,TSC1,FUZ,PTCH1,CTNNB1,RET,KDM6A,SKI,BMP7,CC2D2A,CEP290 |
| **GO:0016043** | cellular component organization | 59 | 5163 | 3.51e-12 | TNFRSF1A,KRT14,MYH9,MMP2,TGFB1,TTC21B,APC,PHYH,SMARCB1,EP300,ANK1,WDPCP,EGFR,NOTCH1,ATM,IFT122,COL1A2,TSC1,TCTN2,FUZ,WDR35,FBN1,PTCH1,CTNNB1,RET,RYR1,HLA-DRB1,ACTA1,AHI1,RPS27,ABCA4,POMT1,STXBP1,RPL11,FGD1,ASXL1,KDM6A,SKI,RPGRIP1L,BRCA2,ADAMTS20,ACTN1,BMP7,DYNC2H1,TBC1D32,CTNND1,WDR60,TMEM237,FGD4,CC2D2A,PAX6,ESR1,BRCA1,FLNB,PIK3R1,KMT2A,CEP290,ACTG1,MYH3 |
| **GO:0071840** | cellular component organization or biogenesis | 60 | 5342 | 3.51e-12 | TNFRSF1A,KRT14,MYH9,MMP2,TGFB1,TTC21B,APC,PHYH,SMARCB1,EP300,ANK1,WDPCP,EGFR,NOTCH1,ATM,IFT122,COL1A2,TSC1,TCTN2,FUZ,WDR35,FBN1,PTCH1,RPS7,CTNNB1,RET,RYR1,HLA-DRB1,ACTA1,AHI1,RPS27,ABCA4,POMT1,STXBP1,RPL11,FGD1,ASXL1,KDM6A,SKI,RPGRIP1L,BRCA2,ADAMTS20,ACTN1,BMP7,DYNC2H1,TBC1D32,CTNND1,WDR60,TMEM237,FGD4,CC2D2A,PAX6,ESR1,BRCA1,FLNB,PIK3R1,KMT2A,CEP290,ACTG1,MYH3 |
| **GO:0048583** | regulation of response to stimulus | 51 | 3882 | 5.03e-12 | TNFRSF1A,MMP2,TGFB1,FKTN,TTC21B,JAG1,APC,EDAR,SMARCB1,EP300,ARHGAP31,EGFR,NOTCH1,ATM,CD96,TDGF1,IFT122,COL1A2,TSC1,FUZ,WDR35,FBN1,PTCH1,RPS7,FGFR3,CTNNB1,RET,HLA-DRB1,PTCH2,STXBP1,RPL11,FGD1,ASXL1,ROR2,FOXH1,SKI,RPGRIP1L,ADAMTS20,CDON,BMP7,DYNC2H1,CTNND1,TMEM237,FGD4,HLA-B,ESR1,COLEC11,BRCA1,PIK3R1,KMT2A,ACTG1 |
| **GO:0048729** | tissue morphogenesis | 21 | 522 | 6.58e-12 | TGFB1,JAG1,EGFR,NOTCH1,IFT122,TSC1,FUZ,FRAS1,PTCH1,CTNNB1,RET,ACTA1,AHI1,FOXH1,KDM6A,SKI,RPGRIP1L,BMP7,CC2D2A,ESR1,CEP290 |
| **GO:0035239** | tube morphogenesis | 22 | 615 | 1.42e-11 | MYH9,MMP2,TGFB1,JAG1,EDAR,EGFR,NOTCH1,TDGF1,IFT122,TSC1,FUZ,PTCH1,CTNNB1,RET,AHI1,FOXH1,KDM6A,SKI,BMP7,CC2D2A,ESR1,CEP290 |
| **GO:0048568** | embryonic organ development | 19 | 417 | 1.42e-11 | TGFB1,WDPCP,EGFR,NOTCH1,IFT122,FUZ,FBN1,PTCH1,CTNNB1,AHI1,ROR2,FOXH1,KDM6A,BMP7,WDR60,CC2D2A,PAX6,KMT2A,CEP290 |
| **GO:0032501** | multicellular organismal process | 65 | 6507 | 1.68e-11 | TNFRSF1A,KRT14,MYH9,MMP2,DHODH,TGFB1,FKTN,TTC21B,JAG1,EDAR,SMARCB1,EP300,WDPCP,EGFR,NOTCH1,ATM,CD96,TDGF1,IFT122,COL1A2,TSC1,FUZ,WDR35,FBN1,FRAS1,PTCH1,FGFR3,CTNNB1,LARGE,RET,RYR1,SMOC1,ACTA1,AHI1,ABCA4,PTCH2,POMT1,NUP188,STXBP1,FGD1,ASXL1,ROR2,FOXH1,KDM6A,SKI,BRCA2,CHRNG,CDON,ACTN1,BMP7,DYNC2H1,TBC1D32,PAX1,CTNND1,WDR60,CC2D2A,PAX6,ESR1,COLEC11,BRCA1,FLNB,PIK3R1,KMT2A,CEP290,MYH3 |
| **GO:0008104** | protein localization | 36 | 1966 | 1.82e-11 | TNFRSF1A,MYH9,TGFB1,TTC21B,PHYH,ANK1,WDPCP,EGFR,NOTCH1,ATM,IFT122,TSC1,FUZ,WDR35,FBN1,FRAS1,PTCH1,RPS7,CTNNB1,RPS26,AHI1,RPS27,NUP188,STXBP1,RPL11,BRCA2,BMP7,DYNC2H1,TBC1D32,WDR60,CC2D2A,PAX6,ESR1,FLNB,PIK3R1,CEP290 |
| **GO:0030030** | cell projection organization | 27 | 1067 | 3.68e-11 | MYH9,TTC21B,WDPCP,EGFR,NOTCH1,IFT122,TSC1,TCTN2,FUZ,WDR35,PTCH1,CTNNB1,RET,AHI1,STXBP1,FGD1,RPGRIP1L,BMP7,DYNC2H1,TBC1D32,WDR60,TMEM237,FGD4,CC2D2A,PAX6,PIK3R1,CEP290 |
| **GO:0023051** | regulation of signaling | 46 | 3360 | 4.59e-11 | TNFRSF1A,TGFB1,FKTN,TTC21B,JAG1,APC,EDAR,SMARCB1,EP300,ARHGAP31,EGFR,NOTCH1,ATM,TDGF1,IFT122,TSC1,FUZ,WDR35,FBN1,PTCH1,RPS7,FGFR3,CTNNB1,RET,RYR1,HLA-DRB1,PTCH2,STXBP1,RPL11,FGD1,ASXL1,ROR2,FOXH1,SKI,RPGRIP1L,ADAMTS20,CDON,BMP7,DYNC2H1,CTNND1,TMEM237,FGD4,ESR1,BRCA1,PIK3R1,KMT2A |
| **GO:0001654** | eye development | 17 | 339 | 6.28e-11 | TGFB1,JAG1,WDPCP,EGFR,IFT122,FBN1,CTNNB1,RET,SMOC1,AHI1,SKI,CDON,BMP7,TBC1D32,CC2D2A,PAX6,CEP290 |
| **GO:0060562** | epithelial tube morphogenesis | 16 | 298 | 1.10e-10 | TGFB1,NOTCH1,IFT122,TSC1,FUZ,PTCH1,CTNNB1,RET,AHI1,FOXH1,KDM6A,SKI,BMP7,CC2D2A,ESR1,CEP290 |
| **GO:0120036** | plasma membrane bounded cell projection organization | 26 | 1034 | 1.10e-10 | MYH9,TTC21B,WDPCP,EGFR,NOTCH1,IFT122,TCTN2,FUZ,WDR35,PTCH1,CTNNB1,RET,AHI1,STXBP1,FGD1,RPGRIP1L,BMP7,DYNC2H1,TBC1D32,WDR60,TMEM237,FGD4,CC2D2A,PAX6,PIK3R1,CEP290 |
| **GO:0009966** | regulation of signal transduction | 43 | 3033 | 1.21e-10 | TNFRSF1A,TGFB1,FKTN,TTC21B,JAG1,APC,EDAR,SMARCB1,EP300,ARHGAP31,EGFR,NOTCH1,ATM,TDGF1,IFT122,TSC1,FUZ,WDR35,FBN1,PTCH1,RPS7,FGFR3,CTNNB1,RET,HLA-DRB1,PTCH2,RPL11,FGD1,ASXL1,ROR2,FOXH1,SKI,RPGRIP1L,ADAMTS20,CDON,BMP7,DYNC2H1,CTNND1,TMEM237,FGD4,ESR1,BRCA1,PIK3R1 |
| **GO:0010646** | regulation of cell communication | 45 | 3327 | 1.27e-10 | TNFRSF1A,TGFB1,FKTN,TTC21B,JAG1,APC,EDAR,SMARCB1,EP300,ARHGAP31,EGFR,NOTCH1,ATM,TDGF1,IFT122,TSC1,FUZ,WDR35,FBN1,PTCH1,RPS7,FGFR3,CTNNB1,RET,HLA-DRB1,PTCH2,STXBP1,RPL11,FGD1,ASXL1,ROR2,FOXH1,SKI,RPGRIP1L,ADAMTS20,CDON,BMP7,DYNC2H1,CTNND1,TMEM237,FGD4,ESR1,BRCA1,PIK3R1,KMT2A |
| **GO:0030326** | embryonic limb morphogenesis | 12 | 126 | 1.58e-10 | WDPCP,NOTCH1,IFT122,FRAS1,PTCH1,CTNNB1,ROR2,SKI,BMP7,DYNC2H1,TBC1D32,MYH3 |
| **GO:0033036** | macromolecule localization | 37 | 2268 | 1.65e-10 | TNFRSF1A,MYH9,TGFB1,TTC21B,PHYH,ANK1,WDPCP,EGFR,NOTCH1,ATM,IFT122,TSC1,FUZ,WDR35,FBN1,FRAS1,PTCH1,RPS7,CTNNB1,RPS26,AHI1,RPS27,ABCA4,NUP188,STXBP1,RPL11,BRCA2,BMP7,DYNC2H1,TBC1D32,WDR60,CC2D2A,PAX6,ESR1,FLNB,PIK3R1,CEP290 |
| **GO:0072175** | epithelial tube formation | 12 | 128 | 1.75e-10 | TGFB1,IFT122,TSC1,FUZ,PTCH1,CTNNB1,RET,KDM6A,SKI,BMP7,CC2D2A,CEP290 |
| **GO:0034613** | cellular protein localization | 29 | 1367 | 2.00e-10 | TNFRSF1A,TGFB1,TTC21B,PHYH,ANK1,EGFR,ATM,IFT122,TSC1,WDR35,PTCH1,RPS7,CTNNB1,RPS26,AHI1,RPS27,NUP188,STXBP1,RPL11,BRCA2,BMP7,DYNC2H1,TBC1D32,WDR60,CC2D2A,PAX6,ESR1,FLNB,PIK3R1 |
| **GO:0060173** | limb development | 13 | 174 | 2.73e-10 | WDPCP,NOTCH1,IFT122,FRAS1,PTCH1,CTNNB1,SMOC1,ROR2,SKI,BMP7,DYNC2H1,TBC1D32,MYH3 |
| **GO:0007423** | sensory organ development | 19 | 515 | 2.88e-10 | TGFB1,JAG1,WDPCP,EGFR,NOTCH1,IFT122,FBN1,CTNNB1,RET,SMOC1,AHI1,ROR2,SKI,CDON,BMP7,TBC1D32,CC2D2A,PAX6,CEP290 |
| **GO:0007224** | smoothened signaling pathway | 10 | 72 | 3.29e-10 | TTC21B,WDPCP,IFT122,TCTN2,PTCH1,ROR2,CDON,TBC1D32,CC2D2A,PAX6 |
| **GO:0043010** | camera-type eye development | 15 | 292 | 7.51e-10 | TGFB1,JAG1,WDPCP,EGFR,IFT122,FBN1,CTNNB1,RET,AHI1,SKI,CDON,BMP7,TBC1D32,CC2D2A,PAX6 |
| **GO:0120031** | plasma membrane bounded cell projection assembly | 17 | 413 | 7.85e-10 | TTC21B,WDPCP,NOTCH1,IFT122,TCTN2,FUZ,WDR35,AHI1,FGD1,RPGRIP1L,DYNC2H1,TBC1D32,WDR60,TMEM237,FGD4,CC2D2A,CEP290 |
| **GO:0009952** | anterior/posterior pattern specification | 13 | 197 | 9.89e-10 | EP300,NOTCH1,ATM,TDGF1,CTNNB1,ROR2,FOXH1,KDM6A,SKI,CDON,PAX1,PAX6,KMT2A |
| **GO:0021915** | neural tube development | 12 | 154 | 1.01e-09 | TGFB1,NOTCH1,IFT122,TSC1,FUZ,PTCH1,KDM6A,SKI,BMP7,TBC1D32,CC2D2A,PAX6 |
| **GO:0010628** | positive regulation of gene expression | 32 | 1826 | 1.15e-09 | TNFRSF1A,MYH9,TGFB1,JAG1,EDAR,SMARCB1,EP300,EGFR,NOTCH1,PTCH1,RPS7,CTNNB1,RET,HLA-DRB1,ACTA1,AHI1,RPL11,ASXL1,ROR2,FOXH1,KDM6A,SKI,BRCA2,CDON,BMP7,PAX1,PAX6,ESR1,BRCA1,PIK3R1,KMT2A,CEP290 |
| **GO:0051179** | localization | 55 | 5233 | 1.15e-09 | TNFRSF1A,TCN2,MYH9,DHODH,TGFB1,TTC21B,APC,PHYH,ANK1,WDPCP,EGFR,NOTCH1,ATM,TDGF1,IFT122,COL1A2,TSC1,TCTN2,FUZ,WDR35,FBN1,FRAS1,PTCH1,RPS7,CTNNB1,RET,RPS26,SLC2A10,RYR1,AHI1,RPS27,ABCA4,NUP188,STXBP1,RPL11,ROR2,SKI,RPGRIP1L,BRCA2,CHRNG,ACTN1,BMP7,DYNC2H1,TBC1D32,WDR60,HLA-B,CC2D2A,PAX6,ESR1,COLEC11,FLNB,PIK3R1,CEP290,ACTG1,TMCO1 |
| **GO:0007166** | cell surface receptor signaling pathway | 35 | 2198 | 1.23e-09 | TNFRSF1A,MYH9,MMP2,TGFB1,TTC21B,JAG1,APC,EDAR,EP300,WDPCP,EGFR,NOTCH1,IFT122,COL1A2,TCTN2,PTCH1,FGFR3,CTNNB1,RET,HLA-DRB1,AHI1,ROR2,FOXH1,KDM6A,SKI,CHRNG,CDON,BMP7,TBC1D32,CTNND1,HLA-B,CC2D2A,PAX6,PIK3R1,ACTG1 |
| **GO:0001838** | embryonic epithelial tube formation | 11 | 120 | 1.37e-09 | TGFB1,IFT122,TSC1,FUZ,PTCH1,CTNNB1,RET,KDM6A,SKI,BMP7,CC2D2A |
| **GO:0022607** | cellular component assembly | 36 | 2343 | 1.47e-09 | TNFRSF1A,KRT14,TGFB1,TTC21B,APC,SMARCB1,EP300,WDPCP,NOTCH1,IFT122,COL1A2,TSC1,TCTN2,FUZ,WDR35,CTNNB1,RYR1,HLA-DRB1,ACTA1,AHI1,RPS27,RPL11,FGD1,SKI,RPGRIP1L,ACTN1,DYNC2H1,TBC1D32,WDR60,TMEM237,FGD4,CC2D2A,KMT2A,CEP290,ACTG1,MYH3 |
| **GO:0070925** | organelle assembly | 20 | 666 | 2.04e-09 | TTC21B,WDPCP,NOTCH1,IFT122,TCTN2,FUZ,WDR35,ACTA1,AHI1,RPS27,RPL11,RPGRIP1L,DYNC2H1,TBC1D32,WDR60,TMEM237,CC2D2A,CEP290,ACTG1,MYH3 |
| **GO:0010604** | positive regulation of macromolecule metabolic process | 41 | 3081 | 2.61e-09 | TNFRSF1A,MYH9,TGFB1,JAG1,APC,EDAR,POMT2,SMARCB1,EP300,EGFR,NOTCH1,ATM,TDGF1,WDR35,FBN1,PTCH1,RPS7,FGFR3,CTNNB1,RET,HLA-DRB1,ACTA1,AHI1,POMT1,RPL11,ASXL1,ROR2,FOXH1,KDM6A,SKI,BRCA2,CDON,ACTN1,BMP7,PAX1,PAX6,ESR1,BRCA1,PIK3R1,KMT2A,CEP290 |
| **GO:0060271** | cilium assembly | 15 | 326 | 2.69e-09 | TTC21B,WDPCP,NOTCH1,IFT122,TCTN2,FUZ,WDR35,AHI1,RPGRIP1L,DYNC2H1,TBC1D32,WDR60,TMEM237,CC2D2A,CEP290 |
| **GO:0061061** | muscle structure development | 17 | 457 | 2.94e-09 | MYH9,TGFB1,FKTN,JAG1,EP300,NOTCH1,TSC1,CTNNB1,LARGE,RYR1,ACTA1,FOXH1,SKI,CDON,FLNB,ACTG1,MYH3 |
| **GO:0050793** | regulation of developmental process | 36 | 2416 | 3.24e-09 | TNFRSF1A,MYH9,DHODH,TGFB1,JAG1,EP300,WDPCP,EGFR,NOTCH1,IFT122,TSC1,FUZ,FBN1,PTCH1,FGFR3,CTNNB1,RET,HLA-DRB1,SMOC1,AHI1,PTCH2,FGD1,ASXL1,ROR2,SKI,BRCA2,ADAMTS20,CDON,BMP7,FGD4,HLA-B,PAX6,ESR1,BRCA1,PIK3R1,KMT2A |
| **GO:0044085** | cellular component biogenesis | 37 | 2556 | 3.46e-09 | TNFRSF1A,KRT14,TGFB1,TTC21B,APC,SMARCB1,EP300,WDPCP,NOTCH1,IFT122,COL1A2,TSC1,TCTN2,FUZ,WDR35,RPS7,CTNNB1,RYR1,HLA-DRB1,ACTA1,AHI1,RPS27,RPL11,FGD1,SKI,RPGRIP1L,ACTN1,DYNC2H1,TBC1D32,WDR60,TMEM237,FGD4,CC2D2A,KMT2A,CEP290,ACTG1,MYH3 |
| **GO:0009893** | positive regulation of metabolic process | 42 | 3280 | 4.11e-09 | TNFRSF1A,MYH9,TGFB1,JAG1,APC,EDAR,POMT2,SMARCB1,EP300,EGFR,NOTCH1,ATM,TDGF1,TSC1,WDR35,FBN1,PTCH1,RPS7,FGFR3,CTNNB1,RET,HLA-DRB1,ACTA1,AHI1,POMT1,RPL11,ASXL1,ROR2,FOXH1,KDM6A,SKI,BRCA2,CDON,ACTN1,BMP7,PAX1,PAX6,ESR1,BRCA1,PIK3R1,KMT2A,CEP290 |
| **GO:0051641** | cellular localization | 34 | 2180 | 4.11e-09 | TNFRSF1A,MYH9,TGFB1,TTC21B,PHYH,ANK1,EGFR,ATM,IFT122,TSC1,TCTN2,WDR35,PTCH1,RPS7,CTNNB1,RPS26,RYR1,AHI1,RPS27,NUP188,STXBP1,RPL11,RPGRIP1L,BRCA2,BMP7,DYNC2H1,TBC1D32,WDR60,CC2D2A,PAX6,ESR1,FLNB,PIK3R1,CEP290 |
| **GO:0048518** | positive regulation of biological process | 55 | 5459 | 5.36e-09 | TNFRSF1A,MYH9,MMP2,DHODH,TGFB1,JAG1,APC,EDAR,POMT2,SMARCB1,EP300,ANK1,EGFR,NOTCH1,ATM,TDGF1,TSC1,FUZ,WDR35,FBN1,PTCH1,RPS7,FGFR3,CTNNB1,RET,HLA-DRB1,ACTA1,AHI1,PTCH2,POMT1,STXBP1,RPL11,FGD1,ASXL1,ROR2,FOXH1,KDM6A,SKI,BRCA2,ADAMTS20,CDON,ACTN1,BMP7,DYNC2H1,PAX1,FGD4,HLA-B,PAX6,ESR1,COLEC11,BRCA1,PIK3R1,KMT2A,CEP290,ACTG1 |
| **GO:0001655** | urogenital system development | 14 | 299 | 8.76e-09 | TGFB1,JAG1,WDPCP,NOTCH1,TSC1,FBN1,FRAS1,PTCH1,CTNNB1,RET,AHI1,BMP7,ESR1,CEP290 |
| **GO:0033365** | protein localization to organelle | 19 | 649 | 8.76e-09 | TGFB1,TTC21B,PHYH,ATM,IFT122,WDR35,RPS7,RPS26,RPS27,NUP188,RPL11,BRCA2,BMP7,DYNC2H1,TBC1D32,CC2D2A,PAX6,ESR1,PIK3R1 |
| **GO:0010557** | positive regulation of macromolecule biosynthetic process | 30 | 1758 | 9.03e-09 | TNFRSF1A,TGFB1,JAG1,POMT2,SMARCB1,EP300,EGFR,NOTCH1,ATM,PTCH1,CTNNB1,RET,HLA-DRB1,AHI1,POMT1,ASXL1,ROR2,FOXH1,SKI,BRCA2,CDON,ACTN1,BMP7,PAX1,PAX6,ESR1,BRCA1,PIK3R1,KMT2A,CEP290 |
| **GO:0010033** | response to organic substance | 38 | 2815 | 1.13e-08 | TNFRSF1A,MMP2,DHODH,TGFB1,JAG1,APC,EDAR,POMT2,EP300,EGFR,NOTCH1,CD96,TDGF1,COL1A2,TSC1,WDR35,FBN1,PTCH1,FGFR3,CTNNB1,RET,RYR1,HLA-DRB1,ACTA1,POMT1,STXBP1,ASXL1,ROR2,FOXH1,SKI,BMP7,HLA-B,PAX6,ESR1,BRCA1,FLNB,PIK3R1,ACTG1 |
| **GO:0001822** | kidney development | 13 | 251 | 1.22e-08 | TGFB1,JAG1,WDPCP,NOTCH1,TSC1,FBN1,FRAS1,PTCH1,CTNNB1,RET,AHI1,BMP7,CEP290 |
| **GO:0006996** | organelle organization | 40 | 3131 | 1.48e-08 | KRT14,MYH9,TTC21B,PHYH,SMARCB1,EP300,ANK1,WDPCP,NOTCH1,ATM,IFT122,TCTN2,FUZ,WDR35,CTNNB1,ACTA1,AHI1,RPS27,STXBP1,RPL11,FGD1,ASXL1,KDM6A,RPGRIP1L,BRCA2,ACTN1,DYNC2H1,TBC1D32,WDR60,TMEM237,FGD4,CC2D2A,PAX6,ESR1,BRCA1,FLNB,KMT2A,CEP290,ACTG1,MYH3 |
| **GO:0048584** | positive regulation of response to stimulus | 32 | 2054 | 1.68e-08 | TNFRSF1A,MMP2,TGFB1,JAG1,EDAR,SMARCB1,EP300,EGFR,NOTCH1,ATM,TDGF1,WDR35,RPS7,FGFR3,CTNNB1,RET,HLA-DRB1,STXBP1,ASXL1,ROR2,SKI,ADAMTS20,CDON,BMP7,DYNC2H1,HLA-B,ESR1,COLEC11,BRCA1,PIK3R1,KMT2A,ACTG1 |
| **GO:0060322** | head development | 19 | 692 | 2.28e-08 | MMP2,TGFB1,TTC21B,EGFR,NOTCH1,ATM,TSC1,PTCH1,CTNNB1,AHI1,SKI,BRCA2,CDON,BMP7,DYNC2H1,CTNND1,PAX6,CEP290,MYH3 |
| **GO:0071310** | cellular response to organic substance | 33 | 2219 | 2.51e-08 | TNFRSF1A,MMP2,TGFB1,APC,EDAR,POMT2,EP300,EGFR,NOTCH1,TDGF1,COL1A2,WDR35,FBN1,PTCH1,FGFR3,CTNNB1,RET,RYR1,HLA-DRB1,ACTA1,POMT1,STXBP1,ROR2,FOXH1,SKI,BMP7,HLA-B,PAX6,ESR1,BRCA1,FLNB,PIK3R1,ACTG1 |
| **GO:0048468** | cell development | 27 | 1493 | 2.55e-08 | MYH9,TGFB1,JAG1,EP300,WDPCP,EGFR,NOTCH1,ATM,PTCH1,CTNNB1,RET,RYR1,ACTA1,AHI1,STXBP1,ROR2,SKI,BRCA2,ACTN1,BMP7,PAX6,ESR1,FLNB,PIK3R1,CEP290,ACTG1,MYH3 |
| **GO:0031328** | positive regulation of cellular biosynthetic process | 30 | 1846 | 2.58e-08 | TNFRSF1A,TGFB1,JAG1,POMT2,SMARCB1,EP300,EGFR,NOTCH1,ATM,PTCH1,CTNNB1,RET,HLA-DRB1,AHI1,POMT1,ASXL1,ROR2,FOXH1,SKI,BRCA2,CDON,ACTN1,BMP7,PAX1,PAX6,ESR1,BRCA1,PIK3R1,KMT2A,CEP290 |
| **GO:0048732** | gland development | 15 | 395 | 2.62e-08 | DHODH,TGFB1,EDAR,EGFR,NOTCH1,ATM,TDGF1,WDR35,PTCH1,CTNNB1,ASXL1,BMP7,PAX1,PAX6,ESR1 |
| **GO:0048869** | cellular developmental process | 42 | 3533 | 3.44e-08 | KRT14,MYH9,MMP2,TGFB1,JAG1,EDAR,SMARCB1,EP300,WDPCP,EGFR,NOTCH1,ATM,TDGF1,TSC1,PTCH1,FGFR3,CTNNB1,RET,RYR1,SMOC1,ACTA1,AHI1,PTCH2,STXBP1,ASXL1,ROR2,FOXH1,KDM6A,SKI,BRCA2,CDON,ACTN1,BMP7,DYNC2H1,PAX1,PAX6,ESR1,FLNB,PIK3R1,CEP290,ACTG1,MYH3 |
| **GO:1903508** | positive regulation of nucleic acid-templated transcription | 27 | 1520 | 3.59e-08 | TNFRSF1A,TGFB1,JAG1,SMARCB1,EP300,EGFR,NOTCH1,PTCH1,CTNNB1,RET,HLA-DRB1,AHI1,ASXL1,ROR2,FOXH1,SKI,BRCA2,CDON,ACTN1,BMP7,PAX1,PAX6,ESR1,BRCA1,PIK3R1,KMT2A,CEP290 |
| **GO:0048562** | embryonic organ morphogenesis | 13 | 279 | 3.60e-08 | WDPCP,NOTCH1,FUZ,FBN1,CTNNB1,AHI1,ROR2,FOXH1,KDM6A,BMP7,WDR60,PAX6,CEP290 |
| **GO:0007154** | cell communication | 52 | 5219 | 3.90e-08 | TNFRSF1A,MYH9,MMP2,TGFB1,TTC21B,JAG1,APC,EDAR,EP300,ARHGAP31,ANK1,WDPCP,EGFR,NOTCH1,ATM,IFT122,COL1A2,TSC1,TCTN2,FBN1,FRAS1,PTCH1,FGFR3,CTNNB1,RET,RYR1,HLA-DRB1,AHI1,ABCA4,PTCH2,STXBP1,FGD1,ROR2,FOXH1,KDM6A,SKI,BRCA2,CHRNG,CDON,BMP7,TBC1D32,CTNND1,FGD4,HLA-B,CC2D2A,PAX6,ESR1,BRCA1,FLNB,PIK3R1,ACTG1,TMCO1 |
| **GO:0014020** | primary neural tube formation | 9 | 93 | 4.11e-08 | TGFB1,IFT122,TSC1,FUZ,PTCH1,KDM6A,SKI,BMP7,CC2D2A |
| **GO:0045893** | positive regulation of transcription, DNA-templated | 26 | 1435 | 5.02e-08 | TNFRSF1A,TGFB1,JAG1,SMARCB1,EP300,EGFR,NOTCH1,PTCH1,CTNNB1,RET,HLA-DRB1,AHI1,ASXL1,ROR2,FOXH1,SKI,BRCA2,CDON,BMP7,PAX1,PAX6,ESR1,BRCA1,PIK3R1,KMT2A,CEP290 |
| **GO:0007165** | signal transduction | 49 | 4738 | 5.25e-08 | TNFRSF1A,MYH9,MMP2,TGFB1,TTC21B,JAG1,APC,EDAR,EP300,ARHGAP31,ANK1,WDPCP,EGFR,NOTCH1,ATM,IFT122,COL1A2,TCTN2,FBN1,PTCH1,FGFR3,CTNNB1,RET,RYR1,HLA-DRB1,AHI1,ABCA4,PTCH2,FGD1,ROR2,FOXH1,KDM6A,SKI,BRCA2,CHRNG,CDON,BMP7,TBC1D32,CTNND1,FGD4,HLA-B,CC2D2A,PAX6,ESR1,BRCA1,FLNB,PIK3R1,ACTG1,TMCO1 |
| **GO:0050896** | response to stimulus | 65 | 7824 | 5.25e-08 | TNFRSF1A,TCN2,MYH9,MMP2,DHODH,TGFB1,TTC21B,JAG1,APC,EDAR,POMT2,SMARCB1,EP300,ARHGAP31,ANK1,WDPCP,EGFR,NOTCH1,ATM,CD96,TDGF1,IFT122,COL1A2,TSC1,TCTN2,WDR35,FBN1,PTCH1,FGFR3,CTNNB1,LARGE,RET,RYR1,HLA-DRB1,ACTA1,AHI1,ABCA4,PTCH2,POMT1,STXBP1,FGD1,ASXL1,ROR2,FOXH1,KDM6A,SKI,BRCA2,CHRNG,CDON,BMP7,TBC1D32,CTNND1,FGD4,HLA-B,CC2D2A,PAX6,ESR1,COLEC11,BRCA1,FLNB,PIK3R1,KMT2A,CEP290,ACTG1,TMCO1 |
| **GO:0051716** | cellular response to stimulus | 57 | 6212 | 5.79e-08 | TNFRSF1A,MYH9,MMP2,TGFB1,TTC21B,JAG1,APC,EDAR,POMT2,SMARCB1,EP300,ARHGAP31,ANK1,WDPCP,EGFR,NOTCH1,ATM,TDGF1,IFT122,COL1A2,TSC1,TCTN2,WDR35,FBN1,PTCH1,FGFR3,CTNNB1,RET,RYR1,HLA-DRB1,ACTA1,AHI1,ABCA4,PTCH2,POMT1,STXBP1,FGD1,ROR2,FOXH1,KDM6A,SKI,BRCA2,CHRNG,CDON,BMP7,TBC1D32,CTNND1,FGD4,HLA-B,CC2D2A,PAX6,ESR1,BRCA1,FLNB,PIK3R1,ACTG1,TMCO1 |
| **GO:0030154** | cell differentiation | 41 | 3457 | 6.05e-08 | KRT14,MYH9,MMP2,TGFB1,JAG1,EDAR,SMARCB1,EP300,WDPCP,EGFR,NOTCH1,ATM,TDGF1,TSC1,PTCH1,FGFR3,CTNNB1,RET,RYR1,SMOC1,ACTA1,AHI1,PTCH2,STXBP1,ROR2,FOXH1,KDM6A,SKI,BRCA2,CDON,ACTN1,BMP7,DYNC2H1,PAX1,PAX6,ESR1,FLNB,PIK3R1,CEP290,ACTG1,MYH3 |
| **GO:0008285** | negative regulation of cell population proliferation | 18 | 669 | 7.66e-08 | TGFB1,FKTN,APC,SMARCB1,NOTCH1,ATM,IFT122,TSC1,FUZ,PTCH1,CTNNB1,HLA-DRB1,ROR2,SKI,BRCA2,BMP7,PAX6,KMT2A |
| **GO:0042127** | regulation of cell population proliferation | 27 | 1594 | 8.67e-08 | TNFRSF1A,MMP2,TGFB1,FKTN,JAG1,APC,SMARCB1,EGFR,NOTCH1,ATM,TDGF1,IFT122,TSC1,FUZ,PTCH1,FGFR3,CTNNB1,HLA-DRB1,ROR2,SKI,BRCA2,CDON,BMP7,PAX6,ESR1,BRCA1,KMT2A |
| **GO:0048585** | negative regulation of response to stimulus | 26 | 1483 | 9.06e-08 | TNFRSF1A,TGFB1,FKTN,APC,EGFR,NOTCH1,ATM,CD96,IFT122,TSC1,FUZ,FBN1,PTCH1,CTNNB1,HLA-DRB1,PTCH2,ASXL1,ROR2,FOXH1,SKI,RPGRIP1L,BMP7,CTNND1,HLA-B,ESR1,BRCA1 |
| **GO:0048592** | eye morphogenesis | 10 | 145 | 9.06e-08 | JAG1,IFT122,FBN1,CTNNB1,AHI1,SKI,CDON,BMP7,PAX6,CEP290 |
| **GO:0090596** | sensory organ morphogenesis | 12 | 248 | 9.62e-08 | JAG1,WDPCP,IFT122,FBN1,CTNNB1,AHI1,ROR2,SKI,CDON,BMP7,PAX6,CEP290 |
| **GO:0010171** | body morphogenesis | 7 | 44 | 1.21e-07 | MMP2,TGFB1,IFT122,FUZ,SKI,CDON,MYH3 |
| **GO:0051173** | positive regulation of nitrogen compound metabolic process | 37 | 2946 | 1.21e-07 | TNFRSF1A,MYH9,TGFB1,JAG1,APC,EDAR,POMT2,SMARCB1,EP300,EGFR,NOTCH1,ATM,TDGF1,WDR35,FBN1,PTCH1,FGFR3,CTNNB1,RET,HLA-DRB1,AHI1,POMT1,ASXL1,ROR2,FOXH1,SKI,BRCA2,CDON,ACTN1,BMP7,PAX1,PAX6,ESR1,BRCA1,PIK3R1,KMT2A,CEP290 |
| **GO:0031399** | regulation of protein modification process | 28 | 1747 | 1.25e-07 | TNFRSF1A,TGFB1,FKTN,APC,EDAR,POMT2,SMARCB1,EP300,EGFR,NOTCH1,ATM,TDGF1,TSC1,FBN1,RPS7,FGFR3,CTNNB1,RET,HLA-DRB1,POMT1,RPL11,ROR2,SKI,CDON,BMP7,PAX6,BRCA1,KMT2A |
| **GO:0001501** | skeletal system development | 15 | 457 | 1.40e-07 | MMP2,TGFB1,EP300,COL1A2,FUZ,FBN1,FGFR3,CTNNB1,RYR1,ASXL1,ROR2,SKI,BMP7,PAX1,WDR60 |
| **GO:0048522** | positive regulation of cellular process | 49 | 4898 | 1.48e-07 | TNFRSF1A,MYH9,MMP2,DHODH,TGFB1,JAG1,APC,EDAR,POMT2,SMARCB1,EP300,ANK1,EGFR,NOTCH1,ATM,TDGF1,TSC1,FUZ,WDR35,FBN1,PTCH1,RPS7,FGFR3,CTNNB1,RET,HLA-DRB1,AHI1,PTCH2,POMT1,STXBP1,FGD1,ASXL1,ROR2,FOXH1,SKI,BRCA2,ADAMTS20,CDON,ACTN1,BMP7,DYNC2H1,PAX1,FGD4,PAX6,ESR1,BRCA1,PIK3R1,KMT2A,CEP290 |
| **GO:0045935** | positive regulation of nucleobase-containing compound metabolic process | 28 | 1770 | 1.62e-07 | TNFRSF1A,TGFB1,JAG1,SMARCB1,EP300,EGFR,NOTCH1,ATM,PTCH1,CTNNB1,RET,HLA-DRB1,AHI1,ASXL1,ROR2,FOXH1,SKI,BRCA2,CDON,ACTN1,BMP7,PAX1,PAX6,ESR1,BRCA1,PIK3R1,KMT2A,CEP290 |
| **GO:0023052** | signaling | 50 | 5108 | 1.85e-07 | TNFRSF1A,MYH9,MMP2,TGFB1,TTC21B,JAG1,APC,EDAR,EP300,ARHGAP31,ANK1,WDPCP,EGFR,NOTCH1,ATM,IFT122,COL1A2,TCTN2,FBN1,PTCH1,FGFR3,CTNNB1,RET,RYR1,HLA-DRB1,AHI1,ABCA4,PTCH2,STXBP1,FGD1,ROR2,FOXH1,KDM6A,SKI,BRCA2,CHRNG,CDON,BMP7,TBC1D32,CTNND1,FGD4,HLA-B,CC2D2A,PAX6,ESR1,BRCA1,FLNB,PIK3R1,ACTG1,TMCO1 |
| **GO:0032989** | cellular component morphogenesis | 18 | 720 | 2.07e-07 | MYH9,EP300,WDPCP,EGFR,NOTCH1,PTCH1,CTNNB1,RET,ACTA1,STXBP1,ASXL1,ACTN1,BMP7,PAX6,FLNB,PIK3R1,ACTG1,MYH3 |
| **GO:0007399** | nervous system development | 31 | 2206 | 2.90e-07 | TGFB1,FKTN,TTC21B,JAG1,SMARCB1,EP300,WDPCP,EGFR,NOTCH1,ATM,IFT122,TSC1,FUZ,PTCH1,CTNNB1,RET,AHI1,STXBP1,ROR2,KDM6A,SKI,BRCA2,CDON,BMP7,DYNC2H1,TBC1D32,CTNND1,CC2D2A,PAX6,PIK3R1,CEP290 |
| **GO:0031325** | positive regulation of cellular metabolic process | 37 | 3060 | 3.17e-07 | TNFRSF1A,MYH9,TGFB1,JAG1,EDAR,POMT2,SMARCB1,EP300,EGFR,NOTCH1,ATM,TDGF1,TSC1,WDR35,FBN1,PTCH1,FGFR3,CTNNB1,RET,HLA-DRB1,AHI1,POMT1,ASXL1,ROR2,FOXH1,SKI,BRCA2,CDON,ACTN1,BMP7,PAX1,PAX6,ESR1,BRCA1,PIK3R1,KMT2A,CEP290 |
| **GO:0006928** | movement of cell or subcellular component | 24 | 1355 | 3.20e-07 | MYH9,TGFB1,TTC21B,APC,WDPCP,EGFR,NOTCH1,TDGF1,IFT122,COL1A2,WDR35,PTCH1,CTNNB1,RET,ACTA1,NUP188,ROR2,SKI,BMP7,DYNC2H1,WDR60,PAX6,PIK3R1,MYH3 |
| **GO:0010648** | negative regulation of cell communication | 23 | 1255 | 3.73e-07 | TGFB1,FKTN,APC,EGFR,NOTCH1,ATM,IFT122,TSC1,FUZ,FBN1,PTCH1,CTNNB1,PTCH2,STXBP1,ASXL1,ROR2,FOXH1,SKI,RPGRIP1L,BMP7,CTNND1,ESR1,BRCA1 |
| **GO:0001843** | neural tube closure | 8 | 86 | 3.81e-07 | TGFB1,IFT122,TSC1,FUZ,PTCH1,KDM6A,SKI,CC2D2A |
| **GO:0023057** | negative regulation of signaling | 23 | 1258 | 3.83e-07 | TGFB1,FKTN,APC,EGFR,NOTCH1,ATM,IFT122,TSC1,FUZ,FBN1,PTCH1,CTNNB1,PTCH2,STXBP1,ASXL1,ROR2,FOXH1,SKI,RPGRIP1L,BMP7,CTNND1,ESR1,BRCA1 |
| **GO:0007517** | muscle organ development | 12 | 287 | 4.02e-07 | TGFB1,FKTN,EP300,NOTCH1,LARGE,RYR1,ACTA1,FOXH1,SKI,CDON,FLNB,MYH3 |
| **GO:0009967** | positive regulation of signal transduction | 25 | 1493 | 4.17e-07 | TNFRSF1A,TGFB1,JAG1,EDAR,SMARCB1,EP300,EGFR,NOTCH1,ATM,TDGF1,WDR35,RPS7,FGFR3,CTNNB1,RET,HLA-DRB1,ASXL1,ROR2,SKI,ADAMTS20,CDON,BMP7,DYNC2H1,ESR1,PIK3R1 |
| **GO:0009968** | negative regulation of signal transduction | 22 | 1160 | 4.34e-07 | TGFB1,FKTN,APC,EGFR,NOTCH1,ATM,IFT122,TSC1,FUZ,FBN1,PTCH1,CTNNB1,PTCH2,ASXL1,ROR2,FOXH1,SKI,RPGRIP1L,BMP7,CTNND1,ESR1,BRCA1 |
| **GO:0070887** | cellular response to chemical stimulus | 34 | 2672 | 4.55e-07 | TNFRSF1A,MMP2,TGFB1,APC,EDAR,POMT2,EP300,EGFR,NOTCH1,TDGF1,COL1A2,TSC1,WDR35,FBN1,PTCH1,FGFR3,CTNNB1,RET,RYR1,HLA-DRB1,ACTA1,POMT1,STXBP1,ROR2,FOXH1,SKI,BMP7,HLA-B,PAX6,ESR1,BRCA1,FLNB,PIK3R1,ACTG1 |
| **GO:0009798** | axis specification | 8 | 89 | 4.61e-07 | NOTCH1,TDGF1,PTCH1,CTNNB1,AHI1,KDM6A,SKI,PAX6 |
| **GO:0007417** | central nervous system development | 19 | 861 | 4.76e-07 | TGFB1,TTC21B,EGFR,NOTCH1,ATM,IFT122,TSC1,PTCH1,CTNNB1,AHI1,ROR2,SKI,BRCA2,CDON,BMP7,DYNC2H1,CTNND1,PAX6,CEP290 |
| **GO:2000026** | regulation of multicellular organismal development | 28 | 1876 | 4.98e-07 | TNFRSF1A,TGFB1,JAG1,EP300,WDPCP,EGFR,NOTCH1,IFT122,TSC1,FUZ,FBN1,PTCH1,CTNNB1,RET,HLA-DRB1,AHI1,PTCH2,ROR2,SKI,BRCA2,CDON,BMP7,HLA-B,PAX6,ESR1,BRCA1,PIK3R1,KMT2A |
| **GO:0010647** | positive regulation of cell communication | 26 | 1631 | 5.08e-07 | TNFRSF1A,TGFB1,JAG1,EDAR,SMARCB1,EP300,EGFR,NOTCH1,ATM,TDGF1,WDR35,RPS7,FGFR3,CTNNB1,RET,HLA-DRB1,STXBP1,ASXL1,ROR2,SKI,ADAMTS20,CDON,BMP7,DYNC2H1,ESR1,PIK3R1 |
| **GO:0023056** | positive regulation of signaling | 26 | 1638 | 5.49e-07 | TNFRSF1A,TGFB1,JAG1,EDAR,SMARCB1,EP300,EGFR,NOTCH1,ATM,TDGF1,WDR35,RPS7,FGFR3,CTNNB1,RET,HLA-DRB1,STXBP1,ASXL1,ROR2,SKI,ADAMTS20,CDON,BMP7,DYNC2H1,ESR1,PIK3R1 |
| **GO:0009987** | cellular process | 88 | 14652 | 5.54e-07 | TNFRSF1A,KRT14,TCN2,MYH9,MMP2,DHODH,TGFB1,FKTN,TTC21B,JAG1,DMGDH,APC,EDAR,POMT2,PHYH,SMARCB1,EP300,ARHGAP31,ANK1,WDPCP,EGFR,NOTCH1,ATM,TDGF1,IFT122,COL1A2,TSC1,TCTN2,FUZ,WDR35,FBN1,FRAS1,PTCH1,RPS7,FGFR3,CTNNB1,LARGE,RET,RPS26,RYR1,HLA-DRB1,SMOC1,ACTA1,AHI1,RPS27,ABCA4,INPP5E,PTCH2,POMT1,NUP188,STXBP1,RPL11,FGD1,ASXL1,ROR2,FOXH1,KDM6A,SKI,PIGO,RPGRIP1L,BRCA2,ADAMTS20,CHRNG,CDON,ACTN1,BMP7,DYNC2H1,TBC1D32,PAX1,CTNND1,WDR60,TMEM237,FGD4,HLA-B,CC2D2A,PAX6,ESR1,COLEC11,PIGG,BRCA1,FLNB,ADH1C,PIK3R1,KMT2A,CEP290,ACTG1,MYH3,TMCO1 |
| **GO:0022612** | gland morphogenesis | 8 | 94 | 6.54e-07 | TGFB1,EDAR,EGFR,NOTCH1,PTCH1,BMP7,PAX6,ESR1 |
| **GO:0043588** | skin development | 13 | 373 | 6.95e-07 | KRT14,JAG1,EDAR,EGFR,NOTCH1,COL1A2,FUZ,FRAS1,CTNNB1,RYR1,PTCH2,PAX6,FLNB |
| **GO:0045595** | regulation of cell differentiation | 26 | 1695 | 1.06e-06 | TNFRSF1A,TGFB1,JAG1,EP300,EGFR,NOTCH1,TSC1,FUZ,FBN1,PTCH1,CTNNB1,RET,HLA-DRB1,SMOC1,AHI1,PTCH2,ASXL1,ROR2,SKI,ADAMTS20,CDON,BMP7,HLA-B,PAX6,PIK3R1,KMT2A |
| **GO:0045184** | establishment of protein localization | 24 | 1467 | 1.24e-06 | MYH9,TGFB1,TTC21B,PHYH,WDPCP,EGFR,NOTCH1,ATM,IFT122,TSC1,FUZ,WDR35,FRAS1,RPS7,RPS26,RPS27,NUP188,STXBP1,RPL11,BRCA2,DYNC2H1,WDR60,PIK3R1,CEP290 |
| **GO:0061512** | protein localization to cilium | 6 | 38 | 1.37e-06 | TTC21B,IFT122,WDR35,DYNC2H1,TBC1D32,CC2D2A |
| **GO:0048705** | skeletal system morphogenesis | 10 | 204 | 1.50e-06 | MMP2,TGFB1,FUZ,FGFR3,CTNNB1,ROR2,SKI,BMP7,PAX1,WDR60 |
| **GO:0050789** | regulation of biological process | 76 | 11116 | 1.52e-06 | TNFRSF1A,MYH9,MMP2,DHODH,TGFB1,FKTN,TTC21B,JAG1,APC,EDAR,POMT2,SMARCB1,EP300,ARHGAP31,ANK1,WDPCP,EGFR,NOTCH1,ATM,CD96,TDGF1,IFT122,COL1A2,TSC1,TCTN2,FUZ,WDR35,FBN1,PTCH1,RPS7,FGFR3,CTNNB1,RET,RPS26,RYR1,HLA-DRB1,SMOC1,ACTA1,AHI1,RPS27,ABCA4,PTCH2,POMT1,STXBP1,RPL11,FGD1,ASXL1,ROR2,FOXH1,KDM6A,SKI,RPGRIP1L,BRCA2,ADAMTS20,CHRNG,CDON,ACTN1,BMP7,DYNC2H1,TBC1D32,PAX1,CTNND1,TMEM237,FGD4,HLA-B,CC2D2A,PAX6,ESR1,COLEC11,BRCA1,FLNB,PIK3R1,KMT2A,CEP290,ACTG1,TMCO1 |
| **GO:0007420** | brain development | 16 | 650 | 1.62e-06 | TTC21B,EGFR,NOTCH1,ATM,TSC1,PTCH1,CTNNB1,AHI1,SKI,BRCA2,CDON,BMP7,DYNC2H1,CTNND1,PAX6,CEP290 |
| **GO:0048593** | camera-type eye morphogenesis | 8 | 109 | 1.81e-06 | JAG1,IFT122,CTNNB1,AHI1,SKI,CDON,BMP7,PAX6 |
| **GO:0014706** | striated muscle tissue development | 11 | 271 | 1.93e-06 | TGFB1,EP300,NOTCH1,TSC1,RYR1,ACTA1,FOXH1,SKI,CDON,BMP7,FLNB |
| **GO:0065007** | biological regulation | 78 | 11740 | 2.29e-06 | TNFRSF1A,MYH9,MMP2,DHODH,TGFB1,FKTN,TTC21B,JAG1,DMGDH,APC,EDAR,POMT2,SMARCB1,EP300,ARHGAP31,ANK1,WDPCP,EGFR,NOTCH1,ATM,CD96,TDGF1,IFT122,COL1A2,TSC1,TCTN2,FUZ,WDR35,FBN1,PTCH1,RPS7,FGFR3,CTNNB1,LARGE,RET,RPS26,RYR1,HLA-DRB1,SMOC1,ACTA1,AHI1,RPS27,ABCA4,PTCH2,POMT1,STXBP1,RPL11,FGD1,ASXL1,ROR2,FOXH1,KDM6A,SKI,RPGRIP1L,BRCA2,ADAMTS20,CHRNG,CDON,ACTN1,BMP7,DYNC2H1,TBC1D32,PAX1,CTNND1,TMEM237,FGD4,HLA-B,CC2D2A,PAX6,ESR1,COLEC11,BRCA1,FLNB,PIK3R1,KMT2A,CEP290,ACTG1,TMCO1 |
| **GO:0010243** | response to organonitrogen compound | 18 | 876 | 3.03e-06 | MMP2,DHODH,TGFB1,JAG1,APC,POMT2,EGFR,NOTCH1,COL1A2,TSC1,FBN1,CTNNB1,RYR1,ACTA1,POMT1,BMP7,BRCA1,PIK3R1 |
| **GO:0045944** | positive regulation of transcription by RNA polymerase II | 20 | 1104 | 3.97e-06 | TNFRSF1A,TGFB1,JAG1,SMARCB1,EP300,EGFR,NOTCH1,CTNNB1,AHI1,ASXL1,FOXH1,SKI,CDON,BMP7,PAX1,PAX6,ESR1,BRCA1,PIK3R1,KMT2A |
| **GO:0042692** | muscle cell differentiation | 10 | 230 | 4.11e-06 | MYH9,NOTCH1,TSC1,CTNNB1,RYR1,ACTA1,SKI,CDON,ACTG1,MYH3 |
| **GO:0003007** | heart morphogenesis | 10 | 235 | 4.94e-06 | TGFB1,JAG1,NOTCH1,PTCH1,RYR1,AHI1,ASXL1,FOXH1,KDM6A,BMP7 |
| **GO:0009719** | response to endogenous stimulus | 22 | 1353 | 5.23e-06 | MMP2,TGFB1,APC,EGFR,NOTCH1,TDGF1,COL1A2,TSC1,FBN1,PTCH1,FGFR3,CTNNB1,RYR1,ACTA1,STXBP1,ROR2,FOXH1,SKI,BMP7,ESR1,BRCA1,PIK3R1 |
| **GO:0072073** | kidney epithelium development | 8 | 128 | 5.46e-06 | TGFB1,JAG1,NOTCH1,PTCH1,CTNNB1,RET,AHI1,BMP7 |
| **GO:0048519** | negative regulation of biological process | 46 | 4953 | 5.57e-06 | TNFRSF1A,MYH9,TGFB1,FKTN,JAG1,APC,SMARCB1,EP300,EGFR,NOTCH1,ATM,CD96,TDGF1,IFT122,TSC1,FUZ,WDR35,FBN1,PTCH1,RPS7,FGFR3,CTNNB1,RPS26,RYR1,HLA-DRB1,AHI1,RPS27,PTCH2,STXBP1,RPL11,ASXL1,ROR2,FOXH1,SKI,RPGRIP1L,BRCA2,ADAMTS20,ACTN1,BMP7,CTNND1,HLA-B,PAX6,ESR1,BRCA1,PIK3R1,KMT2A |
| **GO:0000902** | cell morphogenesis | 15 | 626 | 5.66e-06 | MYH9,EP300,WDPCP,EGFR,NOTCH1,PTCH1,CTNNB1,RET,STXBP1,ASXL1,ACTN1,BMP7,PAX6,FLNB,PIK3R1 |
| **GO:0060538** | skeletal muscle organ development | 8 | 130 | 5.99e-06 | EP300,NOTCH1,LARGE,RYR1,ACTA1,SKI,CDON,FLNB |
| **GO:0009953** | dorsal/ventral pattern formation | 7 | 87 | 6.32e-06 | TTC21B,IFT122,PTCH1,CTNNB1,DYNC2H1,TBC1D32,PAX6 |
| **GO:0031401** | positive regulation of protein modification process | 20 | 1149 | 7.01e-06 | TNFRSF1A,TGFB1,EDAR,POMT2,SMARCB1,EGFR,NOTCH1,ATM,TDGF1,FBN1,FGFR3,CTNNB1,RET,HLA-DRB1,POMT1,ROR2,CDON,BMP7,BRCA1,KMT2A |
| **GO:0042633** | hair cycle | 7 | 89 | 7.21e-06 | KRT14,EDAR,EGFR,NOTCH1,FUZ,CTNNB1,PTCH2 |
| **GO:0060485** | mesenchyme development | 9 | 187 | 7.26e-06 | TGFB1,JAG1,NOTCH1,CTNNB1,RET,ACTA1,FOXH1,BMP7,PAX1 |
| **GO:0042221** | response to chemical | 41 | 4153 | 7.98e-06 | TNFRSF1A,TCN2,MMP2,DHODH,TGFB1,JAG1,APC,EDAR,POMT2,EP300,EGFR,NOTCH1,CD96,TDGF1,COL1A2,TSC1,WDR35,FBN1,PTCH1,FGFR3,CTNNB1,RET,RYR1,HLA-DRB1,ACTA1,POMT1,STXBP1,ASXL1,ROR2,FOXH1,SKI,CHRNG,BMP7,HLA-B,PAX6,ESR1,BRCA1,FLNB,PIK3R1,KMT2A,ACTG1 |
| **GO:0030856** | regulation of epithelial cell differentiation | 8 | 137 | 8.36e-06 | TNFRSF1A,JAG1,NOTCH1,PTCH1,CTNNB1,AHI1,PTCH2,PAX6 |
| **GO:0050794** | regulation of cellular process | 72 | 10484 | 8.84e-06 | TNFRSF1A,MYH9,MMP2,DHODH,TGFB1,FKTN,TTC21B,JAG1,APC,EDAR,POMT2,SMARCB1,EP300,ARHGAP31,ANK1,WDPCP,EGFR,NOTCH1,ATM,TDGF1,IFT122,COL1A2,TSC1,TCTN2,FUZ,WDR35,FBN1,PTCH1,RPS7,FGFR3,CTNNB1,RET,RPS26,RYR1,HLA-DRB1,SMOC1,AHI1,ABCA4,PTCH2,POMT1,STXBP1,RPL11,FGD1,ASXL1,ROR2,FOXH1,KDM6A,SKI,RPGRIP1L,BRCA2,ADAMTS20,CHRNG,CDON,ACTN1,BMP7,DYNC2H1,TBC1D32,PAX1,CTNND1,TMEM237,FGD4,HLA-B,CC2D2A,PAX6,ESR1,BRCA1,FLNB,PIK3R1,KMT2A,CEP290,ACTG1,TMCO1 |
| **GO:0007435** | salivary gland morphogenesis | 5 | 28 | 9.19e-06 | TGFB1,EDAR,EGFR,BMP7,PAX6 |
| **GO:0035721** | intraciliary retrograde transport | 4 | 10 | 9.30e-06 | TTC21B,IFT122,WDR35,DYNC2H1 |
| **GO:0008544** | epidermis development | 12 | 403 | 1.04e-05 | KRT14,JAG1,EDAR,WDPCP,EGFR,NOTCH1,FUZ,PTCH1,CTNNB1,PTCH2,PAX6,FLNB |
| **GO:0048534** | hematopoietic or lymphoid organ development | 14 | 573 | 1.09e-05 | MYH9,TGFB1,JAG1,EP300,ATM,TSC1,CTNNB1,RET,ASXL1,BRCA2,ACTN1,PAX1,PIK3R1,KMT2A |
| **GO:0032268** | regulation of cellular protein metabolic process | 30 | 2486 | 1.11e-05 | TNFRSF1A,MYH9,TGFB1,FKTN,APC,EDAR,POMT2,SMARCB1,EP300,EGFR,NOTCH1,ATM,TDGF1,TSC1,WDR35,FBN1,RPS7,FGFR3,CTNNB1,RET,HLA-DRB1,POMT1,RPL11,ROR2,SKI,CDON,BMP7,PAX6,BRCA1,KMT2A |
| **GO:1901700** | response to oxygen-containing compound | 22 | 1427 | 1.14e-05 | TNFRSF1A,MMP2,DHODH,TGFB1,JAG1,APC,EGFR,NOTCH1,CD96,COL1A2,TSC1,WDR35,FBN1,PTCH1,CTNNB1,RET,STXBP1,ASXL1,BMP7,ESR1,BRCA1,PIK3R1 |
| **GO:0051239** | regulation of multicellular organismal process | 32 | 2788 | 1.18e-05 | TNFRSF1A,TGFB1,JAG1,EP300,WDPCP,EGFR,NOTCH1,CD96,TDGF1,IFT122,TSC1,FUZ,FBN1,PTCH1,CTNNB1,RET,RYR1,HLA-DRB1,SMOC1,AHI1,PTCH2,ROR2,SKI,BRCA2,CDON,BMP7,HLA-B,PAX6,ESR1,BRCA1,PIK3R1,KMT2A |
| **GO:0030858** | positive regulation of epithelial cell differentiation | 6 | 59 | 1.19e-05 | NOTCH1,PTCH1,CTNNB1,AHI1,PTCH2,PAX6 |
| **GO:0072358** | cardiovascular system development | 13 | 496 | 1.32e-05 | MYH9,MMP2,TGFB1,JAG1,WDPCP,NOTCH1,TDGF1,COL1A2,CTNNB1,FOXH1,KDM6A,DYNC2H1,PAX6 |
| **GO:0000904** | cell morphogenesis involved in differentiation | 13 | 498 | 1.37e-05 | MYH9,EP300,WDPCP,NOTCH1,PTCH1,CTNNB1,RET,STXBP1,ACTN1,BMP7,PAX6,FLNB,PIK3R1 |
| **GO:0071495** | cellular response to endogenous stimulus | 19 | 1106 | 1.59e-05 | MMP2,TGFB1,APC,EGFR,NOTCH1,TDGF1,COL1A2,FBN1,FGFR3,CTNNB1,RYR1,ACTA1,ROR2,FOXH1,SKI,BMP7,ESR1,BRCA1,PIK3R1 |
| **GO:0051247** | positive regulation of protein metabolic process | 23 | 1587 | 1.64e-05 | TNFRSF1A,MYH9,TGFB1,APC,EDAR,POMT2,SMARCB1,EGFR,NOTCH1,ATM,TDGF1,WDR35,FBN1,FGFR3,CTNNB1,RET,HLA-DRB1,POMT1,ROR2,CDON,BMP7,BRCA1,KMT2A |
| **GO:0048523** | negative regulation of cellular process | 42 | 4454 | 1.68e-05 | TNFRSF1A,MYH9,TGFB1,FKTN,JAG1,APC,SMARCB1,EP300,EGFR,NOTCH1,ATM,TDGF1,IFT122,TSC1,FUZ,WDR35,FBN1,PTCH1,RPS7,CTNNB1,RPS26,RYR1,HLA-DRB1,AHI1,PTCH2,STXBP1,RPL11,ASXL1,ROR2,FOXH1,SKI,RPGRIP1L,BRCA2,ADAMTS20,ACTN1,BMP7,CTNND1,PAX6,ESR1,BRCA1,PIK3R1,KMT2A |
| **GO:0042733** | embryonic digit morphogenesis | 6 | 64 | 1.77e-05 | WDPCP,NOTCH1,IFT122,CTNNB1,ROR2,TBC1D32 |
| **GO:0051093** | negative regulation of developmental process | 17 | 910 | 2.12e-05 | TGFB1,JAG1,EGFR,NOTCH1,IFT122,TSC1,FUZ,FBN1,PTCH1,FGFR3,CTNNB1,ASXL1,SKI,BRCA2,BMP7,PAX6,PIK3R1 |
| **GO:0032270** | positive regulation of cellular protein metabolic process | 22 | 1496 | 2.32e-05 | TNFRSF1A,MYH9,TGFB1,EDAR,POMT2,SMARCB1,EGFR,NOTCH1,ATM,TDGF1,WDR35,FBN1,FGFR3,CTNNB1,RET,HLA-DRB1,POMT1,ROR2,CDON,BMP7,BRCA1,KMT2A |
| **GO:0030097** | hemopoiesis | 13 | 526 | 2.34e-05 | MYH9,TGFB1,JAG1,EP300,ATM,TSC1,CTNNB1,ASXL1,BRCA2,ACTN1,PAX1,PIK3R1,KMT2A |
| **GO:0002088** | lens development in camera-type eye | 6 | 68 | 2.40e-05 | TGFB1,CTNNB1,SKI,CDON,TBC1D32,PAX6 |
| **GO:0042981** | regulation of apoptotic process | 22 | 1501 | 2.41e-05 | TNFRSF1A,DHODH,TGFB1,APC,EGFR,NOTCH1,ATM,TDGF1,WDR35,RPS7,CTNNB1,RET,AHI1,STXBP1,FGD1,ADAMTS20,ACTN1,BMP7,FGD4,ESR1,BRCA1,PIK3R1 |
| **GO:1902531** | regulation of intracellular signal transduction | 24 | 1764 | 2.58e-05 | TNFRSF1A,TGFB1,FKTN,EDAR,EP300,ARHGAP31,EGFR,NOTCH1,ATM,TDGF1,TSC1,RPS7,FGFR3,CTNNB1,RET,HLA-DRB1,RPL11,FGD1,ROR2,CDON,BMP7,FGD4,ESR1,PIK3R1 |
| **GO:0045667** | regulation of osteoblast differentiation | 7 | 112 | 2.61e-05 | JAG1,NOTCH1,PTCH1,CTNNB1,SMOC1,SKI,BMP7 |
| **GO:0010941** | regulation of cell death | 23 | 1638 | 2.65e-05 | TNFRSF1A,DHODH,TGFB1,APC,EGFR,NOTCH1,ATM,TDGF1,TSC1,WDR35,RPS7,CTNNB1,RET,AHI1,STXBP1,FGD1,ADAMTS20,ACTN1,BMP7,FGD4,ESR1,BRCA1,PIK3R1 |
| **GO:0008630** | intrinsic apoptotic signaling pathway in response to DNA damage | 6 | 71 | 2.94e-05 | TNFRSF1A,EP300,ATM,BRCA2,BRCA1,PIK3R1 |
| **GO:0035269** | protein O-linked mannosylation | 4 | 15 | 3.04e-05 | FKTN,POMT2,LARGE,POMT1 |
| **GO:0001708** | cell fate specification | 6 | 72 | 3.13e-05 | NOTCH1,PTCH1,CTNNB1,PTCH2,CDON,PAX6 |
| **GO:0009628** | response to abiotic stimulus | 18 | 1052 | 3.14e-05 | TNFRSF1A,MMP2,TGFB1,EP300,EGFR,NOTCH1,ATM,TSC1,PTCH1,RYR1,ACTA1,ABCA4,BRCA2,BMP7,BRCA1,PIK3R1,KMT2A,ACTG1 |
| **GO:1905515** | non-motile cilium assembly | 5 | 39 | 3.39e-05 | IFT122,FUZ,DYNC2H1,TBC1D32,CC2D2A |
| **GO:0035735** | intraciliary transport involved in cilium assembly | 5 | 40 | 3.78e-05 | TTC21B,IFT122,WDR35,DYNC2H1,WDR60 |
| **GO:0008589** | regulation of smoothened signaling pathway | 6 | 75 | 3.83e-05 | TTC21B,IFT122,FUZ,PTCH1,PTCH2,DYNC2H1 |
| **GO:0007519** | skeletal muscle tissue development | 7 | 122 | 4.23e-05 | EP300,NOTCH1,RYR1,ACTA1,SKI,CDON,FLNB |
| **GO:0071363** | cellular response to growth factor stimulus | 12 | 477 | 4.81e-05 | TGFB1,EGFR,NOTCH1,TDGF1,COL1A2,FBN1,FGFR3,CTNNB1,ROR2,FOXH1,SKI,BMP7 |
| **GO:0030278** | regulation of ossification | 8 | 181 | 4.97e-05 | TGFB1,JAG1,NOTCH1,PTCH1,CTNNB1,SMOC1,SKI,BMP7 |
| **GO:0061053** | somite development | 6 | 79 | 4.97e-05 | EP300,ATM,PTCH1,ROR2,KDM6A,PAX1 |
| **GO:0001709** | cell fate determination | 5 | 43 | 5.06e-05 | JAG1,PTCH1,CTNNB1,PTCH2,PAX6 |
| **GO:0032355** | response to estradiol | 7 | 126 | 5.06e-05 | TGFB1,EGFR,PTCH1,CTNNB1,STXBP1,BMP7,ESR1 |
| **GO:0051146** | striated muscle cell differentiation | 8 | 182 | 5.06e-05 | MYH9,TSC1,RYR1,ACTA1,SKI,CDON,ACTG1,MYH3 |
| **GO:0072594** | establishment of protein localization to organelle | 11 | 396 | 5.06e-05 | TGFB1,PHYH,ATM,IFT122,RPS7,RPS26,RPS27,NUP188,RPL11,BRCA2,PIK3R1 |
| **GO:0051241** | negative regulation of multicellular organismal process | 18 | 1098 | 5.32e-05 | TNFRSF1A,TGFB1,JAG1,EGFR,NOTCH1,CD96,IFT122,TSC1,FUZ,FBN1,PTCH1,CTNNB1,HLA-DRB1,SKI,BRCA2,BMP7,PAX6,PIK3R1 |
| **GO:0001656** | metanephros development | 6 | 83 | 6.27e-05 | FBN1,FRAS1,PTCH1,CTNNB1,RET,BMP7 |
| **GO:0045596** | negative regulation of cell differentiation | 14 | 683 | 6.54e-05 | TGFB1,JAG1,EGFR,NOTCH1,TSC1,FUZ,FBN1,PTCH1,CTNNB1,ASXL1,SKI,BMP7,PAX6,PIK3R1 |
| **GO:1903706** | regulation of hemopoiesis | 11 | 412 | 7.09e-05 | TGFB1,JAG1,EP300,NOTCH1,FBN1,CTNNB1,HLA-DRB1,ROR2,HLA-B,PIK3R1,KMT2A |
| **GO:0031056** | regulation of histone modification | 7 | 134 | 7.16e-05 | TGFB1,SMARCB1,ATM,CTNNB1,SKI,BRCA1,KMT2A |
| **GO:0051128** | regulation of cellular component organization | 27 | 2306 | 7.16e-05 | TNFRSF1A,MYH9,DHODH,TGFB1,APC,SMARCB1,EP300,ANK1,WDPCP,EGFR,NOTCH1,ATM,TSC1,FUZ,WDR35,CTNNB1,RET,AHI1,STXBP1,FGD1,SKI,BMP7,FGD4,ESR1,BRCA1,PIK3R1,KMT2A |
| **GO:0031058** | positive regulation of histone modification | 6 | 86 | 7.39e-05 | TGFB1,SMARCB1,ATM,CTNNB1,BRCA1,KMT2A |
| **GO:0007167** | enzyme linked receptor protein signaling pathway | 14 | 698 | 8.11e-05 | MMP2,TGFB1,APC,EGFR,COL1A2,FGFR3,RET,AHI1,ROR2,FOXH1,SKI,BMP7,PIK3R1,ACTG1 |
| **GO:0065008** | regulation of biological quality | 35 | 3559 | 8.14e-05 | MYH9,DHODH,TGFB1,DMGDH,SMARCB1,EP300,WDPCP,EGFR,NOTCH1,ATM,COL1A2,TSC1,WDR35,FBN1,PTCH1,RPS7,CTNNB1,LARGE,RET,RYR1,HLA-DRB1,ABCA4,STXBP1,RPL11,FGD1,ASXL1,BRCA2,CHRNG,FGD4,PAX6,ESR1,FLNB,PIK3R1,KMT2A,TMCO1 |
| **GO:0043065** | positive regulation of apoptotic process | 13 | 604 | 8.58e-05 | TNFRSF1A,DHODH,TGFB1,APC,NOTCH1,ATM,WDR35,RPS7,CTNNB1,RET,FGD1,BMP7,FGD4 |
| **GO:0055123** | digestive system development | 7 | 139 | 8.63e-05 | TGFB1,WDPCP,EGFR,NOTCH1,CTNNB1,RET,AHI1 |
| **GO:0051234** | establishment of localization | 39 | 4248 | 8.94e-05 | TCN2,MYH9,DHODH,TGFB1,TTC21B,PHYH,ANK1,WDPCP,EGFR,NOTCH1,ATM,IFT122,TSC1,FUZ,WDR35,FRAS1,RPS7,CTNNB1,RPS26,SLC2A10,RYR1,AHI1,RPS27,ABCA4,NUP188,STXBP1,RPL11,BRCA2,CHRNG,ACTN1,DYNC2H1,WDR60,HLA-B,PAX6,COLEC11,PIK3R1,CEP290,ACTG1,TMCO1 |
| **GO:0015031** | protein transport | 20 | 1391 | 9.02e-05 | MYH9,TGFB1,TTC21B,PHYH,NOTCH1,IFT122,TSC1,FUZ,WDR35,FRAS1,RPS7,RPS26,RPS27,NUP188,STXBP1,RPL11,DYNC2H1,WDR60,PIK3R1,CEP290 |
| **GO:0061311** | cell surface receptor signaling pathway involved in heart development | 4 | 22 | 9.71e-05 | TGFB1,JAG1,NOTCH1,CTNNB1 |
| **GO:0002376** | immune system process | 27 | 2370 | 0.00011 | TNFRSF1A,MYH9,TGFB1,JAG1,EP300,NOTCH1,ATM,CD96,TDGF1,COL1A2,TSC1,CTNNB1,RET,HLA-DRB1,STXBP1,ASXL1,ROR2,BRCA2,ACTN1,PAX1,HLA-B,COLEC11,FLNB,PIK3R1,KMT2A,CEP290,ACTG1 |
| **GO:0014902** | myotube differentiation | 5 | 52 | 0.00011 | MYH9,RYR1,ACTA1,SKI,CDON |
| **GO:0033993** | response to lipid | 15 | 825 | 0.00011 | TNFRSF1A,TGFB1,EGFR,NOTCH1,CD96,WDR35,PTCH1,CTNNB1,RET,ACTA1,STXBP1,ASXL1,BMP7,ESR1,BRCA1 |
| **GO:0035282** | segmentation | 6 | 93 | 0.00011 | EP300,ATM,TDGF1,ROR2,KDM6A,PAX1 |
| **GO:0010172** | embryonic body morphogenesis | 3 | 6 | 0.00012 | IFT122,FUZ,CDON |
| **GO:0051961** | negative regulation of nervous system development | 9 | 279 | 0.00012 | TGFB1,JAG1,NOTCH1,IFT122,TSC1,CTNNB1,SKI,BMP7,PAX6 |
| **GO:0007010** | cytoskeleton organization | 16 | 953 | 0.00013 | KRT14,MYH9,ANK1,WDPCP,CTNNB1,ACTA1,FGD1,BRCA2,ACTN1,FGD4,CC2D2A,PAX6,BRCA1,FLNB,ACTG1,MYH3 |
| **GO:0046488** | phosphatidylinositol metabolic process | 8 | 211 | 0.00013 | EGFR,ATM,FGFR3,INPP5E,PIGO,ESR1,PIGG,PIK3R1 |
| **GO:0071345** | cellular response to cytokine stimulus | 16 | 953 | 0.00013 | TNFRSF1A,MMP2,TGFB1,EDAR,TDGF1,COL1A2,WDR35,HLA-DRB1,STXBP1,FOXH1,HLA-B,PAX6,BRCA1,FLNB,PIK3R1,ACTG1 |
| **GO:1902533** | positive regulation of intracellular signal transduction | 16 | 959 | 0.00014 | TNFRSF1A,TGFB1,EDAR,EGFR,NOTCH1,ATM,TDGF1,RPS7,FGFR3,CTNNB1,RET,HLA-DRB1,ROR2,CDON,ESR1,PIK3R1 |
| **GO:0010001** | glial cell differentiation | 7 | 154 | 0.00015 | TGFB1,EGFR,NOTCH1,CTNNB1,ROR2,SKI,PAX6 |
| **GO:0010468** | regulation of gene expression | 40 | 4533 | 0.00015 | TNFRSF1A,MYH9,TGFB1,TTC21B,JAG1,EDAR,SMARCB1,EP300,EGFR,NOTCH1,ATM,TSC1,WDR35,FBN1,PTCH1,RPS7,CTNNB1,RET,RPS26,HLA-DRB1,ACTA1,AHI1,RPS27,RPL11,ASXL1,ROR2,FOXH1,KDM6A,SKI,BRCA2,CDON,BMP7,PAX1,CTNND1,PAX6,ESR1,BRCA1,PIK3R1,KMT2A,CEP290 |
| **GO:0022603** | regulation of anatomical structure morphogenesis | 16 | 961 | 0.00015 | TNFRSF1A,MYH9,DHODH,TGFB1,WDPCP,NOTCH1,FUZ,CTNNB1,RET,AHI1,FGD1,ROR2,BMP7,FGD4,ESR1,BRCA1 |
| **GO:0030111** | regulation of Wnt signaling pathway | 9 | 293 | 0.00017 | APC,EGFR,NOTCH1,FUZ,ROR2,SKI,CTNND1,TMEM237,ESR1 |
| **GO:0042325** | regulation of phosphorylation | 20 | 1465 | 0.00017 | TNFRSF1A,TGFB1,FKTN,APC,EDAR,EGFR,NOTCH1,ATM,TDGF1,TSC1,FBN1,FGFR3,CTNNB1,RET,HLA-DRB1,ROR2,CDON,BMP7,PAX6,PIK3R1 |
| **GO:0060831** | smoothened signaling pathway involved in dorsal/ventral neural tube patterning | 3 | 7 | 0.00017 | IFT122,PTCH1,TBC1D32 |
| **GO:0001568** | blood vessel development | 11 | 464 | 0.00018 | MYH9,MMP2,TGFB1,JAG1,NOTCH1,TDGF1,COL1A2,CTNNB1,FOXH1,DYNC2H1,PAX6 |
| **GO:0010721** | negative regulation of cell development | 9 | 298 | 0.00018 | TGFB1,JAG1,NOTCH1,TSC1,FBN1,CTNNB1,SKI,BMP7,PAX6 |
| **GO:0031503** | protein-containing complex localization | 8 | 223 | 0.00018 | TTC21B,ATM,IFT122,TSC1,WDR35,NUP188,DYNC2H1,WDR60 |
| **GO:0044087** | regulation of cellular component biogenesis | 15 | 867 | 0.00018 | TNFRSF1A,TGFB1,APC,EP300,WDPCP,NOTCH1,ATM,TSC1,FUZ,CTNNB1,STXBP1,BMP7,ESR1,BRCA1,PIK3R1 |
| **GO:1902692** | regulation of neuroblast proliferation | 4 | 27 | 0.00018 | TGFB1,NOTCH1,CTNNB1,PAX6 |
| **GO:0051649** | establishment of localization in cell | 21 | 1616 | 0.00020 | MYH9,TGFB1,TTC21B,PHYH,ANK1,ATM,IFT122,TSC1,WDR35,RPS7,CTNNB1,RPS26,RYR1,RPS27,NUP188,STXBP1,RPL11,DYNC2H1,WDR60,PAX6,PIK3R1 |
| **GO:0045165** | cell fate commitment | 8 | 230 | 0.00021 | JAG1,NOTCH1,PTCH1,CTNNB1,PTCH2,ROR2,CDON,PAX6 |
| **GO:0001756** | somitogenesis | 5 | 63 | 0.00022 | EP300,ATM,ROR2,KDM6A,PAX1 |
| **GO:0009913** | epidermal cell differentiation | 9 | 306 | 0.00022 | KRT14,JAG1,WDPCP,NOTCH1,PTCH1,CTNNB1,PTCH2,PAX6,FLNB |
| **GO:0060348** | bone development | 7 | 166 | 0.00022 | TGFB1,EP300,FGFR3,RYR1,ASXL1,SKI,PAX1 |
| **GO:0001932** | regulation of protein phosphorylation | 19 | 1370 | 0.00023 | TNFRSF1A,TGFB1,FKTN,APC,EDAR,EGFR,NOTCH1,ATM,TDGF1,TSC1,FBN1,FGFR3,CTNNB1,RET,HLA-DRB1,ROR2,CDON,BMP7,PAX6 |
| **GO:0009880** | embryonic pattern specification | 5 | 64 | 0.00024 | TDGF1,PTCH1,CTNNB1,KDM6A,BMP7 |
| **GO:0001763** | morphogenesis of a branching structure | 7 | 169 | 0.00025 | TGFB1,NOTCH1,TDGF1,PTCH1,CTNNB1,BMP7,ESR1 |
| **GO:0071417** | cellular response to organonitrogen compound | 11 | 485 | 0.00025 | MMP2,TGFB1,APC,EGFR,COL1A2,FBN1,CTNNB1,RYR1,ACTA1,BRCA1,PIK3R1 |
| **GO:0002682** | regulation of immune system process | 19 | 1391 | 0.00028 | MMP2,TGFB1,JAG1,EP300,NOTCH1,ATM,CD96,COL1A2,FBN1,CTNNB1,HLA-DRB1,STXBP1,ROR2,HLA-B,ESR1,COLEC11,PIK3R1,KMT2A,ACTG1 |
| **GO:0040011** | locomotion | 17 | 1144 | 0.00028 | MYH9,TGFB1,APC,WDPCP,EGFR,NOTCH1,TDGF1,COL1A2,PTCH1,CTNNB1,RET,NUP188,ROR2,SKI,BMP7,PAX6,PIK3R1 |
| **GO:0045601** | regulation of endothelial cell differentiation | 4 | 31 | 0.00028 | TNFRSF1A,JAG1,NOTCH1,CTNNB1 |
| **GO:0060325** | face morphogenesis | 4 | 31 | 0.00028 | MMP2,TGFB1,SKI,MYH3 |
| **GO:0007368** | determination of left/right symmetry | 6 | 115 | 0.00029 | NOTCH1,AHI1,FOXH1,DYNC2H1,TBC1D32,CC2D2A |
| **GO:0045930** | negative regulation of mitotic cell cycle | 8 | 243 | 0.00029 | TGFB1,APC,EP300,EGFR,ATM,CTNNB1,BMP7,BRCA1 |
| **GO:0001837** | epithelial to mesenchymal transition | 5 | 68 | 0.00030 | TGFB1,JAG1,NOTCH1,CTNNB1,BMP7 |
| **GO:0048870** | cell motility | 15 | 914 | 0.00030 | MYH9,TGFB1,APC,WDPCP,EGFR,NOTCH1,TDGF1,COL1A2,CTNNB1,RET,NUP188,ROR2,SKI,PAX6,PIK3R1 |
| **GO:0072657** | protein localization to membrane | 10 | 405 | 0.00030 | TNFRSF1A,ANK1,EGFR,IFT122,PTCH1,RPS7,RPS26,RPS27,STXBP1,RPL11 |
| **GO:0048839** | inner ear development | 7 | 177 | 0.00031 | TGFB1,JAG1,WDPCP,NOTCH1,AHI1,ROR2,CEP290 |
| **GO:0002064** | epithelial cell development | 7 | 179 | 0.00033 | JAG1,WDPCP,NOTCH1,CTNNB1,PAX6,ESR1,FLNB |
| **GO:0016477** | cell migration | 14 | 812 | 0.00033 | MYH9,TGFB1,APC,WDPCP,EGFR,NOTCH1,TDGF1,COL1A2,CTNNB1,RET,NUP188,ROR2,PAX6,PIK3R1 |
| **GO:0021532** | neural tube patterning | 4 | 33 | 0.00033 | IFT122,PTCH1,TBC1D32,PAX6 |
| **GO:0045637** | regulation of myeloid cell differentiation | 8 | 249 | 0.00033 | JAG1,EP300,FBN1,CTNNB1,HLA-DRB1,ROR2,PIK3R1,KMT2A |
| **GO:1903054** | negative regulation of extracellular matrix organization | 3 | 10 | 0.00034 | TNFRSF1A,TGFB1,NOTCH1 |
| **GO:0007017** | microtubule-based process | 12 | 605 | 0.00035 | MYH9,TTC21B,IFT122,WDR35,CTNNB1,BRCA2,DYNC2H1,WDR60,CC2D2A,PAX6,BRCA1,MYH3 |
| **GO:0071705** | nitrogen compound transport | 21 | 1690 | 0.00035 | TCN2,MYH9,TGFB1,TTC21B,PHYH,NOTCH1,IFT122,TSC1,FUZ,WDR35,FRAS1,RPS7,RPS26,RPS27,NUP188,STXBP1,RPL11,DYNC2H1,WDR60,PIK3R1,CEP290 |
| **GO:0072006** | nephron development | 6 | 121 | 0.00035 | JAG1,NOTCH1,PTCH1,CTNNB1,AHI1,BMP7 |
| **GO:0001942** | hair follicle development | 5 | 72 | 0.00036 | EDAR,EGFR,NOTCH1,FUZ,CTNNB1 |
| **GO:0035315** | hair cell differentiation | 4 | 34 | 0.00036 | JAG1,WDPCP,NOTCH1,CTNNB1 |
| **GO:0043408** | regulation of MAPK cascade | 13 | 712 | 0.00036 | TGFB1,FKTN,EDAR,EGFR,NOTCH1,TDGF1,FGFR3,CTNNB1,RET,HLA-DRB1,ROR2,CDON,BMP7 |
| **GO:0097421** | liver regeneration | 4 | 34 | 0.00036 | TGFB1,EGFR,WDR35,PTCH1 |
| **GO:0043410** | positive regulation of MAPK cascade | 11 | 512 | 0.00037 | TGFB1,EDAR,EGFR,NOTCH1,TDGF1,FGFR3,CTNNB1,RET,HLA-DRB1,ROR2,CDON |
| **GO:0030182** | neuron differentiation | 15 | 940 | 0.00038 | JAG1,WDPCP,EGFR,NOTCH1,PTCH1,CTNNB1,RET,AHI1,STXBP1,SKI,BMP7,DYNC2H1,PAX6,PIK3R1,CEP290 |
| **GO:0001934** | positive regulation of protein phosphorylation | 15 | 941 | 0.00039 | TNFRSF1A,TGFB1,EDAR,EGFR,NOTCH1,ATM,TDGF1,FBN1,FGFR3,CTNNB1,RET,HLA-DRB1,ROR2,CDON,BMP7 |
| **GO:0000578** | embryonic axis specification | 4 | 36 | 0.00042 | TDGF1,PTCH1,CTNNB1,KDM6A |
| **GO:0006886** | intracellular protein transport | 14 | 836 | 0.00042 | TGFB1,TTC21B,PHYH,IFT122,TSC1,WDR35,RPS7,RPS26,RPS27,NUP188,RPL11,DYNC2H1,WDR60,PIK3R1 |
| **GO:0010559** | regulation of glycoprotein biosynthetic process | 4 | 36 | 0.00042 | FKTN,POMT2,CTNNB1,POMT1 |
| **GO:0048565** | digestive tract development | 6 | 126 | 0.00042 | TGFB1,EGFR,NOTCH1,CTNNB1,RET,AHI1 |
| **GO:0048589** | developmental growth | 9 | 340 | 0.00042 | TGFB1,NOTCH1,ATM,FGFR3,CTNNB1,LARGE,KDM6A,BRCA2,ESR1 |
| **GO:0050768** | negative regulation of neurogenesis | 8 | 260 | 0.00042 | TGFB1,JAG1,NOTCH1,TSC1,CTNNB1,SKI,BMP7,PAX6 |
| **GO:0060255** | regulation of macromolecule metabolic process | 47 | 6072 | 0.00042 | TNFRSF1A,MYH9,TGFB1,FKTN,TTC21B,JAG1,APC,EDAR,POMT2,SMARCB1,EP300,EGFR,NOTCH1,ATM,TDGF1,TSC1,WDR35,FBN1,PTCH1,RPS7,FGFR3,CTNNB1,RET,RPS26,HLA-DRB1,ACTA1,AHI1,RPS27,POMT1,RPL11,ASXL1,ROR2,FOXH1,KDM6A,SKI,BRCA2,CDON,ACTN1,BMP7,PAX1,CTNND1,PAX6,ESR1,BRCA1,PIK3R1,KMT2A,CEP290 |
| **GO:0060541** | respiratory system development | 7 | 188 | 0.00042 | EP300,WDPCP,EGFR,NOTCH1,CTNNB1,ASXL1,SKI |
| **GO:2000615** | regulation of histone H3-K9 acetylation | 3 | 11 | 0.00042 | SMARCB1,BRCA1,KMT2A |
| **GO:0035050** | embryonic heart tube development | 5 | 76 | 0.00044 | NOTCH1,IFT122,CTNNB1,AHI1,FOXH1 |
| **GO:0072080** | nephron tubule development | 5 | 77 | 0.00046 | JAG1,NOTCH1,PTCH1,CTNNB1,AHI1 |
| **GO:0048762** | mesenchymal cell differentiation | 6 | 130 | 0.00048 | TGFB1,JAG1,NOTCH1,CTNNB1,RET,BMP7 |
| **GO:0051129** | negative regulation of cellular component organization | 12 | 632 | 0.00048 | TNFRSF1A,TGFB1,APC,SMARCB1,EP300,NOTCH1,ATM,TSC1,STXBP1,SKI,BMP7,BRCA1 |
| **GO:0051338** | regulation of transferase activity | 15 | 964 | 0.00048 | TGFB1,APC,EGFR,TDGF1,TSC1,FBN1,RPS7,FGFR3,CTNNB1,RET,HLA-DRB1,RPL11,ROR2,BMP7,PIK3R1 |
| **GO:1905330** | regulation of morphogenesis of an epithelium | 6 | 130 | 0.00048 | TGFB1,CTNNB1,AHI1,ROR2,BMP7,ESR1 |
| **GO:0009725** | response to hormone | 14 | 854 | 0.00051 | TGFB1,APC,EGFR,NOTCH1,TSC1,FBN1,PTCH1,CTNNB1,ACTA1,STXBP1,BMP7,ESR1,BRCA1,PIK3R1 |
| **GO:0031100** | animal organ regeneration | 5 | 79 | 0.00051 | TGFB1,EGFR,NOTCH1,WDR35,PTCH1 |
| **GO:2000177** | regulation of neural precursor cell proliferation | 5 | 79 | 0.00051 | TGFB1,NOTCH1,CTNNB1,CDON,PAX6 |
| **GO:0006950** | response to stress | 31 | 3267 | 0.00052 | TNFRSF1A,MMP2,DHODH,TGFB1,APC,POMT2,SMARCB1,EP300,EGFR,NOTCH1,ATM,CD96,TDGF1,COL1A2,LARGE,RET,RYR1,HLA-DRB1,POMT1,STXBP1,ROR2,BRCA2,BMP7,HLA-B,PAX6,COLEC11,BRCA1,FLNB,PIK3R1,ACTG1,TMCO1 |
| **GO:0034330** | cell junction organization | 7 | 197 | 0.00052 | KRT14,TGFB1,APC,CTNNB1,ACTN1,CTNND1,ACTG1 |
| **GO:0048754** | branching morphogenesis of an epithelial tube | 6 | 134 | 0.00054 | TGFB1,NOTCH1,PTCH1,CTNNB1,BMP7,ESR1 |
| **GO:0071702** | organic substance transport | 23 | 2040 | 0.00054 | TCN2,MYH9,TGFB1,TTC21B,PHYH,NOTCH1,IFT122,TSC1,FUZ,WDR35,FRAS1,RPS7,RPS26,SLC2A10,RPS27,ABCA4,NUP188,STXBP1,RPL11,DYNC2H1,WDR60,PIK3R1,CEP290 |
| **GO:0010717** | regulation of epithelial to mesenchymal transition | 5 | 81 | 0.00056 | TGFB1,NOTCH1,FUZ,CTNNB1,BMP7 |
| **GO:0030334** | regulation of cell migration | 13 | 753 | 0.00056 | TGFB1,JAG1,APC,WDPCP,EGFR,NOTCH1,TDGF1,FUZ,RET,ROR2,BMP7,PAX6,PIK3R1 |
| **GO:0006661** | phosphatidylinositol biosynthetic process | 6 | 136 | 0.00057 | ATM,FGFR3,INPP5E,PIGO,PIGG,PIK3R1 |
| **GO:0060041** | retina development in camera-type eye | 6 | 136 | 0.00057 | RET,AHI1,SKI,CDON,TBC1D32,PAX6 |
| **GO:0030855** | epithelial cell differentiation | 12 | 649 | 0.00058 | KRT14,TGFB1,JAG1,WDPCP,NOTCH1,PTCH1,CTNNB1,PTCH2,BMP7,PAX6,ESR1,FLNB |
| **GO:0048666** | neuron development | 13 | 758 | 0.00059 | WDPCP,EGFR,NOTCH1,PTCH1,CTNNB1,RET,AHI1,STXBP1,SKI,BMP7,PAX6,PIK3R1,CEP290 |
| **GO:0055001** | muscle cell development | 6 | 137 | 0.00059 | NOTCH1,RYR1,ACTA1,SKI,ACTG1,MYH3 |
| **GO:0030850** | prostate gland development | 4 | 41 | 0.00061 | NOTCH1,PTCH1,CTNNB1,ESR1 |
| **GO:0048145** | regulation of fibroblast proliferation | 5 | 84 | 0.00063 | TGFB1,EGFR,CTNNB1,SKI,ESR1 |
| **GO:0030900** | forebrain development | 9 | 366 | 0.00065 | TTC21B,EGFR,NOTCH1,TSC1,CTNNB1,SKI,CDON,DYNC2H1,PAX6 |
| **GO:2000027** | regulation of animal organ morphogenesis | 7 | 207 | 0.00066 | TGFB1,NOTCH1,CTNNB1,AHI1,ROR2,BMP7,ESR1 |
| **GO:0060049** | regulation of protein glycosylation | 3 | 14 | 0.00067 | FKTN,POMT2,POMT1 |
| **GO:1902337** | regulation of apoptotic process involved in morphogenesis | 3 | 14 | 0.00067 | TNFRSF1A,NOTCH1,BMP7 |
| **GO:0001657** | ureteric bud development | 5 | 86 | 0.00069 | TGFB1,PTCH1,CTNNB1,RET,BMP7 |
| **GO:0051270** | regulation of cellular component movement | 14 | 886 | 0.00069 | TGFB1,JAG1,APC,WDPCP,EGFR,NOTCH1,TDGF1,FUZ,RET,ROR2,ACTN1,BMP7,PAX6,PIK3R1 |
| **GO:0022008** | neurogenesis | 19 | 1519 | 0.00070 | TGFB1,JAG1,WDPCP,EGFR,NOTCH1,TSC1,PTCH1,CTNNB1,RET,AHI1,STXBP1,ROR2,SKI,CDON,BMP7,DYNC2H1,PAX6,PIK3R1,CEP290 |
| **GO:0097327** | response to antineoplastic agent | 5 | 87 | 0.00072 | TGFB1,EGFR,WDR35,CTNNB1,BRCA1 |
| **GO:0048538** | thymus development | 4 | 44 | 0.00075 | ATM,CTNNB1,ASXL1,PAX1 |
| **GO:0001503** | ossification | 7 | 215 | 0.00080 | MMP2,TGFB1,EGFR,FGFR3,RYR1,ROR2,BMP7 |
| **GO:0008283** | cell population proliferation | 12 | 676 | 0.00080 | TGFB1,EGFR,NOTCH1,PTCH1,FGFR3,CTNNB1,RPS27,SKI,BRCA2,BMP7,PAX1,ESR1 |
| **GO:0010718** | positive regulation of epithelial to mesenchymal transition | 4 | 45 | 0.00080 | TGFB1,NOTCH1,CTNNB1,BMP7 |
| **GO:0021700** | developmental maturation | 7 | 216 | 0.00082 | MMP2,FGFR3,CTNNB1,RET,RYR1,STXBP1,BRCA2 |
| **GO:0090130** | tissue migration | 5 | 90 | 0.00082 | MYH9,WDPCP,TDGF1,ACTA1,NUP188 |
| **GO:0097190** | apoptotic signaling pathway | 8 | 295 | 0.00083 | TNFRSF1A,TGFB1,EP300,ATM,FGFR3,BRCA2,BRCA1,PIK3R1 |
| **GO:0008593** | regulation of Notch signaling pathway | 5 | 91 | 0.00085 | JAG1,EP300,EGFR,NOTCH1,BMP7 |
| **GO:0045597** | positive regulation of cell differentiation | 14 | 908 | 0.00085 | TGFB1,JAG1,NOTCH1,PTCH1,CTNNB1,RET,HLA-DRB1,AHI1,PTCH2,ROR2,ADAMTS20,CDON,BMP7,PAX6 |
| **GO:0048708** | astrocyte differentiation | 4 | 46 | 0.00085 | EGFR,NOTCH1,ROR2,PAX6 |
| **GO:0033043** | regulation of organelle organization | 16 | 1155 | 0.00086 | DHODH,TGFB1,APC,SMARCB1,ANK1,ATM,TSC1,FUZ,WDR35,CTNNB1,STXBP1,SKI,BMP7,BRCA1,PIK3R1,KMT2A |
| **GO:0036293** | response to decreased oxygen levels | 8 | 298 | 0.00088 | MMP2,TGFB1,EP300,NOTCH1,ATM,TSC1,RYR1,BMP7 |
| **GO:0051571** | positive regulation of histone H3-K4 methylation | 3 | 16 | 0.00088 | CTNNB1,BRCA1,KMT2A |
| **GO:1903798** | regulation of production of miRNAs involved in gene silencing by miRNA | 3 | 16 | 0.00088 | TGFB1,EGFR,ESR1 |
| **GO:0031099** | regeneration | 6 | 151 | 0.00090 | TGFB1,EGFR,NOTCH1,WDR35,PTCH1,LARGE |
| **GO:0048699** | generation of neurons | 18 | 1422 | 0.00091 | TGFB1,JAG1,WDPCP,EGFR,NOTCH1,TSC1,PTCH1,CTNNB1,RET,AHI1,STXBP1,SKI,CDON,BMP7,DYNC2H1,PAX6,PIK3R1,CEP290 |
| **GO:0048546** | digestive tract morphogenesis | 4 | 48 | 0.00097 | EGFR,NOTCH1,CTNNB1,AHI1 |
| **GO:0061383** | trabecula morphogenesis | 4 | 48 | 0.00097 | MMP2,NOTCH1,FOXH1,BMP7 |
| **GO:0080134** | regulation of response to stress | 17 | 1299 | 0.00098 | TNFRSF1A,MMP2,FKTN,EDAR,EP300,EGFR,ATM,CD96,TSC1,CTNNB1,HLA-DRB1,ROR2,BMP7,HLA-B,ESR1,BRCA1,PIK3R1 |
| **GO:0097711** | ciliary basal body-plasma membrane docking | 5 | 95 | 0.00099 | TCTN2,AHI1,RPGRIP1L,CC2D2A,CEP290 |
| **GO:0001701** | in utero embryonic development | 8 | 306 | 0.0010 | MYH9,EGFR,NOTCH1,PTCH1,CTNNB1,KDM6A,BRCA2,BMP7 |
| **GO:0030098** | lymphocyte differentiation | 7 | 226 | 0.0010 | TGFB1,EP300,ATM,TSC1,CTNNB1,PAX1,PIK3R1 |
| **GO:0031326** | regulation of cellular biosynthetic process | 36 | 4266 | 0.0010 | TNFRSF1A,TGFB1,FKTN,TTC21B,JAG1,POMT2,SMARCB1,EP300,EGFR,NOTCH1,ATM,TSC1,WDR35,FBN1,PTCH1,CTNNB1,RET,HLA-DRB1,AHI1,POMT1,ASXL1,ROR2,FOXH1,SKI,BRCA2,CDON,ACTN1,BMP7,PAX1,CTNND1,PAX6,ESR1,BRCA1,PIK3R1,KMT2A,CEP290 |
| **GO:0031400** | negative regulation of protein modification process | 11 | 592 | 0.0010 | TGFB1,FKTN,APC,SMARCB1,RPS7,CTNNB1,RPL11,SKI,BMP7,PAX6,BRCA1 |
| **GO:0051897** | positive regulation of protein kinase B signaling | 6 | 157 | 0.0010 | TGFB1,EGFR,FGFR3,RET,ESR1,PIK3R1 |
| **GO:0071346** | cellular response to interferon-gamma | 6 | 155 | 0.0010 | TDGF1,HLA-DRB1,STXBP1,HLA-B,FLNB,ACTG1 |
| **GO:2000179** | positive regulation of neural precursor cell proliferation | 4 | 49 | 0.0010 | NOTCH1,CTNNB1,CDON,PAX6 |
| **GO:0002521** | leukocyte differentiation | 8 | 313 | 0.0011 | MYH9,TGFB1,EP300,ATM,TSC1,CTNNB1,PAX1,PIK3R1 |
| **GO:0007169** | transmembrane receptor protein tyrosine kinase signaling pathway | 10 | 499 | 0.0011 | MMP2,TGFB1,APC,EGFR,FGFR3,RET,AHI1,ROR2,PIK3R1,ACTG1 |
| **GO:0009948** | anterior/posterior axis specification | 4 | 51 | 0.0011 | TDGF1,CTNNB1,KDM6A,SKI |
| **GO:0033044** | regulation of chromosome organization | 8 | 313 | 0.0011 | TGFB1,APC,SMARCB1,ATM,CTNNB1,SKI,BRCA1,KMT2A |
| **GO:0035065** | regulation of histone acetylation | 4 | 51 | 0.0011 | TGFB1,SMARCB1,BRCA1,KMT2A |
| **GO:0048799** | animal organ maturation | 3 | 18 | 0.0011 | FGFR3,RET,RYR1 |
| **GO:0050678** | regulation of epithelial cell proliferation | 8 | 311 | 0.0011 | TGFB1,EGFR,NOTCH1,IFT122,PTCH1,CTNNB1,BRCA2,PAX6 |
| **GO:0140056** | organelle localization by membrane tethering | 6 | 159 | 0.0011 | TCTN2,AHI1,STXBP1,RPGRIP1L,CC2D2A,CEP290 |
| **GO:0007346** | regulation of mitotic cell cycle | 11 | 608 | 0.0012 | TGFB1,APC,EP300,EGFR,ATM,PTCH1,CTNNB1,BRCA2,BMP7,BRCA1,CEP290 |
| **GO:0010556** | regulation of macromolecule biosynthetic process | 35 | 4143 | 0.0012 | TNFRSF1A,TGFB1,FKTN,TTC21B,JAG1,POMT2,SMARCB1,EP300,EGFR,NOTCH1,ATM,TSC1,FBN1,PTCH1,CTNNB1,RET,HLA-DRB1,AHI1,POMT1,ASXL1,ROR2,FOXH1,SKI,BRCA2,CDON,ACTN1,BMP7,PAX1,CTNND1,PAX6,ESR1,BRCA1,PIK3R1,KMT2A,CEP290 |
| **GO:0021515** | cell differentiation in spinal cord | 4 | 52 | 0.0012 | NOTCH1,PTCH1,DYNC2H1,PAX6 |
| **GO:0048608** | reproductive structure development | 9 | 405 | 0.0012 | EGFR,NOTCH1,ATM,PTCH1,CTNNB1,ROR2,BRCA2,BMP7,ESR1 |
| **GO:0003170** | heart valve development | 4 | 53 | 0.0013 | TNFRSF1A,TGFB1,JAG1,NOTCH1 |
| **GO:0003177** | pulmonary valve development | 3 | 19 | 0.0013 | TNFRSF1A,JAG1,NOTCH1 |
| **GO:0003215** | cardiac right ventricle morphogenesis | 3 | 19 | 0.0013 | JAG1,NOTCH1,FOXH1 |
| **GO:0010560** | positive regulation of glycoprotein biosynthetic process | 3 | 19 | 0.0013 | POMT2,CTNNB1,POMT1 |
| **GO:0021510** | spinal cord development | 5 | 103 | 0.0013 | NOTCH1,IFT122,PTCH1,DYNC2H1,PAX6 |
| **GO:0030220** | platelet formation | 3 | 19 | 0.0013 | MYH9,EP300,ACTN1 |
| **GO:0046854** | phosphatidylinositol phosphorylation | 5 | 103 | 0.0013 | EGFR,ATM,FGFR3,ESR1,PIK3R1 |
| **GO:0048545** | response to steroid hormone | 8 | 324 | 0.0013 | TGFB1,EGFR,NOTCH1,CTNNB1,ACTA1,BMP7,ESR1,BRCA1 |
| **GO:0050821** | protein stabilization | 6 | 166 | 0.0013 | EP300,TSC1,RPS7,STXBP1,RPL11,PIK3R1 |
| **GO:0060688** | regulation of morphogenesis of a branching structure | 4 | 53 | 0.0013 | TGFB1,CTNNB1,BMP7,ESR1 |
| **GO:0080135** | regulation of cellular response to stress | 11 | 618 | 0.0013 | FKTN,EDAR,EP300,EGFR,ATM,TSC1,CTNNB1,ROR2,BMP7,BRCA1,PIK3R1 |
| **GO:0090183** | regulation of kidney development | 4 | 54 | 0.0013 | TGFB1,CTNNB1,RET,BMP7 |
| **GO:0003006** | developmental process involved in reproduction | 11 | 622 | 0.0014 | TGFB1,EGFR,NOTCH1,ATM,PTCH1,CTNNB1,STXBP1,ROR2,BRCA2,BMP7,ESR1 |
| **GO:0042592** | homeostatic process | 18 | 1491 | 0.0014 | TGFB1,EGFR,NOTCH1,ATM,WDR35,FBN1,PTCH1,CTNNB1,LARGE,RYR1,ABCA4,ASXL1,BRCA2,PAX6,ESR1,PIK3R1,KMT2A,TMCO1 |
| **GO:0051171** | regulation of nitrogen compound metabolic process | 44 | 5827 | 0.0014 | TNFRSF1A,MYH9,TGFB1,FKTN,TTC21B,JAG1,APC,EDAR,POMT2,SMARCB1,EP300,EGFR,NOTCH1,ATM,TDGF1,TSC1,WDR35,FBN1,PTCH1,RPS7,FGFR3,CTNNB1,RET,RPS26,HLA-DRB1,AHI1,POMT1,RPL11,ASXL1,ROR2,FOXH1,SKI,BRCA2,CDON,ACTN1,BMP7,PAX1,CTNND1,PAX6,ESR1,BRCA1,PIK3R1,KMT2A,CEP290 |
| **GO:2000377** | regulation of reactive oxygen species metabolic process | 6 | 169 | 0.0014 | TGFB1,EGFR,WDR35,BMP7,ESR1,BRCA1 |
| **GO:0002052** | positive regulation of neuroblast proliferation | 3 | 21 | 0.0015 | NOTCH1,CTNNB1,PAX6 |
| **GO:0002683** | negative regulation of immune system process | 9 | 425 | 0.0015 | TGFB1,NOTCH1,ATM,CD96,FBN1,CTNNB1,HLA-DRB1,HLA-B,PIK3R1 |
| **GO:0003176** | aortic valve development | 3 | 21 | 0.0015 | TNFRSF1A,JAG1,NOTCH1 |
| **GO:0009314** | response to radiation | 9 | 425 | 0.0015 | TGFB1,EP300,EGFR,ATM,ABCA4,BRCA2,BRCA1,PIK3R1,KMT2A |
| **GO:0019538** | protein metabolic process | 35 | 4194 | 0.0015 | MYH9,MMP2,TGFB1,FKTN,APC,POMT2,EP300,EGFR,ATM,TDGF1,FBN1,PTCH1,RPS7,FGFR3,LARGE,RET,RPS26,RPS27,POMT1,RPL11,ASXL1,ROR2,KDM6A,PIGO,BRCA2,ADAMTS20,DYNC2H1,PAX6,ESR1,COLEC11,PIGG,BRCA1,PIK3R1,KMT2A,MYH3 |
| **GO:0030258** | lipid modification | 7 | 245 | 0.0015 | PHYH,EGFR,ATM,FGFR3,INPP5E,ESR1,PIK3R1 |
| **GO:0060972** | left/right pattern formation | 3 | 21 | 0.0015 | NOTCH1,IFT122,AHI1 |
| **GO:0021987** | cerebral cortex development | 5 | 109 | 0.0016 | EGFR,TSC1,CTNNB1,CDON,PAX6 |
| **GO:0031060** | regulation of histone methylation | 4 | 58 | 0.0016 | SMARCB1,CTNNB1,BRCA1,KMT2A |
| **GO:0060395** | SMAD protein signal transduction | 4 | 58 | 0.0016 | TGFB1,ROR2,SKI,BMP7 |
| **GO:0003332** | negative regulation of extracellular matrix constituent secretion | 2 | 3 | 0.0017 | TNFRSF1A,NOTCH1 |
| **GO:0009957** | epidermal cell fate specification | 2 | 3 | 0.0017 | PTCH1,PTCH2 |
| **GO:0051968** | positive regulation of synaptic transmission, glutamatergic | 3 | 22 | 0.0017 | EGFR,STXBP1,ROR2 |
| **GO:1904100** | positive regulation of protein O-linked glycosylation | 2 | 3 | 0.0017 | POMT2,POMT1 |
| **GO:2000112** | regulation of cellular macromolecule biosynthetic process | 34 | 4050 | 0.0017 | TNFRSF1A,TGFB1,FKTN,TTC21B,JAG1,POMT2,SMARCB1,EP300,EGFR,NOTCH1,ATM,TSC1,FBN1,PTCH1,CTNNB1,RET,HLA-DRB1,AHI1,POMT1,ASXL1,ROR2,FOXH1,SKI,BRCA2,CDON,BMP7,PAX1,CTNND1,PAX6,ESR1,BRCA1,PIK3R1,KMT2A,CEP290 |
| **GO:0044403** | symbiont process | 11 | 650 | 0.0019 | TNFRSF1A,TGFB1,SMARCB1,EP300,EGFR,CTNNB1,HLA-DRB1,NUP188,CTNND1,HLA-B,PIK3R1 |
| **GO:0048741** | skeletal muscle fiber development | 3 | 23 | 0.0019 | RYR1,ACTA1,SKI |
| **GO:0051130** | positive regulation of cellular component organization | 15 | 1128 | 0.0019 | TGFB1,APC,SMARCB1,ANK1,ATM,TSC1,FUZ,WDR35,CTNNB1,RET,AHI1,BMP7,ESR1,BRCA1,KMT2A |
| **GO:0002761** | regulation of myeloid leukocyte differentiation | 5 | 116 | 0.0020 | FBN1,CTNNB1,HLA-DRB1,ROR2,PIK3R1 |
| **GO:1902105** | regulation of leukocyte differentiation | 7 | 261 | 0.0020 | TGFB1,FBN1,CTNNB1,HLA-DRB1,ROR2,HLA-B,PIK3R1 |
| **GO:0019827** | stem cell population maintenance | 5 | 118 | 0.0021 | JAG1,NOTCH1,TDGF1,CTNNB1,SKI |
| **GO:0045606** | positive regulation of epidermal cell differentiation | 3 | 24 | 0.0021 | NOTCH1,PTCH1,PTCH2 |
| **GO:0048812** | neuron projection morphogenesis | 9 | 448 | 0.0021 | EGFR,NOTCH1,PTCH1,CTNNB1,RET,STXBP1,BMP7,PAX6,PIK3R1 |
| **GO:2001251** | negative regulation of chromosome organization | 5 | 117 | 0.0021 | APC,SMARCB1,ATM,SKI,BRCA1 |
| **GO:0010638** | positive regulation of organelle organization | 10 | 552 | 0.0022 | TGFB1,SMARCB1,ANK1,ATM,TSC1,FUZ,WDR35,CTNNB1,BRCA1,KMT2A |
| **GO:1901701** | cellular response to oxygen-containing compound | 13 | 896 | 0.0022 | MMP2,TGFB1,APC,EGFR,COL1A2,WDR35,FBN1,PTCH1,CTNNB1,RET,ESR1,BRCA1,PIK3R1 |
| **GO:0006810** | transport | 34 | 4130 | 0.0023 | TCN2,MYH9,DHODH,TGFB1,TTC21B,PHYH,ANK1,NOTCH1,IFT122,TSC1,FUZ,WDR35,FRAS1,RPS7,CTNNB1,RPS26,SLC2A10,RYR1,AHI1,RPS27,ABCA4,NUP188,STXBP1,RPL11,CHRNG,ACTN1,DYNC2H1,WDR60,HLA-B,COLEC11,PIK3R1,CEP290,ACTG1,TMCO1 |
| **GO:0007178** | transmembrane receptor protein serine/threonine kinase signaling pathway | 6 | 189 | 0.0023 | TGFB1,COL1A2,ROR2,FOXH1,SKI,BMP7 |
| **GO:0009605** | response to external stimulus | 22 | 2152 | 0.0023 | TNFRSF1A,DHODH,TGFB1,EGFR,NOTCH1,CD96,TDGF1,TSC1,WDR35,PTCH1,RET,HLA-DRB1,ACTA1,ABCA4,STXBP1,BMP7,HLA-B,PAX6,COLEC11,FLNB,PIK3R1,ACTG1 |
| **GO:0030335** | positive regulation of cell migration | 9 | 452 | 0.0023 | TGFB1,APC,EGFR,NOTCH1,TDGF1,RET,ROR2,BMP7,PIK3R1 |
| **GO:0042493** | response to drug | 13 | 900 | 0.0023 | TCN2,DHODH,TGFB1,JAG1,EGFR,NOTCH1,WDR35,PTCH1,CTNNB1,RET,RYR1,CHRNG,BRCA1 |
| **GO:0046649** | lymphocyte activation | 8 | 358 | 0.0023 | MYH9,TGFB1,EP300,ATM,TSC1,CTNNB1,PAX1,PIK3R1 |
| **GO:0051094** | positive regulation of developmental process | 16 | 1286 | 0.0023 | TNFRSF1A,TGFB1,JAG1,NOTCH1,PTCH1,CTNNB1,RET,HLA-DRB1,AHI1,PTCH2,ROR2,ADAMTS20,CDON,BMP7,PAX6,BRCA1 |
| **GO:0051172** | negative regulation of nitrogen compound metabolic process | 23 | 2307 | 0.0023 | TGFB1,FKTN,APC,SMARCB1,EP300,EGFR,NOTCH1,ATM,TSC1,WDR35,PTCH1,RPS7,CTNNB1,RPS26,RPL11,FOXH1,SKI,BRCA2,BMP7,PAX6,ESR1,BRCA1,KMT2A |
| **GO:0055002** | striated muscle cell development | 5 | 121 | 0.0023 | RYR1,ACTA1,SKI,ACTG1,MYH3 |
| **GO:0071695** | anatomical structure maturation | 5 | 121 | 0.0023 | MMP2,FGFR3,RET,RYR1,BRCA2 |
| **GO:0080090** | regulation of primary metabolic process | 44 | 5982 | 0.0023 | TNFRSF1A,MYH9,TGFB1,FKTN,TTC21B,JAG1,APC,EDAR,POMT2,SMARCB1,EP300,EGFR,NOTCH1,ATM,TDGF1,TSC1,WDR35,FBN1,PTCH1,RPS7,FGFR3,CTNNB1,RET,RPS26,HLA-DRB1,AHI1,POMT1,RPL11,ASXL1,ROR2,FOXH1,SKI,BRCA2,CDON,ACTN1,BMP7,PAX1,CTNND1,PAX6,ESR1,BRCA1,PIK3R1,KMT2A,CEP290 |
| **GO:0050680** | negative regulation of epithelial cell proliferation | 5 | 122 | 0.0024 | TGFB1,IFT122,PTCH1,BRCA2,PAX6 |
| **GO:0090521** | glomerular visceral epithelial cell migration | 2 | 4 | 0.0024 | WDPCP,NUP188 |
| **GO:0001889** | liver development | 5 | 123 | 0.0025 | TGFB1,EGFR,NOTCH1,WDR35,PTCH1 |
| **GO:0030879** | mammary gland development | 5 | 123 | 0.0025 | DHODH,TGFB1,TDGF1,PTCH1,ESR1 |
| **GO:0042491** | inner ear auditory receptor cell differentiation | 3 | 26 | 0.0025 | JAG1,WDPCP,NOTCH1 |
| **GO:0045992** | negative regulation of embryonic development | 3 | 26 | 0.0025 | FUZ,CTNNB1,BMP7 |
| **GO:0090090** | negative regulation of canonical Wnt signaling pathway | 5 | 123 | 0.0025 | APC,NOTCH1,FUZ,ROR2,CTNND1 |
| **GO:2000736** | regulation of stem cell differentiation | 4 | 67 | 0.0025 | JAG1,NOTCH1,BMP7,KMT2A |
| **GO:0007018** | microtubule-based movement | 7 | 276 | 0.0026 | MYH9,TTC21B,IFT122,WDR35,DYNC2H1,WDR60,MYH3 |
| **GO:0045995** | regulation of embryonic development | 5 | 125 | 0.0026 | WDPCP,NOTCH1,FUZ,CTNNB1,BMP7 |
| **GO:0045844** | positive regulation of striated muscle tissue development | 4 | 69 | 0.0027 | TGFB1,NOTCH1,CTNNB1,CDON |
| **GO:0051640** | organelle localization | 10 | 574 | 0.0027 | MYH9,ATM,TCTN2,CTNNB1,AHI1,STXBP1,RPGRIP1L,CC2D2A,PAX6,CEP290 |
| **GO:0071383** | cellular response to steroid hormone stimulus | 6 | 197 | 0.0027 | TGFB1,EGFR,CTNNB1,BMP7,ESR1,BRCA1 |
| **GO:0003206** | cardiac chamber morphogenesis | 5 | 128 | 0.0028 | TGFB1,JAG1,NOTCH1,FOXH1,BMP7 |
| **GO:0035066** | positive regulation of histone acetylation | 3 | 28 | 0.0029 | TGFB1,SMARCB1,BRCA1 |
| **GO:0045671** | negative regulation of osteoclast differentiation | 3 | 28 | 0.0029 | FBN1,CTNNB1,PIK3R1 |
| **GO:0065009** | regulation of molecular function | 29 | 3322 | 0.0029 | TGFB1,JAG1,APC,SMARCB1,EP300,ARHGAP31,EGFR,NOTCH1,TDGF1,TSC1,WDR35,FBN1,PTCH1,RPS7,FGFR3,CTNNB1,RET,HLA-DRB1,RPL11,FGD1,ROR2,FOXH1,SKI,CDON,BMP7,FGD4,ESR1,PIK3R1,KMT2A |
| **GO:0003151** | outflow tract morphogenesis | 4 | 71 | 0.0030 | JAG1,NOTCH1,RYR1,FOXH1 |
| **GO:0030279** | negative regulation of ossification | 4 | 71 | 0.0030 | TGFB1,NOTCH1,PTCH1,SKI |
| **GO:0051704** | multi-organism process | 24 | 2514 | 0.0030 | TNFRSF1A,MMP2,DHODH,TGFB1,SMARCB1,EP300,EGFR,NOTCH1,ATM,CD96,TDGF1,WDR35,CTNNB1,HLA-DRB1,NUP188,STXBP1,BRCA2,CTNND1,HLA-B,ESR1,COLEC11,FLNB,PIK3R1,ACTG1 |
| **GO:0060249** | anatomical structure homeostasis | 7 | 285 | 0.0030 | NOTCH1,ATM,CTNNB1,LARGE,ABCA4,BRCA2,KMT2A |
| **GO:0001666** | response to hypoxia | 7 | 288 | 0.0031 | MMP2,TGFB1,EP300,NOTCH1,ATM,RYR1,BMP7 |
| **GO:0003208** | cardiac ventricle morphogenesis | 4 | 72 | 0.0031 | TGFB1,JAG1,NOTCH1,FOXH1 |
| **GO:0031323** | regulation of cellular metabolic process | 44 | 6082 | 0.0031 | TNFRSF1A,MYH9,TGFB1,FKTN,TTC21B,JAG1,APC,EDAR,POMT2,SMARCB1,EP300,EGFR,NOTCH1,ATM,TDGF1,TSC1,WDR35,FBN1,PTCH1,RPS7,FGFR3,CTNNB1,RET,RPS26,HLA-DRB1,AHI1,POMT1,RPL11,ASXL1,ROR2,FOXH1,SKI,BRCA2,CDON,ACTN1,BMP7,PAX1,CTNND1,PAX6,ESR1,BRCA1,PIK3R1,KMT2A,CEP290 |
| **GO:0032870** | cellular response to hormone stimulus | 10 | 585 | 0.0031 | TGFB1,APC,EGFR,NOTCH1,FBN1,CTNNB1,BMP7,ESR1,BRCA1,PIK3R1 |
| **GO:0045879** | negative regulation of smoothened signaling pathway | 3 | 29 | 0.0031 | IFT122,PTCH1,PTCH2 |
| **GO:0048514** | blood vessel morphogenesis | 8 | 381 | 0.0031 | MYH9,MMP2,TGFB1,JAG1,NOTCH1,TDGF1,CTNNB1,FOXH1 |
| **GO:0060603** | mammary gland duct morphogenesis | 3 | 29 | 0.0031 | TGFB1,PTCH1,ESR1 |
| **GO:0060751** | branch elongation involved in mammary gland duct branching | 2 | 5 | 0.0031 | TGFB1,ESR1 |
| **GO:0071681** | cellular response to indole-3-methanol | 2 | 5 | 0.0031 | CTNNB1,BRCA1 |
| **GO:0044255** | cellular lipid metabolic process | 13 | 946 | 0.0032 | TNFRSF1A,PHYH,EGFR,ATM,FGFR3,LARGE,ABCA4,INPP5E,PIGO,ESR1,PIGG,BRCA1,PIK3R1 |
| **GO:0071396** | cellular response to lipid | 9 | 486 | 0.0033 | TGFB1,EGFR,WDR35,PTCH1,CTNNB1,RET,BMP7,ESR1,BRCA1 |
| **GO:0034329** | cell junction assembly | 5 | 135 | 0.0034 | KRT14,APC,CTNNB1,ACTN1,ACTG1 |
| **GO:0009612** | response to mechanical stimulus | 6 | 210 | 0.0035 | TNFRSF1A,TGFB1,EGFR,PTCH1,ACTA1,ACTG1 |
| **GO:0007492** | endoderm development | 4 | 76 | 0.0036 | MMP2,TGFB1,NOTCH1,CTNNB1 |
| **GO:0033143** | regulation of intracellular steroid hormone receptor signaling pathway | 4 | 76 | 0.0036 | EP300,FOXH1,ESR1,BRCA1 |
| **GO:0090288** | negative regulation of cellular response to growth factor stimulus | 5 | 137 | 0.0036 | TGFB1,NOTCH1,FUZ,FBN1,SKI |
| **GO:0010605** | negative regulation of macromolecule metabolic process | 24 | 2558 | 0.0037 | TGFB1,FKTN,APC,SMARCB1,EP300,EGFR,NOTCH1,ATM,TSC1,WDR35,PTCH1,RPS7,CTNNB1,RPS26,RPS27,RPL11,FOXH1,SKI,BRCA2,BMP7,PAX6,ESR1,BRCA1,KMT2A |
| **GO:0050790** | regulation of catalytic activity | 22 | 2249 | 0.0038 | TGFB1,APC,ARHGAP31,EGFR,NOTCH1,TDGF1,TSC1,WDR35,FBN1,RPS7,FGFR3,CTNNB1,RET,HLA-DRB1,RPL11,FGD1,ROR2,SKI,BMP7,FGD4,ESR1,PIK3R1 |
| **GO:0007155** | cell adhesion | 12 | 843 | 0.0039 | APC,EGFR,CD96,TSC1,FBN1,CTNNB1,RET,STXBP1,CDON,ACTN1,BMP7,CTNND1 |
| **GO:0021522** | spinal cord motor neuron differentiation | 3 | 32 | 0.0039 | PTCH1,DYNC2H1,PAX6 |
| **GO:0071560** | cellular response to transforming growth factor beta stimulus | 5 | 140 | 0.0039 | TGFB1,COL1A2,FBN1,FOXH1,SKI |
| **GO:1905332** | positive regulation of morphogenesis of an epithelium | 3 | 32 | 0.0039 | TGFB1,CTNNB1,AHI1 |
| **GO:0060284** | regulation of cell development | 12 | 846 | 0.0040 | TNFRSF1A,TGFB1,JAG1,NOTCH1,TSC1,FBN1,CTNNB1,RET,SKI,CDON,BMP7,PAX6 |
| **GO:1900020** | positive regulation of protein kinase C activity | 2 | 6 | 0.0040 | EGFR,ROR2 |
| **GO:0043549** | regulation of kinase activity | 12 | 849 | 0.0041 | TGFB1,APC,EGFR,TDGF1,TSC1,FBN1,FGFR3,RET,HLA-DRB1,ROR2,BMP7,PIK3R1 |
| **GO:0048048** | embryonic eye morphogenesis | 3 | 33 | 0.0041 | FBN1,BMP7,PAX6 |
| **GO:0048667** | cell morphogenesis involved in neuron differentiation | 8 | 400 | 0.0041 | WDPCP,NOTCH1,PTCH1,RET,STXBP1,BMP7,PAX6,PIK3R1 |
| **GO:0060828** | regulation of canonical Wnt signaling pathway | 6 | 218 | 0.0041 | APC,EGFR,NOTCH1,FUZ,ROR2,CTNND1 |
| **GO:0090322** | regulation of superoxide metabolic process | 3 | 33 | 0.0041 | TGFB1,EGFR,BMP7 |
| **GO:0010631** | epithelial cell migration | 4 | 80 | 0.0042 | MYH9,WDPCP,TDGF1,NUP188 |
| **GO:0010629** | negative regulation of gene expression | 18 | 1670 | 0.0043 | TGFB1,EP300,NOTCH1,ATM,TSC1,WDR35,PTCH1,RPS7,CTNNB1,RPS26,RPS27,RPL11,FOXH1,SKI,BMP7,PAX6,ESR1,BRCA1 |
| **GO:0051090** | regulation of DNA-binding transcription factor activity | 8 | 403 | 0.0043 | TGFB1,SMARCB1,EP300,PTCH1,CTNNB1,FOXH1,BMP7,ESR1 |
| **GO:0044267** | cellular protein metabolic process | 30 | 3603 | 0.0044 | MMP2,TGFB1,FKTN,APC,POMT2,EP300,EGFR,ATM,TDGF1,FBN1,RPS7,FGFR3,LARGE,RET,RPS26,RPS27,POMT1,RPL11,ASXL1,ROR2,KDM6A,PIGO,BRCA2,PAX6,ESR1,PIGG,BRCA1,PIK3R1,KMT2A,MYH3 |
| **GO:0001667** | ameboidal-type cell migration | 5 | 146 | 0.0045 | MYH9,WDPCP,TDGF1,RET,NUP188 |
| **GO:0030330** | DNA damage response, signal transduction by p53 class mediator | 4 | 82 | 0.0045 | EP300,ATM,BRCA2,BRCA1 |
| **GO:0046907** | intracellular transport | 16 | 1390 | 0.0045 | TGFB1,TTC21B,PHYH,ANK1,IFT122,TSC1,WDR35,RPS7,CTNNB1,RPS26,RPS27,NUP188,RPL11,DYNC2H1,WDR60,PIK3R1 |
| **GO:0060993** | kidney morphogenesis | 4 | 82 | 0.0045 | FRAS1,PTCH1,CTNNB1,AHI1 |
| **GO:0045682** | regulation of epidermis development | 4 | 83 | 0.0047 | NOTCH1,PTCH1,CTNNB1,PTCH2 |
| **GO:0045778** | positive regulation of ossification | 4 | 83 | 0.0047 | TGFB1,JAG1,CTNNB1,BMP7 |
| **GO:0061384** | heart trabecula morphogenesis | 3 | 35 | 0.0047 | NOTCH1,FOXH1,BMP7 |
| **GO:0110111** | negative regulation of animal organ morphogenesis | 3 | 35 | 0.0047 | NOTCH1,CTNNB1,BMP7 |
| **GO:0010742** | macrophage derived foam cell differentiation | 2 | 7 | 0.0048 | TGFB1,EP300 |
| **GO:0021798** | forebrain dorsal/ventral pattern formation | 2 | 7 | 0.0048 | TTC21B,PAX6 |
| **GO:0045786** | negative regulation of cell cycle | 9 | 517 | 0.0048 | TGFB1,APC,EP300,EGFR,NOTCH1,ATM,CTNNB1,BMP7,BRCA1 |
| **GO:0060687** | regulation of branching involved in prostate gland morphogenesis | 2 | 7 | 0.0048 | BMP7,ESR1 |
| **GO:0070200** | establishment of protein localization to telomere | 2 | 7 | 0.0048 | ATM,BRCA2 |
| **GO:0072203** | cell proliferation involved in metanephros development | 2 | 7 | 0.0048 | PTCH1,BMP7 |
| **GO:0090240** | positive regulation of histone H4 acetylation | 2 | 7 | 0.0048 | SMARCB1,BRCA1 |
| **GO:1902339** | positive regulation of apoptotic process involved in morphogenesis | 2 | 7 | 0.0048 | TNFRSF1A,NOTCH1 |
| **GO:2000617** | positive regulation of histone H3-K9 acetylation | 2 | 7 | 0.0048 | SMARCB1,BRCA1 |
| **GO:0014070** | response to organic cyclic compound | 12 | 873 | 0.0049 | DHODH,TGFB1,EGFR,NOTCH1,PTCH1,CTNNB1,RYR1,ACTA1,STXBP1,BMP7,ESR1,BRCA1 |
| **GO:0033619** | membrane protein proteolysis | 3 | 36 | 0.0049 | MYH9,TGFB1,RET |
| **GO:0035137** | hindlimb morphogenesis | 3 | 36 | 0.0049 | NOTCH1,PTCH1,CTNNB1 |
| **GO:0048524** | positive regulation of viral process | 4 | 85 | 0.0049 | SMARCB1,EP300,NOTCH1,HLA-DRB1 |
| **GO:0048599** | oocyte development | 3 | 36 | 0.0049 | ATM,CTNNB1,BRCA2 |
| **GO:0050776** | regulation of immune response | 12 | 873 | 0.0049 | MMP2,TGFB1,EP300,CD96,COL1A2,HLA-DRB1,STXBP1,HLA-B,ESR1,COLEC11,PIK3R1,ACTG1 |
| **GO:0071158** | positive regulation of cell cycle arrest | 4 | 85 | 0.0049 | TGFB1,EP300,ATM,BRCA1 |
| **GO:0006357** | regulation of transcription by RNA polymerase II | 24 | 2633 | 0.0050 | TNFRSF1A,TGFB1,TTC21B,JAG1,SMARCB1,EP300,EGFR,NOTCH1,FBN1,PTCH1,CTNNB1,AHI1,ASXL1,ROR2,FOXH1,SKI,CDON,BMP7,PAX1,PAX6,ESR1,BRCA1,PIK3R1,KMT2A |
| **GO:0030036** | actin cytoskeleton organization | 8 | 418 | 0.0050 | MYH9,ACTA1,FGD1,ACTN1,FGD4,FLNB,ACTG1,MYH3 |
| **GO:0060349** | bone morphogenesis | 4 | 86 | 0.0050 | TGFB1,FGFR3,SKI,PAX1 |
| **GO:0001738** | morphogenesis of a polarized epithelium | 3 | 37 | 0.0052 | FUZ,AHI1,RPGRIP1L |
| **GO:0007093** | mitotic cell cycle checkpoint | 5 | 153 | 0.0052 | TGFB1,APC,EP300,ATM,BRCA1 |
| **GO:0045839** | negative regulation of mitotic nuclear division | 3 | 37 | 0.0052 | APC,ATM,BMP7 |
| **GO:0001101** | response to acid chemical | 7 | 323 | 0.0053 | MMP2,DHODH,EGFR,COL1A2,PTCH1,RET,ASXL1 |
| **GO:0044093** | positive regulation of molecular function | 18 | 1713 | 0.0053 | TGFB1,SMARCB1,EP300,ARHGAP31,EGFR,TDGF1,TSC1,WDR35,FBN1,FGFR3,CTNNB1,RET,HLA-DRB1,RPL11,ROR2,SKI,ESR1,KMT2A |
| **GO:0048738** | cardiac muscle tissue development | 5 | 154 | 0.0053 | TGFB1,NOTCH1,TSC1,FOXH1,BMP7 |
| **GO:0007179** | transforming growth factor beta receptor signaling pathway | 4 | 88 | 0.0054 | TGFB1,COL1A2,FOXH1,SKI |
| **GO:0003156** | regulation of animal organ formation | 3 | 38 | 0.0055 | NOTCH1,CTNNB1,BMP7 |
| **GO:0051701** | interaction with host | 5 | 156 | 0.0056 | TGFB1,EGFR,CTNNB1,HLA-DRB1,CTNND1 |
| **GO:0001775** | cell activation | 13 | 1024 | 0.0057 | MYH9,TGFB1,EP300,EGFR,ATM,COL1A2,TSC1,CTNNB1,STXBP1,PAX1,HLA-B,PIK3R1,CEP290 |
| **GO:0021537** | telencephalon development | 6 | 237 | 0.0057 | EGFR,TSC1,CTNNB1,SKI,CDON,PAX6 |
| **GO:0035635** | entry of bacterium into host cell | 2 | 8 | 0.0057 | CTNNB1,CTNND1 |
| **GO:0048793** | pronephros development | 2 | 8 | 0.0057 | AHI1,CEP290 |
| **GO:1903799** | negative regulation of production of miRNAs involved in gene silencing by miRNA | 2 | 8 | 0.0057 | TGFB1,ESR1 |
| **GO:1903800** | positive regulation of production of miRNAs involved in gene silencing by miRNA | 2 | 8 | 0.0057 | TGFB1,EGFR |
| **GO:0014014** | negative regulation of gliogenesis | 3 | 39 | 0.0058 | NOTCH1,CTNNB1,SKI |
| **GO:0030857** | negative regulation of epithelial cell differentiation | 3 | 39 | 0.0058 | JAG1,NOTCH1,CTNNB1 |
| **GO:0031057** | negative regulation of histone modification | 3 | 39 | 0.0058 | SMARCB1,SKI,BRCA1 |
| **GO:1901652** | response to peptide | 8 | 431 | 0.0058 | TGFB1,JAG1,APC,NOTCH1,TSC1,FBN1,BMP7,PIK3R1 |
| **GO:1903506** | regulation of nucleic acid-templated transcription | 30 | 3683 | 0.0058 | TNFRSF1A,TGFB1,TTC21B,JAG1,SMARCB1,EP300,EGFR,NOTCH1,FBN1,PTCH1,CTNNB1,RET,HLA-DRB1,AHI1,ASXL1,ROR2,FOXH1,SKI,BRCA2,CDON,ACTN1,BMP7,PAX1,CTNND1,PAX6,ESR1,BRCA1,PIK3R1,KMT2A,CEP290 |
| **GO:0002065** | columnar/cuboidal epithelial cell differentiation | 4 | 91 | 0.0059 | JAG1,WDPCP,NOTCH1,PAX6 |
| **GO:0021782** | glial cell development | 4 | 91 | 0.0059 | TGFB1,EGFR,ROR2,SKI |
| **GO:0006614** | SRP-dependent cotranslational protein targeting to membrane | 4 | 92 | 0.0061 | RPS7,RPS26,RPS27,RPL11 |
| **GO:0030509** | BMP signaling pathway | 4 | 92 | 0.0061 | TGFB1,ROR2,SKI,BMP7 |
| **GO:0033146** | regulation of intracellular estrogen receptor signaling pathway | 3 | 40 | 0.0061 | FOXH1,ESR1,BRCA1 |
| **GO:0048704** | embryonic skeletal system morphogenesis | 4 | 92 | 0.0061 | FUZ,CTNNB1,BMP7,WDR60 |
| **GO:2000826** | regulation of heart morphogenesis | 3 | 40 | 0.0061 | NOTCH1,CTNNB1,BMP7 |
| **GO:0030324** | lung development | 5 | 162 | 0.0063 | EP300,EGFR,NOTCH1,CTNNB1,ASXL1 |
| **GO:0032879** | regulation of localization | 23 | 2524 | 0.0063 | TNFRSF1A,TGFB1,JAG1,APC,WDPCP,EGFR,NOTCH1,TDGF1,FUZ,PTCH1,CTNNB1,RET,RYR1,HLA-DRB1,AHI1,STXBP1,ROR2,ACTN1,BMP7,PAX6,PIK3R1,KMT2A,CEP290 |
| **GO:0097191** | extrinsic apoptotic signaling pathway | 4 | 93 | 0.0063 | TNFRSF1A,TGFB1,FGFR3,PIK3R1 |
| **GO:0012501** | programmed cell death | 13 | 1042 | 0.0065 | TNFRSF1A,KRT14,TGFB1,EDAR,EP300,NOTCH1,ATM,FGFR3,BRCA2,COLEC11,BRCA1,PIK3R1,KMT2A |
| **GO:0051252** | regulation of RNA metabolic process | 31 | 3890 | 0.0065 | TNFRSF1A,TGFB1,TTC21B,JAG1,SMARCB1,EP300,EGFR,NOTCH1,FBN1,PTCH1,CTNNB1,RET,RPS26,HLA-DRB1,AHI1,ASXL1,ROR2,FOXH1,SKI,BRCA2,CDON,ACTN1,BMP7,PAX1,CTNND1,PAX6,ESR1,BRCA1,PIK3R1,KMT2A,CEP290 |
| **GO:0003344** | pericardium morphogenesis | 2 | 9 | 0.0066 | NOTCH1,BMP7 |
| **GO:0006915** | apoptotic process | 12 | 915 | 0.0066 | TNFRSF1A,TGFB1,EDAR,EP300,NOTCH1,ATM,FGFR3,BRCA2,COLEC11,BRCA1,PIK3R1,KMT2A |
| **GO:0034504** | protein localization to nucleus | 5 | 164 | 0.0066 | TGFB1,NUP188,RPL11,BMP7,PIK3R1 |
| **GO:0061314** | Notch signaling involved in heart development | 2 | 9 | 0.0066 | JAG1,NOTCH1 |
| **GO:1901201** | regulation of extracellular matrix assembly | 2 | 9 | 0.0066 | TGFB1,NOTCH1 |
| **GO:1990314** | cellular response to insulin-like growth factor stimulus | 2 | 9 | 0.0066 | TGFB1,FBN1 |
| **GO:0003197** | endocardial cushion development | 3 | 42 | 0.0068 | JAG1,NOTCH1,BMP7 |
| **GO:0090311** | regulation of protein deacetylation | 3 | 42 | 0.0068 | TGFB1,EP300,SKI |
| **GO:0043122** | regulation of I-kappaB kinase/NF-kappaB signaling | 5 | 167 | 0.0070 | TNFRSF1A,EDAR,CTNNB1,HLA-DRB1,ESR1 |
| **GO:0006629** | lipid metabolic process | 14 | 1192 | 0.0071 | TNFRSF1A,PHYH,EGFR,ATM,FGFR3,LBR,LARGE,ABCA4,INPP5E,PIGO,ESR1,PIGG,BRCA1,PIK3R1 |
| **GO:0007409** | axonogenesis | 7 | 346 | 0.0071 | NOTCH1,PTCH1,RET,STXBP1,BMP7,PAX6,PIK3R1 |
| **GO:0031032** | actomyosin structure organization | 4 | 97 | 0.0071 | MYH9,ACTA1,ACTG1,MYH3 |
| **GO:0045216** | cell-cell junction organization | 5 | 168 | 0.0071 | TGFB1,APC,CTNNB1,ACTN1,CTNND1 |
| **GO:0061005** | cell differentiation involved in kidney development | 3 | 43 | 0.0071 | JAG1,NOTCH1,PTCH1 |
| **GO:0042593** | glucose homeostasis | 5 | 169 | 0.0072 | WDR35,FBN1,PTCH1,PAX6,PIK3R1 |
| **GO:0090287** | regulation of cellular response to growth factor stimulus | 6 | 254 | 0.0074 | TGFB1,NOTCH1,FUZ,FBN1,CTNNB1,SKI |
| **GO:0001840** | neural plate development | 2 | 10 | 0.0075 | PTCH1,CTNNB1 |
| **GO:0003266** | regulation of secondary heart field cardioblast proliferation | 2 | 10 | 0.0075 | NOTCH1,CTNNB1 |
| **GO:0006508** | proteolysis | 14 | 1203 | 0.0075 | MYH9,MMP2,TGFB1,APC,POMT2,PTCH1,RET,POMT1,ASXL1,ADAMTS20,DYNC2H1,ESR1,COLEC11,BRCA1 |
| **GO:0007440** | foregut morphogenesis | 2 | 10 | 0.0075 | NOTCH1,CTNNB1 |
| **GO:0045668** | negative regulation of osteoblast differentiation | 3 | 44 | 0.0075 | NOTCH1,PTCH1,SKI |
| **GO:0051573** | negative regulation of histone H3-K9 methylation | 2 | 10 | 0.0075 | SMARCB1,BRCA1 |
| **GO:0060768** | regulation of epithelial cell proliferation involved in prostate gland development | 2 | 10 | 0.0075 | NOTCH1,CTNNB1 |
| **GO:1901990** | regulation of mitotic cell cycle phase transition | 7 | 351 | 0.0075 | TGFB1,APC,EP300,EGFR,ATM,BRCA1,CEP290 |
| **GO:1904667** | negative regulation of ubiquitin protein ligase activity | 2 | 10 | 0.0075 | RPS7,RPL11 |
| **GO:1905207** | regulation of cardiocyte differentiation | 3 | 44 | 0.0075 | TGFB1,EGFR,BMP7 |
| **GO:0010564** | regulation of cell cycle process | 10 | 684 | 0.0076 | TGFB1,APC,EP300,EGFR,ATM,CTNNB1,BRCA2,BMP7,BRCA1,CEP290 |
| **GO:0044409** | entry into host | 4 | 100 | 0.0076 | EGFR,CTNNB1,HLA-DRB1,CTNND1 |
| **GO:0051099** | positive regulation of binding | 5 | 172 | 0.0076 | TGFB1,EP300,CTNNB1,RPL11,SKI |
| **GO:0042461** | photoreceptor cell development | 3 | 45 | 0.0078 | AHI1,PAX6,CEP290 |
| **GO:0016032** | viral process | 9 | 571 | 0.0079 | TNFRSF1A,TGFB1,SMARCB1,EP300,EGFR,HLA-DRB1,NUP188,HLA-B,PIK3R1 |
| **GO:0019219** | regulation of nucleobase-containing compound metabolic process | 32 | 4133 | 0.0079 | TNFRSF1A,TGFB1,TTC21B,JAG1,SMARCB1,EP300,EGFR,NOTCH1,ATM,FBN1,PTCH1,CTNNB1,RET,RPS26,HLA-DRB1,AHI1,ASXL1,ROR2,FOXH1,SKI,BRCA2,CDON,ACTN1,BMP7,PAX1,CTNND1,PAX6,ESR1,BRCA1,PIK3R1,KMT2A,CEP290 |
| **GO:0051248** | negative regulation of protein metabolic process | 13 | 1075 | 0.0080 | TGFB1,FKTN,APC,SMARCB1,EGFR,TSC1,RPS7,CTNNB1,RPL11,SKI,BMP7,PAX6,BRCA1 |
| **GO:0003179** | heart valve morphogenesis | 3 | 46 | 0.0082 | TGFB1,JAG1,NOTCH1 |
| **GO:0008610** | lipid biosynthetic process | 9 | 575 | 0.0082 | ATM,FGFR3,LBR,LARGE,INPP5E,PIGO,PIGG,BRCA1,PIK3R1 |
| **GO:0043200** | response to amino acid | 4 | 103 | 0.0082 | MMP2,DHODH,EGFR,COL1A2 |
| **GO:0043551** | regulation of phosphatidylinositol 3-kinase activity | 3 | 46 | 0.0082 | TGFB1,FGFR3,PIK3R1 |
| **GO:0045599** | negative regulation of fat cell differentiation | 3 | 46 | 0.0082 | TGFB1,JAG1,ASXL1 |
| **GO:0051960** | regulation of nervous system development | 11 | 817 | 0.0082 | TGFB1,JAG1,NOTCH1,IFT122,TSC1,CTNNB1,RET,SKI,CDON,BMP7,PAX6 |
| **GO:0002011** | morphogenesis of an epithelial sheet | 3 | 47 | 0.0085 | JAG1,NOTCH1,BMP7 |
| **GO:0060525** | prostate glandular acinus development | 2 | 11 | 0.0085 | NOTCH1,ESR1 |
| **GO:0072132** | mesenchyme morphogenesis | 3 | 47 | 0.0085 | NOTCH1,ACTA1,BMP7 |
| **GO:0001952** | regulation of cell-matrix adhesion | 4 | 105 | 0.0087 | JAG1,WDPCP,TSC1,PIK3R1 |
| **GO:1901564** | organonitrogen compound metabolic process | 38 | 5281 | 0.0089 | TCN2,MYH9,MMP2,DHODH,TGFB1,FKTN,DMGDH,APC,POMT2,EP300,EGFR,ATM,TDGF1,FBN1,PTCH1,RPS7,FGFR3,LARGE,RET,RPS26,RPS27,POMT1,RPL11,ASXL1,ROR2,KDM6A,PIGO,BRCA2,ADAMTS20,DYNC2H1,PAX6,ESR1,COLEC11,PIGG,BRCA1,PIK3R1,KMT2A,MYH3 |
| **GO:0045747** | positive regulation of Notch signaling pathway | 3 | 48 | 0.0090 | JAG1,EP300,NOTCH1 |
| **GO:0048806** | genitalia development | 3 | 48 | 0.0090 | CTNNB1,ROR2,ESR1 |
| **GO:0055010** | ventricular cardiac muscle tissue morphogenesis | 3 | 48 | 0.0090 | TGFB1,NOTCH1,FOXH1 |
| **GO:0030514** | negative regulation of BMP signaling pathway | 3 | 49 | 0.0094 | NOTCH1,FBN1,SKI |
| **GO:0031324** | negative regulation of cellular metabolic process | 22 | 2463 | 0.0094 | TGFB1,FKTN,APC,SMARCB1,EP300,NOTCH1,ATM,TSC1,WDR35,PTCH1,RPS7,CTNNB1,RPS26,RPL11,FOXH1,SKI,BRCA2,BMP7,PAX6,ESR1,BRCA1,KMT2A |
| **GO:0035556** | intracellular signal transduction | 16 | 1528 | 0.0094 | TNFRSF1A,TGFB1,EP300,ARHGAP31,EGFR,ATM,IFT122,COL1A2,FBN1,FGFR3,RET,RYR1,ROR2,BRCA2,BRCA1,PIK3R1 |
| **GO:0043967** | histone H4 acetylation | 3 | 49 | 0.0094 | EP300,BRCA2,KMT2A |
| **GO:0050778** | positive regulation of immune response | 9 | 589 | 0.0094 | MMP2,TGFB1,EP300,HLA-DRB1,STXBP1,HLA-B,COLEC11,PIK3R1,ACTG1 |
| **GO:0006355** | regulation of transcription, DNA-templated | 29 | 3661 | 0.0096 | TNFRSF1A,TGFB1,TTC21B,JAG1,SMARCB1,EP300,EGFR,NOTCH1,FBN1,PTCH1,CTNNB1,RET,HLA-DRB1,AHI1,ASXL1,ROR2,FOXH1,SKI,BRCA2,CDON,BMP7,PAX1,CTNND1,PAX6,ESR1,BRCA1,PIK3R1,KMT2A,CEP290 |
| **GO:0007494** | midgut development | 2 | 12 | 0.0096 | EGFR,RET |
| **GO:0048505** | regulation of timing of cell differentiation | 2 | 12 | 0.0096 | NOTCH1,PAX6 |
| **GO:0048715** | negative regulation of oligodendrocyte differentiation | 2 | 12 | 0.0096 | NOTCH1,CTNNB1 |
| **GO:0060272** | embryonic skeletal joint morphogenesis | 2 | 12 | 0.0096 | CTNNB1,BMP7 |
| **GO:0060742** | epithelial cell differentiation involved in prostate gland development | 2 | 12 | 0.0096 | NOTCH1,CTNNB1 |
| **GO:0072017** | distal tubule development | 2 | 12 | 0.0096 | JAG1,NOTCH1 |
| **GO:0072425** | signal transduction involved in G2 DNA damage checkpoint | 2 | 12 | 0.0096 | ATM,BRCA1 |
| **GO:1990403** | embryonic brain development | 2 | 12 | 0.0096 | CTNNB1,CC2D2A |
| **GO:0036092** | phosphatidylinositol-3-phosphate biosynthetic process | 3 | 51 | 0.0102 | ATM,FGFR3,PIK3R1 |
| **GO:0090101** | negative regulation of transmembrane receptor protein serine/threonine kinase signaling pathway | 4 | 111 | 0.0102 | TGFB1,NOTCH1,FBN1,SKI |
| **GO:0010810** | regulation of cell-substrate adhesion | 5 | 189 | 0.0105 | JAG1,WDPCP,NOTCH1,TSC1,PIK3R1 |
| **GO:0060560** | developmental growth involved in morphogenesis | 4 | 112 | 0.0105 | TGFB1,NOTCH1,CTNNB1,ESR1 |
| **GO:0007173** | epidermal growth factor receptor signaling pathway | 3 | 52 | 0.0107 | TGFB1,EGFR,PIK3R1 |
| **GO:0021781** | glial cell fate commitment | 2 | 13 | 0.0107 | CTNNB1,PAX6 |
| **GO:0042476** | odontogenesis | 4 | 113 | 0.0107 | EDAR,COL1A2,CTNNB1,BMP7 |
| **GO:0046323** | glucose import | 2 | 13 | 0.0107 | TSC1,SLC2A10 |
| **GO:0048641** | regulation of skeletal muscle tissue development | 3 | 52 | 0.0107 | TGFB1,CTNNB1,CDON |
| **GO:0051052** | regulation of DNA metabolic process | 7 | 381 | 0.0107 | TGFB1,EGFR,ATM,CTNNB1,BRCA2,BRCA1,KMT2A |
| **GO:0070986** | left/right axis specification | 2 | 13 | 0.0107 | NOTCH1,AHI1 |
| **GO:0071712** | ER-associated misfolded protein catabolic process | 2 | 13 | 0.0107 | POMT2,POMT1 |
| **GO:0090192** | regulation of glomerulus development | 2 | 13 | 0.0107 | RET,BMP7 |
| **GO:0000956** | nuclear-transcribed mRNA catabolic process | 5 | 191 | 0.0108 | ATM,RPS7,RPS26,RPS27,RPL11 |
| **GO:0006464** | cellular protein modification process | 25 | 2999 | 0.0108 | TGFB1,FKTN,APC,POMT2,EP300,EGFR,ATM,TDGF1,FBN1,FGFR3,LARGE,RET,POMT1,ASXL1,ROR2,KDM6A,PIGO,BRCA2,PAX6,ESR1,PIGG,BRCA1,PIK3R1,KMT2A,MYH3 |
| **GO:0050767** | regulation of neurogenesis | 10 | 730 | 0.0109 | TGFB1,JAG1,NOTCH1,TSC1,CTNNB1,RET,SKI,CDON,BMP7,PAX6 |
| **GO:0071214** | cellular response to abiotic stimulus | 6 | 282 | 0.0109 | TNFRSF1A,TGFB1,EP300,EGFR,ATM,PIK3R1 |
| **GO:0043388** | positive regulation of DNA binding | 3 | 53 | 0.0110 | TGFB1,CTNNB1,SKI |
| **GO:0051726** | regulation of cell cycle | 13 | 1129 | 0.0111 | TGFB1,APC,EP300,EGFR,NOTCH1,ATM,TSC1,PTCH1,CTNNB1,BRCA2,BMP7,BRCA1,CEP290 |
| **GO:0001704** | formation of primary germ layer | 4 | 115 | 0.0112 | MMP2,CTNNB1,KDM6A,BMP7 |
| **GO:0007219** | Notch signaling pathway | 4 | 116 | 0.0114 | TGFB1,JAG1,EP300,NOTCH1 |
| **GO:0045661** | regulation of myoblast differentiation | 3 | 54 | 0.0114 | TGFB1,NOTCH1,CDON |
| **GO:0048146** | positive regulation of fibroblast proliferation | 3 | 54 | 0.0114 | TGFB1,EGFR,ESR1 |
| **GO:0061448** | connective tissue development | 5 | 194 | 0.0114 | TGFB1,NOTCH1,FGFR3,ROR2,BMP7 |
| **GO:0044089** | positive regulation of cellular component biogenesis | 8 | 498 | 0.0116 | TGFB1,APC,ATM,TSC1,FUZ,CTNNB1,BMP7,ESR1 |
| **GO:0045934** | negative regulation of nucleobase-containing compound metabolic process | 15 | 1424 | 0.0116 | TGFB1,EP300,NOTCH1,ATM,PTCH1,CTNNB1,RPS26,FOXH1,SKI,BRCA2,BMP7,PAX6,ESR1,BRCA1,KMT2A |
| **GO:0002716** | negative regulation of natural killer cell mediated immunity | 2 | 14 | 0.0117 | CD96,HLA-B |
| **GO:0010812** | negative regulation of cell-substrate adhesion | 3 | 55 | 0.0117 | JAG1,NOTCH1,PIK3R1 |
| **GO:0033147** | negative regulation of intracellular estrogen receptor signaling pathway | 2 | 14 | 0.0117 | FOXH1,BRCA1 |
| **GO:0035112** | genitalia morphogenesis | 2 | 14 | 0.0117 | CTNNB1,ROR2 |
| **GO:0060253** | negative regulation of glial cell proliferation | 2 | 14 | 0.0117 | NOTCH1,SKI |
| **GO:0070374** | positive regulation of ERK1 and ERK2 cascade | 5 | 196 | 0.0117 | TGFB1,EGFR,NOTCH1,FGFR3,HLA-DRB1 |
| **GO:0070977** | bone maturation | 2 | 14 | 0.0117 | FGFR3,RYR1 |
| **GO:0071599** | otic vesicle development | 2 | 14 | 0.0117 | AHI1,CEP290 |
| **GO:0000184** | nuclear-transcribed mRNA catabolic process, nonsense-mediated decay | 4 | 118 | 0.0118 | RPS7,RPS26,RPS27,RPL11 |
| **GO:0071356** | cellular response to tumor necrosis factor | 5 | 197 | 0.0118 | TNFRSF1A,EDAR,TDGF1,WDR35,BRCA1 |
| **GO:0009416** | response to light stimulus | 6 | 290 | 0.0120 | EP300,EGFR,ABCA4,BRCA2,PIK3R1,KMT2A |
| **GO:0007160** | cell-matrix adhesion | 4 | 119 | 0.0121 | CD96,TSC1,CTNNB1,ACTN1 |
| **GO:0010518** | positive regulation of phospholipase activity | 3 | 56 | 0.0122 | EGFR,FGFR3,ESR1 |
| **GO:0030155** | regulation of cell adhesion | 9 | 623 | 0.0123 | TGFB1,JAG1,WDPCP,NOTCH1,TSC1,RET,HLA-DRB1,BMP7,PIK3R1 |
| **GO:0071407** | cellular response to organic cyclic compound | 8 | 505 | 0.0123 | TGFB1,EGFR,PTCH1,CTNNB1,RYR1,BMP7,ESR1,BRCA1 |
| **GO:0032269** | negative regulation of cellular protein metabolic process | 12 | 1014 | 0.0126 | TGFB1,FKTN,APC,SMARCB1,TSC1,RPS7,CTNNB1,RPL11,SKI,BMP7,PAX6,BRCA1 |
| **GO:0002181** | cytoplasmic translation | 3 | 57 | 0.0127 | RPS7,RPS26,RPL11 |
| **GO:0002684** | positive regulation of immune system process | 11 | 882 | 0.0128 | MMP2,TGFB1,JAG1,EP300,HLA-DRB1,STXBP1,ROR2,HLA-B,COLEC11,PIK3R1,ACTG1 |
| **GO:0016045** | detection of bacterium | 2 | 15 | 0.0129 | HLA-DRB1,HLA-B |
| **GO:0030099** | myeloid cell differentiation | 5 | 203 | 0.0130 | MYH9,TGFB1,EP300,CTNNB1,ACTN1 |
| **GO:0030198** | extracellular matrix organization | 6 | 296 | 0.0130 | MMP2,TGFB1,COL1A2,FBN1,POMT1,ADAMTS20 |
| **GO:0043123** | positive regulation of I-kappaB kinase/NF-kappaB signaling | 4 | 122 | 0.0130 | TNFRSF1A,EDAR,CTNNB1,HLA-DRB1 |
| **GO:0050900** | leukocyte migration | 6 | 296 | 0.0130 | MYH9,TGFB1,COL1A2,RET,ROR2,PIK3R1 |
| **GO:0051347** | positive regulation of transferase activity | 9 | 630 | 0.0130 | TGFB1,EGFR,TDGF1,FBN1,FGFR3,CTNNB1,RET,HLA-DRB1,ROR2 |
| **GO:0001525** | angiogenesis | 6 | 297 | 0.0131 | MYH9,MMP2,JAG1,NOTCH1,TDGF1,CTNNB1 |
| **GO:0034502** | protein localization to chromosome | 3 | 58 | 0.0131 | ATM,BRCA2,ESR1 |
| **GO:0045669** | positive regulation of osteoblast differentiation | 3 | 58 | 0.0131 | JAG1,CTNNB1,BMP7 |
| **GO:0022402** | cell cycle process | 11 | 890 | 0.0135 | MYH9,TGFB1,APC,EP300,NOTCH1,ATM,CTNNB1,BRCA2,PAX6,BRCA1,CEP290 |
| **GO:0045665** | negative regulation of neuron differentiation | 5 | 205 | 0.0135 | JAG1,NOTCH1,TSC1,BMP7,PAX6 |
| **GO:0001947** | heart looping | 3 | 59 | 0.0137 | NOTCH1,AHI1,FOXH1 |
| **GO:0030239** | myofibril assembly | 3 | 59 | 0.0137 | ACTA1,ACTG1,MYH3 |
| **GO:0048844** | artery morphogenesis | 3 | 59 | 0.0137 | JAG1,NOTCH1,FOXH1 |
| **GO:0003184** | pulmonary valve morphogenesis | 2 | 16 | 0.0141 | JAG1,NOTCH1 |
| **GO:0003222** | ventricular trabecula myocardium morphogenesis | 2 | 16 | 0.0141 | NOTCH1,FOXH1 |
| **GO:0009950** | dorsal/ventral axis specification | 2 | 16 | 0.0141 | CTNNB1,PAX6 |
| **GO:0010463** | mesenchymal cell proliferation | 2 | 16 | 0.0141 | CTNNB1,BMP7 |
| **GO:0031000** | response to caffeine | 2 | 16 | 0.0141 | DHODH,RYR1 |
| **GO:0032495** | response to muramyl dipeptide | 2 | 16 | 0.0141 | JAG1,NOTCH1 |
| **GO:0036270** | response to diuretic | 2 | 16 | 0.0141 | DHODH,RYR1 |
| **GO:0045603** | positive regulation of endothelial cell differentiation | 2 | 16 | 0.0141 | NOTCH1,CTNNB1 |
| **GO:0071230** | cellular response to amino acid stimulus | 3 | 60 | 0.0141 | MMP2,EGFR,COL1A2 |
| **GO:0051054** | positive regulation of DNA metabolic process | 5 | 209 | 0.0143 | TGFB1,EGFR,ATM,CTNNB1,BRCA1 |
| **GO:0014031** | mesenchymal cell development | 3 | 62 | 0.0152 | JAG1,NOTCH1,RET |
| **GO:1901796** | regulation of signal transduction by p53 class mediator | 4 | 129 | 0.0152 | EP300,ATM,RPS7,RPL11 |
| **GO:0006978** | DNA damage response, signal transduction by p53 class mediator resulting in transcription of p21 class mediator | 2 | 17 | 0.0153 | BRCA2,BRCA1 |
| **GO:0032930** | positive regulation of superoxide anion generation | 2 | 17 | 0.0153 | TGFB1,EGFR |
| **GO:0043923** | positive regulation by host of viral transcription | 2 | 17 | 0.0153 | SMARCB1,EP300 |
| **GO:0046847** | filopodium assembly | 2 | 17 | 0.0153 | FGD1,FGD4 |
| **GO:0090185** | negative regulation of kidney development | 2 | 17 | 0.0153 | CTNNB1,BMP7 |
| **GO:0035690** | cellular response to drug | 6 | 310 | 0.0154 | TGFB1,EGFR,WDR35,CTNNB1,RYR1,BRCA1 |
| **GO:0009247** | glycolipid biosynthetic process | 3 | 63 | 0.0157 | LARGE,PIGO,PIGG |
| **GO:0030217** | T cell differentiation | 4 | 131 | 0.0158 | TGFB1,TSC1,CTNNB1,PAX1 |
| **GO:0043401** | steroid hormone mediated signaling pathway | 4 | 131 | 0.0158 | CTNNB1,BMP7,ESR1,BRCA1 |
| **GO:0048863** | stem cell differentiation | 4 | 131 | 0.0158 | JAG1,NOTCH1,RET,ESR1 |
| **GO:0072078** | nephron tubule morphogenesis | 3 | 64 | 0.0162 | PTCH1,CTNNB1,AHI1 |
| **GO:0090150** | establishment of protein localization to membrane | 5 | 217 | 0.0162 | EGFR,RPS7,RPS26,RPS27,RPL11 |
| **GO:0003180** | aortic valve morphogenesis | 2 | 18 | 0.0166 | JAG1,NOTCH1 |
| **GO:0003272** | endocardial cushion formation | 2 | 18 | 0.0166 | NOTCH1,BMP7 |
| **GO:0007221** | positive regulation of transcription of Notch receptor target | 2 | 18 | 0.0166 | EP300,NOTCH1 |
| **GO:1903204** | negative regulation of oxidative stress-induced neuron death | 2 | 18 | 0.0166 | TSC1,CTNNB1 |
| **GO:1905331** | negative regulation of morphogenesis of an epithelium | 2 | 18 | 0.0166 | CTNNB1,BMP7 |
| **GO:0001570** | vasculogenesis | 3 | 65 | 0.0167 | TGFB1,NOTCH1,CTNNB1 |
| **GO:0022414** | reproductive process | 14 | 1350 | 0.0168 | MYH9,MMP2,DHODH,TGFB1,EGFR,NOTCH1,ATM,PTCH1,CTNNB1,STXBP1,ROR2,BRCA2,BMP7,ESR1 |
| **GO:0007411** | axon guidance | 5 | 220 | 0.0169 | PTCH1,RET,BMP7,PAX6,PIK3R1 |
| **GO:0009607** | response to biotic stimulus | 13 | 1206 | 0.0169 | TNFRSF1A,TGFB1,NOTCH1,CD96,TDGF1,WDR35,HLA-DRB1,STXBP1,HLA-B,COLEC11,FLNB,ACTG1,TMCO1 |
| **GO:0090092** | regulation of transmembrane receptor protein serine/threonine kinase signaling pathway | 5 | 220 | 0.0169 | TGFB1,NOTCH1,FBN1,SKI,BMP7 |
| **GO:0048709** | oligodendrocyte differentiation | 3 | 66 | 0.0172 | TGFB1,NOTCH1,PAX6 |
| **GO:0050777** | negative regulation of immune response | 4 | 136 | 0.0174 | TGFB1,CD96,HLA-DRB1,HLA-B |
| **GO:0007520** | myoblast fusion | 2 | 19 | 0.0178 | MYH9,CDON |
| **GO:0009611** | response to wounding | 8 | 547 | 0.0178 | TGFB1,EGFR,NOTCH1,COL1A2,LARGE,STXBP1,PAX6,PIK3R1 |
| **GO:0060571** | morphogenesis of an epithelial fold | 2 | 19 | 0.0178 | EGFR,BMP7 |
| **GO:2000178** | negative regulation of neural precursor cell proliferation | 2 | 19 | 0.0178 | TGFB1,PAX6 |
| **GO:0042110** | T cell activation | 5 | 225 | 0.0182 | MYH9,TGFB1,TSC1,CTNNB1,PAX1 |
| **GO:0009411** | response to UV | 4 | 139 | 0.0185 | EP300,EGFR,BRCA2,PIK3R1 |
| **GO:0031347** | regulation of defense response | 9 | 676 | 0.0186 | TNFRSF1A,MMP2,EP300,EGFR,ATM,CD96,HLA-DRB1,HLA-B,ESR1 |
| **GO:0033674** | positive regulation of kinase activity | 8 | 553 | 0.0187 | TGFB1,EGFR,TDGF1,FBN1,FGFR3,RET,HLA-DRB1,ROR2 |
| **GO:0002089** | lens morphogenesis in camera-type eye | 2 | 20 | 0.0193 | CTNNB1,SKI |
| **GO:2000737** | negative regulation of stem cell differentiation | 2 | 20 | 0.0193 | JAG1,NOTCH1 |
| **GO:0006325** | chromatin organization | 9 | 683 | 0.0197 | SMARCB1,EP300,ATM,CTNNB1,ASXL1,KDM6A,BRCA2,ESR1,KMT2A |
| **GO:0006413** | translational initiation | 4 | 142 | 0.0197 | RPS7,RPS26,RPS27,RPL11 |
| **GO:0030512** | negative regulation of transforming growth factor beta receptor signaling pathway | 3 | 70 | 0.0197 | TGFB1,FBN1,SKI |
| **GO:0008360** | regulation of cell shape | 4 | 143 | 0.0202 | MYH9,WDPCP,FGD1,FGD4 |
| **GO:0000910** | cytokinesis | 3 | 71 | 0.0203 | MYH9,APC,BRCA2 |
| **GO:0010639** | negative regulation of organelle organization | 6 | 333 | 0.0203 | APC,SMARCB1,ATM,SKI,BMP7,BRCA1 |
| **GO:0035265** | organ growth | 3 | 71 | 0.0203 | NOTCH1,FGFR3,ESR1 |
| **GO:0045446** | endothelial cell differentiation | 3 | 71 | 0.0203 | JAG1,NOTCH1,CTNNB1 |
| **GO:0050672** | negative regulation of lymphocyte proliferation | 3 | 71 | 0.0203 | TGFB1,ATM,HLA-DRB1 |
| **GO:0060548** | negative regulation of cell death | 11 | 953 | 0.0203 | EGFR,NOTCH1,TDGF1,TSC1,CTNNB1,AHI1,STXBP1,ADAMTS20,BMP7,BRCA1,PIK3R1 |
| **GO:0030902** | hindbrain development | 4 | 144 | 0.0204 | CTNNB1,AHI1,BMP7,CEP290 |
| **GO:0032101** | regulation of response to external stimulus | 11 | 955 | 0.0204 | TNFRSF1A,MMP2,TGFB1,EP300,EGFR,NOTCH1,ATM,CD96,HLA-DRB1,HLA-B,ESR1 |
| **GO:1903047** | mitotic cell cycle process | 8 | 564 | 0.0206 | TGFB1,APC,EP300,ATM,BRCA2,PAX6,BRCA1,CEP290 |
| **GO:0001736** | establishment of planar polarity | 2 | 21 | 0.0207 | FUZ,RPGRIP1L |
| **GO:0007094** | mitotic spindle assembly checkpoint | 2 | 21 | 0.0207 | APC,ATM |
| **GO:0043586** | tongue development | 2 | 21 | 0.0207 | EGFR,CTNNB1 |
| **GO:0044030** | regulation of DNA methylation | 2 | 21 | 0.0207 | BRCA1,KMT2A |
| **GO:1901798** | positive regulation of signal transduction by p53 class mediator | 2 | 21 | 0.0207 | ATM,RPS7 |
| **GO:1904886** | beta-catenin destruction complex disassembly | 2 | 21 | 0.0207 | APC,CTNNB1 |
| **GO:0043405** | regulation of MAP kinase activity | 6 | 337 | 0.0209 | TGFB1,EGFR,TDGF1,RET,ROR2,BMP7 |
| **GO:0046427** | positive regulation of receptor signaling pathway via JAK-STAT | 3 | 73 | 0.0212 | TNFRSF1A,NOTCH1,FGFR3 |
| **GO:0072422** | signal transduction involved in DNA damage checkpoint | 3 | 73 | 0.0212 | EP300,ATM,BRCA1 |
| **GO:0050730** | regulation of peptidyl-tyrosine phosphorylation | 5 | 237 | 0.0213 | TNFRSF1A,TGFB1,EGFR,TDGF1,FGFR3 |
| **GO:0051216** | cartilage development | 4 | 147 | 0.0213 | TGFB1,FGFR3,ROR2,BMP7 |
| **GO:0051240** | positive regulation of multicellular organismal process | 15 | 1551 | 0.0213 | TGFB1,JAG1,EP300,EGFR,NOTCH1,TDGF1,CTNNB1,RET,HLA-DRB1,ROR2,CDON,BMP7,PAX6,BRCA1,PIK3R1 |
| **GO:0033554** | cellular response to stress | 15 | 1553 | 0.0215 | TNFRSF1A,APC,POMT2,SMARCB1,EP300,EGFR,NOTCH1,ATM,POMT1,ROR2,BRCA2,BMP7,BRCA1,PIK3R1,TMCO1 |
| **GO:0006486** | protein glycosylation | 5 | 238 | 0.0216 | FKTN,POMT2,LARGE,HLA-DRB1,POMT1 |
| **GO:0021513** | spinal cord dorsal/ventral patterning | 2 | 22 | 0.0217 | IFT122,PAX6 |
| **GO:0034332** | adherens junction organization | 3 | 74 | 0.0217 | CTNNB1,ACTN1,CTNND1 |
| **GO:0036294** | cellular response to decreased oxygen levels | 4 | 148 | 0.0217 | EP300,NOTCH1,TSC1,BMP7 |
| **GO:0043627** | response to estrogen | 3 | 74 | 0.0217 | EP300,ESR1,BRCA1 |
| **GO:0051147** | regulation of muscle cell differentiation | 4 | 148 | 0.0217 | TGFB1,NOTCH1,CTNNB1,CDON |
| **GO:0060411** | cardiac septum morphogenesis | 3 | 74 | 0.0217 | JAG1,NOTCH1,BMP7 |
| **GO:0060740** | prostate gland epithelium morphogenesis | 2 | 22 | 0.0217 | NOTCH1,ESR1 |
| **GO:0097150** | neuronal stem cell population maintenance | 2 | 22 | 0.0217 | JAG1,NOTCH1 |
| **GO:1900407** | regulation of cellular response to oxidative stress | 3 | 74 | 0.0217 | TSC1,CTNNB1,BMP7 |
| **GO:1903707** | negative regulation of hemopoiesis | 4 | 148 | 0.0217 | NOTCH1,FBN1,CTNNB1,PIK3R1 |
| **GO:0007050** | cell cycle arrest | 4 | 149 | 0.0218 | TGFB1,APC,NOTCH1,ATM |
| **GO:0010212** | response to ionizing radiation | 4 | 149 | 0.0218 | TGFB1,ATM,BRCA2,BRCA1 |
| **GO:0034976** | response to endoplasmic reticulum stress | 5 | 240 | 0.0219 | POMT2,EP300,POMT1,PIK3R1,TMCO1 |
| **GO:0033045** | regulation of sister chromatid segregation | 3 | 75 | 0.0221 | APC,ATM,CTNNB1 |
| **GO:0090407** | organophosphate biosynthetic process | 8 | 577 | 0.0222 | DHODH,TGFB1,ATM,FGFR3,INPP5E,PIGO,PIGG,PIK3R1 |
| **GO:0046425** | regulation of receptor signaling pathway via JAK-STAT | 4 | 151 | 0.0226 | TNFRSF1A,NOTCH1,FGFR3,RET |
| **GO:0000729** | DNA double-strand break processing | 2 | 23 | 0.0229 | ATM,BRCA1 |
| **GO:0010614** | negative regulation of cardiac muscle hypertrophy | 2 | 23 | 0.0229 | TNFRSF1A,NOTCH1 |
| **GO:0016570** | histone modification | 6 | 347 | 0.0229 | EP300,ATM,ASXL1,KDM6A,BRCA2,KMT2A |
| **GO:0018193** | peptidyl-amino acid modification | 10 | 842 | 0.0229 | EP300,EGFR,ATM,TDGF1,FGFR3,RET,ROR2,KDM6A,BRCA2,KMT2A |
| **GO:0030539** | male genitalia development | 2 | 23 | 0.0229 | CTNNB1,ROR2 |
| **GO:0042060** | wound healing | 7 | 461 | 0.0229 | TGFB1,EGFR,NOTCH1,COL1A2,LARGE,STXBP1,PIK3R1 |
| **GO:0070168** | negative regulation of biomineral tissue development | 2 | 23 | 0.0229 | TGFB1,NOTCH1 |
| **GO:0070723** | response to cholesterol | 2 | 23 | 0.0229 | TGFB1,PTCH1 |
| **GO:0072215** | regulation of metanephros development | 2 | 23 | 0.0229 | CTNNB1,RET |
| **GO:1901522** | positive regulation of transcription from RNA polymerase II promoter involved in cellular response to chemical stimulus | 2 | 23 | 0.0229 | EP300,NOTCH1 |
| **GO:0007162** | negative regulation of cell adhesion | 5 | 245 | 0.0232 | TGFB1,JAG1,NOTCH1,HLA-DRB1,PIK3R1 |
| **GO:0071478** | cellular response to radiation | 4 | 153 | 0.0232 | TGFB1,EP300,ATM,PIK3R1 |
| **GO:0007098** | centrosome cycle | 3 | 78 | 0.0239 | CTNNB1,BRCA2,BRCA1 |
| **GO:0042475** | odontogenesis of dentin-containing tooth | 3 | 78 | 0.0239 | EDAR,CTNNB1,BMP7 |
| **GO:0071260** | cellular response to mechanical stimulus | 3 | 78 | 0.0239 | TNFRSF1A,TGFB1,EGFR |
| **GO:0001894** | tissue homeostasis | 4 | 155 | 0.0241 | NOTCH1,CTNNB1,ABCA4,KMT2A |
| **GO:0002437** | inflammatory response to antigenic stimulus | 2 | 24 | 0.0241 | NOTCH1,HLA-DRB1 |
| **GO:0002719** | negative regulation of cytokine production involved in immune response | 2 | 24 | 0.0241 | TGFB1,CD96 |
| **GO:0048596** | embryonic camera-type eye morphogenesis | 2 | 24 | 0.0241 | BMP7,PAX6 |
| **GO:2000241** | regulation of reproductive process | 4 | 155 | 0.0241 | NOTCH1,CTNNB1,BMP7,ESR1 |
| **GO:2000679** | positive regulation of transcription regulatory region DNA binding | 2 | 24 | 0.0241 | TGFB1,CTNNB1 |
| **GO:0006338** | chromatin remodeling | 4 | 156 | 0.0243 | SMARCB1,CTNNB1,KDM6A,ESR1 |
| **GO:0030518** | intracellular steroid hormone receptor signaling pathway | 3 | 79 | 0.0243 | CTNNB1,ESR1,BRCA1 |
| **GO:0072659** | protein localization to plasma membrane | 4 | 156 | 0.0243 | TNFRSF1A,ANK1,PTCH1,STXBP1 |
| **GO:0043433** | negative regulation of DNA-binding transcription factor activity | 4 | 157 | 0.0247 | PTCH1,FOXH1,BMP7,ESR1 |
| **GO:0051091** | positive regulation of DNA-binding transcription factor activity | 5 | 250 | 0.0247 | TGFB1,SMARCB1,EP300,CTNNB1,ESR1 |
| **GO:0051098** | regulation of binding | 6 | 356 | 0.0249 | TGFB1,EP300,CTNNB1,RPL11,SKI,CDON |
| **GO:0007548** | sex differentiation | 5 | 252 | 0.0253 | ATM,CTNNB1,ROR2,BRCA2,ESR1 |
| **GO:0043066** | negative regulation of apoptotic process | 10 | 859 | 0.0253 | EGFR,NOTCH1,TDGF1,CTNNB1,AHI1,STXBP1,ADAMTS20,BMP7,BRCA1,PIK3R1 |
| **GO:0060765** | regulation of androgen receptor signaling pathway | 2 | 25 | 0.0254 | EP300,FOXH1 |
| **GO:0070167** | regulation of biomineral tissue development | 3 | 81 | 0.0256 | TGFB1,NOTCH1,BMP7 |
| **GO:1903321** | negative regulation of protein modification by small protein conjugation or removal | 3 | 81 | 0.0256 | RPS7,CTNNB1,RPL11 |
| **GO:0007088** | regulation of mitotic nuclear division | 4 | 160 | 0.0260 | TGFB1,APC,ATM,BMP7 |
| **GO:1901991** | negative regulation of mitotic cell cycle phase transition | 4 | 160 | 0.0260 | APC,EP300,ATM,BRCA1 |
| **GO:0043434** | response to peptide hormone | 6 | 362 | 0.0264 | TGFB1,APC,TSC1,FBN1,BMP7,PIK3R1 |
| **GO:0010762** | regulation of fibroblast migration | 2 | 26 | 0.0270 | TGFB1,WDPCP |
| **GO:0019637** | organophosphate metabolic process | 11 | 1011 | 0.0270 | DHODH,TGFB1,EGFR,ATM,FGFR3,INPP5E,PIGO,ESR1,PIGG,PIK3R1,MYH3 |
| **GO:0031069** | hair follicle morphogenesis | 2 | 26 | 0.0270 | NOTCH1,CTNNB1 |
| **GO:0043534** | blood vessel endothelial cell migration | 2 | 26 | 0.0270 | MYH9,TDGF1 |
| **GO:0060037** | pharyngeal system development | 2 | 26 | 0.0270 | PTCH1,BMP7 |
| **GO:0097306** | cellular response to alcohol | 3 | 83 | 0.0270 | PTCH1,CTNNB1,BRCA1 |
| **GO:0110110** | positive regulation of animal organ morphogenesis | 3 | 83 | 0.0270 | TGFB1,CTNNB1,BMP7 |
| **GO:1905209** | positive regulation of cardiocyte differentiation | 2 | 26 | 0.0270 | TGFB1,BMP7 |
| **GO:0008585** | female gonad development | 3 | 84 | 0.0276 | ATM,BRCA2,ESR1 |
| **GO:2001022** | positive regulation of response to DNA damage stimulus | 3 | 84 | 0.0276 | EGFR,ATM,BRCA1 |
| **GO:0008284** | positive regulation of cell population proliferation | 10 | 878 | 0.0284 | MMP2,TGFB1,EGFR,NOTCH1,TDGF1,FGFR3,CTNNB1,CDON,PAX6,ESR1 |
| **GO:0010575** | positive regulation of vascular endothelial growth factor production | 2 | 27 | 0.0284 | TGFB1,BRCA1 |
| **GO:0043921** | modulation by host of viral transcription | 2 | 27 | 0.0284 | SMARCB1,EP300 |
| **GO:0045662** | negative regulation of myoblast differentiation | 2 | 27 | 0.0284 | TGFB1,NOTCH1 |
| **GO:0048643** | positive regulation of skeletal muscle tissue development | 2 | 27 | 0.0284 | CTNNB1,CDON |
| **GO:0051149** | positive regulation of muscle cell differentiation | 3 | 85 | 0.0284 | TGFB1,CTNNB1,CDON |
| **GO:0052472** | modulation by host of symbiont transcription | 2 | 27 | 0.0284 | SMARCB1,EP300 |
| **GO:0071900** | regulation of protein serine/threonine kinase activity | 7 | 488 | 0.0286 | TGFB1,APC,EGFR,TDGF1,RET,ROR2,BMP7 |
| **GO:0001817** | regulation of cytokine production | 8 | 615 | 0.0291 | TGFB1,EP300,CD96,CTNNB1,HLA-DRB1,HLA-B,BRCA1,PIK3R1 |
| **GO:0043406** | positive regulation of MAP kinase activity | 5 | 264 | 0.0291 | TGFB1,EGFR,TDGF1,RET,ROR2 |
| **GO:0051707** | response to other organism | 12 | 1173 | 0.0295 | TNFRSF1A,TGFB1,NOTCH1,CD96,TDGF1,WDR35,HLA-DRB1,STXBP1,HLA-B,COLEC11,FLNB,ACTG1 |
| **GO:0003338** | metanephros morphogenesis | 2 | 28 | 0.0297 | FRAS1,CTNNB1 |
| **GO:0007274** | neuromuscular synaptic transmission | 2 | 28 | 0.0297 | STXBP1,CHRNG |
| **GO:0021953** | central nervous system neuron differentiation | 4 | 169 | 0.0297 | PTCH1,CTNNB1,DYNC2H1,PAX6 |
| **GO:0031063** | regulation of histone deacetylation | 2 | 28 | 0.0297 | TGFB1,SKI |
| **GO:0031327** | negative regulation of cellular biosynthetic process | 14 | 1479 | 0.0297 | TGFB1,EP300,NOTCH1,TSC1,WDR35,PTCH1,CTNNB1,FOXH1,SKI,BRCA2,BMP7,PAX6,ESR1,BRCA1 |
| **GO:0033273** | response to vitamin | 3 | 87 | 0.0297 | TGFB1,EGFR,BMP7 |
| **GO:0035116** | embryonic hindlimb morphogenesis | 2 | 28 | 0.0297 | NOTCH1,CTNNB1 |
| **GO:0035909** | aorta morphogenesis | 2 | 28 | 0.0297 | JAG1,FOXH1 |
| **GO:0046328** | regulation of JNK cascade | 4 | 169 | 0.0297 | FKTN,EDAR,EGFR,ROR2 |
| **GO:0060021** | roof of mouth development | 3 | 87 | 0.0297 | WDPCP,FRAS1,SKI |
| **GO:0060317** | cardiac epithelial to mesenchymal transition | 2 | 28 | 0.0297 | JAG1,NOTCH1 |
| **GO:0071549** | cellular response to dexamethasone stimulus | 2 | 28 | 0.0297 | TGFB1,EGFR |
| **GO:1903076** | regulation of protein localization to plasma membrane | 3 | 87 | 0.0297 | TGFB1,EGFR,PIK3R1 |
| **GO:0030216** | keratinocyte differentiation | 5 | 267 | 0.0299 | KRT14,JAG1,NOTCH1,PAX6,FLNB |
| **GO:0045787** | positive regulation of cell cycle | 6 | 376 | 0.0299 | TGFB1,EP300,EGFR,ATM,BRCA2,BRCA1 |
| **GO:0000724** | double-strand break repair via homologous recombination | 3 | 88 | 0.0300 | ATM,BRCA2,BRCA1 |
| **GO:0034645** | cellular macromolecule biosynthetic process | 26 | 3518 | 0.0308 | FKTN,POMT2,SMARCB1,EP300,EGFR,NOTCH1,ATM,RPS7,CTNNB1,LARGE,RPS26,RPS27,POMT1,RPL11,ASXL1,FOXH1,SKI,PIGO,BRCA2,PAX1,CTNND1,PAX6,ESR1,PIGG,BRCA1,KMT2A |
| **GO:0045321** | leukocyte activation | 10 | 894 | 0.0308 | MYH9,TGFB1,EP300,ATM,TSC1,CTNNB1,PAX1,HLA-B,PIK3R1,CEP290 |
| **GO:0045639** | positive regulation of myeloid cell differentiation | 3 | 89 | 0.0308 | JAG1,HLA-DRB1,ROR2 |
| **GO:0014002** | astrocyte development | 2 | 29 | 0.0310 | EGFR,ROR2 |
| **GO:0040036** | regulation of fibroblast growth factor receptor signaling pathway | 2 | 29 | 0.0310 | FUZ,CTNNB1 |
| **GO:0044818** | mitotic G2/M transition checkpoint | 2 | 29 | 0.0310 | ATM,BRCA1 |
| **GO:0051271** | negative regulation of cellular component movement | 5 | 270 | 0.0310 | TGFB1,JAG1,NOTCH1,FUZ,ACTN1 |
| **GO:1901137** | carbohydrate derivative biosynthetic process | 8 | 625 | 0.0310 | DHODH,TGFB1,FKTN,POMT2,LARGE,POMT1,PIGO,PIGG |
| **GO:1904837** | beta-catenin-TCF complex assembly | 2 | 29 | 0.0310 | EP300,CTNNB1 |
| **GO:2000144** | positive regulation of DNA-templated transcription, initiation | 2 | 29 | 0.0310 | CTNNB1,ESR1 |
| **GO:0009791** | post-embryonic development | 3 | 90 | 0.0313 | ATM,FBN1,KMT2A |
| **GO:0097435** | supramolecular fiber organization | 6 | 383 | 0.0318 | KRT14,COL1A2,ACTA1,ACTN1,ACTG1,MYH3 |
| **GO:1905114** | cell surface receptor signaling pathway involved in cell-cell signaling | 6 | 383 | 0.0318 | APC,CTNNB1,ROR2,KDM6A,CHRNG,CTNND1 |
| **GO:0010948** | negative regulation of cell cycle process | 5 | 273 | 0.0319 | APC,EP300,ATM,BMP7,BRCA1 |
| **GO:0032388** | positive regulation of intracellular transport | 4 | 174 | 0.0319 | TGFB1,STXBP1,PIK3R1,CEP290 |
| **GO:0010800** | positive regulation of peptidyl-threonine phosphorylation | 2 | 30 | 0.0324 | TGFB1,BMP7 |
| **GO:0033280** | response to vitamin D | 2 | 30 | 0.0324 | TGFB1,BMP7 |
| **GO:0042771** | intrinsic apoptotic signaling pathway in response to DNA damage by p53 class mediator | 2 | 30 | 0.0324 | EP300,BRCA2 |
| **GO:2000113** | negative regulation of cellular macromolecule biosynthetic process | 13 | 1348 | 0.0326 | TGFB1,EP300,NOTCH1,TSC1,PTCH1,CTNNB1,FOXH1,SKI,BRCA2,BMP7,PAX6,ESR1,BRCA1 |
| **GO:0042472** | inner ear morphogenesis | 3 | 92 | 0.0327 | WDPCP,ROR2,CEP290 |
| **GO:0002703** | regulation of leukocyte mediated immunity | 4 | 176 | 0.0328 | TGFB1,CD96,STXBP1,HLA-B |
| **GO:1901576** | organic substance biosynthetic process | 32 | 4656 | 0.0328 | DHODH,TGFB1,FKTN,POMT2,SMARCB1,EP300,EGFR,NOTCH1,ATM,RPS7,FGFR3,LBR,CTNNB1,LARGE,RPS26,RPS27,INPP5E,POMT1,RPL11,ASXL1,FOXH1,SKI,PIGO,BRCA2,PAX1,CTNND1,PAX6,ESR1,PIGG,BRCA1,PIK3R1,KMT2A |
| **GO:1903708** | positive regulation of hemopoiesis | 4 | 177 | 0.0333 | TGFB1,JAG1,HLA-DRB1,ROR2 |
| **GO:0042552** | myelination | 3 | 93 | 0.0335 | TGFB1,TSC1,SKI |
| **GO:0060070** | canonical Wnt signaling pathway | 3 | 93 | 0.0335 | APC,CTNNB1,KDM6A |
| **GO:0006506** | GPI anchor biosynthetic process | 2 | 31 | 0.0338 | PIGO,PIGG |
| **GO:0035115** | embryonic forelimb morphogenesis | 2 | 31 | 0.0338 | IFT122,CTNNB1 |
| **GO:0038128** | ERBB2 signaling pathway | 2 | 31 | 0.0338 | EGFR,PIK3R1 |
| **GO:0050679** | positive regulation of epithelial cell proliferation | 4 | 178 | 0.0338 | TGFB1,EGFR,NOTCH1,CTNNB1 |
| **GO:0048771** | tissue remodeling | 3 | 94 | 0.0342 | TGFB1,JAG1,CTNNB1 |
| **GO:0051302** | regulation of cell division | 4 | 179 | 0.0342 | TGFB1,PTCH1,BRCA2,PAX6 |
| **GO:0065003** | protein-containing complex assembly | 14 | 1514 | 0.0342 | TNFRSF1A,TGFB1,APC,SMARCB1,EP300,COL1A2,TSC1,CTNNB1,RYR1,HLA-DRB1,RPS27,RPL11,SKI,KMT2A |
| **GO:2000379** | positive regulation of reactive oxygen species metabolic process | 3 | 94 | 0.0342 | TGFB1,EGFR,ESR1 |
| **GO:0000226** | microtubule cytoskeleton organization | 6 | 393 | 0.0348 | MYH9,CTNNB1,BRCA2,CC2D2A,PAX6,BRCA1 |
| **GO:0007565** | female pregnancy | 4 | 180 | 0.0348 | MMP2,DHODH,TGFB1,ESR1 |
| **GO:0031348** | negative regulation of defense response | 4 | 180 | 0.0348 | TNFRSF1A,CD96,HLA-DRB1,HLA-B |
| **GO:0050731** | positive regulation of peptidyl-tyrosine phosphorylation | 4 | 180 | 0.0348 | TNFRSF1A,TGFB1,TDGF1,FGFR3 |
| **GO:0008625** | extrinsic apoptotic signaling pathway via death domain receptors | 2 | 32 | 0.0354 | TNFRSF1A,PIK3R1 |
| **GO:0032689** | negative regulation of interferon-gamma production | 2 | 32 | 0.0354 | CD96,HLA-DRB1 |
| **GO:0035850** | epithelial cell differentiation involved in kidney development | 2 | 32 | 0.0354 | JAG1,NOTCH1 |
| **GO:0043537** | negative regulation of blood vessel endothelial cell migration | 2 | 32 | 0.0354 | TGFB1,NOTCH1 |
| **GO:0051145** | smooth muscle cell differentiation | 2 | 32 | 0.0354 | NOTCH1,CTNNB1 |
| **GO:0051568** | histone H3-K4 methylation | 2 | 32 | 0.0354 | KDM6A,KMT2A |
| **GO:0061418** | regulation of transcription from RNA polymerase II promoter in response to hypoxia | 2 | 32 | 0.0354 | EP300,NOTCH1 |
| **GO:1901566** | organonitrogen compound biosynthetic process | 13 | 1370 | 0.0356 | DHODH,TGFB1,FKTN,POMT2,EGFR,RPS7,LARGE,RPS26,RPS27,POMT1,RPL11,PIGO,PIGG |
| **GO:1901222** | regulation of NIK/NF-kappaB signaling | 3 | 97 | 0.0362 | EDAR,EGFR,BMP7 |
| **GO:0001953** | negative regulation of cell-matrix adhesion | 2 | 33 | 0.0367 | JAG1,PIK3R1 |
| **GO:0010165** | response to X-ray | 2 | 33 | 0.0367 | ATM,BRCA2 |
| **GO:0034333** | adherens junction assembly | 2 | 33 | 0.0367 | CTNNB1,ACTN1 |
| **GO:0043552** | positive regulation of phosphatidylinositol 3-kinase activity | 2 | 33 | 0.0367 | TGFB1,FGFR3 |
| **GO:0045859** | regulation of protein kinase activity | 9 | 788 | 0.0367 | TGFB1,APC,EGFR,TDGF1,TSC1,FBN1,RET,ROR2,BMP7 |
| **GO:0048873** | homeostasis of number of cells within a tissue | 2 | 33 | 0.0367 | NOTCH1,KMT2A |
| **GO:1903829** | positive regulation of cellular protein localization | 5 | 287 | 0.0367 | TGFB1,APC,EGFR,PIK3R1,CEP290 |
| **GO:0043900** | regulation of multi-organism process | 8 | 653 | 0.0368 | MMP2,SMARCB1,EP300,NOTCH1,CD96,HLA-DRB1,HLA-B,ESR1 |
| **GO:0019221** | cytokine-mediated signaling pathway | 8 | 655 | 0.0373 | TNFRSF1A,MMP2,TGFB1,EDAR,COL1A2,HLA-DRB1,HLA-B,PIK3R1 |
| **GO:0043085** | positive regulation of catalytic activity | 13 | 1381 | 0.0374 | TGFB1,ARHGAP31,EGFR,TDGF1,TSC1,WDR35,FBN1,FGFR3,CTNNB1,RET,HLA-DRB1,ROR2,ESR1 |
| **GO:0030183** | B cell differentiation | 3 | 99 | 0.0376 | EP300,ATM,PIK3R1 |
| **GO:0032212** | positive regulation of telomere maintenance via telomerase | 2 | 34 | 0.0383 | ATM,CTNNB1 |
| **GO:0042462** | eye photoreceptor cell development | 2 | 34 | 0.0383 | PAX6,CEP290 |
| **GO:0006952** | defense response | 12 | 1234 | 0.0385 | TNFRSF1A,TGFB1,EGFR,NOTCH1,CD96,TDGF1,HLA-DRB1,STXBP1,HLA-B,COLEC11,FLNB,ACTG1 |
| **GO:0006606** | protein import into nucleus | 3 | 101 | 0.0395 | TGFB1,NUP188,PIK3R1 |
| **GO:0007223** | Wnt signaling pathway, calcium modulating pathway | 2 | 35 | 0.0402 | CTNNB1,ROR2 |
| **GO:0030501** | positive regulation of bone mineralization | 2 | 35 | 0.0402 | TGFB1,BMP7 |
| **GO:0048645** | animal organ formation | 2 | 35 | 0.0402 | CTNNB1,FOXH1 |
| **GO:0002573** | myeloid leukocyte differentiation | 3 | 102 | 0.0404 | MYH9,TGFB1,CTNNB1 |
| **GO:0090100** | positive regulation of transmembrane receptor protein serine/threonine kinase signaling pathway | 3 | 102 | 0.0404 | TGFB1,NOTCH1,BMP7 |
| **GO:0035051** | cardiocyte differentiation | 3 | 103 | 0.0413 | JAG1,NOTCH1,TSC1 |
| **GO:0006955** | immune response | 14 | 1560 | 0.0414 | TNFRSF1A,TGFB1,JAG1,NOTCH1,CD96,TDGF1,TSC1,HLA-DRB1,STXBP1,HLA-B,COLEC11,FLNB,CEP290,ACTG1 |
| **GO:0048872** | homeostasis of number of cells | 4 | 193 | 0.0416 | TGFB1,NOTCH1,ASXL1,KMT2A |
| **GO:0032496** | response to lipopolysaccharide | 5 | 298 | 0.0417 | TNFRSF1A,TGFB1,NOTCH1,CD96,WDR35 |
| **GO:0000122** | negative regulation of transcription by RNA polymerase II | 9 | 809 | 0.0418 | TGFB1,EP300,NOTCH1,PTCH1,CTNNB1,FOXH1,SKI,PAX6,ESR1 |
| **GO:0032526** | response to retinoic acid | 3 | 104 | 0.0418 | PTCH1,RET,ASXL1 |
| **GO:0044249** | cellular biosynthetic process | 31 | 4567 | 0.0418 | DHODH,TGFB1,FKTN,POMT2,SMARCB1,EP300,EGFR,NOTCH1,ATM,RPS7,FGFR3,CTNNB1,LARGE,RPS26,RPS27,INPP5E,POMT1,RPL11,ASXL1,FOXH1,SKI,PIGO,BRCA2,PAX1,CTNND1,PAX6,ESR1,PIGG,BRCA1,PIK3R1,KMT2A |
| **GO:0051293** | establishment of spindle localization | 2 | 36 | 0.0418 | MYH9,PAX6 |
| **GO:0071392** | cellular response to estradiol stimulus | 2 | 36 | 0.0418 | EGFR,ESR1 |
| **GO:1901532** | regulation of hematopoietic progenitor cell differentiation | 2 | 36 | 0.0418 | NOTCH1,KMT2A |
| **GO:0098609** | cell-cell adhesion | 6 | 416 | 0.0422 | EGFR,CTNNB1,RET,STXBP1,BMP7,CTNND1 |
| **GO:0018108** | peptidyl-tyrosine phosphorylation | 4 | 195 | 0.0425 | EGFR,FGFR3,RET,ROR2 |
| **GO:0030048** | actin filament-based movement | 3 | 105 | 0.0427 | MYH9,ACTA1,MYH3 |
| **GO:0033138** | positive regulation of peptidyl-serine phosphorylation | 3 | 105 | 0.0427 | TGFB1,EGFR,RET |
| **GO:0045087** | innate immune response | 8 | 676 | 0.0429 | TGFB1,TDGF1,HLA-DRB1,STXBP1,HLA-B,COLEC11,FLNB,ACTG1 |
| **GO:0071229** | cellular response to acid chemical | 4 | 196 | 0.0430 | MMP2,EGFR,COL1A2,RET |
| **GO:0045746** | negative regulation of Notch signaling pathway | 2 | 37 | 0.0432 | EGFR,BMP7 |
| **GO:0016055** | Wnt signaling pathway | 5 | 303 | 0.0436 | APC,CTNNB1,ROR2,KDM6A,CTNND1 |
| **GO:0007049** | cell cycle | 12 | 1263 | 0.0441 | MYH9,TGFB1,APC,SMARCB1,EP300,NOTCH1,ATM,CTNNB1,BRCA2,PAX6,BRCA1,CEP290 |
| **GO:0051817** | modification of morphology or physiology of other organism involved in symbiotic interaction | 3 | 107 | 0.0443 | TGFB1,SMARCB1,EP300 |
| **GO:0002067** | glandular epithelial cell differentiation | 2 | 38 | 0.0450 | NOTCH1,PAX6 |
| **GO:0030049** | muscle filament sliding | 2 | 38 | 0.0450 | ACTA1,MYH3 |
| **GO:0030316** | osteoclast differentiation | 2 | 38 | 0.0450 | TGFB1,CTNNB1 |
| **GO:0045214** | sarcomere organization | 2 | 38 | 0.0450 | ACTG1,MYH3 |
| **GO:0045445** | myoblast differentiation | 2 | 38 | 0.0450 | JAG1,NOTCH1 |
| **GO:0043254** | regulation of protein complex assembly | 6 | 425 | 0.0453 | TGFB1,EP300,ATM,CTNNB1,STXBP1,ESR1 |
| **GO:0050769** | positive regulation of neurogenesis | 6 | 425 | 0.0453 | NOTCH1,CTNNB1,RET,CDON,BMP7,PAX6 |
| **GO:0002705** | positive regulation of leukocyte mediated immunity | 3 | 109 | 0.0459 | TGFB1,STXBP1,HLA-B |
| **GO:0009617** | response to bacterium | 7 | 555 | 0.0466 | TNFRSF1A,TGFB1,NOTCH1,CD96,WDR35,HLA-DRB1,HLA-B |
| **GO:0032102** | negative regulation of response to external stimulus | 5 | 310 | 0.0467 | TNFRSF1A,NOTCH1,CD96,HLA-DRB1,HLA-B |
| **GO:0051056** | regulation of small GTPase mediated signal transduction | 5 | 310 | 0.0467 | ARHGAP31,NOTCH1,FGD1,CDON,FGD4 |
| **GO:0007044** | cell-substrate junction assembly | 2 | 40 | 0.0485 | KRT14,ACTN1 |
| **GO:0010863** | positive regulation of phospholipase C activity | 2 | 40 | 0.0485 | EGFR,ESR1 |
| **GO:0046849** | bone remodeling | 2 | 40 | 0.0485 | TGFB1,CTNNB1 |
| **GO:0050670** | regulation of lymphocyte proliferation | 4 | 205 | 0.0485 | TGFB1,ATM,CTNNB1,HLA-DRB1 |
| **GO:0071364** | cellular response to epidermal growth factor stimulus | 2 | 40 | 0.0485 | EGFR,TDGF1 |
| **GO:0090184** | positive regulation of kidney development | 2 | 40 | 0.0485 | TGFB1,RET |
| **GO:1901343** | negative regulation of vasculature development | 3 | 112 | 0.0487 | NOTCH1,CTNNB1,BMP7 |
| **GO:0002698** | negative regulation of immune effector process | 3 | 113 | 0.0497 | TGFB1,CD96,HLA-B |
