# Supplementary material for: Identification of Novel Variants in Cleft Palate-Associated Genes in Brazilian Patients With Non-syndromic Cleft Palate Only
Source: Front Cell Dev Biol. 2021 Jul 8;9:638522. doi: 10.3389/fcell.2021.638522 (PMC8297955; doi:10.3389/fcell.2021.638522)
Supplement: Supplementary file 11 [file Data_Sheet_9.docx]

| **Supplementary Table 9.** Activated pathways characterized with cleft lip-palate variant-containing-genes identified in patients with nonsyndromic cleft palate only (NSCPO). | | | | | |
| --- | --- | --- | --- | --- | --- |
| **Pathway ID** | **Term description** | **Observed gene count** | **Background gene count** | **False discovery rate** | **Matching proteins in your network** |
| **hsa05165** | Human papillomavirus infection | 12 | 317 | 3.58e-06 | TNFRSF1A,JAG1,APC,EP300,EGFR,NOTCH1,ATM,COL1A2,TSC1,CTNNB1,HLA-B,PIK3R1 |
| **hsa05200** | Pathways in cancer | 15 | 515 | 3.58e-06 | MMP2,TGFB1,JAG1,APC,EP300,EGFR,NOTCH1,PTCH1,FGFR3,CTNNB1,RET,PTCH2,BRCA2,ESR1,PIK3R1 |
| **hsa05205** | Proteoglycans in cancer | 10 | 195 | 3.58e-06 | MMP2,TGFB1,ANK1,EGFR,PTCH1,CTNNB1,ESR1,FLNB,PIK3R1,ACTG1 |
| **hsa05224** | Breast cancer | 9 | 147 | 3.58e-06 | JAG1,APC,EGFR,NOTCH1,CTNNB1,BRCA2,ESR1,BRCA1,PIK3R1 |
| **hsa04520** | Adherens junction | 6 | 71 | 5.49e-05 | EP300,EGFR,CTNNB1,ACTN1,CTNND1,ACTG1 |
| **hsa05166** | HTLV-I infection | 9 | 250 | 9.61e-05 | TNFRSF1A,TGFB1,APC,EP300,ATM,CTNNB1,HLA-DRB1,HLA-B,PIK3R1 |
| **hsa00515** | Mannose type O-glycan biosynthesis | 4 | 22 | 0.00015 | FKTN,POMT2,LARGE,POMT1 |
| **hsa01522** | Endocrine resistance | 6 | 95 | 0.00015 | MMP2,JAG1,EGFR,NOTCH1,ESR1,PIK3R1 |
| **hsa04810** | Regulation of actin cytoskeleton | 8 | 205 | 0.00015 | MYH9,APC,EGFR,FGFR3,FGD1,ACTN1,PIK3R1,ACTG1 |
| **hsa05206** | MicroRNAs in cancer | 7 | 149 | 0.00015 | APC,EP300,EGFR,NOTCH1,ATM,FGFR3,BRCA1 |
| **hsa05225** | Hepatocellular carcinoma | 7 | 163 | 0.00023 | TGFB1,APC,SMARCB1,EGFR,CTNNB1,PIK3R1,ACTG1 |
| **hsa04670** | Leukocyte transendothelial migration | 6 | 112 | 0.00027 | MMP2,CTNNB1,ACTN1,CTNND1,PIK3R1,ACTG1 |
| **hsa04919** | Thyroid hormone signaling pathway | 6 | 115 | 0.00029 | EP300,NOTCH1,CTNNB1,ESR1,PIK3R1,ACTG1 |
| **hsa04510** | Focal adhesion | 7 | 197 | 0.00059 | EGFR,COL1A2,CTNNB1,ACTN1,FLNB,PIK3R1,ACTG1 |
| **hsa05210** | Colorectal cancer | 5 | 85 | 0.00074 | TGFB1,APC,EGFR,CTNNB1,PIK3R1 |
| **hsa05213** | Endometrial cancer | 4 | 58 | 0.0022 | APC,EGFR,CTNNB1,PIK3R1 |
| **hsa05217** | Basal cell carcinoma | 4 | 63 | 0.0028 | APC,PTCH1,CTNNB1,PTCH2 |
| **hsa05230** | Central carbon metabolism in cancer | 4 | 65 | 0.0030 | EGFR,FGFR3,RET,PIK3R1 |
| **hsa04015** | Rap1 signaling pathway | 6 | 203 | 0.0039 | EGFR,FGFR3,CTNNB1,CTNND1,PIK3R1,ACTG1 |
| **hsa04068** | FoxO signaling pathway | 5 | 130 | 0.0039 | TGFB1,EP300,EGFR,ATM,PIK3R1 |
| **hsa04550** | Signaling pathways regulating pluripotency of stem cells | 5 | 138 | 0.0039 | APC,FGFR3,CTNNB1,PAX6,PIK3R1 |
| **hsa04915** | Estrogen signaling pathway | 5 | 133 | 0.0039 | KRT14,MMP2,EGFR,ESR1,PIK3R1 |
| **hsa04926** | Relaxin signaling pathway | 5 | 130 | 0.0039 | MMP2,TGFB1,EGFR,COL1A2,PIK3R1 |
| **hsa05212** | Pancreatic cancer | 4 | 74 | 0.0039 | TGFB1,EGFR,BRCA2,PIK3R1 |
| **hsa05418** | Fluid shear stress and atherosclerosis | 5 | 133 | 0.0039 | TNFRSF1A,MMP2,CTNNB1,PIK3R1,ACTG1 |
| **hsa04145** | Phagosome | 5 | 145 | 0.0046 | HLA-DRB1,DYNC2H1,HLA-B,COLEC11,ACTG1 |
| **hsa05226** | Gastric cancer | 5 | 147 | 0.0047 | TGFB1,APC,EGFR,CTNNB1,PIK3R1 |
| **hsa05132** | Salmonella infection | 4 | 84 | 0.0048 | MYH9,DYNC2H1,FLNB,ACTG1 |
| **hsa04390** | Hippo signaling pathway | 5 | 152 | 0.0051 | TGFB1,APC,CTNNB1,BMP7,ACTG1 |
| **hsa04218** | Cellular senescence | 5 | 156 | 0.0055 | TGFB1,ATM,TSC1,HLA-B,PIK3R1 |
| **hsa03440** | Homologous recombination | 3 | 40 | 0.0060 | ATM,BRCA2,BRCA1 |
| **hsa05146** | Amoebiasis | 4 | 94 | 0.0063 | TGFB1,COL1A2,ACTN1,PIK3R1 |
| **hsa05219** | Bladder cancer | 3 | 41 | 0.0063 | MMP2,EGFR,FGFR3 |
| **hsa04933** | AGE-RAGE signaling pathway in diabetic complications | 4 | 98 | 0.0066 | MMP2,TGFB1,COL1A2,PIK3R1 |
| **hsa05164** | Influenza A | 5 | 168 | 0.0066 | TNFRSF1A,EP300,HLA-DRB1,PIK3R1,ACTG1 |
| **hsa05215** | Prostate cancer | 4 | 97 | 0.0066 | EP300,EGFR,CTNNB1,PIK3R1 |
| **hsa04340** | Hedgehog signaling pathway | 3 | 46 | 0.0074 | PTCH1,PTCH2,CDON |
| **hsa04330** | Notch signaling pathway | 3 | 48 | 0.0081 | JAG1,EP300,NOTCH1 |
| **hsa05167** | Kaposi's sarcoma-associated herpesvirus infection | 5 | 183 | 0.0084 | TNFRSF1A,EP300,CTNNB1,HLA-B,PIK3R1 |
| **hsa05416** | Viral myocarditis | 3 | 56 | 0.0117 | HLA-DRB1,HLA-B,ACTG1 |
| **hsa03010** | Ribosome | 4 | 130 | 0.0154 | RPS7,RPS26,RPS27,RPL11 |
| **hsa04210** | Apoptosis | 4 | 135 | 0.0171 | TNFRSF1A,ATM,PIK3R1,ACTG1 |
| **hsa05211** | Renal cell carcinoma | 3 | 68 | 0.0184 | TGFB1,EP300,PIK3R1 |
| **hsa01524** | Platinum drug resistance | 3 | 70 | 0.0194 | ATM,BRCA1,PIK3R1 |
| **hsa00514** | Other types of O-glycan biosynthesis | 2 | 21 | 0.0202 | POMT2,POMT1 |
| **hsa05100** | Bacterial invasion of epithelial cells | 3 | 72 | 0.0202 | CTNNB1,PIK3R1,ACTG1 |
| **hsa01521** | EGFR tyrosine kinase inhibitor resistance | 3 | 78 | 0.0233 | EGFR,FGFR3,PIK3R1 |
| **hsa04151** | PI3K-Akt signaling pathway | 6 | 348 | 0.0233 | EGFR,COL1A2,TSC1,FGFR3,BRCA1,PIK3R1 |
| **hsa04934** | Cushing's syndrome | 4 | 153 | 0.0233 | APC,EGFR,CTNNB1,KMT2A |
| **hsa00563** | Glycosylphosphatidylinositol (GPI)-anchor biosynthesis | 2 | 25 | 0.0250 | PIGO,PIGG |
| **hsa04350** | TGF-beta signaling pathway | 3 | 83 | 0.0264 | TGFB1,EP300,BMP7 |
| **hsa04060** | Cytokine-cytokine receptor interaction | 5 | 263 | 0.0284 | TNFRSF1A,TGFB1,EDAR,EGFR,BMP7 |
| **hsa04530** | Tight junction | 4 | 167 | 0.0284 | MYH9,ACTN1,ACTG1,MYH3 |
| **hsa04658** | Th1 and Th2 cell differentiation | 3 | 88 | 0.0291 | JAG1,NOTCH1,HLA-DRB1 |
| **hsa05152** | Tuberculosis | 4 | 172 | 0.0299 | TNFRSF1A,TGFB1,EP300,HLA-DRB1 |
| **hsa05168** | Herpes simplex infection | 4 | 181 | 0.0348 | TNFRSF1A,EP300,HLA-DRB1,HLA-B |
| **hsa05203** | Viral carcinogenesis | 4 | 183 | 0.0354 | EP300,ACTN1,HLA-B,PIK3R1 |
| **hsa04066** | HIF-1 signaling pathway | 3 | 98 | 0.0360 | EP300,EGFR,PIK3R1 |
| **hsa05231** | Choline metabolism in cancer | 3 | 98 | 0.0360 | EGFR,TSC1,PIK3R1 |
| **hsa04010** | MAPK signaling pathway | 5 | 293 | 0.0377 | TNFRSF1A,TGFB1,EGFR,FGFR3,FLNB |
| **hsa05142** | Chagas disease (American trypanosomiasis) | 3 | 101 | 0.0377 | TNFRSF1A,TGFB1,PIK3R1 |
| **hsa05330** | Allograft rejection | 2 | 35 | 0.0377 | HLA-DRB1,HLA-B |
| **hsa05169** | Epstein-Barr virus infection | 4 | 194 | 0.0384 | EP300,HLA-DRB1,HLA-B,PIK3R1 |
| **hsa05332** | Graft-versus-host disease | 2 | 36 | 0.0384 | HLA-DRB1,HLA-B |
| **hsa05216** | Thyroid cancer | 2 | 37 | 0.0391 | CTNNB1,RET |
| **hsa04668** | TNF signaling pathway | 3 | 108 | 0.0408 | TNFRSF1A,JAG1,PIK3R1 |
| **hsa05145** | Toxoplasmosis | 3 | 109 | 0.0411 | TNFRSF1A,TGFB1,HLA-DRB1 |
| **hsa04940** | Type I diabetes mellitus | 2 | 40 | 0.0431 | HLA-DRB1,HLA-B |
